# Measuring the composition and availability of human resources for health by sex in 204 countries and territories, 1990–2023, and workforce gaps for universal health coverage: a systematic analysis for the Global Burden of Disease Study 2023

**DOI:** 10.1016/S2468-2667(26)00145-3

**Published:** 2026-08-18

**Authors:** GBD 2023 Human Resources for Health Collaborators, Megan Knight, Kelly Bienhoff, Bhoomadevi A, Hazim S Ababneh, Madineh Abbasi, Zahra Abbasi Dolatabadi, Mitra Abbasifard, Samar Abd ElHafeez, Ashraf Nabiel Abdalla, Reda Abdel-Hameed, Atef Abdelkader, Sherief Abd-Elsalam, Deldar Morad Abdulah, Auwal Abdullahi, Meklit Girma Abebe, Tesfaye Birhanu Abebe, Habtamu Abebe Getahun, Armita Abedi, Asrat Agalu Abejew, E S Abhilash, Alemwork Abie, Richard Gyan Aboagye, Abdullahi Tunde Aborode, Nagah M Abourashed, Mohamed Abouzid, Lucas Guimarães Abreu, Michael R M Abrigo, Dariush Abtahi, Amr Selim Abu Lila, Bilyaminu Abubakar, Rana Kamal Abu-Farha, Eman Abu-Gharbieh, Ahmad Y Abuhelwa, Hana J Abukhadijah, Anirudh Balakrishna Acharya, Krishna Prasad Acharya, Swetha Acharya, Maryam Adabi, Lisa C Adams, Nuhu Lawan Adamu, Isaac Yeboah Addo, Olusegun Adetomiwa Adediran, Kamoru Ademola Adedokun, Oluwatobi E Adegbile, Olumide Thomas Adeleke, Miracle Ayomikun Adesina, Ridwan Olamilekan Adesola, Charles Oluwaseun Adetunji, Juliana Bunmi Adetunji, Ripon Kumar Adhikary, Atman Adiba, Prince Owusu Adoma, Aanuoluwapo Adeyimika Afolabi, Muhammad Sohail Afzal, Saira Afzal, Victor Ibukun Agbajelola, Feleke Doyore Doyore Agide, Percival Delali Agordoh, César Agostinis Sobrinho, Prince Agwu, Williams Agyemang-Duah, Adamu Adamu Ahmad, Aqeel Ahmad, Danish Ahmad, Khabir Ahmad, Muayyad M Ahmad, Rabbiya Ahmad, Sajjad Ahmad, Suleiman Idris Ahmad, Ali Ahmadi, Asma Ahmed, Ayman Ahmed, Jahanzaib Mian Ahmed, Junaid Ahmed, Mehrunnisha Sharif Ahmed, Muktar Beshir Ahmed, Mushood Ahmed, Syed Anees Ahmed, Marjan Ajami, Muhammad Nadeem Akhtar, Samina Akhtar, Wole Akosile, Mohammad Khaled Al Nawayseh, Yazan Al Thaher, Omar Ali Mohammed Al Zaabi, Mohammad Ahmmad Mahmoud Al Zoubi, Tariq Al Zoubi, Timothy Olukunle Aladelusi, Fares Alahdab, Ziyad Al-Aly, Khurshid Alam, Mohammad Khursheed Alam, Mohammad Jahoor Alam, Pravej Alam, Zufishan Alam, Rasmieh Mustafa Al-Amer, Fahad Mashhour Alanezi, Turki M Alanzi, Fahmi Y Al-Ashwal, Hussin Albargi, Mohammed Albashtawy, Jacqueline Elizabeth Alcalde-Rabanal, Khalifah A Aldawsari, Mohammed S Aldossary, Robert W Aldridge, Shereen M Aleidi, Bezawit Abeje Alemayehu, Ayman Al-Eyadhy, Ali M Alfalki, Abdelazeem M Algammal, Robert Kaba Alhassan, Ashraf Alhumaidi, Irfan Ali, Kamran Ali, Liaqat Ali, Mohammad Daud Ali, Mohammed Usman Ali, Rafat Ali, Syed Shujait Ali, Akram Al-Ibraheem, Montaha Al-Iede, Esubalew Muluneh Aligaz, Hamid Alinejad Rokny, Cyrus Alinia, Morteza Alipour, Samah W Al-Jabi, Moath Saleh Aljohani, Syed Mohamed Aljunid, Wesam Taher Almagharbeh, Mohammed J Almalki, Md Al-Mamun, Sabah Al-Marwani, Joseph Uy Almazan, Mohmmad Minwer Alnaeem, Khaldoon Aied Alnawafleh, Mahmoud A Alomari, Saleh A Alqahtani, Mohammad AlQudah, Intima Alrimawi, Sahel Majed Alrousan, Ali Salman Al-Shami, Mamdouh Alshammari, Abdalkarem Fedgash Alsharari, Alaa B Al-Tammemi, Malik A Althobiani, Nelson Alvis-Guzman, Yaser Mohammed Al-Worafi, Mohammad Sharif Ibrahim Alyahya, Karem H Alzoubi, Masoud Aman Mohammadi, McKing Izeiza Amedari, Faten Amer, Amr Amin, Saeed Amini, Majid Aminzare, Sohrab Amiri, Ganiyu Adeniyi Amusa, Jimoh Amzat, Pedro Prata Andrade, Song Peng Ang, Blake Angell, Nguyen Hoang Anh, Samuel Egyakwa Ankomah, Kabilan Annadurai, Boluwatife Stephen Anuoluwa, Iyadunni Adesola Anuoluwa, Saeid Anvari, Sumadi Lukman Anwar, Razique Anwer, Anayochukwu Edward Anyasodor, Jalal Arabloo, Mosab Arafat, Aleksandr Y Aravkin, Demelash Areda, Abdulfatai Aremu, Ghazal Arjmand, Jesu Arockiaraj, Mahwish Arooj, Anton A Artamonov, Nabiy Arystan, Syed Mohammed Basheeruddin Asdaq, Saeed Asgary, Nestor Asiamah, Muhammad Shahzad Aslam, Gizew Dessie Asres, Anil Raj Assariparambil, Batyrbek Assembekov, Seyyed Shamsadin Athari, Sulaiman Olusegun Atiku, Ba-Etilayoo Atinga, Prince Atorkey, Alok Atreya, Avinash Aujayeb, Nyan Min Aung, Sana Javaid Awan, Kemal Lemnuro Awol, Camila Ospina Ayala, Martin Amogre Ayanore, Mohd Yusmaidie Aziz, Ahmed Y Azzam, Zaharaddeen Shuaibu Babandi, Fatemeh Baberi, Rasha Babiker, Abraham Samuel Babu, Syed Babar Jamal Bacha, Alaa Aboelnour Badran, Youngoh Bae, Abdul Rasheed Bahar, Awsan Abdullah Saeed Bahattab, Razieh Bahreini, Ruhai Bai, Atif Amin Baig, Lovenish Bains, Alpha Umaru Bai-Sesay, Mohamad Amin Bakhshali, Shankar M Bakkannavar, Abdulaziz T Bako, Pachamuthu Balakrishnan, Senthilkumar Balakrishnan, Wondu Feyisa Balcha, Ovidiu Constantin Baltatu, Biswajit Banik, Aleksandra Barac, Till Winfried Bärnighausen, Hiba Jawdat Barqawi, Cornelia Anne Barth, Zarrin Basharat, Mohammad-Mahdi Bastan, Abdul-Monim Batiha, Kavita Batra, Samuel Debas Bayable, Mulat Tirfie Bayih, Zemenu Wube Bayleyegn, Narasimha M Beeraka, Diana Fernanda Bejarano Ramirez, Bekalu Mekonen Belay, Asnake Gashaw Belayneh, Aminu K Bello, Samiun Nazrin Bente Kamal Tune, Kelemu Zelalem Berhanu, Robert S Bernstein, Nikha Bhardwaj, Pankaj Bhardwaj, Sonu Bhaskar, Priyadarshini Bhattacharjee, Gurjit Kaur Bhatti, Jasvinder Singh Bhatti, Rajbir Bhatti, Zulfiqar A Bhutta, Sibhatu Kassa Biadgilign, Bijit Biswas, Mohammad Shahangir Biswas, Fassikaw Kebede Bizuneh, Micheal Kofi Boachie, Trupti Bodhare, Somayeh Bohlouli, Lucimere Bohn, Aime Bonny, Sudipta Bose, Souad Bouaoud, Dejana Braithwaite, Datonye Christopher Briggs, Ovidiu-Iulian Bunea, Felix Busch, Reinhard Busse, Yasser Bustanji, Zahid A Butt, Jack Cagney, Ismael Campos-Nonato, Si Cao, Yu Cao, Mate Car, Rosario Cárdenas, Andre F Carvalho, Felix Carvalho, Alberico L Catapano, Pamela Roxana Chacón-Uscamaita, Rama Mohan Chandika, Miyuru Chandradasa, Viswa Chaitanya Chandu, Jung-Chen Chang, Qinghua Chang, Vijay Kumar Chattu, Lam Duc Chau, Anis Ahmad Chaudhary, Sirshendu Chaudhuri, Akhilanand Chaurasia, Che Mohd Nasril Che Mohd Nassir, Fentahun Alemnew Chekole, Haowei Chen, Mingling Chen, Yun-Jung Choi, Bryan Chong, Hitesh Chopra, Shivani Chopra, Sonali Gajanan Choudhari, Dinh-Toi Chu, Jinky Marie T Chua, Isaac Sunday Chukwu, Sunghyun Chung, Arrigo Francesco Giuseppe Cicero, Fred Cohen, Joao Conde, Claudia Cosma, Natalia Cruz-Martins, Ali Dabbagh, Omid Dadras, Bishal Dahal Khatri, Xiaochen Dai, Maxwell Ayindenaba Dalaba, Mayank Dalakoti, Emanuele D’Amico, Anh Kim Dang, Samuel Demissie Darcho, Reza Darvishi Cheshmeh Soltani, Mauro Henrique Nogueira Guimarães de Abreu, Jan-Walter De Neve, Ivan Delgado-Enciso, Endalkachew Dellie, Dessalegn Demeke, Ismail Dergaa, Nikolaos Dervenis, Muamer Dervišević, Aragaw Tesfaw Desale, Vinoth Gnana Chellaiyan Devanbu, Syed Masudur Rahman Dewan, Thakur Dhakal, Rajinder K Dhamija, Amol S Dhane, Sreedhar Dharmagadda, Marcello Di Pumpo, Adriana Dima, Delaney D Ding, Isaac Oluwafemi Dipeolu, Huyen Phuc Do, Mai Ngoc Do, Mario D’Oria, Fariba Dorostkar, Ojas Prakashbhai Doshi, Robert Kokou Dowou, Emeka W Dumbili, Senbagam Duraisamy, Oyewole Christopher Durojaiye, Anar Dushpanova, Ejemai Eboreime, Ikenna D Ebuenyi, Afework Edmealem, David Edvardsson, Ferry Efendi, Abiodun Egwuenu, Michael Ekholuenetale, Rabie Adel El Arab, Mohamed Ahmed Eladl, Muhammed Elhadi, Yasir Ahmed Mohammed Elhadi, Salem Elkahoui, Randa Elsheikh, Chadi Eltaha, Stanley Chinedu Eneh, Majid Eslami, Fahima Nasrin Eva, Elochukwu Ezenwankwo, Adewale Oluwaseun Fadaka, Heidar Fadavian, Adeniyi Francis Fagbamigbe, Ayesha Fahim, Ildar Ravisovich Fakhradiyev, Mohammad Fareed, Aisha Farhana, Liliana Faria, Andre Faro, Fatemeh Farshad, Folorunso Oludayo Fasina, Davood Fathi, Ginenus Fekadu, Abdullah H Feroze, Pietro Ferrara, Marta Figueiredo, Safia Firdous, Florian Fischer, Federica Fogacci, Artem Alekseevich Fomenkov, Arianna Fornari, Alina-Ioana Forray, Alberto Freitas, Takeshi Fukumoto, Nancy Fullman, Adam Fusheini, Peter Andras Gaal, Muktar A Gadanya, Dominic Dormenyo Gadeka, Anna D Gage, Márió Gajdács, Balasubramanian Ganesh, Ezhilkugan Ganessane, Fernando Jr Barroga Garcia, Chandi Garg, Jacopo Garlasco, Rupesh K Gautam, Tsion S Gebre, Beza A Gelaw, Stefano Gelibter, Julian Lassiter Gendreau, Kabiru Ishola Genty, Gebremariam Wulie Geremew, Genanew Kassie Getahun, Kalab Yigermal Gete, Mustafa Ghaderzadeh, Fataneh Ghadirian, Kazem Ghaffari, Leila Ghalichi, Arin Ghamkhar, Ali Ghandili, Ahmad Ghashghaee, Sailaja Ghimire, Ali Gholamrezanezhad, Vaitsa Giannouli, Artyom Urievich Gil, Syed Abdullah Gilani, Bikash Ranjan Giri, Alem Abera Girmay, Mahaveer Golechha, Pouya Goleij, Davide Golinelli, Melika Golmohammadi, Yitayal Ayalew Goshu, Barbara Niegia Garcia Goulart, Tulasi Govardhan, Ayman Grada, Giovanni Guarducci, Stefano Guicciardi, Sheffali Gulati, Kabiru Abubakar Gulma, Damitha Asanga Gunawardane, Lei Guo, Aayush Gupta, Bhawna Gupta, Rajeev Gupta, Sapna Gupta, Vijai Kumar Gupta, Roberth Steven Gutiérrez-Murillo, Jose Guzman-Esquivel, Farrokh Habibzadeh, Zahra Hadian, Khalid Rehman Rehman Hakeem, Alex Hakuzimana, Pritam Halder, Kosar Hikmat Hama Aziz, Ayman M Hamdan-Mansour, Mohamed Hamed, Parvaneh Hamian Roumiani, Samer Hamidi, Asif Hanif, Mohd Anul Haq, Josep Maria Haro, Mohammad Jahid Hasan, S M Mahmudul Hasan, Towhid Hasan, Ali Hasanpour- Dehkordi, Arezou Hashem Zadeh, Ahmad Shoeb Hashmi, Md Saquib Hasnain, Amr Hassan, Ikrama Hassan, Md Imtaiyaz Hassan, Simon I Hay, Ahmad Hayatu, Mohammad Heidari, Majid Heydari, Thomas Kwadwo Hinneh, Yuta Hiraike, Ramesh Holla, Yu Hong, Mehdi Hoseinzadeh, Lubna Hossain, Md Mahbub Hossain, Mohammad Bellal Hossain, Mihaela Hostiuc, Sorin Hostiuc, Guoqing Hu, Junjie Huang, Yongsong Huang, Ayesha Humayun, Javid Hussain, Olayemi Joshua Ibidoja, Aminu Alhassan Ibrahim, Umar Idris Ibrahim, Anel Ibrayeva, Pulwasha Maria Iftikhar, Aqsa Ikram, Jibran Ikram, Olayinka Stephen Ilesanmi, Irena M Ilic, Milena D Ilic, Mohammad Tarique Imam, Arit Inok, Meesha Iqbal, Mujahid Iqbal, Usman Iqbal, Lalu Muhammad Irham, Fahrul Islam, Md Rabiul Islam, Md Sahidul Islam, Md Shahinul Islam, Nahlah Elkudssiah Ismail, Gaetano Isola, Yussif Issaka, Masao Iwagami, Chidozie Declan Iwu, Jaison Jacob, Louis Jacob, Udeme Samuel Jacob, Ali Jadidi, Shabbar Jaffar, Nader Jahanmehr, Haitham Jahrami, Mihajlo Jakovljevic, Reza Jalilzadeh Yengejeh, Armaan Jamal, Anju James, Jerin James, Masoud Jamshidi, Rajiv Janardhanan, Mu’taman Jarrar, Krishna Mahendrabhai Jasani, Talha Jawaid, Sathish Kumar Jayapal, Shubha Jayaram, Yovanthi Anurangi Jayasinghe, Jae Joon Jeon, Seogsong Jeong, Worku Jimma, Mohammad Jokar, Jobinse Jose, Akaninyene Paul Joseph, Nitin Joseph, Charity Ehimwenma Joshua, Farahnaz Joukar, Jacek Jerzy Jozwiak, Sang-Hyuk Jung, Malik E Juweid, Hema Shree K, Dler Hussein Kadir, Adel Kadri, Ashish Kumar Kakkar, Khalil Kalavani, Feroze Kaliyadan, Aidana Kaliyakparova, Sanjay Kalra, Rajesh Kamath, Ramat T Kamorudeen, Hesam Kamyab, Jiseung Kang, Kehinde Kazeem Kanmodi, Rami S Kantar, Mehrdad Karajizadeh, Paschalis Karakasis, Jafar Karami, Marina Karanikolos, Mohmed Isaqali Karobari, Faizan Zaffar Kashoo, Nehemia I Kassa, Nicholas J Kassebaum, Kanica Kaushal, Foad Kazemi, Vikash Ranjan Keshri, Aïmen Khacharem, Yousef Saleh Khader, Himanshu Khajuria, Asaad Khalid, Anees Ahmed Khalil, Ajmal Khan, Faiz Ullah Khan, Iqra Hamid Khan, MD Sazzad Khan, Mohd Faheem Khan, Muhammad Arslan Khan, Muhammad Umer Khan, Ramsha Mushtaq Khan, Serab Khan, Sumaiya Khan, Yusuf Saleem Khan, Zahid Khan, Sameer Uttamaro Khasbage, Khaled Khatab, Haitham Khatatbeh, Moawiah Mohammad Khatatbeh, Hamid Reza Khayat Kashani, Afshin Khazaei, Khalid A Kheirallah, Zahra Khorrami, Parisa Khoshvaght, Mahmood Khosrowjerdi, Jinho Kim, Kwanghyun Kim, Min Seo Kim, Ruth W Kimokoti, Adnan Kisa, Shivakumar KM Kondlahalli, Sonali Kochhar, Prakash Babu Kodali, Michail Kokkorakis, Ali-Asghar Kolahi, Farzad Kompani, Tapos Kormoker, Oleksii Korzh, Michal Koscik, Archana Koul, Sindhura Lakshmi Koulmane Laxminarayana, Arthi S Kozhumam, Irene Akwo Kretchy, Kewal Krishan, Bindu Krishnan, Mohammed Kuddus, Shikha Kukreti, Mukhtar Kulimbet, Emmanuel Kumah, Chandan Kumar, Dewesh Kumar, Kamal Kumar, Laksh Kumar, Manasi Kumar, Mukesh Kumar, Nitesh Kumar, Pradeep Kumar, Rakesh Kumar, Sandeep Kumar, Sanjeet Kumar, Vijay Kumar, Senthil Kumaran D, Satyajit Kundu, Jibin Kunjavara, Almagul Kurmanova, Donata Kurpas, Christina Yeni Kustanti, Dian Kusuma, Tezer Kutluk, Assylkhan Kuttybayev, Michael Agyemang Kwarteng, Ville Kytö, Muhammad Awwal Ladan, Desireé Alexa LaGrappe, Chandrakant Lahariya, Daphne Teck Ching Lai, Hanpeng Lai, Dharmesh Kumar Lal, Judit Lám, Ariane Laplante-Lévesque, Anders O Larsson, Savita Lasrado, Areeba Latif, Basira Kankia Lawal, Eilean Rathinasamy Lazarus, Caterina Ledda, Munjae Lee, Sergey Vadimovich Lee, Seung Won Lee, Wei-Chen Lee, Yo Han Lee, Vasileios Leivaditis, Francesco Leonforte, Chengfeng Li, Honghong Li, Ming-Chieh Li, Shaojie Li, Virendra S Ligade, Jialing Lin, Jue Liu, Xianliang Liu, Zihao Liu, Madeeha Shahzad Lodhi, Niroshan Chandrajith Lokunarangoda, Jaifred Christian F Lopez, José Francisco López-Gil, Arianna Maever Loreche, Masoud Lotfizadeh, Surbala Devi Lourembam, Giancarlo Lucchetti, Hengliang Lv, Miltiadis D Lytras, Ellina Lytvyak, Hawraz Ibrahim M Amin, Kevin Sheng-Kai Ma, Monika Machoy-Rakoczy, Samatar Abshir Mahamed, Nozad Hussein Mahmood, Shakeel Ahmed Ibne Mahmood, Kafayat Mahmoud, Hao Xuan Mai, Ghaseb Naser Makhadmeh, Irsa Fatima Makhdoom, Ahmad Azam Malik, Kamaruddeen Mannethodi, Marjan Mansourian, Tahir Maqbool, Hamed Markazi Moghadam, Adolfo Martinez-Valle, J Jenifer Florence Mary, Roy Rillera Marzo, Sammer Marzouk, Yasith Mathangasinghe, Manu Raj Mathur, Neeta Mathur, Fernanda Penido Matozinhos, Khurshid A Mattoo, Richard James Maude, Suleiman Mayaki, Maryam Mazaheri, Martin McKee, Steven M McPhail, Ravi Mehrotra, Vini Mehta, Tadesse Belayneh Melkie, Ziad Ahmed Memish, Godfred Antony Menezes, Emiru Ayalew Mengistie, Leweyehu Alemaw Mengstie, Atte Meretoja, Tuomo J Meretoja, Giovanni Merlino, Muayad Aghali Merza, Tomislav Mestrovic, Chamila Dinushi Kukulege Mettananda, Sachith Mettananda, Mohamed M M Metwally, Sandrine Donfack D Mewoabi, Muhammad Agus Mikrajab, Giuseppe Minervini, Wai-Kit Ming, GK Mini, Andreea Mirica, Erkin M Mirrakhimov, Vinaytosh Mishra, Chaitanya Mittal, Dhruti Modi, Mona Gamal Mohamed, Nouh Saad Mohamed, Khabab Abbasher Hussien Mohamed Ahmed, Taj Mohammad, Sakineh Mohammad-Alizadeh-Charandabi, Omer Mohammed, Shafiu Mohammed, Syam Mohan, Yugal Kishore Mohanta, Siba Sankar Mohanty, Ali H Mokdad, TJ Robinson T Moncatar, Himel Mondal, Mohammad Ali Moni, Marco Montalti, Mohammad Moradi-Joo, Danilo Farias Morais, Shane Douglas Morrison, Reza Mosaddeghi Heris, Freiser Eceomo Cruz Mosquera, Elias Mossialos, Kimia Mozahheb Yousefi, Nicollas Mozart Vieira, Matías Mrejen, Rabia Mubarak, Sumaira Mubarik, Jibran Sualeh Muhammad, Amartya Mukhopadhyay, Joshua Kanaabi Muliira, Francesk Mulita, Kavita Munjal, Sani Musa, David Musoke, Ghulam Mustafa, Mubarak Taiwo Mustapha, Sathish Muthu, Claude Mambo Muvunyi, Lutfi Muzaqi, Woojae Myung, Ayoub Nafei, Mobin Naghshbandi, Hiten Naik, Firzan Nainu, Hastyar Hama Rashid Najmuldeen, Noureddin Nakhostin Ansari, Gopal Nambi, Abdulqadir J Nashwan, Mahmoud Nassar, Samidi Nirasha Kumari Navaratna, Biswa Prakash Nayak, Kinjal Nayak, Vinod C Nayak, Javad Nazari, Mojdeh Nazari, Ionut Negoi, Ruxandra Irina Negoi, Alina Gabriela Negru, Chakib Nejjari, Nikita Nekliudov, Cuong Tat Nguyen, Hien Thu Nguyen, Huong Lan Thi Nguyen, Tham Thi Nguyen, Trang Nguyen, Yeshambel T Nigatu, Ali Nikoobar, Shuhei Nomura, Mamoona Noreen, Emira Noumi, Mohammed Noushad, Chisom Adaobi Nri-Ezedi, Fred Nugen, Bogdan Oancea, Akinyemi O D Ofakunrin, Oluwakemi Christie Ogidan, Ayomide Samuel Ogunrinde, Olusegun Olatunji Ojedoyin, Olalekan John Okesanya, Osaretin Christabel Okonji, John Olayemi Okunlola, Oluwaseyi Isaiah Olabisi, Musa Kolapo Oladejo, Andrew T Olagunju, Oladotun Victor Olalusi, Abimbola Olaniran, Gláucia Maria Moraes Oliveira, Abdulhakeem Abayomi Olorukooba, Bolajoko Olubukunola Olusanya, Oluwafemi G Oluwole, Ahmed Omar Bali, Obinna E Onwujekwe, Aksoltan Shyhdurdyevna Oradova, Michal Ordak, Verner N Orish, Atakan Orscelik, Esteban Ortiz-Prado, Godwin Osawaru Osaigbovo, Augustus Osborne, George Nkrumah Osei, Godfred Otchere, Adrian Otoiu, Jerry John Ouner, Amel Ouyahia, Simon Øverland, Mayowa O Owolabi, Oladayo Ayobami Oyebanji, Kehinde Adewole Oyeniran, Ilker Ozsahin, Jagadish Rao Padubidri, Vijayakumar Palaniswamy, Tamás Palicz, Adrian Pana, Sujogya Kumar Panda, Bipindra Pandey, Prateek Pandya, Santa Maria Pangaribuan, Anca Pantea Stoian, Ilias Papadimopoulos, Shahina Pardhan, Romil R Parikh, Chulwoo Park, Arpit Parmar, Maja Pasovic, Roberto Passera, Mitesh Patel, Neel Navinkumar Patel, Shankargouda Patil, Dimitrios Patoulias, Apurba Patra, Snigdha Pattnaik, Uttam Paudel, Shrikant Pawar, Hamidreza Pazoki Toroudi, Paolo Pedersini, Veincent Christian Filipino Pepito, Prince Peprah, Maria Odete Pereira, Mario F P Peres, Simone Perna, Olumuyiwa James Peter, Tung Thanh Pham, Michael R Phillips, Zahra Zahid Piracha, Edoardo Pirera, Mapa M Prabhath N Piyasena, Evgenii Plotnikov, Dimitri Poddighe, Ion Popa, Fabio Porru, Sajjad Pourasghary, Mohsen Poursadeqiyan, Manya Prasad, Akila Prashant, Elton Junio Sady Prates, Harsh Priya, Nicola Riccardo Pugliese, Jagadeesh Puvvula, Muhammad Usman Qamar, Nameer Hashim Qasim, Xiang Qi, Navid Rabiee, Reza Rabiei, Venkatraman Radhakrishnan, Pracheth Raghuveer, Sajjad Rahimi, Afarin Rahimi-Movaghar, Vafa Rahimi-Movaghar, Firman Suryadi Rahman, Fryad Majeed Rahman, Mahbubur Rahman, Mosiur Rahman, Muhammad Aziz Rahman, Pramila Rai, Adarsh Raja, Ramya Raja Reddy, Judah Rajendran, Kairolla Dyusenbayevich Rakhimov, Rafał Rakoczy, Shakthi Kumaran Ramasamy, Pramod W Ramteke, Lokesh Rana, Nemanja Rancic, Manjula Rangappa, Magesh Rangasamy, Chythra R Rao, Kumuda Rao, Mithun Rao, Sowmya J Rao, Devarajan Rathish, Jigyasa Rathore, Ramin Ravangard, Rashita Ravi, Renju Ravi, David Laith Rawaf, Salman Rawaf, Lal Rawal, Ramu Rawat, Ayita Ray, Muhammad Liaquat Raza, Bahman Razi, C Mahony Reategui-Rivera, Najeeb Ur Rehman, Lennart Reifels, Bhagwan Narayan Rekadwad, Serge Resnikoff, Stefano Restaino, Luis Felipe Reyes, Nazila Rezaei, Nima Rezaei, Mohsen Rezaeian, Muhammad Riaz, Jennifer Rickard, Syed Mohd Danish Rizvi, Jefferson Antonio Buendia Rodriguez, Leonardo Roever, Ravi Rohilla, Moustaq Karim Khan Rony, Kevin T Root, Himanshu Sekhar Rout, Adrija Roy, Gaurav Kumar Roy, Sharmistha Roy, Godfrey Mutashambara Rwegerera, Chandan S N, Aly M A Saad, Korosh Saber, Siamak Sabour, Bashdar Abuzed Sadee, Fatemeh Sadeghi-Ghyassi, Hossein Sadr, Mohammad Reza Saeb, Umar Saeed, Maryam Saeedi, Sahar Saeedi Moghaddam, Muhammad Safiullah, Rajesh Sagar, Amene Saghazadeh, Indranil Saha, Fatemeh Saheb Sharif-Askari, Narjes Saheb Sharif-Askari, Vaibhav Sahni, Ambika Sahoo, Kirti Sundar Sahu, S Mohammad Sajadi, Mirza Rizwan Sajid, Joseph W Sakshaug, Afeez Abolarinwa Salami, Bukola Salami, Samreen Saleem, Mohamed A Saleh, Aanuoluwa James Salemcity, Pegah Salimi Pormehr, Giovanni A Salum, Hossein Samadi Kafil, Janmejaya Samal, Abdallah M Samy, Elaheh Sanjari, Sathish Sankar, Lucas H C C Santos, Milena M Santric-Milicevic, Malik Adebayo Sanusi, Sivan Yegnanarayana Iyer Saraswathy, Yaser Sarikhani, Hemen Sarma, Mohammad Sarmadi, Gargi Sachin Sarode, Sachin C Sarode, Michele Sassano, Mukesh Kumar Sathya Narayanan, Maheswar Satpathy, Amar Saurabh, Sangeeta Gopal Saxena, Yaser Sayadi, Ahmed M Sayed, Christophe Schinckus, Soraya Seedat, Durairaj Sekar, Chandrabose Selvaraj, Siddharthan Selvaraj, Yuliya Semenova, Fikadu Waltengus Sendeku, Subramanian Senthilkumaran, Sadaf G Sepanlou, Edson Serván-Mori, Francesco Sessa, Yashendra Sethi, Abubakar Sha’aban, Shipraa Arpan Shah, Sweni Shah, Muhammad Shahab, Samiah Shahid, Wajeehah Shahid, Endrit Shahini, Farshad Shahkarami, Ali Shahriari, Moyad Jamal Shahwan, Babar Tasneem Shaikh, Masood Ali Shaikh, Ali Shakerimoghaddam, Alfiya Shamsutdinova, Dan Shan, Mohd Shanawaz, Nadim Sharif, Amin Sharifan, Prof Fariborz Sharifian Jazi, Avimanu Sharma, Bhoopesh Kumar Sharma, Jyoti Sharma, Kamal Sharma, Kamlesh Sharma, Manoj Sharma, Vishal Sharma, Ramzi Shawahna, Anas Shehadeh, Somia Shehzadi, Ali Sheidaei, Rekha Raghuveer Shenoy, Suchitra M Shenoy, Samendra P Sherchan, Mahabalesh Shetty, Pavanchand Shetty, Premalatha K Shetty, Muhammad J A Shiddiky, Tariku Shimels, Md Monir Hossain Shimul, Priyanka Arun Shirali, Shirindokht Shirazi, Aminu Shittu, Km Shivangi, Azad Shokri, Sina Shool, Seyed Afshin Shorofi, Sunil Shrestha, Suleiman Adeiza Shuaibu, Akhenaten Benjamin Siankam Tankwanchi, Negussie Boti Sidamo, Emmanuel Edwar Siddig, Arif J Siddiqui, Gustavo Correia Basto da Silva, Padam Prasad Simkhada, David Larbi Simpong, Colin R Simpson, Abhinav Singh, Baljinder Singh, Bhoopendra Singh, Brij Pal Singh, Harmanjit Singh, Jasvinder A Singh, Jawahar Singh, Mayank Singh, Puneetpal Singh, Shilpy Singh, Siddharth Singh, Mukesh Kumar Sinha, Ratnesh Sinha, Gisha Sivan, Natia Skhvitaridze, Valentin Yurievich Skryabin, Mejdi Snoussi, Farrukh Sobia, Md Salman Sohel, Solikhah Solikhah, Jukun Song, Michael Spartalis, Lokasani Vijaya Sree, Chandrashekhar T Sreeramareddy, Shyamkumar Sriram, Panagiotis Stachteas, Simona Cătălina Ștefan, Brendon Stubbs, Peter Stubbs, Chen-Yang Su, Omer Subasi, Vetriselvan Subramaniyan, Aswin Sugunan, Mahwish Suhaib, Oksana Sulaieva, Sahabi K Sulaiman, Mark J M Sullman, Yan Sun, Zhong Sun, Thanigaivel Sundaram, David Sunkersing, Chandan Kumar Swain, Shreya P Swami, Lukasz Szarpak, Payam Tabaee Damavandi, Rafael Tabarés-Seisdedos, Seyed-Amir Tabatabaeizadeh, Shima Tabatabai, Ramin Tabibi, Mohd Tabish, Biniyam Beyene Tabor, Zanan Mohammed-Ameen Taha, Niloofar Taheri, Jabeen Taiba, Mircea Tampa, Jianye Tan, Ker-Kan Tan, Baimakhan Tanabayev, Dariga Tanabayeva, Shynar Tanabayeva, Shenglan Tang, Ekamol Tantisattamo, Mohsan Tanveer, Hongliang Tao, Anika Tasnim, Nathan Y Tat, Vivian Y Tat, Aigul Yelgondiyevna Tazhiyeva, Mohamad-Hani Temsah, Wegen Beyene Tesfamariam, Azimeraw Arega Tesfu, Pugazhenthan Thangaraju, Kavumpurathu Raman Thankappan, Ismaeel Tharwat, Rasiah Thayakaran, Jeevarathinam Thirumalai, Muthu Thiruvengadam, Rekha Thiruvengadam, Jansje Henny Vera Ticoalu, Mariya Vladimirovna Titova, Vikas Kumar Tiwari, Madi Tleshev, Santo Imanuel Tonapa, Roman Topor-Madry, Stephanie M Topp, Marcos Roberto Tovani-Palone, Quynh Thuy Huong Tran, Thang Huu Tran, Nguyen Tran Minh Duc, Domenico Trico, Quynh Xuan Nguyen Truong, Gary Tse, Vasilis-Spyridon Tseriotis, Evangelia Tsermpini, Lorainne Tudor Car, Atta Ullah, Riaz Ullah, Saeed Ullah, Shehu Salihu Umar, Era Upadhyay, Jibrin Sammani Usman, Dilber Uzun Ozsahin, Hande Uzunçıbuk, Pascual R Valdez, Sven Vanneste, Joe Varghese, Santosh Varughese, Tommi Juhani Vasankari, Anoop Velayudhan, Balachandar Vellingiri, U Venkatesh, Narayanaswamy Venketasubramanian, David Villarreal-Zegarra, Tuan Vinh, Luciano Magalhães Vitorino, Vasily Vlassov, Ave Voit, Simona Ruxandra Volovat, Linh Vu, Yasir Waheed, Ethan Waisberg, Eric Lee Wan, Jin-Yi Wan, Haozhen Wang, Shaopan Wang, Shu Wang, Wanzhou Wang, Wei Wang, Xuequan Wang, Yan Wang, Yanzhong Wang, Youxin Wang, Mary Njeri Wanjau, Paul R Ward, Wesley Warriner, Toyiba Hiyaru Wassie, Anggi Lukman Wicaksana, Nuwan Darshana Wickramasinghe, Samuel Wiebe, Angga Wilandika, Martin Wiredu Agyekum, Axel Walter Wolf, Zhijia Xia, Wanqing Xie, Saba Yahoo, Haibo Yang, Yuichiro Yano, Haiqiang Yao, Haya Khader Ahmad Yasin, Yuichi Yasufuku, Sanni Yaya, Kuanysh A Yergaliyev, Subah Abderehim Yesuf, Saber Yezli, Xinglin Yi, Dehui Yin, Yulai Yin, Yazachew Engida Yismaw, Dong Keon Yon, Naohiro Yonemoto, Mustafa Younis, Hairui Yu, Yong Yu, Quan Yuan, Ghazala Yunus, Siddhesh Zadey, Manijeh Zaghampour, Dilmurat Zairov, Nelson Zamora, Aurora Zanghì, Kourosh Zarea, Ahmad Zarei, Michael Zastrozhin, Saad Zbiri, Mohammed G M Zeariya, Zahra Zeinali, Ruba A Zenati, Eyael M Zeru, Julio Min Fei Zhang, Chenyu Zhao, Yingxi Zhao, Jinxin Zheng, Anthony Zhong, Hao Zhou, Bin Zhu, Zhaohua Zhu, Abzal Zhumagaliuly, Mohamed Ali Zoromba, Alimuddin Zumla, Ahed H Zyoud, Sa’ed H Zyoud, Shaher H Zyoud, Enis Baris, Rafael Lozano, Christopher J L Murray, Annie Haakenstad

**Affiliations:** a Institute for Health Metrics and Evaluation, University of Washington, Seattle, WA, USA; b Amity Institute of Public Health and Hospital administration, Amity University Noida, Uttar Pradesh, India; c Department of Radiation Oncology, Massachusetts General Hospital, Boston, MA, USA; d Infectious and Tropical Research Center, Tabriz University of Medical Sciences, Tabriz, Iran; e Department of Medical-surgical Nursing, Tehran University of Medical Sciences, Tehran, Iran; f Department of Internal Medicine, Rafsanjan University of Medical Sciences, Rafsanjan, Iran; g Clinical Research Development Unit, Rafsanjan University of Medical Sciences, Rafsanjan, Iran; h Department of Epidemiology, Alexandria University, Alexandria, Egypt; i College of Pharmacy, Umm Al-qura University, Makka, Saudi Arabia; j Basic Science Department, University of Ha’il, Hail, Saudi Arabia; k Chemistry Department, Al-Azhar University, Cairo, Egypt; l Department of Mathematics and Sciences, Ajman University, Ajman, United Arab Emirates; m Department of Tropical Medicine and Infectious Diseases, Tanta University, Egypt, Tanta, Egypt; n Community and Maternity Nursing Unit, University of Duhok, Duhok, Iraq; o Department of Physiotherapy, Bayero University Kano, Kano, Nigeria; p Department of Physiotherapy, Federal University Wukari, Wukari, Nigeria; q Department of Pediatrics and Child Health, Landmark General Hospital, Addis Ababa, Ethiopia; r School of Medicine, Salale University, Fitche, Ethiopia; s Department of Epidemiology and Biostatistics, University of Gondar, Gondar, Ethiopia; t Department of Emergency Medicine, Zanjan University of Medical Sciences, Zanjan, Iran; u School of Pharmacy, Bahir Dar University, Bahir Dar, Ethiopia; v Department of Botany, Sree Narayana Guru College Chelannur, Kozhikode, India; w Department of Midwifery, Bahir Dar University, Bahir Dar, Ethiopia; x Department of Family and Community Health, University of Health and Allied Sciences, Ho, Ghana; y School of Population Health, University of New South Wales, Sydney, NSW, Australia; z Department of Research and Development, Healthy Africans Platform, Ibadan, Nigeria; aa Zoology Department, Benha University, Benha 13518, Egypt; ab Department of Physical Pharmacy and Pharmacokinetics, Poznan University of Medical Sciences, Poznan, Poland; ac Department of Pediatric Dentistry, Federal University of Minas Gerais, Belo Horizonte, Brazil; ad Philippine Institute for Development Studies, Quezon City, Philippines; ae Department of Anesthesiology, Shahid Beheshti University of Medical Sciences, Tehran, Iran; af Department of Pharmaceutics, University of Hail, Hail, Saudi Arabia; ag Department of Pharmacology and Toxicology, Usmanu Danfodiyo University, Sokoto, Sokoto, Nigeria; ah Nigerian Institute of Medical Research, Lagos, Nigeria; ai Clinical Pharmacy and Therapeutics Department, Faculty of Pharmacy, Applied Science Private University, Amman, Jordan; aj Clinical Sciences Department, University of Sharjah, Sharjah, United Arab Emirates; ak Department of Biopharmaceutics and Clinical Pharmacy, University of Jordan, Amman, Jordan; al Department Pharmacy Practice and Pharmacotherapeutics, University of Sharjah, Sharjah, United Arab Emirates; am Clinical Trials Unit, Hamad Medical Corporation, Doha, Qatar; an Department of Restorative Dentistry, University of Sharjah, Sharjah, United Arab Emirates; ao Department of Livestock Services (DLS), Government of Nepal (GoN), Lalitpur, Nepal; ap Department of Oral Pathology and Microbiology, JSS Academy of Higher Education and Research, Mysuru, India; aq Research Center for Food Hygiene and Safety, School of Public Health, Shahid Sadoughi University of Medical Sciences, Yazd, Iran, Shahid Sadoughi University of Medical Sciences, Yazd, Iran; ar Department of Diagnostic and Interventional Radiology, Technical University of Munich, Munich, Germany; as Stanford University, Palo Alto, CA, USA; at Department of Nursing Science, Modibbo Adama University, Yola., Yola, Nigeria; au School of Medicine, University of Sydney, Sydney, NSW, Australia; av Centre for Social Research in Health, University of New South Wales, Sydney, NSW, Australia; aw Department of Ophthalmology, Faculty of Clinical Sciences, College of Medicine, University of Ibadan, Ibadan, Nigeria; ax Department of Ophthalmology, University College Hospital, Ibadan, Ibadan, Nigeria; ay Department of Immunology, Roswell Park Comprehensive Cancer Center, Buffalo, NY, USA; az Graduate Program Division, University at Buffalo, Buffalo, NY, USA; ba Center for Cardiovascular Risk Research, East Tennessee State University, Johnson City, TN, USA; bb Center for Disease Control and Prevention, Cephas Health Research Initiative Inc., Ilesa, Nigeria; bc Department of Family Medicine, Bowen University, Iwo, Nigeria; bd Department of Family Medicine, Bowen University Teaching Hospital, Ogbomoso, Nigeria; be Slum and Rural Health Initiative Research Academy, Slum and Rural Health Initiative, Ibadan, Nigeria; bf Department of Physiotherapy, University of Ibadan, Ibadan, Nigeria; bg Department of Veterinary Medicine and Surgery, University of Missouri, Columbia, MO, USA; bh Department of Microbiology, Edo State University Uzairue, Iyamho, Nigeria; bi Department of Biochemistry, Osun State University, Osogbo, Nigeria; bj Department of Fisheries and Marine Bioscience, Jashore University of Science and Technology, Jashore, Bangladesh; bk Research School of Population Health, Australian National University, Canberra, ACT, Australia; bl Department of Biotechnology, National Institute for Agronomic Research, Morocco, Beni Mellal, Morocco; bm Department of Health Administration and Education, University of Education Winneba, Winneba, Ghana; bn Technical Services Directorate, MSI Nigeria Reproductive Choices, Abuja, Nigeria; bo Department of Life Sciences, University of Management and Technology, Lahore, Pakistan; bp Department of Community Medicine, King Edward Memorial Hospital, Lahore, Pakistan; bq Department of Public Health, Public Health Institute, Lahore, Pakistan; br Department of Pathobiology and Intergrative Biomedical Sciences, University of Missouri, Columbia, MO, USA; bs Department of Public Health, Kampala International University, Kampala, Uganda; bt Department of Nutrition and Dietetics, University of Health and Allied Sciences, Ho, Ghana; bu SPRINT Sport Physical Activity and Health Research & Innovation Center, Polytechnic Institute of Guarda, Guarda, Portugal; bv Health Research and Innovation Sciences Center, Polytechnic Institute of Guarda, Klaipeda, Lithuania; bw University of Dundee, University of Dundee, Dundee, UK; bx Health Policy Research Group, University of Nigeria, Enugu, Nigeria; by Department of Public Health Sciences, Queen’s University, Kingston, ON, Canada; bz Department of Physical Therapy, University of Nizwa, Birkat Al Mawz, Oman; ca College of Medicine, Shaqra University, Shaqra, Saudi Arabia; cb School of Medicine and Psychology, Australian National University, Canberra, ACT, Australia; cc Health Research Institute, University of Canberra, Canberra, NSW, Australia; cd King Khaled Eye Specialist Hospital and Research Center, Riyadh, Saudi Arabia; ce School of Nursing, University of Jordan, Amman, Jordan; cf Department of Clinical Pharmacy, Universiti Sains Malaysia, Penang, Malaysia; cg Department of Pharmacy, The University of Lahore, Lahore, Pakistan; ch Department of Health and Biological Sciences, Abasyn University, Peshawar, Pakistan; ci Department of Natural Sciences, Lebanese American University, Beirut, Lebanon; cj Vaccines and Immunity Department, Medical Research Council Unit, The Gambia, Fajara, The Gambia; ck Department of Epidemiology and Biostatistics, Shahrekord University of Medical Sciences, Shahrekord, Iran; cl Department of Epidemiology, Shahid Beheshti University of Medical Sciences, Tehran, Iran; cm Institute of Molecular Biology and Biotechnology, The University of Lahore, Lahore, Pakistan; cn INTI International University Malaysia, Nilai, Malaysia; co Institute of Endemic Diseases, University of Khartoum, Khartoum, Sudan; cp Pan-African One Health Institute (PAOHI), Kigali, Rwanda; cq Health Department, Sandeman Provincial Hospital, Quetta, Pakistan; cr Faculty of Pharmaceuticals and Allied Health Sciences, University of Balochistan Quetta, Quetta, Pakistan; cs Manipal College of Dental Sciences,Mangalore, Manipal Academy of Higher Education, Mangalore, India; ct College of Nursing, Majmaah University, Al Majmaah, Saudi Arabia; cu College of Medicine and Public Health, Flinders University, Adelaide, SA, Australia; cv Faculty of Public Health, Jimma University, Jimma, Ethiopia; cw Department of Medicine, Rawalpindi Medical University, Rawalpindi, Pakistan; cx Brody School of Medicine, East Carolina University, Greenville, NC, USA; cy National Nutrition and Food Technology Research Institute, Shahid Beheshti University of Medical Sciences, Tehran, Iran; cz University Institute of Diet and Nutritional Sciences, The University of Lahore, Lahore, Pakistan; da Institute of Global Health and Development, Aga Khan University, karachi, Pakistan; db Faculty of Health and Behavioural Sciences, The University of Queensland, Brisbane, QLD, Australia; dc College of Engineering and Information Technology, Ajman University, Ajman, United Arab Emirates; dd School of Business, University of Jordan, Amman, Jordan; de Faculty of Pharmacy, Philadelphia University, Amman, Jordan; df School of Pharmacy, Cardiff University, Cardiff, Wales; dg Department of Adult Health and Critical Care, Sultan Qaboos University, Muscat, Oman; dh School of Public Health, University of Texas, Houston, TX, USA; di College of Engineering and Technology, American University of Ras Al Khaimah, Egaila, Kuwait; dj Department of Oral and Maxillofacial Surgery, University of Ibadan, Ibadan, Nigeria; dk Department of Oral and Maxillofacial Surgery, University College Hospital, Ibadan, Ibadan, Nigeria; dl Department of Biomedical Informatics, Biostatistics, and Epidemiology, University of Missouri, Columbia, MO, USA; dm McWilliams School of Biomedical Informatics, University of Texas, Houston, TX, USA; dn Department of Research and Development, Washington University in St. Louis, St. Louis, MO, USA; do Clinical Epidemiology Center, US Department of Veterans Affairs (VA), St. Louis, MO, USA; dp Murdoch Business School, Murdoch University, Perth, WA, Australia; dq Preventive Dentistry Department, Jouf University, Sakaka, Saudi Arabia; dr Department of Biology, College of Science, University of Hail, Hail, Saudi Arabia; ds Department of Biology, College of Science and Humanities in Al-Kharj, Prince Sattam bin Abdulaziz University, Al-Kharj, Saudi Arabia; dt School of Health and Environmental Studies, Hamdan Bin Mohammed Smart University, Dubai, United Arab Emirates; du School of Nursing, Yarmouk University, Irbid, Jordan; dv School of Nursing and Midwifery, Western Sydney University, Sydney, NSW, Australia; dw Imam Abdulrahman Bin Faisal University, Dammam, Saudi Arabia; dx Department of Health Information Management and Technology, Imam Abdulrahman Bin Faisal University, Dammam, Saudi Arabia; dy Department of Clinical Pharmacy, Al-Ayen Iraqi University, Thi-Qar, Iraq; dz Department of Clinical Pharmacy and Pharmacy Practice, University of Science and Technology, Sana’a, Yemen; ea College of nursing and health sciences, Jazan University, Jazan, Saudi Arabia; eb Department of Community and Mental Health, Al al-Bayt University, Mafraq, Jordan; ec Center for Health Systems Research, National Institute of Public Health, Cuernavaca, Mexico; ed Division of Pediatric Cardiology, University of Colorado, Aurora, CO, USA; ee Heart Center, King Faisal Specialist Hospital & Research Center, Riyadh, Saudi Arabia; ef General Directorate of Research and Studies, Ministry of Health, Riyadh, Saudi Arabia; eg Institute of Health Informatics, University College London, London, England; eh Department of Health Metrics Sciences, School of Medicine, University of Washington, Seattle, WA, USA; ei College of Pharmacy, University of Sharjah, Sharjah, United Arab Emirates; ej School of Pharmacy, The University of Jordan, Amman, Jordan; ek Pediatric Intensive Care Unit, King Saud University, Riyadh, Saudi Arabia; el Department of Epidemiology and Biostatistics, University of South Carolina, Columbia, SC, USA; em Department of Bacteriology, Immunology, and Mycology, Suez Canal University, Ismailia, Egypt; en Institute of Health Sciences, University of Health and Allied Sciences, Ho, Ghana; eo Faculty of Dentistry, Ibn Al-Nafis University for Medical Sciences, Sana’a, Yemen; ep Department of Oral and Maxillofacial Surgery, Xi’an Jiaotong University, Xi’an, China; eq Department of Statistics and Operations Research, Aligarh Muslim University, Aligarh, India; er College of Dental Medicine, Qatar University, Doha, Qatar; es Department of Microbiology and Immunology, National University of Medical Sciences (NUMS), Rawalpindi, Pakistan; et Department of Pharmacy, Mohammed Al-Mana College for Medical Sciences, Dammam, Saudi Arabia; eu Department of Medical Rehabilitation (Physiotherapy), University of Maiduguri, Maiduguri, Nigeria; ev Nethersole School of Nursing, The Chinese University of Hong Kong, Hong Kong, China; ew Department of Biosciences, Jamia Millia Islamia, New Delhi, India; ex Biodiversity and Genomic unit, University of Tabuk, University of Tabuk, Tabuk, Saudi Arabia; ey University of Swat, Swat, Pakistan; ez Department of Nuclear Medicine, King Hussein Cancer Center, Amman, Jordan; fa Department of Diagnostic Radiology and Nuclear Medicine, The University of Jordan, Amman, Jordan; fb The School of Medicine, The University of Jordan, Amman, Jordan; fc Department of Anesthesia, College of Medicine and Health Sciences, Bahir Dar University, Bahir Dar, Ethiopia; fd Visiting Scholar (Collaborative Projects), Center of Excellence in Precision Medicine and Digital Health, Chulalongkorn University, Bangkok, Thailand; fe School of Biomedical Engineering, University of New South Wales, Sydney, NSW, Australia; ff Department of Health Care Management and Economics, Urmia University of Medical Sciences, Urmia, Iran; fg Biomedical Physics Group, University of Hamburg, Hamburg, Germany; fh Department of Clinical and Community Pharmacy, An-Najah National University, Nablus, Palestine; fi Family and Community Medicine Department, Qassim University, Al Qassim, Saudi Arabia; fj Department of Public Health and Community Medicine, International Medical University, Kuala Lumpur, Malaysia; fk International Centre for Casemix and Clinical Coding, National University of Malaysia, Bandar Tun Razak, Malaysia; fl Faculty of Nursing, University of Tabuk, Tabuk, Saudi Arabia; fm College of Nursing and Health Sciences, Jazan University, Jazan, Saudi Arabia; fn BRAC Institute of Governance and Development (BIGD), BRAC University, Dhaka, Bangladesh; fo Department of Public and Community Health, Frontier University Garowe (FUG), Puntland, Somalia; fp Independent Consultant, Amman, Jordan; fq Department of Medicine, Nazarbayev University, Astana, Kazakhstan; fr Department of Clinical Nursing, Al-Zaytoonah University of Jordan, Amman, Jordan; fs Nursing Faculty, Fahad Bin Sultan University, Tabuk, Saudi Arabia; ft Department of Physical Therapy and Rehabilitation Sciences, Jordan University of Science and Technology, Irbid, Jordan; fu Department of Rehabilitation Sciences and Physical Therapy, Jordan University of Science and Technology, Irbid, Jordan; fv Liver, Digestive, and Lifestyle Health Research Section and Organ Transplant Center of Excellence, King Faisal Specialist Hospital & Research Center, Riyadh, Saudi Arabia; fw Division of Gastroenterology and Hepatology, Weill Cornell Medicine, New York, NY, USA; fx Department of Pathology and Microbiology, Jordan University of Science and Technology, Irbid, Jordan; fy Cell Therapy and Applied Genomics Department, King Hussein Cancer Center, Amman, Jordan; ga Department of Nursing, Georgetown University, Washington, DC, USA; gb Macro-Fiscal Policy Department, Ministry of Finance, Dubai, United Arab Emirates; gc Department of Pharmacology, Sana’a University, Sanaa, Yemen; gd Department of Basic Medical Sciences, Universiti Malaysia Sarawak, Kota Samarahan, Malaysia; ge Department of Biology, University of Hail, Hail, Saudi Arabia; gf College of Nursing, Jouf University, Sakaka, Saudi Arabia; gg Research, Policy, and Training Directorate, Jordan Center for Disease Control, Amman, Jordan; gh Applied Science Research Center, Applied Science Private University, Amman, Jordan; gi Department of Respiratory Therapy, King Abdulaziz University, Jeddah, Saudi Arabia; gj Department of Respiratory Medicine, University College London, London, England; gk Research Group in Health Economics, (University of Cartagena) (Universidad de Cartagena), Cartagena, Colombia; gl Research Group in Hospital Management and Health Policies, (University of the Coast) (Universidad de la Costa), Barranquilla, Colombia; gm Fakeeh College for Medical Sciences, Fakeeh College for Medical Sciences, Jeddah, Saudi Arabia; gn College of Medical Sciences, Azal University for Human Development, Sana’a, Yemen; go Faculty of Medicine, Jordan University of Science and Technology, Irbid, Jordan; gp Department of Pharmaceutical Sciences, Qatar University, Doha, Qatar; gq Food and Beverages Safety Research Center, Urmia University of Medical Sciences, Urmia, Iran; gr John D. Bower School of Population Health Science, University of Mississippi Medical Center, Jackson, MS, USA; gs Department of Preventive and Community Dentistry, Obafemi Awolowo University, Ile-Ife, Nigeria; gt Department of Pharmacy, An-Najah National University, Nablus, Palestine; gu College of Medicine, University of Sharjah, Sharjah, United Arab Emirates; gv Biological Sciences Collegiate Division, Cairo University, Cairo, Egypt; gw Department of Health and Management Sciences, Khomein University of Medical Sciences, Khomein, Iran; gx Department of Food Safety and Hygiene, Zanjan University of Medical Sciences, Zanjan, Iran; gy Spiritual Health Research Centre, Baqiyatallah University of Medical Sciences, Tehran, Iran; gz Department of Medicine, University of Jos, Jos, Nigeria; ha Department of Internal Medicine, Jos University Teaching Hospital, Jos, Nigeria; hb Department of Sociology, Usmanu Danfodiyo University, Sokoto, Sokoto, Nigeria; hc Department of Sociology, University of Johannesburg, Johannesburg, South Africa; hd Department of Health Care Management, Technical University of Berlin, Berlin, Germany; he European University, Lisbon, Portugal; hf Department of Internal Medicine, Rutgers University, Toms River, NJ, USA; hg Sarver Heart Center, University of Arizona, Tucson, AZ, USA; hh Institute of Global Health, University College London, London, England; hi Health System Science Program, The George Institute for Global Health, Newtown, NSW, Australia; hj Department of General Medicine, Thai Binh University of Medicine and Pharmacy in Vietnam, Thai Binh City, Viet Nam; hk Ophthalmology Department, Hoa Binh General Hospital, Hoa Binh City, Viet Nam; hl Department of Management, University of Cape Coast, Cape Coast, Ghana; hm Department of Public Health, The Apollo University, Chittoor, India; hn Department of Environmental and Occupational Health, University of Medical Sciences, Ondo, Ondo, Nigeria; ho Department of Microbiology, University of Medical Sciences, Ondo, Ondo, Nigeria; hp Regenerative Medicine, Organ Procurement and Transplantation Multi-disciplinary Center, Guilan University of Medical Sciences, Rasht, Iran; hq Department of Surgery, Gadjah Mada University, Yogyakarta, Indonesia; hr College of Medicine, Imam Mohammad Ibn Saud Islamic University, Riyadh, Saudi Arabia; hs Rural Health Research Institute, Charles Sturt University, Orange, NSW, Australia; ht Health Management and Economics Research Center, Iran University of Medical Sciences, Tehran, Iran; hu Al Ain University, Al Ain University, Abu Dhabi, United Arab Emirates; hv Department of Applied Mathematics, University of Washington, Seattle, WA, USA; hw College of Art and Science, Ottawa University, Surprise, AZ, USA; hx Department of Veterinary Pharmacology and Toxicology, University of Ilorin, Ilorin, Nigeria; hy School of Medicine, Shahid Beheshti University of Medical Sciences, Tehran, Iran; hz Department of Biotechnology, Sri Ramaswamy Memorial Institute of Science and Technology, Kattankulathur, India; ia University College of Medicine & Dentistry, The University of Lahore, Lahore, Pakistan; ib Institute of Social, Medical, and Applied Research Technology, Institute of Social, Medical, and Applied Research Technology, Moscow, Russia; ic Nazarbayev University, Nazarbayev University, Astana, Kazakhstan; id Department of Pharmacy Practice, AlMaarefa University, Riyadh, Saudi Arabia; ie Research Institute of Dental Sciences, Shahid Beheshti University of Medical Sciences, Tehran, Iran; if National Agency for Strategic Research in Medical Education (NASRME), Ministry of Health and Medical Education, Tehran, Iran; ig Health and Social Care, University of Essex, Colchester, England; ih School of Traditional Chinese Medicine, Xiamen University Malaysia, Sepang, Malaysia; ii Researcher, Amhara Public Health Institute, Bahir Dar, Ethiopia; ij Department of Medical-Surgical Nursing, Manipal Academy of Higher Education, Udupi, India; ik Atchabarov Scientific Research Institute of Fundamental and Applied Medicine, Kazakh National Medical University, Almaty, Kazakhstan; il Department of Immunology, Zanjan University of Medical Sciences, Zanjan, Iran; im Harold Pupkewitz Graduate School of Business, Namibia University of Science and Technology, Windhoek, Namibia; in Department of Economics and Business Sciences, Walter Sisulu University, Mthatha, South Africa; io nursing, University of Energy and Natural Resources, Sunyani, Ghana; ip School of Medicine and Public Health, University of Newcastle, Newcastle, NSW, Australia; iq Discipline of Psychological Science, ACAP University College, Sydney, NSW, Australia; ir Department of Forensic Medicine, Lumbini Medical College, Palpa, Nepal; is Northumbria HealthCare NHS Foundation Trust, Newcastle upon Tyne, UK; it Department of Oral Biological Science, University of Dental Medicine, Mandalay, Myanmar; iu Dental Unit, U Hla Tun’s Hospice Cancer Foundation, Mandalay, Myanmar; iv Institute of Molecular Biology and Biotechnology (IMBB), The University of Lahore, lahore, Pakistan; iw Internal Medicine Department, St Paul’s Hospital Millennium Medical College, Addis Ababa, Ethiopia; ix Bloomberg School of Public Health, Johns Hopkins University, Baltimore, MD, USA; iy Postgraduate Program in Pediatrics and Child Health, Pontifical Catholic University of Rio Grande do Sul, Porto Alegre, Brazil; iz Department of Health Policy Planning and Management, University of Health and Allied Sciences, Ho, Ghana; ja Department of Health Economics, Centre for Health Policy Advocacy Innovation & Research in Africa (CHPAIR-Africa), Accra, Ghana; jb Advanced Medical & Dental Institute, Universiti Sains Malaysia, Penang, Malaysia; jc ASIDE Healthcare, Lewes, DE, USA; jd Faculty of Medicine, October 6 University, 6th of October City, Egypt; je Community Medicine Department, Ahmadu Bello University, Zaria, Nigeria; jf Department of Epidemiology, Shiraz University of Medical Sciences, Shiraz, Iran; jg Department of Epidemioloy, Shahid Beheshti University of Medical Sciences, Tehran, Iran; jh Physiology department, RAK Medical and Health Sciences University, Ras Alkhaimah, United Arab Emirates; ji Department of Physiotherapy, Manipal Academy of Higher Education, Manipal, India; jj Department of Biological Sciences, National University of Medical Sciences (NUMS), Rawalpindi, Pakistan; jk Department of Clinical Pathology, Mansoura Faculty of Medicine, Mansoura, Egypt; jl Microbiology Department, Horus University Egypt, Damietta, Egypt; jm Department of Neurological Surgery, Asan Medical Center, University of Ulsan, Seoul, South Korea; jn Department of Medicine, Wayne State University, Detroit, MI, USA; jo CRIMEDIM-Center for Research and Training in Disaster Medicine, Humanitarian Aid and Global Health, Università del Piemonte Orientale, Novara, Italy; jp Department of Community Medicine and Public Health, University of Aden, Aden, Yemen; jq College of Optometry, Pacific University, Forest Grove, OR, USA; jr Clinical Medical Research Center, Nanjing Children’s Hospital, Nanjing, China; js School of Public Affairs, Nanjing University of Science and Technology, Nanjing, China; jt Applied College, University of Tabuk, Tabuk, Saudi Arabia; ju Surgery, Global Surgery, Emergency Medicine Department, Maulana Azad Medical College, New Delhi, India; jv WHO Collaborating Centre for Research in Surgical Care Delivery in LMICs, Mumbai, India; jw Research and Scientific Division, Ministry of Health, Free Town, Sierra Leone; jx Department of Medical Informatics, Mashhad University of Medical Sciences, Mashhad, Iran; jy Department of Forensic Medicine and Toxicology, Manipal Academy of Higher Education, Manipal, India; jz TIRR Memorial Hermann, Houston, TX, USA; ka MAHER Centre for Global Health Research, Meenakshi Academy of Higher Education and Research (MAHER), Chennai, India; kb Royal College of Medicine Perak, Universiti Kuala Lumpur, Ipoh, Malaysia; kc Division of Biological Sciences, Tamil Nadu State Council for Science and Technology, Chennai, India; kd Center of Innovation, Technology, and Education (CITE), Anhembi Morumbi University, São José dos Campos, Brazil; ke Institute of Health and Wellbeing, Federation University Australia, Melbourne, VIC, Australia; kf Manna Institute, University of New England, Armidale, NSW, Australia; kg Clinic for Infectious and Tropical Diseases, Clinical Center of Serbia, Belgrade, Serbia; kh Faculty of Medicine, University of Belgrade, Belgrade, Serbia; ki Heidelberg Institute of Global Health (HIGH), Heidelberg University, Heidelberg, Germany; kj T.H. Chan School of Public Health, Harvard University, Boston, MA, USA; kk School of Health Professions, Bern University of Applied Sciences, 3008 Bern, Switzerland; kl Alpha Genomics Private Limited, Islamabad, Pakistan; km Non-communicable Diseases Research Center, Tehran University of Medical Sciences, Tehran, Iran; kn School of Medicine, Iran University of Medical Sciences, Tehran, Iran; ko Faculty of Nursing, King Khalid University, Mahyil Asir, Saudi Arabia; kp Department of Medical Education, University of Nevada Las Vegas, Las Vegas, NV, USA; kq Anesthesia and critical care, Bahir Dar University, Bahir Dar, Ethiopia; kr Department Nutrition and Dietetics, Bahir Dar University, Bahir Dar, Ethiopia; ks Department of Social and Administrative pharmacy, University of Gondar, Gondar, Ethiopia; kt Department of Human Anatomy and Histology, I.M. Sechenov First Moscow State Medical University, Moscow, Russia; ku Transplant and Hepatobiliary Surgery Service, Hospital Universitario Fundación Santa Fe de Bogotá (University Hospital Santa Fe Foundation of Bogotá), Bogota, Colombia; kv Subdirectorate of Clinical Studies and Clinical Epidemiology, Hospital Universitorio Fundación Santa Fe de Bogotá, Bogotá, Colombia; kw Department of Nursing, Debre Tabor University, Debre Tabor, Ethiopia; kx Department of Emergency and Critical Care Nursing, Bahir Dar University, Bahir Dar, Ethiopia; ky Department of Medicine, University of Alberta, Edmonton, AB, Canada; kz BRAC James P Grant School of Public Health, BRAC University, Dhaka, Bangladesh; la Education, University of Johannesburg, Johannesburg, South Africa; lb Institute of Behavioral Sciences, Debre Markos University, Debre Markos, Ethiopia; lc Hubert Department of Global Health, Emory University, Atlanta, GA, USA; ld Department of Global Health, George Washington University, Washington, DC, USA; le Department of Anatomy, All India Institute of Medical Sciences, Jodhpur, India; lf National Institute for Implementation Research on Non Communicable Diseases, Indian Council of Medical Research, Jodhpur, India; lg Global Health Neurology Lab, NSW Brain Clot Bank, Sydney, NSW, Australia; lh Division of Cerebrovascular Medicine and Neurology, National Cerebral and Cardiovascular Center, Suita, Japan; li Translational and Clinical Research Institute, Newcastle University, Newcastle upon Tyne, England; lj University Centre for Research and Development, Chandigarh University, Mohali, India; lk Department of Human Genetics and Molecular Medicine, Central University of Punjab, Bathinda, India; ll Department of Pharmaceutical Sciences, Guru Nanak Dev University, Amritsar, India; lm Centre for Global Child Health, University of Toronto, Toronto, ON, Canada; ln Institute for Global Health & Development, Aga Khan University, Karachi, Pakistan; lo Independent Consultant, Addis Ababa, Ethiopia; lp Department of Community and Family Medicine, All India Institute of Medical Sciences, Deoghar, India; lq Department of Biochemistry and Biotechnology, University of Science and Technology Chittagong, Chittagong, Bangladesh; lr Department of Public Health, Daffodil International University, Dhaka, Bangladesh; ls Department of Public Health, Woldia University, Woldia, Ethiopia; lt SAMRC/Wits Centre for Health Economics and Decision Science - PRICELESS SA, Wits Health Consortium, Johannesburg, South Africa; lu Department of Community and Family Medicine, All India Institute of Medical Sciences, Ramanathapuram, India; lv Department of Veterinary Medicine, Islamic Azad University, Kermanshah, Iran; lw Faculty of Sport - Research Centre for Physical Activity, Health, and Leisure (CIAFEL), University of Porto, Porto, Portugal; lx College of Medecine and Health Sciences, University of Rwanda, Kigali, Rwanda; ly Department of Cardiology, (Centre Hospitalier Montfermeil) (Montfermeil Hospital Center), Montfermeil, France; ma Newcastle Business School, College of Human and Social Futures, University of Newcastle, Sydney, NSW, Australia; mb Department of Medicine, University Ferhat Abbas of Setif, Setif, Algeria; mc Department of Epidemiology and Preventive Medicine, University Hospital Saadna Abdenour, Setif, Algeria; md Department of Epidemiology, University of Florida, Gainesville, FL, USA; me Cancer Population Sciences Program, University of Florida Health Cancer Center, Gainesville, FL, USA; mf Department of Paediatrics and Child Health, Rivers State University, Nkpolu-Oroworukwo in Nigeria, Port Harcourt, Nigeria; mg Department of Paediatrics and Child Health, Rivers State University Teaching Hospital, Port Harcourt, Nigeria; mh Department of Management, Bucharest University of Economic Studies, Bucharest, Romania; mi School of Medicine and Health, Technical University of Munich, Munich, Germany; mj Department of Radiology, University of Cambridge, Cambridge, England; mk Department of Health Care Management, Technische Universität Berlin (Technical University of Berlin), Berlin, Germany; ml Department of Basic Biomedical Sciences, University of Sharjah, Sharjah, United Arab Emirates; mm School of Public Health Sciences, University of Waterloo, Waterloo, ON, Canada; mn Al Shifa School of Public Health, Al Shifa Trust Eye Hospital, Rawalpindi, Pakistan; mo Center for Nutrition and Health Research, National Institute of Public Health, Cuernavaca, Mexico; mp Department of Anesthesiology, Third Xiangya Hospital of Central South University, Changsha, China; mq Faculty of Dentistry, National University of Singapore, Singapore, Singapore; mr Centre For Data Driven Policy and Management, Catholic University of Croatia, Zagreb, Croatia; ms Department of Primary Care and Public Health, Imperial College London, London, England; mt Department of Health Care, Metropolitan Autonomous University, Mexico City, Mexico; mu IMPInstitute for Mental and Physical Health and Clinical Translation (IMPACT), Deakin University, Geelong, VIC, Australia; mv Research Unit on Applied Molecular Biosciences (UCIBIO), University of Porto, Porto, Portugal; mw Department of Pharmacological and Biomolecular Sciences, University of Milan, Milan, Italy; mx MultiMedica Sesto San Giovanni IRCCS, Sesto San Giovanni, Italy; my Posgrado de Medicina (Postgraduate Program in Medicine), Universidad Cientifica del Sur, Lima, Peru; mz Clinical Nutrition Department, Jazan University, Jazan, Saudi Arabia; na Department of Psychiatry, University of Kelaniya, Ragama, Sri Lanka; nb University Psychiatry Unit, Colombo North Teaching Hospital, Ragama, Sri Lanka; nc Department of Public Health Dentistry, Government Dental College & Hospital, Vijayawada, Vijayawada, India; nd College of Medicine, National Taiwan University, Taipei, Taiwan; ne Department of Nursing, National Taiwan University Hospital, Taipei, Taiwan; nf Cardiology Department, the First Affiliated Hospital of Jinzhou Medical University, Jinzhou, China; ng Department of Public Health and Health Sciences, Tennessee State University (TSU), Nashville, TN, USA; nh Department of Community Medicine, Datta Meghe Institute of Medical Sciences, Sawangi, India; ni Department of Epidemiology, The University of Texas Health Science Center at Houston (UTHealth Houston), Houston, TX, USA; nj Department of Biology, Imam Mohammad Ibn Saud Islamic University, Riyadh, Saudi Arabia; nk ICMR National Institute of Health Research, Dibrugarh, Indian Council of Medical Research, Dibrugarh, India; nl Department of Oral Medicine and Radiology, King George’s Medical University, Lucknow, India; nm Department of Anatomy and Physiology, Faculty of Medicine, Universiti Sultan Zainal Abidin, Kuala Terengganu, Malaysia; nn Clinical Research Center, Zhujiang Hospital of Southern Medical University, Guangzhou, China; no Department of Endocrine and Metabolic Diseases, Shanghai Jiao Tong University, Shanghai, China; np Red Cross College of Nursing, Chung-Ang University, Seoul, South Korea, Seoul, South Korea; nq Department of Medicine, National University of Singapore, Singapore, Singapore; nr Centre for Research Impact & Outcome, Chitkara University, Rajpura, India; ns Department of Biosciences, Saveetha University, Chennai, India; nt Department of Community Medicine, Jawaharlal Nehru Medical College, Wardha, India; nu The Interdisciplinary Research Group on Biomedicine and Health, VNU International School (VNUIS), Hanoi, Viet Nam; nv Faculty of Applied Sciences, VNU International School (VNUIS), Hanoi, Viet Nam; nw Department of Public Health, Other, Tuguegarao City, Philippines; nx Department of Paediatric Surgery, Federal Medical Centre, Umuahia, Nigeria; ny Department of Health Behavior, Texas A&M University, College Station, TX, USA; nz Hypertension and Cardiovascular Risk Research Group, University of Bologna, Bologna, Italy; oa Heart-Chest-Vascular Department, IRCCS Azianda Ospedaliero-Universitaria di Boloogna, Bologna, Italy; ob The David S. and Ruth L. Gottesman Center for Headache Treatment and Translational Research, Icahn School of Medicine at Mount Sinai, New York, NY, USA; oc Department of Medicine, Icahn School of Medicine at Mount Sinai, New York, NY, USA; od Nova Medical School, Nova University of Lisbon, Lisbon, Portugal; oe Department of Health Sciences, University of Florence, Florence, Italy; of Life and Health Sciences Research Institute (ICVS), University of Minho, Braga, Portugal; og Institute for Research and Innovation in Health (i3S), University of Porto, Porto, Portugal; oh Department of Anesthesia, Critical Care and Pain Medicine, Shahid Beheshti University of Medical Sciences, Tehran, Iran; oi Center for Immigrant and Refugee Health, Public Health Institute, Los Angeles, CA, USA; oj Faculty of Medicine, Public Health, and Nursing (FK-KMK), Gadjah Mada University, Yogyakarta, Indonesia; ok Institute of Health and Allied Sciences, University of Health and Allied Sciences, Ho, Ghana; ol Department of Public Health and Primary Care, University of Cambridge, Cambridge, England; om Cardiovascular Metabolic Translational Research Program, National University of Singapore, Singapore, Singapore; on Department of Medical and Surgical Sciences, University of Foggia, Foggia, Italy; oo Institute for Global Health Innovations, Duy Tan University, Hanoi, Viet Nam; op Department of Public Health, Haramaya University, Harar, Ethiopia; oq Department of Environmental Health, Arak University of Medical Sciences, Arak, Iran; or Department of Community and Preventive Dentistry, Federal University of Minas Gerais, Belo Horizonte, Brazil; os Department of Health Policy & Health Services Research, Boston University, Boston, MA, USA; ot School of Public Health, San Diego State University, San Diego, CA, USA; ou School of Medicine, University of Colima, Colima, Mexico; ov Department of Research, State Cancerology Institute of Colima, IMSS-BIENESTAR, Colima, Mexico; ow Department of Health Systems and Policy, University of Gondar, Gondar, Ethiopia; ox Department of Physiology, Bahir Dar University, Bahir Dar, Ethiopia; oy Department of Biological Sciences, University of Manouba, Manouba, Tunisia; oz Department of Social Sciences, University of Jendouba, El Kef, Tunisia; pa St Paul’s Eye Unit, Royal Liverpool University Hospital, Liverpool, UK; pb Clinic for Pulmonary Diseases and Tuberculosis “Podhrastovi”, University Clinical Center in Sarajevo, Sarajevo, Bosnia and Herzegovina; pc Department of Public Health, Debre Tabor University, Debre Tabor, Ethiopia; pd Chettinad Hospital & Research Institute, Chettinad Academy of Research and Education, Chennai, India; pe Department of Pharmacy, United International University, Dhaka, Bangladesh; pf Pharmacology Division, Center for Life Sciences Research Bangladesh, Dhaka, Bangladesh; pg Yeungnam University, Yeungnam University, Daegu, South Korea; ph Neurology Department Institute of Human Behavior and Allied Sciences, University of Delhi, New Delhi, India; pi Research and Development Cell, Dr. D. Y. Patil Vidyapeeth, Pune (Deemed to be University), Pune, India; pj Department of Pharmaceutical Regulatory Affairs and Management, Manipal Academy of Higher Education, Manipal, India; pk Department of Life Science and Public Health, Università Cattolica del Sacro Cuore (Catholic University of the Sacred Heart), Rome, Italy; pl Faculty of Management, Bucharest University of Economic Studies, Bucharest, Romania; pm Department of Health Promotion and Education, University of Ibadan, Ibadan, Nigeria; pn College of Health Sciences, VinUniversity, Hanoi, Viet Nam; po Institute of Health Economics and Technology (iHEAT), Hanoi, Viet Nam; pp International Institute for Training and Research (INSTAR), Vietnam National University, Hanoi, Viet Nam; pq Department of Medical, Surgical, and Health Sciences, University of Trieste, Trieste, Italy; pr Cardio-Thoraco-Vascular Department, Azienda Sanitaria Universitaria Giuliano Isontina, Trieste, Italy; ps Department of Medical Laboratory Sciences, Iran University of Medical Sciences, Tehran, Iran; pt Independent Consultant, Bridgewater, NJ, USA; pu Department of Epidemiology and Biostatistics, University of Health and Allied Sciences, Ho, Ghana; pv School of Sociology, University College Dublin, Dublin, Ireland; pw Faculty of Science and Humanities, SRM Institute of Science and Technology, Kattankulathur, India; px Department of Infection and Tropical Medicine, University of Sheffield, Sheffield, England; py Health Research Institute, Al Farabi Kazakh National University, Almaty, Kazakhstan; pz Department of Psychiatry, Dalhousie University, Halifax, NS, Canada; qa Department of Psychiatry, University of Alberta, Edmonton, AB, Canada; qb Department of Rehabilitation Science & Technology, School of Health and Rehabilitation Sciences, University of Pittsburgh, Pittsburgh, PA, USA; qc College of Medicine and Health Science, Debre Markos University, Debre Markos, Ethiopia; qd School of Nursing and Midwifery, La Trobe University, Melbourne, VIC, Australia; qe Advanced Nursing Department, Universitas Airlangga (Airlangga University), Surabaya, Indonesia; qf La Trobe University, Melbourne, VIC, Australia; qg Antimicrobial Resistance - Department of Planning, Research and Statistics, NCDC, Independent Consultant, Abuja, Nigeria; qh Faculty of Science and Health, University of Portsmouth, Hampshire, UK; qi Mohammed Al-Mana College for Medical Sciences, Dammam, Saudi Arabia; qj Department of Basic Medical Sciences, University of Sharjah, Sharjah, United Arab Emirates; qk Department of Anatomy and Embryology, Mansoura University, Mansoura, Egypt; ql College of Medicine, Korea University, Seoul, South Korea; qm Institute of Public Health, United Arab Emirates University, Al Ain, United Arab Emirates; qn Department of Healthcare Policy and Financing, Global Health Focus, Khartoum, Sudan; qo Department of Biology, College of Sciences, University of Hail, Hail, Saudi Arabia; qp Deanery of Biomedical Sciences, University of Edinburgh, Edinburgh, Scotland; qq Department of Pediatrics, University of Texas, Dallas, TX, USA; qr Department of Community Health, Obafemi Awolowo University, Ile-Ife, Nigeria; qs Interdisciplinary Health Sciences, University of Texas at El Paso, Texas, TX, USA; qt Department of Bacteriology and Virology, Semnan University of Medical Sciences, Semnan, Iran; qu Cancer Research Center, Semnan University of Medical Scinces, Semnan, Iran; qv Department of Public Health Epidemiology, Florida International University, Miami, FL, USA; qw Drexel Dornsife School of Public Health, Drexel University, Philadelphia, PA, USA; qx Department of Anesthesia, Cincinnati Children’s Hospital Medical Center, Cincinnati, OH, USA; qy Department of Biotechnology, University of the Western Cape, Cape Town, South Africa; qz Department of Electrical and Computer Engineering, Tarbiat Modares University, Tehran, Iran; ra Department of Epidemiology and Medical Statistics, University of Ibadan, Ibadan, Nigeria; rb Research Centre for Healthcare and Community, Coventry University, Coventry, UK; rc Tanner College of Dental Medicine, University of Pikeville, Pikeville, KY, USA; rd Scientific and Technological Park, Kazakh National Medical University, Almaty, Kazakhstan; re Department of Medicine, Korea University, Seoul, South Korea; rf College of Medicine, AlMaarefa University, Riyadh, Saudi Arabia; rg Department of Clinical Laboratory Sciences, Jouf University, Sakaka, Saudi Arabia; rh School of Human and Social Sciences (FCHS), University of Algarve, Faro, Portugal; ri University Research Center in Psychology, Faro, Portugal; rj Department of Psychology, Federal University of Sergipe, São Cristóvão, Brazil; rk Dentistry Research Institute, Tehran University of Medical Sciences, Tehran, Iran; rl Department of Agriculture and Animal Health, University of South Africa, Roodepoort, South Africa; rm Department of Electrical and Computer Engineering (ECE), Tarbiat Modares University, Tehran, Iran; rn Department of Infectious Diseases and Public Health, City University of Hong Kong, Hong Kong, China; ro Department of Pharmacy, Wollega University, Nekemte, Ethiopia; rp Department of Neurosurgery, Children’s National Medical Center, Washington, DC, USA; rq Department of Neurosurgery, George Washington University, Washington, DC, USA; rr Center for Public Health Research, University of Milan Bicocca, Monza, Italy; rs Laboratory of Public Health, IRCCS Istituto Auxologico Italiano, Milan, Italy; rt European Network of Occupational Therapy in Higher Education, Vienna, Austria; ru Faculty of Rehabilitation and Allied Health Sciences, Riphah International University, Lahore, Pakistan; rv Institute of Public Health, Charité Universitätsmedizin Berlin (Charité Medical University Berlin), Berlin, Germany; rw Hypertension and Cardiovascular Risk Research Center, University of Bologna, Bologna, Italy; rx Department of Medical Pharmacology, Ataturk University, Erzurum, Türkiye; ry Department of Cell Biology and Biotechnology, K.A. Timiryazev Institute of Plant Physiology, Moscow, Russia; rz UO Neurologia, Salute Pubblica e Disabilità (The Neurology, Public Health and Disability Unit), Fondazione IRCCS Istituto Neurologico Carlo Besta (IRCCS Foundation Carlo Besta Neurological Institute), Milan, Italy; sa Department of Community Medicine, “Iuliu Hatieganu” University of Medicine and Pharmacy, Cluj-Napoca, Romania; sb MEDCIDS, Faculty of Medicine of the University of Porto, University of Porto, Porto, Portugal; sc Center for Health Technology and Services Research (CINTESIS), Porto, Portugal; sd Department of Dermatology, Kyoto Prefectural University of Medicine, Kyoto, Japan; se Gates Foundation, Seattle, WA, USA; sf Department of Healthcare Management, University of Doha for Science and Technology, Doha, Qatar; sg Department of Preventive and Social Medicine, University of Otago, Dunedin, New Zealand; sh Health Services Management Training Centre, Semmelweis University, Budapest, Hungary; si Department of Applied Social Sciences, Sapientia Hungarian University of Transylvania, Târgu-Mureş, Romania; sj Department of Community Medicine, Bayero University Kano, Kano, Nigeria; sk Department of Community Medicine, Aminu Kano Teaching Hospital, Kano, Nigeria; sl School of Public Health, University of Ghana, Legon-Accra, Ghana; sm Department of Global Health and Population, Harvard University, Boston, MA, USA; sn Department of Public Health, University of Szeged, Szeged, Hungary; so Laboratory Sciences Division, National Institute of Epidemiology, Chennai, India; sp Trauma and Emergency Medicine, All India Institute of Medical Sciences, Madurai, India; sq Department of Health Policy and Administration, University of the Philippines Manila, Manila, Philippines; sr Internal Medicine Department, University of Pittsburgh Medical Center, Harrisburg, PA, USA; ss College of Medicine, Drexel University, Philadelphia, PA, USA; st Infectious Diseases Unit, University of Verona, Verona, Italy; su Amity Institute of Pharmacy, Amity University Uttar Pradesh, Noida, India; sv Cochlear Center for Hearing and Public Health, Johns Hopkins University, Philadelphia, PA, USA; sw Department of Public Health, Johns Hopkins University, Baltimore, MD, USA; sx Director General Office, Ethiopian Public Health Institute, Addis Ababa, Ethiopia; sy Department of Neurosciences, Neurology and Stroke Unit, ASST Grande Ospedale Metropolitano Niguarda, Milan, Italy; sz Department of Neurosurgery, Oregon Health and Science University, Portland, OR, USA; ta Harold Pupkewitz Graduate School of Business, Polytechnic of Namibia, Windhoek, Namibia; tb Department of Industrial Relations and Human Resource Management, Lagos State University, Lagos, Nigeria; tc College of Medicine and Health Science, University of Gondar, Gondar, Ethiopia; td School of Medical and Health Science, Edith Cowan University, Australia, Perth, WA, Australia; te College of Medicine and Health Science, Bahir Dar University, Bahir Dar, Ethiopia; tf Department of Health Information Technology and Management, Shahid Beheshti University of Medical Sciences, Tehran, Iran; tg School of Nursing and Midwifery, Shahid Beheshti University of Medical Sciences, Tehran, Iran; th Department of Laboratory Sciences, Khomein University of Medical Sciences, Khomein, Iran; ti Tehran University of Medical Sciences, Tehran, Iran; tj Department of Applied Health Sciences, University of Birmingham, Birmingham, England; tk Research Committee of Qom University of Medical Sciences, Qom University of Medical Sciences, Qom, Iran; tl Department of Medical Biochemical Analysis, Cihan University-Erbil, Erbil, Iraq; tm School of Public Health, Qazvin University of Medical Sciences, Qazvin, Iran; tn Research Department, Nepal Health Research Council, Kathmandu, Nepal; to Department of Radiology, University of Southern California, Los Angeles, CA, USA; tp School of Social Sciences, Hellenic Open University, Patras, Greece; tq Department of Social and Behavioral Sciences, European University Cyprus, Nicosia, Cyprus; tr Country Office, World Health Organization (WHO), Astana, Kazakhstan; ts Department of Biological Sciences and Chemistry (DBSC), University of Nizwa, Nizwa, Oman; tt Department of Zoology, KKS Women’s College, Balasore, India; tu Department of Nursing, Aksum University, Aksum, Ethiopia; tv Department of Health Systems and Policy Research, Indian Institute of Public Health, Gandhinagar, India; tw Department of Genetics, Sana Institute of Higher Education, Sari, Iran; tx Universal Scientific Education and Research Network (USERN), Kermanshah University of Medical Sciences, Kermanshah, Iran; ty Department of Life Sciences, Health and Healthcare Professions, Link Campus University, Rome, Italy; tz Research Institute for Endocrine Sciences, Tehran, Iran; ua Midwifery Department, Debre Tabor University, Debre Tabor, Ethiopia; ub Postgraduate Program in Epidemiology, Federal University of Rio Grande do Sul, Porto Alegre, Brazil; uc Department of Pathology, Apollo Institute of Medical Sciences and Research, Chittoor, India; ud Department of Dermatology, Case Western Reserve University, Cleveland, OH, USA; ue Medical Directorate, Local Health Authority of Ferrara, Ferrara, Italy; uf Department of the Health Directorate, Local Health Authority of Bologna, Bologna, Italy; ug Department of Biomedical and Neuromotor Sciences, University of Bologna, Bologna, Italy; uh Department of Pediatrics, All India Institute of Medical Sciences, New Delhi, India; ui School of Global Health and Bioethics, Euclid University, Banjul, The Gambia; uj Department of Community Medicine, University of Peradeniya, Kandy, Sri Lanka; uk Global Health Research Center, Duke Kunshan University, Kunshan, China; ul Department of Dermatology, Dr. D.Y. Patil Medical College, Hospital and Research Centre, Pune, India; um Department of Public Health, Torrens University Australia, Melbourne, VIC, Australia; un Department of Preventive Cardiology & Medicine, Eternal Heart Care Centre & Research Institute, Jaipur, India; uo Department of Medicine, Mahatma Gandhi University Medical Sciences, Jaipur, India; up Department of Toxicology, Shriram Institute for Industrial Research, Delhi, India; uq School of Biotechnology, Dublin City University, Dublin, Ireland; ur Doctoral Program in Biomedical Gerontology, Pontifical Catholic University of Rio Grande do Sul, Porto Alegre, Brazil; us Research Unit in Epidemiology Clinic, Mexican Institute of Social Segurity, Colima, Mexico; ut Global Virus Network, Middle East Region, Shiraz, Iran; uu Biological Sciences, King Abdulaziz University, Jeddah, Saudi Arabia; uv Euclid University, Greater Banjul Area, The Gambia; uw Department of Community Medicine, Post Graduate Institute of Medical Education and Research, Chandigarh, India; ux Centre for Community Medicine, All India Institute of Medical Sciences, New Delhi, India; uy Department of Chemistry, College of Science, University of Sulaimani, Sulaymaniyah, Iraq; uz The University of Jordan, The University of Jordan, Amman, Jordan; va Oral & Maxillofacial Rehabilitation Department, King Abdulaziz University, Jeddah, Saudi Arabia; vb Department of Fixed Prosthodontics, Cairo University, Cairo, Egypt; vc Department of Cardiology, Azerbaijan Medical University, Baku, Azerbaijan; vd Sakarya University, Sakarya, Türkiye; ve Department of Computer and Information Science, Majmaah University, Al Majmaah, Saudi Arabia; vf Research Unit, Parc Sanitari Sant Joan de Deu, Barcelona, Spain; vg Department of Mental Health, Biomedical Research Networking Center for Mental Health Network (CiberSAM), Madrid, Spain; vh Department of Public Health, Tropical Disease and Health Research Center, Dhaka, Bangladesh; vi Department of Biomedical Engineering and Public Health, World University of Bangladesh, Dhaka, Bangladesh; vj Department of Food Technology and Nutrition Science, Noakhali Science and Technology University, Noakhali, Bangladesh; vk Department of Medical Surgical, Shahroud University of Medical Sciences, Shahrekord, Iran; vl Institute of Radiology and Radiological Sciences, Tehran University of Medical Sciences, tehran, Iran; vm Department of Surgery, University of California San Francisco, Oakland, MD, USA; vn Dept. of Periodontia & Community dentistry, Dr Ziauddin Ahmad Dental College, Aligarh Muslim University, Aligarh, India; vo Department of Pharmaceutical Technology, Adamas University, Kolkata, India; vp Department of Neurology, Cairo University, Cairo, Egypt; vq Department of Community Medicine, Federal University Teaching Hospital, Lafia, Nigeria; vr Department of Epidemiology and Community Medicine, Federal University of Lafia, Lafia, Nigeria; vs Centre for Interdisciplinary Research in Basic Sciences, Jamia Millia Islamia, New Delhi, India; vt Aberdeen Royal Infirmary, NHS National Services Scotland, Aberdeen, Scotland; vu Department of Internal Medicine, University of Edinburgh, Scotland, Scotland; vv Community-Oriented Nursing Midwifery Research Center, Shahrekord University of Medical Sciences, Shahrekord, Iran; vw National Agency for Strategic Research in Medical Sciences Education, Ministry of Health and Medical Education, Tehran, Iran; vx School of Nursing, Johns Hopkins University, Baltimore, MD, USA; vy Graduate School of Medicine, University of Tokyo, Tokyo, Japan; vz Kasturba Medical College Mangalore, Manipal Academy of Higher Education, Manipal, India; wa School of Health in Social Science, University of Edinburgh, Edinburgh, Scotland; wb School of Computer Science, Duy Tan University, Da Nang, Viet Nam; wc International Center for Materials Sciences and Technology, Western Caspian University, Baku, Azerbaijan; wd Maternal and Child Health Division (MCHD), International Centre for Diarrhoeal Disease Research, Bangladesh, Dhaka, Bangladesh; we Department of Decision and Information Sciences, University of Houston, Houston, TX, USA; wf Public Health Research Group, Nature Study Society of Bangladesh, Khulna, Bangladesh; wg Department of Population Sciences, University of Dhaka, Dhaka, Bangladesh; wh Department of Internal Medicine, Carol Davila University of Medicine and Pharmacy, Bucharest, Romania; wi Department of Legal Medicine and Bioethics, Carol Davila University of Medicine and Pharmacy, Bucharest, Romania; wj Department of Clinical Legal Medicine, National Institute of Legal Medicine Mina Minovici, Bucharest, Romania; wk Department of Epidemiology and Health Statistics, Central South University, Changsha, China; wl Faculty of Medicine, The Chinese University of Hong Kong, Hong Kong, China; wm Advanced Institute of Convergence Knowledge Informatics, Tohoku University, Sendai, Japan; wn Graduate School of Engineering, Tohoku University, Sendai, Japan; wo Department of Public Health and Community Medicine, Shaikh Zayed Postgraduate Medical Institute, Lahore, Pakistan; wp Department of Mathematical Sciences, Federal University Gusau, Nigeria, Gusau, Nigeria; wq Physiotherapy Department, Tishk International University, Erbil, Iraq; wr Faculty of Pharmacy, Sultan Zainal Abidin University, Malaysia, Terengganu, Malaysia; ws Health Policy and Management Department, City University of New York, New York, NY, USA; wt Department of Cardiovascular Medicine, Cleveland Clinic, Cleveland, OH, USA; wu West Africa RCC, Africa Centre for Disease Control and Prevention, Abuja, Nigeria; wv Department of Community Medicine, University College Hospital, Ibadan, Ibadan, Nigeria; ww Faculty of Medical Sciences, University of Kragujevac, Kragujevac, Serbia; wx Department of Clinical Pharmacy, Prince Sattam bin Abdulaziz University, Al Kharj, Saudi Arabia; wy Faculty of Health and Life Sciences, University of Exeter, Exeter, England; wz Management Policy and Community Health, University of Texas, Houston, TX, USA; xa Department of Psychology, Wuhan University, Wuhan, China; xb Institute for Evidence-Based Healthcare, Bond University, Gold Coast, QLD, Australia; xc Faculty of Pharmacy, Universitas Ahmad Dahlan, Yogyakarta, Indonesia; xd Department of Environmental Health, Poltekkes Kemenkes Mamuju (Health Polytechnic of the Ministry of Health, Mamuju), Mamuju, Indonesia; xe School of Pharmacy, BRAC University, Dhaka, Bangladesh; xf Research and Publication Department, World Health Organization (WHO), Dhaka, Bangladesh; xg Department of Public Health, International University of Business Agriculture and Technology (IUBAT), Dhaka, Bangladesh; xh 4-Green Research Socoiety, Bangladesh, Dhaka, Bangladesh; xi Department of Clinical Pharmacy & Pharmacy Practice, Asian Institute of Medicine, Science and Technology, Bedong, Malaysia; xj Malaysian Academy of Pharmacy, Puchong, Malaysia; xk Department of General Surgery and Medical-Surgical Specialties, University of Catania, Catania, Italy; xl Department of Internal Medicine, Bridgeport Hospital, Bridgeport, CT, USA; xm Department of Digital Health, University of Tsukuba, Tsukuba, Japan; xn Department of Non-Communicable Disease Epidemiology, London School of Hygiene & Tropical Medicine, London, England; xo School of Health Systems and Public Health, University of Washington, Seattle, WA, USA; xp College of Nursing, All India Institute of Medical Sciences, Bhubaneswar, India; xq Department of Physical Medicine and Rehabilitation, Université Paris Cité, Paris, France; xr Research and Development Unit, Biomedical Research Networking Center for Mental Health Network (CiberSAM), Barcelona, Spain; xs Department of Special Education, University of Ibadan, Ibadan, Nigeria; xt Department of Nursing, Arak University of Medical Sciences, Arak, Iran; xu UCL Institute for Global Health, University of London, London, England; xv Department of Health Policy and Management, School of Public Health And Safety, Shahid Beheshti University of Medical Sciences, Tehran, Iran; xw College of Medicine and Health Sciences, Arabian Gulf University, Manama, Bahrain; xx Government Hospitals, Manama, Bahrain; xy Section of Economic & Social Sciences, Humanities & Arts, The World Academy of Sciences UNESCO-TWAS, Trieste, Italy; xz Shaanxi University of Technology, Hanzhong, China; ya Department of Environmental Engineering, Islamic Azad University, Ahvaz, Iran; yb Department of Public Health Sciences, University of Chicago, Chicago, IL, USA; yc Public Health Dentistry, Amrita Institute of Medical Sciences, Kochi, India; yd SRM Medical College Hospital and Research Centre, Sri Ramaswamy Memorial Institute of Science and Technology, Kattankulathur, India; ye Royal Prince Alfred Hospital, Sydney Local Health District, Sydney, NSW, Australia; yf Division of Medical Research, Sri Ramaswamy Memorial Institute of Science and Technology, Kattankulathur, India; yg Department of Community Health Nursing, Princess Aisha Bint Al-Hussein College of Nursing, Al-Hussein Bin Talal University in Jordan, Maan, Jordan; yh Department of Community and Family Medicine, All India Institute of Medical Sciences, Rajkot, India; yi Department of Pharmacology, Imam Mohammad Ibn Saud Islamic University, Riyadh, Saudi Arabia; yj The Medical City for Military and Security Services School, Muscat, Oman; yk Department of Biochemistry, Government Medical College, Mysuru, India; yl Centre for Evidence Synthesis and Implementation Research, Cephas Health Research Initiative Inc, Ibadan, Nigeria; ym Jindal School of Public Health and Human Development, O.P. Jindal Global University, Sonipat, India; yn Department of Dermatology, Yonsei University Wonju College of Medicine, Wonju, South Korea; yo Institute of Evidence Based Medicine, Yonsei University Wonju College of Medicine, Wonju, South Korea; yp Department of Biomedical Informatics, Korea University, Seoul, South Korea; yq Department of Information Science, Jimma University, Jimma, Ethiopia; yr Faculty of Veterinary Medicine, University of Calgary, Calgary, AB, Canada; ys Young Researchers and Elite Club, Islamic Azad University, Karaj, Iran; yt Department of Community Medicine, Jubilee Mission Medical College & Research Institute, Thrissur, Thrissur, India; yu School of Engineering and Technology, The University of New South Wales, Canberra, NSW, Australia; yv Department of Community Medicine, Manipal Academy of Higher Education, Mangalore, India; yw Department of Economics, National Open University, Benin City, Nigeria; yx Gastrointestinal and Liver Diseases Research Center, Guilan University of Medical Sciences, Rasht, Iran; yy Caspian Digestive Disease Research Center, Guilan University of Medical Sciences, Rasht, Iran; yz Department of Family Medicine and Public Health, University of Opole, Opole, Poland; za Department of Medical Informatics, Kangwon National University College of Medicine in South Korea, Chuncheon, South Korea; zb Department of Radiology and Nuclear Medicine, University of Jordan, Amman, Jordan; zc Department of Pathology, Saveetha Institute of Medical and Technical Sciences, Chennai, India; zd Department of Statistics, Salahaddin University-Erbil, Erbil, Iraq; ze Department of Business Administrations, Cihan University-Erbil, Erbil, Iraq; zf Faculty of Science, Al-Baha University, Al-Baha, Saudi Arabia; zg Department of Pharmacology, Post Graduate Institute of Medical Education and Research, Chandigarh, India; zh Department of Health, Khoy Medical Sciences, Khoy, Iran; zi Department of Dermatology, Amrita Institute of Medical Sciences, Kochi, India; zj Research Institute of Cardiology and Internal Medicine, Almaty, Research Institute of Cardiology and Internal Medicine, Almaty, Almaty, Kazakhstan; zk Department of Endocrinology, Bharti Hospital Karnal, Karnal, India; zl Prasanna School of Public Health, Manipal Academy of Higher Education, Manipal, India; zm Care and Public Health Research Institute (CAPHRI), Maastricht University, Maastricht, Netherlands; zn Elderwood Rehabilitation Clinical Centre, Buffalo, NY, USA; zo Department of Public Health, South Wales University, Treforest, UK; zp The KU-KIST Graduate School of Energy and Environment, Korea University, Seongbuk-Gu, South Korea; zq School of Health and Environmental Science, Korea University, Seoul, South Korea; zr Department of Anesthesia, Critical Care and Pain Medicine, Massachusetts General Hospital, Boston, MA, USA; zs Office of the Executive Director, Cephas Health Research Initiative Inc, Ibadan, Nigeria; zt Department of Public Health, Thomas Adewumi University, Oko, Nigeria; zu The Hansjörg Wyss Department of Plastic and Reconstructive Surgery, New York University, New York, NY, USA; zv Trauma Research Center, Shiraz University of Medical Sciences, Shiraz, Iran; zw 2nd Department of Cardiology, Aristotle University of Thessaloniki, Thessaloniki, Greece; zx Laboratory Science Department, Khomein University of Medical Sciences, Khomein, Iran; zy Department of Immunology, Tehran University of Medical Sciences, Tehran, Iran; zz European Observatory on Health Systems and Policies, London, UK; aaa Department of Health Services Research and Policy, London School of Hygiene & Tropical Medicine, London, England; aab Saveetha Medical College and Hospital, Saveetha University, Chennai, India; aac Department of Physical Therapy and Health Rehabilitation, Majmaah University, Al Majmaah, Saudi Arabia; aad Department of Surgery, MyungSung Medical College, Addis Ababa, Ethiopia; aae Department of Anesthesiology & Pain Medicine, University of Washington, Seattle, WA, USA; aaf Department of Clinical Research and Epidemiology, Institute of Liver and Biliary Sciences, New Delhi, India; aag Department of Neurosurgery, Johns Hopkins University, Baltimore, MD, USA; aah Jindal School of Public Health and Human Development, O. P. Jindal Global University, Sonipat, India, Sonipat, India; aai College of Sport Sciences, Qatar University, Doha, Qatar; aaj Department of Public Health, Jordan University of Science and Technology, Irbid, Jordan; aak Amity Institute of Forensic Sciences, Amity University, Noida, India; aal Health Research Center, Jazan University, Jazan, Saudi Arabia; aam National Center for Research, Khartoum, Sudan; aan Natural and Medical Sciences Research Center, University of Nizwa, Nizwa, Oman; aao Department of Pharmacy Administration and Clinical Pharmacy, Peking University, Beijing, China; aap University Institute of Public Health, The University of Lahore, Lahore, Pakistan; aaq Neurology, University of Tennessee, Memphis, TN, USA; aar Department of Biotechnology and Microbiology, M.J.P. Rohilkhand University, Bareilly, India; aas Department of Pharmacy, Minhaj University Lahore, Lahore, Pakistan; aat Institute of Molecular Biology and Biotechnology (IMBB), The University of Lahore, Lahore, Pakistan; aau Department of Community and Preventive Medicine, King Edward Medical University, Lahore, Pakistan; aav International Center for Chemical and Biological Sciences, International Center for Chemical and Biological Sciences, Karachi, Pakistan; aaw College of Medicine, University of Hail, Hail, Saudi Arabia; aax Department of Cardiology, University of South Wales, Treforest, UK; aay Department of Cardiology, University of Buckingham, Buckingham, UK; aaz Department of Pharmacology, All India Institute of Medical Sciences, Raipur, India; bba College of Health, Wellbeing and Life Sciences, Sheffield Hallam University, Sheffield, UK; bbb College of Arts and Sciences, Ohio University, Zanesville, OH, USA; bbc Faculty of Nursing, Yarmouk University, Irbid, Jordan; bbd Department of Basic Medical Sciences, Yarmouk University, Irbid, Jordan; bbe Department of Neurosurgery, Shahid Beheshti University of Medical Sciences, Tehran, Iran; bbf Asadabad School of Medical Sciences, Asadabad School of Medical Sciences, Asadabad, Iran; bbg Ophthalmic Epidemiology Research Center, Research Institute for Ophthalmology and Vision Science, Shahid Beheshti University of Medical Sciences, Tehran, Iran, Shahid Beheshti University of Medical Sciences, Tehran, Iran; bbh Institute of Research and Development, Duy Tan University, Da Nang, Viet Nam; bbi Research Center of High Technologies and Innovative Engineering, Western Caspian University, Baku, Azerbaijan; bbj Research Department, University of Inland Norway, Elverum, Norway; bbk Department of Health Policy and Management, Korea University, Seoul, South Korea; bbl School of Medicine, Creighton University, Omaha, NE, USA; bbm Cardiovascular Disease Initiative, Broad Institute of MIT and Harvard, Cambridge, MA, USA; bbn Massachusetts General Hospital, Boston, MA, USA; bbo Health and Healing Research, Education, and Service, Inc., Boston, MA, USA; bbp Millennium Prevention, Inc., Westwood, MA, USA; bbq School of Health Sciences, Kristiania University College, Oslo, Norway; bbr Department of International Health and Sustainable Development, Tulane University, New Orleans, LA, USA; bbs Department of Public Health Dentistry, Krishna Vishwa Vidyapeeth Deemed to be University, Karad, India; bbt Department of Global Health, University of Washington, Seattle, WA, USA; bbu Global Healthcare Consulting, New Delhi, India; bbv Department of Public Health and Community Medicine, Central University of Kerala, Kasaragod, India; bbw Center for Tobacco Control Research and Education, University of California San Francisco, San Francisco, CA, USA; bbx Department of Internal Medicine, Yale University, New Haven, CT, USA; bby Social Determinants of Health Research Center, Shahid Beheshti University of Medical Sciences, Tehran, Iran; bbz Children’s Medical Center, Tehran University of Medical Sciences, Tehran, Iran; cca Department of Science and Environmental Studies, The Education University of Hong Kong, Hong Kong, China; ccb Department of General Practice and Family Medicine, Kharkiv National Medical University, Kharkiv, Ukraine; ccc Department of Public Health, Masaryk University, Brno, Czech Republic; ccd Amity institute of Public Health and Hospital Administration, Amity University, Noida, India; cce Kasturba Medical College, Manipal, Manipal Academy of Higher Education, Udupi, India; ccf Feinberg School of Medicine, Northwestern University, Chicago, IL, USA; ccg School of Pharmacy, University of Ghana, Legon, Ghana; cch Department of Anthropology, Panjab University, Chandigarh, India; cci Department of Physiology, Sree Balaji Medical College and Hospital, Chennai, India; ccj Department of Biochemistry, University of Hail, Hail, Saudi Arabia; cck College of Medicine, National Cheng Kung University, Tainan, Taiwan; ccl Department of Research Management, Research Institute of Cardiology and Internal Diseases, Almaty, Kazakhstan; ccm Department of Health Administration and Education, University of Education, Winneba, Winneba, Ghana; ccn Amity Institute of Health Allied Sciences, Amity University Noida, Uttar Pradesh, Noida, India; cco Department of Community Medicine, Rajendra Institute of Medical Sciences, Ranchi, India; ccp Department of Mathematics, Amity University Haryana, Gurugram, India; ccq Shaheed Mohtarma Benazir Bhutto Medical College Lyari, Dow University of Health Sciences, Karachi, Pakistan; ccr Institute for Excellence in Health Equity, New York University, New York, NY, USA; ccs Department of Psychiatry, University of Nairobi, Nairobi, Kenya; cct Department of Community Medicine, Vardhman Mahavir Medical College and Safdarjung Hospital, Delhi, India; ccu Department of Pharmacology and Toxicology, National Institute of Pharmaceutical Education and Research, Hajipur, Hajipur, India; ccv Wits Advanced Drug Delivery Platform Research Unit, University of the Witwatersrand, Johannesburg, South Africa; ccw Wits Infectious Diseases and Oncology Research Institute, University of the Witwatersrand, Johannesburg, South Africa; ccx Department of Health Management, University of Hail, Hail, Saudi Arabia; ccy Faculty of Education, Shree Guru Gobind Singh Tricentenary University, Shree Guru Gobind Singh Tricentenary University, Gurugram, India; ccz Department of Medicine, Dow University of Health Sciences, Karachi, Pakistan; dda Department of Economics, Manipal University Jaipur, Jaipur, India; ddb School of Medicine and Dentistry, Griffith University, Gold Coast, QLD, Australia; ddc Nursing & Midwifery Research Department (NMRD), Hamad Medical Corporation, Doha, Qatar; ddd Department of Clinical Subjects, Al Farabi Kazakh National University, Almaty, Kazakhstan; dde Division of Research Methodology, Wroclaw Medical University, Wroclaw, Poland; ddf Institute for Health Sciences, STIKES Bethesda Yakkum Yogyakarta Indonesia, Yogyakarta, Indonesia; ddg Department of Public Health and Epidemiology, Khalifa University of Science and Technology, Abu Dhabi, United Arab Emirates; ddh Faculty of Public Health, University of Indonesia, Depok, Indonesia; ddi Department of Pediatric Oncology, Medicana Health International, Istanbul, Türkiye; ddj Department of Pediatric Oncology, Hacettepe University, Ankara, Türkiye; ddk Department of Public Health, Kazakh National Medical University, Almaty, Kazakhstan; ddl Centre for Vision across the Life Span, University of Huddersfield, Huddersfield, England; ddm Clinical Research Center, Turku University Hospital, Turku, Finland; ddn Heart Center, University of Turku and Turku University Hospital, Turku, Finland; ddo Department of Nursing Science, Bayero University Kano, Kano, Nigeria; ddp School of Nursing and Midwifery, La Trobe University, Bundoora, VIC, Australia; ddq Division of Evidence Synthesis, Foundation for People-centric Health Systems, New Delhi, India; ddr Division of Lifestyle Medicine, Centre for Health: The Specialty Practice, New Delhi, India; dds School of Digital Science, (University of Brunei Darussalam) (Universiti Brunei Darussalam), Bandar Seri Begawan, Brunei; ddt Institute of Applied Data Analytics, (University of Brunei Darussalam) (Universiti Brunei Darussalam), Bandar Seri Begawan, Brunei; ddu Department of Occupational and Environmental Health, School of Public Health, Huazhong University of Science and Technology, Wuhan, China; ddv Indian Council of Medical Research, New Delhi, India; ddw NEVES Society for Patient Safety, Budapest, Hungary; ddx Department of Behavioural Sciences and Learning, Linköping University, Linköping, Sweden; ddy Department of Medical Sciences, Uppsala University, Uppsala, Sweden; ddz Department of Clinical Chemistry and Pharmacology, Uppsala University Hospital, Uppsala, Sweden; eea Department of Otorhinolaryngology, Father Muller Medical College, Mangalore, India; eeb University of Management and Technology, Lahore, Pakistan; eec Department of Clinical Pharmacy and Pharmacy Management, Kaduna State University, Kaduna, Nigeria; eed College of Nursing, Sultan Qaboos University, Muscat, Oman; eee Department of Clinical and Experimental Medicine, University of Catania, Catania, Italy; eef Graduate School of Transdisciplinary Health Sciences, Yonsei University, Seoul, South Korea; eeg Kazakh National Medical University, Almaty, Kazakhstan; eeh Department of Precision Medicine, Sungkyunkwan University, Suwon-si, South Korea; eei Department of Family Medicine, University of Texas Medical Branch, Galveston, TX, USA; eej Department of Preventive Medicine, Korea University, Seoul, South Korea; eek Department of Cardiothoracic and Vascular Surgery, Westpfalz Klinikum, Kaiserslautern, Germany; eel Department of Cardiothoracic Surgery, University of Patras, Patras, Greece; eem Department of Integrated Hygiene, Organizational, and Service Activities, AOU Polyclinc “G.Rodolico-San Marco” Catania, Catania, Italy; een Univertisy of Catania, University of Catania, Catania, Italy; eeo Nutrition & Health Innovation Research Institute, Edith Cowan University, Perth, WA, Australia; eep Stockholm Resilience Centre, Stockholm University, Stockholm, Sweden; eeq Department of Health Promotion and Health Education, National Taiwan Normal University, Taipei, Taiwan; eer Third Hospital, Peking University, Beijing, China; ees International Centre for Future Health Systems, University of New South Wales, Sydney, NSW, Australia; eet Department of Epidemiology and Biostatistics, Peking University, Beijing, China; eeu School of Nursing and Health Sciences, Hong Kong Metropolitan University, Hong Kong, China; eev School of Management, Beijing University of Chinese Medicine, Beijing, China; eew Department of Medicine and Mental Health, University of Moratuwa, Sri Lanka, Moratuwa, Sri Lanka; eex Department of Population Health Sciences, Duke University, Durham, NC, USA; eey Durham Center of Innovation to Accelerate Discovery and Practice Transformation, US Department of Veterans Affairs (VA), Durham, NC, USA; eez School of Medicine, Universidad Espíritu Santo, Samborondón, Ecuador; ffa Vicerrectoría de Investigación y Postgrado, Universidad de Los Lagos, Osorno, Chile; ffb National Institutes of Health, University of the Philippines Manila, Manila, Philippines; ffc Center for Research and Innovation, Ateneo De Manila University, Pasig City, Philippines; ffd Department of Community Health, Shahrekord University of Medical Sciences, Shahrekord, Iran; ffe Social Determinants of Health Research Center, Shahrekord University of Medical Sciences, Shahrekord, Iran; fff Ashok & Rita Patel Institute of Physiotherapy, Charotar University of Science and Technology, Anand, India; ffg School of Medicine, Federal University of Juiz de Fora, Juiz de Fora, Brazil; ffh Department of Epidemiology, Army Medical University, Chongqing, China; ffi College of Engineering, Effat University, Jeddah, Saudi Arabia; ffj Management of Information Systems Department, The American College of Greece, Aghia Paraskevi, Greece; ffk Tulane School of Public Health and Tropical Medicine, Tulane University, New Orleans, LA, USA; ffl Faculty of Pharmacy, Tishk International University, Erbil, Iraq; ffm Department of Pharmaceutical Science, University of Eastern Piedmont, Novara, Italy; ffn Center for Global Health, University of Pennsylvania, Philadelphia, PA, USA; ffo Department of Periodontology, Pomeranian Medical University, Szczecin, Poland; ffp Department of One Health in Tropical Infectiousness Diseases, Jigjiga University, Jigjiga, Ethiopia; ffq College of Health Science, Amoud University, Borama, Somalia; ffr Research Center, Cihan University-Sulaimaniya, Sulaymaniyah, Iraq; ffs Edson College of Nursing and Health Innovation, Arizona State University, Phoenix, AZ, USA; fft Gene Solutions, Ho Chi Minh city, Viet Nam; ffu Physics Department, Faculty of Science, Al al-Bayt University, Mafraq, Jordan; ffv Department of Psychology, University of Sargodha, Sargodha, Pakistan; ffw Rabigh Faculty of Medicine, King Abdulaziz University, Jeddah, Saudi Arabia; ffx Corporate Nursing and Midwifery Research Department, Hamad Medical Corporation, Doha, Qatar; ffy Department of Epidemiology and Biostatistics, Isfahan University of Medical Sciences, Isfahan, Iran; ffz Biomedical Engineering Research Center (CREB), Universitat Politècnica de Catalunya (Barcelona Tech - UPC), Barcelona, Spain; gga Institute of Labour Economics, Leibniz University Hannover, Hannover, Germany; ggb RGS Econ, Ruhr Graduate School in Economics, Essen, Germany; ggc Health, Policy and Population Research Center (CIPPS), National Autonomous University of Mexico, Mexico City, Mexico; ggd Harvard University, Boston, VA, USA; gge Department of Community Medicine, Sri Balaji Vidyapeeth (Deemed to be University), Pondicherry, India; ggf Faculty of Humanities and Health Sciences, Curtin University, Sarawak, Malaysia; ggg Jeffrey Cheah School of Medicine and Health Sciences, Monash University, Subang Jaya, Malaysia; ggh Medical Scientist Training Program, Northwestern University, Chicago, IL, USA; ggi Department of Anatomy and Developmental Biology, Monash University, Clayton, VIC, Australia; ggj Department of Anatomy, Genetics and Biomedical Informatics, University of Colombo, Colombo, Sri Lanka; ggk Department of Health Policy Research, Public Health Foundation of India, Gurugram, India; ggl Institute of Population Health Sciences, University of Liverpool, Liverpool, England; ggm Department of Community Medicine, Apollo Institute of Medical Sciences and Research, Hyderabad, India; ggn Department of Maternal-Child Nursing and Public Health, Federal University of Minas Gerais, Belo Horizonte, Brazil; ggo Department of Prosthetic Dental Sciences, Jazan University, Jazan, Saudi Arabia; ggp Department of Epidemiology, Mahidol-Oxford Tropical Medicine Research Unit, Bangkok, Thailand; ggq Nuffield Department of Medicine, University of Oxford, Oxford, England; ggr Department of International Public Health, Liverpool School of Tropical Medicine, Liverpool, England; ggs Department of Social Medicine and Family, Dezful University of Medical Sciences, Dezful, Iran; ggt Australian Centre for Health Services Innovation, Queensland University of Technology, Kelvin Grove, QLD, Australia; ggu Digital Health and Informatics Directorate, Queensland Health, Brisbane, QLD, Australia; ggv Centre for Health Innovation and Policy, Noida, India; ggw Department of Dental Research Cell, Dr. D. Y. Patil University, Pune, India; ggx School of Medicine and Institute of Public Health, University of Gondar, Gondar, Ethiopia; ggy College of Medicine, Alfaisal University, Riyadh, Saudi Arabia; ggz Research & Innovation Center, Ministry of Health, Riyadh, Saudi Arabia; hha Department of Medical Microbiology and Immunology, Trinity Medical Sciences University, St. Vincent, Saint Vincent and the Grenadines; hhb Kasturba Medical College, Manipal Academy of Higher Education, Mangalore, India; hhc Department of Adult Health Nursing, Bahir Dar University, Bahir Dar, Ethiopia; hhd Debre Berhan university, Debre Berhan University, Debre Berhan, Ethiopia; hhe General Administration Department, Helsinki University Hospital, Helsinki, Finland; hhf School of Health Sciences, University of Melbourne, Melbourne, VIC, Australia; hhg Comprehensive Cancer Center, Helsinki University Hospital, Helsinki, Finland; hhh University of Helsinki, Helsinki, Finland; hhi Medical Area Department, University of Udine, Udine, Italy; hhj College of Pharmacy, Nawroz University, Duhok, Iraq; hhk University Centre Varazdin, University North, Varazdin, Croatia; hhl Department of Pharmacology, University of Kelaniya, Ragama, Sri Lanka; hhm Clinical Medicine Department, Colombo North Teaching Hospital, Ragama, Sri Lanka; hhn Department of Paediatrics, University of Kelaniya, Ragama, Sri Lanka; hho University Paediatrics Unit, Colombo North Teaching Hospital, Ragama, Sri Lanka; hhp Department of Pathology, Faculty of Veterinary Medicine, Zagazig University, Zagazig University, Zagazig, Egypt; hhq Faculty of Sciences, University of Buea, Cameroon, Buea, Cameroon; hhr Research Center for Public Health and Nutrition, National Research and Innovation Agency, Bogor District, Indonesia; hhs Multidisciplinary Department of Medical-Surgical and Dental Specialties, University of Campania Luigi Vanvitelli, Naples, Italy; hht Saveetha Dental College and Hospitals, Saveetha University, Chennai, India; hhu Department of Public Health Dentistry, Saveetha Institute of Medical and Technical Sciences (SIMATS), Chennai, India; hhv Global Institute of Public Health, Ananthapuri Hospitals and Research Institute, Trivandrum, India; hhw Department of Statistics and Econometrics, Bucharest University of Economic Studies, Bucharest, Romania; hhx Internal Medicine Department, Kyrgyz State Medical Academy, Bishkek, Kyrgyzstan; hhy Department of Atherosclerosis and Coronary Heart Disease, National Center of Cardiology and Internal Disease, Bishkek, Kyrgyzstan; hhz Thumbay College of Management and AI in Healthcare, Gulf Medical University, Ajman, United Arab Emirates; iia Research and Development Department, Panacea Institute of Interdisciplinary Research and Education, Varanasi, India; iib Department of Forensic Medicine and Toxicology, All India Institute of Medical Sciences, Patna, India; iic RAK College of Nursing, RAK Medicak and Health Sciences University, Ras Al-Khaimah, United Arab Emirates; iid Nursing College, Sohag University, Sohag, Egypt; iie Molecular Biology Unit, Sirius Training and Research Centre, Khartoum, Sudan; iif Bio-Statistical and Molecular Biology Department, Sirius Training and Research Centre, Khartoum, Sudan; iig Faculty of Medicine, University of Khartoum, Khartoum, Sudan; iih Department of Pathophysiology, St. George’s University, St.George’s, Grenada; iii Department of Biophysics, All India Institute of Medical Sciences, New Delhi, India; iij Centre For Interdisciplinary Research In Basic Sciences (CIRBSc), Jamia Millia Islamia, New Delhi, India; iik Social Determinants of Health Research Center, Tabriz University of Medical Sciences, Tabriz, Iran; iil Midwifery Department, Tabriz University of Medical Sciences, Tabriz, Iran; iim Department of Medicine, Government Medical College Kozhikode, Kozhikode, India; iin Health Systems and Policy Research Unit, Ahmadu Bello University, Zaria, Nigeria; iio Department of Biochemistry, Saveetha University, Chennai, India; iip School of Health Sciences, University of Petroleum and Energy Studies, Dehradun, India; iiq Department of Applied Biology, University of Science and Technology Meghalaya, Ri-Bhoi, India; iir Analytical and Applied Economics, Utkal University, Bhubaneswar, India; iis Department of Health Policy and Administration, College of Public Health, University of the Philippines Manila, Manila, Philippines; iit Department of Physiology, All India Institute of Medical Sciences, Deoghar, India; iiu AI & Cyber Futures Institute, Charles Sturt University, Bathurst, NSW, Australia; iiv The University of Queensland, Brisbane, QLD, Australia; iiw Department of Collective Prevention and Public Health, General Directorate for Personal Care, Health, and Welfare, Bologna, Italy; iix Social Determinants of Health Research Center, Yasuj University of Medical Sciences, Yasuj, Iran; iiy Department of Health Sciences (Ph.D. Program), Institute for Medical Assistance to State Public Servants, São Paulo, Brazil; iiz Division of Plastic and Reconstructive Surgery, University of Washington Medical Center, Seattle, WA, USA; jja Neurosciences Research Center (NSRC), Tabriz University of Medical Sciences, Tabriz, Iran; jjb Student Research Committee, Tabriz University of Medical Sciences, Tabriz, Iran; jjc Facultad de Salud, Universidad Santiago de Cali, Santiago de Cali, Colombia; jjd Department of Health Policy, London School of Economics and Political Science, London, England; jje Department of Surgery and Cancer, Imperial College London, London, England; jjf Antimicrobial Resistance Research Center, Iran University of Medical Sciences, Tehran, Iran; jjg Hazrat-e Rasool General Hospital, Iran University of Medical Sciences, Tehran, Iran; jjh Nutrology Fellowship, Nutrology Academy, São Paulo, Brazil; jji René Rachou Institute, Oswaldo Cruz Foundation, Belo Horizonte, Brazil; jjj PMAS Arid Agriculture University Rawalpindi, Rawalpindi, Pakistan; jjk Unit of Pharmacotherapy, Epidemiology and Economics, University of Groningen (Rijksuniversiteit Groningen), Groningen, Netherlands; jjl Department of Epidemiology and Biostatistics, Wuhan University, Wuhan, China; jjm College of Medicine and Health, University of Birmingham, Birmingham, England; jjn Department of Medicine, Integrated Medicine Programme, Alexandra Hospital, National University Health System, Singapore, Singapore; jjo Department of Surgery, General University Hospital of Patras, Patras, Greece; jjp Faculty of Medicine, University of Thessaly, Larissa, Greece; jjq Amity Institute of Pharmacy, Amity University, Noida, India; jjr Department of Paediatrics, Ahmadu Bello University, Zaria, Nigeria; jjs School of Public Health, Makerere University, Kampala, Uganda; jjt Department of Pediatrics & Pediatric Pulmonology, Institute of Mother & Child Care, Multan, Pakistan; jju Business Analytics and Artificial Intelligence, University of Texas, Richardson, TX, USA; jjv Irfan Suat Gunsel Operational Research Institute, Near East University, Mersin, Türkiye; jjw Department of Research Methods, Orthopaedic Research Group, Coimbatore, India; jjx Central Research Laboratory, Vinayaka Mission’s Research Foundation (Deemed to be University), Puducherry, India; jjy Rwanda Biomedical Centre, Kigali, Rwanda; jjz College of Medicine and Health Sciences, University of Rwanda, Kigali, Rwanda; kka Department of Public Health, Universitas Negeri Semarang, Semarang, Indonesia; kkb Department of Psychiatry, Seoul National University, Seoul, South Korea; kkc Department of Neuropsychiatry, Seoul National University Bundang Hospital, Seongnam, South Korea; kkd Elderly Health Research Center, Research and Academic Institution, Tehran, Iran; kke Neurosciences Research Center, Kurdistan University of Medical Sciences, Sanandaj, Iran; kkf Rajaei Cardiovascular Research Center, Iran University of Medical Sciences, Tehran, Iran; kkg Department of Medicine, University of British Columbia, Vancouver, BC, Canada; kkh Faculty of Pharmacy, Hasanuddin University, Makassar, Indonesia; kki College of Health Sciences, Cihan University Sulaimaniya, Sulaimaniya, Iraq; kkj University of Sulaimani, Sulaymaniyah, Iraq; kkk Department of Physiotherapy, Tehran University of Medical Sciences, Tehran, Iran; kkl Research Center for War-affected People, Tehran University of Medical Sciences, Tehran, Iran; kkm Department of Health and Rehabilitation Sciences, Prince Sattam bin Abdulaziz University, Al Kharj, Saudi Arabia; kkn Division of Endocrinology and Diabetes, University of Vermont, South Burlington, VT, USA; kko Postgraduate Institute of Medicine, University of Colombo, Colombo, Sri Lanka; kkp College of Public Health, Kent State University, Kent, OH, USA; kkq Department of Pediatrics, Arak University of Medical Sciences, Arak, Iran; kkr School of Allied Medicine, Shahid Beheshti University of Medical Sciences, Tehran, Iran; kks Department of General Surgery, Carol Davila University of Medicine and Pharmacy, Bucharest, Romania; kkt Department of General Surgery, Emergency University Hospital Bucharest, Bucharest, Romania; kku Department of Anatomy and Embryology, Carol Davila University of Medicine and Pharmacy, Bucharest, Romania; kkv Department of Cardiology, Cardio-Aid, Bucharest, Romania; kkw Department of Cardiology, University of Medicine and Pharmacy “Victor Babes”, Timisoara, Romania; kkx Rocordis Heart Center, Cardiology and Cardiovascular Surgery Hospital, Timisoara, Romania; kky Euromed Research Center, Euromed University of Fes, Fez, Morocco; kkz Faculty of Medicine, Pharmacy, and Dentistry, University Sidi Mohammed Ben Abdellah, Fez, Morocco; lla Department of Public Health, Erasmus University Medical Center, Rotterdam, Netherlands; llb Institute for Global Health Innovations, Duy Tan University, Da Nang, Viet Nam; llc International Institute of Training and Research (INSTAR), University of Medicine and Pharmacy, Vietnam National University (UMP-VNU), Hanoi, Viet Nam; lld Faculty of Public Health, VNU University of Medicine and Pharmacy, Hanoi, Viet Nam; lle Faculty of Medicine, Duy Tan University, Da Nang, Viet Nam; llf Institute for Mental Health Policy Research, Centre for Addiction and Mental Health, Toronto, ON, Canada; llg Global Health Policy Lab, Tohoku University, Miyagi, Japan; llh Department of Health Policy and Management, Keio University, Tokyo, Japan; lli Department of Microbiology and Molecular Genetics, The Women University Multan, Multan, Pakistan; llj College of Science- Biology Department, University of Hail, Hail, Saudi Arabia; llk Faculty of Medicine, Universiti Malaya, Kuala Lumpur, Malaysia; lll Department of Paediatrics, Nnamdi Azikiwe University, Awka, Nigeria; llm Department of Radiology, Mayo Clinic, Rochester, MN, USA; lln School of Information, University of California Berkeley, Berkeley, CA, USA; llo Department of Applied Economics and Quantitative Analysis, University of Bucharest, Bucharest, Romania; llp Bioinformatics Department, National Institute of Research and Development for Biological Sciences, Bucharest, Romania; llq Department of Pediatrics, University of Jos, Jos, Nigeria; llr Department of Pediatrics, Jos University Teaching Hospital, Jos, Nigeria; lls Department of Nursing Science, Ekiti State University, Ado-Ekiti, Ado-Ekiti, Nigeria; llt Department of Political Science, East Carolina University, Greenville, NC, USA; llu Department of Physiotherapy, Faculty of allied health sciences., Federal University of Health Sciences Ila Orangun Osun, Ila-Orangun, Nigeria; llv Department of Physiotherapy, University of KwaZulu-Natal, Durban, South Africa; llw Department of Medical Laboratory Science, Federal Neuropsychiatric Hospital, Abeokuta, Nigeria; llx School of Pharmacy, University of the Western Cape, Cape Town, South Africa; lly Department of Education Leadership and Management, University of Johannesburg, Johannesburg, South Africa; llz College of Health Sciences, Bowen University, Iwo, Nigeria; mma College of Medicine, University of Ibadan, Ibadan, Nigeria; mmb Department of Zoology, King Saud University, Riyadh, Saudi Arabia; mmc Department of Psychiatry and Behavioural Neurosciences, McMaster University, Hamilton, ON, Canada; mmd Department of Psychiatry, University of Lagos, Lagos, Nigeria; mme Department of Neurology, University College Hospital, Ibadan, Ibadan, Nigeria; mmf Department of Medicine, University of Ibadan, Ibadan, Nigeria; mmg Global Health, KIT Royal Tropical Institute, Amsterdam, Netherlands; mmh Cardiology Department, Federal University of Rio de Janeiro, Rio de Janeiro, Brazil; mmi Department of Community Medicine, Ahmadu Bello University, Zaria, Nigeria; mmj Centre for Healthy Start Initiative, Lagos, Nigeria; mmk Department of Pharmacology and Therapeutics, Olabisi Onabanjo University, Sagamu, Nigeria; mml Institute of Infectious Disease and Molecular Medicine, University of Cape Town, Cape Town, South Africa; mmm Jadara Research Center, Jadara University, Irbid, Jordan; mmn Department of Pharmacology and Therapeutics, University of Nigeria Nsukka, Enugu, Nigeria; mmo Scientific Laboratory “Center for Collective Use”, Kazakh National Medical University, Almaty, Kazakhstan; mmp Centre of Regenerative Medicine, Medical University of Bialystok, Bialystok, Poland; mmq Department of Microbiology and Immunology, University of Health and Allied Sciences, Ho, Ghana; mmr Sickle Cell Unit, Ho Teaching Hospital, Ho, Ghana; mms Department of Neurosurgery, University of California San Francisco, San Francisco, CA, USA; mmt One Health Research Group, Universidad De Las Américas, Quito, Ecuador; mmu Faculty of Health Sciences, Department of Medicine and Jos University Teaching Hospital, University of Jos, Jos, Nigeria; mmv Department of Biological Sciences, Njala University, Freetown, Sierra Leone; mmw Department of Medical Laboratory Science, University of Cape Coast, Cape Coast, Ghana; mmx Department of Public Health, University of Otago, Dunedin, New Zealand; mmy School of Nursing, University of California San Francisco, San Francisco, CA, USA; mmz Faculty of Medicine, University Ferhat Abbas of Setif, Setif, Algeria; nna Division of Infectious Diseases, University Hospital of Setif, Setif, Algeria; nnb Department of Global Public Health and Primary Care, University of Bergen, Bergen, Norway; nnc Department of Medicine, University College Hospital, Ibadan, Ibadan, Nigeria; nnd University Hospitals, Case Western Reserve University, Cleveland, OH, USA; nne Department of Biological Sciences, Bamidele Olumilua University of Education Science & Technology, Ikere-Ekiti, Nigeria; nnf Plant Systems Biology, International Center for Genetic Engineering & Biotechnology (ICGEB), Cape Town, South Africa; nng Irfan Suat Gunsel Operational Research Institute, Near East University, Nicosia, Türkiye; nnh Department of Forensic Medicine and Toxicology, Manipal Academy of Higher Education, Mangalore, India; nni Institute of Physiotherapy, Srinivas University, Mangalore, India; nnj Institute of Digital Health Sciences, Semmelweis University, Budapest, Hungary; nnk Department of Community Medicine, Iuliu Hatieganu University of Medicine and Pharmacy Cluj Napoca, Cluj Napoca, Romania; nnl National Center for Statistics in Public Health, National Institute of Public Health, Bucharest, Romania; nnm Centre for Biotechnology, Siksha ‘O’ Anusandhan Deemed to be University, Bhubaneswar, India; nnn Department of Pharmacy, Madan Bhandari Academy of Health Sciences, Hetauda, Nepal; nno Amity Institute of Forensic Sciences, Amity University, Noida, Gautam Buddh Nagar, India; nnp College of Nursing, Krida Wacana Christian University, Jakarta, Indonesia, Krida Wacana Christian University, Jakarta, Indonesia, Taipei, Indonesia; nnq Department of Diabetes, Nutrition and Metabolic Diseases, Carol Davila University of Medicine and Pharmacy, Bucharest, Romania; nnr Department of Medicine and Surgery, University of Bologna, Bologna, Italy; nns Medical University of Vienna, Vienna, Austria; nnt Vision and Eye Research Institute, Anglia Ruskin University, Cambridge, England; nnu Division of Health Policy and Management, University of Minnesota, Minneapolis, MN, USA; nnv Department of Sociology, Anthropology, and Public Health, University of Maryland Baltimore County, Baltimore, MD, USA; nnw Department of Psychiatry, All India Institute of Medical Sciences, Bhubaneswar, India; nnx Department of Medical Sciences, University of Torino, Torino, Italy; nny Department of Imaging, AOU Città della Salute e della Scienza di Torino (AOU City of Health and Science of Turin), Torino, Italy; nnz Department of Computer Science and Bioscience, Marwadi University, Rajkot, India; ooa Department of Cardiovascular Medicine, University of Tennessee, Nashville, TN, USA; oob College of Dental Medicine, Roseman University of Health Sciences, South Jordan, UT, USA; ooc Second Propedeutic Department of Internal Medicine, Aristotle University of Thessaloniki, Thessaloniki, Greece; ood Department of Human Anatomy, All India Institute of Medical Sciences, Bathinda, India; ooe Department of Community Medicine, Mallareddy Medical College for Women, Hyderabad, India; oof Faculty of Humanities and Social Sciences, Tribhuvan University, Kathmandu, Nepal; oog Department of Genetics, Yale University, New Haven, CT, USA; ooh Physiology Research Center, Iran University of Medical Sciences, Tehran, Iran; ooi Department of Physiology, Iran University of Medical Sciences, Tehran, Iran; ooj IRCCS Fondazione Don Carlo Gnocchi, Milan, Italy; ook Department of Clinical and Experimental Sciences, University of Brescia, Brescia, Italy; ool Australian Institute of Health Innovation, Macquarie University, Sydney, NSW, Australia; oom Department of Applied Nursing, Federal University of Minas Gerais, Belo Horizonte, Brazil; oon Department of Psychiatry, University of Sao Paulo, São Paulo, Brazil; ooo International Institute for Educational Planning (IIEP), Albert Einstein Hospital, São Paulo, Brazil; oop Department of Food, Environmental and Nutritional Sciences, University of Milan, Milan, Italy; ooq Mathematical and Computer Sciences, University of Medical Sciences, Ondo, Ondo, Nigeria; oor Research Advancement Consortium in Health, Hanoi, Viet Nam; oos Shanghai Mental Health Center, Shanghai Jiao Tong University, Shanghai, China; oot Departments of Psychiatry and Epidemiology, Columbia University, New York, NY, USA; oou Széchenyi István University, Győr, Pakistan; oov International Center of Medical Sciences Research (ICMSR), Islamabad, Pakistan; oow Department of Promoting Health, Maternal-Infant, Excellence and Internal and Specialized Medicine (PROMISE) G. D’Alessandro, University of Palermo, Palermo, Italy; oox Department of Promoting Health, Maternal-Infant, Excellence and Internal and Specialized Medicine (ProMISE) “G. D’Alessandro”, University of Palermo, Palermo, Italy; ooy Mental Health Research Institute, Tomsk National Research Medical Center, Tomsk, Russia; ooz Siberian State Medical University, Tomsk, Russia; ppa College of Health Sciences (CHS), VinUniversity, Hanoi, Viet Nam; ppb Management Department, Bucharest University of Economic Studies, Bucharest, Romania; ppc Academy of Romanian Scientists, Bucharest, Romania; ppd School of Medicine, Urmia University of Medical Sciences, Urmia, Iran; ppe Drug Applied Research Center, Tabriz University of Medical Sciences, Tabriz, Iran; ppf Department of Occupational Health and Safety Engineering, Ardabil University of Medical Science, Ardabil, Iran; ppg Department of Biochemistry, JSS Academy of Higher Education and Research, Mysuru, India; pph Centre for Dental Education and Research, All India Institute of Medical Sciences, New Delhi, India; ppi Department of Clinical and Experimental Medicine, University of Pisa, Pisa, Italy; ppj Department of Biostatistics, Epidemiology, and Informatics, University of Pennsylvania, Philadelphia, PA, USA; ppk Institute of Microbiology, Government College University Faisalabad, Faisalabad, Pakistan; ppl Department of Medical Instrumentation Techniques Engineering, Al-Rafidain University, Baghdad, Iraq; ppm Department of Mathematics and Informatics, Luhansk Taras Shevchenko National University, Luhansk, Ukraine; ppn Rory Meyers College of Nursing, New York University, New York, NY, USA; ppo Department of Biomaterials, Saveetha University, Chennai, India; ppp Department of Medical Oncology, Cancer Institute (W.I.A), Chennai, India; ppq Department of Epidemiology, National Institute of Mental Health and Neurosciences, Bengaluru, India; ppr Department of Environmental Health Engineering, Torbat Heydariyeh University of Medical Sciences, Torbat Heydariyeh, Iran; pps Health Science Research Centre, Torbat Heydariyeh University of Medical Sciences, Torbat Heydariyeh, Iran; ppt Iranian National Center for Addiction Studies, Tehran University of Medical Sciences, Tehran, Iran; ppu Sina Trauma and Surgery Research Center, Tehran University of Medical Sciences, Tehran, Iran; ppv Department of Public Health, Universitas Airlangga (Airlangga University), Surabaya, Indonesia; ppw College of Health Sciences, Cihan University-Sulaimaniya, Sulaymaniyah, Iraq; ppx Department of Biology, University of Sulaimani, Sulaymaniyah, Iraq; ppy Institute of Epidemiology, Disease Control and Research (IEDCR), Dhaka, Bangladesh; ppz Department of Population Science and Human Resource Development, University of Rajshahi, Rajshahi, Bangladesh; qqa Institute of Health and Wellbeing, Federation University Australia, Berwick, VIC, Australia; qqb School of Public Health and Preventive Medicine, Monash University, Melbourne, VIC, Australia; qqc Department of Cardiology, Dow University of Health Sciences, Karachi, Pakistan; qqd PHYSIOLOGY, Apollo Institute of Medical Sciences and Research, Chittoor, India; qqe Department of Clinical Pharmacology, Kazakh National Medical University, Almaty, Kazakhstan; qqf Department of Chemical Engineerinag and Procesiing, West Pomeranian University of Technology in Szczecin, Szczecin, Poland; qqg Department of Radiology, Stanford University, Stanford, CA, USA; qqh Department of Biotechnology, Hislop College, Nagpur, India; qqi Department of Molecular Biology & Genetic Engineering, RTM Nagpur University, Nagpur, India; qqj AIIMS, Bilaspur, All India Institute of Medical Sciences, Bilaspur, India; qqk Centre for Clinical Pharmacology, Military Medical Academy, Belgrade, Serbia; qql Department of Community Medicine, Rajiv Gandhi University, Karnataka, India; qqm Department of Health System Management Studies, JSS Academy of Higher Education & Research (Deemed to be University), Mysuru, India; qqn Department of Community Medicine, Manipal Academy of Higher Education, Manipal, India; qqo Nitte (Deemed to be University), AB Shetty Memorial Institute of Dental Sciences, Nitte University, Mangalore, India; qqp Department of Oral Pathology, Microbiology and Forensic Odontology, Sharavathi Dental College and Hospital, Shimogga, India; qqq Department of Family Medicine, Rajarata University of Sri Lanka, Anuradhapura, Sri Lanka; qqr Amity Institute of Public Health and Hospital Administration, Amity University, Uttar Pradesh, Uttar Pradesh, India; qqs Department of Health Services Management, Shiraz University of Medical Sciences, Shiraz, Iran; qqt Department of Public health, Amrita Institute of Medical Sciences, Ernakulam, India; qqu Department of Basic Medical Sciences, Jazan University, Jazan, Saudi Arabia; qqv WHO Collaborating Centre for Public Health Education and Training, Imperial College London, London, England; qqw Inovus Medical, St Helens, UK; qqx Academic Public Health England, Public Health England, London, England; qqy School of Health, Medical and Applied Sciences, CQ University, Sydney, NSW, Australia; qqz Department of Mathematical Demography & Statistics, International Institute for Population Sciences, Mumbai, India; rra Interventional Cardiology Department, University of Kentucky, morehead, KY, USA; rrb Infection Prevention & Control, King Saud bin Abdulaziz University for Health Sciences, Riyadh, Saudi Arabia; rrc Department of Hematology, North Khorasan University of Medical Sciences, Bojnurd, Iran; rrd Department of Hematology, Tarbiat Modares University, Tehran, Iran; rre IDEAS Research Group, Universidad Científica del Sur, Lima, Peru; rrf Kohat University of Science and Technology, Kohat University of Science and Technology, Kohat, Pakistan; rrg Natural and Medical Sciences Research Center, Natural and Medical Sciences Research Center, Nizwa, Oman; rrh Melbourne School of Population and Global Health, University of Melbourne, Melbourne, VIC, Australia; rri Yenepoya Research Centre, Yenepoya University, Mangalore, India; rrj School of Optometry and Vision Science, University of New South Wales, Sydney, NSW, Australia; rrk Brien Holden Vision Institute, Sydney, NSW, Australia; rrl Department of Obstetrics and Gynecology, Azienda Sanitaria Universitaria Friuli Centrale, Udine, Italy; rrm Unisabana Center for Translational Science, Universidad de La Sabana (Savannah University), Chia, Colombia; rrn Critical Care Department, Clinica Universidad De La Sabana (Savannah University Clinic), Chia, Colombia; rro Research Center for Immunodeficiencies, Tehran University of Medical Sciences, Tehran, Iran; rrp Network of Immunity in Infection, Malignancy and Autoimmunity (NIIMA), Universal Scientific Education and Research Network (USERN), Tehran, Iran; rrq Department of Epidemiology and Biostatistics, Health School, Occupational Environment Research Center, Rafsanjan University of Medical Sciences, Rafsanjan, Iran; rrr Department of Pharmacy, Shaheed Benazir Bhutto University Sheringal Pakistan, Dir Upper, Pakistan; rrs Department of Surgery, University of Minnesota, Minneapolis, MN, USA; rrt Department of Surgery, University Teaching Hospital of Kigali, Kigali, Rwanda; rru Department of Pharmacology and Toxicology, University of Antioquia, Medellin, Colombia; rrv Warwick Medical School, University of Warwick, Coventry, England; rrw Department of Clinical Research, University of Sao Paulo, Ribeirão Preto, Brazil; rrx Department of Community Medicine, Government Medical College, Chandigarh, India; rry Miyan Research Institute, International University of Business Agriculture and Technology, Dhaka, Bangladesh; rrz College of Medicine, University of Florida, Gainesville, FL, USA; ssa Department of Analytical and Applied Economics, Utkal University, Bhubaneswar, India; ssb RUSA Centre of Excellence in Public Policy and Governance, Utkal University, Bhubaneswar, India; ssc Department of Community Medicine, RVM Medical College and Research Centre, Hyderabad, Hyderabad, India; ssd Achutha Menon Centre for Health Science Studies, Sree Chitra Tirunal Institute for Medical Sciences and Technology, Thiruvananthapuram, India; sse Manipal Tata Medical College, Manipal Academy of Higher Education, Manipal, India; ssf Department of Public Health, New Mexico State University, Las Cruces, NM, USA; ssg Department of Internal Medicine, Muhimbili University of Health and Allied Sciences, Dar es Salaam, Tanzania; ssh Department of Internal Medicine, University of Botswana, Gaborone, Botswana; ssi Department of Oral and Maxillofacial Surgery, Jagadguru Sri Shivarathreeswara University, Mysore, India; ssj Cardiovascular Department, Zagazig University, Zagazig, Egypt; ssk Department of Medical Physics, Isfahan University of Medical Sciences, Isfahan, Iran; ssl Department of Food Technology, Salahaddin University, Erbil, Iraq; ssm Department of Nutrition and Dietetics, Cihan University-Erbil, Erbil, Iraq; ssn Tabriz University of Medical Sciences, Tabriz, Iran; sso Faculty of Advanced Technologies in Medicine, Iran University of Medical Sciences, Tehran, Iran; ssp Nuroscience Research Center, Trauma Institute, Guilan University of Medical Sciences, Rasht, Iran; ssq Department of Pharmaceutical Chemistry, International Medical University, Gdańsk, Poland; ssr Széchenyi István University, Széchenyi István University, Győr, Hungary; sss Irfan Suat Gunsel Operational Research Institute, Near East University, Turkey, Nicosia, Türkiye; sst Department of Nursing and Midwifery, Saveh University of Medical Sciences, Saveh, Iran; ssu Global Health Economy Research Team, Kiel Institute for the World Economy, Kiel, Germany; ssv Endocrinology and Metabolism Population Sciences Institute, Tehran University of Medical Sciences, Tehran, Iran; ssw Department of Community Medicine and Public Health, King Edward Medical University, Lahore, Pakistan; ssx Department of Psychiatry, All India Institute of Medical Sciences, New Delhi, India; ssy Department of Epidemiology, Indian Council of Medical Research, Kolkata, India; ssz Sharjah Institute of Medical Sciences, University of Sharjah, Sharjah, United Arab Emirates; tta Department of Radiation Oncology, All India Institute of Medical Sciences, New Delhi, India; ttb Centre for Biotechnology, Siksha ‘O’ Anusandhan (Deemed to be University), Bhubaneswar, India; ttc Canadian Red Cross, Red Cross, Ottawa, ON, Canada; ttd College of Pharmacy, Al-Hadba University, Mosul, Iraq; tte Department of Statistics, University of Gujrat, Gujrat, Pakistan; ttf LMU-Munich, Munich, Germany; ttg Institute for Employment Research, Nuremberg, Germany; tth Cephas Health Research Initiative Inc, Ibadan, Nigeria; tti Cumming School of Medicine, University of Calgary, Calgary, AB, Canada; ttj Department of Human Nutrition & Dietetics, Health Services Academy, Islamabad, Pakistan; ttk Faculty of Pharmacy, Mansoura University, Mansoura, Egypt; ttl Department of Biochemistry, University of Medical Sciences, Ondo, Ondo city, Nigeria; ttm Department of Research and Development, Tehran University of Medical Sciences, Tehran, Iran; ttn Department of Global Initiatives, Child Mind Institute, New York, NY, USA; tto Department of Psychiatry and Legal Medicine, Federal University of Rio Grande do Sul, Porto Alegre, Brazil; ttp SRM School of Public Health, Sri Ramaswamy Memorial Institute of Science and Technology, Kattankulathur, India; ttq Department of Entomology, Ain Shams University, Cairo, Egypt; ttr Medical Ain Shams Research Institute (MASRI), Ain Shams University, Cairo, Egypt; tts Department of Epidemiology and Biostatistics, Tehran University of Medical Sciences, Tehran, Iran; ttt Department of Genomics and Biotechnology, Madha Medical College and Research Institute, Chennai, India; ttu University of São Paulo City, São Paulo, Brazil; ttv School of Public Health and Health Management, University of Belgrade, Belgrade, Serbia; ttw Department of Biochemistry and Molecular Biology, Obafemi Awolowo University, Ile-Ife, Nigeria; ttx Independent Consultant, Thiruvananthapuram, India; tty Department of Public Health, Jahrom University of Medical Sciences, Jahrom, Iran; ttz Health Policy Research Center, Shiraz University of Medical Sciences, Shiraz, Iran; uua Botany Department, Bodoland University, Kokrajhar, India; uub Faculty of Science, Queensland University of Technology, Brisbane, QLD, Australia; uuc Health Sciences Research Center, Torbat Heydariyeh University of Medical Sciences, Torbat Heydariyeh, Iran; uud Department of Oral Pathology and Microbiology, Dr. D. Y. Patil Vidyapeeth, Pune (Deemed to be University), Pune, India; uue Department of Medical and Surgical Sciences, University of Bologna, Bologna, Italy; uuf Department of Epidemiology, National Institute for Research in Tuberculosis, Chennai, India; uug UGC Centre of Advanced Study in Psychology, Utkal University, Bhubaneswar, India; uuh Udyam-Global Association for Sustainable Development, Bhubaneswar, India; uui Carnegie Mellon University, Carnegie Mellon University, Pittsburgh, PA, USA; uuj Department of Public Health Sciences, Coastal Carolina University, Conway, SC, USA; uuk Harvard Extension School, Harvard University, Cambridge, MA, USA; uul Department of Educational Sciences, Farhangian University, Kermanshah, Iran; uum Substance Abuse Prevention Research Center, Kermanshah University of Medical Sciences, Kermanshah, Iran; uun Chemistry Department, Purdue University, West Lafayette, IN, USA; uuo Universidad ESAN, Universidad ESAN, Lima, Peru; uup Graduate School of Business, University of the Fraser Valley, Abbotsford, BC, Canada; uuq Department of Psychiatry, Stellenbosch University, Cape Town, South Africa; uur Department of Marine Biotechnology, AMET University, Chennai, India; uus Faculty of Dentistry, University of Puthisastra, Phnom Penh, Cambodia; uut School of Medicine, Nazarbayev University, Astana, Kazakhstan; uuu Emergency Department, Manian Medical Centre, Erode, India; uuv Digestive Diseases Research Institute, Tehran University of Medical Sciences, Tehran, Iran; uuw Department of Psychology and Health Sciences Faculty of Human Sciences, Education, and Sports, Pegaso Telematic University, Naples, Italy; uux Department of Medicine, Swami Vivekanand Subharti University, Meerut, India; uuy Division of Population Medicine, Cardiff University, Cardiff, Wales; uuz Charotar University of Science and Technology (CHARUSAT), Ashok and Rita Patel Institute of Physiotherapy, Changa, India; vva Rita A. Patel Institute of Physiotherapy, The Charutar Vidya Mandal(CVM) University, Anand, India; vvb Dongguan Key Laboratory of Computer-Aided Drug Design, Guangdong Medical University, Dongguan, China; vvc State Key Laboratories of Chemical Resources Engineering, Beijing University Of Chemical Technology, Beijing, China; vvd Research Centre for Health Sciences (RCHS), The University of Lahore, Lahore, Pakistan; vve Department of Physics, The University of Lahore, Lahore, Pakistan; vvf Reasearch Centre for Health Sciences (RCHS), The University of Lahore; vvg National Institute of Gastroenterology-IRCCS “Saverio de Bellis”, Castellana Grotte, Italy; vvh Department of Internal Medicine, Tehran University of Medical Sciences, Tehran, Iran; vvi Department of Transitional Residency, Lakeland Regional Health, lakeland, FL, USA; vvj Center for Medical and Bio-Allied Health Sciences Research, Ajman University, Ajman, United Arab Emirates; vvk School of Public health, Health Services Academy, Islamabad, Pakistan; vvl Independent Consultant, Karachi, Pakistan; vvm Noncommunicable Diseases Research Center, Neyshabur University of Medical Sciences, Neyshabur, Iran; vvn Science Department, Kazakh National Medical University, Almaty, Kazakhstan; vvo Lancaster University, Lancaster, England; vvp Columbia University, New York, NY, USA; vvq Department of Microbiology and Molecular Genetics, University of Vermont, Burlington, VT, USA; vvr Department of Microbiology, Jahangirnagar University, Dhaka, Bangladesh; vvs Department for Evidence-based Medicine and Evaluation, University for Continuing Education Krems, Krems, Austria; vvt School of Science and Technology, The University of Georgia, Tbilisi, Georgia; vvu Amity Institute of Biotechnology, Amity University Rajasthan, Jaipur, India; vvv Department of Forensic Science, Shree Guru Gobind Singh Tricentenary University, Gurugram, India; vvw Department of Physiotherapy, Galgotas University, Greater Noida, India; vvx UN Mehta Institute of Cardiology and Research Center, B.J. Medical College, Ahmedabad, India; vvy Department of Cardiology, Government Medical College, Ahmedabad, India; vvz Division of Physical Sciences, Karunya Institute of Technology and Sciences, Tamil Nadu, India; wwa Department of Social and Behavioral Health, University of Nevada Las Vegas, Las Vegas, NV, USA; wwb Department of Forensic Science, Panjab University, Chandigarh, India; wwc Department of Physiology, Pharmacology, and Toxicology, An-Najah National University, Nablus, Palestine; wwd American University of the Middle East, Egaila, Kuwait; wwe University Institute of Medical Lab Technology, The University of Lahore, Lahore, Pakistan; wwf School of Public Health, Tehran University of Medical Sciences, Tehran, Iran; wwg Quantitative Department, Non-Communicable Diseases Research Center (NCDRC), Tehran, Iran; wwh Department of Pharmacology, Manipal Academy of Higher Education, Manipal, India; wwi Department of Microbiology, Kasturba Medical College, Mangalore, India; wwj Department of Biology, Morgan State University, Baltimore, MD, USA; wwk Department of Environmental Health Sciences, Tulane University, New Orleans, LA, USA; wwl Alva’s Institute of Medical Sciences & Research Centre, Rajiv Gandhi University of Health Sciences, Moodubidire, India; wwm KS Hegde Medical Academy, Nitte University, Mangalore, India; wwn Manipal College of Dental Sciences, Mangalore, Manipal Academy of Higher Education, Manipal, India; wwo Rural Health Research Institute, Charles Sturt University, Orange, Austria; wwp Department of Pharmacology, Saint Paul’s Hospital Millennium Medical College, Addis Ababa, Ethiopia; wwq Department of Physiology, Manipal Academy of Higher Education, Manipal, India; wwr Department of Orthodontics, Boston University, Boston, MA, USA; wws Department of Veterinary Public Health and Preventive Medicine, Usmanu Danfodiyo University, Sokoto, Sokoto, Nigeria; wwt School of Biotechnology, Gautam Buddha University, gautam buddh nagar, India; wwu Social Determinants of Health Research Center, Kurdistan University of Medical Sciences, Sanandaj, Iran; wwv Center for Technology and Innovation in Cardiovascular Informatics, Iran University of Medical Sciences, Tehran, Iran; www Department of Medical-Surgical Nursing, Mazandaran University of Medical Sciences, Sari, Iran; wwx Department of Nursing and Health Sciences, Flinders University, Adelaide, SA, Australia; wwy Department of Research and Academics, Kathmandu Cancer Center, Bhaktapur, Nepal; wwz Person-Centred Research Directorate, Eastern Health Clinical, School Faculty of Medicine, Nursing and Health Sciences, Monash University, Box Hill, VIC, Australia; xxa Department of Pharmaceutical Microbiology and Biotechnology, Usmanu Danfodiyo University, Sokoto, Sokoto, Nigeria; xxb Health Systems and Population Health, University of Washington, Seattle, WA, USA; xxc School of Public Health, Arba Minch University, Arba Minch, Ethiopia; xxd Unit of Basic Medical Sciences, University of Khartoum, Khartoum, Sudan; xxe Department of Medical Microbiology and Infectious Diseases, Erasmus University, Rotterdam, Netherlands; xxf Faculty of Dentistry, Federal University of Minas Gerais, Belo Horizonte, Brazil; xxg Research and Innovation, University of Chester, Chester, UK; xxh College of Health and Allied Sciences, University of Cape Coast, Cape Coast, Ghana; xxi Global Brain Health Institute, University of California San Francisco, San Francisco, CA, USA; xxj School of Health, Victoria University of Wellington, Wellington, New Zealand; xxk Usher Institute, University of Edinburgh, Edinburgh, Scotland; xxl Department of Dentistry, All India Institute of Medical Sciences, Bhopal, India; xxm Department of Biochemistry, Central University of Punjab, Bathinda, India; xxn Department of Forensic Medicine and Toxicology, Rajendra Institute of Medical Sciences, Ranchi, India; xxo University Centre for Research and Development (UCRD), Chandigarh University, Mohali, India; xxp Department of Pharmacology, Government Medical College and Hospital, Chandigarh, India; xxq School of Medicine, Baylor College of Medicine, Houston, TX, USA; xxr Department of Medicine Service, US Department of Veterans Affairs (VA), Houston, TX, USA; xxs Department of Psychiatry, All India Institute of Medical Sciences, Bathinda, India; xxt Medical Oncology Lab, All India Institute of Medical Sciences, New Delhi, India; xxu Department of Human Genetics, Punjabi University, Patiala, Patiala, India; xxv Biotechnology, Microbiology and Forensic Science, Noida International University, Greater Noida, India; xxw Mehta Family School of Biosciences and Biomedical Engineering, Indian Institute of Technology Indore, Indore, India; xxx ESIC Medical College and Hospital, Ranchi, India; xxy SRM Medical College Hospital and Research Centre, Sri Ramaswamy Memorial Institute of Science and Technology, Chengalpettu, India; xxz Global and European Health Education and Study Institute, University of Georgia, Tbilisi, Georgia; yya National Center for Disease Control and Public Health, Tbilisi, Georgia; yyb Books Committee, Royal College of Psychiatrists, London, UK; yyc Biology Department, University of Hail, Hail, Saudi Arabia; yyd Department of Public Health, Jazan University, Jazan, Saudi Arabia; yye Department of Development Studies, Daffodil International University, Dhaka, Bangladesh; yyf Faculty of Public Health, Universitas Ahmad Dahlan, Yogyakarta, Indonesia; yyg Department of Oral and Maxillofacial Surgery, the Affiliated Stomatological Hospital of Guizhou Medical University, Guiyang, China; yyh 3rd Department of Cardiology, University of Athens, Athens, Greece; yyi Department of Health, A.C.S. Medical College and Hospital, Hyderabad, India; yyj College of Health and Public Service, University of North Texas, Denton, TX, USA; yyk Department of Psychiatry and Psychotherapy, Medical University of Vienna, Comprehensive Center for Clinical Neurosciences and Mental Health (C3NMH), Vienna, Austria; yyl Division of Psychology and Mental Health, University of Manchester, Manchester, England; yym Discipline of Physiotherapy, University of Technology Sydney, Sydney, NSW, Australia; yyn McGill University, Montreal, QC, Canada; yyo Department of Orthopaedics, Massachusetts General Hospital, Boston, MA, USA; yyp Department of Orthopaedics, Harvard University, Cambridge, MA, USA; yyq Department of Medical Sciences, Sunway University, Subang Jaya, Malaysia; yyr Faculty of Management, Sri Ramaswamy Memorial Institute of Science and Technology, Tiruchirappalli, India; yys Hospital Administration, Sanjay Gandhi Postgraduate Institute of Medical Sciences, Lucknow, India; yyt Hospital Administration, King George’s Medical University, Lucknow, India; yyu Department of Pathology, Medical Laboratory CSD, Kyiv, Ukraine; yyv Research Unit, Ukrainian Association for Precision Medicine, Kyiv, Ukraine; yyw Department of Medicine, Yobe State University Teaching Hospital, Yobe, Nigeria; yyx Department of Social Sciences, University of Nicosia, Nicosia, Cyprus; yyy Department of Life and Health Sciences, University of Nicosia, Nicosia, Cyprus; yyz GROW- Institute for Oncology and Reproduction & NUTRIM - Institute for Nutrition and Translational Research, Maastricht University, Maastricht, Netherlands; zza School of Public Health, Kunming Medical University, Kunming, China; zzb Department of Population Health Sciences, University College London, London, England; zzc Institute of Medical Sciences, The John Paul II Catholic University of Lublin, Lublin, Poland; zzd Department of Neurology, Neurocenter of Southern Switzerland (NSI), Lugano, Switzerland; zze Department of Medicine, University of Valencia, Valencia, Spain; zzf Carlos III Health Institute, Biomedical Research Networking Center for Mental Health Network (CiberSAM), Madrid, Spain; zzg Department of Basic Medical Sciences, Islamic Azad University, Mashhad, Iran; zzh Department of Internal Medicine, Islamic Azad University, Mashhad, Iran; zzi Department of Medical Education, Shahid Beheshti University of Medical Sciences, Tehran, Iran; zzj Department of Health, Safety, and Environmental Management, Abadan University of Medical Sciences, Abadan, Iran; zzk Department of Pharmacy, Mangalayatan University, Aligarh, India; zzl Children’s Hospital of Philadelphia, University of Pennsylvania, Philadelphia, PA, USA; zzm Duhok Research Centre, University of Duhok, Duhok, Iraq; zzn Department of Psychiatry and Behavioral Sciences, Stanford University, Palo Alto, CA, USA; zzo Department of Environmental, Agricultural and Occupational Health, University of Nebraska Medical Center, Omaha, NE, USA; zzp Department of Dermatology, Carol Davila University of Medicine and Pharmacy, Bucharest, Romania; zzq Department of Dermato-Venereology, Dr. Victor Babes Clinical Hospital of Infectious Diseases and Tropical Diseases, Bucharest, Romania; zzr Zhujiang Hospital, Southern Medical University, Guangzhou, China; zzs The First Affiliated Hospital of Chongqing Medical University, Chongqing Medical University, Chongqing, China; zzt Department of Surgery, National University of Singapore, Singapore, Singapore; zzu Department of Morphophysiology, South Kazakhstan Medical Academy, Shymkent, Kazakhstan; zzv Department of Science, University of British Columbia, Vancouver, BC, Canada; zzw Department of Medicine, Kazakh National Medical University, Almaty, Kazakhstan; zzx School of Medicine, Duke University, Durham, NC, USA; zzy American Heart Association Comprehensive Hypertension Center, University of California Irvine, Orange, CA, USA; zzz Excellence Center for Organ Transplantation, Mahidol University, Bangkok, Thailand; aaaa Department of Software Engineering, Foundation University Islamabad, Islamabad, Pakistan; aaab AI Developer, National University of Science and Technology, Islamabad, Pakistan; aaac Xiangya School of Nursing, Central South University, Changsha, China; aaad Department of Public Health and Informatics, Bangladesh Medical University, Dhaka, Bangladesh; aaae Taking Our Best Shot, Houston, TX, USA; aaaf Department of Research and Innovation, Enventure Medical Innovation, Houston, TX, USA; aaag Department of Pathology, University of Texas, Galveston, TX, USA; aaah Department of Science, Kazakh National Medical University, Almaty, Kazakhstan; aaai College of Health Sciences, Mekelle University, Mekelle, Ethiopia; aaaj Amrita Vishwa Vidyapeetham, Kochi, India; aaak Department of Economics, The American University in Cairo, Cairo, Egypt; aaal Institute of Applied Health Research, University of Birmingham, Birmingham, England; aaam Faculty of Medicine, University of Southampton, Southampton, England; aaan Department of Paediatric and Neonatal Sciences, Saveetha College of Physiotherapy, Saveetha Institute of Medical and Technical Sciences, Saveetha University, Chennai, India; aaao Department of Applied Bioscience, Konkuk University, Seoul, South Korea; aaap Department of Neonatology, Saveetha University, Chennai, India; aaaq Faculty of Public Health, Universitas Sam Ratulangi (Sam Ratulangi University), Manado, Indonesia; aaar К.А. Timiryazev Institute of Plant Physiology, Russian Academy of Sciences, Moscow, Russia; aaas Department of Physiology, All India Institute of Medical Sciences, New Delhi, India; aaat Systems Neuroscience, Tohoku University, Sendai, Japan; aaau Department of Prosthetic Dentistry, Kazakh National Medical University, Almaty, Kazakhstan; aaav College of Nursing, Kaohsiung Medical University, Taiwan, Kaohsiung, Taiwan; aaaw Interdisciplinary Health Data Center, Jagiellonian University Medical College, Kraków, Poland; aaax James Cook University, Douglas, QLD, Australia; aaay Centre for Infectious Disease Research in Zambia (CIDRZ), Lusaka, Zambia; aaaz Second Department of Internal Medicine, Kansai Medical University, Hirakata, Japan; bbba Department of Internal Medicine, University of Medicine and Pharmacy at Ho Chi Minh City, Ho Chi Minh City, Viet Nam; bbbb Department of Business Analytics, University of Massachusetts Dartmouth, Dartmouth, MA, USA; bbbc Research and Advocacy Initiative, ALS Vietnam, Quang Ngai, Viet Nam; bbbd College of Health Science, Vin University, Hanoi, Viet Nam; bbbe Department of Population Health, Hong Kong Metropolitan University, Hong Kong, China; bbbf Tianjin Key Laboratory of Ionic-Molecular Function of Cardiovascular Disease, Second Hospital of Tianjin Medical University, Tianjin, China; bbbg Laboratory of Clinical Pharmacology, Aristotle University of Thessaloniki, Thessaloniki, Greece; bbbh Department of Epidemiology and Public Health, University College London, London, England; bbbi Department of Biomedical Data Sciences, Stanford University, Palo Alto, CA, USA; bbbj King’s College London, London, England; bbbk Imperial College London, London, England; bbbl Department of Biological Sciences, National University of Medical Sciences (NUMS), Rawalpindi/ Islamabad, Pakistan; bbbm International Center for Chemical and Biological Sciences, University of Karachi, Karachi, Pakistan; bbbn Department of Biology and Biochemistry, University of Houston, Houston, TX, USA; bbbo Department of Oncology, Federal Medical Centre, Gusau, Nigeria; bbbp Department of Physiotherapy, Bayero University, Kano, Nigeria; bbbq Department of Rehabilitation Sciences, Hong Kong Polytechnic University, Hong Kong, China; bbbr Research Institute for Medical and Health Sciences, University of Sharjah, Sharjah, United Arab Emirates; bbbs Department of Orthodontics, University of Trakya, Edirne, Türkiye; bbbt Sociedad Argentina de Medicina, Buenos Aires, Argentina; bbbu Hospital Vélez Sarsfield, Buenos Aires, Argentina; bbbv Institute for Global Brain Health, Trinity College Dublin, Dublin, Ireland; bbbw Department of Nephrology, Christian Medical College and Hospital (CMC), Vellore, India; bbbx UKK Institute, Tampere, Finland; bbby Faculty of Medicine and Health Technology, Tampere University, Tampere, Finland; bbbz Department of Communicable Diseases, Indian Council of Medical Research, New Delhi, India; ccca School of Epidemioloy, Indian Institute of Health Management Research University, Jaipur, India; cccb Department of Zoology, Central University of Punjab, Bathinda, India; cccc Department of Human Genetics & Molecular Biology, Bharathiar University, Coimbatore, India; cccd Department of Community Medicine and Family Medicine, All India Institute of Medical Sciences, Gorakhpur, India; ccce Raffles Neuroscience Centre, Raffles Hospital, Singapore, Singapore; cccf Yong Loo Lin School of Medicine, National University of Singapore, Singapore, Singapore; cccg Digital Health Research Center, Digital Health S.A., Lima, Peru; ccch Universidad Científica del Sur, Lima, Peru; ccci Radcliffe Department of Medicine, University of Oxford, Oxford, England; cccj Faculty of Medicine of Itajubá -FMIT, Itajubá, Brazil; ccck Universidade do Vale do Sapucaí - UNIVÁS, UNIVÁS, Pouso Alegre, Brazil; cccl Department of Health Care Administration and Economics, National Research University Higher School of Economics, Moscow, Russia; cccm Institute of Family Medicine and Public Health, University of Tartu, Tartu, Estonia; cccn Heidelberg Institute of Global Health, Section for Oral Health, Heidelberg University Hospital, Heidelberg, Germany; ccco Department of Medical Oncology, University of Medicine and Pharmacy “Grigore T Popa” Iasi, Iaşi, Romania; cccp Department of Medical Oncology, Regional Institute of Oncology, Iaşi, Romania; cccq International Institute for Training and Research (INSTAR), VNU University of Medicine and Pharmacy, Hanoi, Viet Nam; cccr Egyetem Square 1, Széchenyi István University, H-9026 Győr, Hungary; cccs Department of Medical Genetics, University of Cambridge, Cambridge, England; ccct School of Medicine, Georgetown University, Arlington, VA, USA; cccu School of Chinese Medicine, Beijing University of Chinese Medicine, Beijing, China; cccv University of Chicago, Chicago, IL, USA; cccw Stomatological Hospital, Military Medical University, Xi’an, China; cccx Department of Artificial Intelligence, Xiamen University, Xiamen, China; cccy Department of Neurosurgery, Beijing Tiantan Hospital, Beijing, China; cccz Department of Neurosurgery, Capital Medical University, Beijing, China; ddda National Institute of Health Data Science, Peking University, Beijing, China; dddb School of Public Health, Xuzhou Medical University, Xuzhou, China; dddc Enze Medical Health Academy, Taizhou Hospital of Zhejiang Province, Taizhou, China; dddd School of Public Health, Zhengzhou University, Zhengzhou, China; ddde School of Life Course and Population Sciences, King’s College London, London, England; dddf School of Public Health, Peking University, Beijing, China; dddg School of Nursing Sciences, University of Nairobi, Nairobi, Kenya; dddh School of Society and Culture, Adelaide University, Adelaide, SA, Australia; dddi Department of Medical Surgical Nursing, Gadjah Mada University, Yogyakarta, Indonesia; dddj Department of Community Medicine, Rajarata University of Sri Lanka, Anuradhapura, Sri Lanka; dddk Department of Clinical Neurosciences, University of Calgary, Calgary, AB, Canada; dddl Department of Community Health Sciences, University of Calgary, Calgary, AB, Canada; dddm Department of Nursing, Universitas Aisyiyah Bandung, Bandung, Indonesia; dddn Legon Centre for Education Research and Policy, University of Ghana, Accra, Ghana; dddo Institute of Health and Care Sciences, University of Gothenburg, Gothenburg, Sweden; dddp Faculty of Health Sciences, Oslo Metropolitan University, Oslo, Norway; dddq Western Institute of Digital-Intelligent Medicine, Chongqing Medical University, Chongqing, China; dddr Department of Intelligent Medical Engineering, Anhui Medical University, Anhui, China; ddds Department of Surgery, The First Affiliated Hospital of Anhui Medical University, Hefei, Anhui, China; dddt Department of Occupational Diseases, Linyi People’s Hospital, Linyi, China; dddu Department of General Medicine, Juntendo University, Tokyo, Japan; dddv Institute of Science Tokyo, Tokyo, Japan; dddw School of Traditional Chinese Medicine, Beijing University of Chinese Medicine, Beijing, China; dddx Pritzker School of Medicine, University of Chicago, Chicago, IL, USA; dddy College of Pharmacy and Health Science, Other, Ajman, United Arab Emirates; dddz Division of Public Health, The University of Osaka, Suita, Japan; eeea The George Institute for Global Health, Imperial College London, London, England; eeeb SDU University, Astana, Kazakhstan; eeec Department of Family Medicine, St. Paul’s Hospital Millennium Medical College, Addis Ababa, Ethiopia; eeed Family Medicine Department, St. Peter’s Specialized Hospital, Addis Ababa, Ethiopia; eeee Innovation and Research, King Faisal Specialist Hospital & Research Center, Riyadh, Saudi Arabia; eeef Department of Respiratory Medicine, Military Medical University, Chongqing, China; eeeg Department of Epidemiology, Xuzhou Medical University, Xuzhou, China; eeeh Nankai University, Other, Tianjin, China; eeei Department of Pharmacology, Bahir Dar University, Bahir Dar, Ethiopia; eeej Department of Pediatrics, Kyung Hee University, Seoul, South Korea; eeek Department of Biostatistics, University of Toyama, Toyama, Japan; eeel Department of Public Health, Juntendo University, Tokyo, Japan; eeem Department of Health Policy and Management, Jackson State University, Jackson, MS, USA; eeen School of Business & Economics, Tsinghua University, Beijing, China; eeeo School of Public Health, University of Alabama, Birmingham, AL, USA; eeep School of Public Health, Hubei University of Medicine, Shiyan, China; eeeq Department of Breast Surgery, Harbin Medical University Cancer Hospital, Harbin, China; eeer Department of Basic Science, University of Hail, Hail, Saudi Arabia; eees Association for Socially Applicable Research (ASAR), Pune, India; eeet Department of Emergency Medicine, Global Emergency Medicine Innovation and Implementation (GEMINI) Research Center, Durham, NC, USA; eeeu Islamic Azad University, Tehran, Iran; eeev Department of Oncology and Mammology, Kazakh Russian Medical University, Almaty, Kazakhstan; eeew Health Investigation Center, Universidad Católica Boliviana San Pablo, Tarija, Bolivia; eeex Nursing Care Research Center in Chronic Diseases, Ahvaz Jundishapur University of Medical Sciences, Ahvaz, Iran; eeey Department of Environmental Health Engineering, Gonabad University of Medical Sciences, Gonabad, Iran; eeez Department of Bioengineering and Therapeutical Sciences, University of California San Francisco, San Francisco, CA, USA; fffa Department of Administration, PGxAI, San Francisco, CA, USA; fffb Mohammed VI International School of Public Health, Mohammed VI University of Sciences and Health (UM6SS), Casablanca, Morocco; fffc Institute for Healthcare System Analysis (IA2S), Paris, France; fffd Department of Public Health, University of Hail, Hail, Saudi Arabia; fffe Department of Zoology and Entomology, Al-Azhar University, Cairo, Egypt; ffff Department of Psychiatry, Johns Hopkins University, Baltimore, MD, USA; fffg Columbia University Mailman School of Public Health, Columbia University, New York, NY, USA; fffh Faculdade de Medicina da Universidade de Sao Paulo, University of Sao Paulo, Sao Paulo, Brazil; fffi School of Pharmacy, Queen’s University Belfast, Belfast, UK; fffj School of Pharmacy, China Medical University, Shenyang, China; fffk School of global health, Shanghai Jiao Tong University, Shanghai, China; fffl National Institute of Parasitic Diseases, Chinese Center for Disease Control and Prevention, Shanghai, China; fffm Harvard Medical School, Harvard University, Boston, MA, USA; fffn Department of Surgery, Zhejiang University, yiwu, Zhejiang province,China, China; fffo School of Public Health and Emergency Management, Southern University of Science and Technology, Shenzhen, China; fffp College of Nursing, Prince Sattam bin Abdulaziz University, Al-Kharj, Saudi Arabia; fffq Faculty of Nursing, Mansoura University, Mansoura, Egypt; fffr Center for Clinical Microbiology, University College London, London, England; fffs NIHR-Biomedical Research Centre (NIHR-BRC), University College London Hospitals, London, England; ffft Department of Chemistry, An-Najah National University, Nablus, Palestine; fffu Clinical Research Centre, An-Najah National University Hospital, Nablus, Palestine; fffv Department of Building Engineering and Environment, Palestine Technical University (Kadoorie), Tulkarem, Palestine; fffw Civil Engineering and Sustainable Structures, Palestine Technical University (Kadoorie), Tulkarem, Palestine; fffx World Bank; fffy School of Medicine, National Autonomous University of Mexico, Mexico City, Mexico; fffz Department of Global Health and Population, T.H. Chan School of Public Health, Boston, MA, USA

## Abstract

**Background:**

Understanding the size, gender composition, and cadre mix of the health workforce and how it has evolved across time and locations can inform policies for planning, recruitment, training, and retention of human resources for health (HRH), and improvement of access to the health-care workforce among populations. Using comparable and standardised data sources, we aim to describe the composition and density of health workers by sex among 20 cadres for 204 countries and territories over 1990–2023 and quantify workforce shortfalls relative to universal health coverage (UHC) attainment.

**Methods:**

We used 1816 country-years of data from population-based surveys, 82 from censuses, 3888 from administrative sources, and 96 from scientific literature. We harmonised reported occupation in all sources with the International Standard Classification of Occupations 2008. We produced estimates for 20 cadres and the total health workforce disaggregated by sex using spatiotemporal Gaussian process regression, a standardised method in the Global Burden of Diseases, Injuries, and Risk Factors study. Finally, stochastic frontier meta-regression was used to estimate the minimum density of doctors, nurses and midwives, dentists, and pharmacists required to reach a score of 80 out of 100 on the UHC effective coverage index.

**Findings:**

In 2023, there were 122·1 million (95% uncertainty interval 115·4–130·5) health workers across 20 cadres, an increase of 81·2 million (72·0–90·5) or 198·5% (161·9–245·6) relative to 1990. Of that total, there were 15·1 million (13·1–18·2) doctors, 33·2 million (30·8–36·3) nurses, 2·4 million (2·0–2·9) midwives, and 7·6 million (6·7–8·6) community health workers (CHWs). 68·9% (66·9–70·6) of all health workers in 2023 were female, and female health workers accounted for 71·4% (64·5–77·4) of net HRH workforce growth. Less than half of doctors were female (43·9%, 34·5–53·2) and most nurses (80·7%, 76·2–83·1), midwives (96·0%, 83·9–98·5), and CHWs (89·5%, 83·6–93·7) were female. Substantial differences in HRH density remained into 2023: sub-Saharan Africa had the lowest HRH densities across cadres, with the exception of CHWs, while high-income countries had the highest HRH densities. To reach 80 out of 100 on the UHC index, an additional 7·1 million (6·6–7·5) doctors, 23·9 million (21·8–26·1) nurses and midwives, 1·8 million (1·6–1·9) dentists, and 1·6 million (1·4–1·9) pharmacists are required globally.

**Interpretation:**

The global health workforce has expanded substantially since 1990, largely due to women entering the formal health workforce. However, this expansion has been uneven across regions and persistent shortfalls remain in doctors, nurses and midwives, dentists, and pharmacists relative to moderate UHC attainment. Addressing these gaps will require expansion of training capacity, retention and remuneration policies for early-career workers, and gender-responsive workforce arrangements.

**Funding:**

Gates Foundation.

## Introduction

A sufficient supply of human resources for health (HRH) is fundamental to the functioning of health systems.^1^ Addressing shortages in health workforce size and shortcomings in HRH performance are imperative for preparing for health challenges around the world. Comparable and comprehensive information on HRH cadres, or the groups of specially trained personnel active in the health workforce, enables comparison across countries that can shed light on the unique organisational and labour force arrangements underpinning health-care delivery.^1^, ^2^

**Research in context**

**Evidence before this study**

There is a paucity of comparable sex-disaggregated estimates of the health workforce by cadre across time and country, limiting both diagnosis of workforce gaps and the design of equitable workforce policies. Global estimates of doctors, nurses, and other human resources for health (HRH) cadres have been published for more than 100 countries in non-peer-reviewed reports by WHO and the International Labour Organization (ILO) as well as in peer-reviewed publications by the Global Burden of Diseases, Injuries, and Risk Factors Study (GBD). WHO–ILO reports from 2022, 2019, and previous years provided estimates of the sex composition of doctors, nurses, and other HRH, as well as health and social sector workers, an aggregate that includes workers outside of the health sector. WHO–ILO-estimated sex shares are limited in not comprehensively presenting trends over time for all locations and cadres, omitting some countries, numerous years, and many types of workers. A PubMed title and abstract search from inception to June 25, 2026, using the terms: “global” AND (“health workforce” OR “health professional” OR “health provider” OR “health personnel” OR “human resources for health”) AND (“sex” OR “gender”), identified just two additional peer-reviewed, multicountry studies on HRH sex composition, both covering 25 or fewer, primarily high-income, countries. No existing evidence provides comparable, sex-specific estimates for more than four cadres and total HRH across all countries over 1990–2023. This precludes full understanding of how the availability, cadre mix, and gender composition of HRH influence the delivery of health services and are shaped by health system design in different contexts.

**Added value of this study**

This study provides the first global, sex-disaggregated estimates of 20 health worker cadres across 204 countries and territories from 1990 to 2023, including the first global quantification of community health workers (CHWs) and offering the most comprehensive assessment of the global health workforce to date. By harmonising survey, census, and administrative sources using International Standard Classification of Occupations 2008 classifications and model-based adjustments, it overcomes limitations in existing HRH data systems. Relative to GBD 2019 estimates of HRH, this study uses an additional 1459 country-years of data. These updated estimates were analysed with an improved stochastic frontier meta-analysis approach, a frontier estimation methodology used to evaluate the minimum HRH density observed in countries attaining a given level of the universal health coverage (UHC) effective coverage index for the cadres identified in Sustainable Development Goal indicator 3.c.1. The use of minimum thresholds recognises that performance can be attained with different cadre mixes while also offering guidance on minimum health workforce needs. These innovations produce new findings that were not possible with previously available estimates, including the quantification of sex-specific HRH growth, information on the global feminisation of health cadres, and new cadre-specific thresholds for UHC. Overall, these findings provide new insights into gender dynamics, cadre composition, and global workforce shortages, with direct relevance for national and international health workforce planning.

**Implications of all the available evidence**

Comparable information on the size, cadre mix, and gender composition of HRH is essential in workforce planning for building a resilient health workforce to attain UHC and prepare for future health threats. Since 1990, there has been extensive growth in human resources for health, driven primarily by women formally joining the health workforce. Women accounted for almost three-quarters of health workforce growth and made up more than two-thirds of the health workforce in 2023. Despite these large increases in formal health workforce participation, women continue to be less than 50% of doctors and are more likely to be working as nurses, midwives, CHWs, and other health workers, who tend to have fewer opportunities to occupy leadership positions. These circumstances lead to structural imbalances in the gender composition of health system leadership versus the health workforce. Evidence consistently emphasises that millions more health workers are needed globally to attain just moderate levels of UHC.

Describing the distribution of sex within each health worker cadre is also important. Sex-disaggregated data and analysis are critical to ensuring that women's role in health systems informs health workforce assessment and planning.^3^ Female health workers might leave the workforce or not enter in the first place if there are not policies that, for example, ensure fair remuneration levels, eliminate violence and harassment, address leadership disparities, and accommodate caregiving responsibilities.^3^, ^4^ Without accounting for sex composition along with cadre mix, health systems could suffer from lower HRH productivity and the loss of talent and human capital.^5^, ^6^

Existing research suggests the health workforce exhibits high levels of occupational segregation by sex, with some cadres, such as doctors, undergoing increasing so-called feminisation, or the movement of women into historically male-dominated occupations, over time.^7^ Most of this research, however, focuses on single countries or high-income countries, with a much more limited evidence base describing health workers by sex and cadre for low-income and middle-income countries. Existing peer-reviewed studies have examined the sex composition of HRH over time in 25 or fewer countries only, with most data sourced from high-income settings.^8^, ^9^ Global reports from the International Labour Organization (ILO) and WHO have used data from 100 or more countries to estimate the sex composition of HRH cadres.^1^, ^4^, ^10^, ^11^, ^12^ However, these reports did not present trends over time by individual location, cadre, and sex composition simultaneously; these reports also did not provide sex-composition estimates of key HRH cadres, such as community health workers (CHWs), pharmacists and pharmaceutical assistants, dentists and dental assistants, and total HRH. Although existing research has made advances in measuring the densities of HRH globally,^13^ gaps persist in information about the sex composition of specific HRH cadres for all countries and how that has changed over the past 30 years with new policies and socioeconomic development. Overall, comparable sex-disaggregated estimates of the health workforce by cadre across time and country have not been available to date.

The present study uses comparable and standardised data sources and standard methodology from the Global Burden of Diseases, Injuries, and Risk Factors Study (GBD) to assess the composition and density of health workers by sex, with a focus on 20 HRH cadres in 204 countries and territories from 1990 to 2023.^14^ We also assess the minimum densities associated with attaining a moderate UHC effective coverage index score, focusing on the cadres emphasised in Sustainable Development Goal (SDG) target 3.c.1. With these estimates, decision makers can assess how the size, sex, and cadre composition of their health workforce has evolved over the past 30 years as well as appreciate the current magnitude of HRH shortfalls, information essential for workforce planning and policy reform.

## Methods

### Overview

We estimated 20 HRH cadres for 204 countries and territories for the period of 1990 to 2023 for GBD 2023. Relative to previous GBD 2019 estimates, this study estimates an expanded set of HRH cadres, including nurses, midwives, and CHWs separately for the first time. This study also disaggregates all cadres by sex for the first time. We increased the number of country-years of data used by 1459 relative to GBD 2019 estimates and harmonised all data sources with the most up-to-date occupation coding system, International Standard Classification of Occupations 2008 (ISCO-08). Compliance with the Guidelines for Accurate and Transparent Health Estimates Reporting (GATHER) is described in appendix 1 (pp 5–6).

Health workers can be described in terms of sex (biological categorisations of people as males, females, or intersex) or by their gender identity (a person's internal sense of the self that is shaped by socially constructed roles and norms tied to sex assigned at birth).^15^ Nearly all of our data identified individuals by a binary definition of sex. We therefore used sex rather than a broader definition of gender identity in our description of health workers.

ISCO-08 categories were used to classify occupations in 73·3% of data and enabled us to estimate nurses separately from midwives and estimate CHW densities. The exact ISCO-08 codes used for each health worker cadre are shown in appendix 1 (pp 7–9). We mapped data sources that used former ISCO coding systems to ISCO-08 using concordance documentation provided by the ILO.^16^ Country-specific coding systems (6·1% of sources) were used only when the matches for HRH cadres were strictly unambiguous.

We used the UHC effective coverage index produced as part of GBD 2023 to estimate minimum HRH density thresholds.^17^ The UHC effective coverage index is composed of 23 effective coverage indicators representing a range of health service types. Indicators were weighted based on the potential disability-adjusted life-years gained from full coverage.

### Data sources

We used population-based surveys, censuses, administrative data, and scientific literature. Surveys and censuses captured self-reported employment status and current occupation. In population-based sources, health workers were counted if they were working for pay or if respondents were on temporary leave or vacation, consistent with globally recognised definitions of employment.^18^ In administrative sources and some scientific literature, we were unable to verify employment status and receipt of pay systematically, but these sources are understood to capture the employed workforce as well. 82 country-years of census data and 1816 country-years of survey data were used. Administrative records were sourced primarily from WHO's National Health Workforce Accounts database^19^ and the Eurostat Health Database,^20^ amounting to 3888 country-years (appendix 1 pp 9–22). Scientific literature sources (96 country-years) were used primarily to augment CHW data because information on this cadre is available in ISCO-08 but not previous ISCO classifications.

We estimated the count per 10 000 population (or density) for 20 distinct health worker cadres. When converting the denominator from the working-age population to the total population, which was necessary for some labour force surveys, or when working with counts that were converted into densities, we used GBD 2023 estimates of population.^21^ In the main text, we focused on results for the largest cadres: doctors; nurses; midwives; dentists and dental assistants; pharmacists and pharmaceutical assistants; and CHWs, along with total HRH. We grouped all other HRH cadres into the other HRH category in the main text, with estimates for the remaining cadres in appendix 2, consisting of: clinical officers and medical assistants; physiotherapists and prosthetic technicians; medical imaging and therapeutic equipment technicians; medical laboratory technicians; health-care aides and ambulance workers; environmental health workers; optometrists and opticians; dieticians and nutritionists; audiologists, speech therapists, and counsellors; psychologists; personal care workers; and traditional and complementary practitioners (appendix 1 p 9).

Administrative data have potential biases that we addressed through cross-walking to survey and census data.^13^ We note that biases in the administrative data have decreased over time, with improvements to data collection and assessment, and thus we updated the adjustment methodology that was deployed previously to produce GBD 2019 estimates. The methodology is described in detail in appendix 1 (pp 29–39).^13^ We did not adjust sex shares because we did not find evidence of systematic source-type differences in sex shares (appendix 1 p 32).

### Modelling

We used spatiotemporal Gaussian process regression (ST-GPR) to estimate densities and sex shares for each of the 20 cadres and total HRH. ST-GPR is a three-stage estimation tool that borrows information across location and time to produce estimates with uncertainty.^14^, ^21^ First, a linear mixed effects regression with random effects on location was fit using the Socio-demographic Index (SDI), a composite index measuring development status,^22^ along with total health expenditure per capita, and the proportion of the employed population aged 15–69 years working as professionals. The female share of the employed population and the proportion of females aged 15–69 years working as professionals were used in addition to SDI for the cadre-specific sex shares. Second, a polynomial regression function (LOESS) was fit to smooth the residuals from the first-stage model. Third, the smoothed estimates were used as the mean in a Gaussian process regression, which accounts for input data variance and model uncertainty to produce 250 draws for every location-year(-sex) group. Point estimates for all reported quantities were calculated from the mean across draws and 95% uncertainty intervals (UIs) were computed from the 2·5th and 97·5th percentiles of draws. More details on all modelling steps are provided in appendix 1 (pp 40–46).

We produced estimates of the minimum densities of the four cadres specified in SDG target 3.c.1 (doctors, nurses and midwives, dentists, and pharmacists) required to achieve moderate (80) to high (90) levels on the UHC effective coverage index, updating previous estimates from GBD 2019. Few, mostly high-income countries have achieved a UHC score of 90 or higher historically, and thus we focused on a UHC threshold of 80 as a more globally representative and moderate target.^13^ Minimum density was determined based on the lowest levels of each HRH density along a production frontier that corresponded with the UHC thresholds (80 or 90). The production frontier was estimated using non-parametric stochastic frontier meta-analysis,^23^ which fits a flexible spline to estimate the functional form of the relationship and was constrained to be monotonically increasing and concave. The production frontier represents the maximum UHC level expected based on current densities of HRH, with higher levels of UHC (or a smaller distance to the frontier) for a given HRH density representing higher levels of efficiency. Because we estimate each minimum cadre threshold separately, we do not consider the distinct skill mix required to attain UHC, only the minimum health worker densities required to attain a given UHC score. Additional details about estimating the frontier and shortages are reported in appendix 1 (pp 46–49).

Analyses and figures were generated with R (version 4.4.2), Python (version 3.12.3), or Stata (version 17.0).

### Role of the funding source

The funder of the study had no role in study design, data collection, data analysis, data interpretation, or writing of the report.

## Results

**Figure 1:**
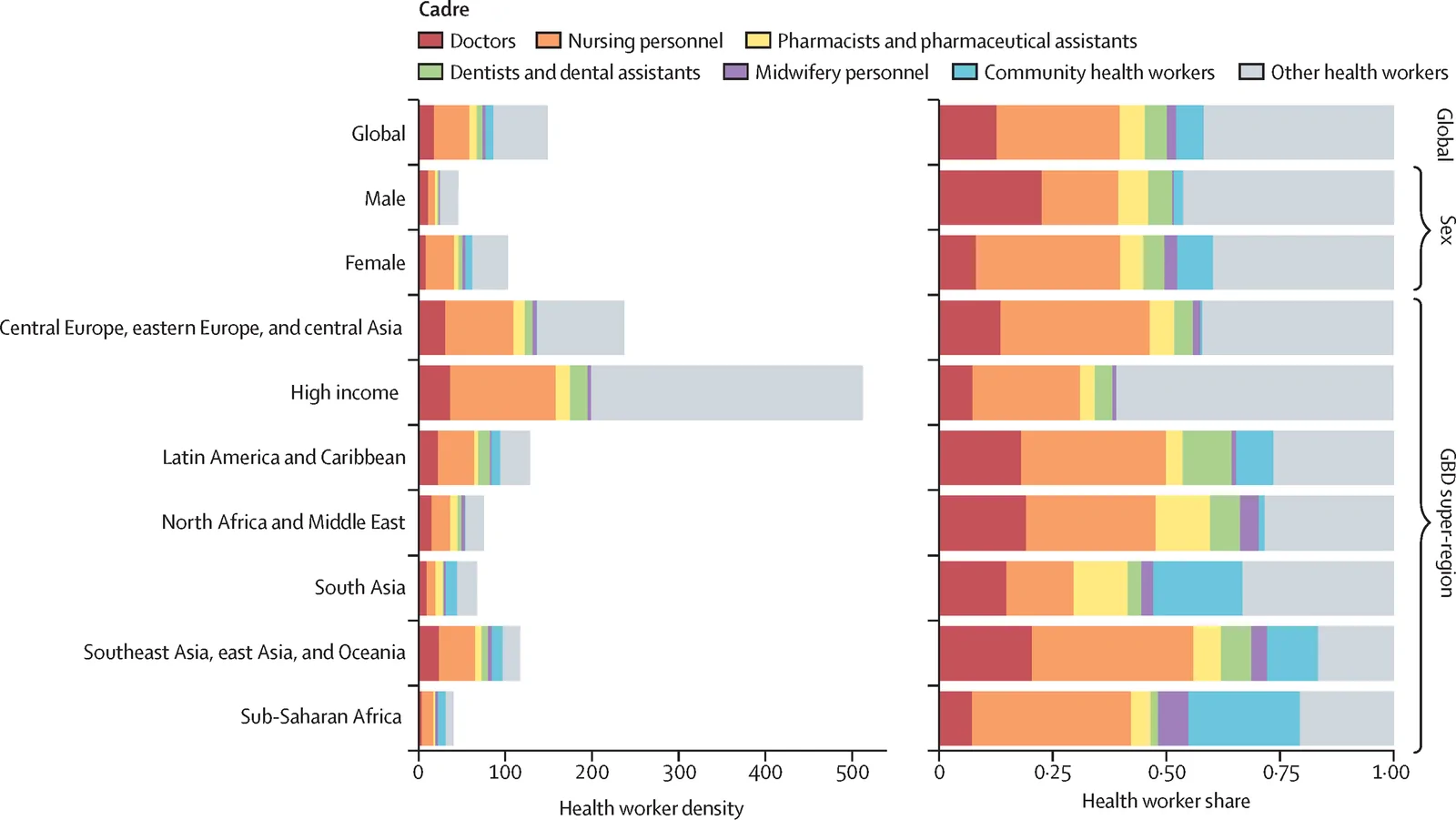
Composition of the health workforce by cadre, sex, and GBD super-region, 2023 Density is reported in count of workers per 10 000 population. GBD=Global Burden of Diseases, Injuries, and Risk Factors Study.

Globally, 122·1 million (95% UI 115·4–130·5) health workers were actively working in health systems in 2023. There were 15·1 million (13·1–18·2) doctors, 33·2 million (30·8–36·3) nurses, 6·8 million (5·8–8·6) pharmacists and pharmaceutical assistants, 6·1 million (5·0–7·9) dentists and dental assistants, 2·4 million (2·0–2·9) midwifery personnel, and 7·6 million (6·7–8·6) CHWs (appendix 2 p 5). In table 1 and figure 1, we present the largest cadres across the health workforce: doctors, nurses, midwives, CHWs, pharmacists and pharmaceutical assistants, and dentists and dental assistants. We group all remaining 12 cadres into “other HRH” for visualisation; estimates are provided in appendix 2 (pp 32–127). Globally, nurses comprised the largest share of the total health workforce, amounting to 27·2% (25·1–29·5) in 2023 (figure 1), with densities of 41·2 (38·2–45·1) per 10 000 population. Doctors were the next largest cadre of health workers with densities of 18·8 (16·3–22·5), comprising 12·4% (10·9–14·7) of health workers in 2023.

**Table 1:** Estimated density of health workers for all GBD super-regions, regions, and 204 countries and territories in 2023

		**All health workers**	**Doctors**	**Nursing personnel**	**Pharmacists and pharmaceutical assistants**	**Dentists and dental assistants**	**Midwifery personnel**	**Community health workers**	**Other health workers**
**Global**		**151·4 (143·1–161·8)**	**18·8 (16·3–22·5)**	**41·2 (38·2–45·1)**	**8·4 (7·2–10·7)**	**7·5 (6·2–9·8)**	**2·9 (2·5–3·6)**	**9·4 (8·3–10·7)**	**63·2 (56·0–71·1)**
High SDI		415·5 (389·0–447·4)	33·7 (32·3–36·2)	100·9 (94·8–108·4)	17·7 (16·3–19·8)	17·4 (16·1–20·7)	3·8 (3·4–4·6)	0·4 (0·2–0·8)	241·7 (214·3–269·7)
High-middle SDI		208·5 (194·5–223·7)	26·6 (22·6–32·6)	55·8 (50·8–62·5)	9·5 (8·4–11·1)	11·2 (8·7–16·1)	3·4 (3·1–4·0)	24·5 (19·7–31·5)	77·5 (67·8–87·7)
Middle SDI		116·4 (106·7–127·8)	23·7 (18·4–30·6)	43·2 (37·9–49·8)	6·7 (5·1–9·2)	8·8 (5·5–13·9)	3·3 (2·4–4·4)	9·3 (7·8–10·8)	21·4 (18·6–24·2)
Low-middle SDI		64·7 (58·0–73·2)	9·1 (6·4–13·9)	12·2 (9·1–16·9)	6·8 (4·5–10·4)	2·3 (1·6–3·5)	2·2 (1·4–3·5)	11·8 (9·5–14·9)	20·2 (17·6–23·0)
Low SDI		37·6 (34·8–41·2)	3·8 (2·9–5·8)	9·9 (7·7–12·5)	2·9 (2·3–4·3)	0·7 (0·6–0·8)	2·2 (2·0–2·5)	9·9 (9·1–10·8)	8·2 (7·1–9·3)
**Central Europe, eastern Europe, and central Asia**		**238·3 (224·3–255·8)**	**31·6 (28·3–36·1)**	**78·5 (72·2–86·8)**	**13·0 (11·3–15·4)**	**10·0 (9·0–12·0)**	**3·7 (3·2–4·4)**	**1·3 (0·9–1·7)**	**100·3 (87·9–113·5)**
Central Asia		142·7 (136·3–151·8)	29·9 (28·1–32·2)	76·6 (72·0–84·0)	5·7 (4·1–8·8)	4·6 (3·7–6·6)	3·4 (2·5–4·6)	0·2 (0·1–0·5)	22·2 (19·4–25·2)
	Armenia	136·1 (122·9–151·2)	33·0 (26·4–40·5)	41·9 (32·4–53·1)	13·9 (7·9–22·8)	5·3 (3·5–8·2)	3·3 (2·4–4·3)	0·2 (0·1–0·4)	38·3 (33·4–43·5)
	Azerbaijan	132·9 (119·2–150·6)	32·6 (26·6–39·9)	55·9 (44·2–71·0)	6·0 (3·1–10·9)	6·4 (3·8–11·8)	3·9 (2·4–5·8)	0·2 (0·1–0·4)	27·9 (24·3–31·6)
	Georgia	189·8 (176·4–204·7)	49·9 (42·3–58·8)	74·8 (66·5–82·9)	19·6 (14·1–25·8)	16·7 (13·9–20·3)	1·0 (0·5–1·8)	0·0 (0·0–0·0)	27·8 (24·2–31·5)
	Kazakhstan	160·3 (145·4–174·4)	38·6 (33·9–45·0)	64·0 (52·3–76·1)	7·3 (4·4–12·0)	5·0 (2·6–8·4)	4·2 (3·1–5·3)	0·2 (0·1–0·4)	41·0 (35·8–46·5)
	Kyrgyzstan	79·4 (70·2–89·8)	19·1 (14·8–23·7)	38·2 (29·3–47·7)	2·3 (1·1–4·5)	4·1 (2·2–6·8)	2·2 (1·4–3·2)	0·2 (0·1–0·5)	13·4 (11·7–15·2)
	Mongolia	132·3 (115·7–149·6)	42·2 (34·5–51·6)	53·0 (41·2–65·8)	12·8 (9·4–16·5)	3·9 (2·6–5·6)	6·6 (4·5–9·7)	1·0 (0·5–1·6)	12·7 (11·1–14·4)
	Tajikistan	99·5 (89·4–110·0)	19·7 (15·2–24·1)	65·7 (56·9–74·8)	4·1 (2·1–8·0)	5·3 (2·5–9·9)	1·3 (0·9–1·9)	0·2 (0·1–0·6)	3·1 (2·7–3·5)
	Turkmenistan	102·4 (90·5–116·1)	25·9 (21·2–31·5)	44·4 (33·8–56·6)	4·6 (2·7–8·0)	3·1 (1·6–5·5)	2·3 (1·2–3·6)	0·2 (0·1–0·4)	21·9 (19·1–24·8)
	Uzbekistan	163·7 (139·0–194·2)	26·1 (19·2–33·5)	111·6 (89·2–141·0)	2·9 (1·2–6·1)	2·6 (1·1–4·9)	4·0 (2·5–5·8)	0·2 (0·1–0·5)	16·4 (14·3–18·5)
Central Europe		250·0 (234·7–264·9)	32·8 (31·4–34·7)	66·8 (62·9–71·6)	19·8 (17·0–24·4)	13·9 (10·6–19·4)	2·9 (2·8–3·1)	1·1 (0·7–1·6)	112·7 (98·5–127·5)
	Albania	153·8 (132·8–179·7)	30·7 (20·8–41·1)	61·5 (43·8–83·0)	13·4 (6·7–24·8)	15·1 (8·2–24·0)	3·9 (2·4–6·2)	0·3 (0·1–0·8)	28·8 (25·1–32·6)
	Bosnia and Herzegovina	130·5 (118·1–146·2)	19·8 (12·5–30·9)	68·7 (59·1–78·6)	5·7 (2·8–10·7)	4·5 (2·4–7·8)	3·2 (2·1–4·9)	0·3 (0·1–0·8)	28·2 (24·6–32·0)
	Bulgaria	263·9 (244·4–286·2)	43·5 (37·2–52·5)	42·8 (34·1–52·6)	13·9 (10·0–19·2)	13·3 (10·4–16·5)	5·0 (3·6–6·7)	0·3 (0·1–0·8)	145·1 (127·4–164·0)
	Croatia	263·4 (241·6–285·0)	32·4 (25·8–39·1)	78·7 (67·5–93·5)	19·2 (13·8–25·5)	17·2 (12·5–22·3)	4·1 (3·0–5·6)	0·0 (0·0–0·0)	111·9 (97·8–126·7)
	Czechia	303·2 (277·2–328·8)	41·6 (34·2–51·3)	91·8 (78·1–108·0)	16·4 (12·1–22·1)	13·8 (9·6–19·2)	4·3 (2·9–6·5)	0·0 (0·0–0·0)	135·3 (118·4–153·1)
	Hungary	282·8 (256·6–307·3)	19·7 (14·2–25·6)	53·7 (43·7–64·7)	19·0 (14·5–25·1)	13·1 (9·4–17·7)	2·5 (1·8–3·4)	4·3 (2·7–6·1)	170·4 (149·0–192·7)
	Montenegro	152·0 (139·7–166·2)	19·1 (15·1–24·2)	54·7 (48·3–62·3)	6·6 (4·9–9·4)	0·9 (0·6–1·5)	4·0 (3·4–4·6)	0·3 (0·1–0·7)	66·4 (57·9–75·3)
	North Macedonia	164·8 (150·2–180·1)	32·2 (24·8–39·5)	52·9 (43·7–64·3)	14·4 (10·9–18·7)	11·8 (8·8–16·7)	4·3 (3·3–5·8)	0·3 (0·1–0·7)	48·8 (42·6–55·4)
	Poland	274·7 (250·7–298·5)	38·6 (33·2–46·0)	57·5 (45·8–70·7)	26·2 (19·2–37·7)	14·5 (6·5–26·0)	2·5 (1·6–3·6)	0·1 (0·1–0·3)	135·3 (118·3–153·1)
	Romania	220·2 (204·7–235·4)	26·4 (20·3–32·7)	83·1 (73·0–92·8)	22·0 (17·9–26·2)	17·0 (12·9–23·9)	1·7 (1·1–2·6)	3·2 (1·4–5·9)	66·9 (58·4–75·8)
	Serbia	132·4 (119·4–147·0)	24·2 (18·3–31·1)	50·3 (42·1–60·2)	9·6 (6·0–13·6)	9·9 (5·0–18·8)	2·9 (1·6–4·8)	1·0 (0·5–1·9)	34·6 (30·1–39·2)
	Slovakia	296·8 (273·2–322·2)	30·2 (23·7–38·4)	84·4 (71·5–99·7)	13·6 (10·5–17·2)	10·8 (8·1–14·6)	3·6 (2·5–5·1)	0·0 (0·0–0·1)	154·2 (135·7–174·2)
	Slovenia	335·3 (308·8–364·5)	35·5 (27·0–44·2)	116·5 (102·4–132·1)	18·2 (12·1–25·2)	18·5 (10·9–30·4)	2·3 (1·5–3·5)	0·0 (0·0–0·0)	144·2 (126·2–163·2)
Eastern Europe		278·6 (256·9–305·0)	31·8 (25·4–39·9)	85·7 (74·2–101·4)	12·8 (10·7–16·4)	10·5 (8·7–12·6)	4·2 (3·2–5·6)	1·9 (1·3–2·5)	131·7 (115·6–148·9)
	Belarus	269·6 (251·3–288·1)	46·1 (39·7–53·5)	102·1 (90·6–114·7)	23·5 (18·8–28·1)	20·5 (15·1–27·5)	5·3 (3·8–6·8)	1·8 (0·8–4·4)	70·4 (61·4–79·8)
	Estonia	293·6 (266·9–319·3)	32·6 (26·1–41·1)	67·1 (54·7–79·7)	13·1 (9·8–16·9)	21·7 (16·7–29·3)	3·9 (2·9–5·1)	0·0 (0·0–0·0)	155·1 (135·9–175·4)
	Latvia	285·1 (258·3–312·5)	31·4 (24·8–38·2)	41·1 (29·6–53·4)	16·4 (12·4–21·0)	15·9 (11·4–24·0)	2·1 (1·4–3·1)	0·1 (0·0–0·1)	178·1 (156·3–201·1)
	Lithuania	310·2 (283·5–335·3)	40·9 (33·6–50·2)	62·6 (52·3–78·0)	16·5 (12·2–21·6)	22·8 (15·2–32·6)	3·4 (2·5–4·5)	0·4 (0·2–0·8)	163·5 (143·8–184·8)
	Moldova	163·9 (148·0–179·3)	35·4 (30·2–41·1)	52·8 (41·1–66·2)	8·7 (4·3–15·6)	12·0 (9·0–16·8)	1·7 (0·7–3·3)	0·7 (0·3–1·7)	52·7 (45·9–59·7)
	Russia	299·4 (270·2–330·1)	28·3 (19·5–37·6)	92·4 (76·8–111·8)	12·1 (7·2–19·0)	8·9 (6·2–13·5)	4·2 (3·0–6·2)	2·0 (1·0–3·2)	151·4 (133·2–171·2)
	Ukraine	207·9 (173·9–260·9)	40·6 (15·6–93·6)	63·4 (50·9–78·3)	12·7 (5·6–30·4)	12·4 (5·6–22·1)	4·3 (1·8–9·2)	1·7 (0·8–4·1)	72·7 (63·3–82·4)
**High income**		**513·6 (477·8–555·7)**	**36·5 (34·1–39·4)**	**121·8 (111·9–134·2)**	**16·8 (15·5–18·4)**	**20·6 (18·3–25·5)**	**4·0 (3·4–5·1)**	**0·1 (0·1–0·3)**	**313·8 (278·9–349·5)**
Australasia		665·7 (611·8–726·1)	40·4 (34·9–46·3)	128·4 (112·2–147·3)	21·1 (15·2–30·4)	10·2 (6·9–16·2)	7·8 (6·4–9·3)	3·2 (1·5–7·4)	454·7 (404·1–506·2)
	Australia	682·0 (625·9–745·2)	41·0 (32·2–49·6)	129·1 (112·0–148·9)	22·1 (14·7–33·9)	9·1 (6·5–13·8)	7·6 (5·9–9·3)	3·3 (1·5–7·9)	469·8 (417·6–522·9)
	New Zealand	585·3 (539·5–632·6)	37·5 (29·4–49·4)	125·0 (111·8–140·0)	15·8 (10·1–24·0)	15·3 (8·6–28·4)	8·6 (6·6–10·7)	2·6 (1·2–5·6)	380·5 (337·7–424·3)
High-income Asia Pacific		360·5 (335·5–387·5)	23·5 (18·9–29·9)	122·1 (115·7–129·6)	25·9 (20·8–31·7)	23·3 (15·8–38·2)	1·3 (1·0–1·6)	0·0 (0·0–0·0)	164·4 (144·8–185·3)
	Brunei	225·4 (201·6–250·7)	19·6 (12·8–28·8)	58·5 (44·6–75·8)	6·6 (3·3–13·2)	7·0 (3·6–12·2)	8·5 (6·6–10·4)	0·0 (0·0–0·0)	125·3 (109·6–141·7)
	Japan	404·6 (369·6–442·0)	24·3 (17·1–34·7)	117·3 (101·1–133·8)	30·3 (23·3–39·1)	23·5 (15·0–40·2)	1·2 (0·8–1·6)	0·0 (0·0–0·0)	208·0 (183·3–234·4)
	South Korea	250·5 (222·3–280·6)	21·1 (15·5–28·5)	140·8 (120·1–166·4)	16·9 (11·0–26·8)	25·1 (17·3–36·9)	1·5 (1·0–2·1)	0·0 (0·0–0·0)	45·1 (39·3–51·1)
	Singapore	403·3 (368·2–444·6)	28·5 (22·3–35·8)	64·7 (55·7–75·4)	14·1 (8·7–21·8)	5·7 (3·8–7·7)	0·2 (0·1–0·5)	0·0 (0·0–0·0)	290·1 (256·5–325·4)
High-income North America		669·4 (612·3–732·6)	29·7 (25·9–34·6)	156·2 (133·9–184·8)	13·2 (10·9–15·3)	20·5 (17·8–24·5)	2·3 (2·0–2·6)	0·1 (0·0–0·1)	447·4 (399·6–497·0)
	Canada	597·8 (551·7–647·5)	27·6 (21·1–35·2)	110·6 (96·8–126·9)	15·5 (11·5–19·9)	18·2 (11·1–30·9)	0·5 (0·3–0·9)	0·1 (0·0–0·1)	425·4 (384·0–470·0)
	Greenland	377·8 (328·1–451·3)	22·7 (11·9–43·1)	87·6 (48·2–144·7)	13·8 (3·0–54·2)	22·3 (11·1–39·2)	4·6 (2·0–9·4)	0·1 (0·1–0·3)	226·7 (200·3–255·8)
	USA	677·7 (618·0–743·0)	29·9 (25·8–35·6)	161·4 (136·7–192·7)	12·9 (10·5–15·2)	20·8 (17·8–24·6)	2·5 (2·2–2·8)	0·1 (0·0–0·1)	450·0 (401·4–500·1)
Southern Latin America		213·9 (200·9–229·3)	44·8 (40·7–49·3)	58·5 (54·0–63·2)	7·7 (5·5–10·8)	16·8 (13·4–24·3)	3·5 (3·3–3·9)	0·1 (0·1–0·3)	82·4 (72·0–93·4)
	Argentina	190·3 (174·6–210·2)	50·7 (44·7–56·9)	45·7 (37·0–54·8)	9·2 (6·4–13·3)	16·1 (10·3–27·4)	1·3 (1·1–1·7)	0·2 (0·1–0·5)	67·2 (58·6–76·2)
	Chile	256·4 (237·0–277·7)	28·8 (23·0–34·4)	86·6 (76·5–98·4)	4·6 (3·0–7·4)	19·7 (14·6–25·7)	9·6 (8·3–11·0)	0·0 (0·0–0·0)	107·2 (93·6–121·4)
	Uruguay	305·1 (279·3–333·0)	51·2 (40·4–64·5)	80·4 (68·9–94·0)	4·2 (2·7–6·3)	11·9 (7·6–17·4)	1·1 (0·7–1·4)	0·0 (0·0–0·1)	156·3 (137·0–176·7)
Western Europe		475·6 (443·1–511·1)	47·7 (44·9–51·8)	98·4 (87·5–113·8)	17·2 (16·2–19·0)	21·0 (18·4–26·3)	6·7 (4·9–10·1)	0·1 (0·0–0·1)	284·5 (252·5–317·3)
	Andorra	467·5 (431·2–511·0)	44·2 (38·2–50·9)	42·9 (36·8–50·7)	13·6 (10·0–18·9)	23·5 (14·6–43·5)	2·2 (1·7–2·7)	0·0 (0·0–0·0)	341·2 (304·1–379·5)
	Austria	419·9 (379·0–463·0)	50·9 (45·0–58·2)	77·8 (54·6–112·1)	19·8 (15·0–26·0)	22·5 (14·0–35·6)	3·1 (2·6–3·7)	0·0 (0·0–0·1)	245·7 (217·2–276·6)
	Belgium	490·9 (442·8–541·6)	38·6 (32·0–47·7)	171·7 (141·7–211·4)	17·7 (13·4–23·2)	14·5 (10·3–19·7)	7·6 (5·3–10·8)	0·0 (0·0–0·0)	240·8 (213·3–271·4)
	Cyprus	243·1 (220·7–268·1)	44·9 (36·9–52·8)	36·4 (25·9–48·4)	16·9 (8·4–32·7)	15·2 (10·3–21·3)	8·7 (4·1–16·7)	0·4 (0·2–0·9)	120·6 (105·4–136·5)
	Denmark	798·1 (731·9–859·5)	55·0 (45·3–68·0)	113·9 (93·3–134·9)	17·7 (12·3–24·6)	19·6 (13·8–27·9)	7·1 (4·3–11·3)	0·0 (0·0–0·0)	584·8 (525·5–644·7)
	Finland	646·0 (587·8–710·2)	32·6 (22·1–43·7)	145·3 (113·2–186·8)	20·8 (14·4–29·5)	20·0 (13·7–28·3)	5·2 (2·8–9·2)	0·0 (0·0–0·0)	422·0 (377·6–467·5)
	France	367·6 (336·0–397·2)	33·7 (28·8–39·3)	81·6 (67·4–95·8)	37·2 (31·0–45·1)	12·8 (9·9–16·3)	4·3 (3·8–4·7)	0·2 (0·1–0·4)	197·8 (172·5–224·4)
	Germany	533·5 (484·6–584·1)	52·1 (43·5–59·6)	130·7 (108·5–159·7)	17·8 (13·5–23·7)	23·8 (16·1–35·0)	11·7 (5·4–23·4)	0·0 (0·0–0·0)	297·4 (263·6–332·3)
	Greece	188·2 (171·9–207·1)	44·2 (36·8–54·3)	40·6 (31·8–52·4)	11·4 (8·5–15·1)	14·0 (10·5–19·0)	3·2 (2·7–3·7)	0·0 (0·0–0·0)	74·8 (65·3–84·7)
	Iceland	642·7 (595·4–694·6)	45·7 (39·4–53·7)	149·6 (133·5–163·7)	18·5 (14·5–23·3)	18·1 (14·0–22·5)	7·3 (6·7–8·0)	0·0 (0·0–0·0)	403·4 (358·8–448·9)
	Ireland	599·6 (555·8–644·9)	25·3 (20·3–31·4)	139·4 (126·4–154·4)	14·0 (11·3–16·9)	19·4 (11·4–31·6)	9·9 (8·6–11·4)	0·0 (0·0–0·0)	391·6 (346·3–438·1)
	Israel	395·0 (359·6–433·0)	34·7 (27·5–41·5)	57·1 (44·7–73·0)	11·7 (8·8–15·0)	22·6 (17·8–27·4)	2·6 (2·0–3·4)	0·3 (0·1–0·5)	266·2 (235·5–298·4)
	Italy	265·7 (242·5–290·3)	43·1 (35·9–52·2)	61·6 (50·7–78·7)	16·4 (12·3–20·4)	17·7 (12·2–26·4)	3·1 (2·5–3·8)	0·1 (0·0–0·2)	123·7 (108·4–139·9)
	Luxembourg	459·4 (414·8–507·5)	43·2 (25·0–64·5)	139·4 (119·8–164·3)	12·0 (6·1–23·5)	12·8 (7·6–20·9)	4·7 (2·3–9·1)	0·0 (0·0–0·0)	247·2 (218·1–279·2)
	Malta	400·5 (369·3–432·9)	42·2 (34·3–49·9)	78·9 (69·8–88·8)	21·1 (14·4–29·2)	10·4 (6·8–16·5)	6·0 (5·2–7·0)	0·0 (0·0–0·1)	241·9 (213·5–273·0)
	Monaco	642·7 (539·8–800·1)	80·5 (64·1–101·4)	213·6 (124·1–367·4)	29·4 (20·4–43·0)	23·9 (13·1–46·7)	6·7 (2·6–13·7)	0·0 (0·0–0·0)	288·7 (253·3–324·4)
	Netherlands	769·6 (712·1–827·1)	52·7 (44·2–63·6)	97·8 (81·1–114·7)	14·5 (9·6–19·9)	20·4 (14·6–27·9)	16·4 (8·8–26·4)	0·0 (0·0–0·0)	567·9 (511·5–627·1)
	Norway	958·4 (856·4–1073·5)	51·5 (44·6–60·5)	184·1 (106·6–292·9)	16·3 (9·4–30·1)	13·7 (9·7–19·1)	9·9 (4·7–19·5)	0·0 (0·0–0·0)	682·8 (614·9–753·7)
	Portugal	268·8 (249·0–291·1)	34·2 (27·0–41·9)	78·2 (70·6–86·8)	14·0 (10·8–18·0)	18·9 (14·0–25·4)	3·7 (3·1–4·3)	0·7 (0·3–1·6)	119·1 (104·1–134·7)
	San Marino	407·7 (373·0–444·4)	43·0 (35·5–51·8)	84·3 (69·9–100·2)	19·9 (14·2–29·2)	21·9 (12·1–41·6)	5·6 (4·4–7·0)	0·0 (0·0–0·0)	233·1 (207·3–261·5)
	Spain	366·7 (340·6–397·3)	64·3 (56·4–74·4)	74·3 (64·6–87·2)	27·0 (22·4–32·5)	17·7 (13·1–24·7)	2·2 (1·8–2·5)	0·0 (0·0–0·0)	181·2 (160·2–204·5)
	Sweden	862·3 (795·8–937·5)	52·9 (40·8–68·8)	112·6 (94·4–134·6)	14·5 (10·1–21·1)	20·3 (11·8–35·8)	9·1 (8·1–10·5)	0·0 (0·0–0·0)	653·0 (586·8–719·0)
	Switzerland	766·0 (705·8–830·1)	42·4 (36·4–50·8)	184·1 (157·7–210·7)	24·4 (17·5–33·9)	24·2 (14·9–38·2)	4·1 (3·5–4·9)	0·0 (0·0–0·0)	486·7 (430·8–547·1)
	UK	494·9 (456·3–540·0)	41·4 (35·2–49·2)	87·7 (72·0–107·2)	12·3 (8·8–17·0)	25·7 (15·5–41·2)	5·8 (4·1–8·2)	0·1 (0·0–0·1)	321·9 (286·4–358·0)
**Latin America and Caribbean**		**129·9 (121·6–140·6)**	**23·1 (20·3–26·6)**	**41·6 (35·3–49·0)**	**4·7 (3·9–6·2)**	**14·3 (11·4–17·6)**	**1·1 (0·8–1·6)**	**10·8 (9·7–12·2)**	**34·3 (29·9–38·9)**
Andean Latin America		108·5 (100·3–116·0)	19·5 (17·1–22·8)	31·0 (25·7–36·5)	4·6 (3·5–6·2)	7·6 (5·8–9·9)	4·0 (3·7–4·3)	0·4 (0·3–0·7)	41·4 (36·1–46·9)
	Bolivia	134·2 (117·5–152·2)	15·6 (10·7–23·1)	47·5 (36·8–61·4)	11·2 (7·6–16·1)	16·7 (11·9–23·9)	1·1 (0·4–2·3)	0·1 (0·0–0·1)	42·0 (36·6–47·6)
	Ecuador	108·8 (93·7–125·2)	29·5 (20·1–39·5)	30·9 (20·4–44·0)	1·6 (0·9–2·8)	11·8 (7·3–17·3)	0·9 (0·5–1·6)	1·6 (0·9–2·5)	32·5 (28·3–36·9)
	Peru	99·9 (90·7–110·6)	15·9 (12·2–20·1)	25·5 (20·0–32·8)	3·9 (2·5–6·7)	2·6 (1·1–5·4)	6·4 (6·0–6·9)	0·0 (0·0–0·0)	45·6 (39·7–51·7)
Caribbean		140·4 (124·8–161·1)	45·0 (38·6–56·3)	39·0 (27·9–54·6)	6·2 (4·6–11·1)	9·1 (7·1–11·9)	2·0 (0·7–4·8)	3·5 (1·6–8·3)	35·7 (31·2–40·4)
	Antigua and Barbuda	218·5 (182·4–259·8)	34·1 (18·5–58·7)	62·1 (39·7–90·4)	9·5 (3·3–25·8)	3·3 (1·4–6·4)	28·2 (18·8–41·8)	3·1 (1·4–7·2)	78·2 (68·3–88·6)
	The Bahamas	341·9 (304·8–386·9)	27·2 (13·4–49·6)	44·0 (27·2–63·5)	10·6 (3·6–33·7)	6·6 (2·8–13·3)	NA	0·1 (0·1–0·4)	249·7 (221·5–280·4)
	Barbados	205·6 (183·0–226·4)	28·0 (21·5–36·1)	40·5 (33·1–49·1)	15·3 (9·7–23·0)	7·1 (2·9–15·2)	5·2 (2·7–9·3)	0·6 (0·3–1·4)	108·9 (95·3–123·3)
	Belize	77·3 (64·9–91·3)	14·5 (7·1–28·1)	22·5 (17·5–29·3)	10·0 (5·6–15·9)	3·2 (1·5–6·6)	1·9 (0·6–4·3)	4·9 (2·2–11·9)	20·1 (17·5–22·8)
	Bermuda	748·9 (650·5–856·3)	58·1 (21·3–114·7)	76·6 (32·2–165·8)	15·3 (5·2–43·2)	14·4 (5·6–30·8)	3·6 (0·9–9·6)	0·2 (0·1–0·5)	580·6 (523·6–642·6)
	Cuba	283·4 (249·4–322·0)	96·6 (80·9–115·2)	77·1 (53·2–108·8)	10·2 (5·0–24·0)	24·1 (17·8–34·1)	2·6 (0·6–6·8)	4·5 (1·9–10·8)	68·4 (59·7–77·5)
	Dominica	130·0 (99·2–170·9)	15·8 (8·0–29·7)	69·6 (43·5–103·7)	9·9 (3·3–27·5)	4·9 (1·9–9·6)	NA	3·9 (1·7–9·4)	22·7 (19·8–25·8)
	Dominican Republic	122·1 (109·8–136·2)	54·6 (45·9–64·3)	28·4 (20·9–37·2)	3·4 (2·1–5·3)	5·9 (3·6–9·8)	2·3 (0·6–6·0)	3·3 (1·6–7·5)	24·3 (21·2–27·6)
	Grenada	118·0 (95·0–145·5)	18·0 (9·3–33·7)	52·5 (34·0–72·8)	11·4 (5·6–19·6)	4·2 (1·8–8·8)	NA	3·8 (1·7–6·4)	25·6 (22·4–29·1)
	Guyana	104·6 (87·2–128·7)	16·0 (8·5–28·0)	46·9 (31·2–66·1)	5·7 (2·8–11·7)	5·1 (2·4–9·6)	4·5 (3·1–6·6)	6·6 (4·2–10·0)	19·8 (17·2–22·4)
	Haiti	24·9 (18·9–34·8)	3·1 (1·2–7·3)	7·6 (3·3–16·1)	1·0 (0·3–3·1)	0·3 (0·1–0·8)	0·3 (0·1–0·7)	4·0 (1·8–9·6)	8·5 (7·4–9·7)
	Jamaica	74·5 (64·2–86·8)	5·7 (3·0–10·2)	17·8 (11·6–24·8)	5·0 (2·6–10·6)	3·0 (1·5–5·9)	1·6 (0·6–3·3)	3·4 (1·8–6·2)	38·0 (33·1–43·1)
	Puerto Rico	221·0 (145·4–353·2)	63·1 (23·0–151·3)	85·2 (35·8–184·3)	18·3 (5·5–58·6)	15·1 (5·8–33·9)	4·6 (1·1–12·1)	0·0 (0·0–0·0)	34·7 (30·3–39·4)
	Saint Kitts and Nevis	206·9 (182·4–240·1)	36·8 (22·5–56·9)	65·6 (51·0–85·1)	10·5 (3·6–29·7)	8·5 (3·7–15·9)	NA	0·8 (0·3–1·3)	81·8 (71·4–92·6)
	Saint Lucia	155·1 (124·8–187·1)	61·3 (41·3–88·6)	43·7 (26·8–65·0)	7·1 (3·0–16·6)	4·9 (2·1–10·6)	8·0 (6·1–10·4)	4·4 (1·9–10·8)	25·6 (22·3–29·1)
	Saint Vincent and the Grenadines	106·6 (80·7–143·1)	18·8 (7·8–43·9)	61·2 (40·2–88·0)	5·3 (1·8–12·8)	4·6 (1·8–10·5)	2·5 (0·6–6·6)	4·4 (2·0–9·9)	9·8 (8·6–11·2)
	Suriname	93·5 (81·0–106·6)	14·9 (10·6–20·1)	26·4 (18·1–37·1)	5·0 (2·6–8·8)	3·4 (1·8–6·0)	1·7 (0·8–3·2)	3·9 (1·9–8·4)	38·1 (33·2–43·2)
	Trinidad and Tobago	151·6 (134·8–170·2)	47·8 (38·7–58·8)	45·8 (37·2–56·4)	16·6 (9·1–28·2)	6·5 (3·4–12·3)	0·5 (0·1–1·4)	0·2 (0·1–0·4)	34·3 (30·0–38·9)
	Virgin Islands	265·9 (194·3–391·3)	66·3 (24·2–159·0)	78·5 (32·9–169·8)	16·8 (5·5–49·3)	14·8 (5·9–31·4)	3·8 (1·0–10·2)	0·1 (0·0–0·2)	85·7 (74·9–97·1)
Central Latin America		104·2 (96·0–114·7)	21·6 (16·9–26·5)	30·7 (26·5–35·1)	4·4 (3·8–5·4)	12·8 (8·5–19·8)	0·6 (0·4–0·8)	6·3 (4·9–8·3)	27·8 (24·3–31·6)
	Colombia	135·8 (116·5–163·3)	26·4 (20·9–32·7)	20·4 (15·0–26·9)	13·7 (11·3–17·3)	31·3 (15·1–58·5)	0·7 (0·2–1·8)	2·8 (1·2–6·7)	40·6 (35·4–46·1)
	Costa Rica	139·1 (122·9–158·9)	19·9 (14·2–27·4)	28·3 (22·5–35·8)	20·2 (14·3–27·3)	27·4 (19·0–42·5)	0·5 (0·1–1·5)	1·0 (0·5–2·3)	41·6 (36·3–47·2)
	El Salvador	84·5 (74·4–96·3)	16·2 (11·9–21·7)	30·4 (24·8–37·3)	1·0 (0·5–1·7)	12·9 (9·3–18·3)	0·2 (0·1–0·5)	10·9 (7·9–14·9)	12·8 (11·2–14·6)
	Guatemala	69·8 (57·8–86·0)	15·0 (7·6–27·1)	26·0 (18·5–35·4)	5·4 (3·0–9·2)	6·5 (3·1–12·4)	0·2 (0·0–0·4)	0·2 (0·1–0·5)	16·5 (14·4–18·7)
	Honduras	40·1 (31·3–50·6)	8·3 (3·9–15·7)	11·3 (6·4–18·2)	1·9 (0·7–4·8)	7·0 (3·7–11·3)	3·1 (2·2–4·7)	7·0 (3·6–11·8)	1·6 (1·4–1·8)
	Mexico	110·7 (100·1–121·8)	24·6 (18·4–31·0)	39·3 (32·2–46·6)	1·1 (0·6–1·8)	8·9 (6·7–11·4)	0·3 (0·1–0·6)	10·1 (6·7–14·5)	26·5 (23·1–30·0)
	Nicaragua	63·3 (48·8–85·3)	10·9 (3·8–20·7)	20·6 (11·6–36·0)	1·7 (0·6–4·2)	7·4 (3·0–15·9)	0·5 (0·1–1·3)	3·6 (1·6–8·7)	18·7 (16·3–21·2)
	Panama	180·6 (162·7–198·9)	17·8 (13·1–25·1)	49·4 (40·4–59·8)	6·2 (4·2–8·6)	8·5 (5·6–12·8)	3·7 (2·9–4·7)	0·6 (0·3–1·4)	94·4 (82·4–106·9)
	Venezuela	62·9 (50·8–79·5)	13·6 (7·1–24·0)	19·8 (11·7–30·3)	2·8 (1·0–6·8)	4·4 (2·0–9·4)	0·5 (0·1–1·4)	0·0 (0·0–0·0)	21·7 (19·0–24·7)
Tropical Latin America		164·9 (147·3–188·2)	21·3 (16·4–27·6)	58·4 (44·2–76·9)	4·8 (3·1–7·6)	19·0 (14·0–24·6)	0·7 (0·3–1·3)	21·0 (16·6–26·0)	39·7 (34·7–45·1)
	Brazil	167·1 (149·4–190·5)	21·4 (16·0–28·1)	59·3 (45·0–77·9)	4·7 (3·1–7·5)	19·4 (14·3–25·1)	NA	21·5 (17·1–26·7)	40·2 (35·1–45·6)
	Paraguay	94·3 (77·0–114·6)	18·7 (10·0–32·0)	28·4 (17·2–45·5)	6·5 (3·1–11·4)	7·4 (3·2–15·5)	6·8 (4·4–10·0)	2·4 (1·1–5·6)	24·1 (21·0–27·3)
**North Africa and Middle East**		**76·4 (72·1–81·5)**	**14·5 (12·7–17·5)**	**21·8 (20·0–24·1)**	**9·2 (7·3–12·0)**	**5·1 (4·5–6·0)**	**3·0 (2·8–3·4)**	**1·1 (0·9–1·5)**	**21·7 (19·0–24·5)**
Afghanistan		23·1 (18·8–28·7)	4·0 (1·9–7·9)	4·8 (2·4–8·9)	1·7 (0·8–3·9)	0·4 (0·2–0·8)	2·2 (1·8–2·8)	7·9 (6·9–9·1)	2·0 (1·7–2·2)
Algeria		52·5 (42·7–63·0)	18·3 (12·9–25·2)	13·4 (8·0–20·7)	5·1 (2·3–10·2)	4·0 (2·2–6·3)	2·1 (1·7–2·6)	0·3 (0·1–0·7)	9·4 (8·2–10·7)
Bahrain		170·2 (151·5–190·7)	9·7 (5·3–16·4)	26·2 (18·4–36·6)	4·2 (2·1–8·6)	1·6 (1·1–2·2)	NA	0·0 (0·0–0·1)	125·3 (109·7–141·8)
Egypt		64·3 (52·8–80·8)	11·5 (7·1–18·3)	24·7 (16·7–35·3)	8·5 (3·7–17·4)	3·1 (1·8–5·4)	1·5 (0·4–4·4)	0·2 (0·1–0·3)	14·8 (12·9–16·8)
Iran		77·4 (66·6–87·6)	18·9 (13·8–25·2)	22·2 (15·3–30·3)	6·1 (3·4–10·3)	6·7 (4·5–9·3)	8·7 (8·2–9·4)	3·1 (1·9–5·1)	11·7 (10·2–13·3)
Iraq		51·1 (43·1–59·8)	11·1 (7·4–16·1)	12·3 (8·0–17·2)	10·4 (6·9–14·3)	5·3 (3·5–7·4)	2·0 (1·5–2·6)	0·7 (0·3–1·3)	9·4 (8·2–10·6)
Jordan		88·1 (77·3–100·0)	9·7 (5·5–15·6)	29·9 (23·7–37·5)	15·9 (10·7–22·8)	8·6 (6·1–12·1)	6·4 (5·0–8·3)	0·0 (0·0–0·0)	17·6 (15·3–20·0)
Kuwait		290·5 (260·0–330·4)	25·4 (17·4–36·6)	64·9 (47·5–87·9)	16·1 (9·4–25·3)	11·4 (6·7–18·0)	NA	0·0 (0·0–0·0)	168·8 (148·3–190·8)
Lebanon		90·0 (76·8–108·2)	26·9 (18·6–38·3)	18·4 (11·3–27·6)	18·2 (11·6–27·6)	12·2 (8·6–17·3)	1·4 (0·8–2·2)	0·2 (0·1–0·6)	12·7 (11·0–14·4)
Libya		110·2 (84·2–142·7)	22·3 (12·0–37·6)	57·9 (35·9–86·9)	8·5 (4·6–16·2)	8·4 (5·1–12·4)	1·8 (0·9–3·2)	0·2 (0·1–0·5)	11·2 (9·7–12·7)
Morocco		36·1 (27·4–47·7)	9·7 (5·0–17·9)	11·1 (5·9–18·6)	3·8 (1·5–9·1)	2·4 (1·0–4·7)	1·9 (1·5–2·4)	0·3 (0·1–0·7)	6·9 (6·0–7·8)
Oman		123·2 (113·5–136·1)	21·1 (15·9–27·8)	43·6 (36·0–52·9)	12·3 (9·0–16·0)	4·6 (3·2–6·3)	2·0 (1·6–2·5)	0·2 (0·1–0·5)	39·4 (34·4–44·7)
Palestine		61·5 (40·1–91·7)	10·3 (3·8–24·5)	34·6 (17·6–60·9)	6·4 (1·2–26·7)	3·0 (0·9–7·7)	2·5 (1·5–3·7)	0·3 (0·1–0·7)	4·4 (3·8–5·0)
Qatar		391·6 (358·0–427·7)	28·7 (22·6–36·6)	81·1 (68·6–95·7)	14·0 (10·2–17·8)	10·4 (8·0–13·2)	1·2 (0·8–1·6)	0·2 (0·1–0·5)	255·9 (226·4–287·8)
Saudi Arabia		204·7 (191·6–219·0)	34·7 (30·0–40·1)	63·8 (56·8–71·8)	15·5 (12·3–21·0)	10·1 (7·6–13·7)	1·5 (1·2–1·8)	1·3 (0·8–2·0)	77·9 (68·0–88·3)
Sudan		38·7 (27·3–53·7)	6·7 (2·7–14·9)	17·5 (9·6–29·5)	3·5 (1·1–10·7)	4·1 (1·9–7·9)	4·6 (2·5–7·4)	0·5 (0·2–0·9)	1·7 (1·5–1·9)
Syria		87·1 (76·5–100·8)	20·5 (14·5–28·8)	20·0 (14·5–27·6)	22·1 (15·9–30·3)	13·2 (10·1–17·5)	2·3 (1·7–3·3)	0·3 (0·1–0·7)	8·6 (7·5–9·8)
Tunisia		55·7 (45·5–67·3)	14·6 (9·6–21·5)	25·0 (17·8–33·5)	3·7 (1·8–6·9)	4·9 (2·9–8·1)	2·3 (1·0–4·6)	0·3 (0·1–0·6)	4·8 (4·2–5·4)
Türkiye		79·1 (67·1–92·3)	16·1 (11·4–22·4)	15·5 (10·4–21·5)	18·7 (10·9–29·0)	6·1 (4·2–8·2)	1·7 (1·3–2·2)	0·0 (0·0–0·0)	21·0 (18·3–23·9)
United Arab Emirates		418·9 (383·6–458·4)	31·0 (26·2–36·8)	66·1 (54·4–80·5)	17·5 (14·2–20·9)	12·7 (10·0–16·3)	0·8 (0·4–1·3)	0·0 (0·0–0·0)	290·9 (257·4–327·0)
Yemen		17·5 (13·6–22·6)	2·1 (0·9–4·7)	6·6 (3·7–10·3)	1·9 (0·7–4·9)	0·5 (0·2–1·0)	1·6 (1·4–2·0)	0·1 (0·0–0·2)	4·6 (4·0–5·2)
**South Asia**		**68·6 (60·7–78·5)**	**10·0 (6·6–15·7)**	**10·2 (6·6–15·6)**	**8·2 (5·4–12·6)**	**2·2 (1·3–3·6)**	**1·7 (0·9–3·0)**	**13·6 (10·5–17·6)**	**22·8 (19·9–25·9)**
Bangladesh		56·7 (50·4–64·0)	7·9 (5·3–12·1)	5·7 (3·1–9·4)	11·0 (9·5–14·2)	1·2 (0·9–1·5)	1·7 (1·0–2·6)	7·1 (4·4–10·4)	22·1 (19·3–25·0)
Bhutan		95·7 (84·6–109·7)	8·6 (5·7–12·6)	30·5 (24·1–38·5)	5·8 (3·8–8·2)	3·4 (2·7–4·2)	0·8 (0·3–2·1)	12·3 (7·8–17·6)	34·3 (29·9–38·9)
India		73·1 (63·4–86·2)	10·5 (6·1–18·0)	10·8 (6·0–18·2)	8·1 (4·4–13·7)	2·5 (1·3–4·3)	1·2 (0·4–2·6)	16·2 (12·2–21·4)	23·9 (20·8–27·1)
Nepal		85·2 (75·1–96·5)	10·1 (6·8–15·0)	29·1 (21·4–36·8)	6·2 (4·3–9·5)	2·7 (1·8–4·0)	12·3 (10·7–14·1)	16·6 (13·8–19·5)	8·2 (7·2–9·3)
Pakistan		49·0 (42·6–56·3)	8·4 (5·1–12·6)	7·2 (4·8–10·6)	7·2 (4·6–10·4)	1·0 (0·6–1·5)	3·2 (1·6–5·5)	3·0 (1·8–4·3)	19·1 (16·6–21·7)
**Southeast Asia, east Asia, and Oceania**		**118·1 (108·6–130·0)**	**23·9 (18·2–31·8)**	**42·0 (37·8–47·6)**	**7·3 (5·0–10·5)**	**8·1 (4·5–13·9)**	**3·9 (2·7–5·5)**	**13·5 (10·9–16·1)**	**19·6 (17·1–22·2)**
East Asia		129·2 (116·4–145·2)	31·1 (23·6–40·9)	51·2 (44·5–59·9)	8·2 (5·3–11·7)	10·6 (5·5–18·9)	1·0 (0·5–2·1)	5·6 (3·4–7·9)	21·6 (18·9–24·5)
	China	129·1 (115·2–146·0)	31·7 (23·7–41·7)	50·5 (43·1–59·8)	7·8 (4·9–11·5)	10·6 (5·0–19·3)	0·7 (0·2–1·8)	5·6 (3·3–8·1)	22·1 (19·3–25·1)
	North Korea	79·7 (59·9–108·0)	10·0 (3·6–23·9)	44·9 (29·3–67·9)	7·7 (3·9–16·4)	3·9 (1·5–8·2)	4·0 (1·1–10·2)	5·4 (2·3–13·1)	3·8 (3·3–4·3)
	Taiwan*	191·2 (139·5–255·6)	17·3 (7·8–33·9)	97·5 (58·2–149·5)	31·6 (14·7–67·3)	19·0 (9·5–35·0)	13·8 (5·1–30·2)	0·0 (0·0–0·0)	12·0 (10·4–13·6)
Oceania		28·1 (23·6–34·0)	2·6 (1·8–4·5)	12·8 (10·4–18·3)	1·0 (0·5–2·2)	1·1 (0·7–1·8)	1·6 (0·8–3·5)	5·4 (2·9–9·9)	3·6 (3·1–4·1)
	American Samoa	125·9 (77·4–209·8)	16·1 (5·9–38·6)	78·1 (32·8–169·0)	6·2 (2·2–16·6)	9·1 (3·6–19·5)	6·7 (1·7–17·8)	4·1 (1·8–9·9)	5·7 (4·9–6·4)
	Cook Islands	190·1 (165·1–220·2)	17·6 (11·0–27·5)	80·0 (63·3–101·4)	7·6 (4·0–14·9)	14·1 (8·0–21·9)	20·1 (8·8–38·8)	4·7 (2·1–11·4)	45·9 (40·1–52·1)
	Fiji	72·0 (55·0–92·2)	11·9 (5·8–22·6)	36·1 (22·9–52·0)	4·4 (1·9–10·3)	4·0 (2·3–6·3)	4·0 (1·8–8·1)	0·3 (0·1–0·8)	11·4 (10·0–13·0)
	Guam	207·0 (157·4–278·2)	24·0 (8·8–57·6)	139·8 (91·8–205·6)	9·8 (3·4–27·0)	16·1 (6·4–34·3)	8·6 (2·2–22·9)	0·1 (0·0–0·2)	8·6 (7·5–9·7)
	Kiribati	75·7 (53·1–102·8)	4·2 (1·6–9·8)	45·0 (26·4–67·4)	2·1 (0·9–4·4)	1·9 (0·8–3·6)	10·5 (3·0–24·0)	5·9 (2·6–14·4)	6·1 (5·3–7·0)
	Marshall Islands	85·3 (62·3–114·5)	8·5 (3·3–19·6)	49·4 (29·1–73·6)	2·5 (0·8–7·6)	5·3 (2·6–10·0)	4·7 (1·3–11·8)	8·4 (3·7–18·2)	6·6 (5·8–7·5)
	Federated States of Micronesia	58·6 (43·7–77·5)	10·6 (5·7–18·7)	25·9 (14·2–41·6)	3·0 (1·2–6·9)	3·2 (1·2–6·6)	2·6 (0·7–6·6)	3·5 (1·5–8·4)	9·8 (8·6–11·1)
	Nauru	143·7 (107·7–200·4)	14·1 (6·6–25·6)	88·4 (57·1–138·8)	10·9 (6·7–17·4)	7·4 (3·9–11·9)	5·9 (1·5–15·6)	6·6 (3·2–11·3)	10·4 (9·1–11·8)
	Niue	200·8 (150·5–261·3)	18·4 (7·3–35·7)	137·8 (99·8–192·6)	6·4 (2·6–16·3)	17·9 (7·2–36·0)	12·7 (3·5–37·6)	0·7 (0·3–1·6)	6·9 (6·0–7·8)
	Northern Mariana Islands	158·7 (96·4–267·8)	21·4 (7·8–51·3)	100·1 (42·1–216·7)	8·3 (3·0–21·8)	13·4 (5·3–28·5)	7·4 (1·9–19·6)	0·2 (0·1–0·6)	8·0 (6·9–9·0)
	Palau	187·9 (172·7–205·1)	23·1 (18·4–28·6)	72·4 (61·1–85·2)	8·3 (5·2–14·4)	11·5 (8·3–16·4)	1·7 (0·4–4·5)	6·4 (3·1–13·2)	64·4 (56·2–73·0)
	Papua New Guinea	16·1 (11·8–22·5)	1·0 (0·4–2·3)	5·7 (2·5–11·8)	0·3 (0·1–1·1)	0·3 (0·2–0·6)	0·8 (0·2–2·1)	5·8 (3·2–9·9)	2·1 (1·8–2·4)
	Samoa	53·8 (39·1–70·6)	6·4 (3·3–12·1)	28·6 (17·2–42·6)	1·9 (0·9–3·6)	3·0 (1·6–5·3)	5·1 (1·5–11·8)	5·1 (2·2–12·5)	3·7 (3·2–4·2)
	Solomon Islands	41·8 (31·5–55·7)	2·8 (1·1–6·3)	19·1 (10·5–31·7)	1·3 (0·4–4·1)	1·1 (0·7–1·6)	2·4 (0·6–6·3)	6·5 (2·8–15·8)	8·7 (7·6–9·9)
	Tokelau	323·1 (285·8–366·0)	20·7 (14·5–28·6)	197·4 (166·3–232·1)	6·3 (2·3–16·8)	19·0 (11·3–27·9)	51·3 (35·8–72·9)	4·0 (1·8–9·8)	24·4 (21·2–27·6)
	Tonga	80·7 (67·8–95·6)	11·2 (7·1–17·5)	46·4 (37·5–58·1)	3·7 (1·9–6·4)	5·4 (2·9–8·7)	3·4 (0·9–8·5)	4·9 (2·2–12·0)	5·6 (4·9–6·4)
	Tuvalu	87·7 (66·2–115·2)	13·9 (7·6–23·8)	45·8 (30·8–64·9)	4·0 (1·7–10·1)	3·6 (1·8–6·7)	11·5 (3·1–32·3)	5·3 (2·3–12·8)	3·7 (3·2–4·2)
	Vanuatu	35·5 (24·3–49·2)	2·3 (0·8–5·3)	17·1 (8·8–33·3)	1·3 (0·4–4·1)	1·1 (0·6–1·8)	3·3 (0·9–8·6)	7·2 (3·4–16·1)	3·3 (2·8–3·7)
Southeast Asia		96·8 (88·6–107·7)	9·5 (6·6–14·2)	23·5 (20·1–27·9)	5·5 (3·8–8·0)	2·9 (2·1–4·5)	9·9 (7·2–13·8)	29·9 (24·0–37·4)	15·6 (13·6–17·7)
	Cambodia	37·0 (28·1–48·3)	7·1 (3·5–12·5)	10·5 (5·9–16·3)	1·6 (0·6–4·2)	0·8 (0·4–1·4)	7·4 (3·2–16·4)	1·6 (0·7–4·0)	8·1 (7·0–9·2)
	Indonesia	75·6 (65·0–87·4)	6·6 (4·0–10·6)	26·8 (20·4–34·8)	4·0 (2·5–6·9)	1·8 (1·3–2·5)	16·3 (10·0–25·3)	2·0 (0·9–4·3)	18·2 (15·8–20·6)
	Laos	34·7 (28·2–44·1)	4·5 (2·3–8·4)	12·6 (8·0–18·0)	3·8 (1·7–8·7)	1·1 (0·6–1·7)	2·9 (0·9–6·5)	0·7 (0·3–1·6)	9·1 (7·9–10·3)
	Malaysia	126·8 (117·5–137·8)	24·5 (20·2–29·7)	46·8 (40·7–53·1)	8·6 (6·3–11·7)	6·8 (4·8–9·8)	0·5 (0·1–1·3)	3·8 (1·6–9·1)	35·8 (31·2–40·6)
	Maldives	144·9 (121·1–169·8)	17·7 (11·3–26·4)	66·9 (46·6–91·3)	15·9 (10·7–22·8)	2·7 (1·7–3·9)	3·2 (1·3–6·5)	9·0 (5·2–12·9)	29·5 (25·8–33·5)
	Mauritius	105·5 (89·1–124·1)	27·8 (19·7–38·9)	42·8 (34·0–54·1)	13·4 (7·9–21·1)	6·4 (3·2–11·9)	6·0 (1·6–14·8)	3·7 (1·7–8·7)	5·6 (4·9–6·3)
	Myanmar	32·3 (24·0–42·0)	8·6 (4·5–15·2)	9·6 (5·0–16·3)	2·1 (0·8–6·5)	1·1 (0·6–1·7)	4·7 (1·4–10·7)	0·7 (0·3–1·8)	5·5 (4·8–6·2)
	Philippines	132·7 (110·7–163·5)	9·2 (5·3–15·0)	16·0 (10·1–23·1)	10·8 (6·4–17·1)	4·8 (2·5–8·6)	8·5 (3·9–16·5)	74·9 (52·6–103·6)	8·4 (7·3–9·6)
	Seychelles	201·5 (178·2–226·4)	68·5 (54·5–85·5)	69·0 (56·0–83·2)	14·1 (10·2–18·7)	13·1 (8·9–18·1)	28·5 (19·6–39·0)	1·0 (0·4–2·5)	7·3 (6·3–8·3)
	Sri Lanka	56·0 (46·6–66·8)	11·7 (8·1–16·3)	22·2 (16·7–28·4)	2·9 (1·4–6·0)	1·2 (0·9–1·6)	5·4 (2·2–11·6)	4·0 (1·7–9·7)	8·7 (7·6–9·8)
	Thailand	214·8 (183·2–255·0)	11·9 (7·5–18·5)	28·3 (20·8–37·3)	5·0 (2·7–8·5)	4·7 (2·5–8·5)	2·8 (0·7–7·4)	149·0 (118·5–191·6)	13·2 (11·5–14·9)
	Timor-Leste	36·5 (28·0–45·5)	9·1 (4·9–15·9)	14·3 (9·0–20·7)	4·0 (1·9–9·4)	0·8 (0·4–1·5)	4·0 (1·9–7·4)	2·0 (0·9–4·9)	2·4 (2·1–2·7)
	Viet Nam	80·9 (64·6–104·7)	11·6 (7·0–18·4)	22·0 (11·6–40·9)	6·0 (2·7–12·8)	2·9 (1·2–7·0)	6·4 (1·9–13·9)	11·4 (5·2–23·9)	20·5 (17·9–23·3)
**Sub-Saharan Africa**		**41·2 (37·4–45·3)**	**2·9 (2·2–4·5)**	**14·5 (11·6–18·1)**	**1·8 (1·4–2·6)**	**0·7 (0·6–0·9)**	**2·7 (2·4–3·1)**	**10·1 (9·0–11·6)**	**8·5 (7·4–9·7)**
Central sub-Saharan Africa		62·2 (57·0–67·0)	3·0 (2·1–5·0)	15·5 (12·2–19·6)	0·8 (0·4–2·2)	0·5 (0·3–0·8)	0·8 (0·6–1·0)	35·7 (32·2–39·2)	5·9 (5·2–6·7)
	Angola	34·5 (24·9–43·9)	3·8 (1·9–6·9)	21·9 (13·9–31·5)	2·1 (0·9–4·6)	0·7 (0·4–1·1)	1·2 (0·5–2·4)	0·7 (0·3–1·6)	4·1 (3·6–4·7)
	Central African Republic	11·9 (9·3–15·6)	0·7 (0·3–1·7)	3·4 (1·6–6·8)	0·4 (0·2–0·8)	0·1 (0·1–0·3)	1·0 (0·5–1·9)	2·8 (1·8–4·1)	3·4 (3·0–3·9)
	Congo (Brazzaville)	27·2 (21·1–36·0)	2·1 (0·9–4·7)	12·1 (6·6–20·2)	0·5 (0·2–1·2)	0·6 (0·2–1·4)	2·0 (1·4–3·0)	1·7 (0·8–4·2)	8·1 (7·1–9·2)
	DR Congo	77·9 (69·5–86·4)	2·8 (1·1–6·2)	13·9 (8·3–20·7)	0·4 (0·1–1·3)	0·4 (0·2–0·8)	0·5 (0·2–0·9)	54·2 (49·0–59·7)	5·9 (5·1–6·6)
	Equatorial Guinea	47·8 (39·5–59·2)	2·9 (1·1–6·5)	15·3 (8·6–25·2)	1·2 (0·5–2·4)	1·2 (0·5–2·4)	0·3 (0·1–0·7)	0·9 (0·4–1·9)	26·1 (22·7–29·6)
	Gabon	73·3 (62·9–85·5)	8·6 (5·4–13·9)	32·0 (23·4–43·3)	2·5 (1·1–5·5)	0·7 (0·4–1·1)	4·9 (4·0–5·9)	0·7 (0·3–1·6)	23·9 (20·9–27·1)
Eastern sub-Saharan Africa		33·0 (29·3–36·7)	3·5 (2·7–5·3)	11·0 (8·6–13·7)	1·7 (1·1–2·8)	0·5 (0·3–0·7)	2·0 (1·7–2·4)	7·1 (5·6–9·1)	7·3 (6·3–8·2)
	Burundi	28·4 (22·8–35·0)	2·3 (0·9–5·1)	9·4 (5·7–13·9)	0·6 (0·1–2·2)	0·2 (0·1–0·4)	0·3 (0·1–0·6)	9·0 (5·9–12·6)	6·6 (5·8–7·5)
	Comoros	40·1 (31·7–49·9)	10·3 (6·5–16·1)	10·4 (5·8–16·6)	2·5 (1·1–5·7)	0·9 (0·5–1·4)	6·8 (5·7–7·9)	4·0 (1·7–9·6)	5·3 (4·6–6·0)
	Djibouti	22·0 (15·5–31·8)	5·3 (2·3–11·3)	3·9 (1·8–8·5)	3·2 (0·9–10·0)	0·9 (0·3–1·7)	0·9 (0·4–1·9)	3·8 (1·7–9·2)	4·0 (3·5–4·5)
	Eritrea	25·8 (20·8–32·7)	1·6 (0·6–3·6)	9·3 (5·2–14·4)	2·0 (0·8–5·0)	0·6 (0·4–1·1)	0·3 (0·1–0·6)	5·7 (3·3–9·3)	6·3 (5·5–7·2)
	Ethiopia	27·7 (21·9–35·2)	3·8 (1·7–7·9)	10·5 (6·2–16·1)	2·6 (1·3–5·5)	0·5 (0·3–0·9)	2·0 (1·5–2·9)	3·8 (2·8–4·8)	4·4 (3·8–5·0)
	Kenya	58·5 (50·9–67·2)	3·0 (1·3–6·6)	20·6 (14·5–28·1)	2·2 (1·1–4·7)	0·6 (0·3–0·9)	0·2 (0·1–0·4)	18·5 (15·1–22·7)	13·4 (11·7–15·2)
	Madagascar	37·7 (32·3–44·8)	4·9 (2·1–10·3)	3·3 (1·4–6·9)	0·2 (0·1–0·9)	0·3 (0·1–0·7)	1·5 (0·8–2·2)	13·6 (10·0–17·2)	13·9 (12·1–15·7)
	Malawi	24·5 (19·5–31·5)	2·3 (0·9–5·1)	7·4 (3·7–12·9)	0·8 (0·2–2·7)	0·4 (0·2–0·7)	0·4 (0·1–1·0)	5·6 (2·9–10·5)	7·7 (6·8–8·8)
	Mozambique	18·4 (15·1–22·9)	2·4 (1·1–5·1)	4·6 (2·3–8·3)	0·9 (0·4–1·9)	0·4 (0·2–0·6)	3·4 (2·4–4·6)	2·1 (1·0–4·7)	4·7 (4·1–5·3)
	Rwanda	63·1 (50·6–77·5)	9·5 (6·7–13·1)	11·9 (8·8–15·7)	1·6 (0·8–3·3)	0·5 (0·3–0·9)	1·3 (0·9–2·0)	34·6 (23·4–47·0)	3·6 (3·2–4·1)
	Somalia	10·2 (7·1–15·0)	1·3 (0·5–3·1)	4·1 (1·7–8·9)	0·4 (0·1–1·6)	0·1 (0·0–0·3)	0·8 (0·2–2·0)	1·4 (0·6–3·3)	2·0 (1·7–2·3)
	South Sudan	19·7 (15·4–25·7)	1·6 (0·6–3·8)	5·7 (2·9–10·1)	1·2 (0·4–3·7)	0·3 (0·2–0·6)	3·2 (2·4–4·2)	2·6 (1·2–6·0)	5·1 (4·4–5·7)
	Uganda	32·7 (26·0–40·0)	2·6 (1·1–5·2)	12·5 (7·6–19·2)	1·2 (0·4–3·3)	0·6 (0·3–0·9)	7·6 (6·0–9·6)	4·7 (2·6–8·0)	3·6 (3·1–4·0)
	Tanzania	30·5 (23·4–38·1)	4·1 (1·9–7·8)	10·6 (6·1–16·0)	2·0 (0·9–4·7)	0·6 (0·3–1·1)	0·6 (0·2–1·3)	3·4 (1·6–7·6)	9·1 (8·0–10·4)
	Zambia	44·1 (36·8–53·5)	4·1 (2·0–7·0)	21·6 (15·6–30·5)	3·1 (1·6–5·9)	0·5 (0·3–0·9)	0·9 (0·5–1·5)	1·3 (0·7–1·9)	12·6 (11·0–14·3)
Southern sub-Saharan Africa		88·0 (69·8–112·2)	7·8 (5·1–12·0)	50·2 (32·2–72·5)	4·6 (3·3–6·3)	2·3 (1·6–3·1)	1·6 (1·2–2·1)	11·0 (10·4–11·8)	10·5 (9·2–11·9)
	Botswana	64·1 (54·5–74·6)	4·8 (2·5–8·3)	33·5 (25·2–42·7)	3·6 (2·2–6·4)	1·6 (1·3–2·1)	0·6 (0·2–1·4)	1·8 (0·7–3·3)	18·1 (15·8–20·6)
	eSwatini	87·2 (77·2–98·8)	6·0 (3·2–11·5)	44·3 (35·4–54·9)	1·8 (0·8–3·5)	0·6 (0·3–1·1)	1·2 (0·3–3·3)	26·8 (25·4–28·2)	6·5 (5·6–7·3)
	Lesotho	102·7 (94·3–113·4)	4·5 (1·8–9·9)	9·7 (4·1–20·7)	3·4 (1·7–7·2)	1·1 (0·6–1·8)	1·2 (0·3–3·1)	76·1 (72·9–79·4)	6·7 (5·9–7·6)
	Namibia	60·1 (51·0–70·6)	5·7 (3·2–9·5)	26·2 (18·6–35·1)	3·8 (2·1–6·0)	1·5 (1·1–1·9)	2·0 (1·0–3·8)	8·5 (6·8–10·5)	12·5 (10·9–14·2)
	South Africa	97·5 (74·4–128·5)	9·6 (6·2–14·5)	59·5 (36·5–89·0)	5·3 (3·6–7·7)	2·8 (1·8–4·0)	0·6 (0·3–0·9)	8·8 (7·7–10·0)	11·0 (9·6–12·5)
	Zimbabwe	56·7 (47·1–65·6)	2·1 (0·9–4·6)	25·4 (17·6–34·4)	2·2 (0·9–5·2)	0·6 (0·4–0·9)	6·1 (4·6–8·0)	12·6 (10·6–14·8)	7·8 (6·8–8·8)
Western sub-Saharan Africa		34·2 (31·1–38·1)	1·5 (1·1–2·6)	11·0 (9·0–13·3)	1·6 (1·2–2·5)	0·7 (0·5–0·8)	4·1 (3·4–4·9)	5·3 (4·0–6·8)	10·0 (8·7–11·3)
	Benin	27·0 (21·9–34·0)	0·8 (0·3–1·8)	14·6 (9·9–20·1)	1·8 (0·6–5·2)	0·5 (0·2–0·9)	1·6 (1·2–2·2)	3·1 (2·1–4·6)	4·6 (4·0–5·2)
	Burkina Faso	20·0 (16·2–25·3)	0·8 (0·3–1·9)	8·1 (4·7–12·0)	0·7 (0·2–2·7)	0·3 (0·1–0·5)	3·8 (3·0–4·9)	1·3 (0·6–3·0)	5·0 (4·4–5·7)
	Cabo Verde	44·6 (38·5–52·0)	3·6 (1·7–7·2)	23·5 (18·2–29·8)	0·7 (0·2–2·0)	1·6 (0·7–3·4)	NA	1·3 (0·6–2·9)	12·9 (11·2–14·6)
	Cameroon	16·4 (12·7–21·5)	1·8 (0·7–3·8)	6·4 (3·1–11·2)	0·5 (0·2–1·7)	0·3 (0·1–0·6)	0·5 (0·2–1·2)	0·1 (0·1–0·3)	6·7 (5·8–7·6)
	Chad	11·4 (8·6–14·8)	0·6 (0·2–1·3)	3·2 (1·6–5·8)	0·4 (0·1–1·1)	0·1 (0·1–0·3)	0·5 (0·2–0·9)	4·5 (2·7–7·5)	2·1 (1·8–2·4)
	Côte d’Ivoire	30·9 (25·3–36·7)	1·1 (0·4–2·6)	14·0 (9·7–19·3)	1·0 (0·3–2·9)	0·4 (0·2–0·7)	2·8 (2·2–3·6)	4·6 (2·7–7·4)	7·2 (6·2–8·1)
	The Gambia	29·1 (24·0–34·9)	0·8 (0·3–1·9)	8·9 (5·1–14·2)	0·7 (0·3–1·3)	0·3 (0·1–0·5)	1·9 (1·2–2·9)	6·4 (4·1–9·4)	10·2 (8·9–11·5)
	Ghana	74·7 (65·6–84·6)	3·3 (1·7–6·1)	40·8 (32·7–49·5)	7·6 (4·7–11·4)	1·0 (0·6–1·6)	8·2 (6·6–10·3)	5·6 (3·4–8·0)	8·2 (7·2–9·4)
	Guinea	23·5 (18·5–29·7)	1·4 (0·6–2·9)	6·4 (3·3–10·3)	0·5 (0·1–1·8)	0·2 (0·1–0·6)	1·1 (0·5–1·9)	12·0 (8·6–15·2)	1·8 (1·6–2·0)
	Guinea-Bissau	46·7 (39·1–54·2)	1·3 (0·5–3·0)	18·2 (12·5–25·2)	0·5 (0·2–0·9)	0·3 (0·1–0·5)	1·1 (0·5–1·8)	21·3 (17·8–25·5)	4·1 (3·5–4·6)
	Liberia	38·8 (32·8–46·3)	0·9 (0·3–2·2)	8·1 (4·2–13·9)	1·2 (0·5–2·7)	0·2 (0·1–0·4)	1·7 (1·2–2·4)	23·0 (19·2–27·3)	3·7 (3·2–4·2)
	Mali	12·2 (9·0–17·1)	1·0 (0·4–2·4)	4·6 (2·3–8·8)	0·9 (0·2–3·5)	0·2 (0·1–0·4)	1·5 (0·9–2·4)	1·2 (0·6–2·9)	2·8 (2·4–3·2)
	Mauritania	29·3 (22·7–37·6)	1·3 (0·5–2·8)	16·9 (10·7–24·0)	1·1 (0·4–3·0)	0·7 (0·4–1·3)	3·3 (2·4–4·5)	1·4 (0·6–3·3)	4·7 (4·1–5·3)
	Niger	15·6 (11·9–20·2)	0·9 (0·3–2·0)	4·3 (2·1–8·0)	0·2 (0·1–0·6)	0·1 (0·0–0·3)	0·6 (0·3–1·0)	7·7 (5·0–10·8)	1·9 (1·6–2·1)
	Nigeria	41·4 (35·6–47·4)	1·7 (0·8–3·5)	9·8 (6·0–14·4)	1·7 (0·8–3·6)	1·0 (0·7–1·4)	6·0 (4·4–7·7)	5·5 (3·7–7·6)	15·6 (13·6–17·7)
	São Tomé and Príncipe	42·9 (33·1–54·5)	5·4 (2·4–11·1)	22·8 (14·6–32·4)	2·7 (1·2–6·3)	0·9 (0·4–1·9)	2·1 (1·3–3·2)	2·3 (1·0–3·9)	6·8 (5·9–7·7)
	Senegal	25·0 (20·7–30·1)	0·8 (0·4–1·5)	7·2 (4·0–11·1)	0·5 (0·2–1·3)	0·3 (0·2–0·5)	1·6 (1·2–2·0)	9·8 (7·5–12·7)	4·9 (4·2–5·5)
	Sierra Leone	28·3 (22·0–36·1)	2·0 (0·9–4·2)	14·5 (8·6–21·3)	1·0 (0·4–2·4)	0·4 (0·2–0·7)	2·9 (1·9–4·1)	0·9 (0·4–2·0)	6·7 (5·8–7·6)
	Togo	25·9 (21·8–31·2)	0·7 (0·3–1·8)	5·1 (2·6–9·1)	0·5 (0·1–2·1)	0·2 (0·1–0·5)	2·3 (1·7–3·1)	8·8 (6·7–11·4)	8·2 (7·2–9·4)

Density is reported in count of workers per 10 000 population. Data in parentheses are 95% uncertainty intervals. GBD=Global Burden of Diseases, Injuries, and Risk Factors Study. NA=not applicable (no estimate provided because available evidence does not indicate the existence of a distinct midwifery cadre separate from nursing in that location). SDI=Socio-demographic Index.

* The United Nations recognises Taiwan as a province of China.

Of total HRH in 2023, an estimated 68·9% (95% UI 66·9–70·6) were female. 43·9% (34·5–53·2) of doctors were female, compared with 80·7% (76·2–83·1) of nurses, 96·0% (83·9–98·5) of midwives, 89·5% (83·6–93·7) of CHWs, and the majority of other cadres in 2023 (figure 1). The majority of dentists and dental assistants (66·9%, 61·1–72·1) and pharmacists and pharmaceutical assistants (63·0%, 51·6–70·6) were female, but there were substantial differences in sex shares in lower versus higher skilled positions: more pharmaceutical assistants (75·4%, 51·7–90·4) were female than pharmacists (53·4%, 47·7–59·0); and more dental assistants were female (90·1%, 83·7–93·7) than dentists (49·3%, 44·8–54·1) in 2023 (appendix 2 pp 127–139).

Across GBD super-regions, the density and mix of HRH differed substantially (figure 1, table 1). High-income countries had substantially more health workers overall and depended more on health workers outside of the main cadres, which includes personal care workers as well as specialised cadres such as psychologists and physical therapists. Although high-income countries also had the largest doctor densities per 10 000 population (36·5, 95% UI 34·1–39·4), the doctor density in central Europe, eastern Europe, and central Asia (31·6, 28·3–36·1) was nearly as high, despite much lower HRH densities overall. High-income countries exceeded all other super-regions in their nursing workforce density at 121·8 (111·9–134·2) per 10 000 population, over eight times higher than the density in sub-Saharan Africa (14·5, 11·6–18·1). The largest densities of HRH outside of the main cadres of focus were in high-income countries (313·8, 278·9–349·5), and central Europe, eastern Europe, and central Asia (100·3, 87·9–113·5).

**Figure 2:**
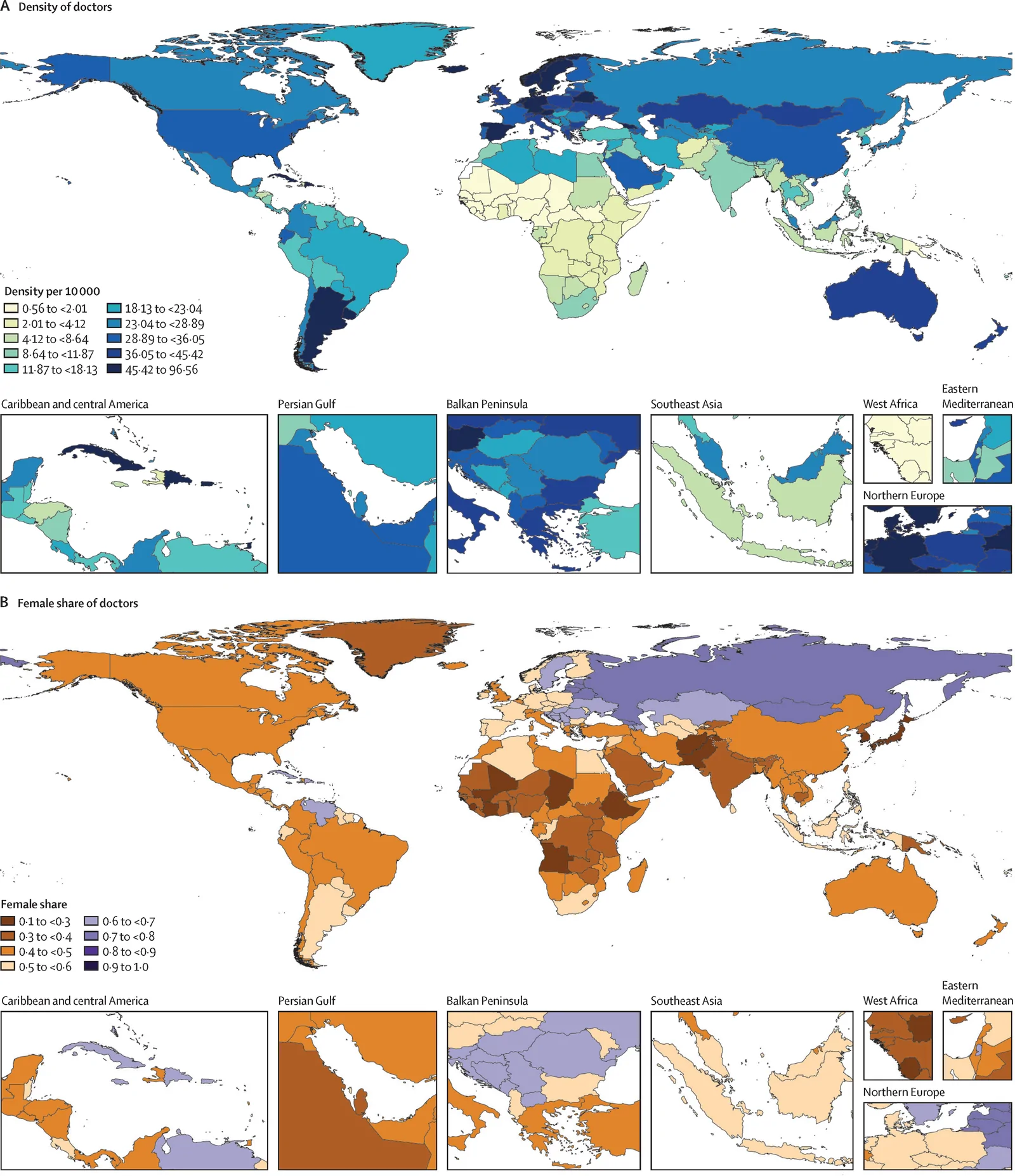

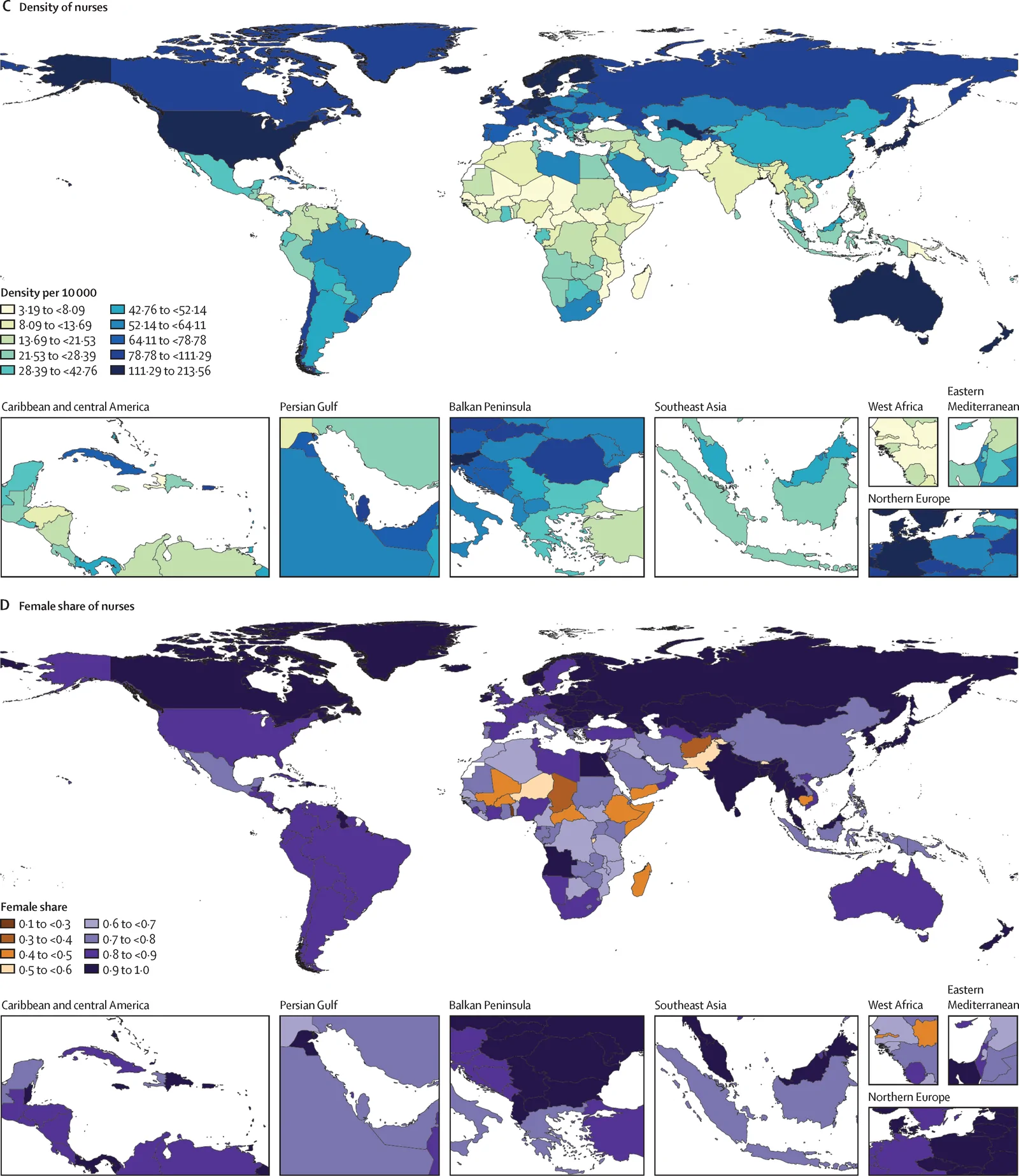
Density and female share of doctors and nurses by country and territory, 2023 (A) Density per 10 000 population of doctors by country and territory. (B) Female share of doctors by country and territory. (C) Density per 10 000 population of nurses by country and territory. (D) Female share of nurses by country and territory. Density is reported in count of workers per 10 000 population.

Across countries, there was a wide range of doctor and nurse densities (figure 2A, C; table 1). Doctor densities were 96·6 (95% UI 80·9–115·2) per 10 000 population in Cuba in 2023, almost 50 times higher than locations with some of the fewest doctors in the world, such as Niger, which had a doctor density of less than two per 10 000 population (figure 2A). Similarly, nurse densities ranged widely (figure 2C). Nurse densities were less than seven per 10 000 population in three countries in sub-Saharan Africa, including Central African Republic (3·4, 1·6–6·8), Madagascar (3·3, 1·4–6·9), and Chad (3·2, 1·6–5·8). In contrast, nurse densities exceeded 110 per 10 000 in high-income locations such as Australia (129·1, 112·0–148·9), Belgium (171·7, 141·7–211·4), South Korea (140·8, 120·1–166·4), the USA (161·4, 136·7–192·7), and Finland (145·3, 113·2–186·8). Taking nurses and doctors together into account, we find a wide range of nurse to doctor ratios, with a 95-percentile range of 0·6–15·1 in 2023 (appendix 2 p 27).

The highest densities of CHWs were concentrated in south Asia (13·6 per 10 000 population, 95% UI 10·5–17·6), southeast Asia, east Asia, and Oceania (13·5, 10·9–16·1), Latin America and the Caribbean (10·8, 9·7–12·2), and sub-Saharan Africa (10·1, 9·0–11·6), with few countries outside of these super-regions relying heavily on this cadre (figure 1, table 1). In 2023, some of the countries with the highest densities of CHWs included Thailand (149·0, 118·5–191·6), Rwanda (34·6, 23·4–47·0), Kenya (18·5, 15·1–22·7), the Philippines (74·9, 52·6–103·6), Democratic Republic of the Congo (54·2, 49·0–59·7), Brazil (21·5, 17·1–26·7), and India (16·2, 12·2–21·4; table 1, appendix 2 p 4). In high-income countries, there was fewer than one CHW per 10 000 people on average.

Notable differences in the sex composition of doctors and nursing personnel were apparent within and across locations. There was a large range in the share of doctors that were female (figure 2B), which exceeded 70% in Russia and other locations in eastern Europe and central Asia. This contrasts with a number of countries in sub-Saharan Africa, such as Ethiopia, Mali, and Chad, as well as in Asia, such as Afghanistan, Pakistan, and Japan, where between 20% and 30% of doctors were female in 2023. The female share was higher among nurses than among doctors in 99·0% of locations in 2023 (figure 1D); the female share was higher among all other HRH cadres in 88·2% of countries in 2023 (appendix 2 p 6). In 2023, 70% or more of nurses were female in 82·3% (95% UI 53·4–94·6) of locations.

**Figure 3:**
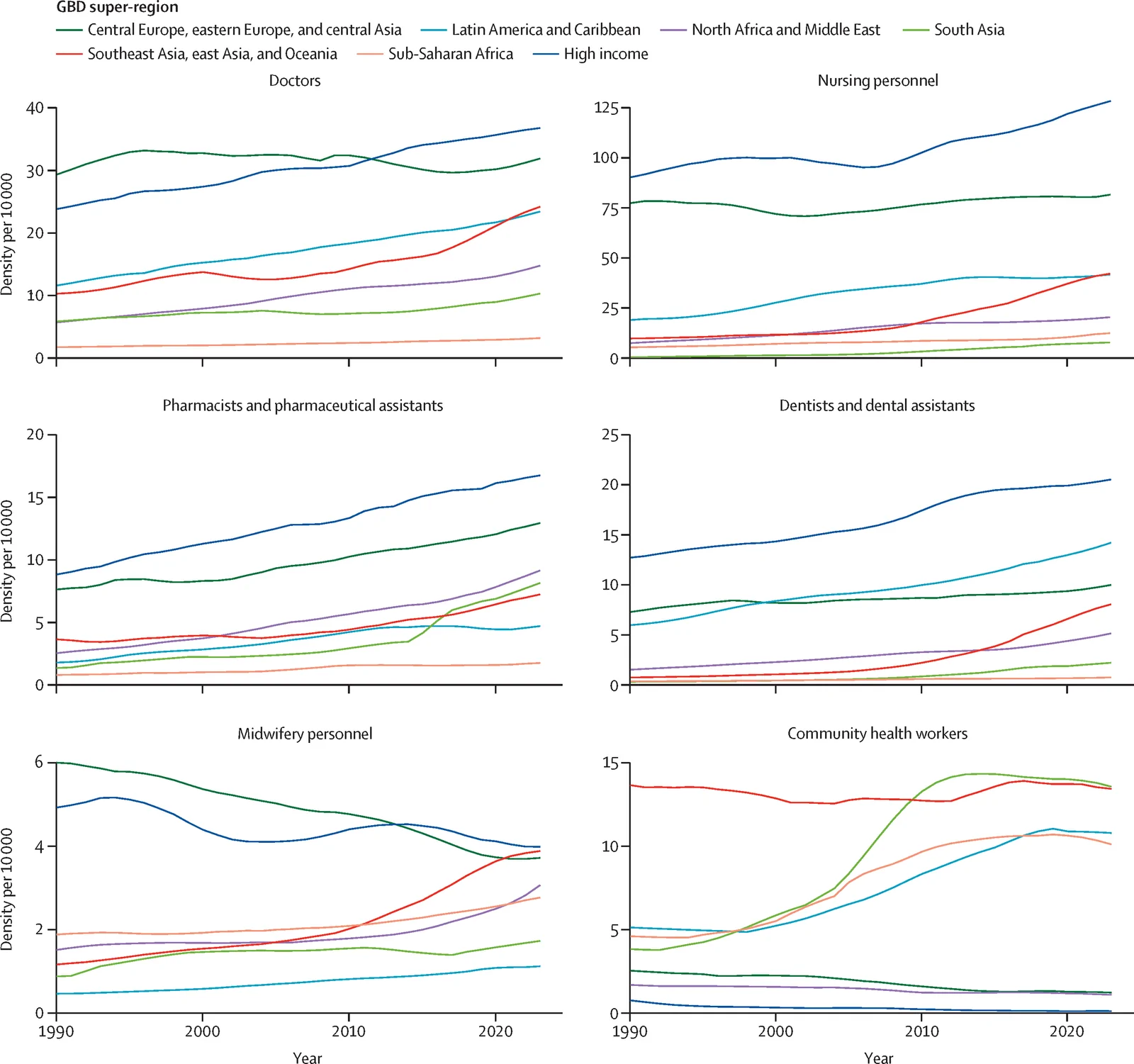
Density of health workers per 10 000 population by cadre and GBD super-region, 1990–2023 Density is reported in count of workers per 10 000 population. GBD=Global Burden of Diseases, Injuries, and Risk Factors Study.

Between 1990 and 2023, 81·2 million (95% UI 72·0–90·5) HRH were added to the health workforce, an increase of 198·5% (161·9–245·6). This included 18·9 million (12·9–24·1) more nurses and 8·7 million (6·6–12·0) more doctors. Patterns of growth differed by cadre (figure 3). In absolute terms, the densities of nurses and doctors increased most across our five cadres of focus, with densities increasing by 6·8 (2·7–11·1) per 10 000 for doctors globally and 14·4 (4·0–22·5) per 10 000 for nurses globally from 1990 to 2023. All super-regions except central Europe, eastern Europe, and central Asia increased their nursing workforce density by 40% or more during this time. Driving up the global doctor workforce size was a large expansion in doctors in China in particular, where the density of doctors increased from 13·2 (8·6–20·2) in 1990 to 31·7 (23·7–41·7) in 2023.

In the remaining cadres of focus trends differed. Dentist and dental assistant densities grew 113·1% (95% UI 36·2–212·5) relative to 1990, followed by pharmacists and pharmaceutical assistants, with growth of 111·2% (57·1–184·2). Consistent growth across super-regions underpinned these increases. CHWs, in contrast, both expanded and contracted, with major increases in sub-Saharan Africa, south Asia, and Latin America and the Caribbean, and declines in the remaining GBD super-regions.

The increased participation of women in the health workforce drove 71·4% (95% UI 64·5–77·4) of the growth in HRH between 1990 and 2023 (appendix 2 p 140), with more women becoming nurses than any other cadre. Across the cadres of focus, only among doctors did the difference in densities between males and females close during this time (appendix 2 p 26).

Globally, the minimum density of doctors associated with a score or 80 or higher on the UHC effective coverage index was 23·8 (95% UI 22·8–24·6) per 10 000 population, while this density was 64·5 (61·2–67·8) for nurses and midwives, 5·2 (4·9–5·5) for dentists, and 5·6 (5·3–6·1) for pharmacists (table 2). For 90 or higher on the UHC score, the minimum densities were higher for doctors (37·1, 36·5–37·6), nurses and midwives (99·9, 96·8–104·7), dentists (8·1, 7·9–8·3), and pharmacists (8·8, 8·4–9·7). Using the thresholds for an 80 or higher UHC score, we estimated a shortage of 34·4 million (31·5–37·4) HRH globally in 2023, including 7·1 million (6·6–7·5) doctors, 23·9 million (21·8–26·1) nurses and midwives, 1·8 million (1·6–1·9) dentists, and 1·6 million (1·4–1·9) pharmacists in 2023 (table 2). Shortages among GBD regions were largest in south Asia, which was short by 2·6 million (2·4–2·8) doctors, 10·0 million (9·3–10·6) nurses and midwives, 0·7 million (0·7–0·8) dentists, and 0·3 million (0·3–0·4) pharmacists. Sub-Saharan Africa also faced major shortages according to our analysis, requiring 2·5 million (2·4–2·6) more doctors, 5·7 million (5·3–6·1) more nurses and midwives, 0·6 million (0·5–0·6) more dentists, and 0·5 million (0·5–0·6) more pharmacists to attain moderate levels of UHC.

**Table 2:** Health worker shortages for four cadre groups at a threshold of 80 out of 100 on the UHC effective coverage index, globally and by GBD super-region, 2023

	**Number of countries with shortage**	**Sum of country-level shortages (number of workers)**
**Doctors (threshold: 23·8 per 10 000 population)**		
Global	121 (120–125)	7 090 000 (6 610 000–7 470 000)
Central Europe, eastern Europe, and central Asia	5 (5–6)	13 400 (10 200–16 300)
High income	2 (2–2)	201 (150–243)
Latin America and Caribbean	19 (19–20)	204 000 (168 000–232 000)
North Africa and Middle East	16 (16–16)	639 000 (581 000–685 000)
South Asia	5 (5–5)	2 620 000 (2 430 000–2 770 000)
Southeast Asia, east Asia, and Oceania	28 (27–30)	1 090 000 (1 020 000–1 150 000)
Sub-Saharan Africa	46 (46–46)	2 530 000 (2 400 000–2 620 000)
**Nurses and midwives (threshold: 64·5 per 10 000 population)**		
Global	132 (131–137)	23 900 000 (21 800 000–26 100 000)
Central Europe, eastern Europe, and central Asia	13 (13–16)	96 000 (62 500–132 000)
High income	2 (2–3)	105 000 (85 700–124 000)
Latin America and Caribbean	26 (25–26)	1 310 000 (1 120 000–1 500 000)
North Africa and Middle East	17 (17–18)	2 530 000 (2 340 000–2 740 000)
South Asia	5 (5–5)	9 960 000 (9 340 000–10 600 000)
Southeast Asia, east Asia, and Oceania	23 (23–23)	4 230 000 (3 510 000–4 950 000)
Sub-Saharan Africa	46 (46–46)	5 710 000 (5 310 000–6 110 000)
**Dentists (threshold: 5·2 per 10 000 population)**		
Global	119 (118–121)	1 760 000 (1 620 000–1 860 000)
Central Europe, eastern Europe, and central Asia	11 (11–11)	32 800 (29 500–35 200)
High income	1 (1–1)	120 (106–130)
Latin America and Caribbean	18 (18–19)	28 000 (24 900–30 500)
North Africa and Middle East	11 (10–12)	94 000 (83 000–104 000)
South Asia	5 (5–5)	745 000 (688 000–787 000)
Southeast Asia, east Asia, and Oceania	27 (27–27)	272 000 (250 000–289 000)
Sub-Saharan Africa	46 (46–46)	585 000 (549 000–612 000)
**Pharmacists (threshold: 5·6 per 10 000 population)**		
Global	123 (121–130)	1 640 000 (1 420 000–1 930 000)
Central Europe, eastern Europe, and central Asia	10 (10–11)	39 200 (35 500–44 100)
High income	3 (3–4)	7 660 (6 860–9 140)
Latin America and Caribbean	21 (20–23)	214 000 (195 000–240 000)
North Africa and Middle East	10 (9–11)	103 000 (89 500–121 000)
South Asia	5 (5–5)	338 000 (272 000–425 000)
Southeast Asia, east Asia, and Oceania	29 (29–30)	392 000 (318 000–491 000)
Sub-Saharan Africa	45 (45–46)	549 000 (508 000–604 000)

Data in parentheses are 95% uncertainty intervals. Estimates for shortages are based on the frontier shown in appendix 2 (p 141) and correspond to the minimum level of each HRH cadre required to attain a score of 80 or higher on the UHC effective coverage index. Minimum threshold estimation is focused on the four cadres in Sustainable Development Goal target 3.c.1. GBD=Global Burden of Diseases, Injuries, and Risk Factors Study. HRH=human resources for health. UHC=universal health coverage.

## Discussion

This study assessed the supply and sex composition of 20 health worker cadres for 204 countries and territories between 1990 and 2023. We found that many more health workers are female than male and that cadre mix differed substantially by sex. Of the 81·2 million increase in total HRH between 1990 and 2023, 71·4% was driven by women formally joining the health workforce. However, this growth was disproportionately concentrated in cadres in which women have historically faced limited pathways into clinical leadership and senior management positions. Even with these increases, the global health workforce was short by 34·4 million workers in 2023.

Our findings emphasised how the cadre composition of the health workforce varies greatly, with large differences in the densities of doctors, nurses, CHWs, and other cadres across countries. This reflects different historical investment patterns, training system capacities, policy preferences (eg, reliance on community-based *vs* facility-based care), planned investment strategies, and the historical context out of which health systems have evolved. Future investigation into how cadre mix connects to the organisation, efficiency, and effectiveness of health systems will be useful for workforce planning efforts.

Our study provides the first global estimates of CHWs. In south Asia, Latin America and the Caribbean, southeast Asia, and sub-Saharan Africa, CHWs are much more prominent than in high-income settings. This cadre has one of the most diverse sets of training, remuneration arrangements, working conditions, and responsibilities and thus densities should be interpreted alongside understanding of those dimensions in a particular setting. High CHW densities can represent prioritisation of primary health-care delivery but could also be a marker of system-level shortfalls, where CHWs serve as low-cost labour substituting for more skilled workers.^24^ Future analyses should investigate the extent to which expansion of the CHW workforce represents underinvestment in other HRH cadres versus concerted efforts to strengthen primary care.

Our estimates of HRH expansion since 1990 can be linked with specific reforms enabling rapid workforce growth. In China, for instance, the number of doctors more than doubled between 1990 and 2023, driven by reforms that quadrupled government investment in health care, expanded insurance coverage, and changed the organisation and financing of the health system.^25^ A large expansion in the health workforce also occurred in Bolivia, where the Single Health System model increased government health expenditure along with strengthening primary care.^26^ Türkiye's Health Transformation Program, launched in 2003, also entailed important increases in HRH as part of large increases in investments in the health sector.^27^ In common across these countries was political and financial commitment to increase HRH, embedded in broader reforms to pursue UHC. Countries with persistent HRH gaps could consider similarly coupled reforms.

Increases in women's formal participation in the health workforce contributed to growth in HRH supply and are likely critical to future HRH expansions.^28^ Although our data enabled us only to consider sex, not gender, we expand our discussion to the gendered social, institutional, and structural processes that explain differences in the female proportion of health workers across cadres and time. Plausible drivers include rising female educational attainment in many countries, which expands the pool of women qualified for health worker roles. Other possible explanations include occupational segregation that channels women towards caregiving professions. Occupational segregation in the health-care sector is longstanding and persistent: women predominate in nursing, midwifery, and CHW cadres, but are under-represented among doctors.^7^, ^8^ Within-cadre progression to leadership is also an issue: even in cadres where women predominate as workers, men are represented in the majority of senior management and clinical leadership roles.^5^ Barriers to female leadership deprive health systems of talent and can lead to workforce strategies that fail to consider the challenges faced by the largely female workforce.^29^ Low remuneration of some health worker cadres, such as CHWs, reinforces the devaluation of women's time and contribution to health systems. Gender discrimination and inequality in the health workforce have been linked to lower morale, reduced productivity, absenteeism, maldistribution of workers, and attrition, all of which can contribute to HRH shortfalls and lower levels of productivity.^5^, ^6^

Our estimates of workforce gaps showed that further expansion of the health workforce is required. We estimated need for an additional 34·4 million health workers, contrasting with a WHO estimate of need for 15·4 million health workers, including the four cadres we consider along with other HRH occupations.^30^ Another recent approach estimated a 100 million cancer workforce shortage.^31^ We note that the WHO approach considers health workforce needs relative to median density levels. Our methodology uses a UHC effective coverage index threshold of 80 and a frontier approach, considering minimum densities, beyond which more workers might be needed. The lower level of aspiration used by WHO might be more appropriate for immediate workforce planning, whereas our higher level of aspiration might be more appropriate for more ambitious, long-term goals. Nonetheless, both estimates are aligned in identifying that nursing shortages account for the largest share of global HRH need.

WHO's *Global Strategy on Human Resources for Health: Workforce 2030* emphasised an array of strategies for bolstering the size and effectiveness of the health workforce.^32^ This included expanding domestic training capabilities and ensuring decent work conditions, among other recommendations. Investments in expanding medical school enrolment, including in sub-Saharan Africa and south Asia, have potential to produce HRH for regions with the most need.^33^, ^34^ However, rapid workforce expansion could generate societal challenges. In South Korea, for example, the doubling of medical school quotas in 2022 triggered widespread doctor strikes.^35^ Digital transformation and artificial intelligence could enable more efficient use of specialty care, including for rural areas.^36^ Careful consideration of remuneration as fair and to attract talent is also crucial. Health systems should strive to ensure health workers previously paid through development assistance channels are not lost to emigration to higher-income settings to attain higher remuneration.^1^, ^37^

Planners are advised to consider institutional policies and practices through a gender lens.^3^, ^5^, ^38^ Formal mentorship and sponsorship programmes, leadership development training, transparent promotion criteria, and gender-responsive leadership initiatives are all candidate strategies for addressing the within-cadre gender leadership gap.^29^ Addressing workplace violence and sexual harassment along with formal reporting systems for harassment and assault are also critical.^6^, ^39^ Flexible work arrangements, universal provision of paid maternity and caregiving leave,^40^, ^41^ and caregiving arrangement policies that do not penalise career interruption^4^, ^5^, ^42^ are all policies that could be considered to bolster HRH capacity by better supporting the female health workforce. Dismantling and refashioning stereotypes surrounding the health workforce to recruit male workers could also be considered.^43^ Gender-neutral job advertisements and systems for peer support, mentorship, and sponsorship all represent ideas for addressing barriers related to socialisation and stereotypes.^4^, ^29^, ^39^

This study has several limitations. First, because of data constraints, this study relies on a binary sex categorisation as an approximation of the broader conceptualisation of gender. Second, availability of data was concentrated in high-income countries and among the doctor and nurse cadres (see appendix 1 pp 12–16). Third, although internationally recognised and standardised occupation coding systems were used, we note that none of these worker cadres are uniform in their duties globally, with CHW duties differing dramatically along with major variation in tasks done by nurses, pharmaceutical assistants, and dental assistants. Yet, this is the most widely recognised system available and the only system that can be fully operationalised for examining the health workforce. Wherever possible, we focused only on CHWs who were paid in some form, including honoraria and stipends, consistent with our definition of employment. However, it was not possible to verify in tabulated sources whether both volunteer and paid CHWs were included, potentially inflating our estimates. Fifth, our study period covered the COVID-19 pandemic, which might have disrupted some HRH data systems.^44^, ^45^ Sixth, our assessment of shortages was limited to the cadres included in the SDGs, with thresholds representing minimum indicative levels or cadre-level floors, not normative targets. Our shortage estimates are aspirational and not additive: the minimum density observed in countries attaining the UHC benchmark might have a high density in a complementary cadre. Attaining all four cadre-specific thresholds simultaneously is neither strictly necessary nor strictly sufficient. Furthermore, countries that already achieved the UHC benchmark should not automatically be interpreted as under-resourced; unmet demand driven by changing health needs and patient expectations could result in need for additional HRH. UHC is also challenging to measure and our analysis is missing important determinants of UHC, such as social and economic development, financing, governance, benefit design, and infrastructure. Seventh, density is widely reported in our study but does not capture skill mix, productivity, distribution within countries, and other factors, such that countries with the same density can face very different workforce constraints. Eighth, because of estimation and adjustment, density estimates, including estimates of uncertainty, can differ from statistics reported by ministries of health and other sources. Some labour force surveys have small sample sizes that introduce noise, but we addressed this through inverse-variance-based weighting in ST-GPR. Some labour force surveys and censuses use national classifications mapped to ISCO, which could lead to misclassification. Finally, we omit key characteristics of the health workforce. Age will be important to assess in the future, since generational effects and workers ageing out are critical factors. Better estimating professional specialties in the future is also important for assessing efficiency, training, and allocation of HRH. Estimating sector (eg, private, public), remuneration levels, emigration status, and full-time versus part-time employment status are crucial future directions as well.

In conclusion, the world faces substantial shortfalls in HRH. For many settings where HRH data remain incomplete or inconsistent, these estimates offer a strategic foundation for countries to benchmark progress against peers, identify structural shortages, understand gender imbalances, and assess alignment between workforce composition and UHC attainment. By integrating gender and cadre mix assessments into planning and policies, countries can strengthen HRH in pursuit of UHC and confront the many health challenges of this century.

### GBD 2023 Human Resources for Health Collaborators

### Affiliations

### Contributors

### Data sharing

This study follows the Guidelines for Accurate and Transparent Health Estimates Reporting (GATHER). Data used in these analyses are available on Global Health Data Exchange (GHDx) tool at https://ghdx.healthdata.org/gbd-2023.

## Declaration of interests
