# Supplementary material for: Measuring the composition and availability of human resources for health by sex in 204 countries and territories, 1990–2023, and workforce gaps for universal health coverage: a systematic analysis for the Global Burden of Disease Study 2023
Source: Lancet Public Health. 2026 Aug 18;11(9):e604–29. doi: 10.1016/S2468-2667(26)00145-3 (PMC13501144; doi:10.1016/S2468-2667(26)00145-3)
Supplement: Supplementary file 1 [file mmc1.pdf]

THE LANCET
Public Health

Supplementary appendix 1

This appendix formed part of the original submission and has been peer reviewed. We post it as supplied by the authors.

Supplement to: GBD 2023 Human Resources for Health Collaborators. Measuring the composition and availability of human resources for health by sex in 204 countries and territories, 1990–2023, and workforce gaps for universal health coverage: a systematic analysis for the Global Burden of Disease Study 2023. *Lancet Public Health* 2026; **11:** 604–29.

Appendix 1: methods appendix to "Measuring the composition and availability of human resources for health by sex in 204 countries, 1990–2023, and workforce gaps for universal health coverage: a systematic analysis for the Global Burden of Disease Study 2023"

This appendix provides further methodological detail for "Measuring the composition and availability of human resources for health by sex in 204 countries, 1990–2023, and workforce gaps for universal health coverage: a systematic analysis for the Global Burden of Disease Study 2023."

Table of Contents

Preamble 3

List of appendix figures and tables 4

GATHER statement...5

Methods 7

Section 1. Overview..7

Section 2. Estimating sex-specific human resources for health densities..7

2.1 Defining health worker cadres..7

2.2 Input data and indicators..9

2.3 Mapping and splitting...23

2.4 Input data adjustment strategy 29

2.5 Modelling strategy 39

Section 3. Improvements from GBD 2019 45

Section 4. Estimating the relationship between health worker densities and universal health coverage[2]......52

4.1 Previous thresholds 52

4.2 Universal health coverage (UHC) effective coverage index 52

4.3 Stochastic frontier meta-analysis (SFM)...55

Section 5. Online tools and glossary of terms 58

Section 6. References 59

Preamble

This appendix provides methodological detail for estimating sex-specific health worker densities and minimum health worker density thresholds needed to achieve universal health coverage. The study complies with the Guidelines for Accurate and Transparent Health Estimates Reporting (GATHER) recommendations.[1] It includes information on objectives, funding, data sources, modelling, and presentation of results in an effort to maximise transparency in our estimation processes and provide a comprehensive description of analytical steps. We intend this appendix to be a living document, to be updated with each iteration of the Global Burden of Disease Study.

Portions of this appendix have been reproduced or adapted from appendix 1 of the published study from GBD 2019 Human Resources for Health Collaborators and other publications from GBD 2023.[2–6] References are provided for reproduced or adapted sections.

List of appendix figures and tables

Appendix 1 Table 1: GATHER Statement

Appendix 1 Figure 1: Flowchart of estimation process

Appendix 1 Table 2: Health worker cadres and the corresponding four-digit occupation codes from ISCO-88 and ISCO-08

Appendix 1 Figure 2a-i: Country-years of health worker density data by cadre, 1990–2023

Appendix 1 Figure 3: Country-years by data type and measure for all cadres, 1990–2023

Appendix 1 Figure 4a-i: Country-years of health worker sex share data by cadre, 1990–2023

Appendix 1 Figure 5: Country-years of input data by occupation coding system

Appendix 1 Table 3: Health worker cadre mappings across most granular available ISCO versions

Appendix 1 Table 4: List of three-digit and four-digit occupation codes used for splitting and corresponding health worker cadre mappings

Appendix 1 Table 5: Three-digit occupation codes that describe the health workforce by occupation coding system

Appendix 1 Table 6: Number of matched pairs by cadre

Appendix 1 Table 7: Lambdas and RMSE from the lasso covariate selection regressions, by cadre

Appendix 1 Figure 6a-g: Adjustment type for administrative densities from WHO NHWA and Eurostat Health Database by cadre

Appendix 1 Figure 7a-g: Average percentage difference adjustment to administrative data from WHO NHWA and Eurostat Health Database by cadre

Appendix 1 Table 8: Covariates for ST-GPR models

Appendix 1 Table 9: RMSE by ST-GPR modelling stage and cadre

Appendix 1 Figure 8a–f: Average modelled health worker density estimate minus input data, by cadre

Appendix 1 Figure 9a-e: Comparison of health worker densities for select cadres from GBD 2019 and GBD 2023 in 1990, 2000, 2010, and 2019

Appendix 1 Table 10: List of abbreviations

GATHER statement

This study complies with the Guidelines for Accurate and Transparent Health Estimates Reporting (GATHER) recommendations. We have described our data sources and documented the steps involved in our analysis. Details on GATHER reporting are depicted in appendix table 1.

Appendix 1 Table 1: GATHER checklist

Item #	Checklist item	Reporting location
**Objectives and funding**		
1	Define the indicator(s), populations (including age, sex, and geographic entities), and time period(s) for which estimates were made.	Main text (Methods – Definitions); Appendix 1 (Sections 2.1 – 2.2)
2	List the funding sources for the work.	Main text (Summary – Funding section; Methods – Role of the funding source section)
**Data Inputs**		
For all data inputs from multiple sources that are synthesized as part of the study:		
3	Describe how the data were identified and how the data were accessed.	Main text (Methods - Data sources); Appendix 1 (Section 2.2)
4	Specify the inclusion and exclusion criteria. Identify all ad-hoc exclusions.	Main text (Methods - Data sources); Appendix 1 (Section 2.2)
5	Provide information on all included data sources and their main characteristics. For each data source used, report reference information or contact name/institution, population represented, data collection method, year(s) of data collection, sex and age range, diagnostic criteria or measurement method, and sample size, as relevant.	Online data citation tool: https://ghdx.healthdata.org/gbd-2023/sources
6	Identify and describe any categories of input data that have potentially important biases (e.g., based on characteristics listed in item 5).	Main text (Methods – Adjusting administrative sources); Appendix 1 (Section 2.4)
For data inputs that contribute to the analysis but were not synthesized as part of the study:		
7	Describe and give sources for any other data inputs.	Online data citation tool: https://ghdx.healthdata.org/gbd-2023/sources
For all data inputs:		
8	Provide all data inputs in a file format from which data can be efficiently extracted (e.g., a spreadsheet rather than a PDF), including all relevant meta-data listed in item 5. For any data inputs that cannot be shared because of ethical or legal reasons, such as third-party ownership, provide a contact name or the name of the institution that retains the right to the data.	Online data citation tool: https://ghdx.healthdata.org/gbd-2023/ sources. Availability of data is dependent on data use agreements; contact information provided when not directly available.
**Data analysis**		
9	Provide a conceptual overview of the data analysis method. A diagram may be helpful.	Appendix 1 (Section 2.1)
10	Provide a detailed description of all steps of the analysis, including mathematical formulae. This description should cover, as relevant, data cleaning, data pre-processing, data adjustments and weighting of data sources, and mathematical or statistical model(s).	Main text (Methods); Appendix 1 (Sections 2 - 4)
11	Describe how candidate models were evaluated and how the final model(s) were selected.	Main text (Methods); Appendix 1 (Section 2.5)
12	Provide the results of an evaluation of model performance, if done, as well as the results of any relevant sensitivity analysis.	Appendix 1 (Section 2.5)
13	Describe methods for calculating uncertainty of the estimates. State which sources of uncertainty were, and were not, accounted for in the uncertainty analysis.	Main text (Methods – Estimating sex-specific health worker densities); Appendix 1 (Section 2.5)
14	State how analytic or statistical source code used to generate estimates can be accessed.	The statistical code used for data processing and analysis is available upon request.
**Results and discussion**		
15	Provide published estimates in a file format from which data can be efficiently extracted.	Online data tools will be updated at time of publication and available at this link: https://ghdx.healthdata.org/gbd-2023

16	Report a quantitative measure of the uncertainty of the estimates (e.g. uncertainty intervals).	Main text (Abstract, Results, Discussion)
17	Interpret results in light of existing evidence. If updating a previous set of estimates, describe the reasons for changes in estimates.	Main text (Abstract, Results, Discussion)
18	Discuss limitations of the estimates. Include a discussion of any modelling assumptions or data limitations that affect interpretation of the estimates.	Main text (Methods – Estimating sex-specific health worker densities, Estimating health worker shortages; Discussion)

Methods

Section 1. Overview

Many of the methods described below were reported in greater detail in an earlier study estimating human resources for health (HRH), universal health coverage (UHC), and previous Global Burden of Diseases, Injuries, and Risk Factors Study (GBD) capstone publications.[2–6] Analyses and figures were generated with R (version 4.4.2), Python (version 3.12.3), or Stata (version 17.0).

Section 2. Estimating sex-specific human resources for health densities

Appendix 1 Figure 1: Flowchart of estimation process

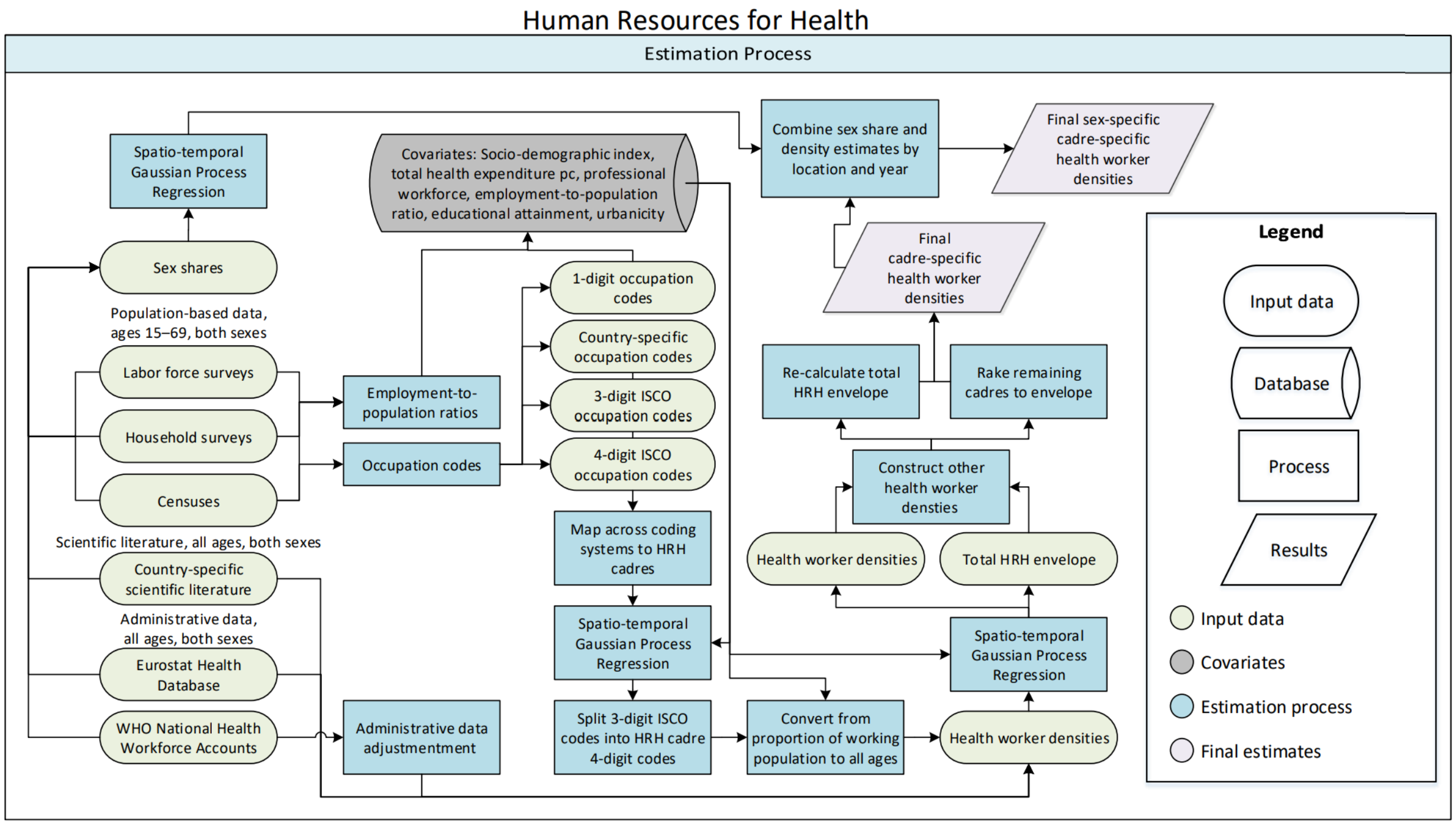

2.1 Defining health worker cadres

This study uses the definition of employment from the International Labor Organizations (ILO) to describe occupations among those who are actively participating in the labor force.[7] We rely on the International Standard Classification of Occupations (ISCO) to define active health workers. ISCO hierarchically organises occupations according to a set of skill levels, tasks, and educational requirements with occupation codes. There are multiple ISCO versions that were developed over time to reflect modern labour markets. This analysis incorporates all ISCO versions, released in 1958 (ISCO-58), 1968 (ISCO-68), 1988 (ISCO-88), and 2008 (ISCO-08).

Codes range in granularity from 1 to 4 digits, where additional digits indicate more specific occupations. For example, a 1-digit code (2) refers to professionals overall, a 2-digit code (22)

represents health professionals broadly, a 3-digit code (222) specifies nursing and midwifery professionals, and a 4-digit code (2222) identifies midwifery professionals. ISCO does not inherently distinguish occupations based on licensure; however, our definition of health workers precludes the inclusion of informal practitioners.

In our study, health worker cadres are defined using the most recent and granular ISCO version, four-digit ISCO-08 codes, due to higher levels of precision and alignment with available data (further described in section 2.3). Cadre groupings are consistent with the guidance on classifying health workers provided from the World Health Organization (WHO).[8,9] The included cadres and their corresponding occupation codes are listed in table 2.[2] Table 2 underscores that using the latest ISCO version allows for a more precise definition of health workers, enabling separate identification of nursing and midwifery personnel and distinct categorisation of community health workers. While the main manuscript focuses on cadres emphasised in Sustainable Development Goal (SDG) 3.c, namely doctors, nurses, midwives, dentists, and pharmacists, the envelope of all health workers in our study encompasses 20 distinct cadres.[10]

Because the distinction between nursing and midwifery personnel in ISCO-08 is based on the tasks performed rather than on formal qualifications or regulatory registration, we operationalize occupation code to disentangle these two cadres where possible. We recognise, however, that a separately constituted midwifery cadre does not exist in all settings. We therefore identified 15 locations in which the WHO National Health Workforce Accounts reported nursing personnel but no midwifery personnel, and reviewed external sources for evidence of a distinct midwifery workforce. In eight of these locations we identified corroborating sources describing midwives; for the seven remaining locations we found no such evidence and re-modelled the nursing and midwifery cadres accordingly, so that a midwifery workforce is not asserted where the available data do not support one.

Appendix 1 Table 2: Health worker cadres and the corresponding four-digit occupation codes from ISCO-88 and ISCO-08

Health worker cadre	4-digit ISCO-88 code	4-digit ISCO-08 code
Doctors	2221	2211, 2212
Nursing personnel	2230, 3231, 3232	2221, 3221
Midwifery personnel		2222, 3222
Pharmaceutical personnel		
Pharmacists	2224	2262
Pharmaceutical assistant	3228	3213
Dental personnel		
Dentists	2222	2261
Dental assistants	3225	3251
Other health workers		
Community health workers	3221	3253
Clinical officers and medical assistants		2240, 3256
Physiotherapists and prosthetic technicians	3226	2264, 3255, 2269
Medical imaging and therapeutic equipment technicians	3133	3211
Medical laboratory technicians	3211	3141, 3212
Health-care aides and ambulance workers	5139, 5132	3258, 5321
Environmental health workers	3222	2263
Optometrists and opticians	3224	2267, 3254
Dieticians and nutritionists	3223	2265
Audiologists, speech therapists, and counsellors	3229	2266, 3259
Psychologists	2445	2634
Personal care workers	5133	5329, 5322
Traditional and complementary practitioners	3241	2230, 3230

2.2 Input data and indicators

2.2.1 Health worker densities

We leveraged three types of data sources to estimate health worker densities: administrative data, population-based data, and scientific literature. These sources met our inclusion criteria regarding population coverage and the ability to map occupations to a health worker cadre of interest. The first criterion requires that the source is nationally representative of the working-age population for both sexes. In this study, working ages are defined as 15 to 69, which is the typical age range covered by national labour force surveys. The second criterion asserts that the source collects information on respondents' current, main occupations to a level of granularity that could identify health workers directly or identify a small aggregate group from which health worker cadres could be accurately disaggregated. This level of granularity corresponds with any ISCO version at 3 or 4 digits of granularity or a custom coding system where at least one occupation is an approximate match to an ISCO HRH-related code. This requirement eliminated

many eligible sources, since most standard international surveys and censuses do not code occupations to the level of detail required to identify health workers in total or specific cadres.

The population-based data sources we drew upon for this work include nationally representative cross-sectional surveys and censuses conducted between 1990 and 2023 with available individual-level data. All published sources were identified through the Global Health Data Exchange (GHDx) using occupation-related keyword searches (eg, "Occupations," "Occupational risk factors", "Occupational injuries"). To download information on the data used in these analyses, please visit the Global Health Data Exchange (GHDx) tool at https://ghdx.healthdata.org/gbd-2023. In total, 1,782 microdata sources with employment and occupation data were identified and extracted through the GHDx, 82 of which were censuses and 1,700 of which were surveys. Considering the country-years represented by those sources, there are in total 1,898 country-years of censuses and population-based surveys used in this study.

From these microdata sources, we reviewed questions describing active employment and occupation. Typical survey questions for identifying active employment asked individuals if they had worked for at least one hour in the previous seven days in any of the following capacities: for a wage, as an apprentice, in self-employment, or for a family business.[7] Those reporting only a temporary absence from such a position in the preceding seven days were also considered to be employed. Among employed individuals, occupation distributions were determined from a respondent's self-reported description of their main job in the previous seven days, or their typical main job if they were temporarily absent from work. From this sequence of questions, we extracted three indicators: densities of HRH-related workers among working-age populations, employment-to-population ratios among working-age populations, and the distribution of workers across broad occupation types, referred to as ISCO major groups, among employed populations. The first indicator is the primary measure for this study. The latter indicators are supplemented with tabulated data resources from the ILO and modelled using methods described in Section 2.5. Because all indicators from population-based data sources were extracted among work-age populations (ages 15-69), these data were converted to an all-age population denominator using previously published population distributions across age, location, and year.[6]

In addition to the survey and census data, we included administrative data from WHO's National Health Workforce Accounts (NHWA) database and the Eurostat Health Database.11,12 NHWA data are formally reported by nationally nominated focal points from ministries of health, with data stemming directly from national Health Resources and Health Information Systems (HRHIS) for the large majority of reporting countries.13 Eurostat health workforce data are similarly collected via nationally designated focal points through the Joint Questionnaire on Non-Monetary Health Care Statistics, with data mainly based on national administrative sources and a small number of countries supplementing with labor force surveys.[12] Based on documentation from WHO and Eurostat, the databases report practicing health worker data with sufficient granularity (four-digit ISCO-08) to meet our inclusion criteria. Eurostat reports age-specific counts for each cadre, allowing for the extraction of the target working-age population.

The NHWA covers all age groups; therefore, this criterion is relaxed for the inclusion of those sources to address data gaps. Ultimately, we included 3,888 country-years of administrative data describing eight cadres in our study: doctors, nurses, midwives, pharmaceutical personnel, dental personnel, and community health workers.

To ensure sufficient data coverage in countries with large populations (>150 million population), we conducted a targeted search for scientific literature from these locations when we had insufficient data from the other sources. From this search, we identified 94 country-years of data from scientific literature and reports like India's Accredited Social Health Activist (ASHA) annual report and China's Statistical Yearbooks.[14,15] We thoroughly assessed these sources to ensure alignment of job titles, definitions, and tasks with ISCO-08's health worker criteria. As these reports do not specify age coverage, this criterion was relaxed to allow their inclusion. Figures 2a–2i summarise by cadre the total number of country-years from all data sources employed in this study.

Appendix 1 Figure 2: Country-years of health worker density data by cadre, 1990–2023

Appendix 1 Figure 2a: Country-years of doctor density data, 1990–2023

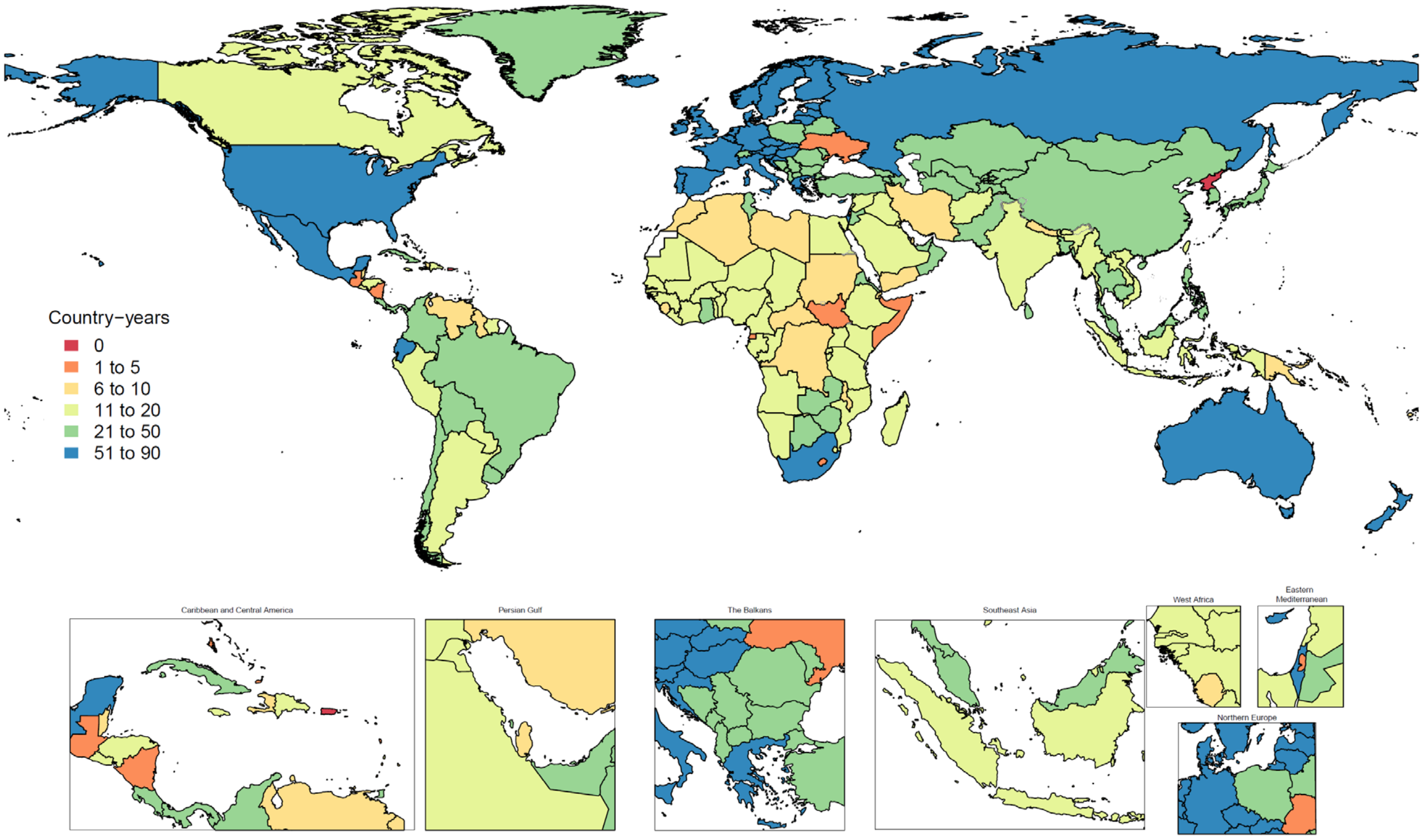

Appendix 1 Figure 2b: Country-years of nursing personnel density data, 1990–2023

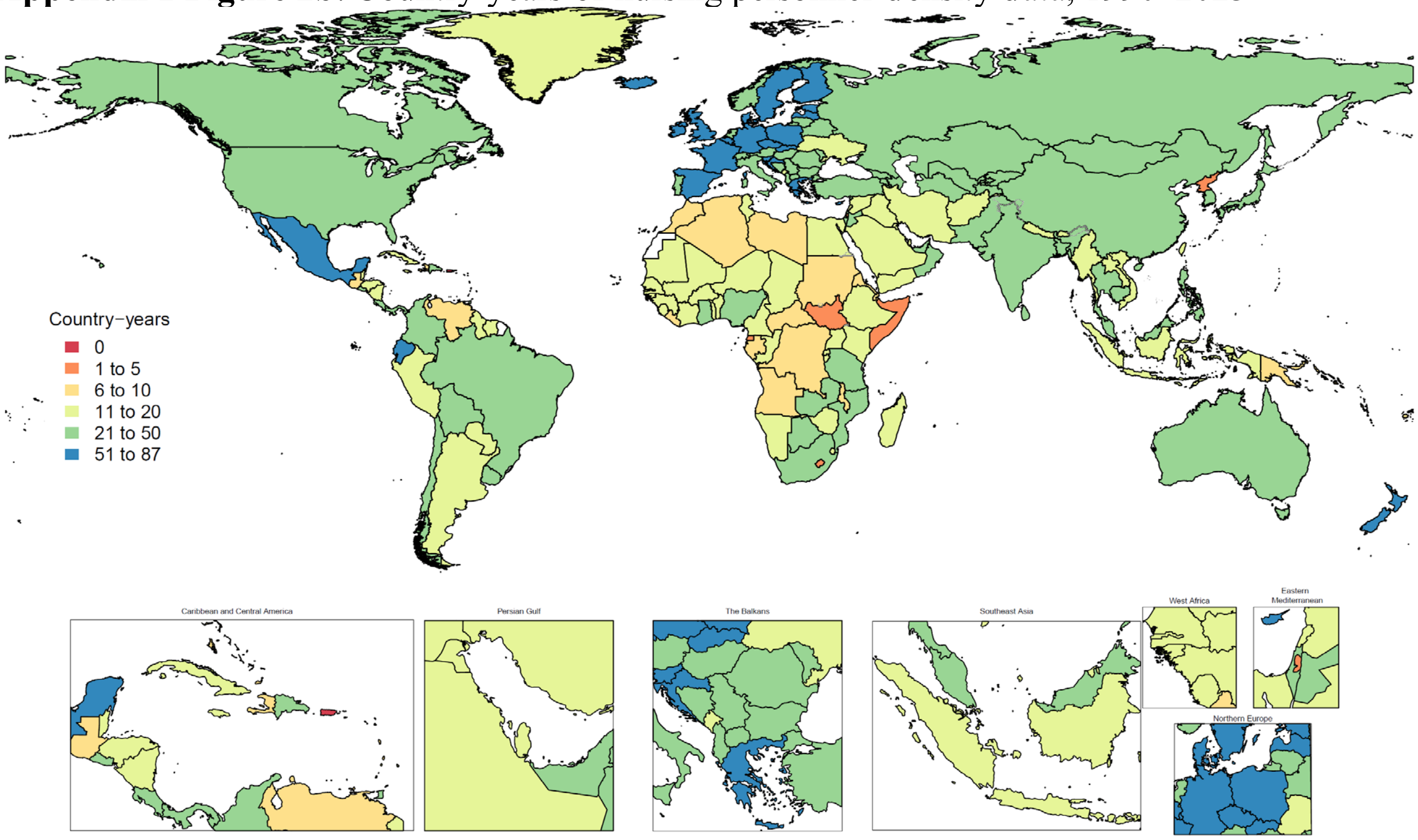

Appendix 1 Figure 2c: Country-years of midwifery personnel density data, 1990–2023

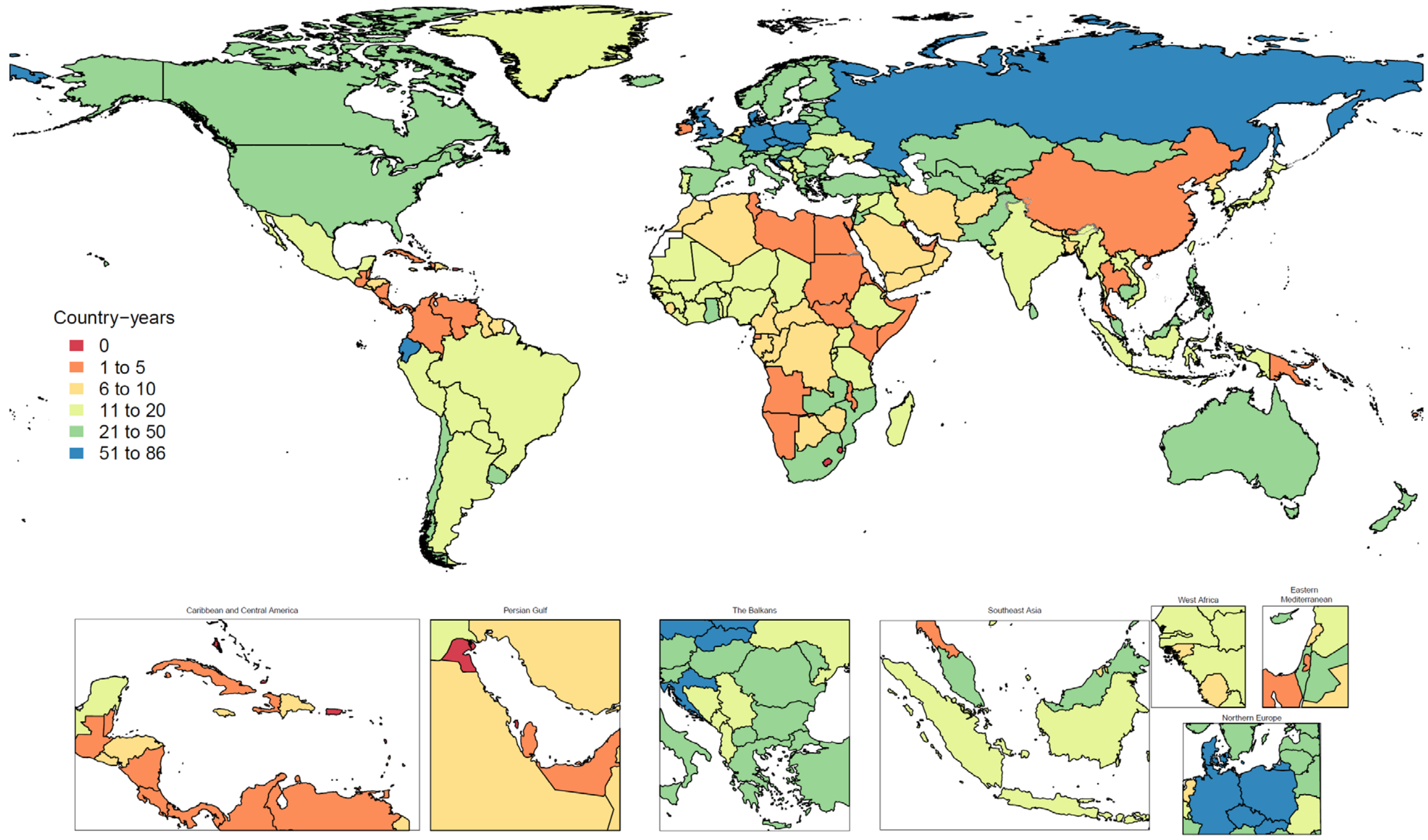

Appendix 1 Figure 2d: Country-years of dentist density data, 1990–2023

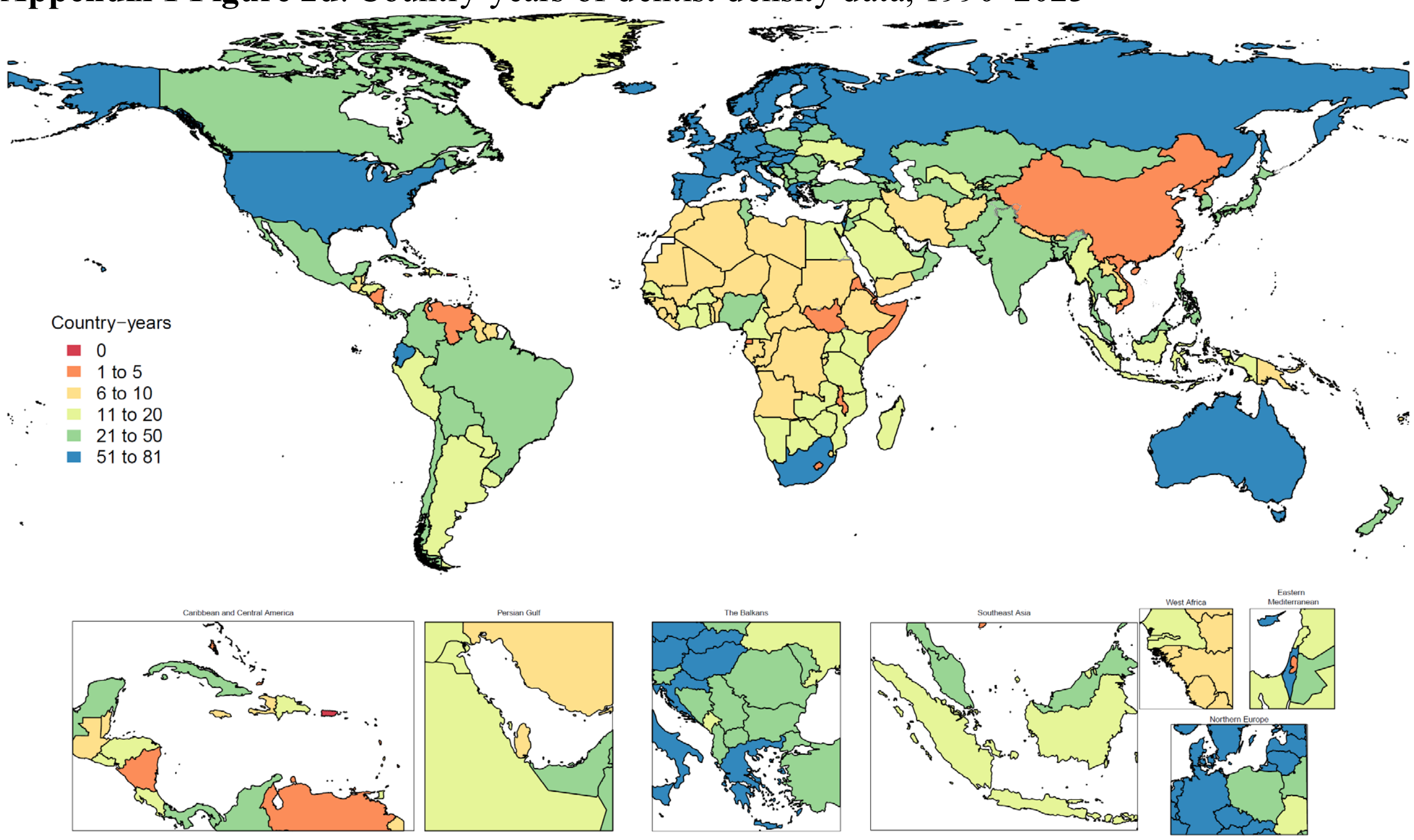

Appendix 1 Figure 2e: Country-years of dental assistant density data, 1990–2023

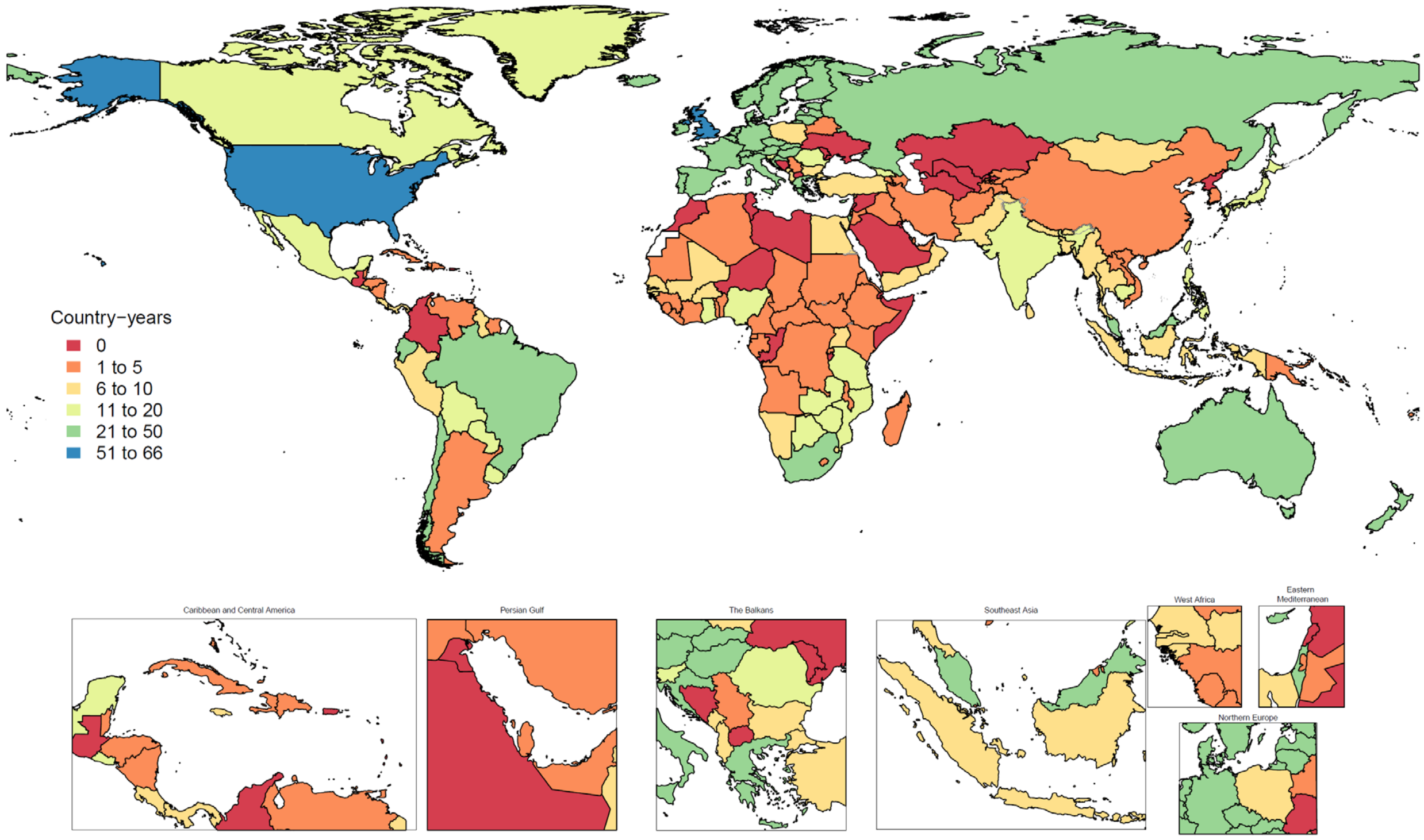

Appendix 1 Figure 2f: Country-years of pharmacist density data, 1990–2023

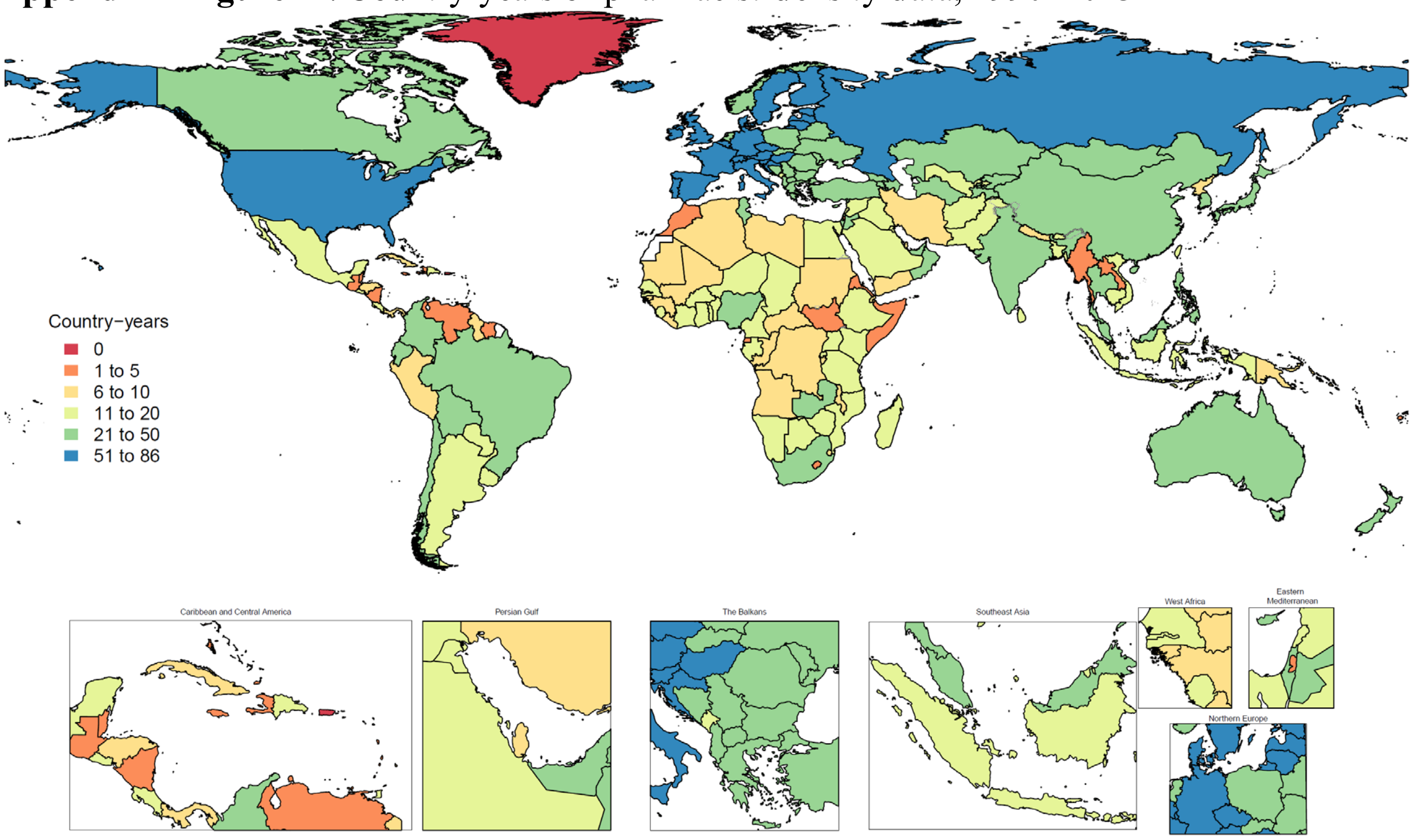

Appendix 1 Figure 2g: Country-years of pharmaceutical assistant and assistant density data, 1990–2023

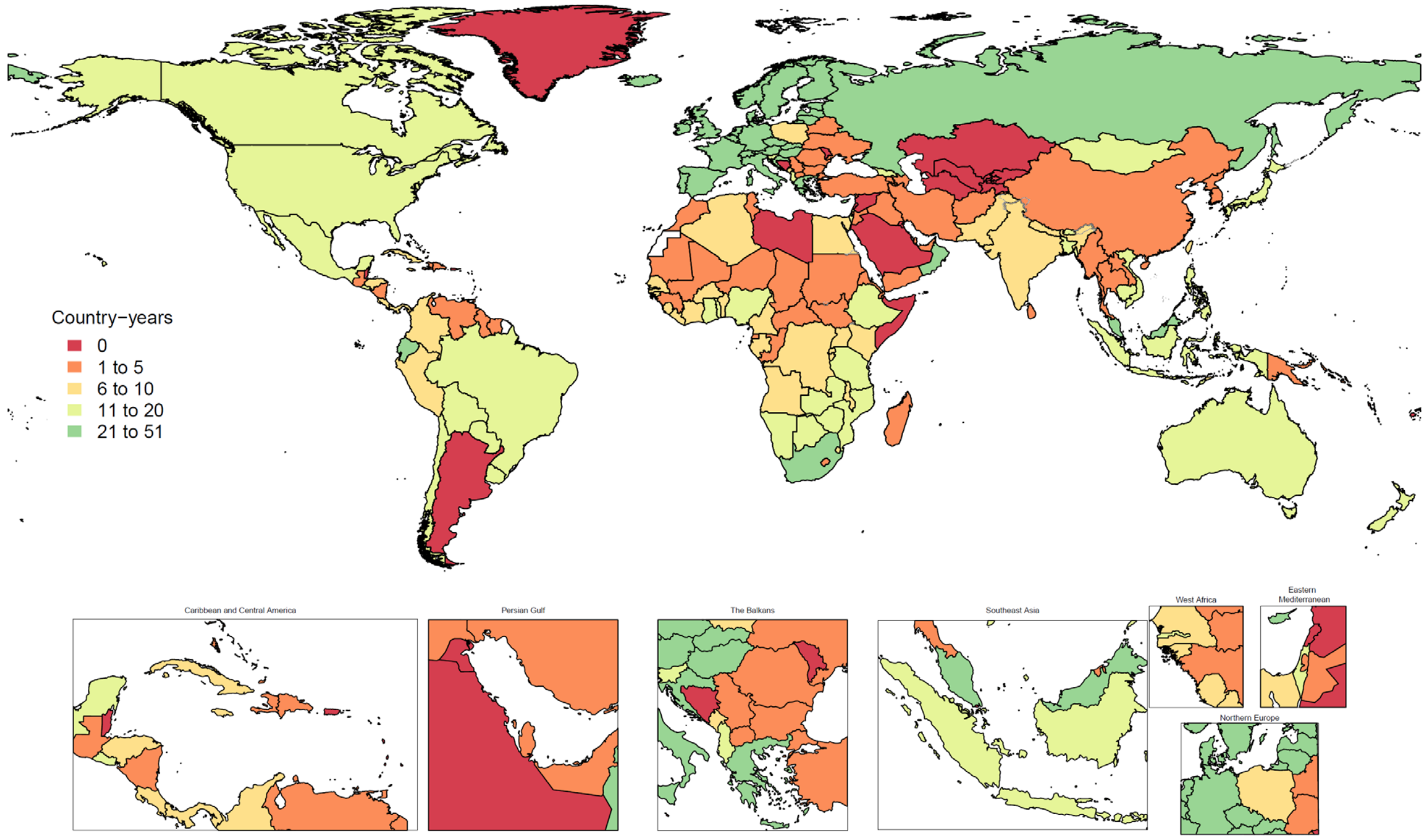

Appendix 1 Figure 2h: Country-years of community health worker density data, 1990–2023

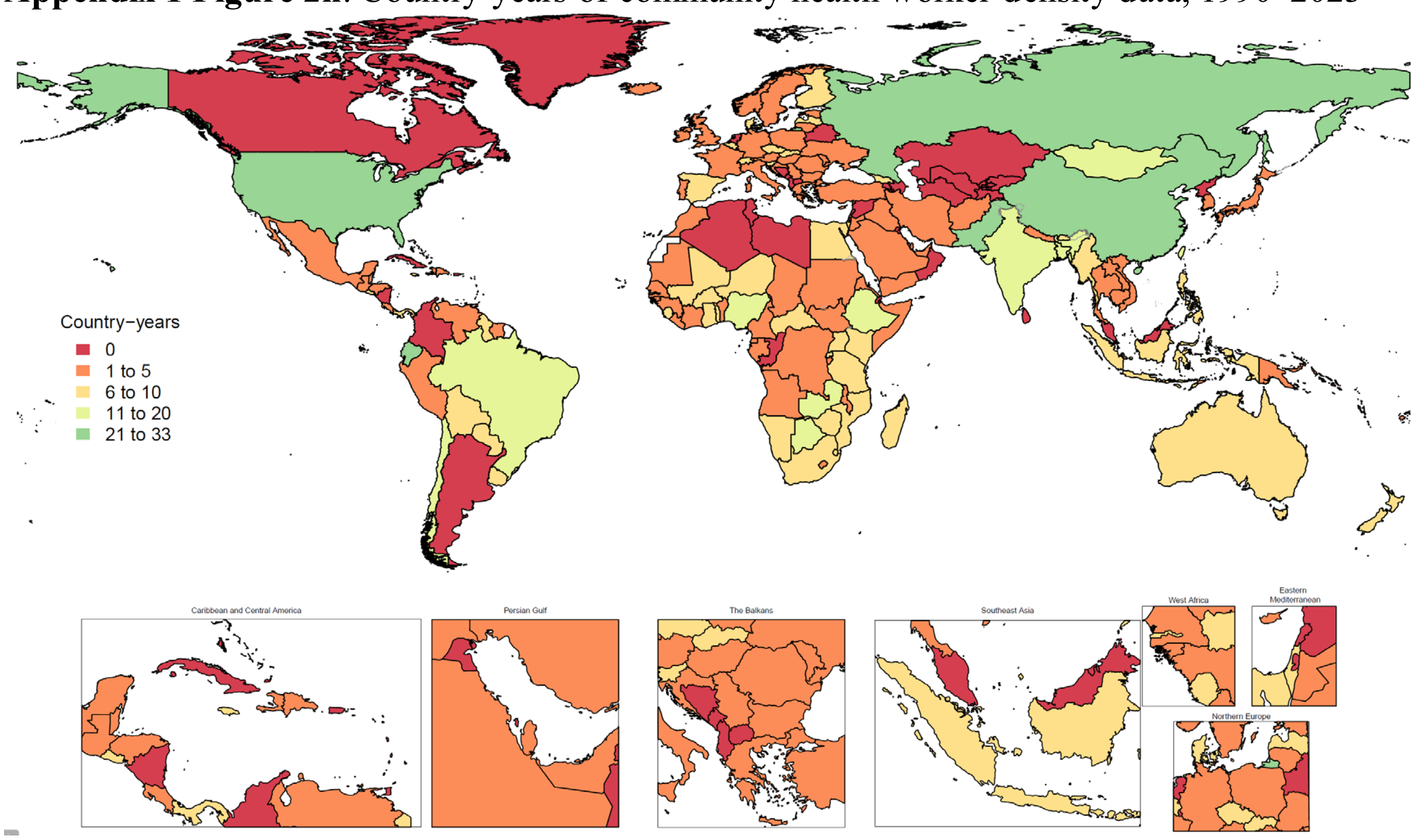

Appendix 1 Figure 2i: Country-years of all health worker density data, 1990–2023

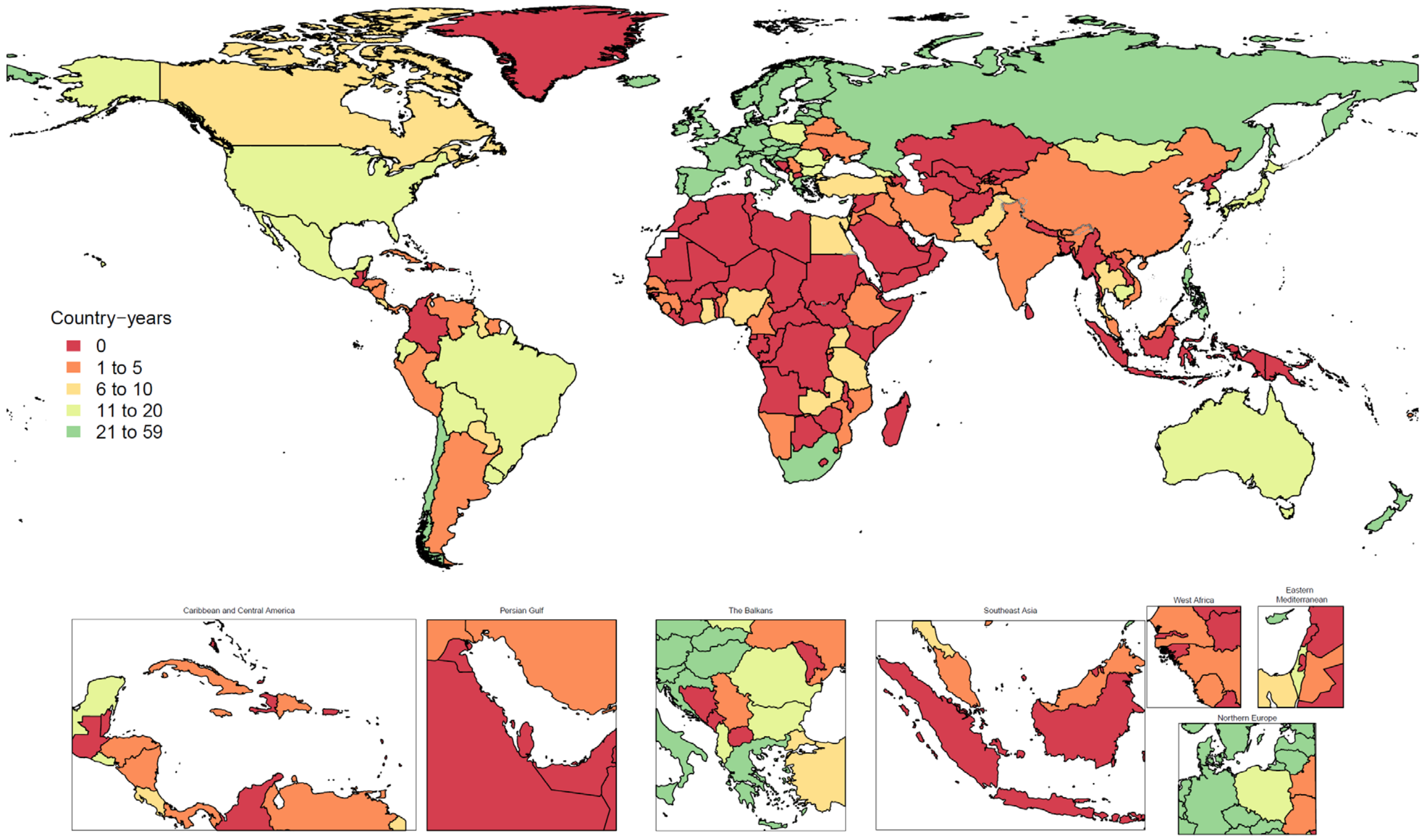

2.2.2 Health worker sex shares

Similarly, we leveraged administrative, population-based, and country-specific data resources to describe health worker sex distributions for eight specific cadres (doctors, nurses, midwives, pharmacists, pharmaceutical assistant, dentists, dental assistants and community health workers) and total health workers. In addition to the inclusion criteria described above, the data sources had to identify individuals using a binary definition of sex to inform the estimation of cadre-specific sex shares. The binary definition of sex was selected to align with the definition used in the vast majority of the data sources. To ensure that population-based data sources accurately reflected both the scale and sex distribution of each health worker cadre, we set a minimum sample size of five workers per cadre as an additional inclusion criterion. As a result of these additional inclusion criteria, less data were available for the estimation of health worker sex shares relative to the inputs available for health worker densities.

The same set of population-based sources was considered for health worker sex share estimation, but some lacked sex information or did not have sufficient sample sizes to reliably gauge sex distributions. Ultimately, 1,691 microdata sources (1,614 surveys and 77 surveys), representing 1,760 country-years, were used to estimate sex shares in this study. Eurostat provided sex-specific counts for doctors and nurses, enabling direct calculation of sex shares for these cadres only. Similarly, sex shares were obtainable for select cadres, namely doctors, nurses, midwives, and pharmacists, among a subset of locations and years from WHO's NHWA platform. Overall, for sex shares, administrative data comprised 1,852 country-years of input data describing the share of female health workers for four cadres: doctors, nurses, midwives, and pharmacists.

Additionally, we included 9 country-years from scientific literature describing doctors, nurses, and pharmacists in China to address data gaps in the second-most populous country in the world.

Figure 3 describes the distribution of sources across the indicators they contribute to in this study. While 92.7% of the country-years derived from population-based sources contained measures of sex shares for at least one cadre, only 47.6% of the country-years from administrative data and 9.6% of the country-years from scientific literature offered sex distributions for any health worker cadre.

Appendix 1 Figure 3: Country-years by data type and measure for all cadres, 1990–2023

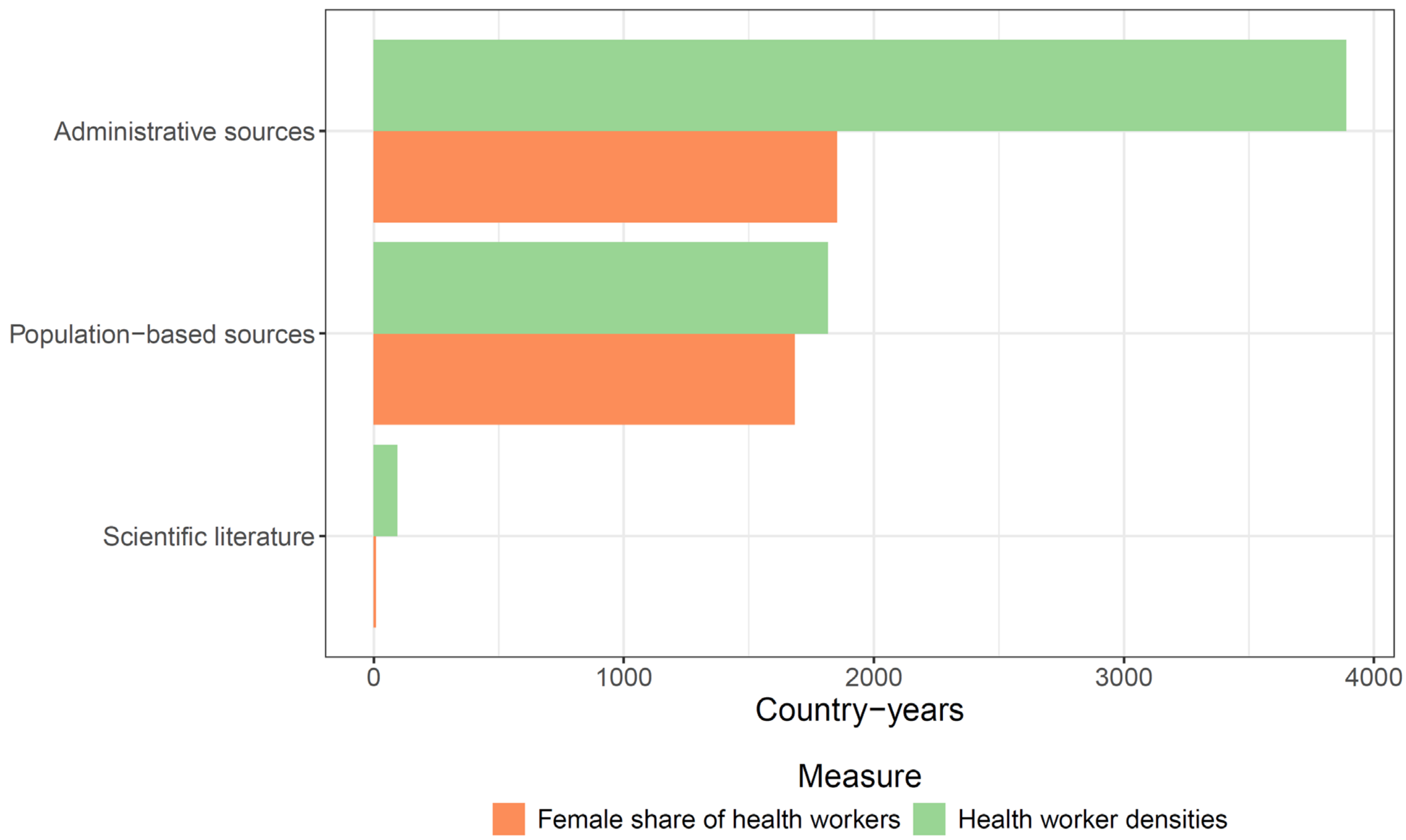

In total, this study employs 3,621 country-years from between 1990 and 2023 to estimate the female share of workers among eight cadres and all health workers. Figures 4a-i summarise the total number of country-years of data sources for sex shares by cadre employed in this study. We note better data coverage for doctors, nursing personnel, and midwifery personnel than the other specific cadres and the total health workforce.

Appendix 1 Figure 4: Country-years of health worker sex share data by cadre, 1990–2023

Appendix 1 Figure 4a: Country-years of doctor sex share data, 1990–2023

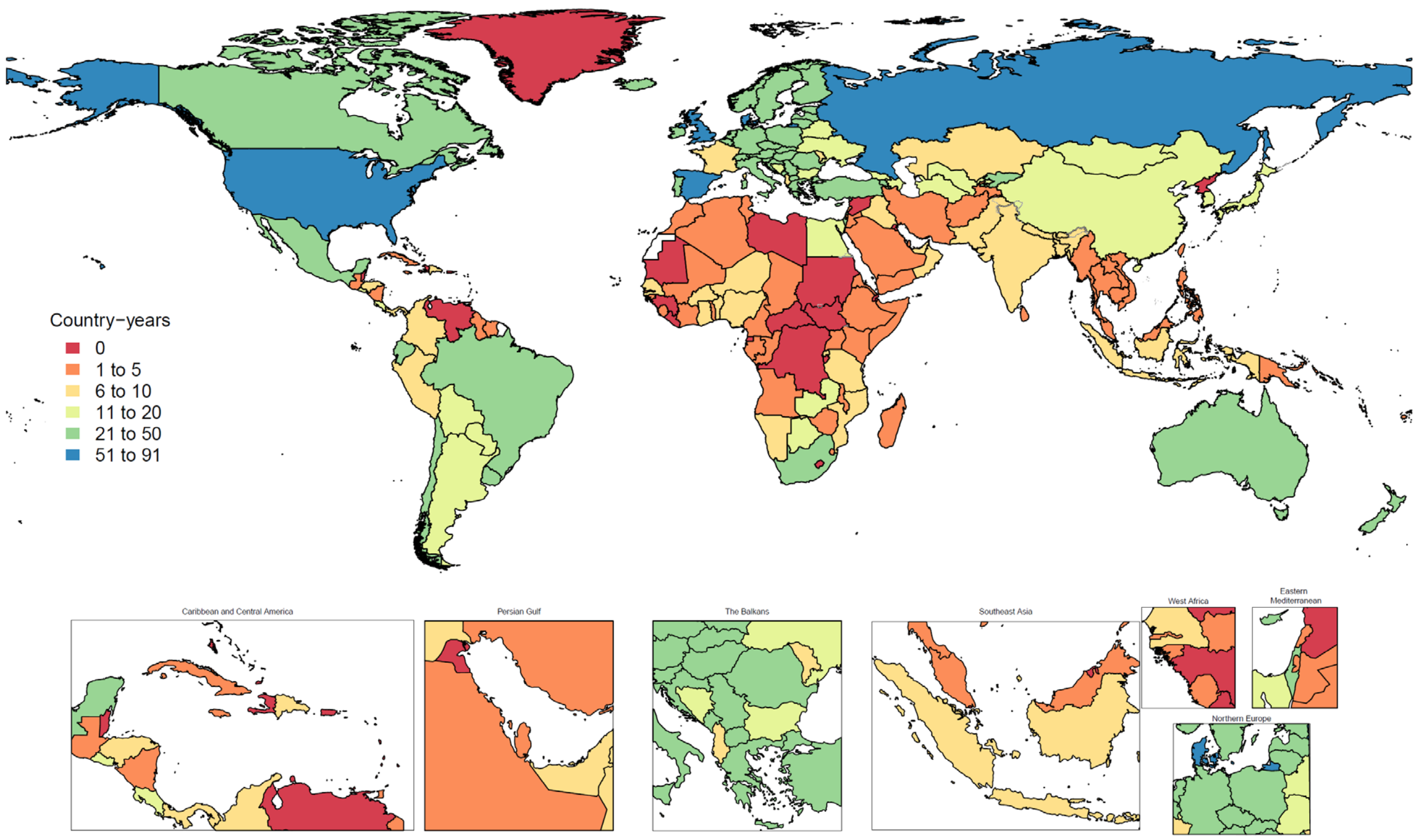

Appendix 1 Figure 4b: Country-years of nursing personnel sex share data, 1990–2023

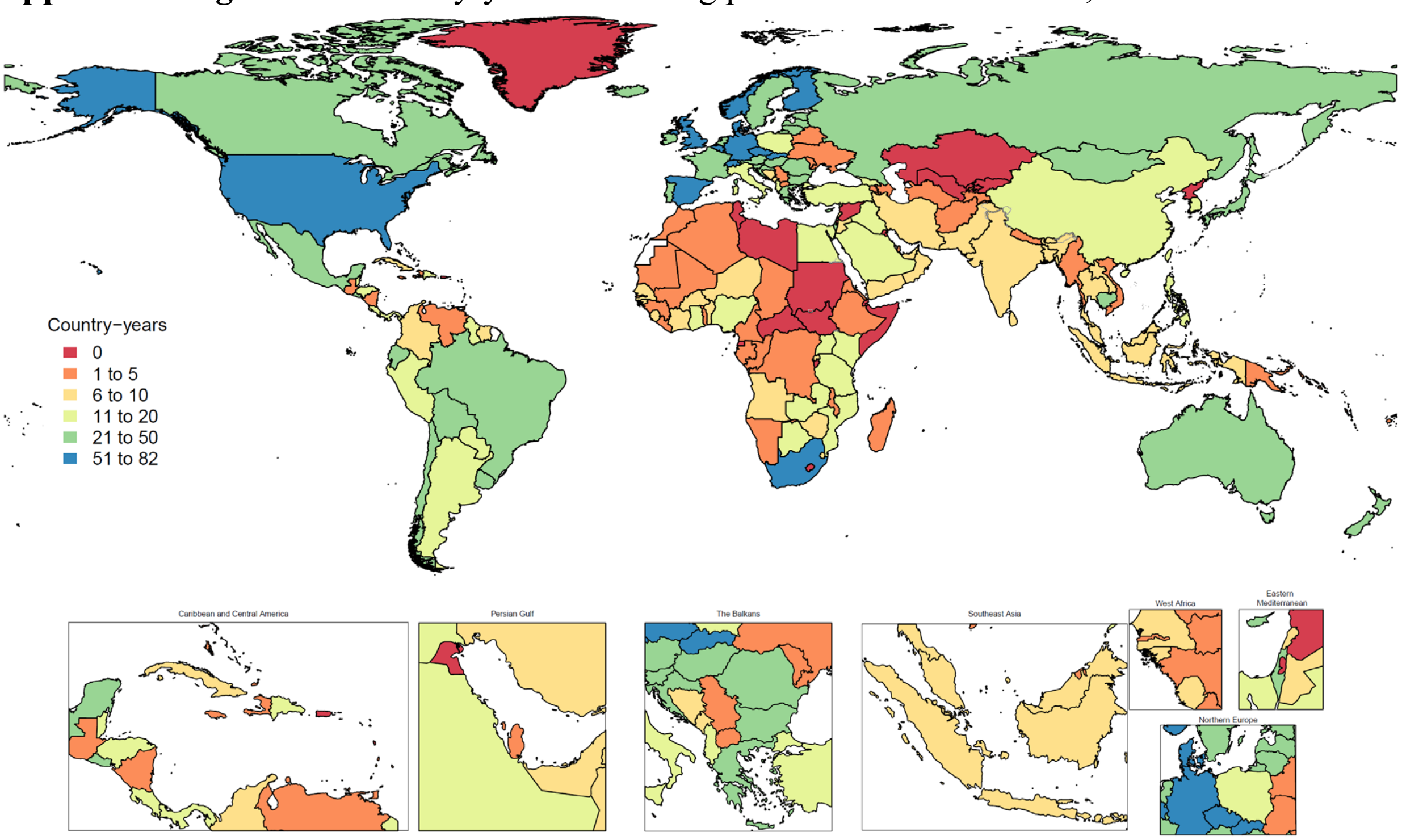

Appendix 1 Figure 4c: Country-years of midwifery personnel sex share data, 1990–2023

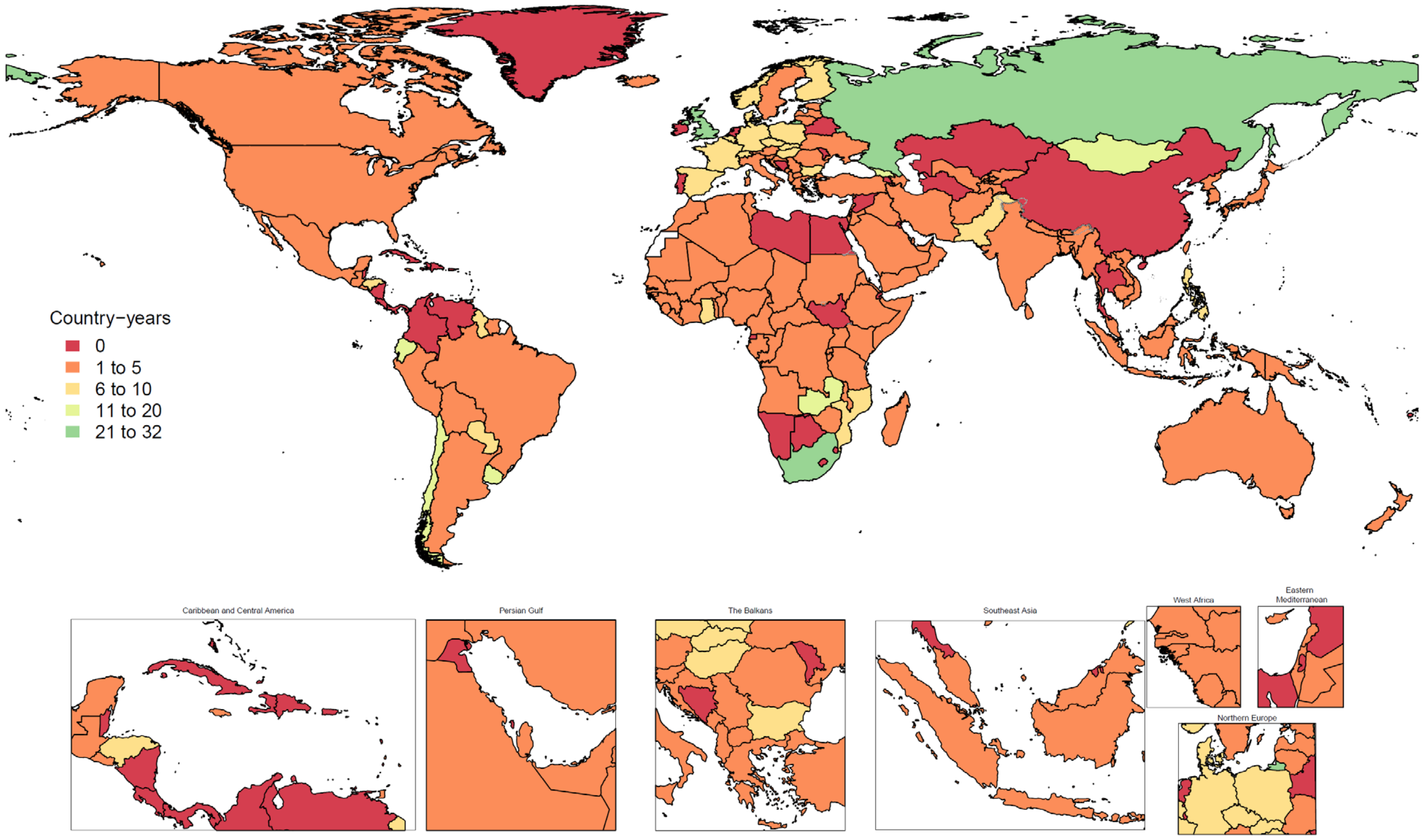

Appendix 1 Figure 4d: Country-years of dentist sex share data, 1990–2023

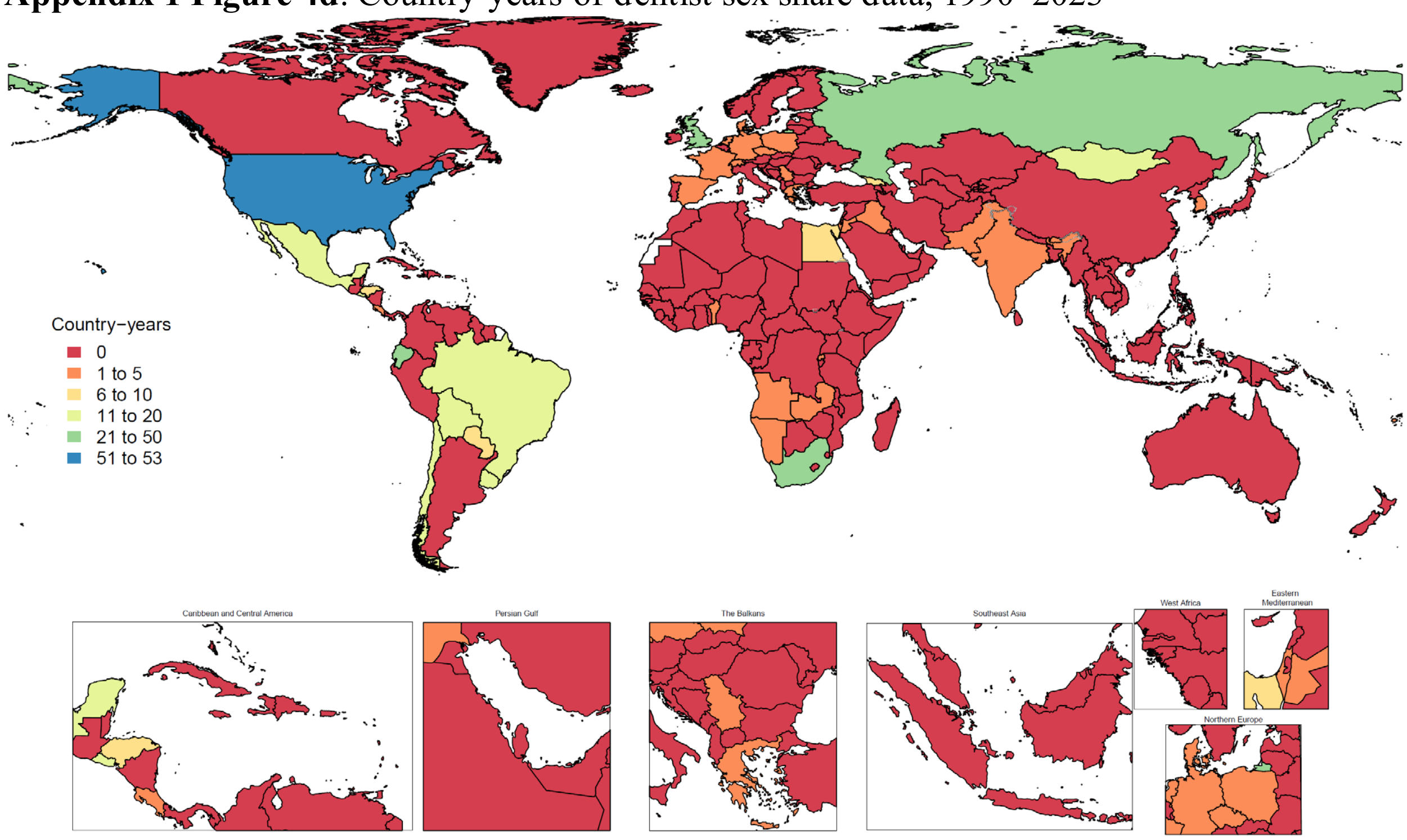

Appendix 1 Figure 4e: Country-years of dental assistant sex share data, 1990–2023

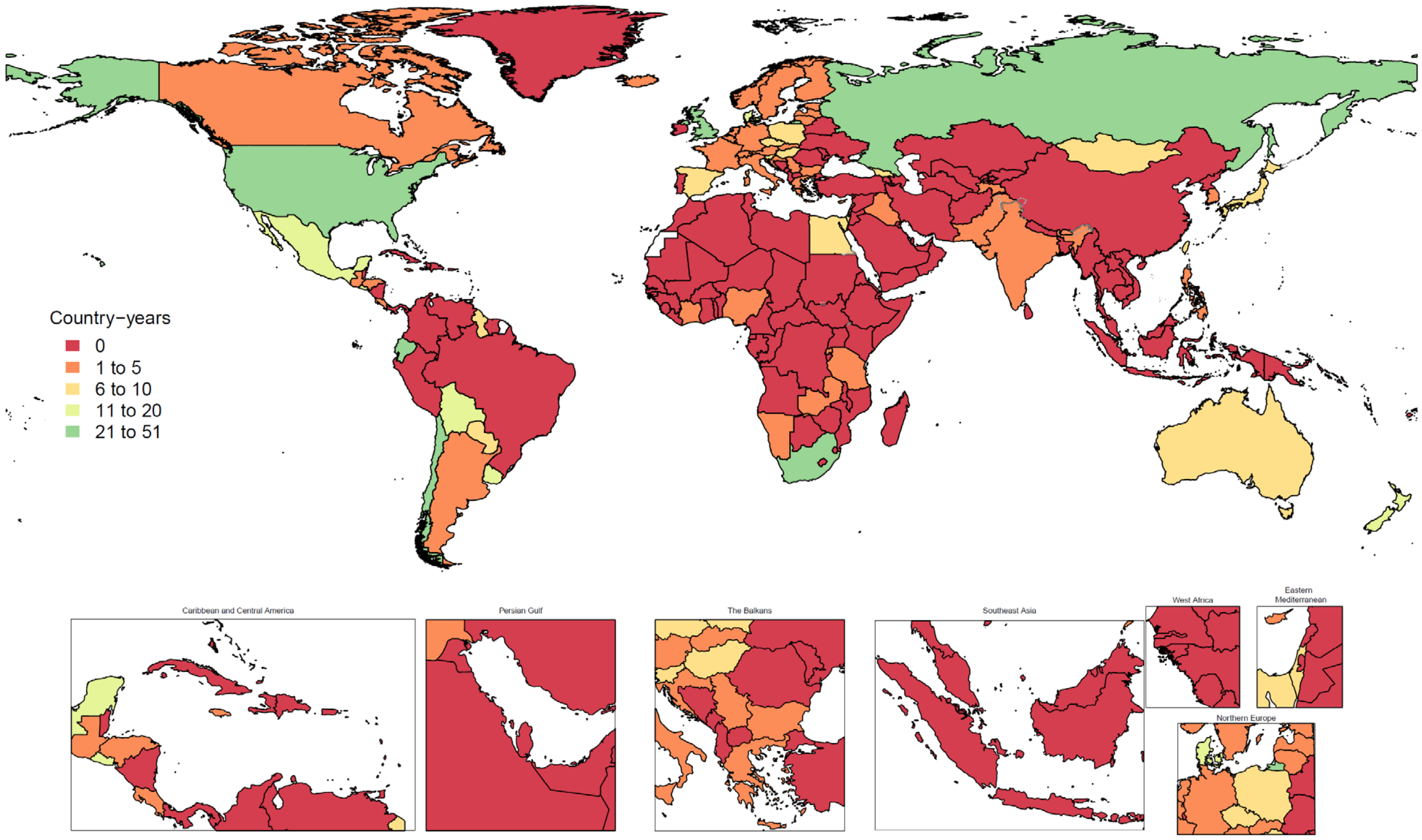

Appendix 1 Figure 4f: Country-years of pharmacist sex share data, 1990–2023

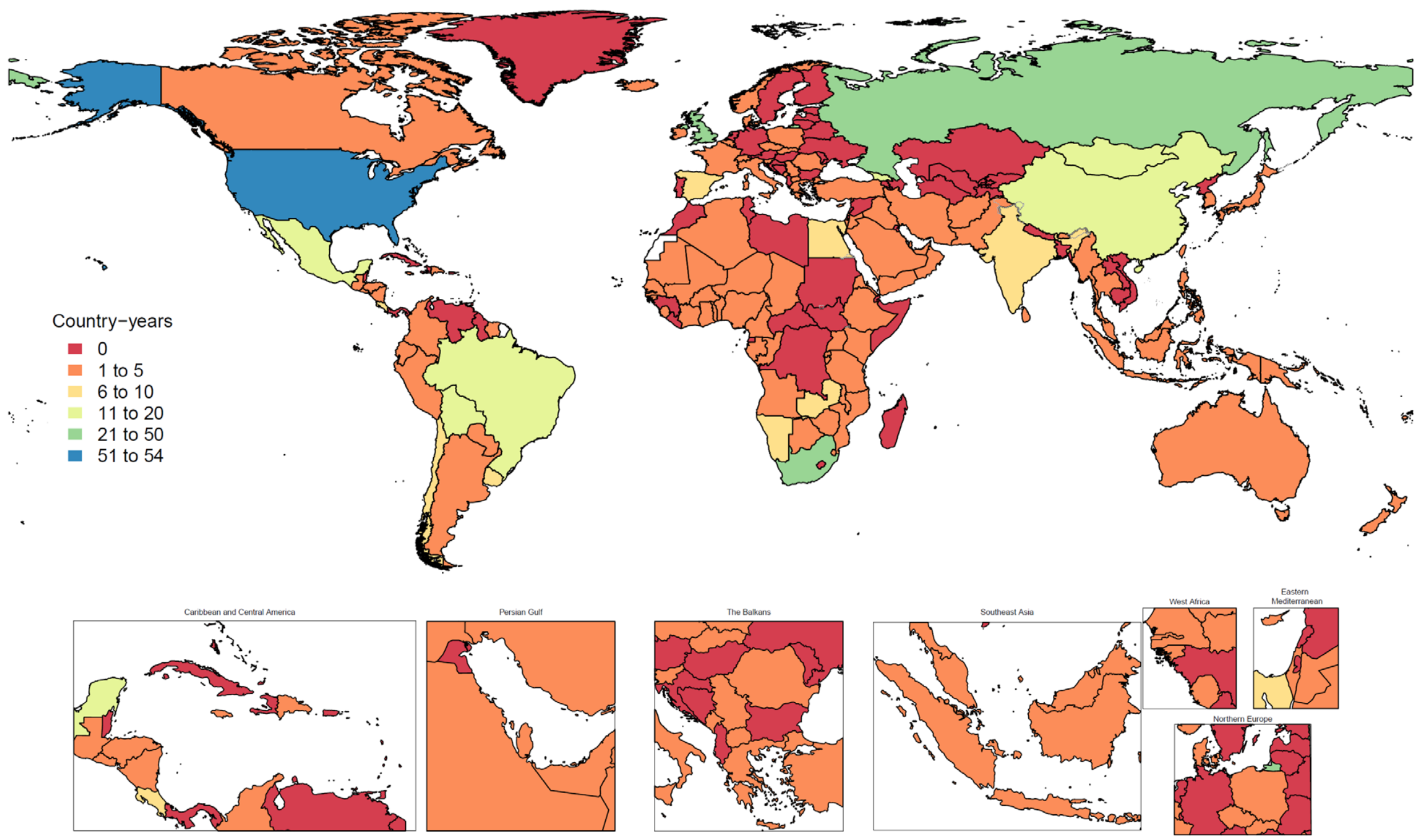

Appendix 1 Figure 4g: Country-years of pharmaceutical assistant sex share data, 1990–2023

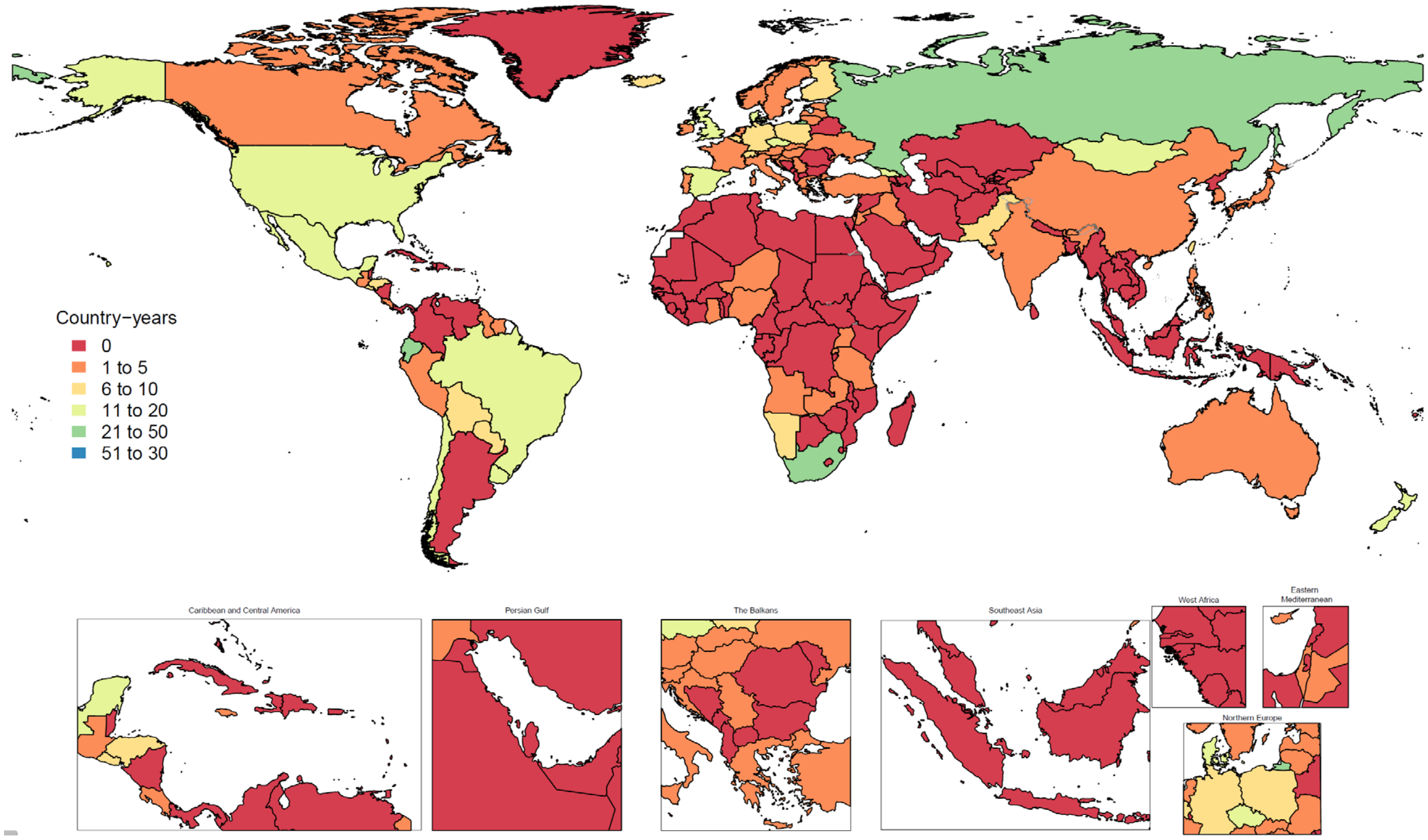

Appendix 1 Figure 4h: Country-years of community health worker sex share data, 1990–2023

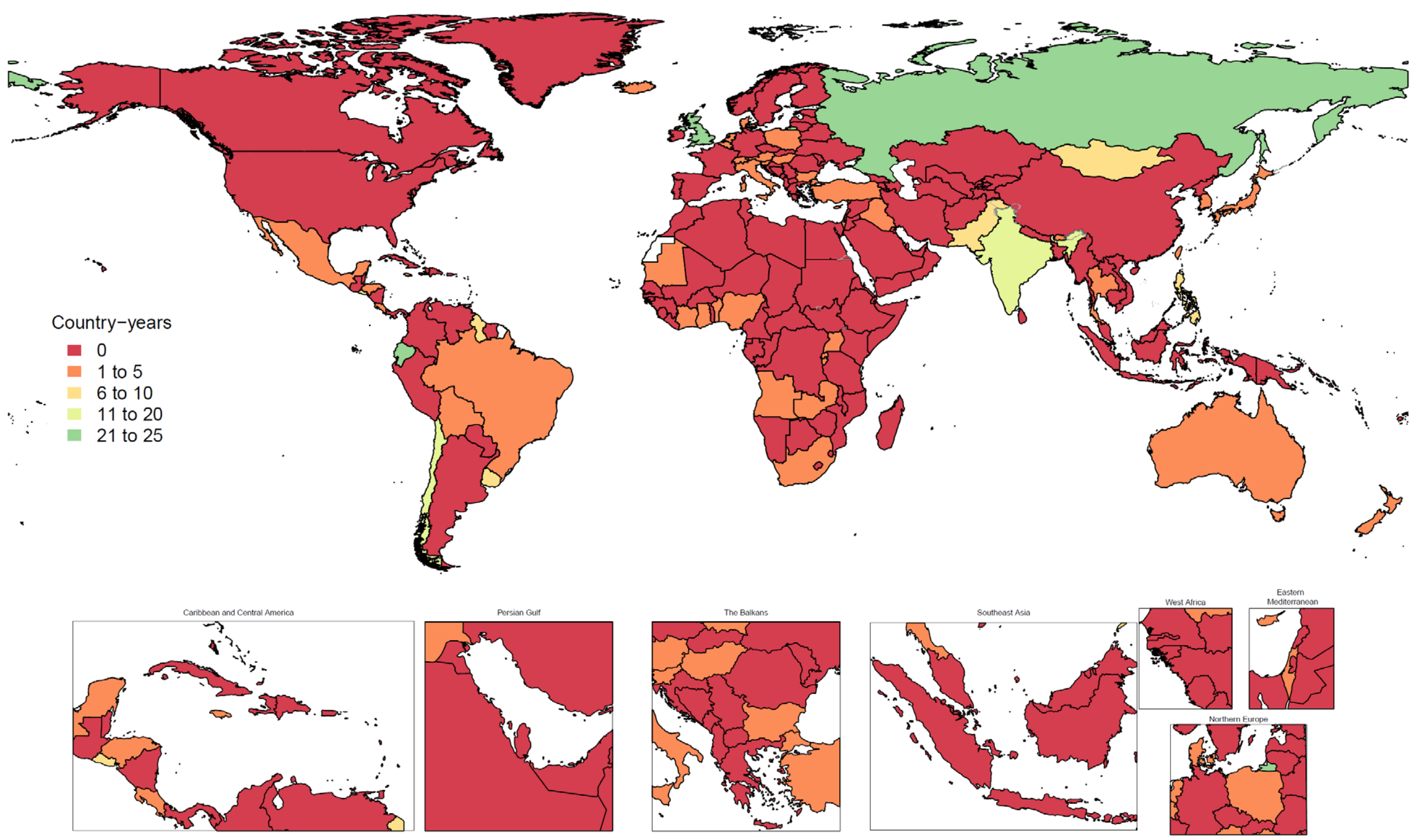

Appendix 1 Figure 4i: Country-years of all health worker sex share data, 1990–2023

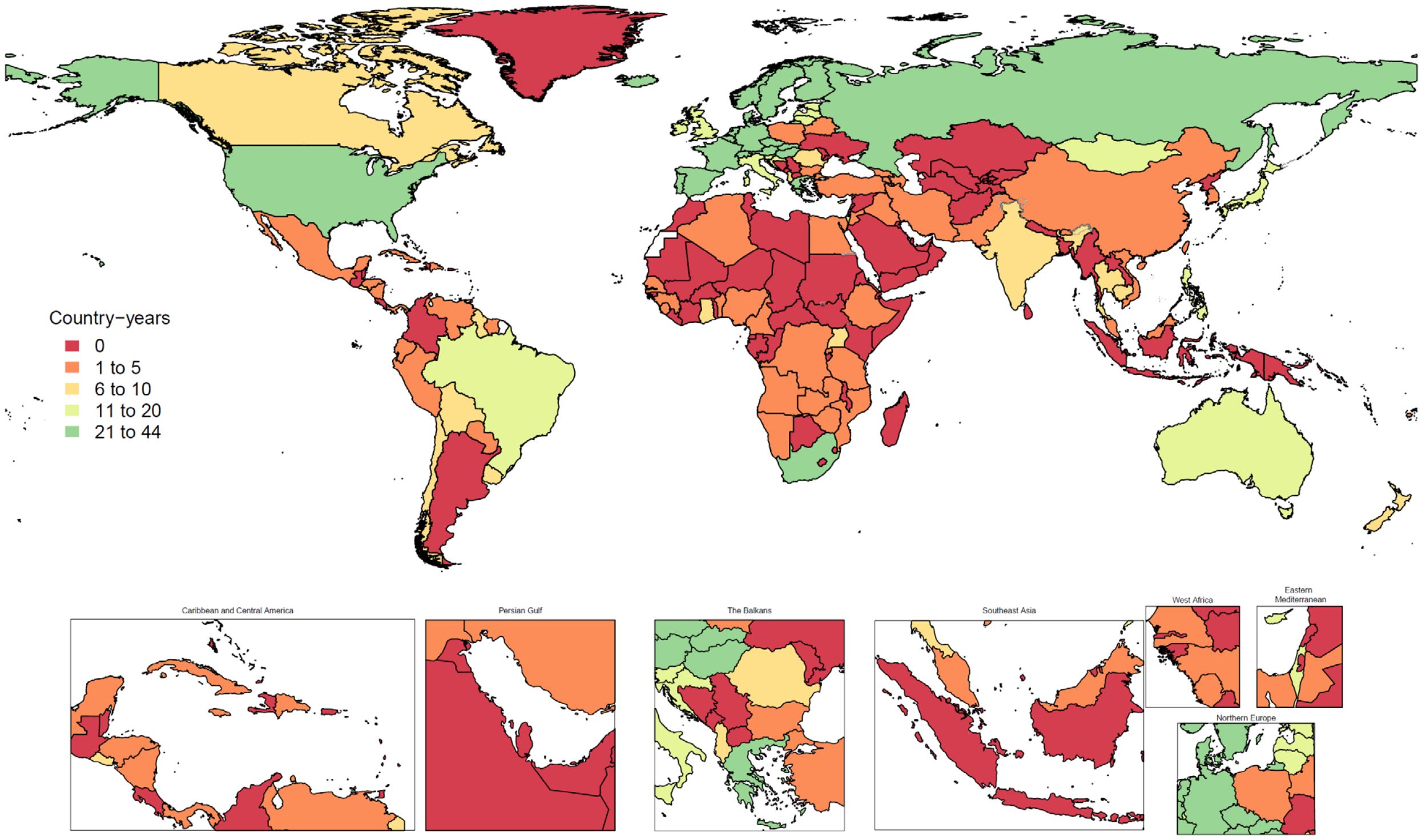

The administrative data describing health worker densities and sex shares do not report variance. We imputed variance in these sources based on the mean regional variance of the labour force surveys and censuses where population-based sources were available; we imputed the mean variance of these sources by super-region in locations without regional population-based data sources available.

2.3 Mapping and splitting

We mapped all coding systems used to classify the health workforce across all data sources to ISCO-08. The vast majority of the country-years of data (73.3%) map to our gold-standard ISCO-08 version because our administrative data sources (WHO NHWA and Eurostat data) use this coding system (Figure 5).

Appendix 1 Figure 5: Country-years of input data by occupation coding system

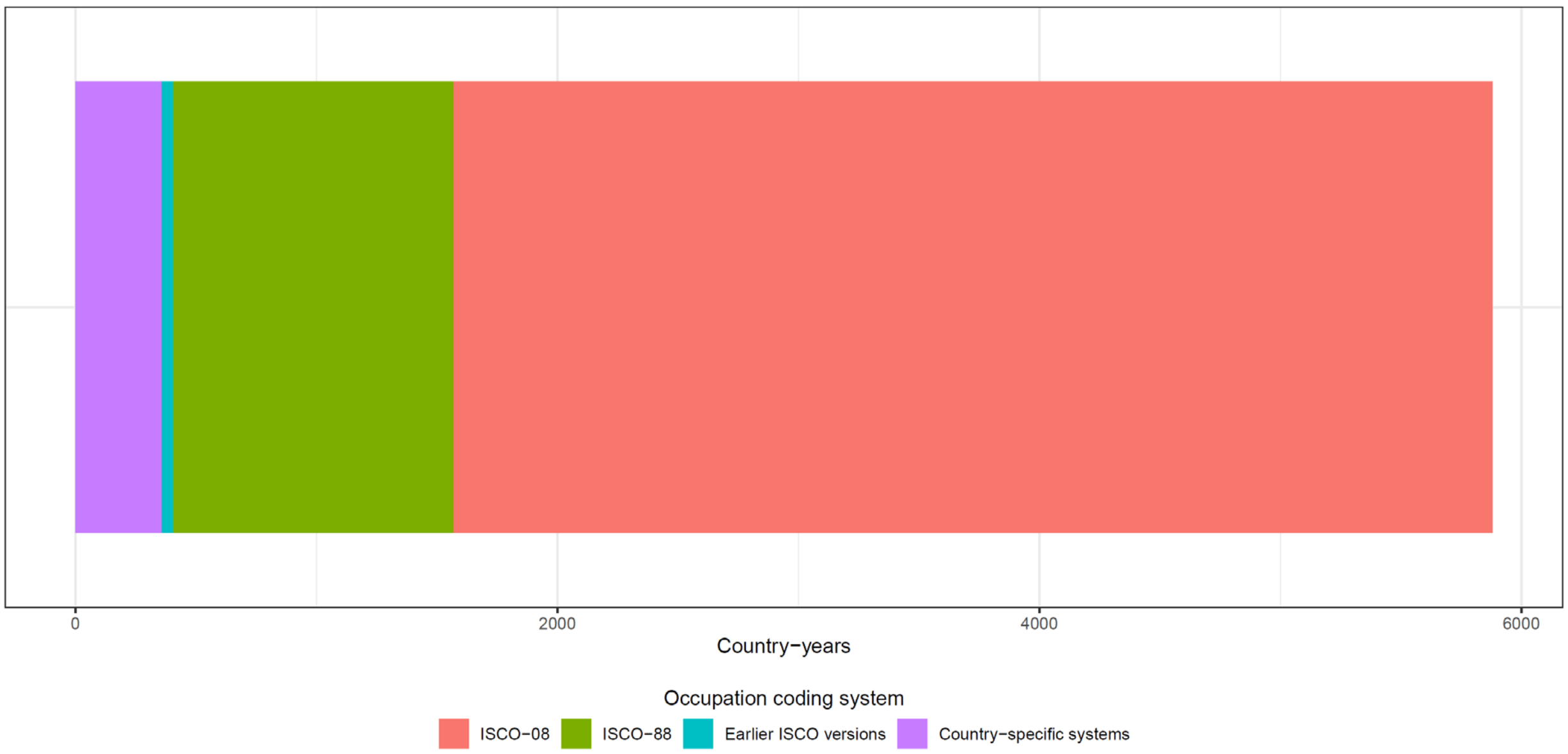

To make the data sources with alternative occupation coding systems useable for our analysis, they were systematically mapped to four-digit ISCO-08 occupation codes. The mapping process primarily involved aligning earlier ISCO versions with ISCO-08. Where previous versions provided sufficient detail to overlap with ISCO-08, direct mappings were possible. In cases where available data were constrained to the less granular three-digit level, codes were split to correspond with the four-digit ISCO-08 structure. For country-specific occupational coding systems, comparable mapping techniques were applied; however, these instances required more thorough procedures to ensure alignment with standard occupation coding structures.

To map previous ISCO versions to ISCO-08 codes, we referenced the ILO's ISCO concordance documentation.[16] The objective of mapping across ISCO versions was to create consistency and retain as much information as possible, but some inconsistencies and information loss were unavoidable. Frequently, precise matches in occupations codes between versions allowed exact mapping from one version to the other. Depicted in table 3, examples of this type of match are ISCO-68 (63) and ISCO-88 (2222) codes describing dentists in a way that directly corresponds to the ISCO-08 definition of the cadre. Note not all cadres from ISCO-08 have corresponding cadres in prior versions of ISCO; the lack of dental assistants in ISCO-68 is an example of this. In some cases, multiple ISCO-08 codes correspond to a single health worker cadre; see generalist (2211) and specialist medical practitioners (2212) being combined into a single doctor cadre.

Finally, table 3 highlights the needs for splitting methods to disaggregate cadres from previous ISCO versions that correspond to multiple health worker cadres that are uniquely identified with separate occupation codes in ISCO-08. For example, nursing and midwifery professionals are described using a single occupation code (2230) by ISCO-88 despite being two distinct fields of practice that are categorised as nursing and midwifery personnel using the updated health worker schema.

Appendix 1 Table 3: Health worker cadre mappings across most granular available ISCO versions

Health worker cadre	ISCO-68		ISCO-88		ISCO-08	
	Code	*Label*	*Code*	*Label*	*Code*	*Label*
Doctors	61	Physicians	2221	Physicians	2211	Generalist medical practitioners
					2212	Specialist medical practitioners
Nursing personnel	0-72	Nursing personnel n.e.c.	3231	Nursing associate professionals	2221	Nursing associates
	0-71	Professional nurses	2230	Nursing and midwifery professionals	3221	Nursing professionals
Midwifery personnel	0-73	Professional midwives	2230	Nursing and midwifery professionals	2222	Midwifery professionals
	0-74	Midwifery personnel n.e.c.	3232	Midwifery associate professionals	3222	Midwifery associates
Dentists	0-63	Dentists	2222	Dentists	2261	Dentists
Dental assistants	..	..	3225	Dental assistants	3251	Dental assistants and therapists
Pharmacists	0-67	Pharmacists	2224	Pharmacists	2262	Pharmacists
Pharmaceutical technicians	0-68	Pharmaceutical assistants	3228	Pharmaceutical assistants	3213	Pharmaceutical technicians and assistants
Community health workers	0-62	Medical assistants	3221	Medical assistants	3253	Community health workers
Clinical officers and medical assistants	0-62	Medical assistants	3221	Medical assistants	2240	Paramedical practitioners
					3256	Medical assistants
Medical laboratory technicians	..	..	3211	Medical equipment operators / Life science technicians	3212	Medical and pathology laboratory technicians

Medical imaging technicians (radiographers)	0-77	Medical X-ray technicians	3133	Medical equipment operators	3211	Medical imaging and therapeutic equipment technicians
Dieticians and nutritionists	0-69	Dieticians and public health nutritionists	3223	Dieticians and nutritionists	2265	Dieticians and nutritionists
Optometrists and opticians	0-75	Optometrists and opticians	3224	Optometrists and opticians	2267	Optometrists and ophthalmic opticians
					3254	Dispensing opticians
Physiotherapists	0-76	Physiotherapists and occupational therapists	3226	Physiotherapists and related associate professionals	2264	Physiotherapists
					3255	Physiotherapy technicians and assistants
Audiologists, speech therapists, and counsellors	..	..	3229	Modern health associate professionals n.e.c.	2266	Audiologists and speech therapists
					3259	Health associate professionals n.e.c.
Environmental health officers	..	..	3222	Sanitarians	2263	Environmental and occupational health and hygiene professionals
					3257	Environmental and occupational health inspectors and associates
Psychologists	1-95	Psychologists	2445	Psychologists	2634	Psychologists
Traditional and complementary medicine practitioners			3241	Traditional medicine practitioners	2230	Traditional and complementary medicine professionals
					3230	Traditional and complementary medicine associate professionals
Personal care workers (home-based)	..	..	5133	Home-based personal care workers	5322	Home-based personal care workers
Aides and emergency medical workers	..	..	5132	Institution-based personal care workers	5321	Health care assistants

					3258	Ambulance workers

Three-digit ISCO occupation codes were commonly employed in surveys and censuses, accounting for 59.4% of all population-based data sources. While three-digit ISCO codes generally did not allow us to identify specific HRH cadres, information from three-digit occupation codes was useful in creating envelopes from which individual health worker cadres could be split. For example, table 4 depicts the utility of a broad occupation code from ISCO-88 (222) that describes health professionals (except nursing) broadly. To perform occupation splits, we first used population-data sources with four-digit codes to inform preliminary models of densities for each health worker cadre. These densities are used to calculate the proportion each cadre represents of a given aggregate occupation, which are then applied to split codes into cadre-specific inputs for a given GBD location and year. Table 4 depicts ISCO minor groups (three-digit occupations) and corresponding unit groups (four-digit occupations) that undergo occupation splitting into designated health worker cadres. For example, table 4 highlights that we modelled four cadres in the preliminary modelling phase: doctors, dentists, pharmacists, and veterinarians, which is a residual category to split a single occupation code describing all health professionals (except nursing). Preliminary modelling requires estimating each HRH cadre and relevant residual categories made up of four-digit codes associated with each three-digit code of interest that did not correspond to any health worker cadre. By using four-digit data to split three-digit codes, we were able to capture variation in the proportional makeup of health worker cadres across locations and years, rather than relying on a single global proportion for all settings and time periods.

Appendix 1 Table 4: List of three-digit and four-digit occupation codes used for splitting and corresponding health worker cadre mappings

Occupation coding system	Three-digit codes		Four-digit codes		Mapped health worker cadre	Number of split points *(n)*
	Codes	*Labels*	*Codes*	*Labels*		
ISCO-88	222	Health professionals (except nursing)	2221	Physicians	Doctors	733
			2222	Dentists	Dentists	
			2222	Pharmacists	Pharmacists	
			2223	Veterinarians	Residual cadre	
ISCO-88	244	Social science and related professionals	2445	Psychologist	Psychologist	712
			2441	Economists	Residual cadre	
			2442	Sociologists, anthropologists, and related professionals		
			2443	Philosophers, historians, and political scientists		
			2444	Philologists, translators, and interpreters		
			2446	Social work professionals		
ISCO-88	313		3133	Medical equipment operators	Medical imaging and therapeutic equipment technicians	670

		Optical and electronic equipment operators	3131	Photographers and image and sound recording equipment operators	Residual cadre	
			3132	Broadcasting and telecommunications equipment operators		
ISCO-88	321	Life science technicians and related associate professionals	3211	Life science technicians	Medical laboratory technicians	656
			3212	Agronomy and forestry technicians	Residual cadre	
			3213	Farming and forestry advisers		
ISCO-88	322	Modern health professionals	3253*	Community health workers	Community health workers	726
			2240*	Paramedical practitioners	Clinical officers and medical assistants	
			3256*	Medical records and health information technicians		
			3222	Environmental health workers	Environmental health workers	
			3223	Dieticians and nutritionists	Dieticians and nutritionists	
			3224	Optometrists and ophthalmic opticians	Optometrists and opticians	
			3225	Dental assistants	Dental assistant	
			3226	Physiotherapists and related associate professionals	Physiotherapists and prosthetic technicians	
			3228	Pharmaceutical assistants	Pharmaceutical technicians	
			3229	Other health associate professionals not elsewhere classified	Audiologists, speech therapists, and counsellors	
			3227	Veterinary assistants	Residual cadre	
ISCO-88	324	Traditional medicine practitioners and faith healers	3241	Traditional medicine practitioners	Traditional and complementary medicine practitioners	84
			3242	Faith healers	Residual cadre	
ISCO-88	513	Personal care and related workers	5133	Home-based personal care workers	Personal care workers	735
			5132	Institution-based personal care workers	Health-care aides, paramedics, and ambulance workers	
			5139	Personal care and related workers		
			5131	Child-care workers	Residual cadre	
ISCO-08	226; 325; 532	Other health professionals; Other health associate professionals; Personal care workers in health services	2261	Dentists	Dentists	208
			2262	Pharmacists	Pharmacists	
			2263	Environmental and occupational health professionals	Environmental health workers	
			2264	Physiotherapists	Physiotherapists and prosthetic technicians	
			2269	Health professionals not elsewhere classified		
			3255	Physiotherapy technicians and assistants		
			2265	Dieticians and nutritionists	Dieticians and nutritionists	
			2266	Audiologists and speech therapists	Audiologists, speech therapists, and counsellors	
			3259	Health associate professionals not elsewhere classified		
			2267	Optometrists and ophthalmic opticians	Optometrists and opticians	
			3254	Dispensing opticians		
			3251	Dental assistants and therapists	Dental assistants	
			3253*	Community health workers	Community health workers	
			2240*	Paramedical practitioners	Clinical officers and medical assistants	
			3256*	Medical records and health information technicians		
			3258	Ambulance workers	Health-care aides, paramedics, and ambulance workers	
			5321	Health care assistants		
			5322	Home-based personal care workers	Personal care workers	

			5329	Personal care workers in health services not elsewhere classified		
			3252	Medical records and health information technicians	Residual cadre	
			3257	Environmental and occupational health inspectors		
ISCO-08	263	Social science and related professionals	2634	Psychologists	Psychologist	216
			2631	Economists	Residual cadre	
			2632	Sociologists, anthropologists, and related professionals		
			2633	Philosophers, historians, and political scientists		
			2635	Social work and counselling professionals		
			2636	Religious professionals		
ISCO-08	314, 321	Life science technicians; Health associate professionals	3141	Life science technicians (excluding medical)	Medical laboratory technicians	219
			3212	Medical and pathology laboratory technicians		
			3211	Medical imaging and therapeutic equipment technicians	Medical imaging and therapeutic equipment technicians	
			3213	Pharmaceutical technicians and assistants	Pharmaceutical technicians	
			3142	Agricultural technicians	Residual cadre	
			3143	Forestry technicians		
			3214	Medical and dental prosthetic technicians		

* Codes described in column d do not correspond with occupation codes indicated in column a. In this case, mappings to the latest occupation coding system (ISCO-08) are depicted.

Some population-based and administrative sources use coding systems not clearly based on ISCO, which we refer to as country-specific occupation coding systems. There was considerable variation in the level of detail available for different health worker cadres, necessitating thorough review of available source documentation to identify overlap with ISCO-08. All country-specific occupation coding systems were manually mapped to health worker cadres after translation, if needed. To verify those mappings, we used ChatGPT-4 to review occupation coding systems and match cadre descriptions to ISCO-08 definitions of relevant health worker cadres. An example of the prompt we employed is: "Using documentation describing the occupations from a *given public source*, identify occupations directly or indirectly related to health care. Export that list of occupations for my review please." Another prompt we utilized to was: "Try mapping occupations from a *given public source* to the following four-digit occupations from ISCO-08 describing health workers: 5139, 5132, 3229, 3221, 2222, 3225, 3223, 3222, 3211, 3232, 2230, 3231, 3224, 5133, 2224, 3228, 2221, 2445, 3133, 3226, 3241. Please make sure to base mappings with consideration to skill level, tasks, and duties." We did not share personal data or rely on automated occupation mappings from ChatGPT-4 in this study. Only occupations where associated descriptions of job titles, definitions, and tasks sufficiently matched those of three-digit or four-digit ISCO-08 were mapped. Typically, country-specific coding systems only provided corresponding cadres for a subset of the HRH cadres included in this analysis. Some examples of data sources that use these types of occupation coding systems include India's

National Sample Survey, which uses the National Classification of Occupations (NCO); Mexico's National Survey of Household Income and Expenditure, which uses Sistema Nacional de Clasificación de Ocupaciones (SINCO); and the United States Current Population Survey, which uses the Standard Occupational Classification (SOC). In cases where country-specific occupation codes corresponded with a four-digit ISCO-08 code, they were included in preliminary and final models along with densities using standardised coding system. Where codes were matched with a three-digit ISCO-08 code and could be split into multiple estimated cadres without residual groups, we used the method described above for three-digit ISCO codes.

We also generated a dataset to estimate the aggregate of all health workers combined. Data for this group consisted of the summed densities of all cadres, after mapping and splitting, from sources whose coding systems allowed identification of every cadre included in this study.. To improve data coverage, we relaxed the inclusion criteria for measuring all health workers in several ways. We allowed flexible definitions of nursing and midwifery personnel, recognising that some sources may classify nurses only as professionals or associates. Additionally, we included mappings derived from three-digit occupation codes that comprehensively describe the health workforce. Table 5 lists the occupation codes included in the expansion of the health worker envelope. Where additional occupation codes mapped directly to health worker cadres, they were included in addition to those depicted in table 5 if available from a given source.

Appendix 1 Table 5: Three-digit occupation codes that describe the health workforce by occupation coding system

Codes	Labels
**ISCO-88 (1988)**	
222	Health professionals (except nursing)
322	Modern health associate professionals
223*	Nursing and midwifery professionals
323*	Nursing and midwifery associate professionals
**ISCO-08 (2008)**	
221	Physicians
222*	Nursing and midwifery professionals
322*	Nursing and midwifery associate professionals
321	Medical and pharmaceutical technicians
224	Paramedical practitioners
226	Other health professionals
325	Other health associate professionals
532	Personal care workers in health services

* Indicates only one of the codes is required to meet inclusion criteria. Both occupation codes were leveraged if available.

2.4 Input data adjustment strategy

We employed methods developed in previously-published research to adjust administrative data, using population-based sources as the gold standard for measuring HRH.[2] Surveys and censuses typically use similar questionnaire formats and occupational coding systems to characterise national workforces. While both sources rely on population-based sampling, they differ in sample sizes, resulting in varying levels of sampling error and precision. Importantly, these errors are random and do not introduce systematic bias. Because they are based on population

sampling, surveys and censuses avoid many potential biases found in administrative data sources. Administrative sources often face challenges related to data quality, coverage, and timeliness, which can lead to both over- and under-reporting of HRH levels. For example, WHO reports that most administrative data included in the NHWA database are derived from HRHIS to WHO offices via nationally nominated focal points from ministries of health. However, limited information is publicly available regarding the source of individual datapoints. Documentation on limitations from the WHO include incomplete capture of the private sector, double-counting of workers employed at multiple facilities, inclusion of individuals no longer practicing or who have emigrated, and other inaccuracies, however data quality have improved greatly over time through the efforts of many, including the designated nationally nominated focal points. We modelled correction factors with 821 matched pairs of population-based data to administrative data, with the total number of matched pairs differing depending on the cadre depicted in table 6.

Appendix 1 Table 6: Number of matched pairs by cadre

Cadre	Number of matched pairs
Doctors	742
Dentists	365
Pharmacists	356
Nursing personnel	280
Midwifery personnel	160
Dental assistants	67
Pharmaceutical assisant	55

We allowed matches for data that differ by up to one year (for example matching a 2016 survey with a 2017 administrative datapoint). The steps to the adjustment procedure are as follows: (1) identify matched pairs of observations from reference (population-based) and alternative (administrative) data sources, defined as health worker densities for the same location with observations within one year of each other; (2) transform observations into logit space using the delta method; (3) calculate the difference between alternative and reference sources and calculate the standard error of the difference; (4) use these differences in a lasso regression with indicators by geography to identify which locations require adjustment; (5) use these differences as the dependent variable in a meta-regression—Bayesian, regularised, trimmed (MR-BRT) model to estimate correction factors; and (6) apply correction factors to administrative data to be used in subsequent modelling steps.

As described in step (4), prior to implementing adjustments for all administrative data sources, we used lasso regression to determine whether to apply an adjustment and what type of adjustment to apply. For each cadre, we ran two lasso regressions on differences in health worker densities from each cadre across sources to test whether adjustments were needed, one using location indicators and another using region and super-region indicators, with the penalty (lambda) set to one standard deviation above the mean minimum lambda from 1000-fold cross-

validation. Adjustments were only made where indicator coefficients were non-zero, indicating the administrative data differed from population-based data. Adjustments with the most geographical specificity took precedence over other adjustment types (eg, location-specific adjustments were used instead of regional adjustments if both were indicated by the regressions). Table 8 shows the lambdas selected and the RMSE from each lasso model.

Appendix 1 Table 7. Lambdas and RMSE from the lasso covariate selection regressions, by cadre

Cadre	Location adjustments		Super-region/region adjustments	
	Lambda	*RSME*	*Lambda*	*RSME*
Doctors	0.006	0.465	0.016	0.532
Nursing personnel	0.007	0.383	0.026	0.463
Midwifery personnel	0.034	0.830	0.095	0.861
Dentists	0.021	0.636	0.024	0.738
Dental assistants	0.094	0.998	0.125	0.998
Pharmacists	0.017	0.765	0.031	0.973
Pharmaceutical assistant	0.282	0.926	0.242	0.940

As described in step (5), for administrative data meeting our adjustment criteria, we estimate correction factors with MR-BRT. The MR-BRT tool is described in detail in other GBD 2023 publications.[4,5] Briefly, MR-BRT is a flexible meta-regression approach that fits a spline informed by a prior for the shape of the curve, with options for linear tails and trimming. In our case, we used the Bayesian properties of MR-BRT, employing a Gaussian prior, to ensure our correction factor estimates were regularised, eg, not unduly influenced by data with small sample sizes or single high-leverage points with sizeable differences from the rest of the evidence considered. MR-BRT propagates model uncertainty and data uncertainty through to the adjusted estimates (of the WHO and Eurostat data), important for subsequent modelling steps. We run MR-BRT models by super-region, region, and location (in selected geographies) to estimate correction factors. The geographical level used to estimate correction factors depended on the results from our lasso regressions, as described above. In addition to this statistical evidence, we opted to not implement corrections where the observed population data were, relative to the administrative data, in the opposite direction from the estimated correction factor—for example, the population data were higher than the administrative data, but the correction factor would reduce the density from the administrative data, moving it farther away from the population-based estimate.

Where:

i denotes a given matched pair of population-based sources to WHO or Eurostat data

$I(location)$ denotes an indicator for each location selected in the lasso regression

$I(region)$ denotes an indicator for each region selected in the lasso regression

$I(super\ region)$ denotes an indicator for each super-region selected in the lasso regression

β is the adjustment factor for locations, regions, or super-regions selected in the lasso regression, modelled with a Gaussian prior in MR-BRT

ε_i is the residual

We opted not to extend this adjustment approach to community health worker densities due to the availability of only a small number of matched pairs (n <50), insufficient data to inform an adjustment. Similarly, we did not employ this approach to adjust health worker sex shares because the sex shares calculated from the administrative data were not systematically different from the sex shares estimated from the labour force surveys and censuses (correlation >0.92). The types of adjustments applied to administrative data from each cadre and location are depicted below (figures 6a-6g). Maps of the percentage adjustment based on the correction factors estimated in MR-BRT are depicted in figures 7a-7g.

Appendix 1 Figure 6: Adjustment type for administrative densities from WHO NHWA and Eurostat Health Database by cadre
Appendix 1 Figure 6a: Adjustment type for administrative doctor densities from WHO NHWA and Eurostat Health Database by cadre

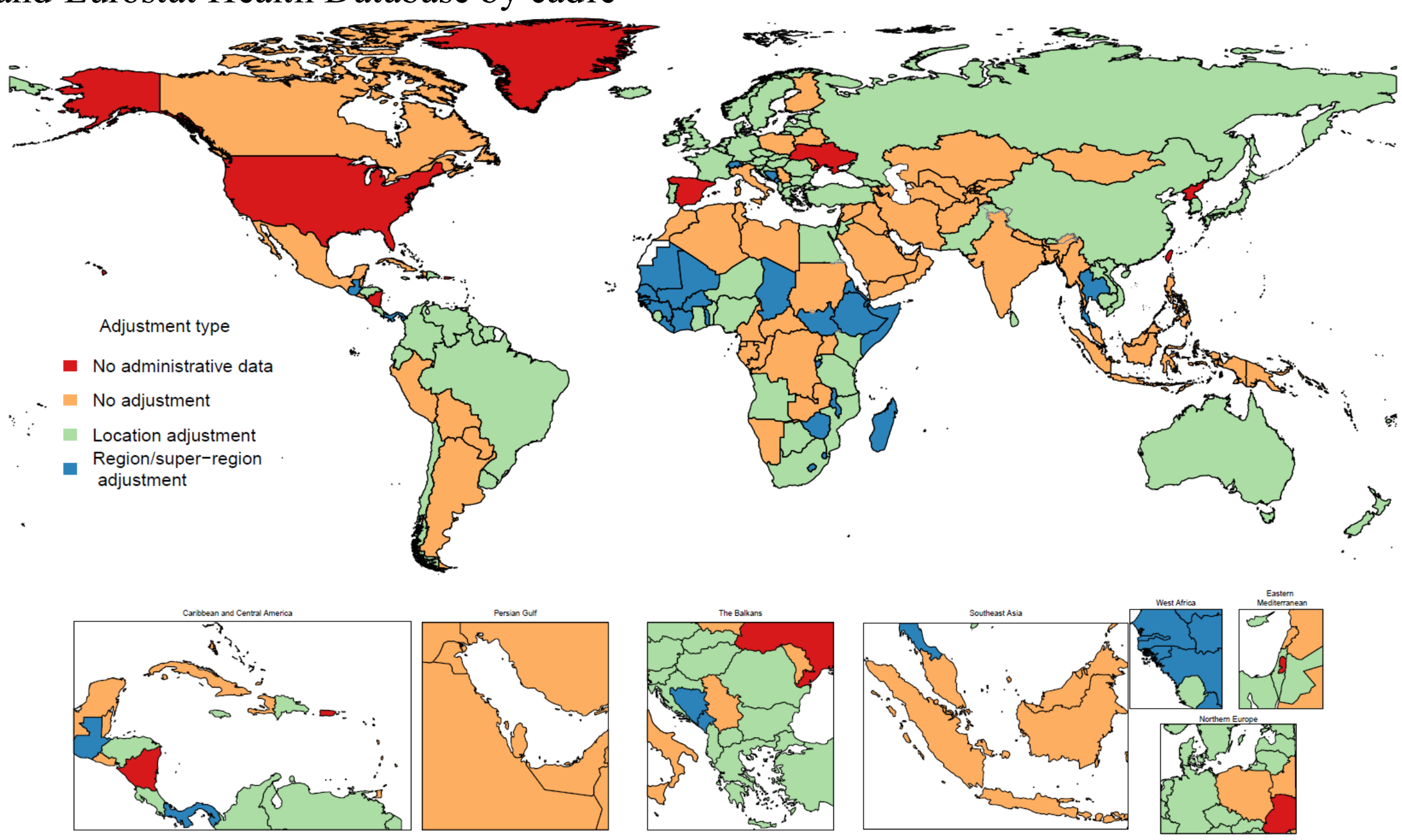

Appendix 1 Figure 6b: Adjustment type for administrative nursing personnel densities from WHO NHWA and Eurostat Health Database by cadre

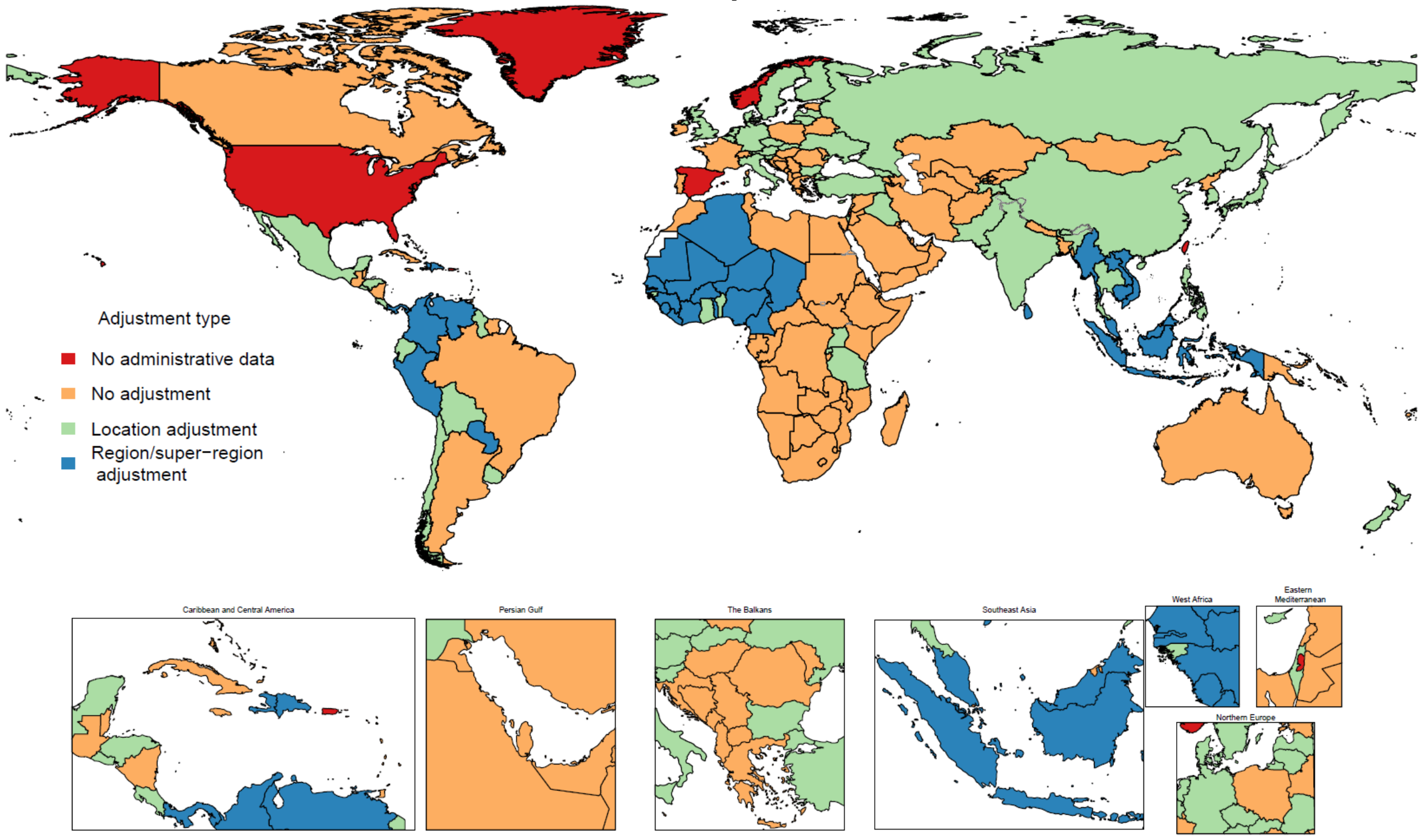

Appendix 1 Figure 6c: Adjustment type for administrative nursing personnel densities from WHO NHWA and Eurostat Health Database by cadre

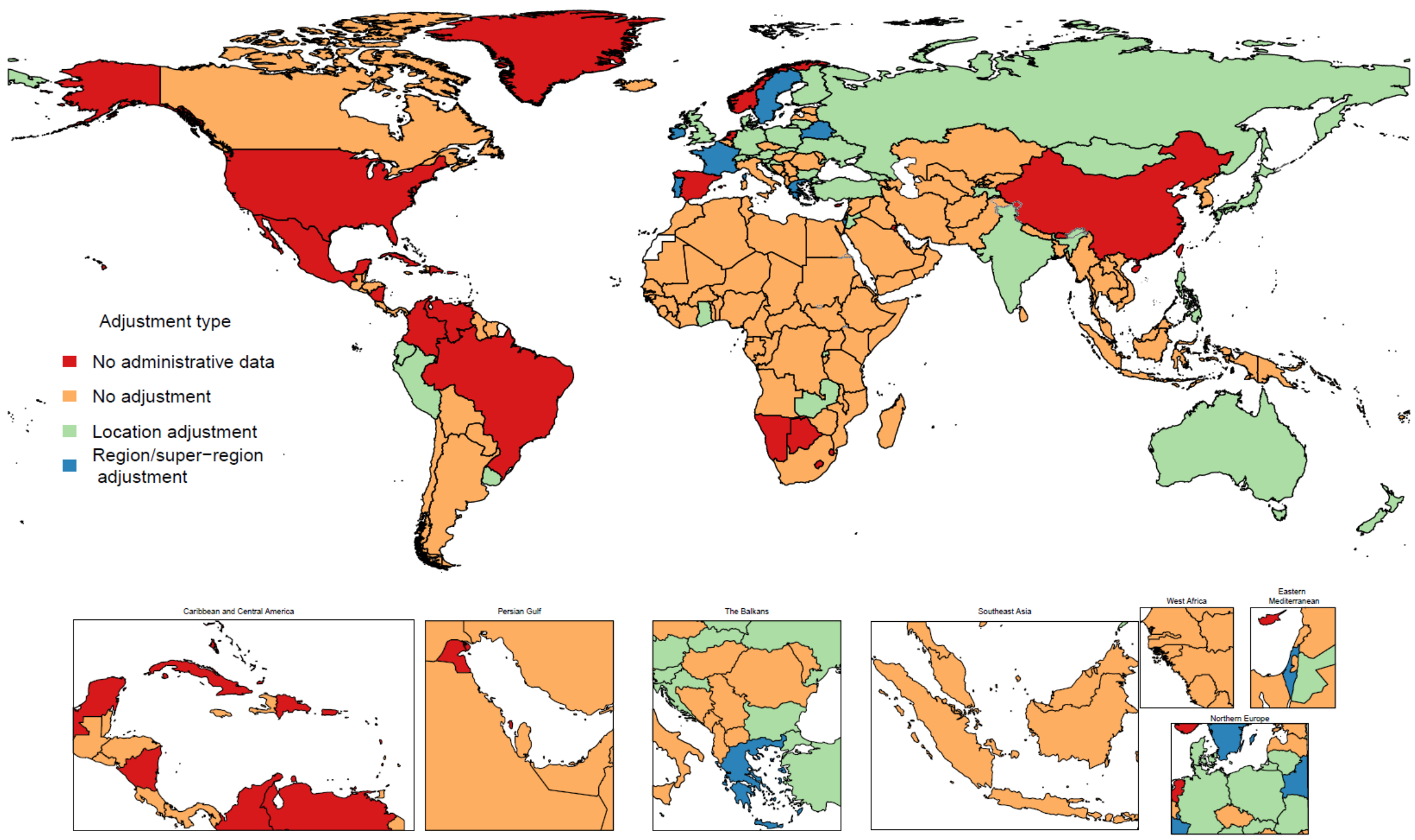

Appendix 1 Figure 6d: Adjustment type for administrative dentist densities from WHO NHWA and Eurostat Health Database by cadre

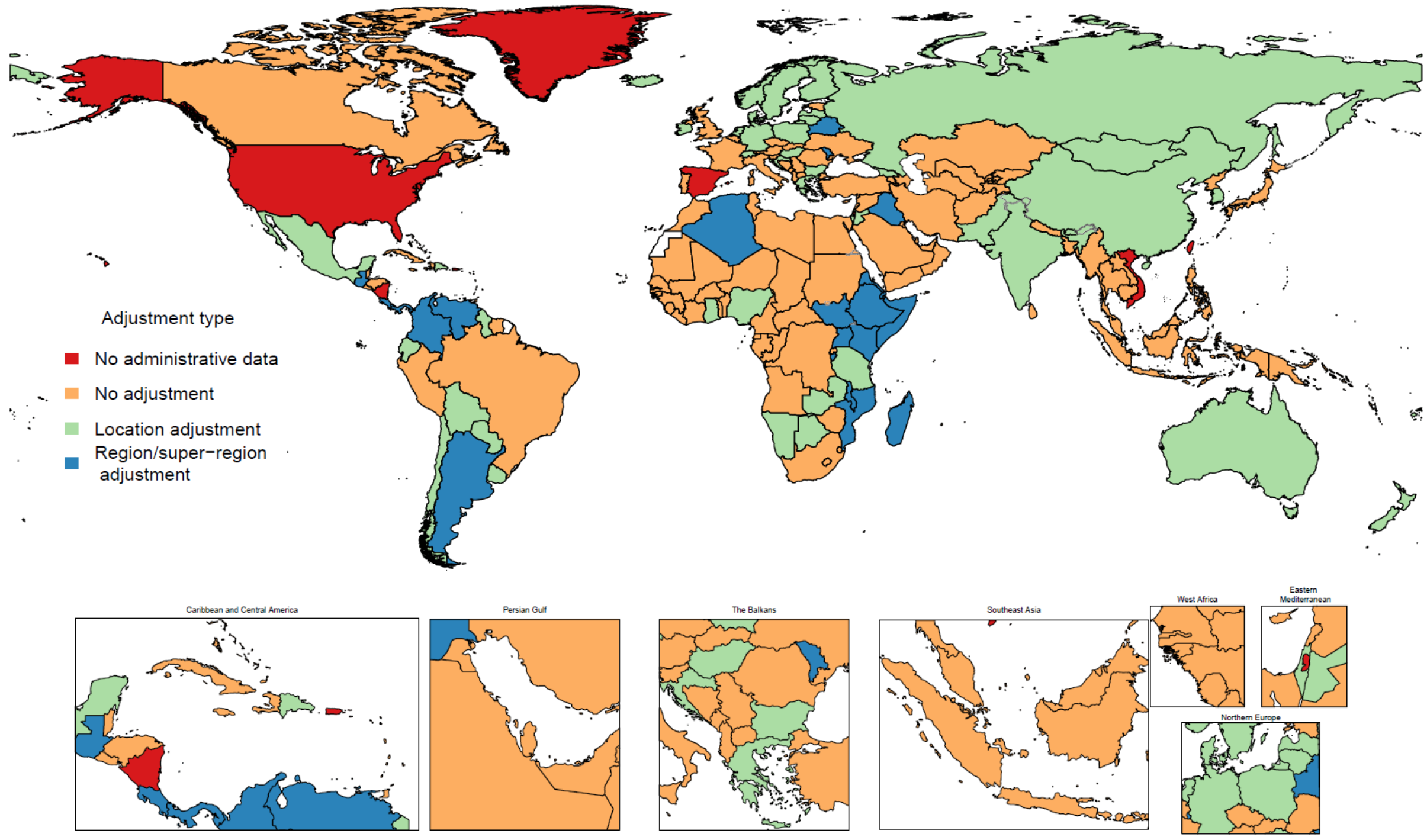

Appendix 1 Figure 6e: Adjustment type for administrative dental assistant densities from WHO NHWA and Eurostat Health Database by cadre

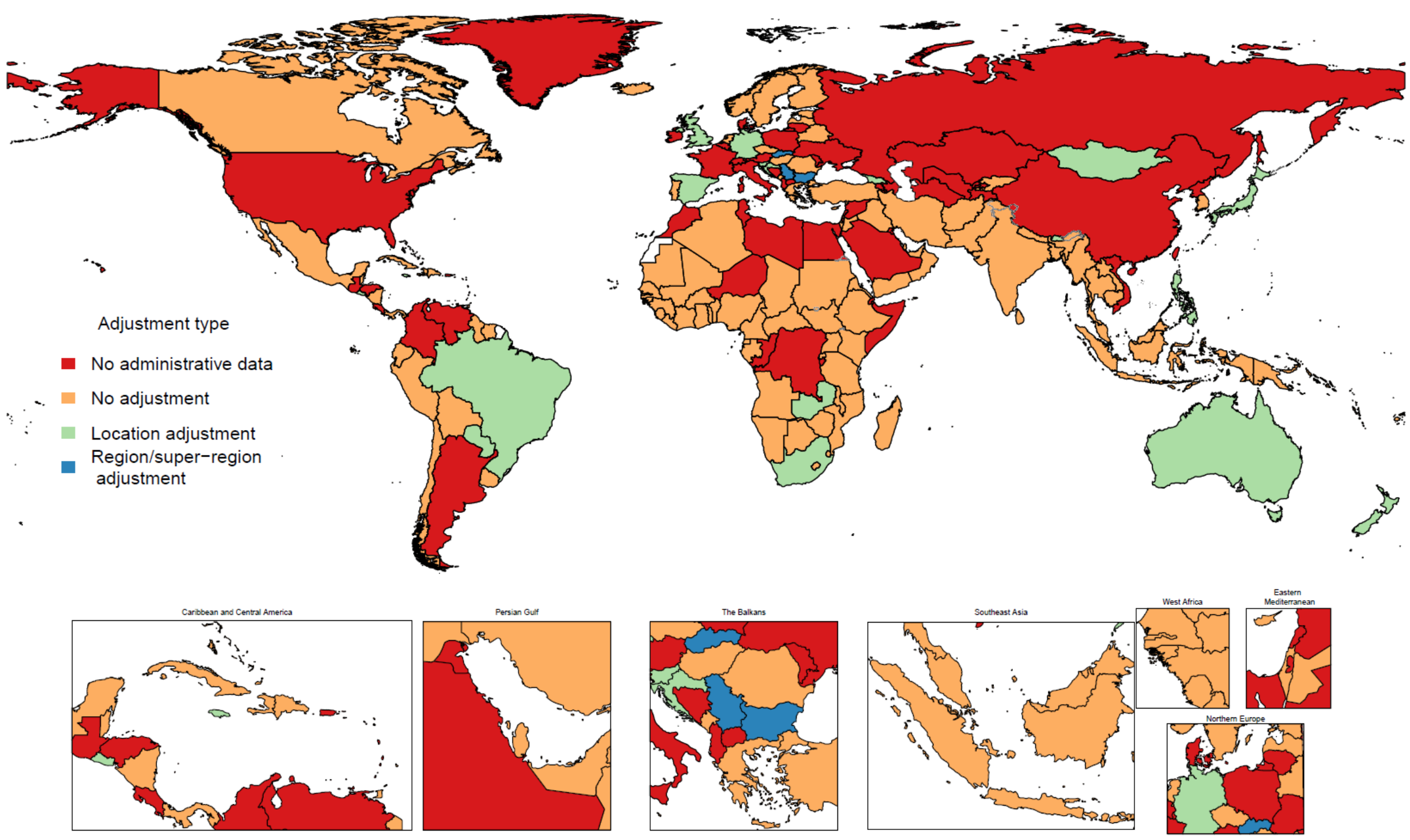

Appendix 1 Figure 6f: Adjustment type for administrative pharmacist densities from WHO NHWA and Eurostat Health Database by cadre

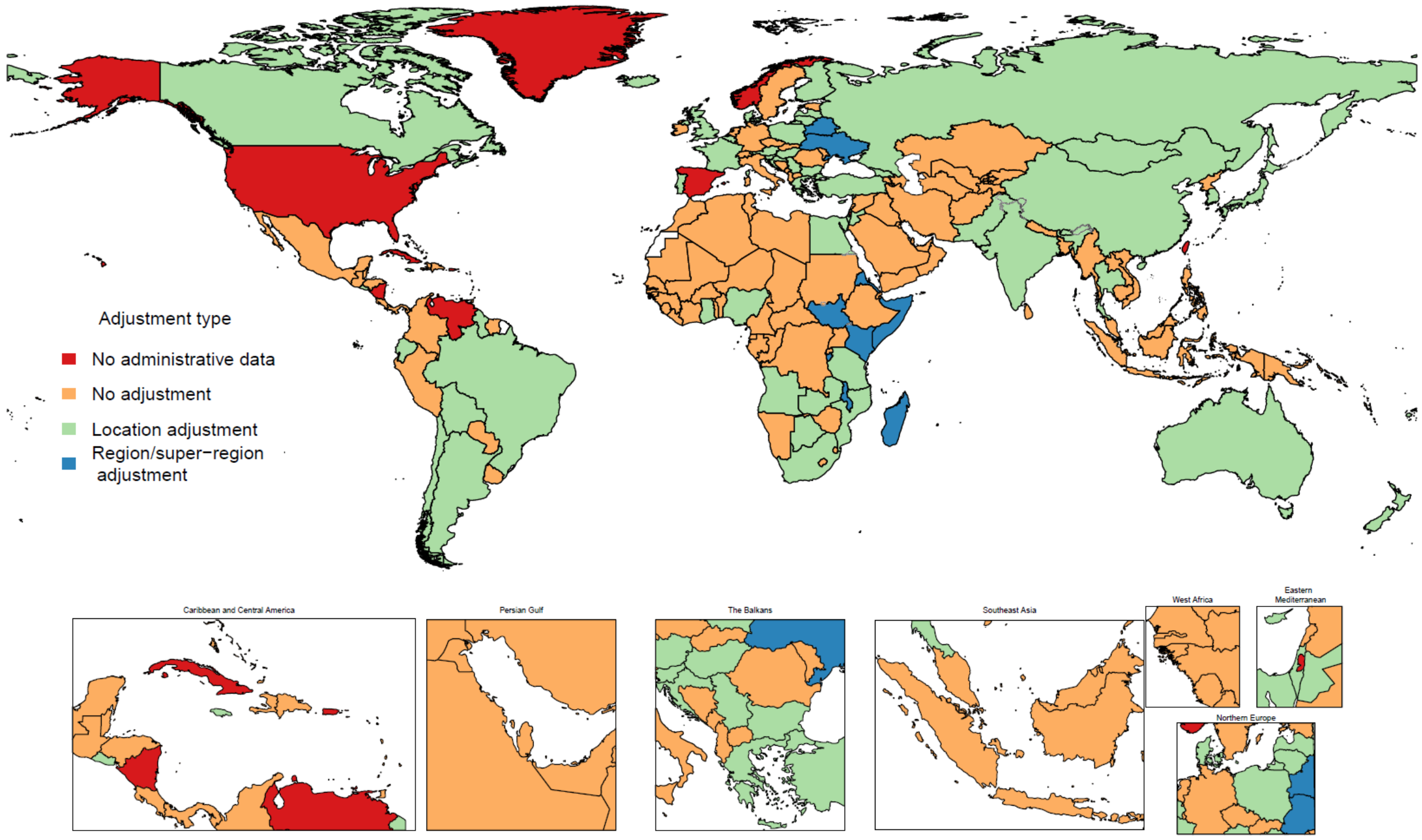

Appendix 1 Figure 6g: Adjustment type for administrative pharmaceutical assistant densities from WHO NHWA and Eurostat Health Database by cadre

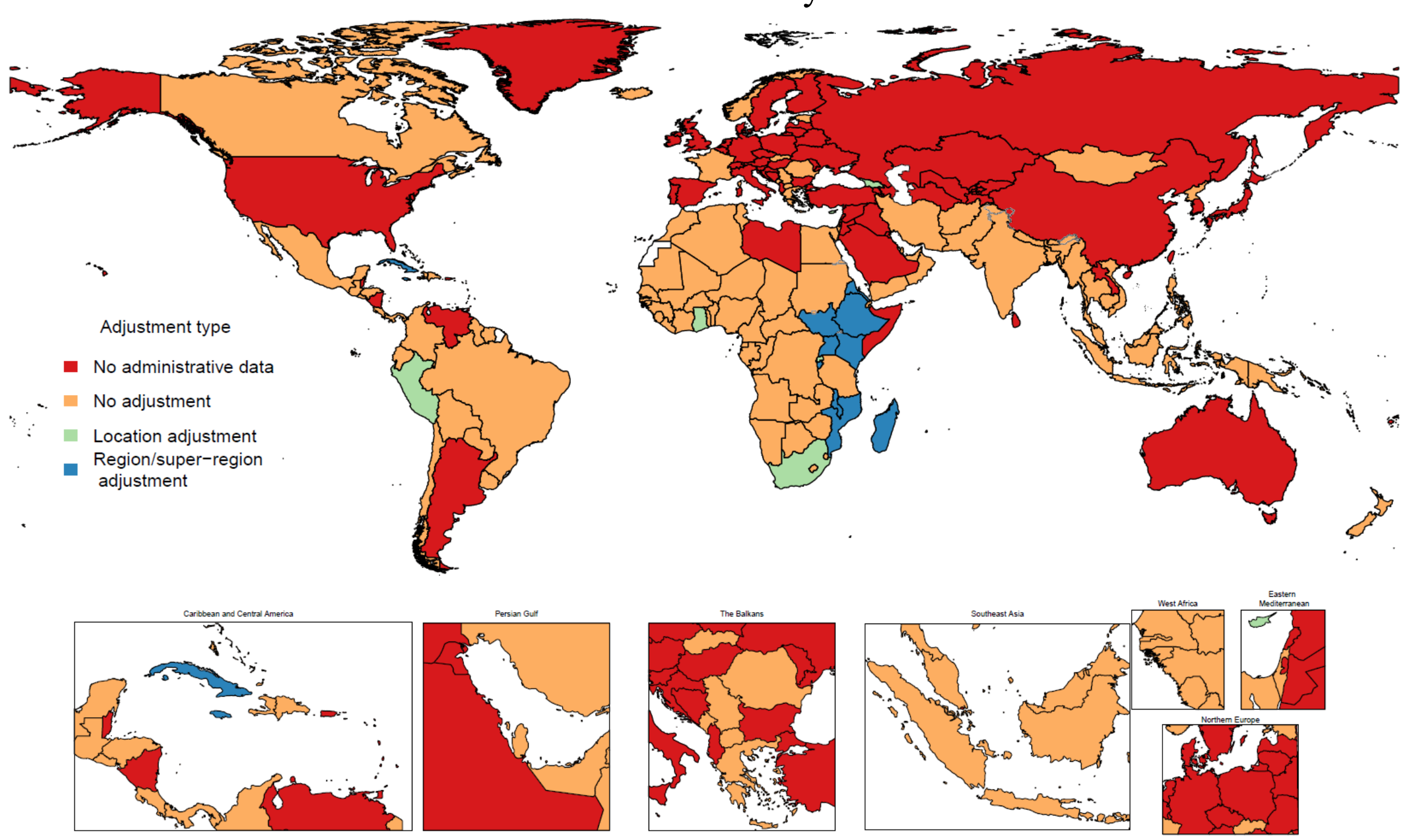

Appendix 1 Figure 7: Average percentage difference adjustment to administrative data from WHO NHWA and Eurostat Health Database by cadre

Appendix 1 Figure 7a: Average percentage difference adjustment to administrative doctor data from WHO NHWA and Eurostat Health Database

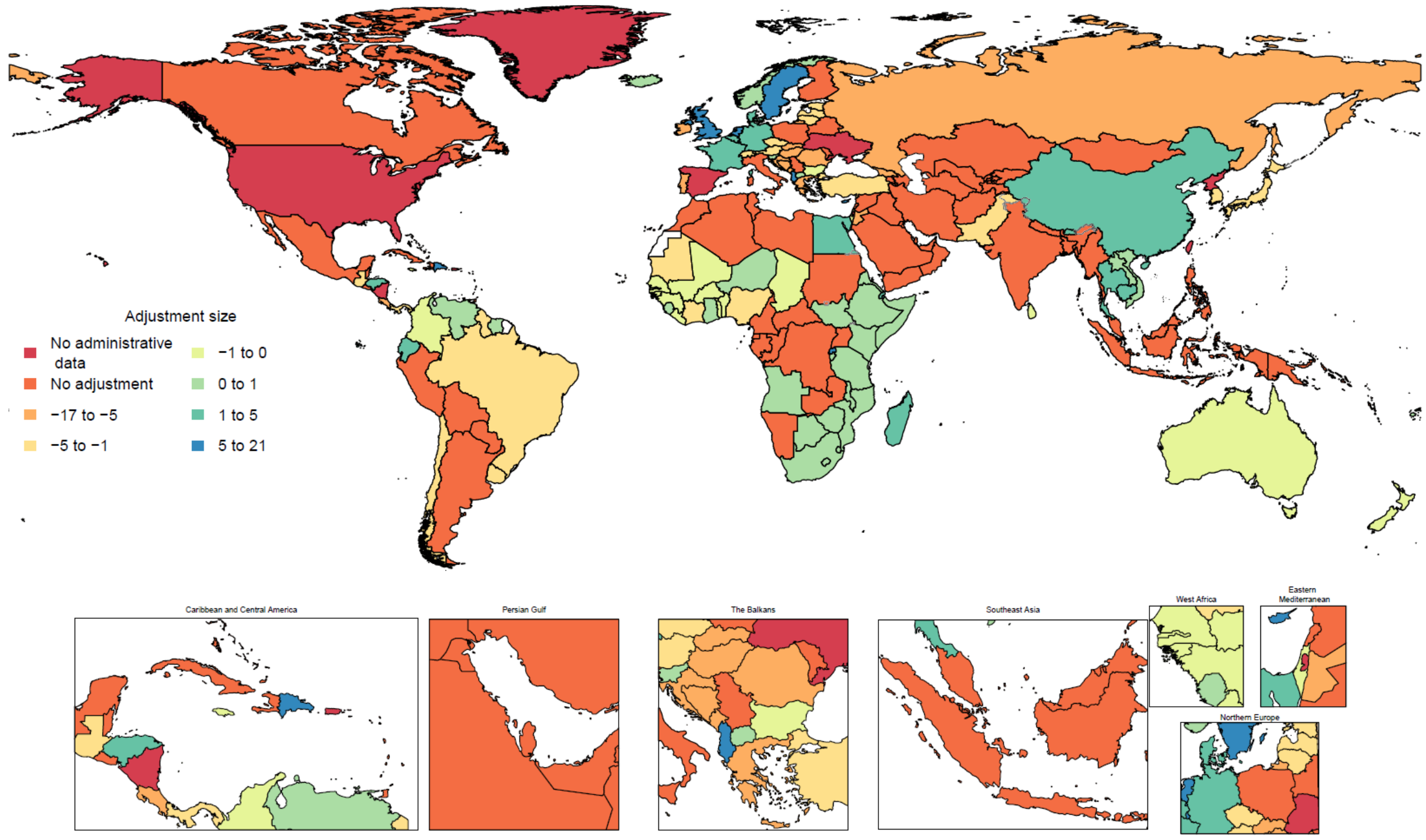

Appendix 1 Figure 7b: Average percentage difference adjustment to administrative nursing personnel data from WHO NHWA and Eurostat Health Database

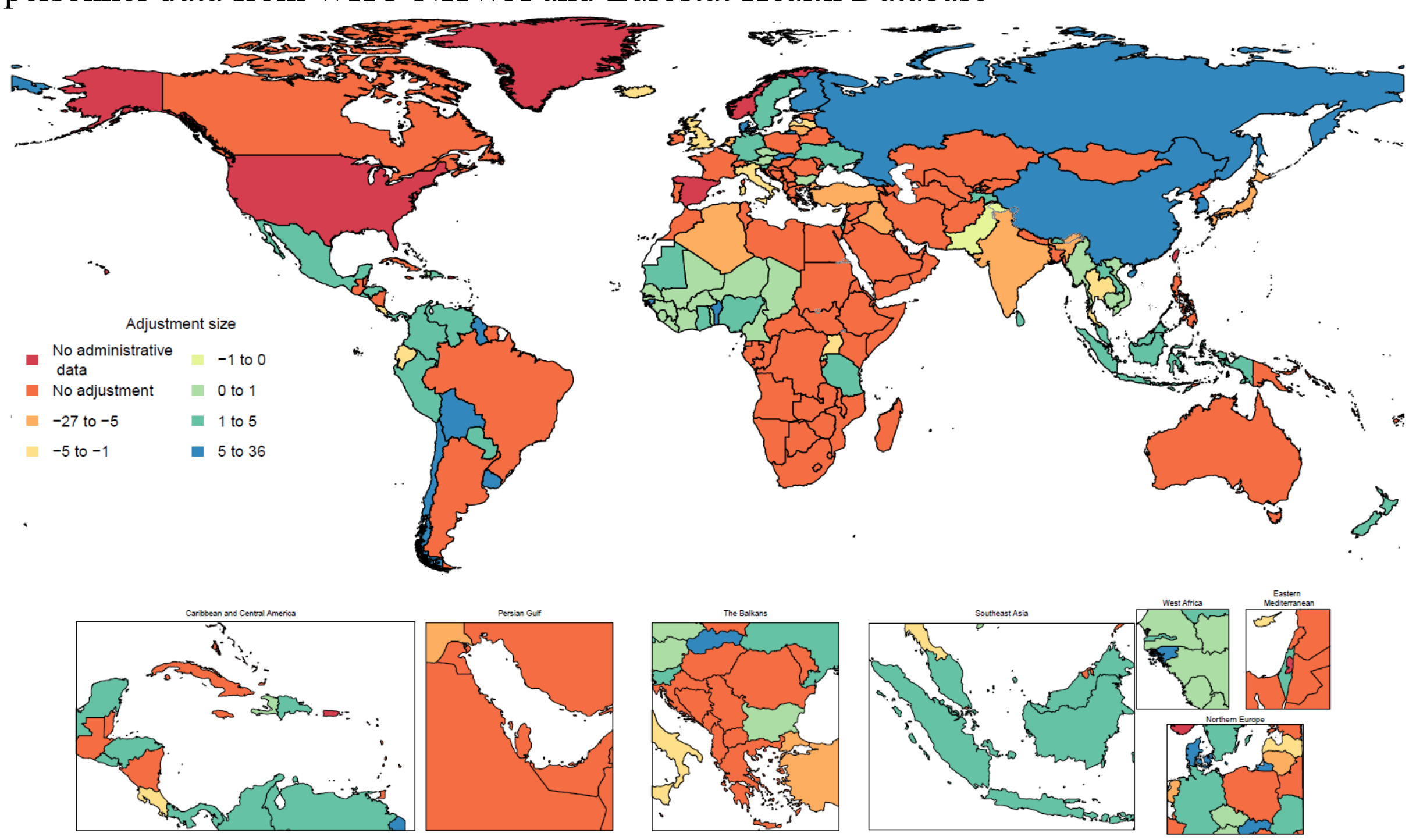

Appendix 1 Figure 7c: Average percentage difference adjustment to administrative midwifery personnel data from WHO NHWA and Eurostat Health Database

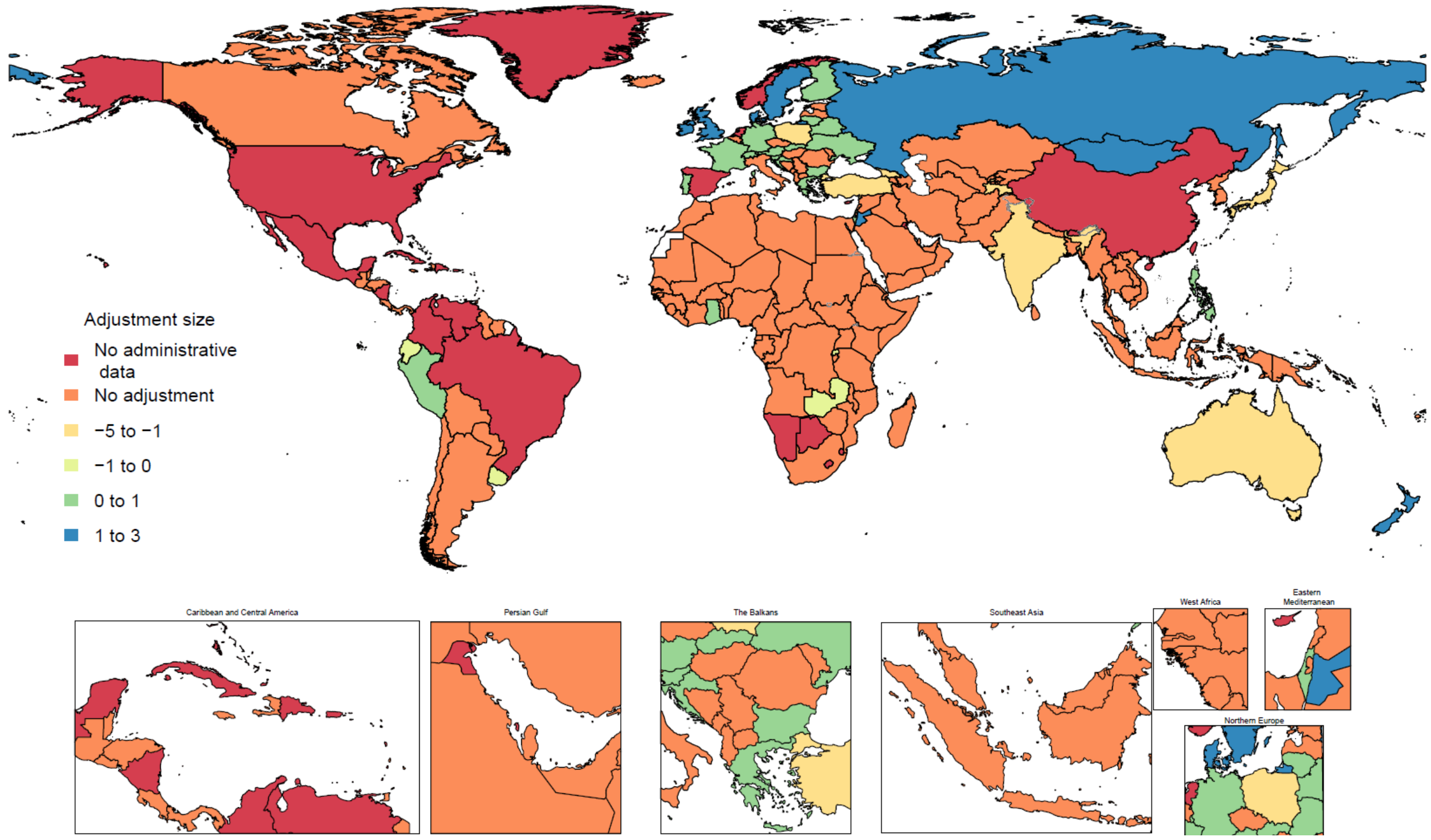

Appendix 1 Figure 7d: Average percentage difference adjustment to administrative dentist data from WHO NHWA and Eurostat Health Database

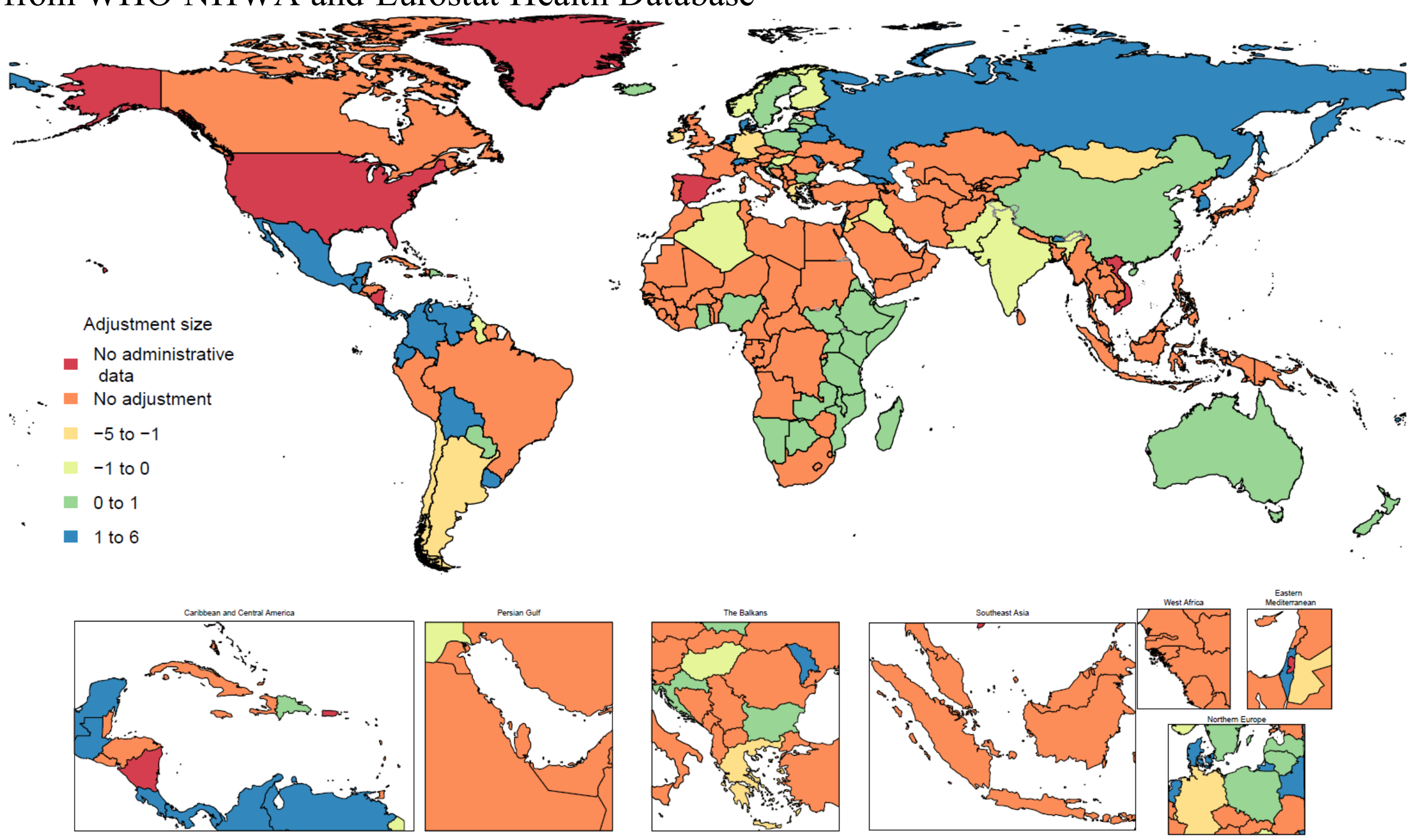

Appendix 1 Figure 7e: Average percentage difference adjustment to administrative dental assistant data from WHO NHWA and Eurostat Health Database

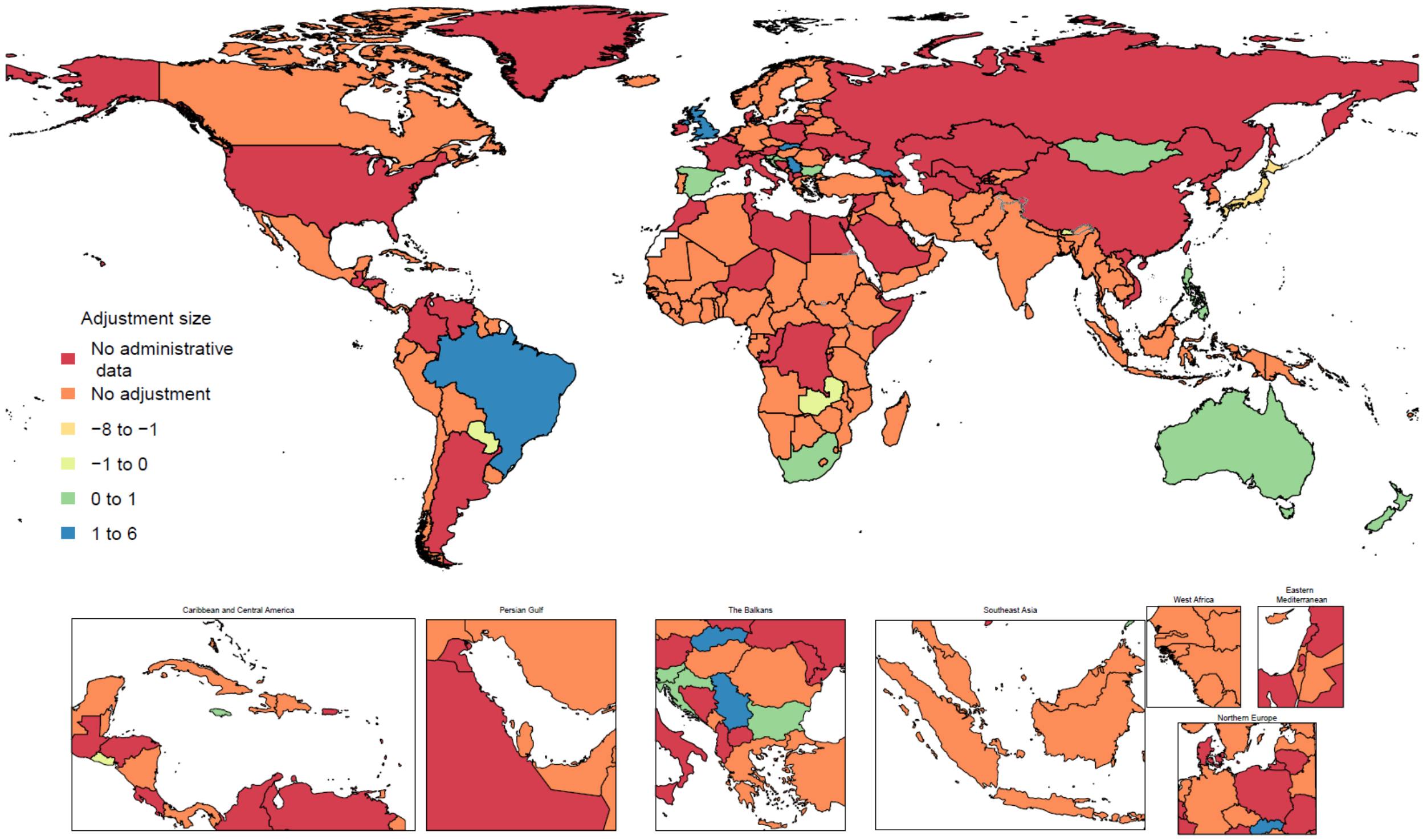

Appendix 1 Figure 7f: Average percentage difference adjustment to administrative pharmacist data from WHO NHWA and Eurostat Health Database

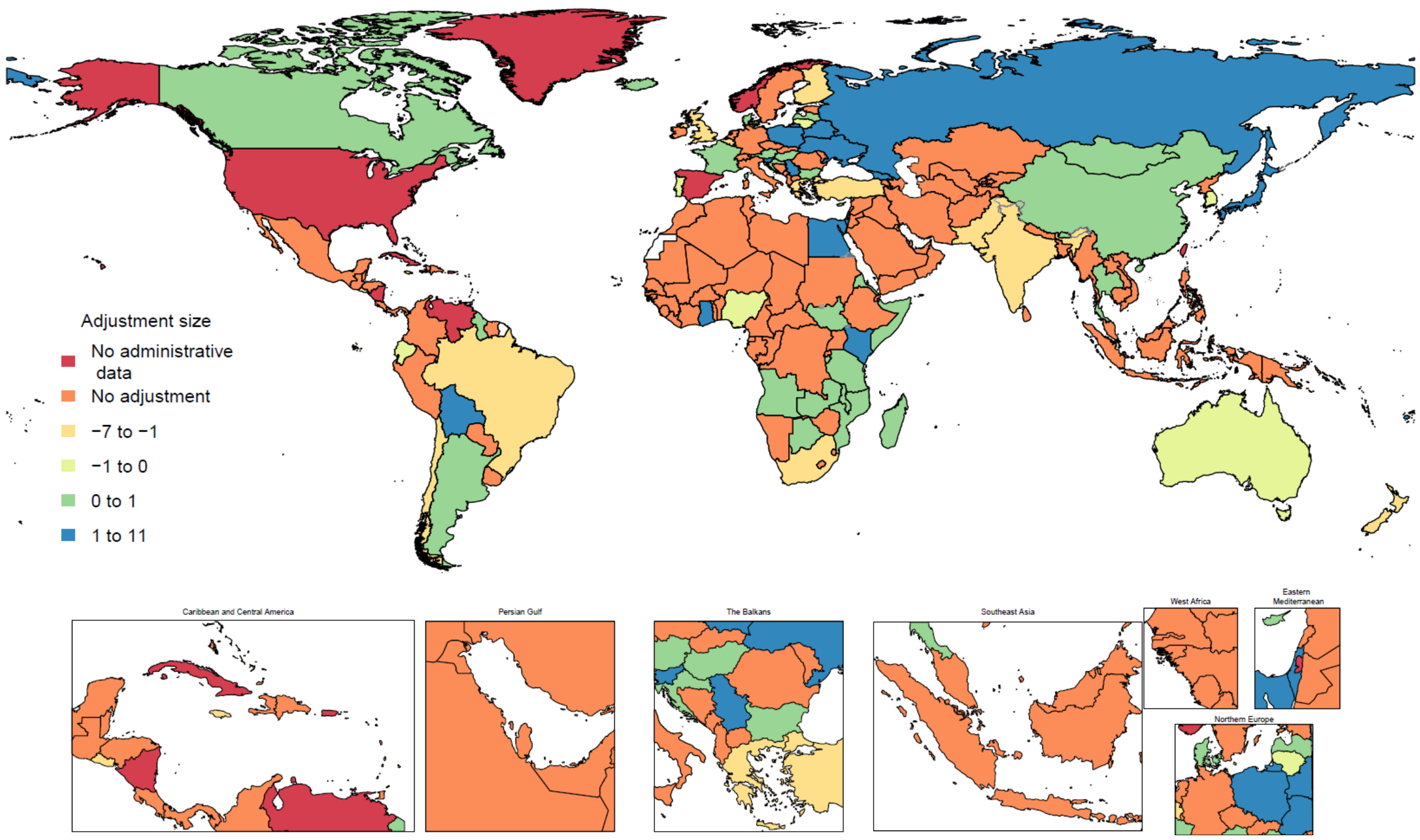

Appendix 1 Figure 7g: Average percentage difference adjustment to administrative pharmaceutical assistant data from WHO NHWA and Eurostat Health Database

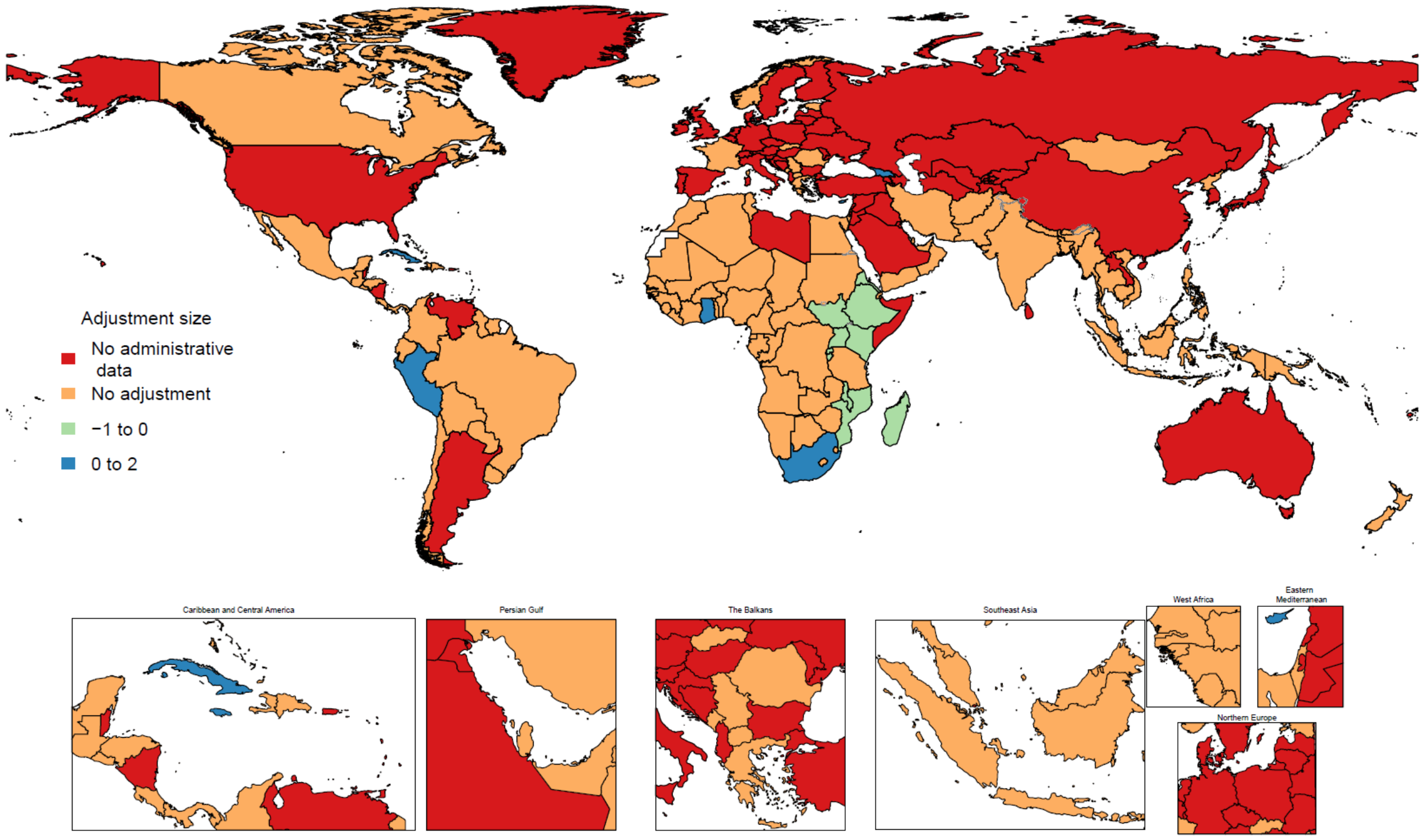

2.5 Modelling strategy

2.5.1 GBD machinery updates

To estimate health workers globally over time, this analysis used a three-stage spatiotemporal Gaussian process regression (ST-GPR). ST-GPR is a flexible modelling strategy that synthesises noisy data by incorporating covariates and borrowing strength across both geography and time to produce comprehensive time series estimates with corresponding uncertainty. ST-GPR has been used widely in GBD research and has been described in detail in other recent publications.[4,5]

2.5.2 Model specifications

ST-GPR was used to estimate densities and sex shares for all health worker cadres and the total health workforce, for the seven GBD super-regions, 21 GBD regions, and 204 countries and territories for the period of 1990 to 2023. Additionally, we modelled two other entities that are used as covariates in the health worker density and sex share models: portion professional workforce and employment-to-population ratios.

As stated in the main text, the first stage of ST-GPR fits a linear mixed effects regression to the data with fixed effects on specified covariates detailed in table 8 and random effects on location, region, and super-region.[16] All densities were log-transformed and all sex shares were logit-transformed prior to modelling. Using the predictions from the first-stage models, residuals are calculated for the second stage. In the second stage, a locally weighted polynomial regression smooths the residuals from the first stage across time and geography to generate a non-linear

trend that follows the available data. Where data are available, the second stage follows the data closely; it also adjudicates between data that disagree based on uncertainty. Where data are not available, the predictions from the second stage are informed by data in surrounding years in a given location, or by data in nearby geographies in the same time frame. The third stage uses the second-stage predictions as a mean function in a Gaussian process regression. Gaussian process regression further smooths the estimates over space and time, and accounts for input data variance, generating uncertainty in the final estimates. Predictive performance was assessed using in-sample and out-of-sample root mean squared error (RMSE) at each stage of the ST-GPR process for each cadre depicted in table 9. One key update on the use of ST-GPR in GBD 2023 is that, to reduce computing power and processing time, we decreased the number of draws (or computations) per process to 250, down from the 1000 draws used in previous rounds. This is consistent with the change in draws implemented more broadly as part of GBD 2023.

Appendix 1 Table 8: Covariates for ST-GPR models

Model	Covariates
**Sex shares**	
All health workers	SDI, female share of employment
Doctors	SDI, female share of workforce, female share of professional workforce
Nursing personnel	
Midwifery personnel	
Dental assistants	
Pharmacists	
Pharmaceutical assistant	
Dentists	SDI, female share of employment, THE pc (logged)
Community health workers	Female share of nurses
**Densities**	
All health workers	SDI, professional workforce, THE pc (logged), employment-to-population ratio
Doctors	SDI, professional workforce
Community health workers	
Nursing personnel	SDI, employment-to-population ratio
Midwifery personnel	
Dental assistants	
Medical laboratory technicians	
Dentists	SDI, professional workforce, employment-to-population ratio
Pharmacists	
Pharmaceutical assistant	
Clinical officers and medical assistants	
Optometrists and opticians	
Dieticians and nutritionists	
Medical imaging and therapeutic equipment technicians	
Audiologists, speech therapists, and counsellors	
Home-based personal care workers	SDI, THE pc (logged), employment-to-population ratio
Psychologists	
Physiotherapists and prosthetic technicians	
Traditional and complementary medicine practitioners	
Healthcare aides, paramedics, and ambulance workers	Professional workforce, THE pc (logged), employment-to-population ratio
Environmental health workers	
**Covariates**	

Employment ratio	GGE pc, age-standardised educational attainment pc, proportion of Muslim population
Professional workforce	SDI, age-standardised educational attainment pc, urbanicity

Notes: GGE=general government expenditure per capita. SDI=Socio-demographic Index. THE pc=total health expenditure per capita.

Appendix 1 Table 9: RMSE by ST-GPR modelling stage and cadre

Health worker cadre	Modelling Stage	In-sample RMSE	Out-of-sample RMSE
**Doctors**	Stage 1 (linear prior)	12.8	12.2
	Spacetime	5.7	7.4
	GPR	5.5	7.1
**Nursing personnel**	Stage 1 (linear prior)	47.6	29.4
	Spacetime	25.8	18.4
	GPR	25.7	17.1
**Pharmacists**	Stage 1 (linear prior)	4.8	3.7
	Spacetime	2.7	3
	GPR	2.6	3
**Dentists**	Stage 1 (linear prior)	4.1	3.4
	Spacetime	2	2.3
	GPR	2	2.3
**Midwifery personnel**	Stage 1 (linear prior)	5	4.3
	Spacetime	2.6	3.3
	GPR	2.1	3.2
**Dental assistants**	Stage 1 (linear prior)	8.4	4.3
	Spacetime	5	4
	GPR	4.8	3.9
**Pharmaceutical technicians**	Stage 1 (linear prior)	4	3.5
	Spacetime	3.3	3.2
	GPR	2.8	3.1
**Community health workers**	Stage 1 (linear prior)	10.2	29.8
	Spacetime	4.3	36.7
	GPR	3.4	36.8

To model each health worker cadre, we used the same model settings for intermediate and final estimates. Intermediate estimates, which were exclusively used in splitting, were run using only four-digit mapped censuses and surveys, while final estimates were run on all available data after three-digit codes had been split into the underlying four-digit cadres.

To evaluate model performance across an uneven data landscape, we examined residuals by computing, for each country, the average difference between the modelled density estimate and the input data for the main cadres of focus (Figures 8a–8h). These residuals show no systematic directional bias in data-sparse settings, including sub-Saharan Africa, indicating that gap-filling does not lead to consistent over- or under-estimation of density where data are limited.

Appendix 1 Figure 8a: Average modelled doctor density estimate minus input data by country

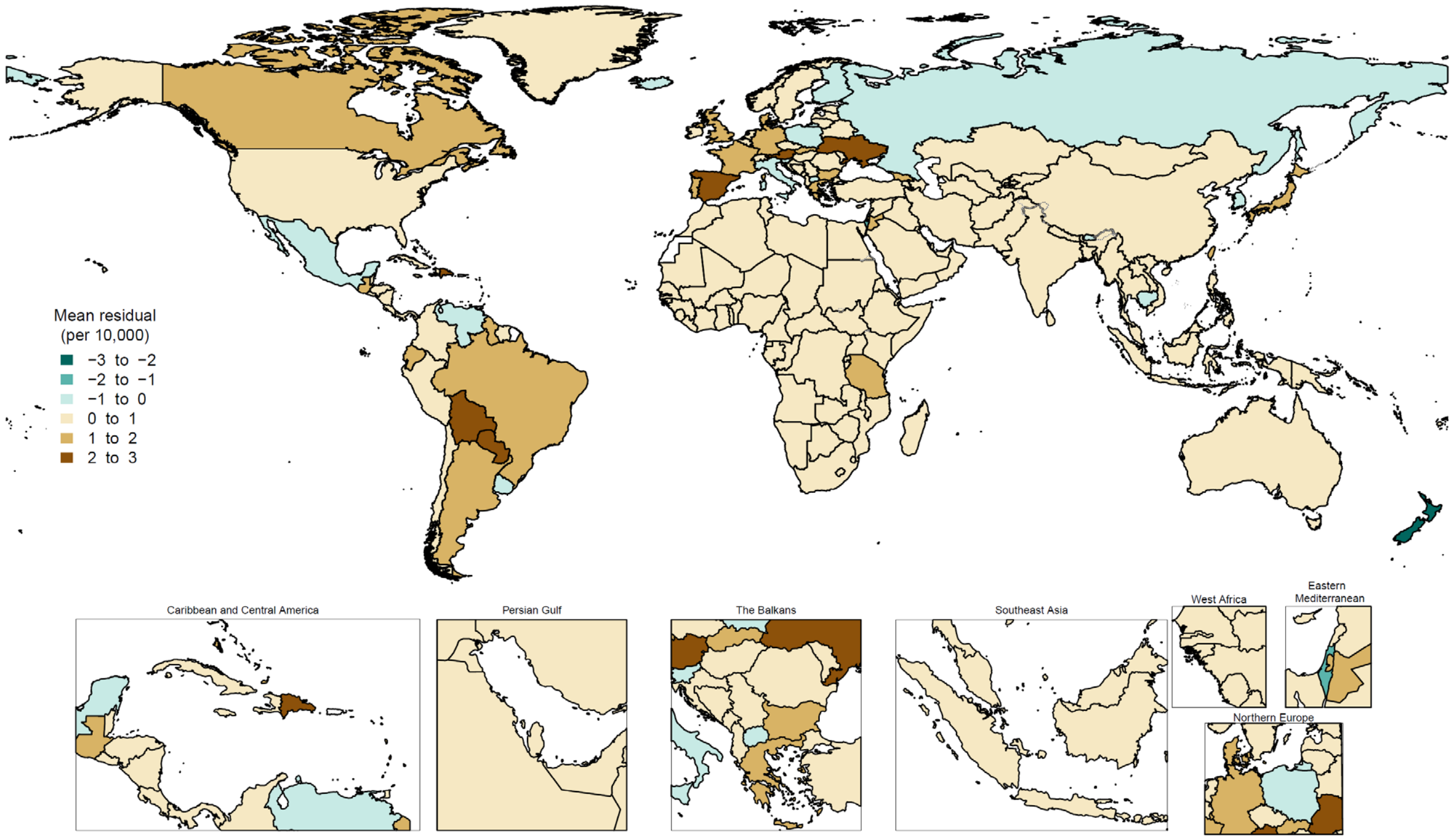

Appendix 1 Figure 8b: Average modelled nursing personnel density estimate minus input data by country

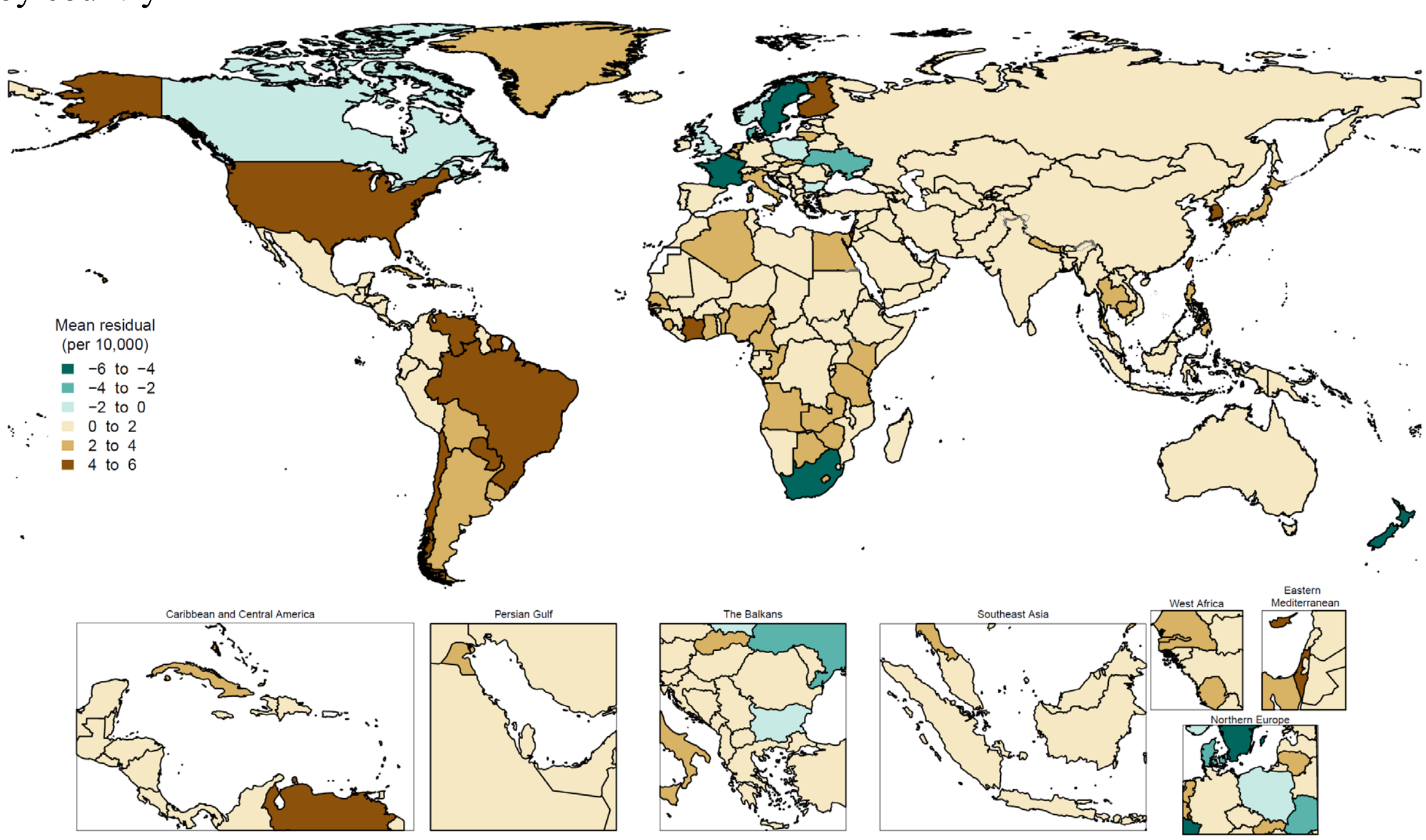

Appendix 1 Figure 8c: Average modelled midwifery personnel density estimate minus input data by country

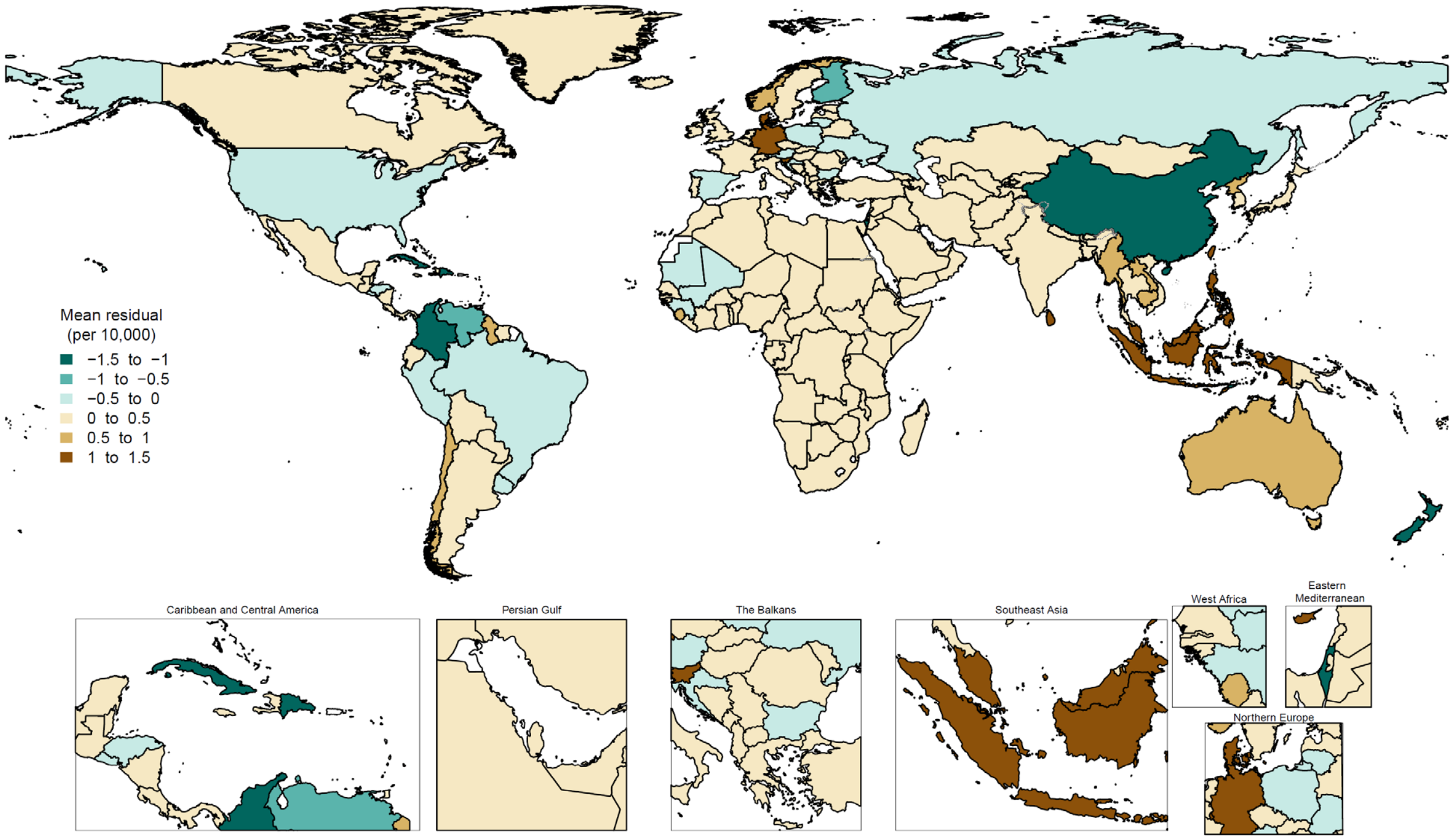

Appendix 1 Figure 8d: Average modelled dentist density estimate minus input data by country

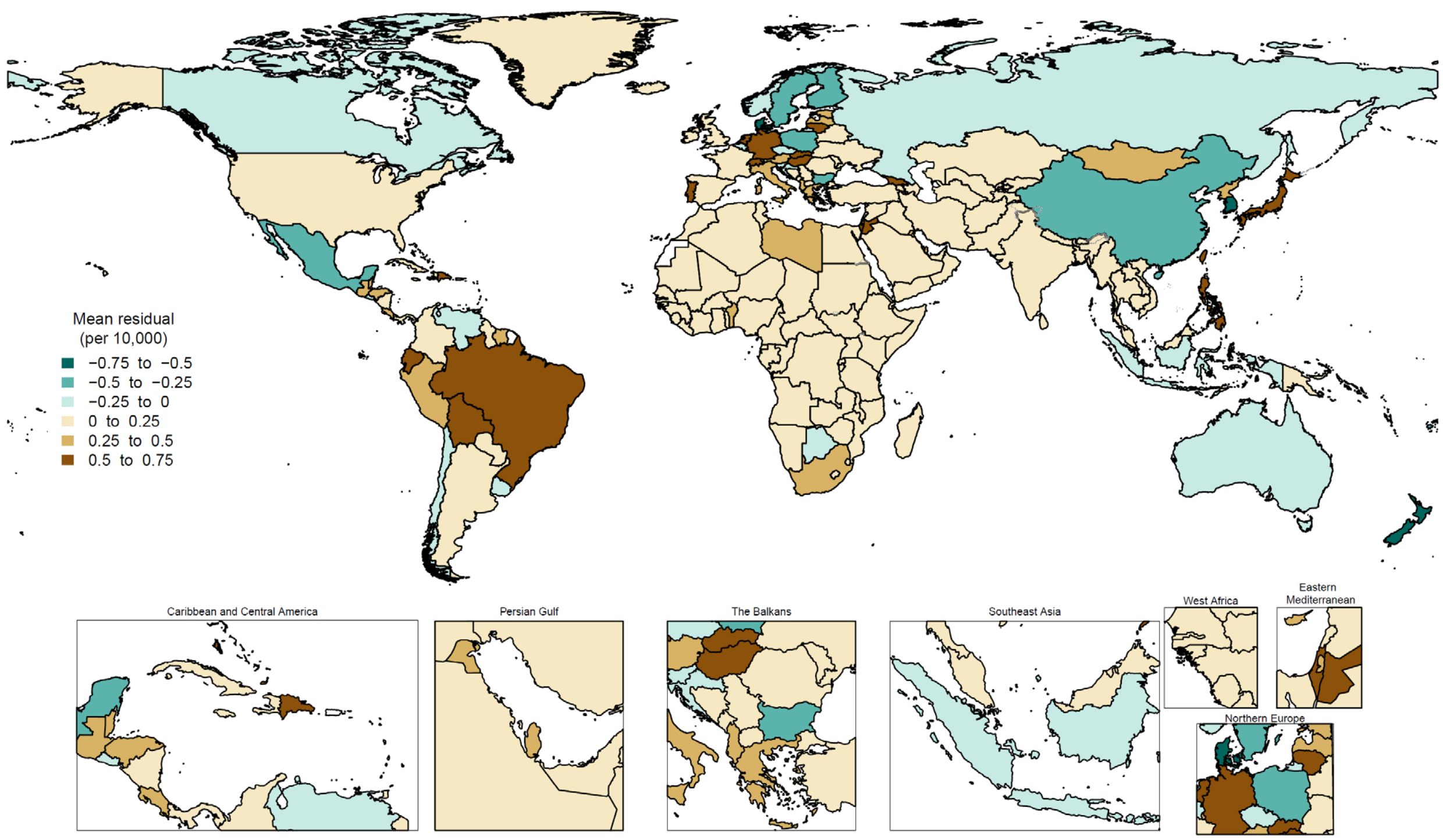

Appendix 1 Figure 8e: Average modelled pharmacist density estimate minus input data by country

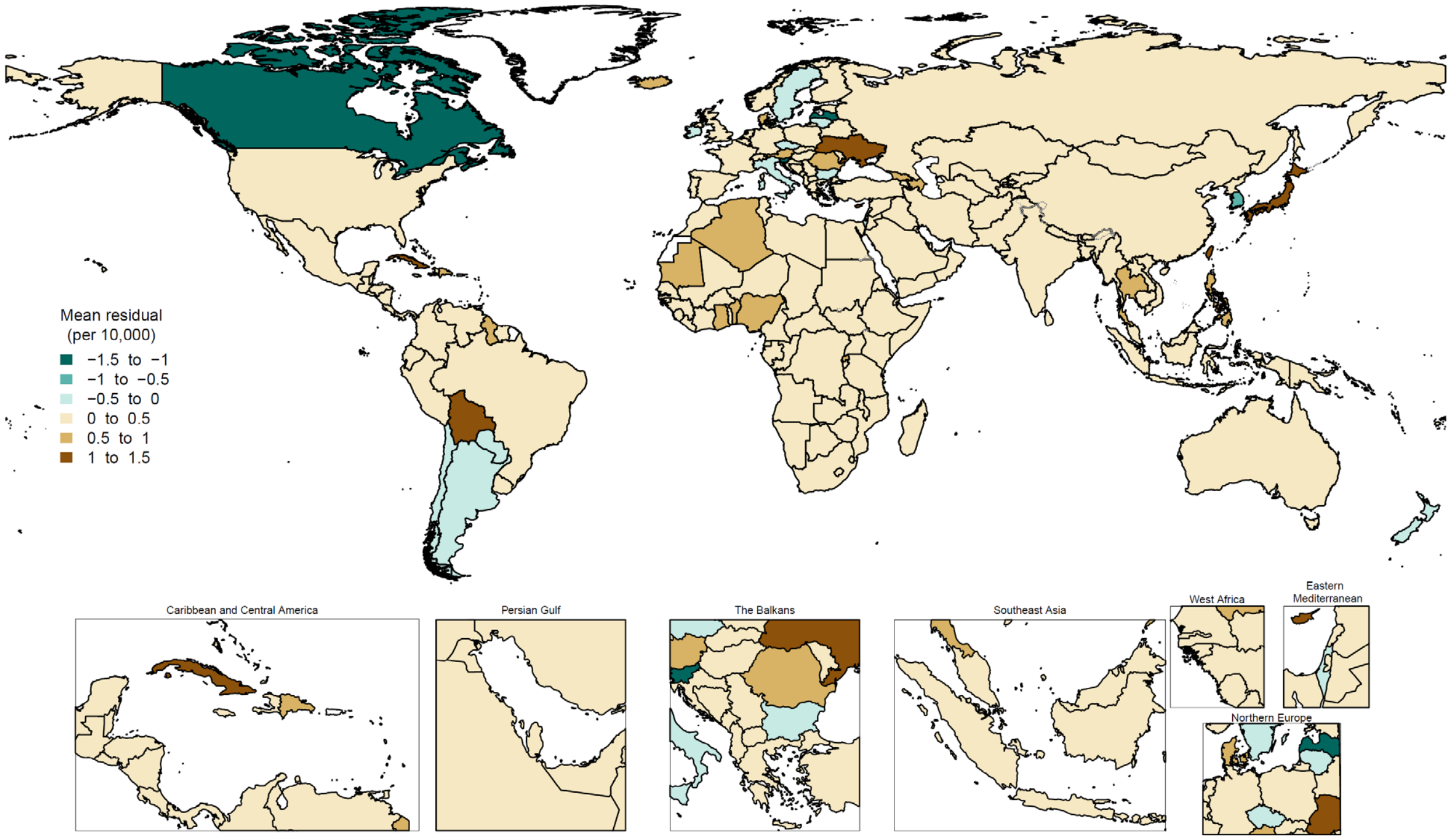

Appendix 1 Figure 8f: Average modelled community health worker density estimate minus input data by country

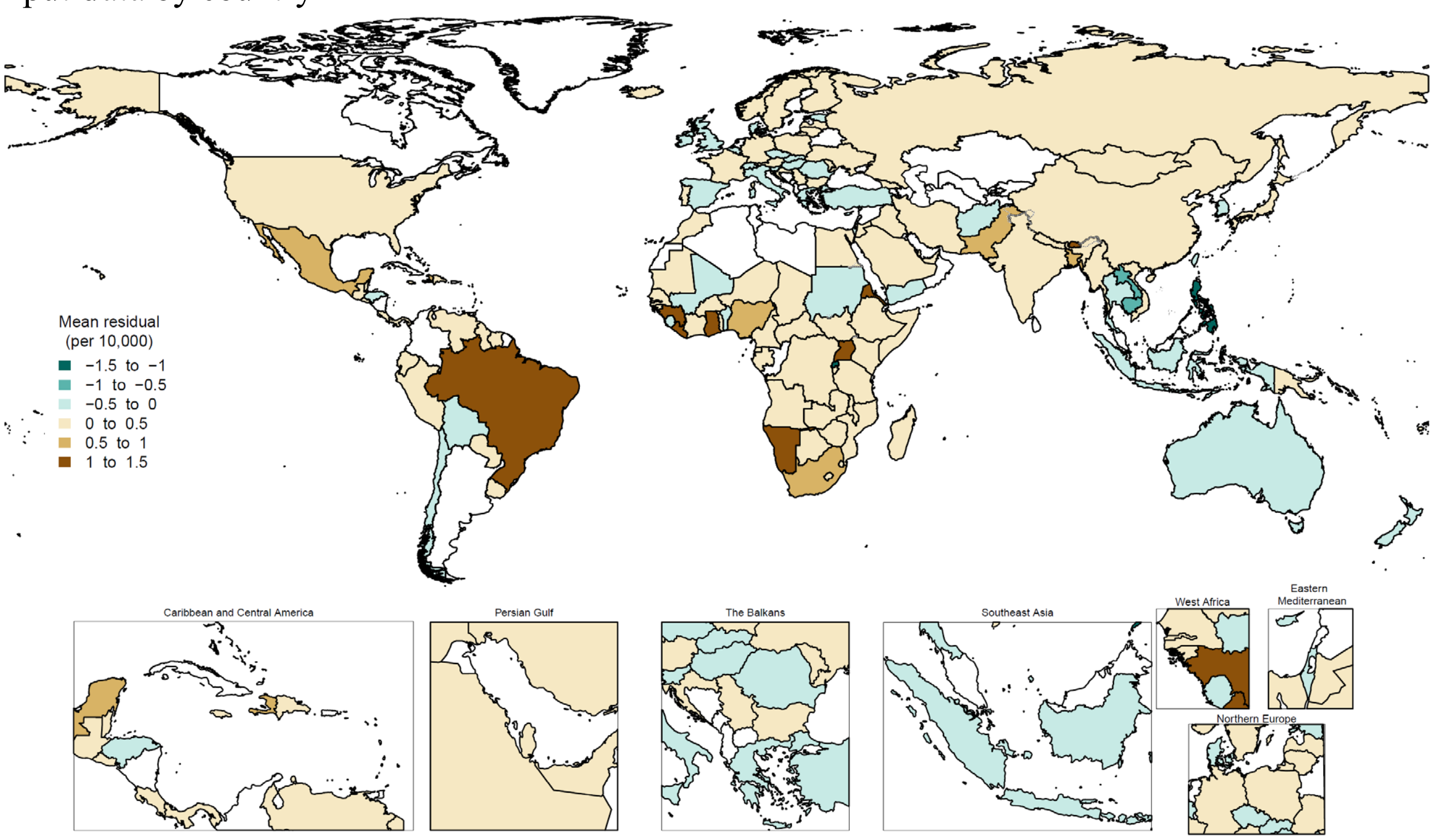

2.5.3 Post-processing of estimates

To avoid unreliable estimates stemming from variation in models of smaller cadres with fewer data, we raked the 12 cadres without administrative data available to the other, data-rich cadres. Raking here refers to the application of a rescaling factor to all cadre-specific estimates to ensure they summed to the envelope category of other health workers. The 12 cadres that underwent raking, also referred to together as "other HRH" in the manuscript, are as follows: clinical officers and medical assistants, physiotherapists and prosthetic technicians, medical imaging and therapeutic equipment technicians, medical laboratory technicians, health-care aides and ambulance workers, environmental health workers, optometrists and opticians, dieticians and nutritionists, audiologists, speech therapists, and counsellors, psychologists, home-based personal care workers, traditional and complementary practitioners. To calculate the other HRH envelope, we subtracted the sum of the eight cadres with higher data density (doctors, nursing personnel, midwifery personnel, dentists, dental assistants, pharmacists, pharmaceutical assistants, community health workers) from preliminary estimates of all health worker densities, by draw. More succinctly, "other HRH" refers to the quantity resulting from total HRH minus those eight most data-rich cadres. When after subtraction that residual quantity fell below zero, that data point was removed from the dataset because it indicated that data quality and estimation in the preliminary model were poor. Using this non-negative set of estimates, other HRH was modelled for a final time in ST-GPR for temporal and geospatial smoothing and to produce final estimates of other health workers per 10,000 population. The sum of the 12 data-scarce cadres was then scaled to the final other HRH estimate to produce final estimates of these 12 cadres among countries with no or limited (less than 25 country-years) of input data where scaling demonstrated more consistency with available raw inputs. For locations with sufficient data coverage (more than 25 country-years) or where scaling resulted in estimates substantially mis-aligned with the raw inputs available, the sum of the 12 cadres was not scaled.

After scaling was implemented, we aggregated cadres of interest and calculated sex-specific health worker densities and counts. Estimates were combined at the draw level. We multiplied estimates of female share of workers by cadre to density estimates by cadre at the draw level to produce sex-specific health worker densities for each cadre. To construct an uncertainty interval, final estimates were summarised using the mean and the 2.5^{th} and 97.5^{th} percentile of 250 draws.

Section 3. Improvements from GBD 2019

We would like to highlight some of the key improvements made in estimation of health workers relative to GBD 2019. Improvements relate to data processing and adjustment along with the post-processing of estimates. First, our processing of NHWA administrative data has been updated to reflect densities among all ages rather than working ages. Extractions of population-based microdata sources are now processed to describe health worker densities among working-age populations rather than employed populations. We continue to be unable to directly calculate densities among all ages directly for this data type because most of our labor force surveys are constrained to ages 15 to 69. We made this change to rely on more stable estimates, using the share of the population ages 15 to 69, rather than the more volatile employment-to-population ratios to convert densities from population-based sources to describe the all-age population for modelling. This shift in the denominator also insulated our HRH estimates from the substantial employment disruptions observed during the COVID-19 pandemic (for example, in the tourism

sector), which would have otherwise introduced sharp declines in HRH in 2020 that were not consistent with the existing evidence on health workforce employment during this time.[17,18]

We also further restricted the inclusion criteria for matched pairs to inform correction factors. Previously, we used data that were the result of our splitting process. Using these data had the potential to overstate the amount of information informing correction factors. In our updated crosswalks, we removed split data, instead using only data that originate with four-digit codes.

Finally, we updated our squeezing approach to avoid scaling data-rich cadres to entities with much less data. Previously, we had squeezed all cadres to the total health worker envelope. This was changed in GBD 2023 to better leverage the expansive data available for doctors, nurses, midwives, dentists and pharmacists. All other modelled entities, including the total health workforce, have many fewer data sources. In our updated approach, data-rich entities are only influenced by the data available specifically for them. We keep these entities stable and instead calculate a total health worker envelope that incorporates those estimates and squeezes all remaining cadres to the remainder of health workers once the data-rich entities have been taken into account (the other health worker category) in settings with no or limited input data that aligns with the approach. Our updated approach ensures consistency with total HRH across cadres while also prioritising the cadres informed by the most data.

These improvements, along with the hundreds of data sources added to the analysis, resulted in substantial changes in densities in some countries. Comparisons of estimates from GBD 2019 and GBD 2023 for the main cadres of focus in the study are depicted in figures 8a-8e.

Appendix 1 Figure 8: Comparison of health worker densities for select cadres from GBD 2019 and GBD 2023 in 1990, 2000, 2010, and 2019

Appendix 1 Figure 8a: Comparison of doctor densities from GBD 2019 and GBD 2023 in 1990, 2000, 2010, and 2019

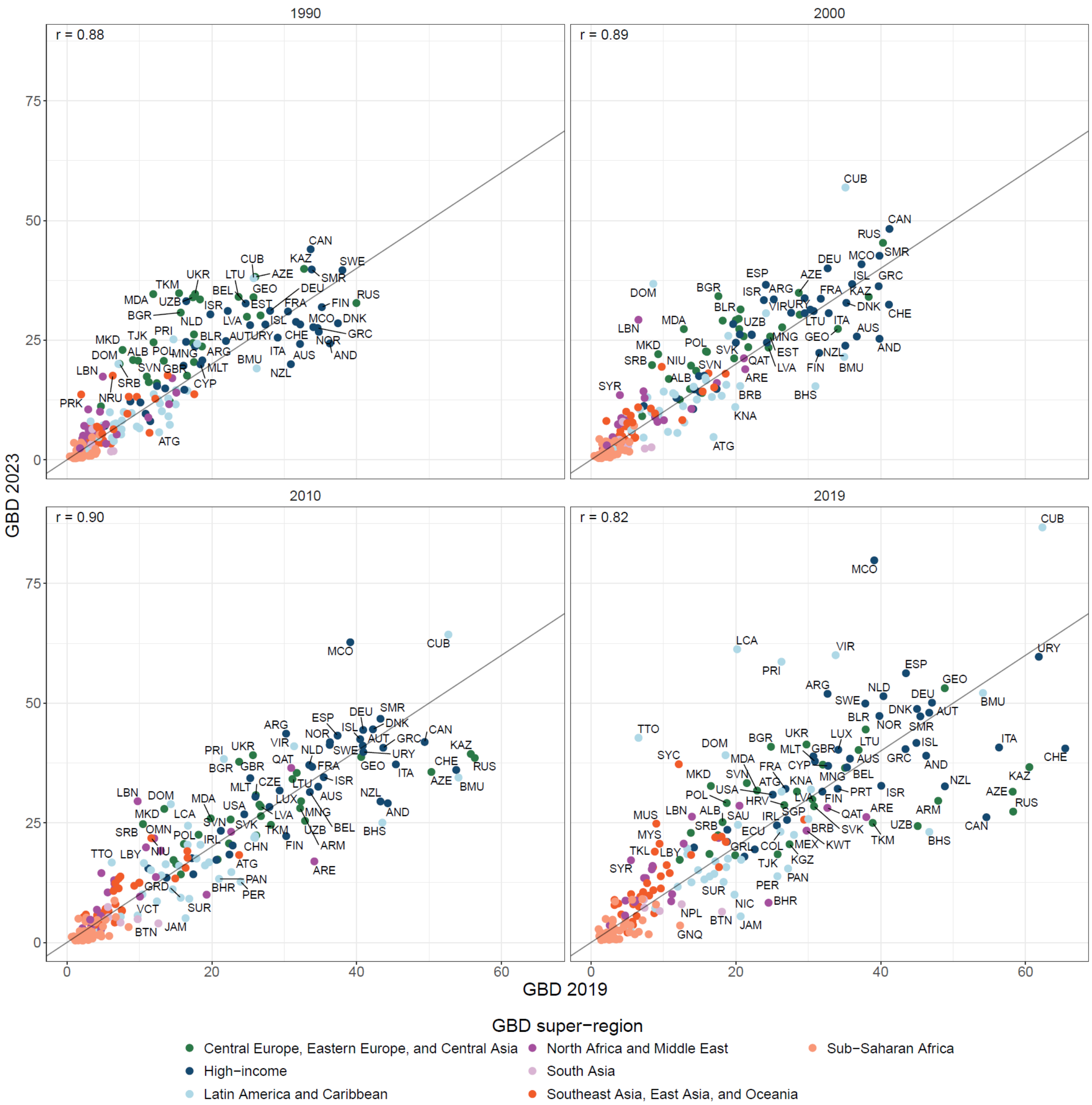

Appendix 1 Figure 8b: Comparison of nursing and midwifery personnel densities from GBD 2019 and GBD 2023 in 1990, 2000, 2010, and 2019

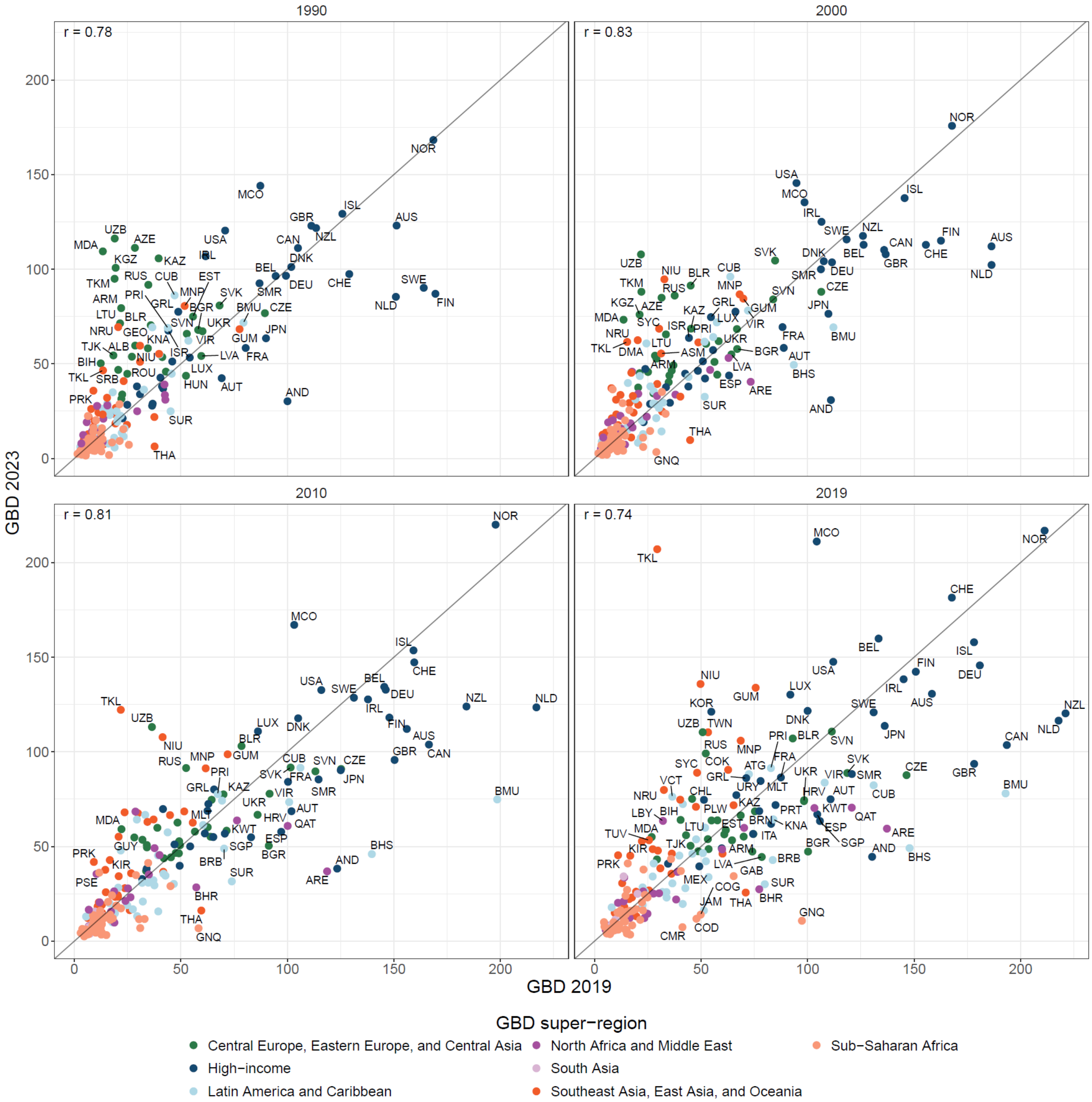

Appendix 1 Figure 8c: Comparison of dentistry personnel densities from GBD 2019 and GBD 2023

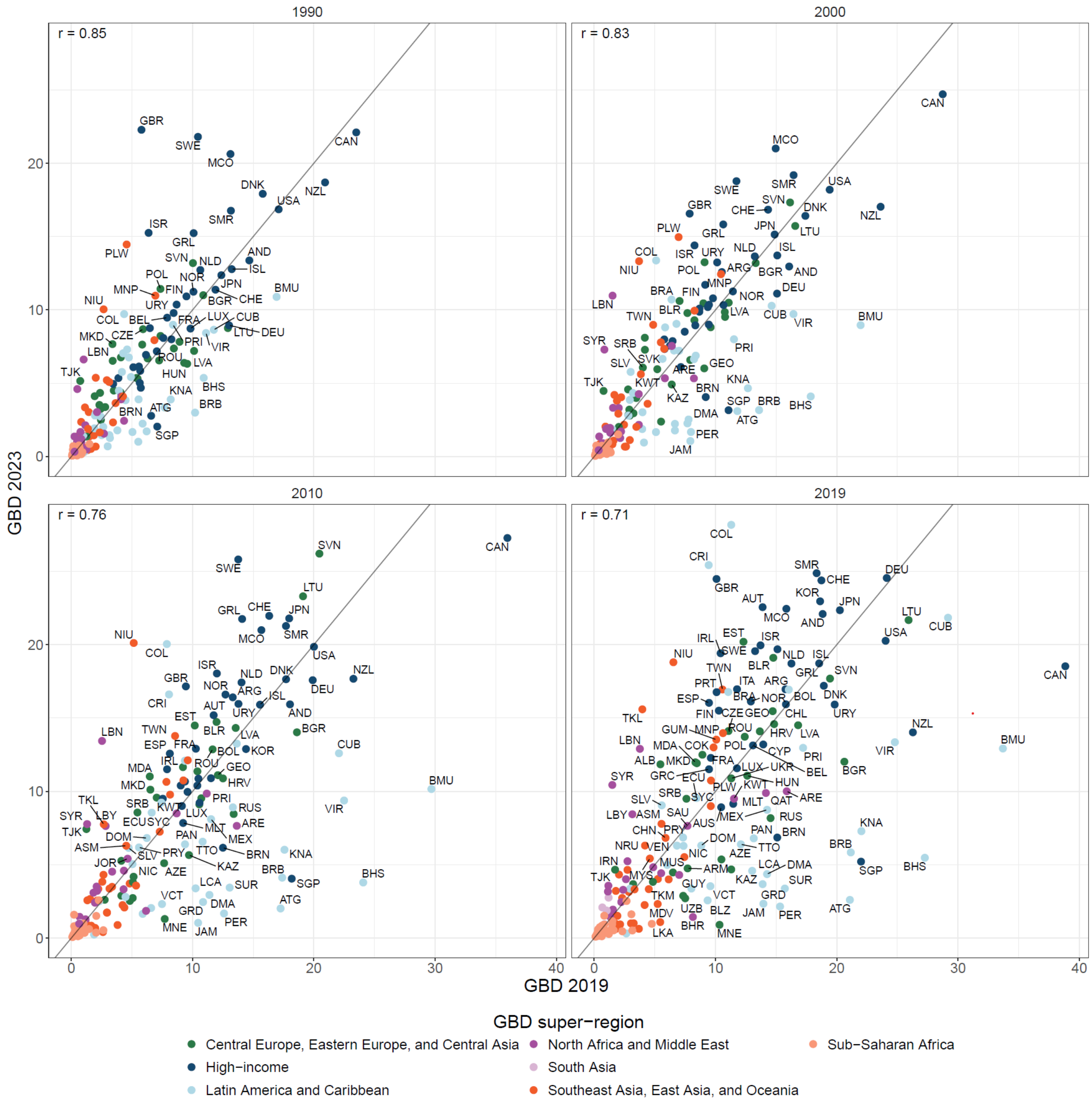

Appendix 1 Figure 8d: Comparison of pharmaceutical personnel densities from GBD 2019 and GBD 2023 in 1990, 2000, 2010, and 2019

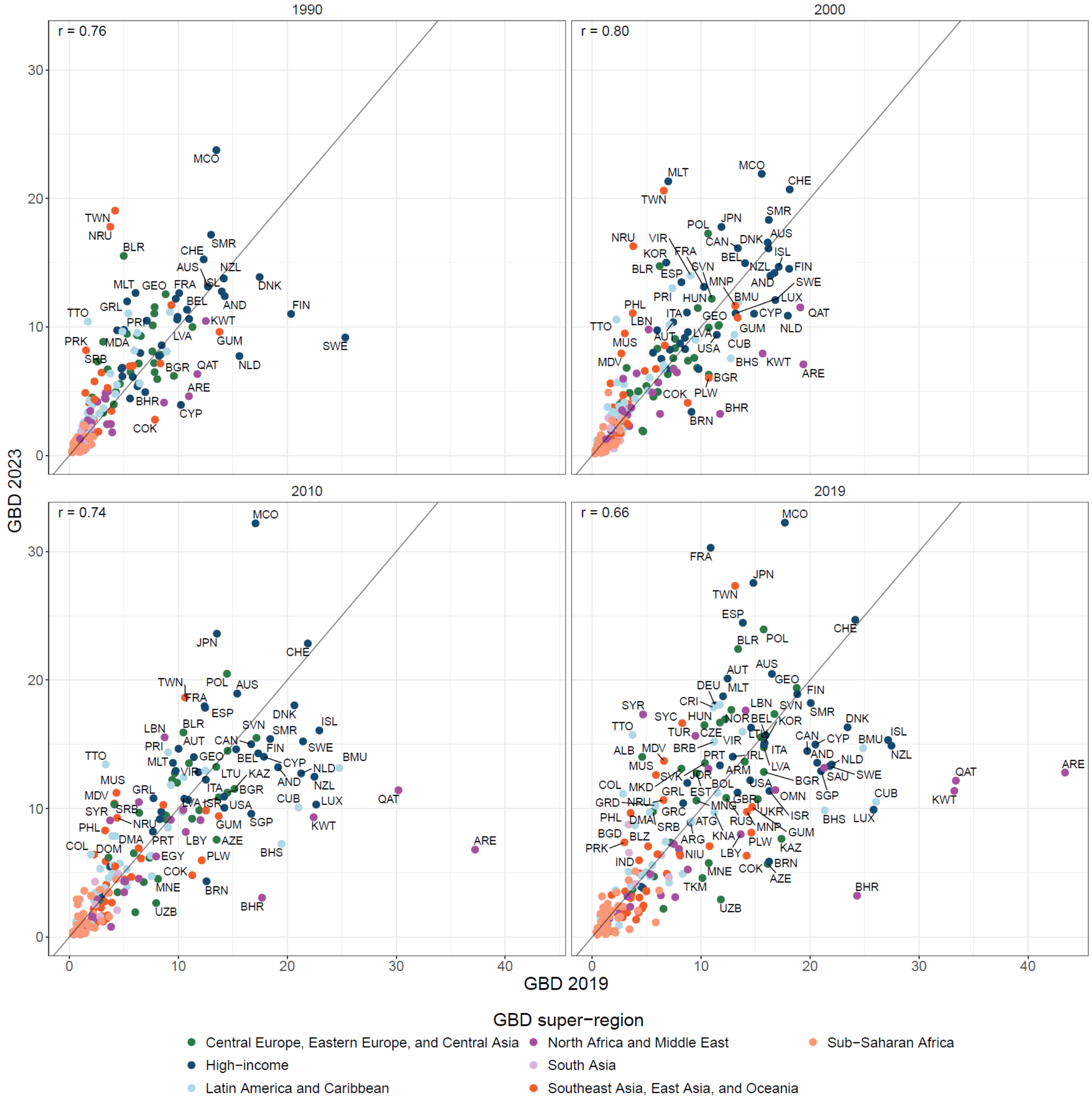

Appendix 1 Figure 8e: Comparison of all health worker densities from GBD 2019 and GBD 2023

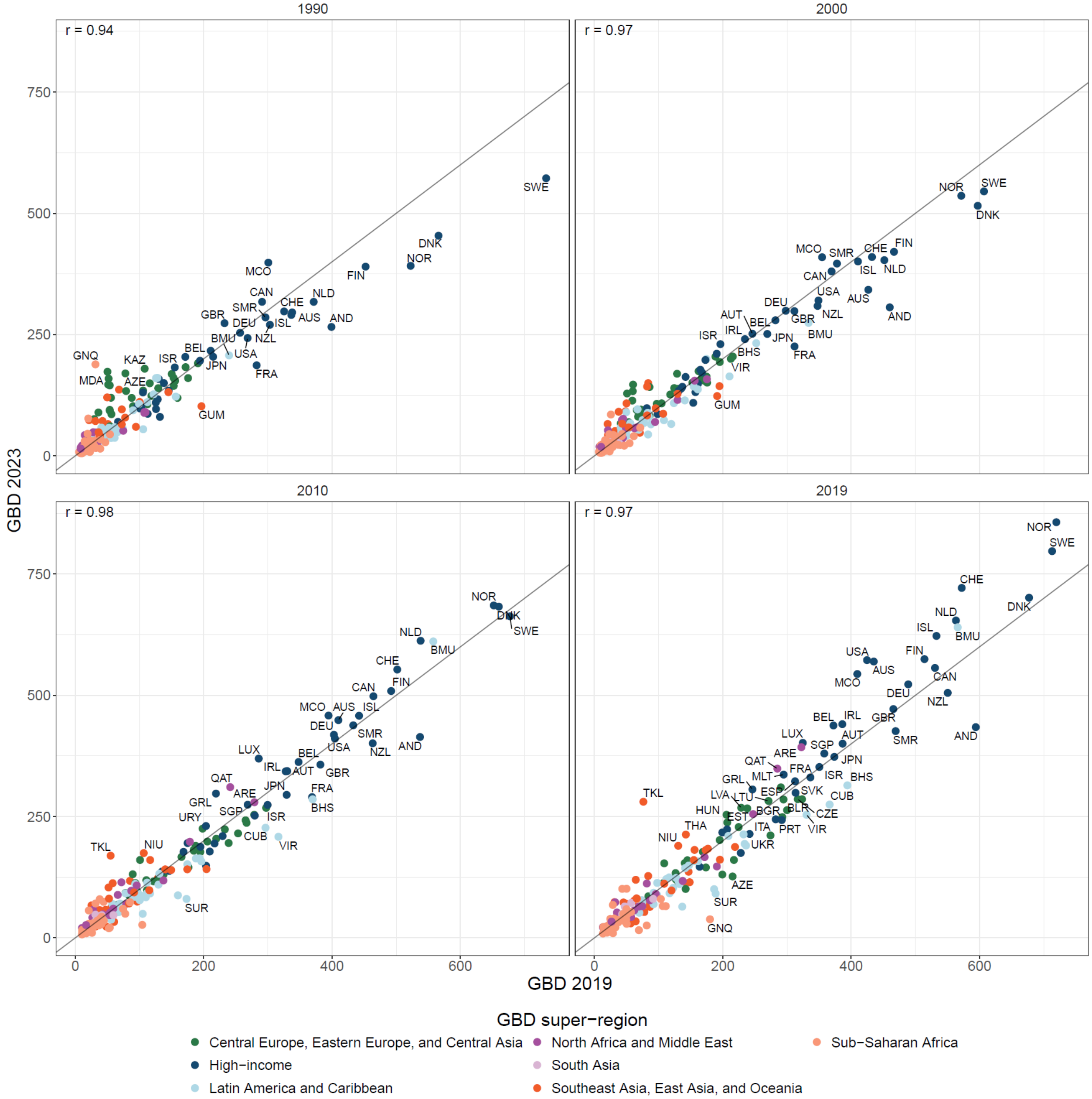

Section 4. Estimating the relationship between health worker densities and universal health coverage[2]

4.1 Previous thresholds

In the 2006 World Health Report, WHO identified 22.8 doctors, nurses, or midwives per 10 000 population as a minimum health workforce threshold.[19] This figure was based on the average number of health workers observed in countries achieving skilled birth attendance above 80%. In 2016, WHO revised this number, suggesting that 44.5 doctors, nurses, or midwives per 10 000 population were needed.[20] This second threshold was based on skilled health worker densities associated with global median achievement on an index composed of 12 SDG indicators. These threshold estimates were used widely, particularly in relation to the Millennium Development Goals and later the Sustainable Development Goals. Most recently, in 2023, WHO used the global median density of doctors, nurses, and midwives as a threshold (49 per 10,000) to describe locations with the most pressing health workforce challenges.[21]

WHO estimates of health workforce shortages have several shortcomings. First, these thresholds are based on datasets that were not produced using standardised methods, but rather compiled predominantly from government reports that used varying data-collection methods and cadre definitions. WHO researchers engage in triangulation efforts to standardise these heterogeneous inputs to available censuses and other gold standards, though WHO HRH datasets still exhibit discrepancies between sources of different types for the same location in similar years. Second, WHO thresholds identify minimum health worker levels in the aggregate, rather than providing cadre-specific health worker thresholds. In other words, the WHO thresholds do not identify needs for specific cadres and imply a 1:1 substitutability between cadres within their aggregate threshold. Third, WHO thresholds rely on a limited measure of health-care performance. The 12 SDG indicators used to fit the WHO threshold all reflect crude coverage rather than effective coverage and therefore do not incorporate the quality or effectiveness of the services provided. They are also heavily skewed toward interventions pertaining to maternal, neonatal, and communicable conditions rather than non-communicable conditions, for which disease burdens are on the rise in most parts of the world.[4,5] Two of the indicators also pertain to risk factors, such as the prevalence of tobacco smoking, which largely fall outside of the direct influence of health system interventions. For these reasons, the WHO thresholds may not accurately capture the broad range of health-care services on which HRH levels are likely to have an impact.

4.2 Universal health coverage (UHC) effective coverage index

We measured health system performance using the universal health coverage (UHC) effective coverage index estimated during the GBD 2023 estimation cycle and first developed as part of GBD 2019. The index leverages a variety of estimates related to disease burden and intervention coverage from GBD 2023. It was intended to improve upon the UHC service coverage index proposed in the GBD 2017 round,[2] and followed measurement guidance from consultations with the WHO General Programme of Work 13 Expert Reference Group. Further details on the construction of the GBD 2019 effective coverage index, which was replicated for GBD 2023, are available in the published GBD 2019 manuscript.[3]

We briefly describe the UHC effective coverage index here. This index quantifies on a scale of 0 to 100 the availability, use, and quality of essential health services for a number of service areas (promotion, prevention, and treatment) applicable to a range of ages across the lifespan. The index is composed of 23 tracer indicators, four of which directly measure intervention coverage and 19 of which, in the absence of reliable effective coverage data, are indirect proxies for access to quality care based on disease outcomes. The four coverage indicators encompass met need for modern contraception, DTP3 immunisations, MCV1 immunisations, and ART coverage. The 19 outcome-based indicators are related to antenatal, peripartum, and postnatal care for mothers and neonates and treatment for the following conditions: lower respiratory infections, diarrhoea, tuberculosis, leukaemia, asthma, epilepsy, appendicitis, paralytic ileus and intestinal obstruction, diabetes, ischaemic heart disease, stroke, chronic kidney disease, chronic obstructive pulmonary disease, cervical cancer, breast cancer, uterine cancer, and colon/rectum cancer. These indicators were each associated with one or more age groups to which existing health system interventions are applicable. The following age groups were used: reproductive and newborn age spans, children under age 5 years, children and adolescents ages 5–19 years, adults ages 20–64 years, and older adults at least 65 years of age. For every location, year, and age group, each indicator was scored using estimates pertaining to the corresponding population (eg, one score for chronic kidney disease treatment among adults ages 20–64 in Thailand in 2005).

Scores for the four health service coverage indicators came directly from estimates of coverage percentages. For the 19 outcome-based proxy measures, scores came from mortality-to-incidence ratios, mortality-to-prevalence ratios (for longer-term conditions), risk-standardised deaths, or other mortality measures like maternal mortality ratios, all of which strive to capture the degree to which health system activities successfully prevent adverse outcomes. These proxy measures were scaled from 0 to 100 within each age group using the 2.5^{th} and 97.5^{th} percentiles estimated across all locations and years.

A weighted average of scores was used to estimate a single index value for every location and year from a variety of indicators and age groups. Weights for these indicators varied across contexts based on the intervention's importance in contributing to health gains. The rationale for weighting indicators is that the meaning of health-service coverage will vary by location due to variations in local disease burdens. Health gain weights were calculated using estimates of what the disease burden would have been if there had been full health service coverage and what it would have been if there had been no health-service coverage. Disease burdens were measured in disability-adjusted life-years (DALYs).

DALY burdens for the two counterfactual scenarios of zero and full intervention coverage were calculated using estimates of intervention coverage, observed DALYs associated with a disease or condition, and external assessments of intervention effectiveness. Interventions were assigned to ordinal categories with associated effectiveness percentages based on the findings of published literature reviews in order to facilitate these calculations. The difference in DALYs between the zero and full service coverage scenarios constituted the potential health gain of the intervention for a given location, year, and age group. Dividing a potential health gain by the sum of all

potential health gains for a given location and year resulted in a fraction representing the health gain weight for that combination of indicator, age group, location, and year. To prevent any specific indicator from dominating the index, health gain weights were capped at the 97.5th and 2.5th percentile value of all weights. Uncertainty was propagated throughout the UHC index estimation using 250 draws of each measure. Contributions to uncertainty in final index values included uncertainty in modelled estimates of coverage and mortality-based proxies as well as uncertainty in estimated health gain weights. The mean and variance of the draws of the overall UHC effective coverage index for all 204 locations from 1990 to 2023 were then used in this study's SFM threshold analysis.

For this study's estimation of minimum health worker density thresholds, we aimed to generate cadre-specific targets rather than one aggregate value. To this end, we used the four health worker cadre groups identified in the Sustainable Development Goals (SDGs) indicator 3.c.1: doctors; nurses and midwives; dentists and dental assistants (dentistry personnel); and pharmacists and pharmaceutical assistants (pharmaceutical personnel). Since some of the modelled cadres in this study are more detailed than these four groups, there are actually six cadre-specific models that contribute to estimates for the groups: one for doctors, one for nurses and midwives, two for dentistry personnel, and two for pharmaceutical personnel.

While aggregating estimates into these groups precludes the identification of distinct thresholds for sub-categories of each type of health worker, it also controls for potential substitution effects between highly related occupations and accounts for variation in the way that different locations and sources may have operationalised ISCO guidelines for very similar positions. Beyond these aggregations, this analysis does not account for substitution effects between cadres. This means that if a location driving the frontier in doctors, for instance, can only maintain its level of UHC by compensating with very high levels of nurses and midwives, then the doctor threshold estimated from such a frontier would not account for that, and thus might be too low for countries with fewer nurses and midwives. The substitution phenomenon is particularly relevant in the case of task-shifting cadres, such as nurse practitioners, where there is notable overlap in roles between cadres. Because this study was unable to estimate task-shifting cadres in isolation, we are unable to comment on the extent to which the proposed thresholds are predicated on the presence of task-shifting in a health system.

Other cadres, though perhaps less similar in their titles than those that were aggregated, likely have the potential for substitution effects with the primary four as well. However, expanding the threshold analysis to include such cadres could have led to substantial misinterpretation of our results. For example, it might be possible for some high-income countries that invest a lot in health system quality and accessibility to achieve high levels of UHC with very few community health workers. If so, the global minimum threshold for community health workers would be very small. Because the thresholds represent minimum necessary levels, this would not be an inaccurate finding. But if we briefly presented such a low threshold without having space to properly discuss its implications, readers might misinterpret this as a statement that community

health workers are not valuable or do not serve a critical function in many health systems. That would be an erroneous implication.

Space constraints in the main text also militated against a broader analysis that included more cadres. We could not do justice within such a short article to the range of health workers, their distribution globally, and their corresponding thresholds. Consequently, we have chosen to focus on a more parsimonious set of high-priority cadres highlighted in the SDGs, but do not mean to imply that these are the only HRH cadres with meaningful contributions to national health-care performance.

4.3 Stochastic frontier meta-analysis (SFM)

In order to establish global evidence-based minimum thresholds for health worker densities, we developed a meta-analytic extension for stochastic frontier analysis (SFA). The classic SFA builds a non-deterministic production frontier expressing the maximum output value obtainable from a given input value, based on all observed inputs and outputs provided in a dataset. In this study, datapoints for the stochastic frontier come from modelled results, and therefore have associated estimates of variance (uncertainty). A meta-analytic extension is required to properly account for these variances. We call the modified approach stochastic frontier meta-analysis (SFM). Just as with standard random-effects SFA, the SFM tool models inefficiencies as random effects that must be negative, using a half-normal model. Unlike SFA, SFM accounts for the variances of individual datapoints. In addition, SFM is equipped with three features that make the analysis of the data robust and straightforward. The first is a robust extension that simultaneously detects and removes outliers from the analysis, based on generalised trimming methodology.[22–24] The second is a spline model for the frontier, which does not assume any parametric function and allows the SFM to be applied directly to the data without pre-analysis or post-analysis (no transformations are needed). The third feature is a Bayesian module that permits placing shape constraints and priors on the frontier. Technical details of the SFM approach are described in detail in the attached technical supplement.

We applied the SFM tool using an established universal health coverage (UHC) effective coverage index (based on GBD 2023 results) and its associated variance as the output, and estimates of health worker cadre groups as the input. The four cadre groups included in the frontier analyses were those specified in SDG indicator 3.c.1: doctors, nursing and midwifery personnel, dentists, and pharmacists.[10] We therefore generated four distinct production frontiers, each using all estimates for the cadre group being analysed, for all locations and years. Additionally, we ran 250 frontiers, using the knot placement and outliers indicated by the mean frontier, with draws from each health worker cadre and the UHC effective coverage index to construct 95% uncertainty intervals (UIs) using the 2·5th and 97·5th percentiles of predicted frontiers. Using all locations and years increased the representativeness and stability of our frontier, as it was fit to a much larger dataset reflecting locations from all parts of the world, and also expanded the range of inputs and outputs for which the frontier could provide estimates. We suspected a priori that frontiers would monotonically increase (the addition of more health workers would not decrease maximum potential UHC achievement) and would exhibit

diminishing returns (the contribution of each additional health worker would be more significant where workforce levels are lower). Having observed this suspected relationship in preliminary analyses, we incorporated both as constraints in the frontier fits. SFM analyses were thus run using monotonically increasing concave splines with seven knots. These constraints ensured that where data were sparse (for example, at very high levels of HRH), stochastic variations and inefficiencies did not drive the flexible frontier toward unrealistic trends (insinuating, for example, that at certain health workforce densities, additional workers would be detrimental to maximum potential UHC attainment). The right-most segment of the spline was also constrained to be flat in order to preclude extrapolation beyond observed levels of UHC attainment. We used 7.5% trimming, so that the most extreme 7.5% of observations were detected as outliers and excluded as the frontier was constructed. This reduced any subjectivity inherent to ad-hoc outlier identification.

For any cadre-specific health worker density, the corresponding production frontier value provides an estimate of the maximum UHC attainment that is theoretically possible if countries or territories are optimising the efficiency with which they translate human resources into UHC. By comparing actual UHC performance to the estimated maximum potential UHC attainment, our frontier analysis identifies the productive efficiencies with which countries and regions are attaining UHC with their existing health worker densities. To be clear, productive efficiency estimates highlight country capacities related to health system components and contexts *beyond* health workforce densities, such as workforce distribution, the quality of workforce training, or non-workforce factors related to health-care access and quality, such as health-care technology, population distributions, and transportation infrastructure. Accordingly, countries and territories with the same health workforce densities can exhibit a range of productive efficiency scores.

Across the spectrum of health worker densities, countries and territories may be identified as highly efficient, and thus driving the UHC frontier at their level of HRH, or less efficient given what would be expected from the size of their health workforce. For example, a country with a relatively small nurse workforce density may have a UHC performance close to the theoretical maximum, and may therefore have a higher efficiency score than a country that has a larger nurse workforce density and a higher level of UHC yet does not come as close to its theoretical maximum. Countries with low frontier efficiencies may look to more efficient performers with similar health workforce densities for insight into potential improvements in other health system factors that would facilitate better UHC attainment. Therefore, reaching a minimum threshold for health workforce density is by no means sufficient for achieving a target level of UHC effective coverage, and many countries with or without health worker shortages also have low productive efficiencies, indicating a need for investments beyond just expanding the health workforce size. Since reaching 100% efficiency in the translation of human resources into UHC effective coverage performance is not possible for every location, many countries and territories may require workforce densities beyond the minimum thresholds proposed in this study. However, it is important to note that increasing workforce sizes cannot always compensate for issues

underlying low productive efficiency, and certainly may not be a cost-effective or appropriate means of advancing UHC when HRH levels are already sufficient.

In this analysis, we use the fitted production frontiers to identify minimum cadre-specific health worker densities needed to achieve specific UHC attainment targets, assuming countries and territories are operating at maximum productive efficiency. Minimum density thresholds for each health worker cadre can be determined as the x-values of each corresponding frontier as it crosses a given target value of UHC performance. We determined minimum density thresholds for each health worker cadre to achieve performance targets of 80 and 90 out of 100 on the UHC effective coverage index. Since UHC is a measure of essential health services for all populations, every country should strive for the highest attainable score on the corresponding index. However, because SFM is fit to historical data, these production frontiers cannot be used to extrapolate HRH requirements for UHC effective coverage beyond what was achieved between 1990 and 2023. This is one reason why the fitted frontiers never reach the x-axis, because no locations in this analysis achieved UHC effective coverage scores so close to zero. During this study period, the highest levels of estimated UHC effective coverage (at or above 90 out of 100) almost entirely occurred in high-income countries and territories. Given limitations in the number and diversity of locations reaching this UHC effective coverage score, the frontier results for such a target are less likely to be accurate or representative at a global scale. For that reason, we chose to focus on a UHC effective coverage of 80 out of 100 for this study, since it was the highest approximate score that had been reached by a wide range of locations. In future analyses as more countries achieve higher levels of UHC, it may be possible to establish stable and representative thresholds for even higher targets of effective coverage.

It is important to note that choosing a high UHC attainment target should not conflict with the interpretation of thresholds as minimum health workforce densities. Our research aimed to determine the minimum human resources required to achieve a high level of health service coverage and quality. In other words, the findings of this frontier analysis are predicated on a high level of UHC effective coverage (an appropriate health system goal) but provide estimates of the minimum HRH densities needed to meet that target (minimising costs and inefficiency).

It is also important to note that the thresholds proposed in this analysis do not necessarily represent an ideal HRH skill mix for any given location. Health systems around the world differ dramatically in their distribution of HRH cadres for a variety of reasons, and this study does not intend to prescribe exact levels or ratios in the makeup of a health workforce. Instead, this study aims to use existing estimates of HRH to identify a globally applicable common denominator of minimum workforce requirements necessary to meet a specified health system performance goal. This study would suggest, for instance, that health systems more heavily reliant on nurses and midwives should still require the minimum threshold densities for doctors, dentistry personnel, and pharmaceutical personnel in order to reach a UHC level of 80 out of 100. However, in maintaining its own preferred skill mix, that health system would likely find itself surpassing the threshold for nurses and midwives in the process. This example is presented to emphasise that these thresholds only reflect minimum HRH densities, not exact targets. They do not imply that

all health systems must employ the same strategies or HRH skill mix in order to achieve high levels of UHC. Future work may explore frontier analyses on subsets of locations that exhibit similar skill mixes to better characterise additional contextual workforce requirements beyond this study's minimum levels. Such tailored thresholds might better characterise what the health workforce would look like in countries achieving high levels of UHC while maintaining a particular skill mix, which could be useful for health system planning purposes.

Section 5. Online tools and glossary of terms

Online tools GBD 2023 data sources and additional results are presented in a series of tools and dynamic visualisations, available at https://ghdx.healthdata.org/gbd-2023.

Appendix 1 Table 9. List of abbreviations

Abbreviation	Description
ART	Antiretroviral therapy
ASHA	Accredited Social Health Activist (India's Community Health Program run by the Ministry of Health and Family Welfare)
DALY	Disability-adjusted life-year
DTP3	Diphtheria, tetanus, and pertussis vaccine
Eurostat	Statistical Office of the European Union
GATHER	Guidelines for Accurate and Transparent Health Estimates Reporting
GBD	Global Burden of Diseases, Injuries, and Risk Factors Study
GGE	General government expenditure
GHDx	Global Health Data Exchange
GPR	Gaussian process regression
HRH	Human resources for health
ILO	International Labour Organization
ISCO	International Standard Classification of Occupations
MDGs	Millennium Development Goals
MIRs	Mortality-to-incidence ratios
MR-BRT	Meta-regression—Bayesian, regularised, trimmed
MCV1	Measles-containing vaccine
NCDs	Non-communicable diseases
NCO	National Classification of Occupations (India's country-specific occupation coding system)
NHWA	National Health Workforce Accounts
OECD	Organisation for Economic Co-operation and Development
PAF	Population attributable fraction
SDG	Sustainable Development Goals
SDI	Socio-demographic Index
SFA	Stochastic frontier analysis
SFM	Stochastic frontier meta-analysis
SINCO	Sistema Nacional de Clasificación de Ocupaciones (Mexico's country-specific occupation coding system)
SOC	Standard Occupational Classification (USA's country-specific occupation system)
ST-GPR	Spatiotemporal Gaussian process regression
THE	Total health expenditure
UHC	Universal health coverage
UI	Uncertainty interval
WHO	World Health Organization

Section 6. References

1 Stevens GA, Alkema L, Black RE, *et al.* Guidelines for Accurate and Transparent Health Estimates Reporting: the GATHER statement. *Lancet* 2016; **388**: e19–23.

2 Haakenstad A, Irvine CMS, Knight M, *et al.* Measuring the availability of human resources for health and its relationship to universal health coverage for 204 countries and territories from 1990 to 2019: a systematic analysis for the Global Burden of Disease Study 2019. *The Lancet* 2022; **399**: 2129–54.

3 Lozano R, Fullman N, Mumford JE, *et al.* Measuring universal health coverage based on an index of effective coverage of health services in 204 countries and territories, 1990–2019: a systematic analysis for the Global Burden of Disease Study 2019. *Lancet* 2020; **396**: 1250–84.

4 Hay SI, Ong LK, Santomauro DF, et al. Burden of 375 diseases and injuries, risk-attributable burden of 88 risk factors, and healthy life expectancy in 204 countries and territories, including 660 subnational locations, 1990-2023: a systematic analysis for the Global Burden of Disease Study 2023. *Lancet* 2025; **406**. DOI:10.1016/S0140-6736(25)01637-X.

5 Naghavi M, Kyu H, Bhoomadevi A, *et al.* Global burden of 292 causes of death in 204 countries and territories and 660 subnational locations, 1990-2023: a systematic analysis for the Global Burden of Disease Study 2023. *Lancet* 2025; **406**. DOI:10.1016/S0140-6736(25)01917-8.

6 Schumacher A, Zheng P, Barber RM, et al. Global age-sex-specific all-cause mortality and life expectancy estimates for 204 countries and territories and 660 subnational locations, 1950-2023: a demographic analysis for the Global Burden of Disease Study 2023. *Lancet* 2025; **406**. DOI:10.1016/S0140-6736(25)01330-3.

7 International Labour Organization. Resolution concerning statistics of work, employment and labour underutilization (Including amendments). Geneva: International Labour Organization, 2023 https://www.ilo.org/resource/resolution-concerning-statistics-work-employment-and-labour (accessed Oct 6, 2025).

8 World Health Organization. Classifying health workers: mapping occupations to the international standard classification. Geneva: World Health Organization, 2019.

9 World Health Organization, World Bank, United States Agency for International Development. Handbook on monitoring and evaluation of human resources for health. Geneva, 2009 https://wkc.who.int/resources/publications/i/item/handbook-on-monitoring-and-evaluation-of-human-resources-for-health (accessed Oct 3, 2025).

10 World Health Organization. SDG indicator metadata: health worker density and distribution (3.c.1). New York: United Nations Statistics Division, 2023 https://unstats.un.org/sdgs/metadata/files/metadata-03-0c-01.pdf.

11 World Health Organization. National health workforce accounts: a handbook, second edition. Geneva: World Health Organization, 2023 https://www.who.int/publications/i/item/9789240081291 (accessed Oct 17, 2025).

12 European Union. Health care resources database. https://ec.europa.eu/eurostat/web/health/database (accessed Aug 14, 2025).

13 World Health Organization. National Health Workforce Accounts: variable definitions (metadata). World Health Organization. 2025; published online Dec 1. https://apps.who.int/nhwaportal/files/notes.pdf (accessed June 23, 2026).

14 ASHA India. Annual Reports. https://asha-india.org/resources/annual-reports/ (accessed Oct 17, 2025).

15 National Bureau of Statistics of China. China Statistical Yearbook. https://www.stats.gov.cn/english/Statisticaldata/yearbook/ (accessed Oct 17, 2025).

16 International Labour Organization. International Standard Classification of Occupations 2008 (ISCO-08): structure, group definitions and correspondence tables. Geneva: International Labour Organization, 2012 https://www.ilo.org/publications/international-standard-classification-occupations-2008-isco-08-structure (accessed Oct 1, 2025).

17 World Health Organization. The impact of COVID-19 on health and care workers: a closer look at deaths. Geneva: World Health Organization, 2021 https://iris.who.int/handle/10665/345300 (accessed Aug 28, 2025).

18 Shen K, Eddelbuettel JCP, Eisenberg MD. Job flows into and out of health care before and after the COVID-19 pandemic. *JAMA Health Forum* 2024; **5**: e234964.

19 World Health Organization. Working together for health. Geneva: World Health Organization, 2006 https://www.who.int/publications/i/item/9241563176 (accessed Oct 15, 2025).

20 World Health Organization. Health workforce requirements for universal health coverage and the Sustainable Development Goals. Geneva: World Health Organization, 2016 https://www.who.int/publications/i/item/9789241511407 (accessed Oct 15, 2025).

21 World Health Organization. WHO health workforce support and safeguards list 2023. Geneva: World Health Organization, 2023 https://www.who.int/publications/i/item/9789240069787 (accessed Oct 17, 2025).

22 Aravkin A, Davis D. Trimmed statistical estimation via variance reduction. *Math Oper Res* 2020; **45**: 292–322.

23 Yang E, Lozano AC, Aravkin A. A general family of trimmed estimators for robust high-dimensional data analysis. *Electron J Stat* 2018; **12**: 3519–53.

24 Zheng P, Worku N, Bannick M, *et al.* Robust nonparametric stochastic frontier analysis. 2024; published online April 11. DOI:10.2139/ssrn.4791808.
