# Supplementary material for: Measuring the composition and availability of human resources for health by sex in 204 countries and territories, 1990–2023, and workforce gaps for universal health coverage: a systematic analysis for the Global Burden of Disease Study 2023
Source: Lancet Public Health. 2026 Aug 18;11(9):e604–29. doi: 10.1016/S2468-2667(26)00145-3 (PMC13501144; doi:10.1016/S2468-2667(26)00145-3)

THE LANCET
Public Health

Supplementary appendix 2

This appendix formed part of the original submission and has been peer reviewed. We post it as supplied by the authors.

Supplement to: GBD 2023 Human Resources for Health Collaborators. Measuring the composition and availability of human resources for health by sex in 204 countries and territories, 1990–2023, and workforce gaps for universal health coverage: a systematic analysis for the Global Burden of Disease Study 2023. *Lancet Public Health* 2026;
11: 604–29.

Appendix 2: supplementary results appendix to “Measuring the composition and availability of human resources for health by sex in 204 countries, 1990–2023, and workforce gaps for universal health coverage: a systematic analysis for the Global Burden of Disease Study 2023”

This supplement provides additional results for “Measuring the composition and availability of human resources for health by sex in 204 countries, 1990–2023, and workforce gaps for universal health coverage: a systematic analysis for the Global Burden of Disease Study 2023.”

Figures

Appendix 2 Figure S1: Densities of midwifery personnel by country, 2023 3

Appendix 2 Figure S2: Densities of dentists by country, 2023 3

Appendix 2 Figure S3: Densities of pharmacists by country, 2023 4

Appendix 2 Figure S4: Densities of community health workers by country, 2023 4

Appendix 2 Figure S5: Densities of all health workers by country, 2023 5

Appendix 2 Table S1: Counts of health workers in millions (95% UI) by cadre globally and by GBD super-region, 2023 5

Appendix 2 Figure S6: Female share of the total health workforce by country, 2023 6

Appendix 2 Figure S7: Composition of HRH by country, 2023 6

Appendix 2 Table S2: Additional count of workers in millions (95% UI) needed to achieve minimum density of workers for four cadres at UHC effective coverage of 80 out of 100 on the UHC effective coverage index by location, 2023 17

Appendix 2 Table S3: Additional count of workers in millions (95% UI) needed to achieve minimum density of workers for four cadres at UHC effective coverage of 90 out of 100 on the UHC effective coverage index by GBD super-region, 2023 24

Appendix 2 Figure S8: Trends in global sex-specific health worker densities by cadre, 1990-2023 26

Appendix 2 Figure S9: Nurse−to−doctor ratio by country, 2023 27

Appendix 2 Figure S10a: Absolute difference in densities of all health workers by country, 1990−2023 27

Appendix 2 Figure S10b: Absolute difference in densities of doctors by country, 1990−2023 28

Appendix 2 Figure S10c: Absolute difference in densities of nursing personnel by country, 1990−2023 28

Appendix 2 Figure S10d: Absolute difference in densities of midwifery personnel by country, 1990−2023 29

Appendix 2 Figure S10e: Absolute difference in densities of dentistry personnel by country, 1990−2023 29

Appendix 2 Figure S10f: Absolute difference in densities of pharmaceutical personnel by country, 1990−2023 30

Appendix 2 Figure S10h: Absolute difference in densities of other health workers by country, 1990−2023 31

Appendix 2 Table S4: Density of health workers (95% UI) for all SDI quintiles, GBD super-regions, regions, and 204 countries and territories in 2023 32

Appendix 2 Table S5: Estimated female share as percent of health workers for all GBD super-regions, regions, and 204 countries and territories in 2023 127

Appendix 2 Figure S11: Sex composition of absolute and proportionate change in health worker densities between 1990 and 2023 140

Appendix 2 Figure S12: Maximum possible UHC effective coverage index achievement given densities in 2023 of doctors (A), nurses and midwives (B), dentists (C), and pharmacists (D) 141

Appendix 2 Figure S1: Densities of midwifery personnel by country, 2023

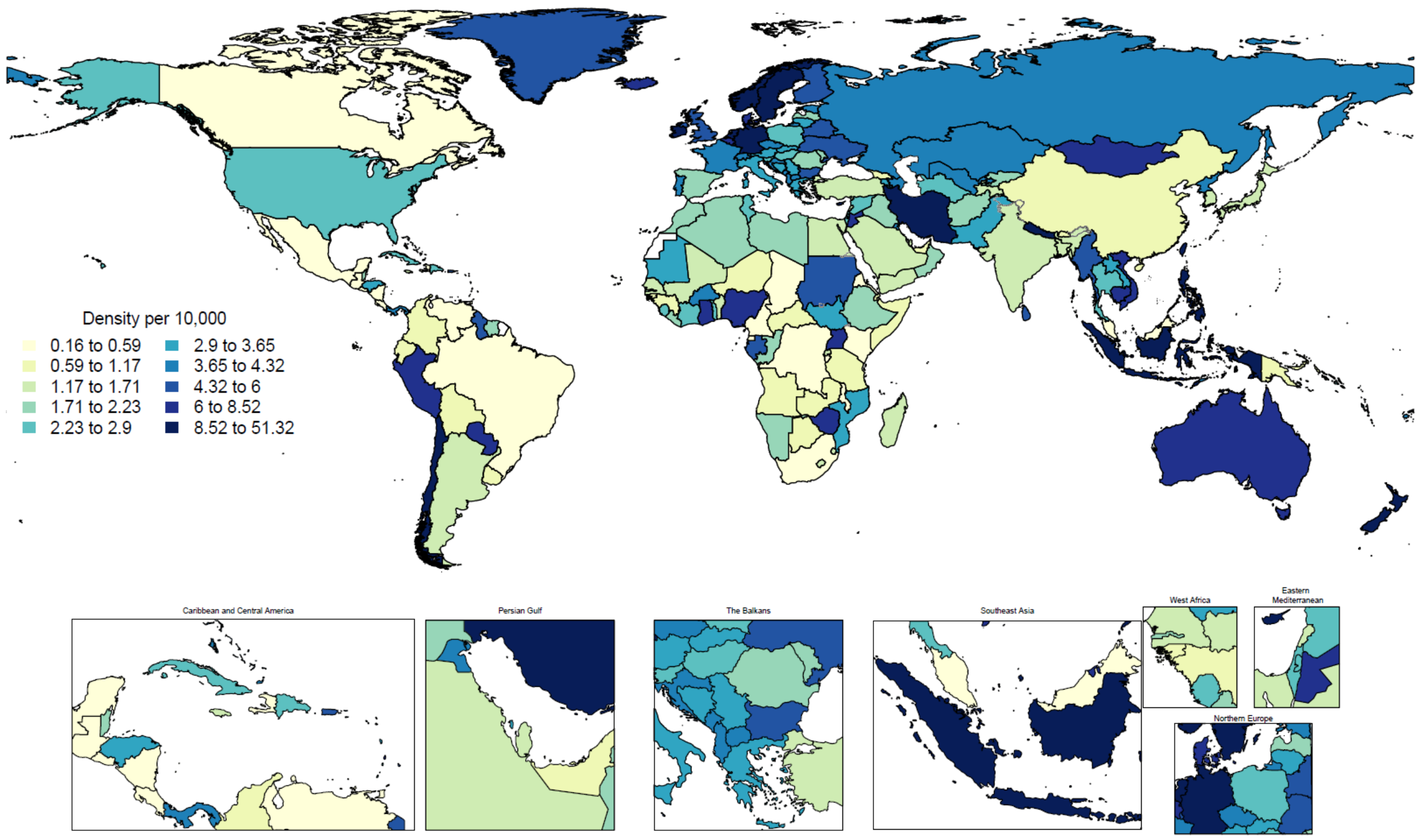

Appendix 2 Figure S2: Densities of dentists by country, 2023

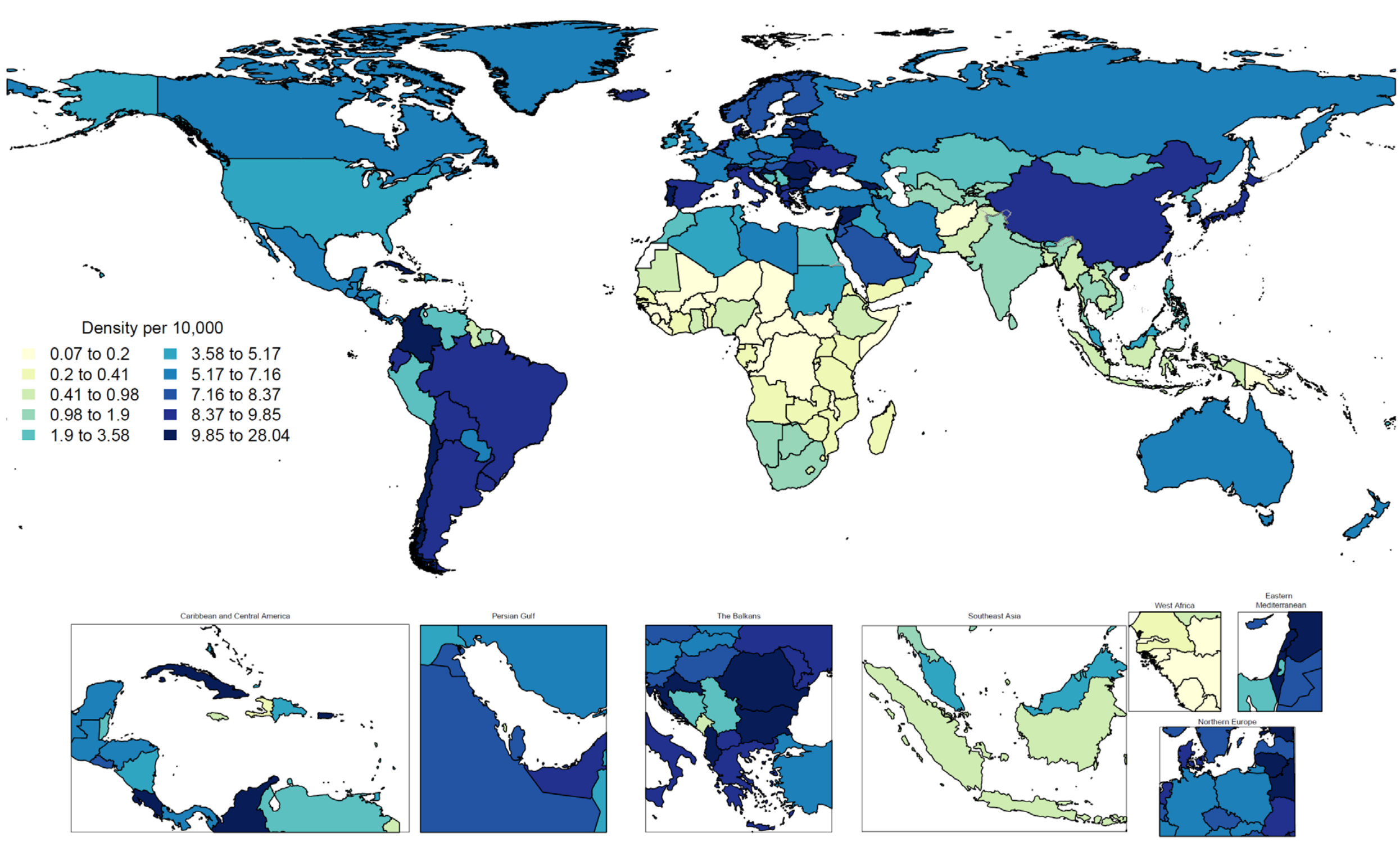

Appendix 2 Figure S3: Densities of pharmacists by country, 2023

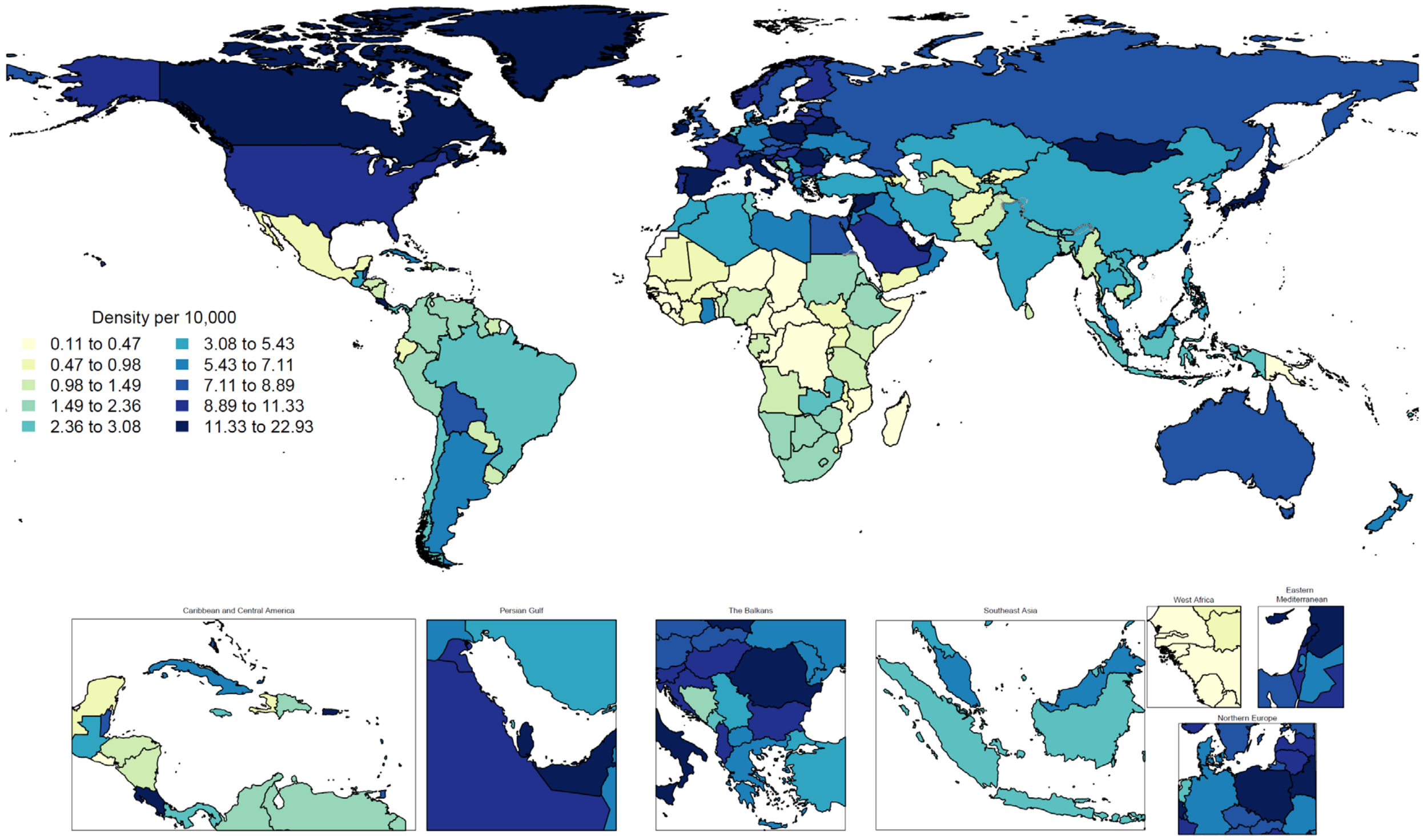

Appendix 2 Figure S4: Densities of community health workers by country, 2023

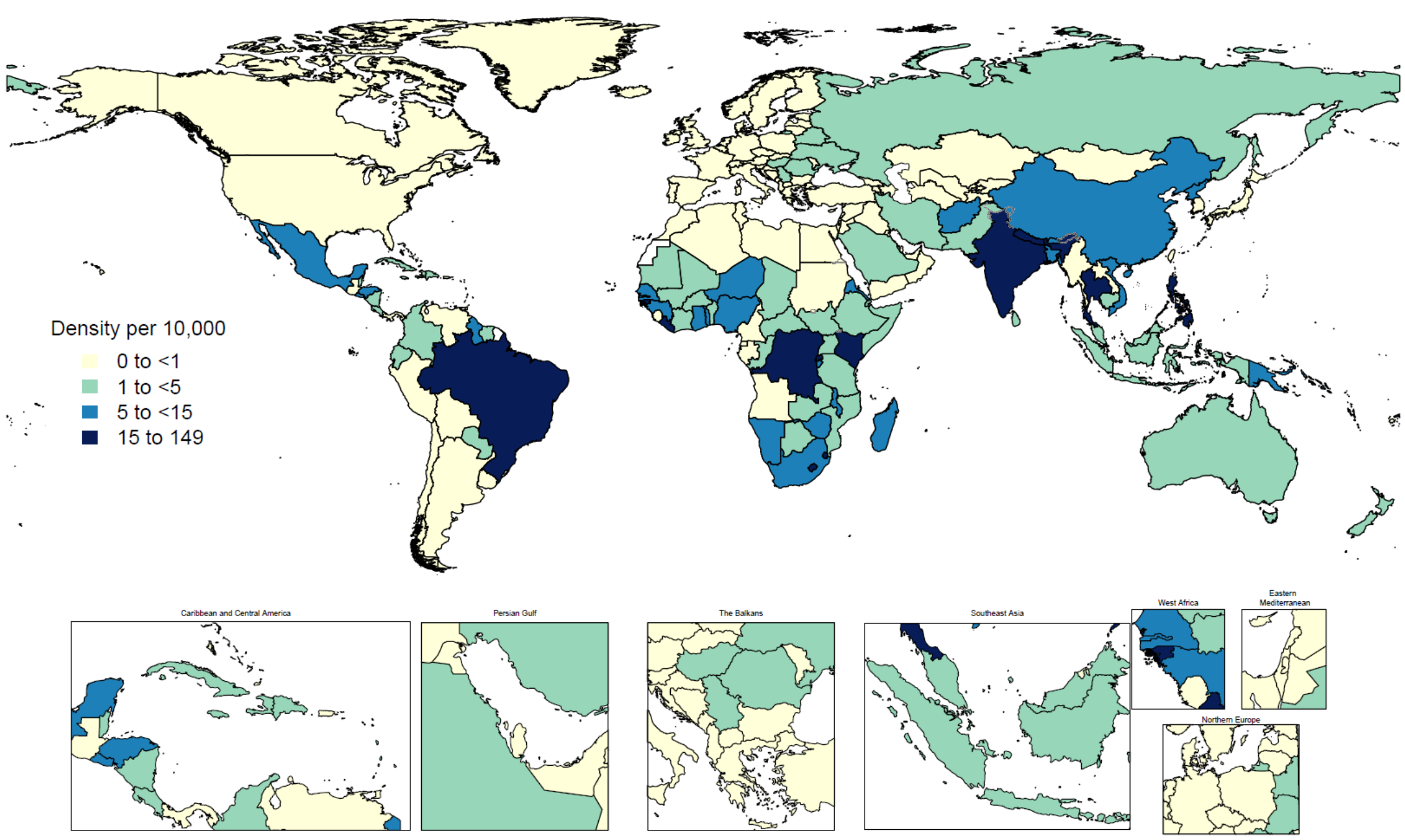

Appendix 2 Figure S5: Densities of all health workers by country, 2023

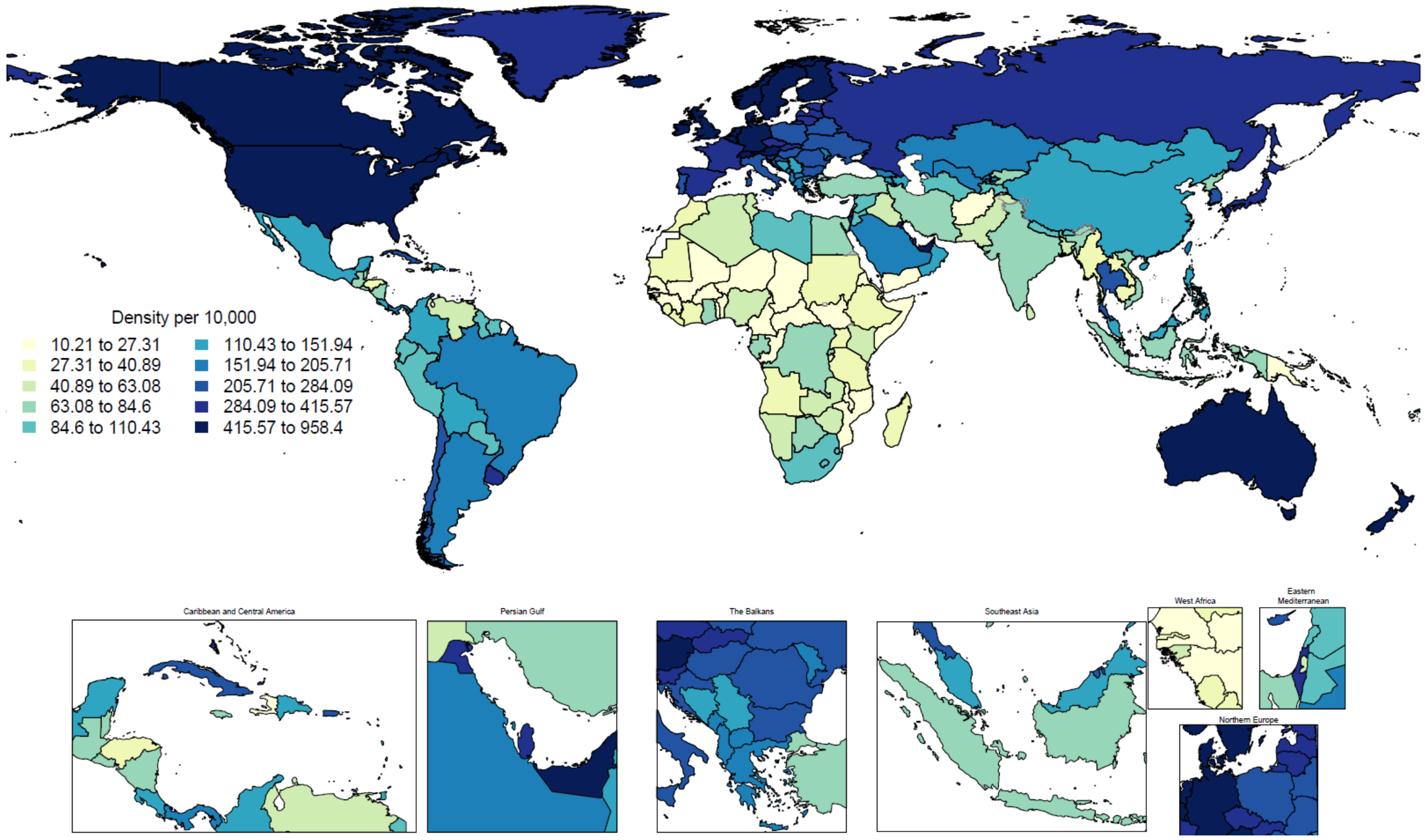

Appendix 2 Table S1: Counts of health workers in millions (95% UI) by cadre globally and by GBD super-region, 2023

	All health workers	Doctors	Nursing personnel	Pharmacists and pharmaceutical technicians and assistants	Dentists and dental assistants	Midwifery personnel	Community health workers
Global	122.1 (115.4 to 130.5)	15.1 (13.1 to 18.2)	33.2 (30.8 to 36.3)	6.8 (5.8 to 8.6)	6.1 (5.0 to 7.9)	2.4 (2.0 to 2.9)	7.6 (6.7 to 8.6)
Central Europe, Eastern Europe, and Central Asia	10.1 (9.5 to 10.8)	1.3 (1.2 to 1.5)	3.3 (3.1 to 3.7)	0.5 (0.5 to 0.7)	0.4 (0.4 to 0.5)	0.2 (0.1 to 0.2)	0.1 (0 to 0.1)
High-income	56.7 (52.7 to 61.3)	4 (3.8 to 4.4)	13.4 (12.4 to 14.8)	1.9 (1.7 to 2)	2.3 (2 to 2.8)	0.4 (0.4 to 0.6)	0 (0 to 0)
Latin America and Caribbean	7.7 (7.2 to 8.3)	1.4 (1.2 to 1.6)	2.5 (2.1 to 2.9)	0.3 (0.2 to 0.4)	0.8 (0.7 to 1)	0.1 (0 to 0.1)	0.6 (0.6 to 0.7)
North Africa and Middle East	4.9 (4.6 to 5.2)	0.9 (0.8 to 1.1)	1.4 (1.3 to 1.5)	0.6 (0.5 to 0.8)	0.3 (0.3 to 0.4)	0.2 (0.2 to 0.2)	0.1 (0.1 to 0.1)
South Asia	13 (11.5 to 14.9)	1.9 (1.3 to 3)	1.9 (1.2 to 3)	1.5 (1 to 2.4)	0.4 (0.2 to 0.7)	0.3 (0.2 to 0.6)	2.6 (2 to 3.3)
Southeast Asia, East Asia, and Oceania	26.1 (24 to 28.7)	5.3 (4 to 7)	9.3 (8.3 to 10.5)	1.6 (1.1 to 2.3)	1.8 (1 to 3.1)	0.9 (0.6 to 1.2)	3 (2.4 to 3.6)
Sub-Saharan Africa	5 (4.5 to 5.5)	0.3 (0.3 to 0.5)	1.7 (1.4 to 2.2)	0.2 (0.2 to 0.3)	0.1 (0.1 to 0.1)	0.3 (0.3 to 0.4)	1.2 (1.1 to 1.4)

Appendix 2 Figure S6: Female share of the total health workforce by country, 2023

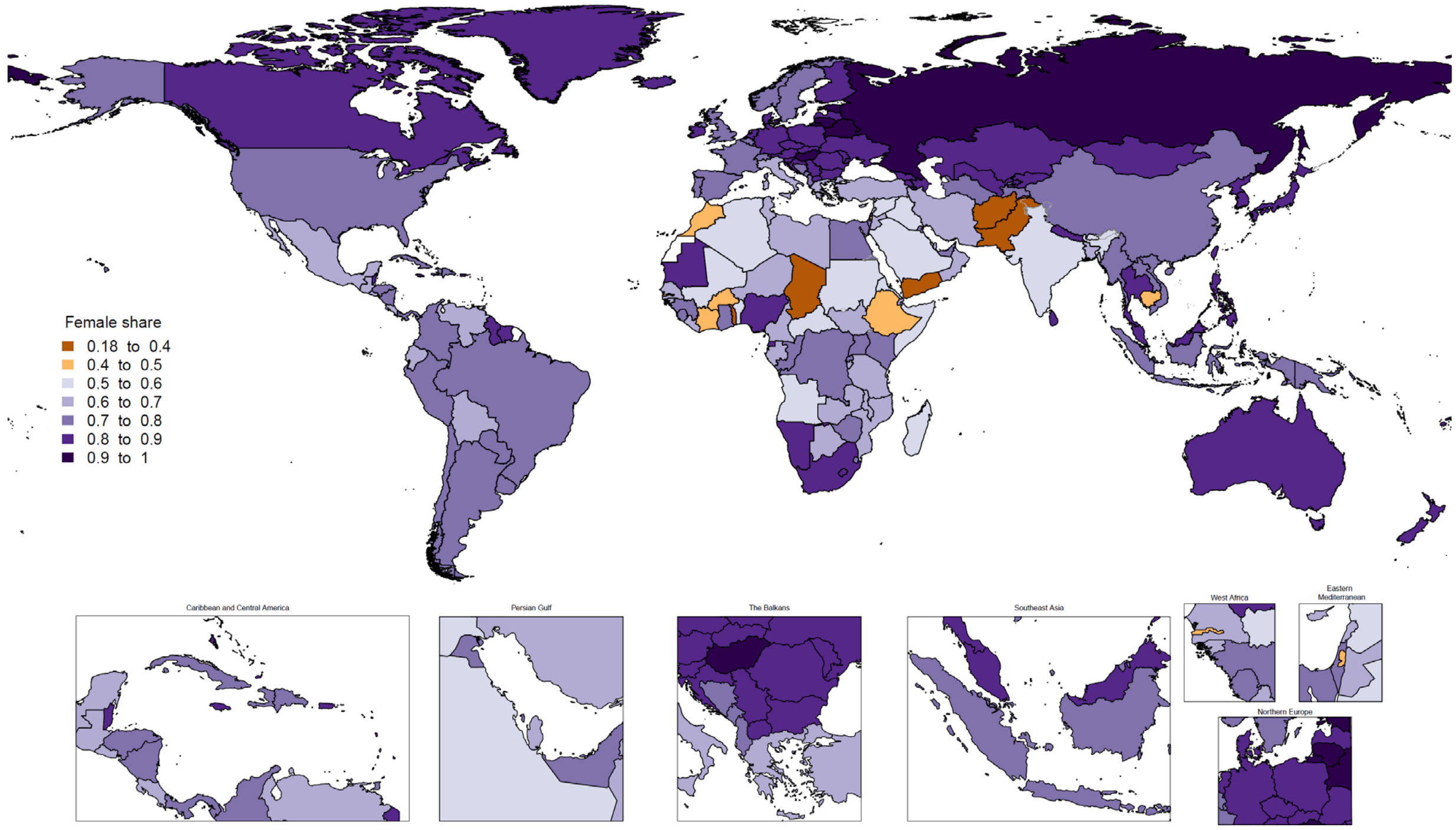

Appendix 2 Figure S7: Composition of HRH by country, 2023

Appendix 2 Figure S7a: East Asia, 2023

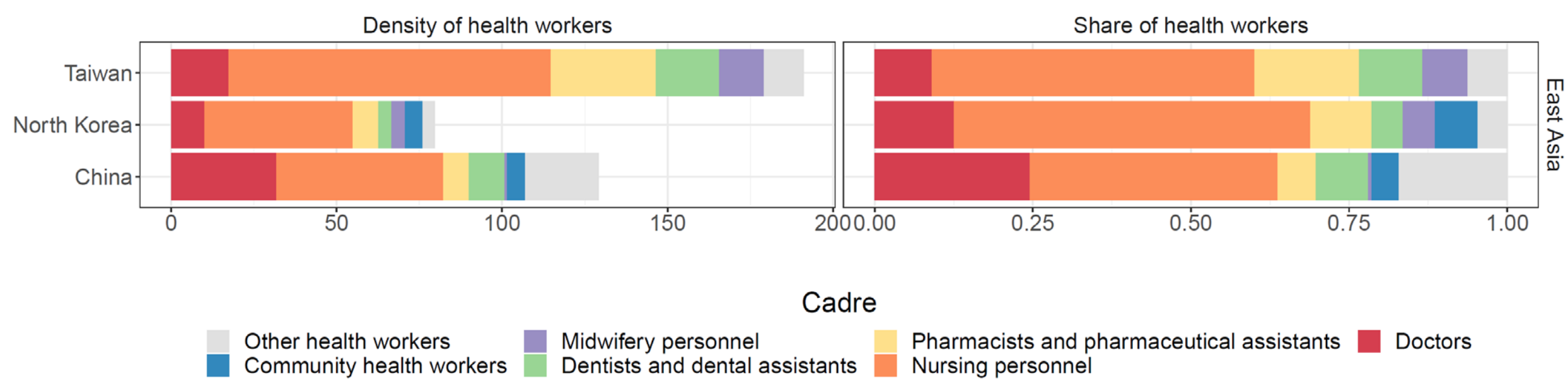

Appendix 2 Figure S7b: Southeast Asia, 2023

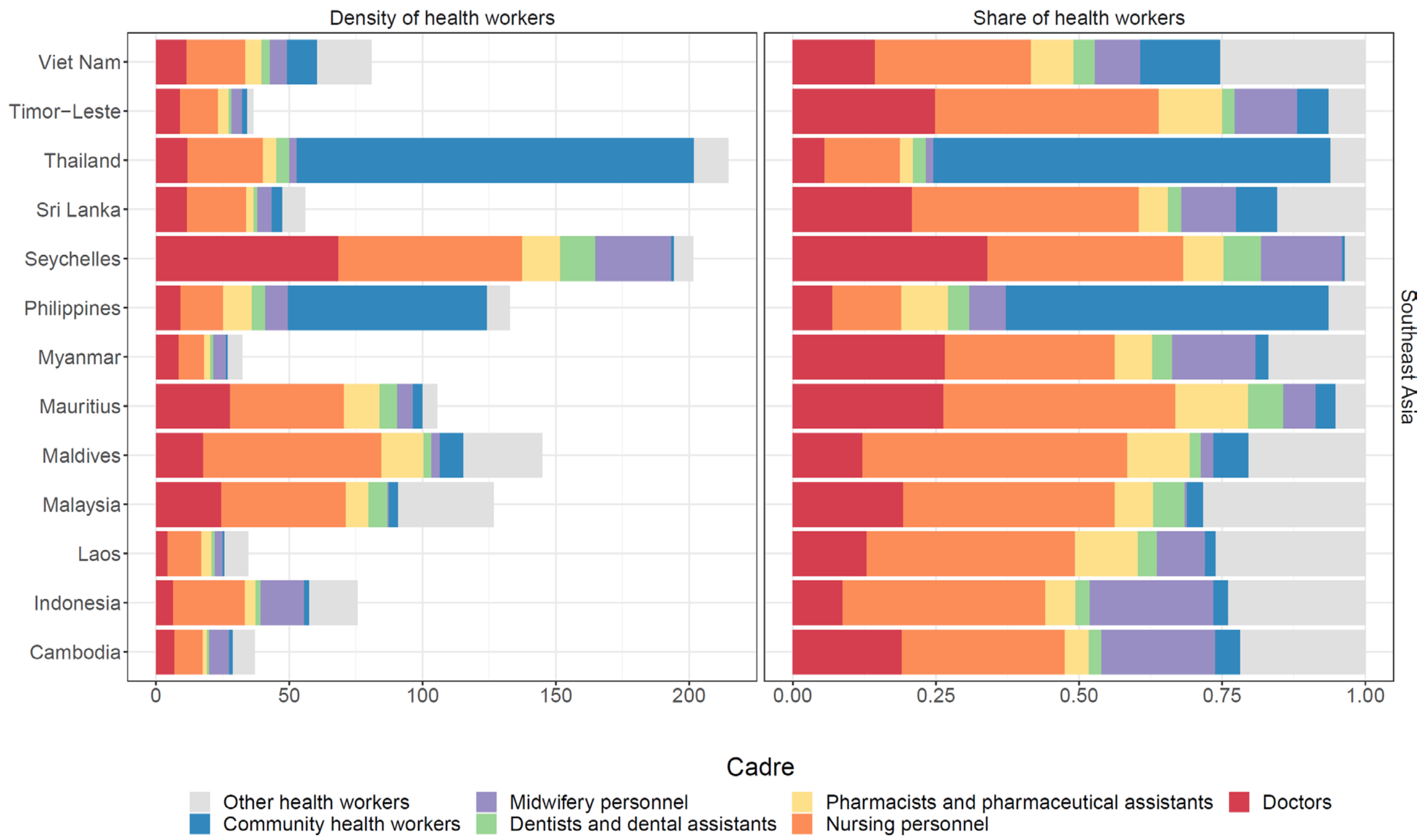

Appendix 2 Figure S7c: Oceania, 2023

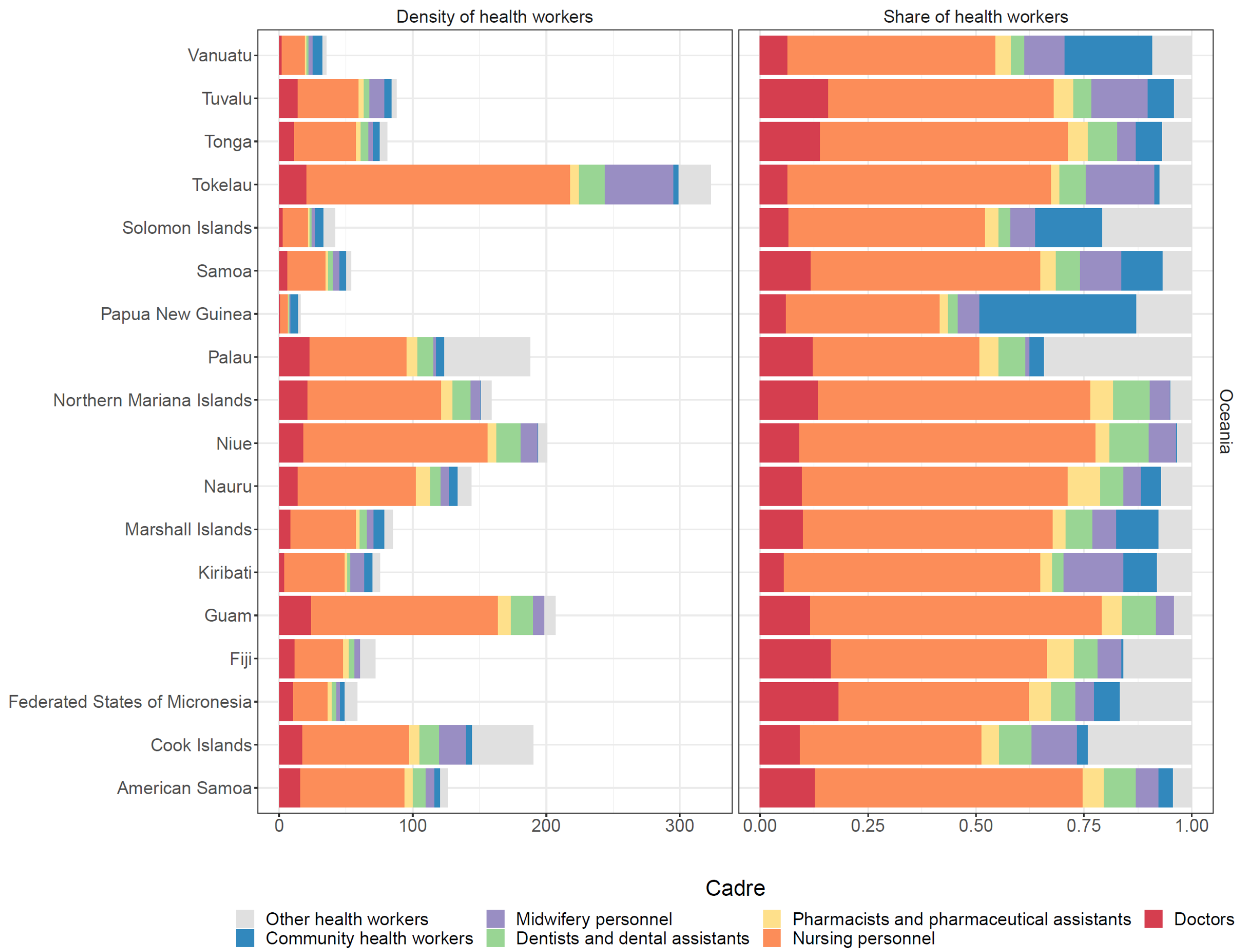

Appendix 2 Figure S7d: Central Asia, 2023

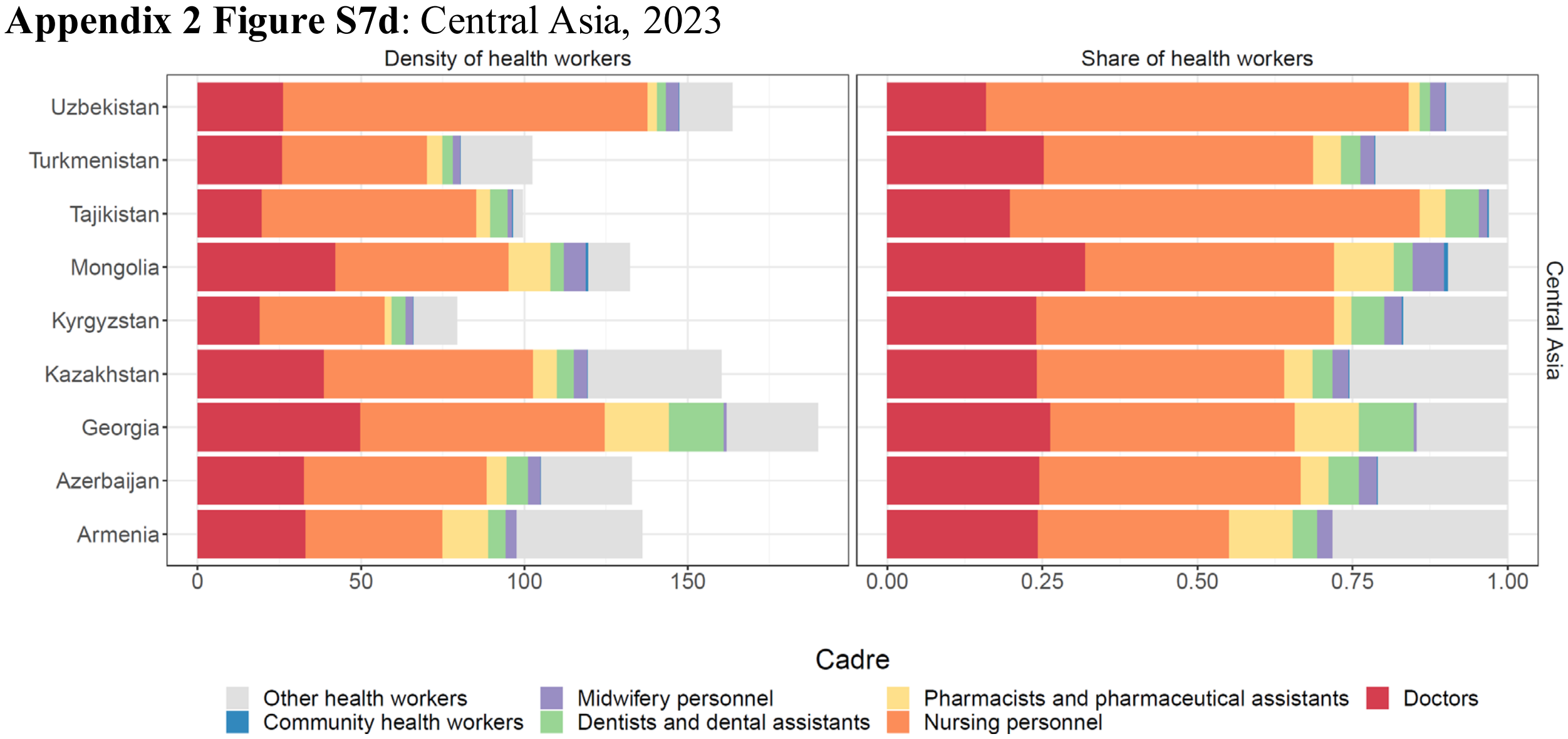

Appendix 2 Figure S7e: Central Europe, 2023

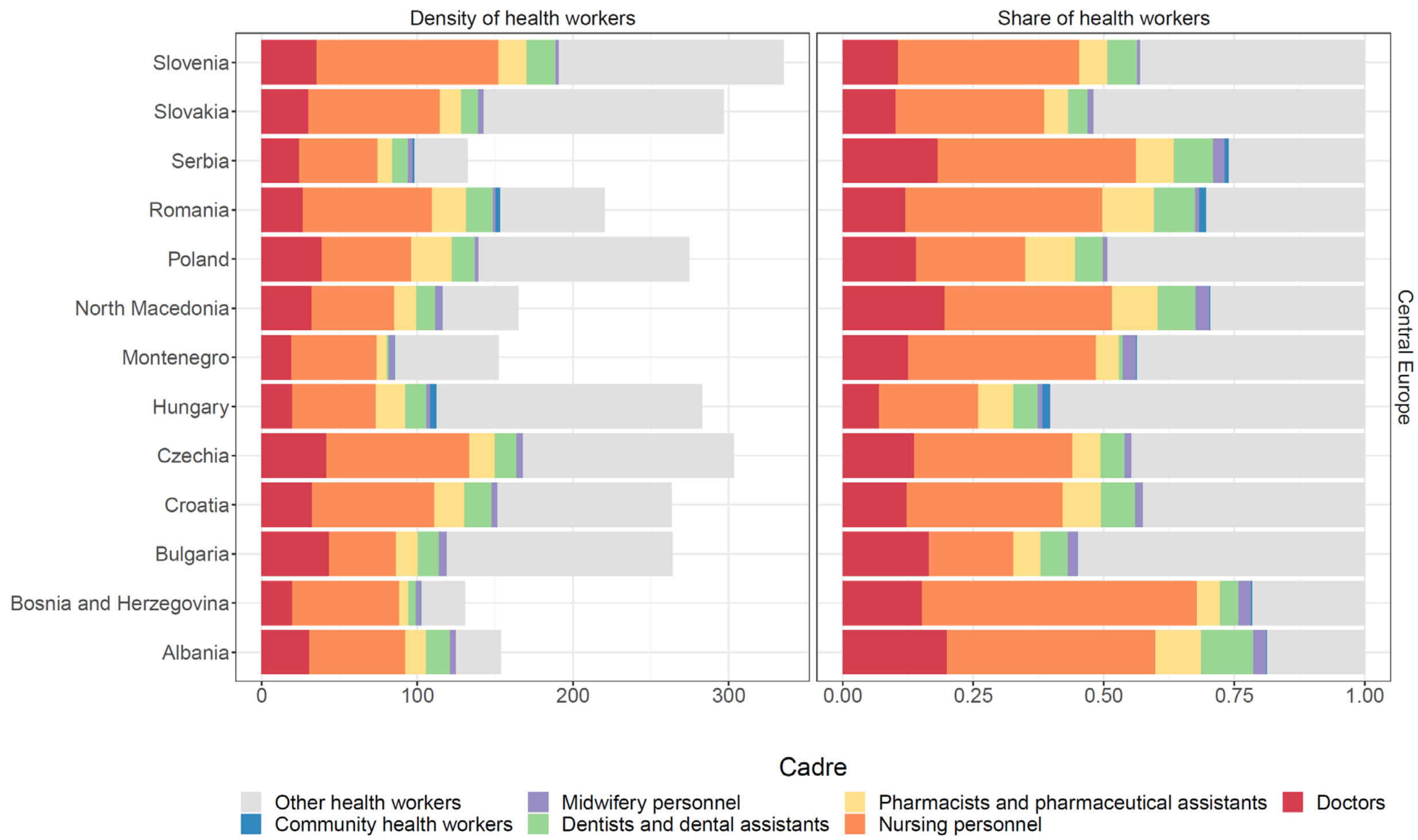

Appendix 2 Figure S7f: Eastern Europe, 2023

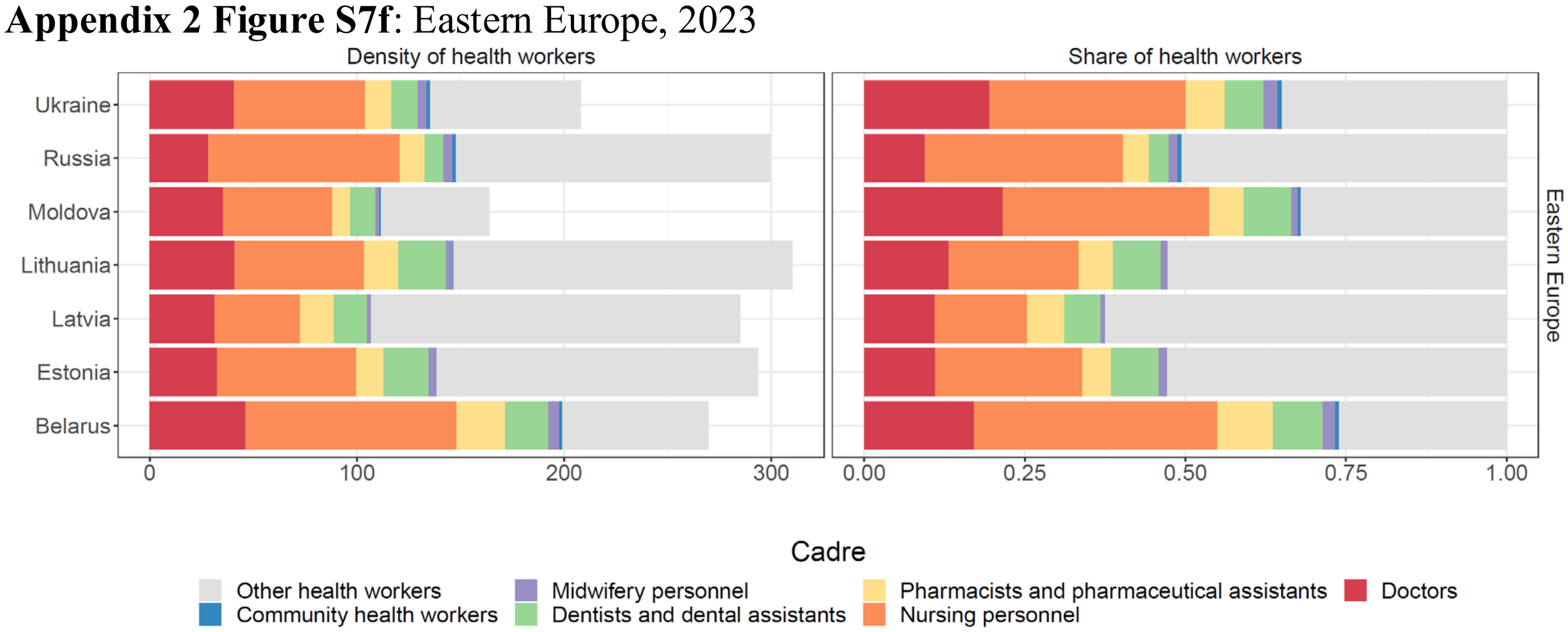

Appendix 2 Figure S7g: High-income Asia Pacific, 2023

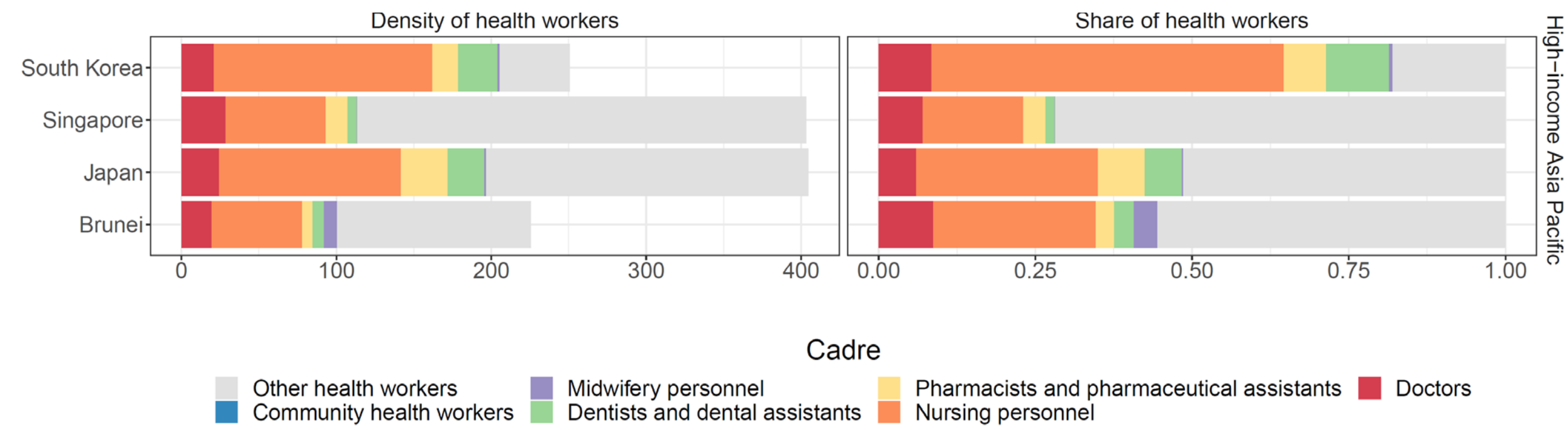

Appendix 2 Figure S7h: Australasia, 2023

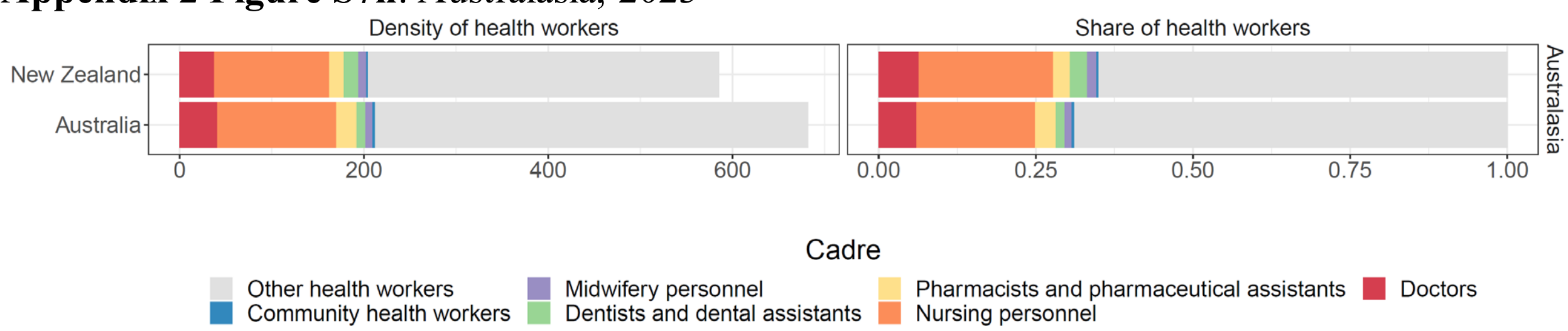

Appendix 2 Figure S7i: Western Europe, 2023

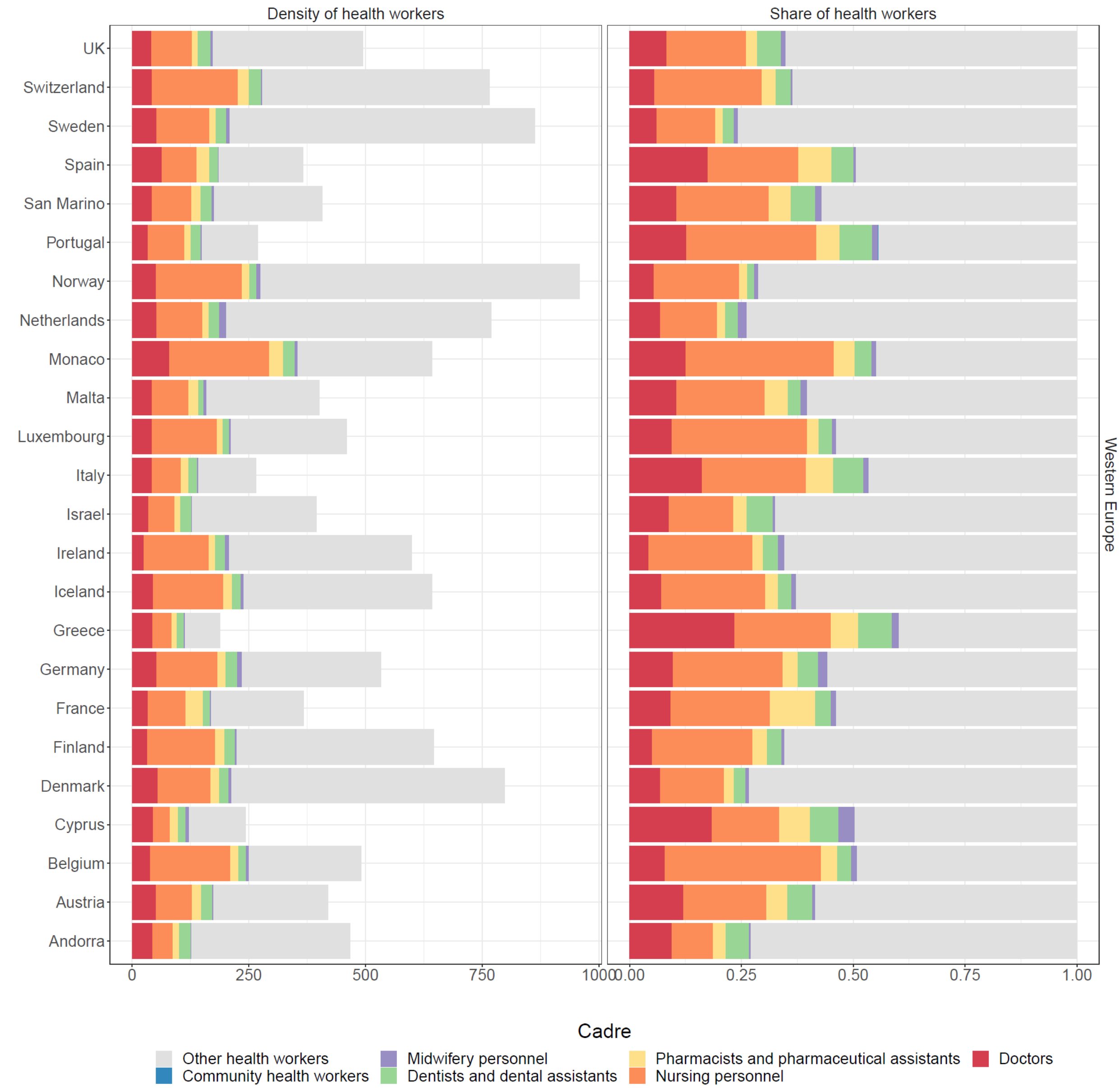

Appendix 2 Figure S7j: Southern Latin America, 2023

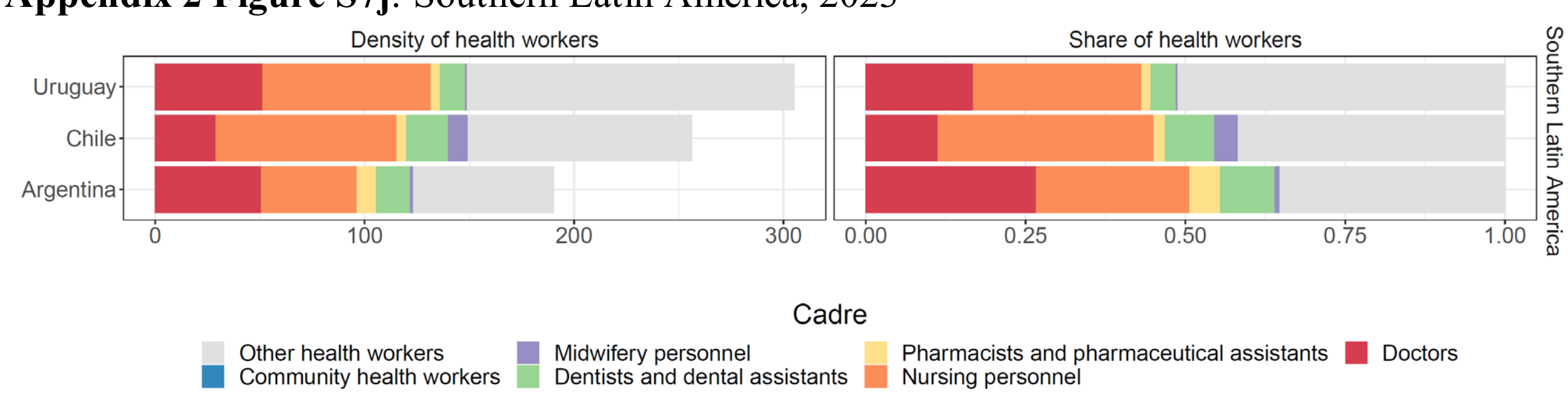

Appendix 2 Figure S7k: High−income North America, 2023

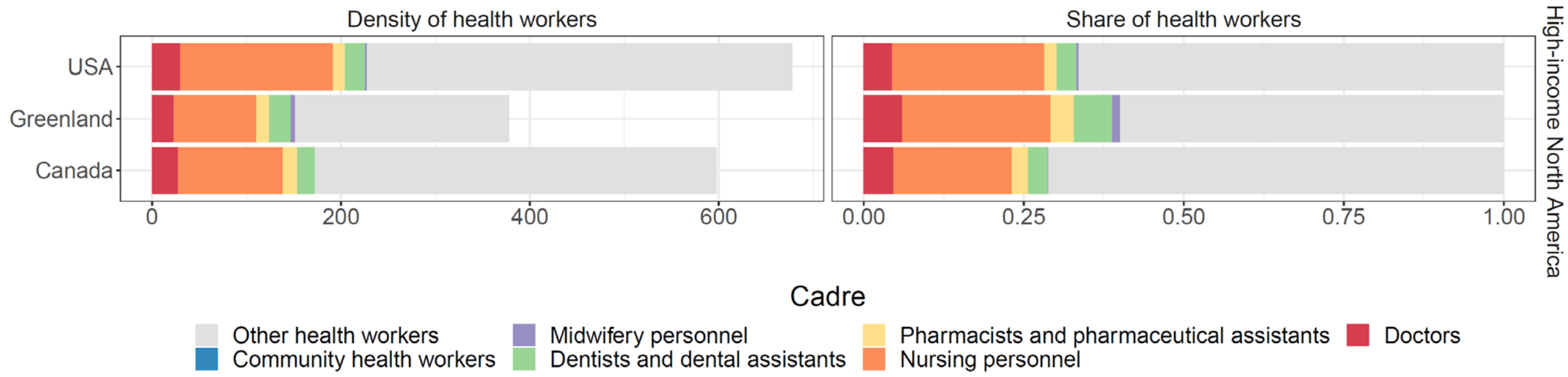

Appendix 2 Figure S7l: Caribbean, 2023

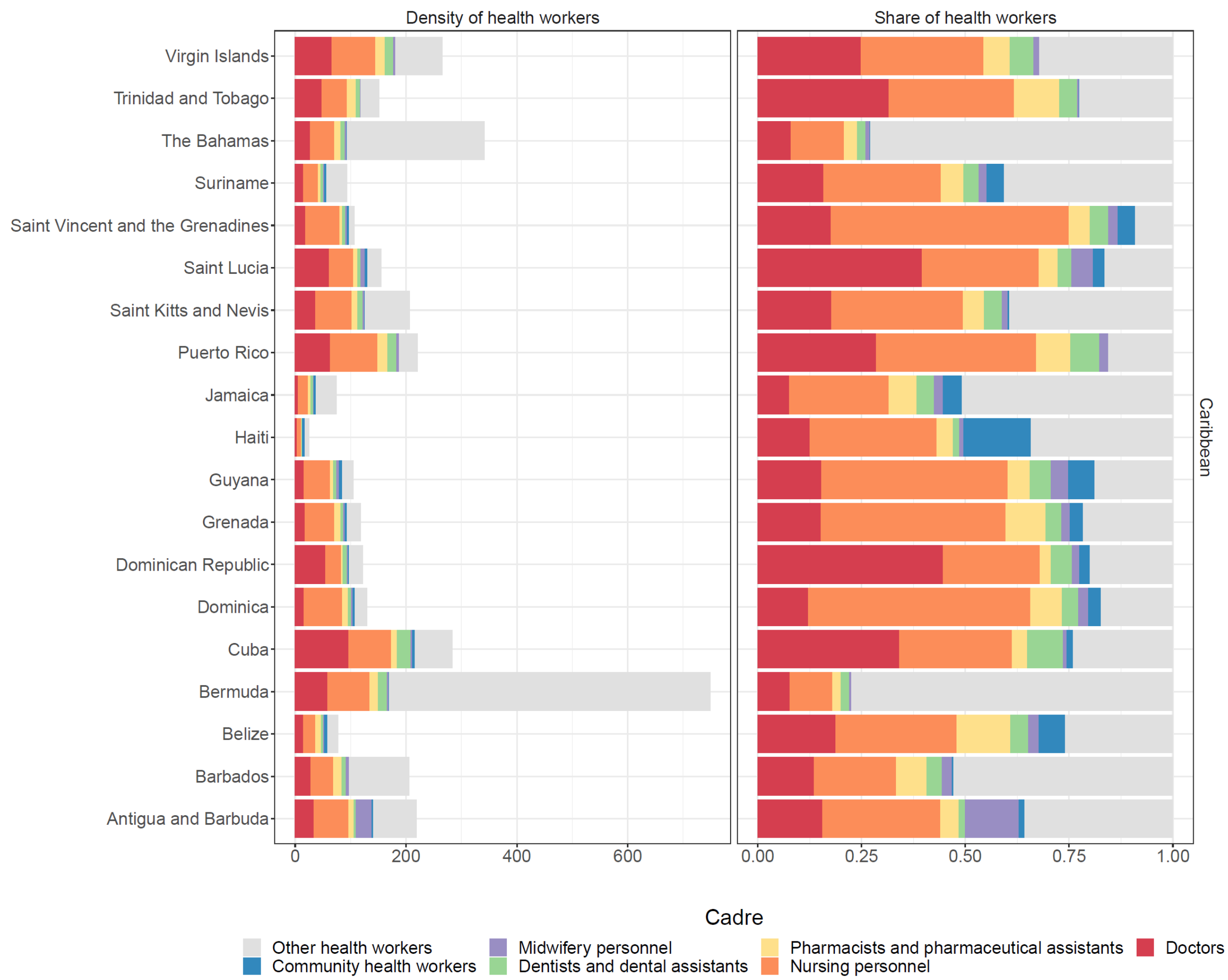

Appendix 2 Figure S7m: Andean Latin America, 2023

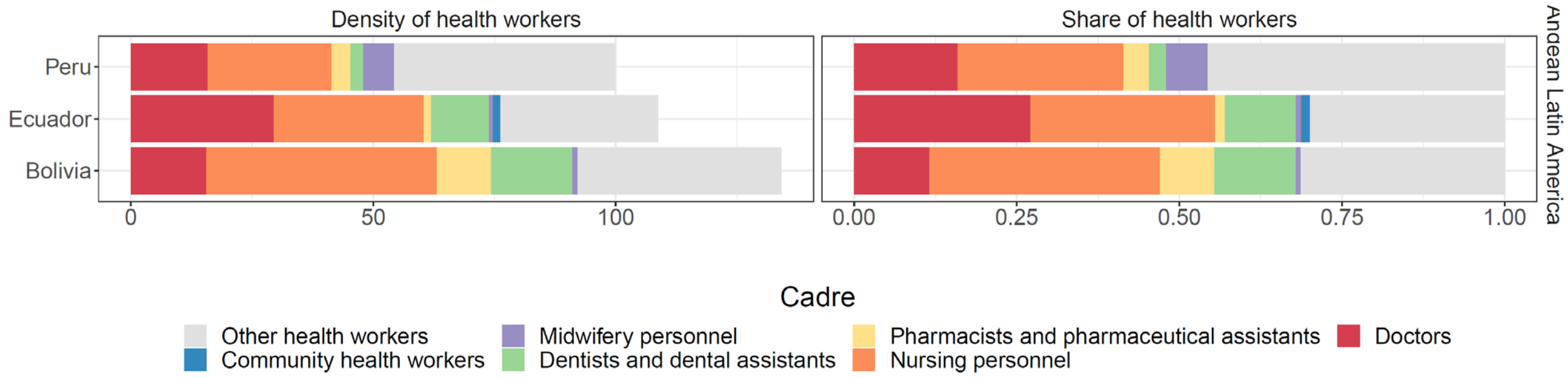

Appendix 2 Figure S7n: Central Latin America, 2023

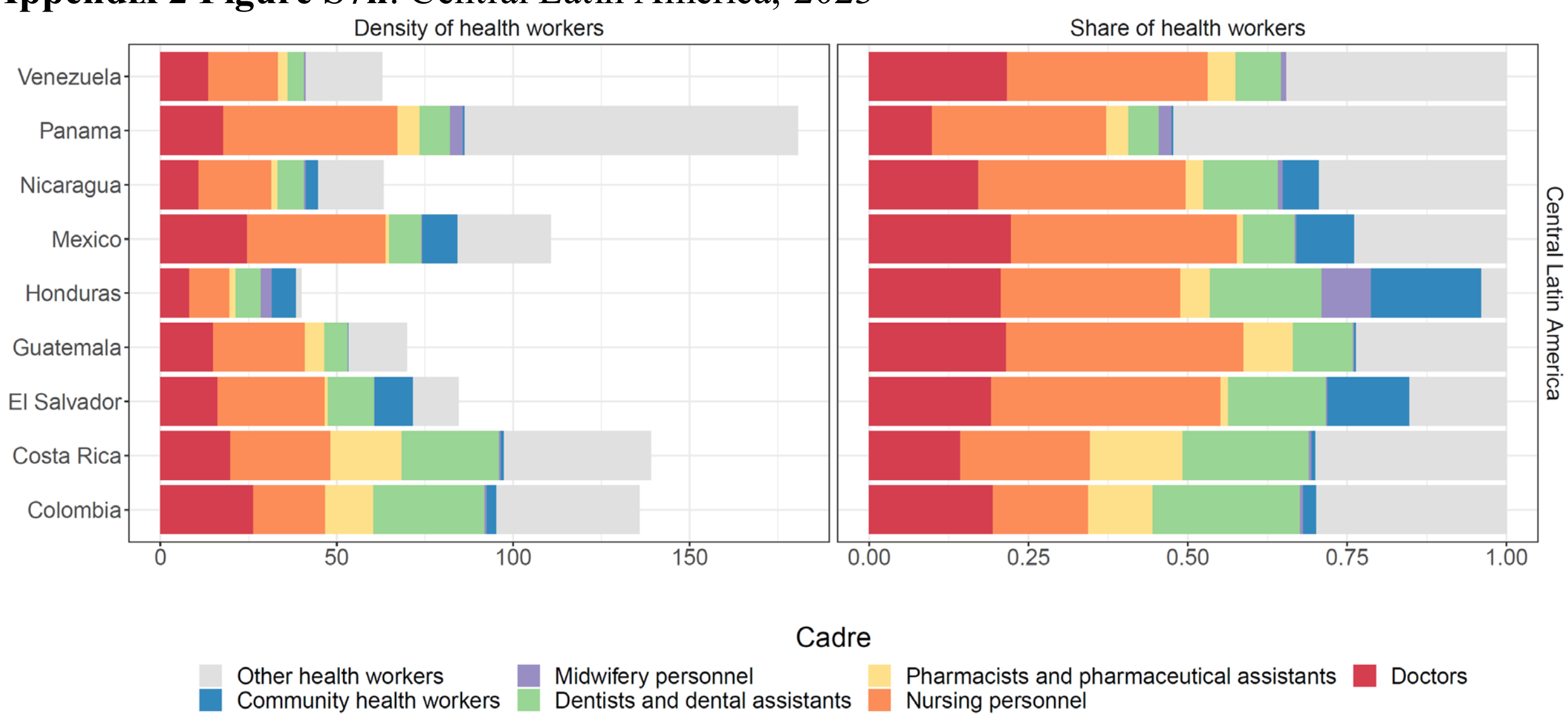

Appendix 2 Figure S7o: Tropical Latin America, 2023

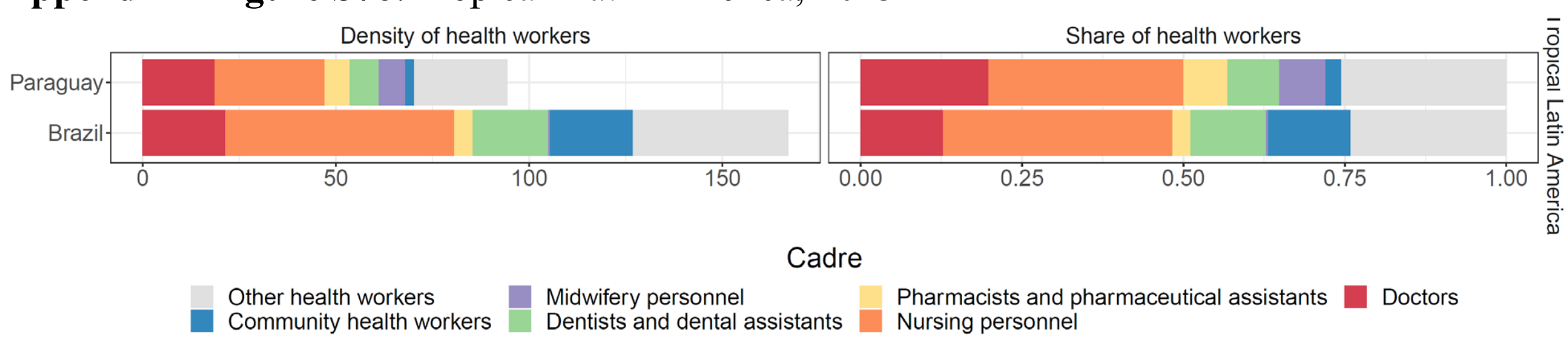

Appendix 2 Figure S7p: North Africa and Middle East, 2023

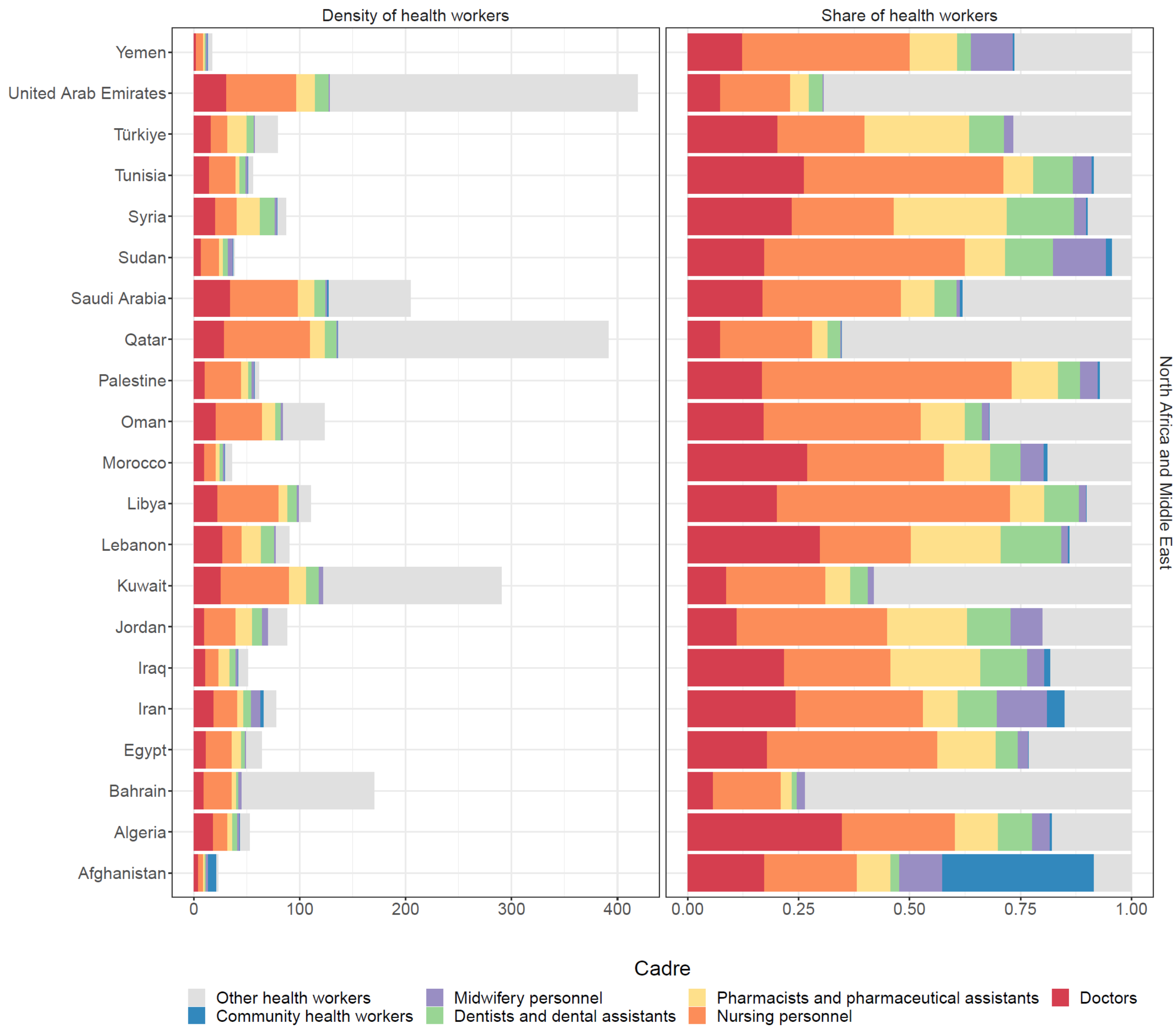

Appendix 2 Figure S7q: South Asia, 2023

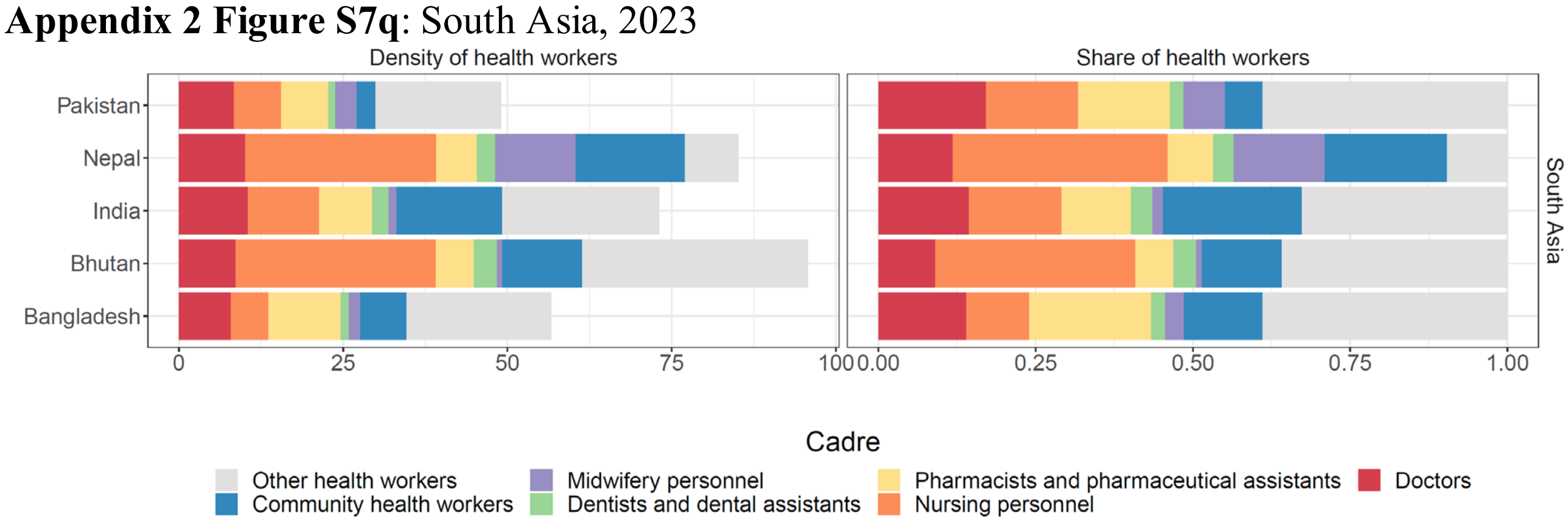

Appendix 2 Figure S7r: Central Sub−Saharan Africa, 2023

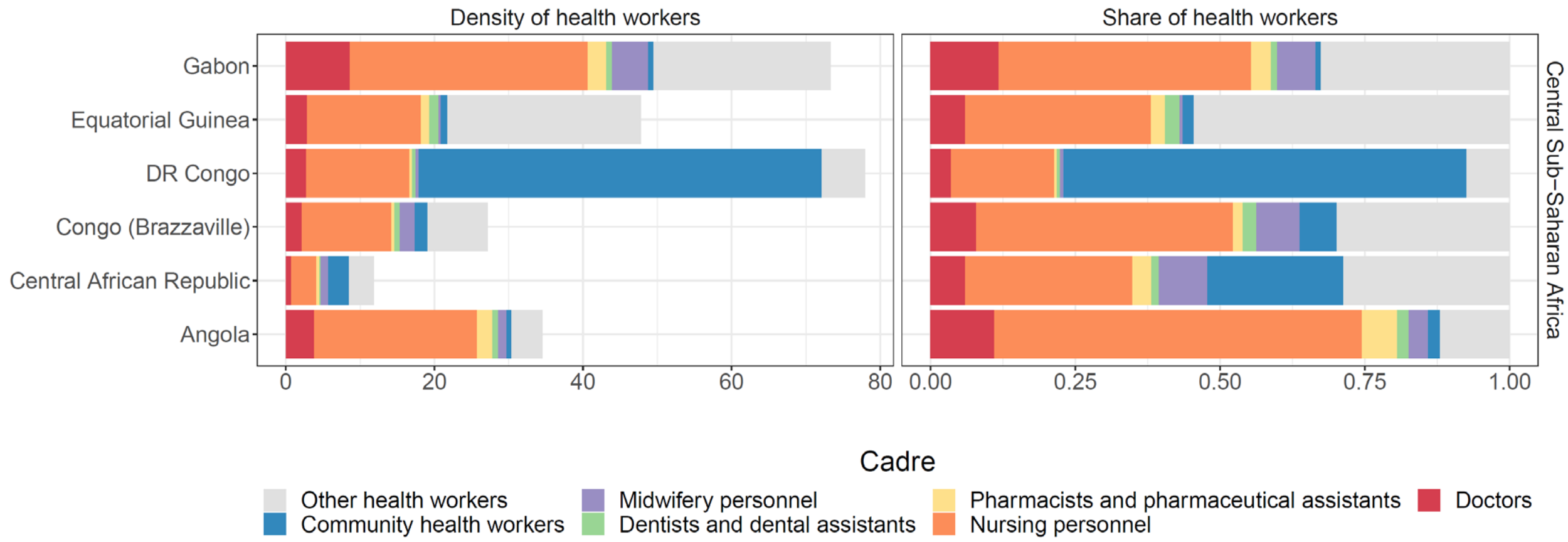

Appendix 2 Figure S7s: Eastern Sub−Saharan Africa, 2023

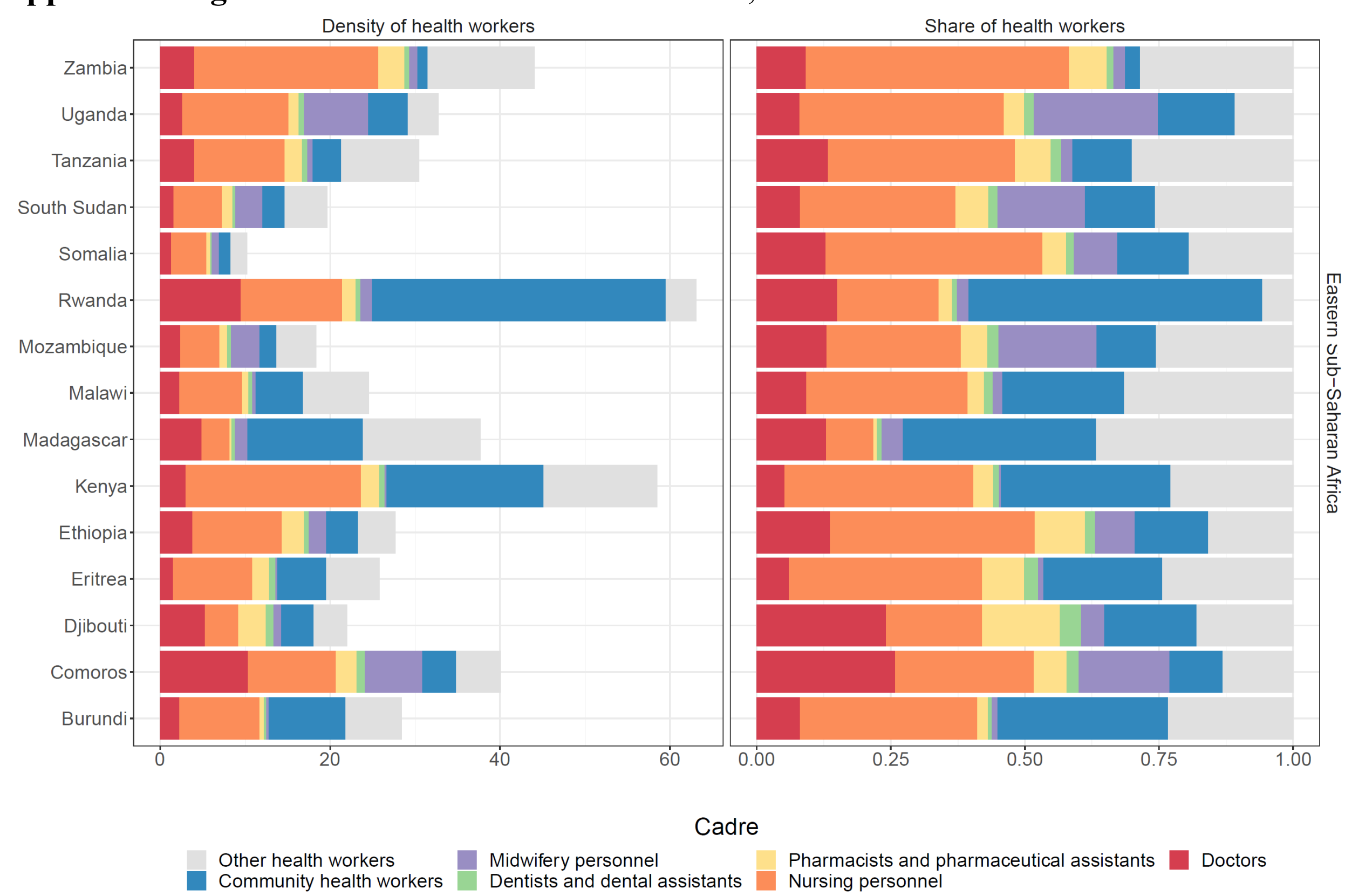

Appendix 2 Figure 7t: Southern Sub−Saharan Africa, 2023

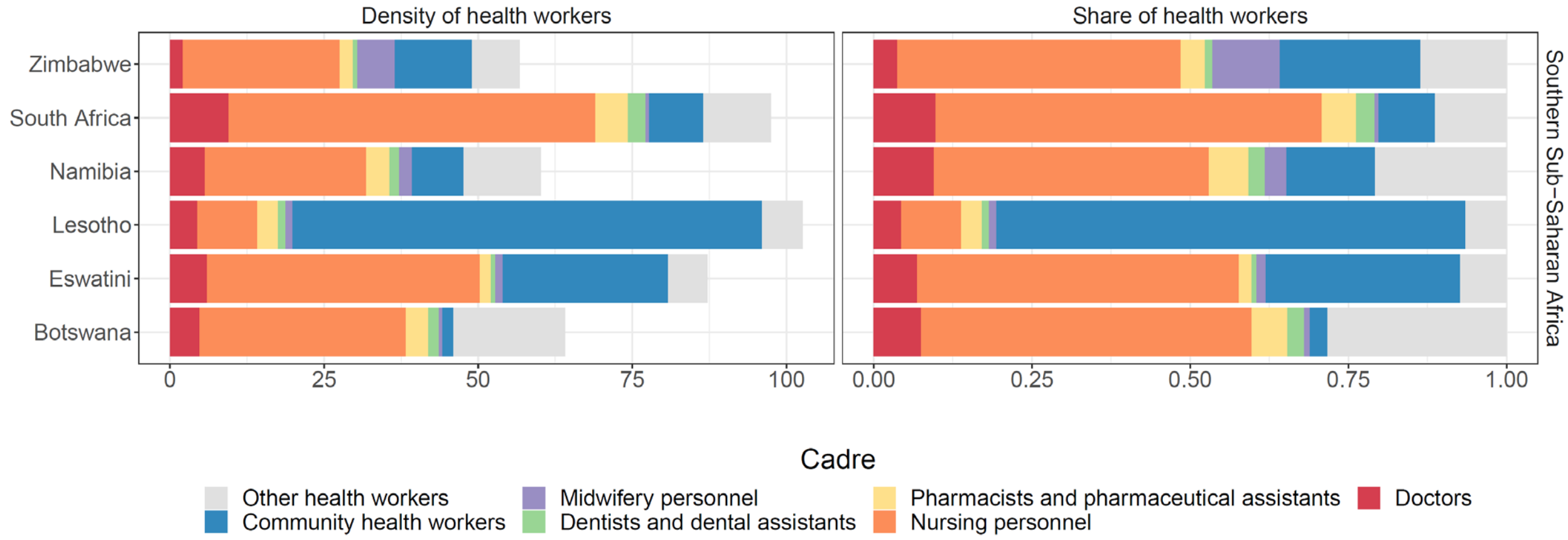

Appendix 2 Figure S7u: Western Sub-Saharan Africa, 2023

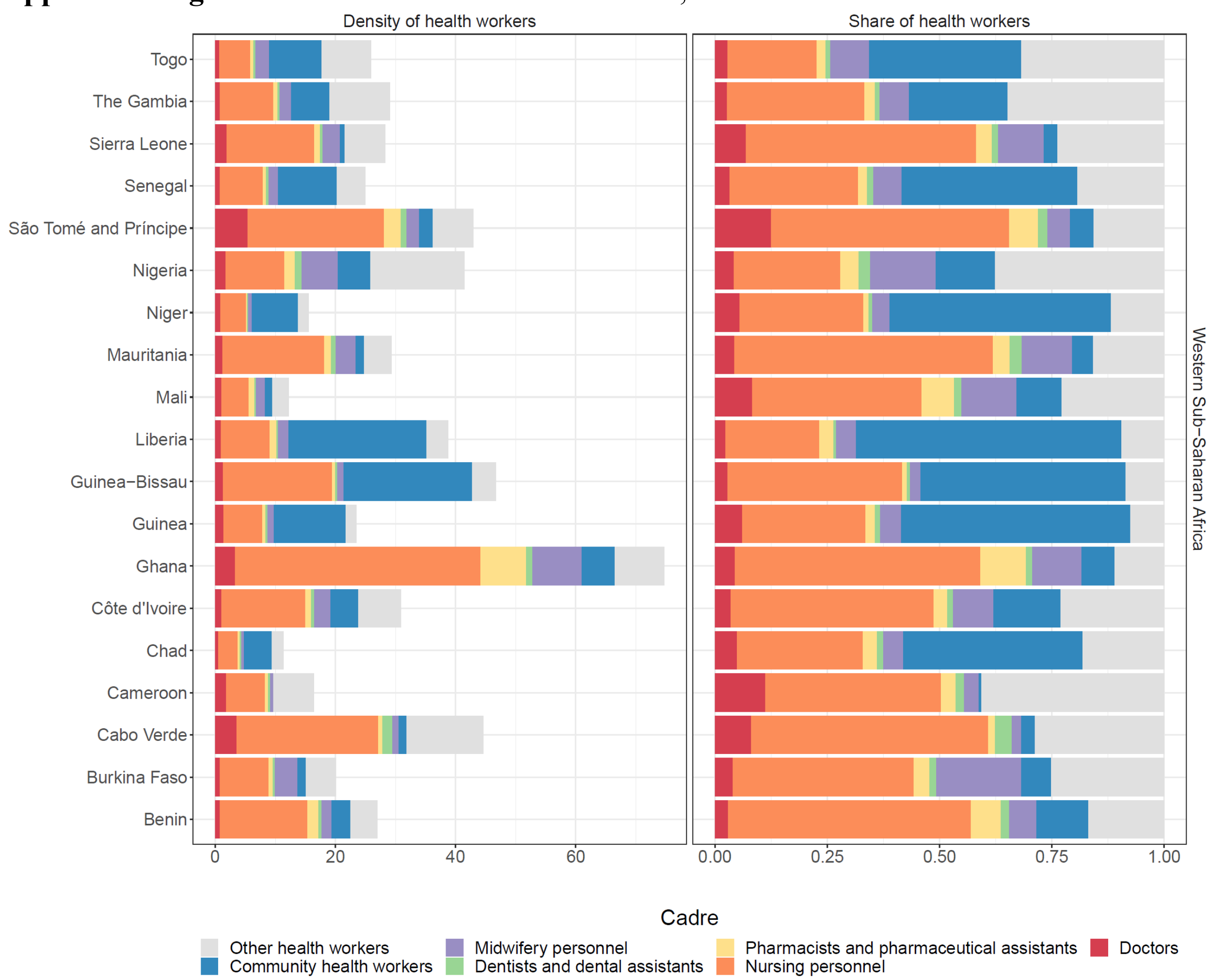

Appendix 2 Table S2: Additional count of workers in millions (95% UI) needed to achieve minimum density of workers for four cadres at UHC effective coverage of 80 out of 100 on the UHC effective coverage index by location, 2023

	Doctors	Nurses and midwives	Dentists	Pharmacists
**Central Europe, Eastern Europe, and Central Asia**				
Armenia	..	5,820<br>(4,830 to 6,830)	464<br>(372 to 531)	1,500<br>(1,390 to 1,640)
Azerbaijan	..	4,900<br>(1,450 to 8,410)	1,790<br>(1,470 to 2,020)	4,970<br>(4,600 to 5,450)
Georgia	..	..	..	..
Kazakhstan	..	..	5,310<br>(4,690 to 5,760)	4,190<br>(3,480 to 5,120)
Kyrgyzstan	3,450<br>(2,720 to 4,030)	17,400<br>(15,100 to 19,900)	2,440<br>(2,220 to 2,600)	3,760<br>(3,500 to 4,090)
Mongolia	..	1,660<br>(526 to 2,810)	635<br>(531 to 712)	..
Tajikistan	4,410<br>(3,340 to 5,260)	0<br>(0 to 824)	3,690<br>(3,370 to 3,930)	2,860<br>(2,490 to 3,350)
Turkmenistan	..	9,960<br>(8,120 to 11,800)	1,970<br>(1,800 to 2,100)	2,000<br>(1,810 to 2,260)
Uzbekistan	..	..	12,700<br>(11,600 to 13,500)	16,500<br>(15,300 to 18,100)
Albania	..	0<br>(0 to 565)	..	..
Bosnia and Herzegovina	1,280<br>(959 to 1,530)	v	749<br>(653 to 820)	1,070<br>(960 to 1,220)
Bulgaria	..	11,500<br>(9,260 to 13,800)	..	..
Croatia	..	..	..	..
Czechia	..	..	..	..
Hungary	3,940<br>(2,970 to 4,700)	7,970<br>(4,820 to 11,200)	..	..
Montenegro	308<br>(242 to 360)	381<br>(166 to 599)	301<br>(281 to 316)	194<br>(171 to 224)
North Macedonia	..	1,340<br>(738 to 1,960)	..	..
Poland	..	17,400<br>(4,580 to 30,400)	..	..
Romania	..	..	..	..
Serbia	..	10,100<br>(7,110 to 13,000)	2,780<br>(2,510 to 2,980)	2,170<br>(1,850 to 2,580)
Slovakia	..	..	..	..
Slovenia	..	..	..	..
Belarus	..	..	..	..
Estonia	..	..	..	..
Latvia	..	4,030<br>(3,410 to 4,660)	..	..
Lithuania	..	0<br>(0 to 500)	..	..

Republic of Moldova	..	3,590<br>(2,410 to 4,790)	..	0<br>(0 to 14)
Russian Federation	..	..	..	..
Ukraine	..	..	..	..
**High-income**				
Australia	..	..	..	..
New Zealand	..	..	..	..
Brunei Darussalam	195<br>(149 to 232)	0<br>(0 to 38)	120<br>(106 to 130)	138<br>(122 to 159)
Japan	..	..	..	..
Republic of Korea	..	..	..	..
Singapore	..	..	..	..
Canada	..	..	..	..
Greenland	6<br>(1 to 11)	..	..	..
United States of America	..	..	..	..
Argentina	..	82,700<br>(67,100 to 98,500)	..	0<br>(0 to 421)
Chile	..	..	..	6,060<br>(5,390 to 6,930)
Uruguay	..	..	..	1,470<br>(1,350 to 1,620)
Andorra	..	..	..	..
Austria	..	..	..	..
Belgium	..	..	..	..
Cyprus	..	..	..	..
Denmark	..	..	..	..
Finland	..	..	..	..
France	..	..	..	..
Germany	..	..	..	..
Greece	..	22,100<br>(18,600 to 25,600)	..	..
Iceland	..	..	..	..
Ireland	..	..	..	..
Israel	..	..	..	..
Italy	..	..	..	..
Luxembourg	..	..	..	..

Malta	..	..	..	..
Monaco	..	..	..	..
Netherlands	..	..	..	..
Norway	..	..	..	..
Portugal	..	..	..	..
San Marino	..	..	..	..
Spain	..	..	..	..
Sweden	..	..	..	..
Switzerland	..	..	..	..
United Kingdom	..	..	..	..
**Latin America and Caribbean**				
Bolivia (Plurinational State of)	9,930<br>(8,730 to 10,900)	19,100<br>(15,200 to 23,100)	..	..
Ecuador	..	58,000<br>(52,200 to 63,900)	..	8,770<br>(8,160 to 9,590)
Peru	28,700<br>(25,100 to 31,500)	117,000<br>(106,000 to 129,000)	10,200<br>(9,070 to 11,000)	14,100<br>(12,800 to 15,800)
Antigua and Barbuda	..	..	33<br>(30 to 35)	4<br>(1 to 9)
Bahamas	..	692<br>(557 to 830)	50<br>(38 to 60)	..
Barbados	..	566<br>(468 to 666)	24<br>(15 to 31)	..
Belize	385<br>(343 to 417)	1,660<br>(1,520 to 1,790)	127<br>(115 to 136)	..
Bermuda	..	..	..	..
Cuba	..	..	..	173<br>(0 to 692)
Dominica	56<br>(49 to 61)	..	25<br>(23 to 27)	0<br>(0 to 2)
Dominican Republic	..	37,900<br>(34,200 to 41,600)	646<br>(307 to 893)	4,660<br>(4,270 to 5,180)
Grenada	67<br>(55 to 76)	108<br>(71 to 146)	32<br>(28 to 34)	v
Guyana	638<br>(556 to 703)	1,060<br>(797 to 1,340)	353<br>(329 to 371)	300<br>(272 to 338)
Haiti	27,900<br>(26,600 to 29,000)	76,300<br>(71,900 to 80,800)	6,750<br>(6,340 to 7,050)	6,800<br>(6,330 to 7,420)
Jamaica	5,160<br>(4,880 to 5,390)	12,800<br>(11,900 to 13,800)	1,330<br>(1,240 to 1,390)	791<br>(692 to 921)
Puerto Rico	..	..	..	..
Saint Kitts and Nevis	..	..	4<br>(3 to 5)	0<br>(0 to 2)
Saint Lucia	..	229<br>(170 to 289)	40<br>(35 to 44)	21<br>(15 to 30)
Saint Vincent and the Grenadines	58<br>(47 to 68)	10<br>(0 to 48)	25<br>(22 to 28)	31<br>(27 to 36)

Suriname	533<br>(473 to 580)	2,150<br>(1,960 to 2,350)	237<br>(219 to 250)	264<br>(243 to 291)
Trinidad and Tobago	..	2,520<br>(2,070 to 2,980)	222<br>(180 to 252)	..
United States Virgin Islands	..	..	..	..
Colombia	..	226,000<br>(209,000 to 244,000)	..	18,100<br>(16,300 to 20,500)
Costa Rica	2,030<br>(1,510 to 2,450)	18,600<br>(16,800 to 20,300)	..	v
El Salvador	4,720<br>(4,100 to 5,210)	20,900<br>(18,900 to 23,000)	..	3,210<br>(2,990 to 3,490)
Guatemala	14,500<br>(12,900 to 15,800)	63,000<br>(57,600 to 68,500)	0<br>(0 to 312)	2,080<br>(1,510 to 2,840)
Honduras	16,700<br>(15,600 to 17,500)	53,700<br>(50,200 to 57,200)	..	4,890<br>(4,520 to 5,380)
Mexico	0<br>(0 to 162)	329,000<br>(285,000 to 373,000)	..	65,100<br>(60,500 to 71,100)
Nicaragua	8,830<br>(8,150 to 9,380)	29,500<br>(27,300 to 31,800)	428<br>(222 to 578)	3,150<br>(2,910 to 3,460)
Panama	2,520<br>(2,100 to 2,850)	4,780<br>(3,400 to 6,180)	v	1,140<br>(991 to 1,330)
Venezuela (Bolivarian Republic of)	26,200<br>(23,600 to 28,200)	113,000<br>(105,000 to 122,000)	7,500<br>(6,730 to 8,070)	10,200<br>(9,320 to 11,400)
Brazil	51,400<br>(30,200 to 68,300)	99,200<br>(29,800 to 170,000)	..	67,500<br>(60,100 to 77,200)
Paraguay	3,240<br>(2,610 to 3,750)	18,500<br>(16,400 to 20,600)	..	2,930<br>(2,710 to 3,220)
**North Africa and Middle East**				
Afghanistan	71,900<br>(68,200 to 74,800)	208,000<br>(196,000 to 220,000)	18,300<br>(17,200 to 19,100)	18,100<br>(16,900 to 19,800)
Algeria	24,600<br>(20,200 to 28,200)	218,000<br>(203,000 to 233,000)	6,140<br>(4,790 to 7,120)	4,940<br>(3,400 to 6,980)
Bahrain	2,210<br>(2,060 to 2,340)	5,500<br>(4,980 to 6,020)	668<br>(620 to 702)	489<br>(435 to 561)
Egypt	135,000<br>(124,000 to 144,000)	420,000<br>(384,000 to 457,000)	30,100<br>(26,700 to 32,500)	..
Iran (Islamic Republic of)	43,400<br>(34,600 to 50,400)	295,000<br>(266,000 to 324,000)	..	16,400<br>(13,300 to 20,400)
Iraq	56,200<br>(51,800 to 59,700)	222,000<br>(207,000 to 237,000)	1,250<br>(0 to 2,220)	..
Jordan	18,600<br>(17,300 to 19,700)	37,100<br>(32,800 to 41,500)	..	..
Kuwait	..	..	..	..
Lebanon	..	25,500<br>(23,600 to 27,400)	..	..
Libya	1,170<br>(427 to 1,760)	3,550<br>(1,130 to 6,020)	..	68<br>(0 to 408)
Morocco	52,000<br>(48,300 to 55,000)	190,000<br>(178,000 to 202,000)	11,800<br>(10,700 to 12,600)	7,710<br>(6,420 to 9,400)
Oman	1,390<br>(873 to 1,800)	9,680<br>(7,990 to 11,400)	711<br>(556 to 824)	..
Palestine	7,310<br>(6,770 to 7,740)	14,800<br>(13,000 to 16,600)	1,390<br>(1,220 to 1,510)	0<br>(0 to 185)
Qatar	..	..	..	..
Saudi Arabia	..	..	..	..

Sudan	64,300 (60,500 to 67,300)	159,000 (146,000 to 171,000)	5,170 (4,040 to 6,000)	13,000 (11,700 to 14,700)
Syrian Arab Republic	5,880 (4,110 to 7,300)	74,900 (69,100 to 80,800)	..	..
Tunisia	11,100 (9,860 to 12,000)	44,400 (40,500 to 48,400)	1,100 (739 to 1,370)	3,270 (2,860 to 3,820)
Türkiye	66,500 (58,000 to 73,400)	406,000 (378,000 to 434,000)	0 (0 to 1,720)	21,900 (18,900 to 25,800)
United Arab Emirates	..	..	..	..
Yemen	77,100 (73,500 to 79,900)	200,000 (188,000 to 212,000)	17,400 (16,300 to 18,200)	16,900 (15,600 to 18,500)
**South Asia**				
Bangladesh	276,000 (259,000 to 290,000)	992,000 (936,000 to 1,050,000)	76,500 (71,300 to 80,400)	71,600 (65,600 to 79,600)
Bhutan	1,130 (1,060 to 1,190)	2,470 (2,230 to 2,720)	170 (147 to 186)	199 (173 to 234)
India	1,920,000 (1,770,000 to 2,030,000)	7,570,000 (7,100,000 to 8,050,000)	544,000 (501,000 to 576,000)	148,000 (98,100 to 214,000)
Nepal	42,600 (39,500 to 45,100)	71,800 (61,600 to 82,100)	10,900 (10,000 to 11,600)	10,300 (9,200 to 11,700)
Pakistan	376,000 (352,000 to 396,000)	1,320,000 (1,240,000 to 1,400,000)	113,000 (106,000 to 119,000)	108,000 (99,200 to 119,000)
**Southeast Asia, East Asia, and Oceania**				
China	..	1,890,000 (1,420,000 to 2,370,000)	..	200,000 (150,000 to 266,000)
Democratic People's Republic of Korea	36,600 (34,000 to 38,700)	41,000 (32,400 to 49,800)	5,890 (5,090 to 6,470)	3,070 (2,150 to 4,280)
Taiwan	..	..	..	..
American Samoa	38 (33 to 42)	..	6 (4 to 7)	12 (10 to 14)
Cook Islands	9 (8 to 10)	..	..	5 (4 to 6)
Fiji	1,120 (1,030 to 1,200)	2,290 (1,990 to 2,610)	229 (201 to 250)	287 (254 to 330)
Guam	0 (0 to 10)	..	..	7 (2 to 15)
Kiribati	251 (239 to 262)	115 (73 to 158)	54 (51 to 57)	63 (59 to 69)
Marshall Islands	64 (60 to 67)	43 (30 to 57)	15 (14 to 16)	18 (16 to 19)
Micronesia (Federated States of)	136 (126 to 145)	373 (339 to 407)	37 (34 to 39)	46 (43 to 51)
Nauru	12 (11 to 13)	..	2 (2 to 3)	4 (4 to 5)
Niue	1 (1 to 1)	..	..	0 (0 to 1)
Northern Mariana Islands	12 (7 to 15)	..	..	7 (6 to 9)
Palau	1 (0 to 3)	..	2 (1 to 2)	4 (4 to 5)
Papua New Guinea	25,400 (24,300 to 26,300)	64,300 (60,700 to 68,000)	5,660 (5,320 to 5,900)	6,030 (5,640 to 6,540)
Samoa	383 (361 to 400)	674 (603 to 747)	90 (83 to 95)	113 (105 to 123)

Solomon Islands	1,810<br>(1,720 to 1,880)	3,690<br>(3,410 to 3,980)	395<br>(369 to 414)	395<br>(365 to 435)
Tokelau	1<br>(0 to 1)	..	..	..
Tonga	131<br>(121 to 140)	153<br>(119 to 188)	36<br>(32 to 38)	46<br>(43 to 51)
Tuvalu	11<br>(10 to 12)	8<br>(4 to 12)	4<br>(4 to 4)	3<br>(3 to 4)
Vanuatu	712<br>(679 to 738)	1,450<br>(1,350 to 1,560)	155<br>(145 to 162)	163<br>(151 to 178)
Cambodia	28,800<br>(27,100 to 30,200)	80,100<br>(74,400 to 85,800)	8,250<br>(7,730 to 8,630)	7,880<br>(7,280 to 8,670)
Indonesia	496,000<br>(467,000 to 519,000)	614,000<br>(520,000 to 710,000)	123,000<br>(114,000 to 129,000)	92,600<br>(82,600 to 106,000)
Lao People's Democratic Republic	14,600<br>(13,800 to 15,200)	36,800<br>(34,300 to 39,300)	3,300<br>(3,080 to 3,470)	2,210<br>(1,950 to 2,550)
Malaysia	0<br>(0 to 366)	58,800<br>(47,600 to 70,200)	3,550<br>(2,520 to 4,300)	..
Maldives	331<br>(276 to 374)	..	201<br>(184 to 213)	..
Mauritius	..	2,010<br>(1,590 to 2,430)	155<br>(116 to 183)	0<br>(0 to 32)
Myanmar	87,500<br>(81,800 to 92,100)	289,000<br>(270,000 to 308,000)	25,200<br>(23,500 to 26,500)	25,600<br>(23,600 to 28,200)
Philippines	167,000<br>(156,000 to 176,000)	457,000<br>(419,000 to 495,000)	26,700<br>(23,300 to 29,300)	8,320<br>(4,340 to 13,600)
Seychelles	..	..	4<br>(1 to 6)	v
Sri Lanka	27,500<br>(25,200 to 29,300)	83,600<br>(76,100 to 91,100)	9,630<br>(8,950 to 10,100)	9,770<br>(8,980 to 10,800)
Thailand	78,100<br>(71,600 to 83,400)	219,000<br>(198,000 to 241,000)	22,900<br>(20,900 to 24,400)	16,500<br>(14,200 to 19,500)
Timor-Leste	2,110<br>(1,970 to 2,230)	6,630<br>(6,160 to 7,110)	723<br>(680 to 755)	386<br>(336 to 452)
Viet Nam	126,000<br>(116,000 to 134,000)	371,000<br>(338,000 to 406,000)	35,900<br>(32,800 to 38,200)	18,800<br>(15,200 to 23,500)
**Sub-Saharan Africa**				
Angola	72,200<br>(68,600 to 75,100)	149,000<br>(137,000 to 161,000)	17,700<br>(16,600 to 18,500)	15,000<br>(13,800 to 16,700)
Central African Republic	15,200<br>(14,500 to 15,700)	39,400<br>(37,300 to 41,600)	3,390<br>(3,190 to 3,530)	3,630<br>(3,400 to 3,930)
Congo	13,300<br>(12,700 to 13,800)	30,800<br>(28,800 to 32,900)	3,110<br>(2,930 to 3,250)	3,320<br>(3,100 to 3,600)
Democratic Republic of the Congo	199,000<br>(189,000 to 206,000)	472,000<br>(441,000 to 504,000)	48,100<br>(45,200 to 50,100)	50,800<br>(47,500 to 55,100)
Equatorial Guinea	3,410<br>(3,240 to 3,540)	7,950<br>(7,420 to 8,490)	791<br>(742 to 827)	877<br>(821 to 952)
Gabon	2,910<br>(2,720 to 3,060)	5,280<br>(4,650 to 5,920)	935<br>(878 to 978)	815<br>(748 to 903)
Burundi	30,700<br>(29,300 to 31,900)	78,200<br>(73,500 to 82,900)	7,340<br>(6,910 to 7,650)	7,410<br>(6,910 to 8,060)
Comoros	1,190<br>(1,100 to 1,260)	4,170<br>(3,880 to 4,470)	411<br>(384 to 430)	327<br>(297 to 368)
Djibouti	2,570<br>(2,440 to 2,690)	8,280<br>(7,820 to 8,740)	648<br>(606 to 679)	427<br>(378 to 490)
Eritrea	15,600<br>(14,900 to 16,200)	38,600<br>(36,300 to 40,900)	3,460<br>(3,240 to 3,610)	2,790<br>(2,540 to 3,110)
Ethiopia	227,000<br>(216,000 to 236,000)	588,000<br>(551,000 to 626,000)	54,500<br>(51,100 to 57,000)	42,600<br>(38,600 to 47,800)

Kenya	108,000 (103,000 to 113,000)	227,000 (210,000 to 245,000)	25,200 (23,600 to 26,400)	23,600 (21,800 to 26,000)
Madagascar	58,400 (55,300 to 60,900)	184,000 (174,000 to 194,000)	15,400 (14,500 to 16,100)	16,900 (15,800 to 18,300)
Malawi	44,700 (42,600 to 46,300)	117,000 (111,000 to 124,000)	10,400 (9,820 to 10,900)	10,600 (9,920 to 11,600)
Mozambique	71,400 (68,000 to 74,000)	188,000 (177,000 to 199,000)	16,700 (15,700 to 17,500)	17,500 (16,300 to 19,000)
Rwanda	20,000 (18,600 to 21,100)	71,300 (66,800 to 76,000)	6,880 (6,450 to 7,180)	5,920 (5,430 to 6,560)
Somalia	49,300 (47,100 to 51,000)	130,000 (123,000 to 137,000)	11,200 (10,500 to 11,700)	11,600 (10,800 to 12,600)
South Sudan	21,900 (20,900 to 22,700)	54,700 (51,500 to 58,000)	5,040 (4,740 to 5,260)	4,620 (4,280 to 5,070)
Uganda	96,600 (92,100 to 100,000)	203,000 (188,000 to 218,000)	22,900 (21,500 to 23,900)	22,200 (20,600 to 24,300)
United Republic of Tanzania	129,000 (123,000 to 135,000)	348,000 (327,000 to 370,000)	32,000 (30,000 to 33,400)	27,900 (25,600 to 30,900)
Zambia	41,400 (39,300 to 43,100)	87,700 (80,900 to 94,700)	10,300 (9,680 to 10,800)	6,850 (6,120 to 7,810)
Botswana	4,650 (4,410 to 4,850)	7,420 (6,620 to 8,230)	936 (862 to 990)	981 (896 to 1,090)
Eswatini	2,110 (1,990 to 2,210)	2,240 (1,850 to 2,640)	575 (540 to 602)	602 (560 to 656)
Lesotho	3,720 (3,520 to 3,870)	10,300 (9,650 to 10,900)	863 (805 to 905)	734 (667 to 822)
Namibia	5,460 (5,160 to 5,700)	10,900 (9,930 to 11,900)	1,250 (1,160 to 1,320)	993 (888 to 1,130)
South Africa	91,400 (85,000 to 96,500)	28,200 (7,200 to 49,500)	23,400 (21,500 to 24,900)	24,900 (22,600 to 27,800)
Zimbabwe	34,600 (33,000 to 35,900)	52,500 (47,300 to 57,800)	7,770 (7,290 to 8,120)	6,600 (6,050 to 7,330)
Benin	32,800 (31,400 to 33,900)	68,600 (63,900 to 73,300)	6,890 (6,460 to 7,200)	6,110 (5,620 to 6,770)
Burkina Faso	55,900 (53,500 to 57,800)	128,000 (120,000 to 136,000)	12,400 (11,600 to 12,900)	12,400 (11,500 to 13,500)
Cabo Verde	1,000 (951 to 1,040)	1,980 (1,810 to 2,140)	246 (231 to 257)	260 (243 to 283)
Cameroon	74,500 (71,100 to 77,200)	195,000 (184,000 to 206,000)	17,100 (16,100 to 17,900)	18,000 (16,800 to 19,600)
Chad	44,900 (42,900 to 46,400)	117,000 (111,000 to 124,000)	9,950 (9,360 to 10,400)	10,500 (9,810 to 11,400)
Côte d'Ivoire	76,900 (73,600 to 79,600)	162,000 (150,000 to 173,000)	16,900 (15,900 to 17,600)	16,800 (15,700 to 18,400)
Gambia	5,810 (5,560 to 6,010)	13,500 (12,700 to 14,400)	1,290 (1,210 to 1,340)	1,390 (1,300 to 1,500)
Ghana	68,300 (65,000 to 71,000)	51,400 (40,500 to 62,500)	14,900 (13,900 to 15,600)	0 (0 to 827)
Guinea	31,800 (30,300 to 32,900)	80,700 (76,100 to 85,400)	7,230 (6,800 to 7,540)	7,380 (6,890 to 8,030)
Guinea-Bissau	4,890 (4,670 to 5,060)	9,800 (9,090 to 10,500)	1,100 (1,040 to 1,150)	1,200 (1,120 to 1,300)
Liberia	13,100 (12,600 to 13,600)	31,300 (29,400 to 33,200)	2,950 (2,780 to 3,070)	2,980 (2,780 to 3,240)
Mali	55,300 (52,900 to 57,200)	141,000 (133,000 to 149,000)	12,400 (11,700 to 13,000)	11,900 (11,100 to 13,000)
Mauritania	10,400 (9,910 to 10,700)	20,400 (18,800 to 21,900)	2,210 (2,070 to 2,310)	2,170 (2,010 to 2,380)
Niger	61,200 (58,500 to 63,300)	159,000 (150,000 to 168,000)	13,700 (12,900 to 14,300)	14,700 (13,800 to 15,900)

Nigeria	541,000<br>(517,000 to 561,000)	1,190,000<br>(1,110,000 to 1,270,000)	117,000<br>(109,000 to 122,000)	114,000<br>(106,000 to 125,000)
Sao Tome and Principe	409<br>(387 to 427)	878<br>(806 to 952)	107<br>(101 to 112)	80<br>(72 to 90)
Senegal	43,100<br>(41,300 to 44,600)	104,000<br>(98,300 to 111,000)	9,420<br>(8,850 to 9,830)	9,810<br>(9,160 to 10,700)
Sierra Leone	17,500<br>(16,700 to 18,200)	37,700<br>(35,100 to 40,400)	4,100<br>(3,850 to 4,270)	4,200<br>(3,920 to 4,570)
Togo	19,800<br>(18,900 to 20,400)	48,800<br>(46,000 to 51,700)	4,360<br>(4,100 to 4,550)	4,470<br>(4,180 to 4,870)

* Locations where health worker densities from 2023 surpassed the minimum density threshold for UHC effective coverage 80 or achieved UHC effective coverage levels of 80 or above in 2023 are depicted with ".."

Appendix 2 Table S3: Additional count of workers in millions (95% UI) needed to achieve minimum density of workers for four cadres at UHC effective coverage of 90 out of 100 on the UHC effective coverage index by GBD super-region, 2023

	Number of countries with shortage	Sum of country-level shortage (number of workers)
**Doctors (threshold: 37·9 per 10,000 population)**		
**Global**	158<br>(157 to 159)	15,000,000<br>(14,600,000 to 15,400,000)
Central Europe, Eastern Europe, and Central Asia	20<br>(20 to 20)	279,000<br>(263,000 to 294,000)
High-income	7<br>(7 to 8)	266,000<br>(245,000 to 285,000)
Latin America and Caribbean	26<br>(25 to 26)	915,000<br>(884,000 to 944,000)
North Africa and Middle East	21<br>(21 to 21)	1,440,000<br>(1,400,000 to 1,470,000)
South Asia	5<br>(5 to 5)	5,120,000<br>(5,020,000 to 5,220,000)
Southeast Asia, East Asia, and Oceania	33<br>(33 to 33)	2,900,000<br>(2,780,000 to 3,010,000)
Sub-Saharan Africa	46<br>(46 to 46)	4,120,000<br>(4,060,000 to 4,180,000)
**Nurses and midwives (threshold: 99·9 per 10,000 population)**		
**Global**	171<br>(170 to 172)	48,000,000<br>(45,900,000 to 51,400,000)
Central Europe, Eastern Europe, and Central Asia	26<br>(26 to 26)	819,000<br>(703,000 to 999,000)
High-income	12<br>(12 to 12)	455,000<br>(401,000 to 540,000)
Latin America and Caribbean	33<br>(33 to 33)	3,370,000<br>(3,190,000 to 3,660,000)
North Africa and Middle East	21<br>(21 to 21)	4,780,000<br>(4,580,000 to 5,080,000)
South Asia	5<br>(5 to 5)	16,700,000<br>(16,100,000 to 17,600,000)
Southeast Asia, East Asia, and Oceania	28<br>(27 to 29)	12,000,000<br>(11,300,000 to 13,000,000)
Sub-Saharan Africa	46<br>(46 to 46)	9,980,000<br>(9,610,000 to 10,600,000)
**Dentists (threshold: 8·1 per 10,000 population)**		
**Global**	151<br>(148 to 154)	3,300,000<br>(3,210,000 to 3,400,000)

Central Europe, Eastern Europe, and Central Asia	18<br>(16 to 18)	97,800<br>(92,500 to 104,000)
High-income	11<br>(10 to 11)	152,000<br>(143,000 to 161,000)
Latin America and Caribbean	24<br>(24 to 26)	93,900<br>(89,400 to 98,800)
North Africa and Middle East	18<br>(18 to 18)	249,000<br>(239,000 to 260,000)
South Asia	5<br>(5 to 5)	1,290,000<br>(1,260,000 to 1,320,000)
Southeast Asia, East Asia, and Oceania	29<br>(29 to 30)	488,000<br>(476,000 to 501,000)
Sub-Saharan Africa	46<br>(46 to 46)	931,000<br>(911,000 to 952,000)
**Pharmacists (threshold: 8·8 per 10,000 population)**		
**Global**	171<br>(170 to 172)	48,000,000<br>(45,900,000 to 51,400,000)
Central Europe, Eastern Europe, and Central Asia	26<br>(26 to 26)	819,000<br>(703,000 to 999,000)
High-income	12<br>(12 to 12)	455,000<br>(401,000 to 540,000)
Latin America and Caribbean	33<br>(33 to 33)	3,370,000<br>(3,190,000 to 3,660,000)
North Africa and Middle East	21<br>(21 to 21)	4,780,000<br>(4,580,000 to 5,080,000)
South Asia	5<br>(5 to 5)	16,700,000<br>(16,100,000 to 17,600,000)
Southeast Asia, East Asia, and Oceania	28<br>(27 to 29)	12,000,000<br>(11,300,000 to 13,000,000)
Sub-Saharan Africa	46<br>(46 to 46)	9,980,000<br>(9,610,000 to 10,600,000)

Appendix 2 Figure S8: Trends in global sex-specific health worker densities by cadre, 1990-2023

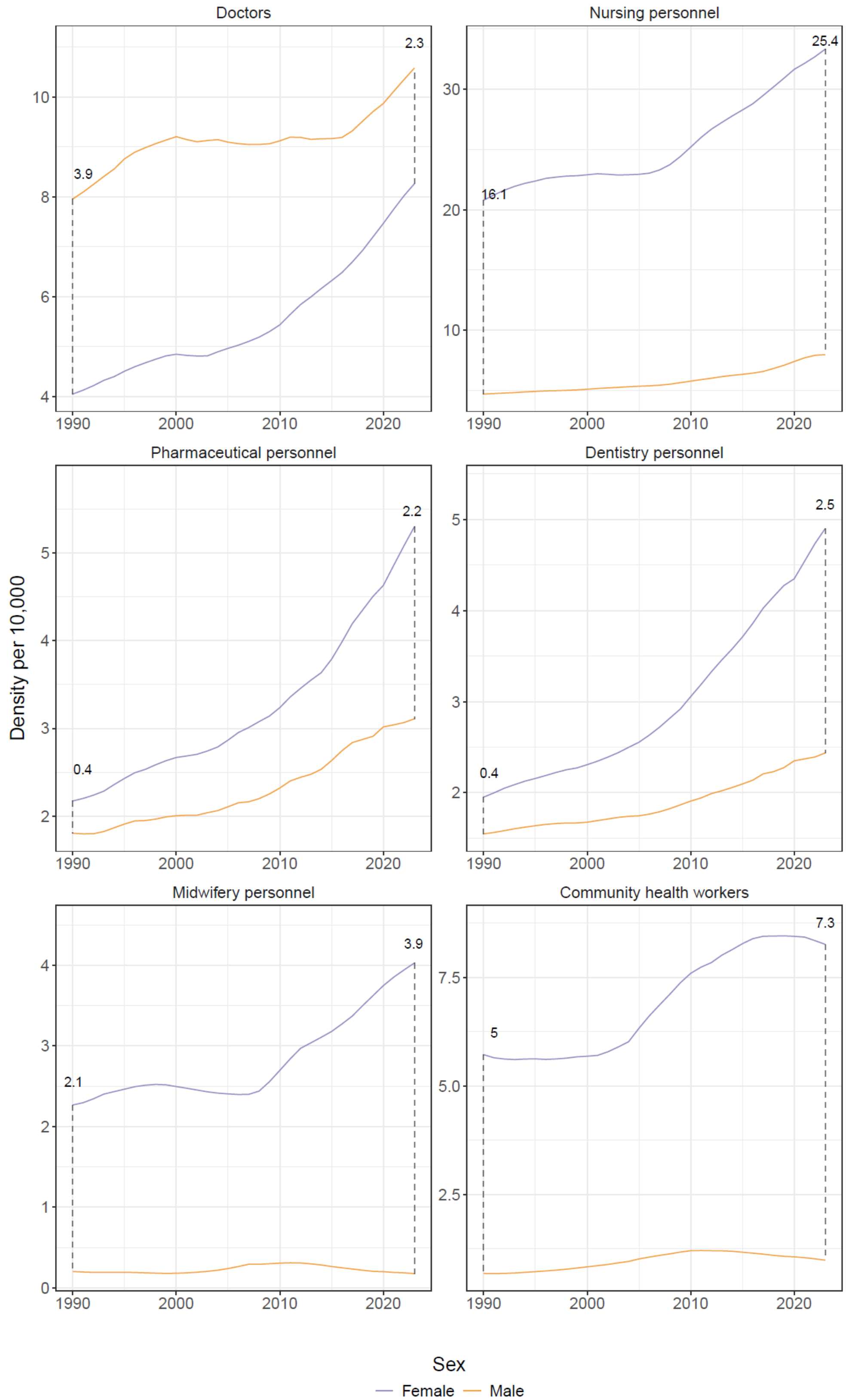

Appendix 2 Figure S9: Nurse−to−doctor ratio by country, 2023

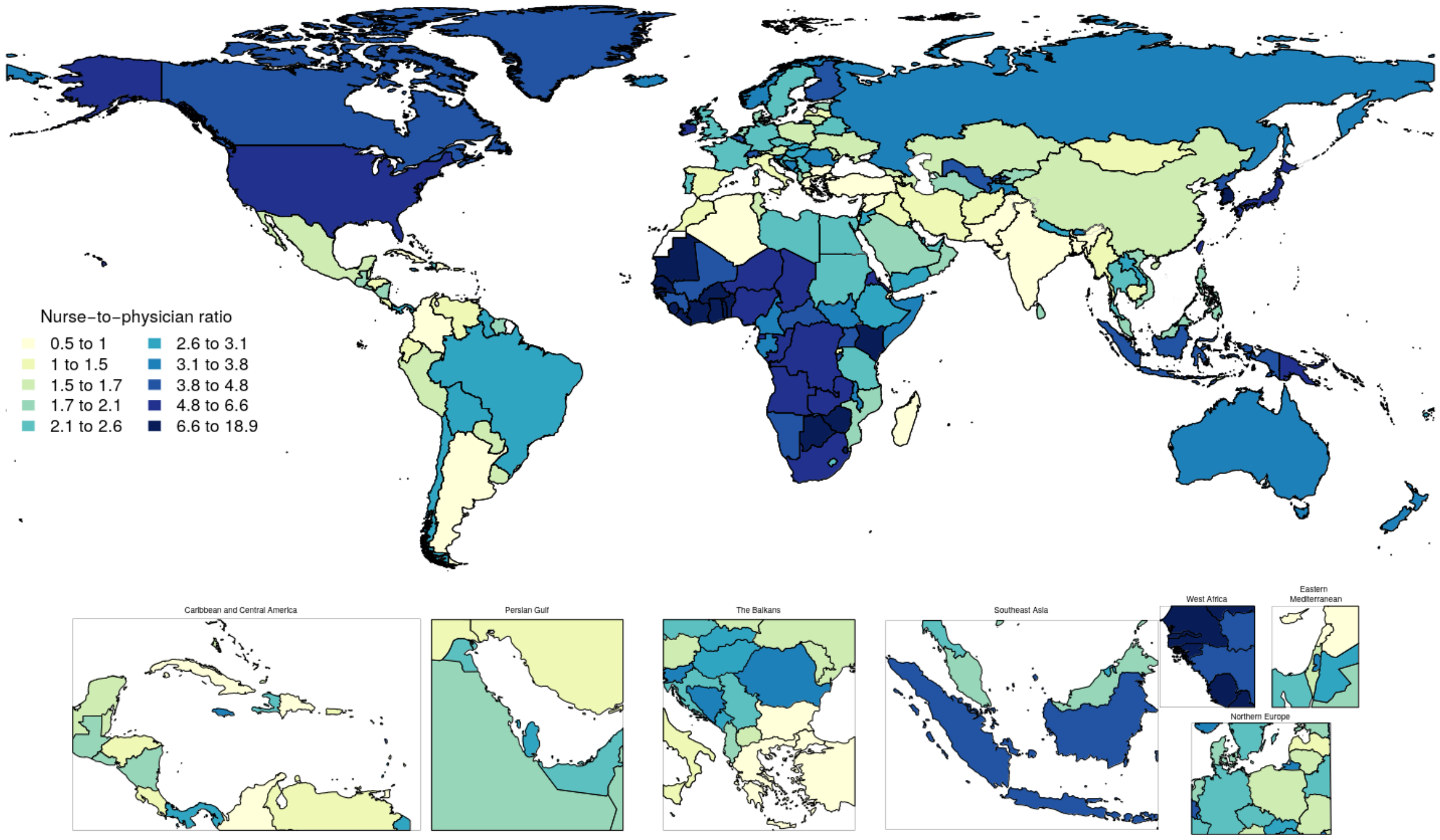

Appendix 2 Figure S10a: Absolute difference in densities of all health workers by country, 1990−2023

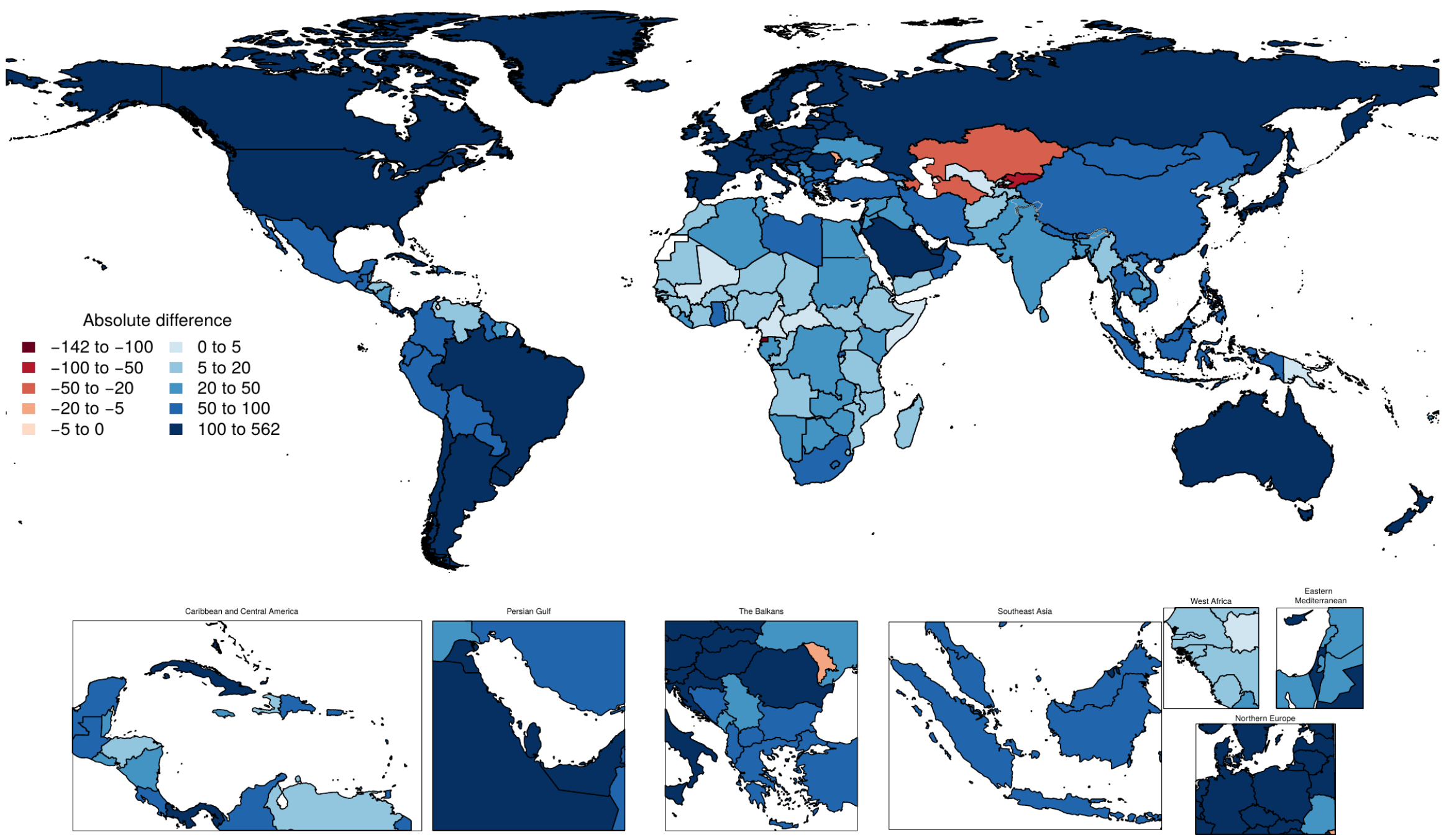

Appendix 2 Figure S10b: Absolute difference in densities of doctors by country, 1990−2023

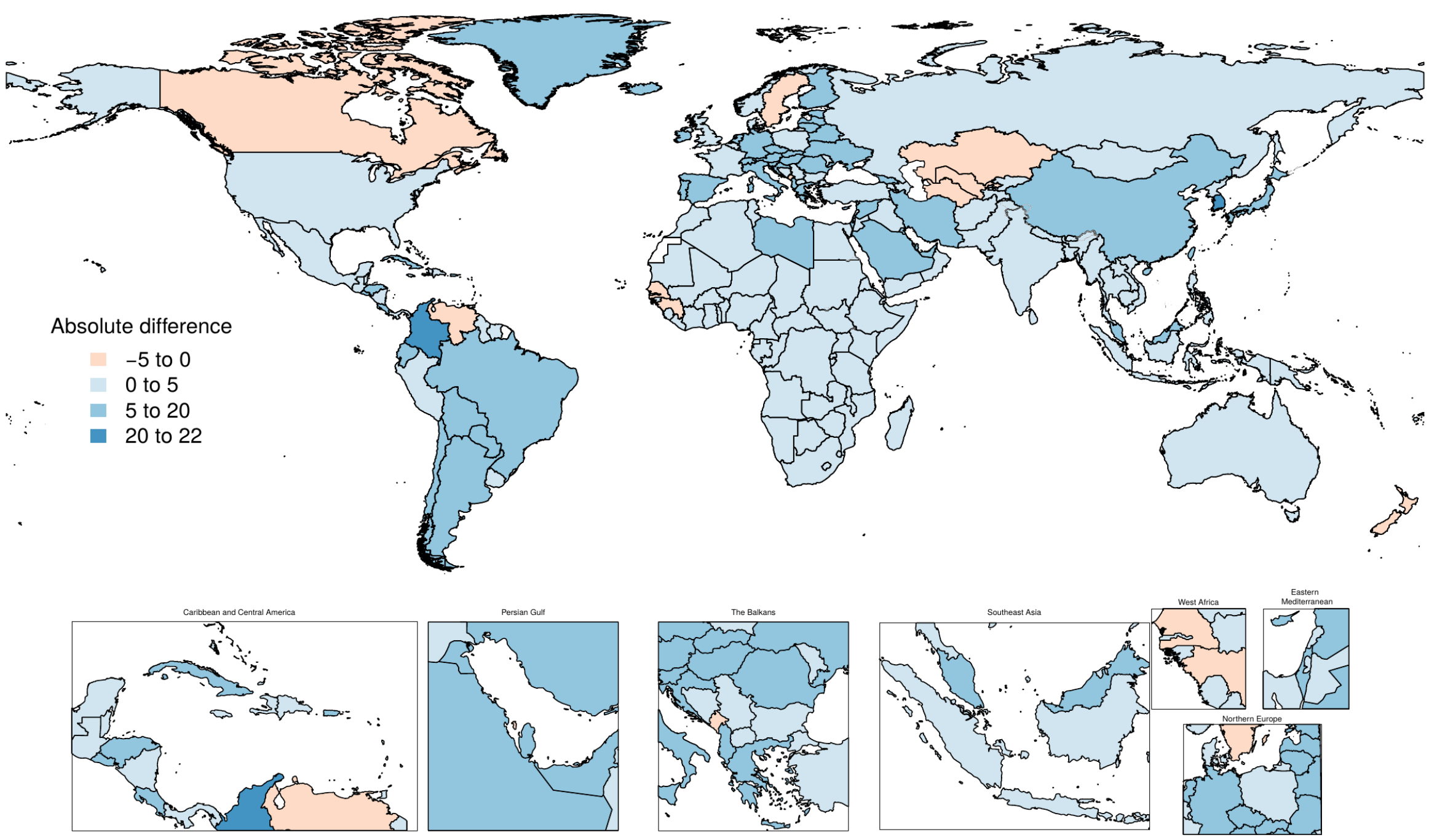

Appendix 2 Figure S10c: Absolute difference in densities of nursing personnel by country, 1990−2023

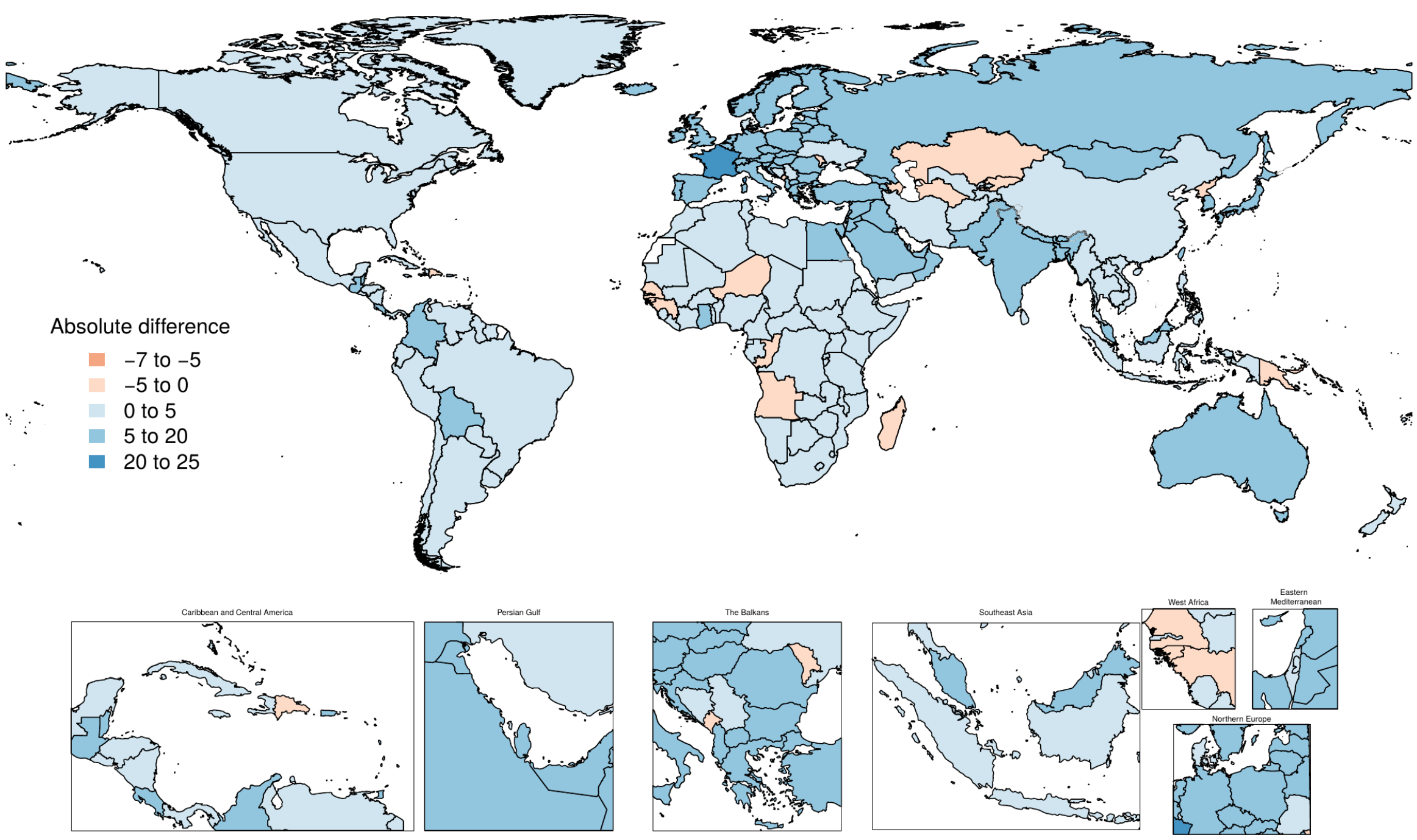

Appendix 2 Figure S10d: Absolute difference in densities of midwifery personnel by country, 1990−2023

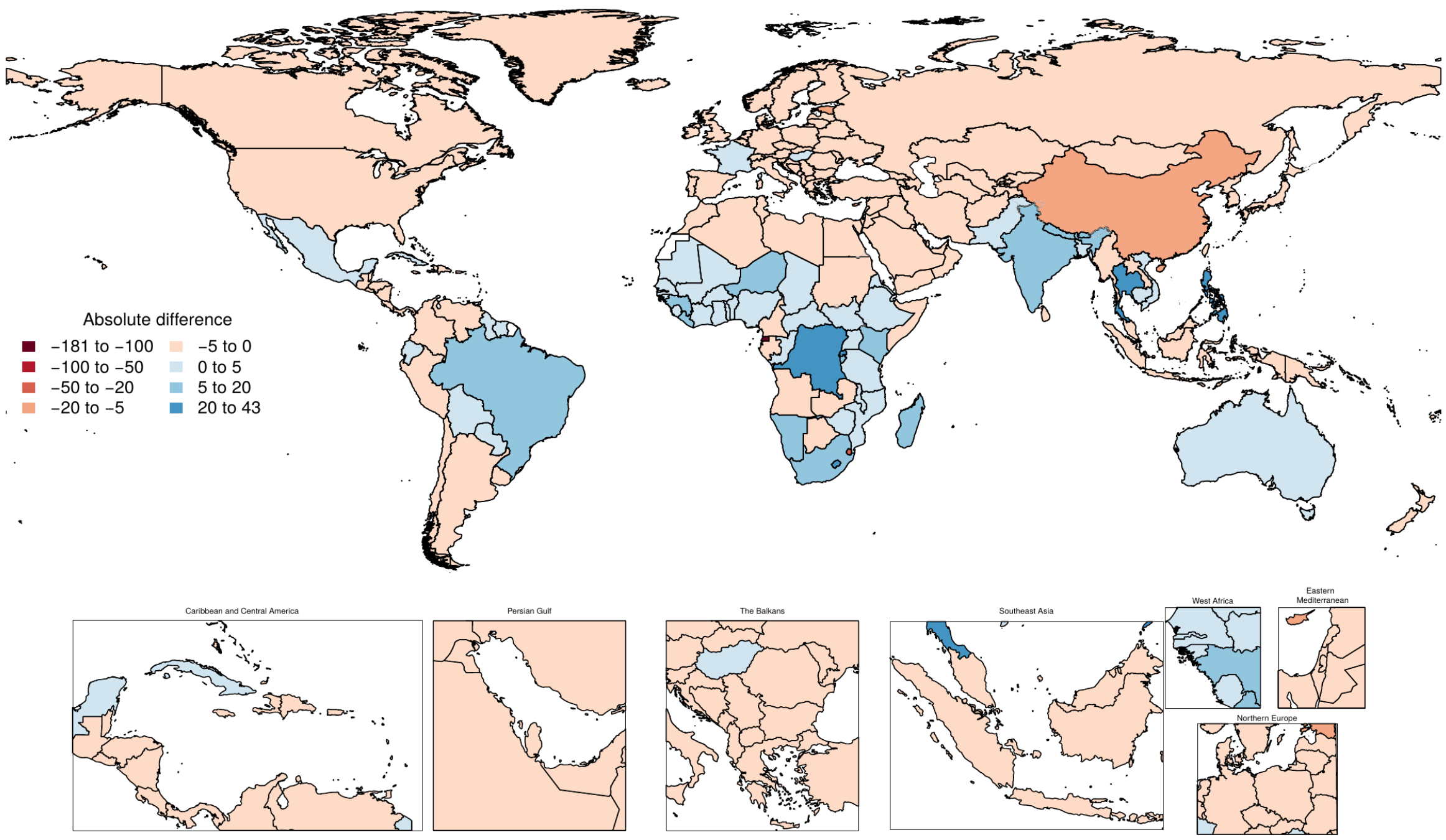

Appendix 2 Figure S10e: Absolute difference in densities of dentistry personnel by country, 1990−2023

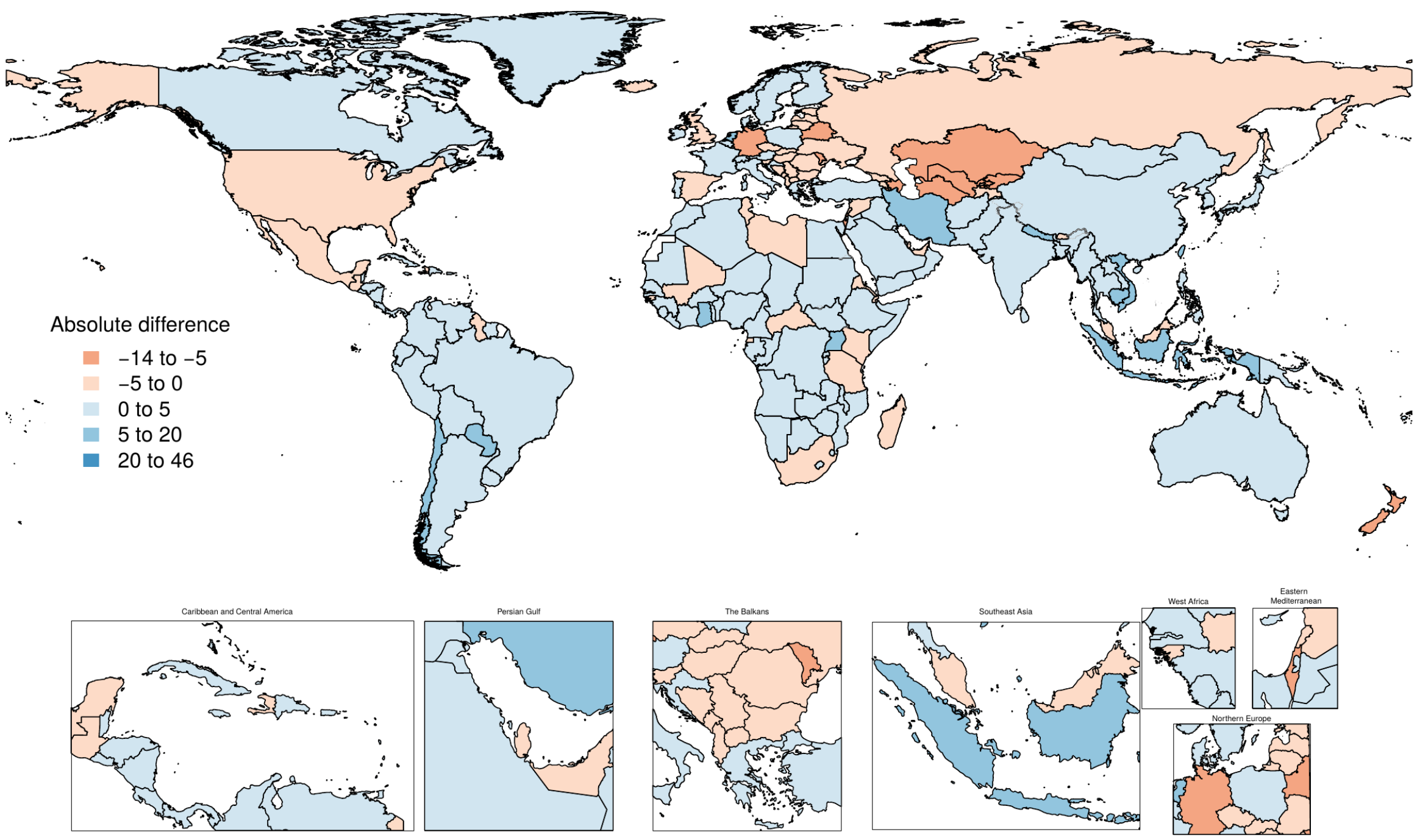

Appendix 2 Figure S10f: Absolute difference in densities of pharmaceutical personnel by country, 1990−2023

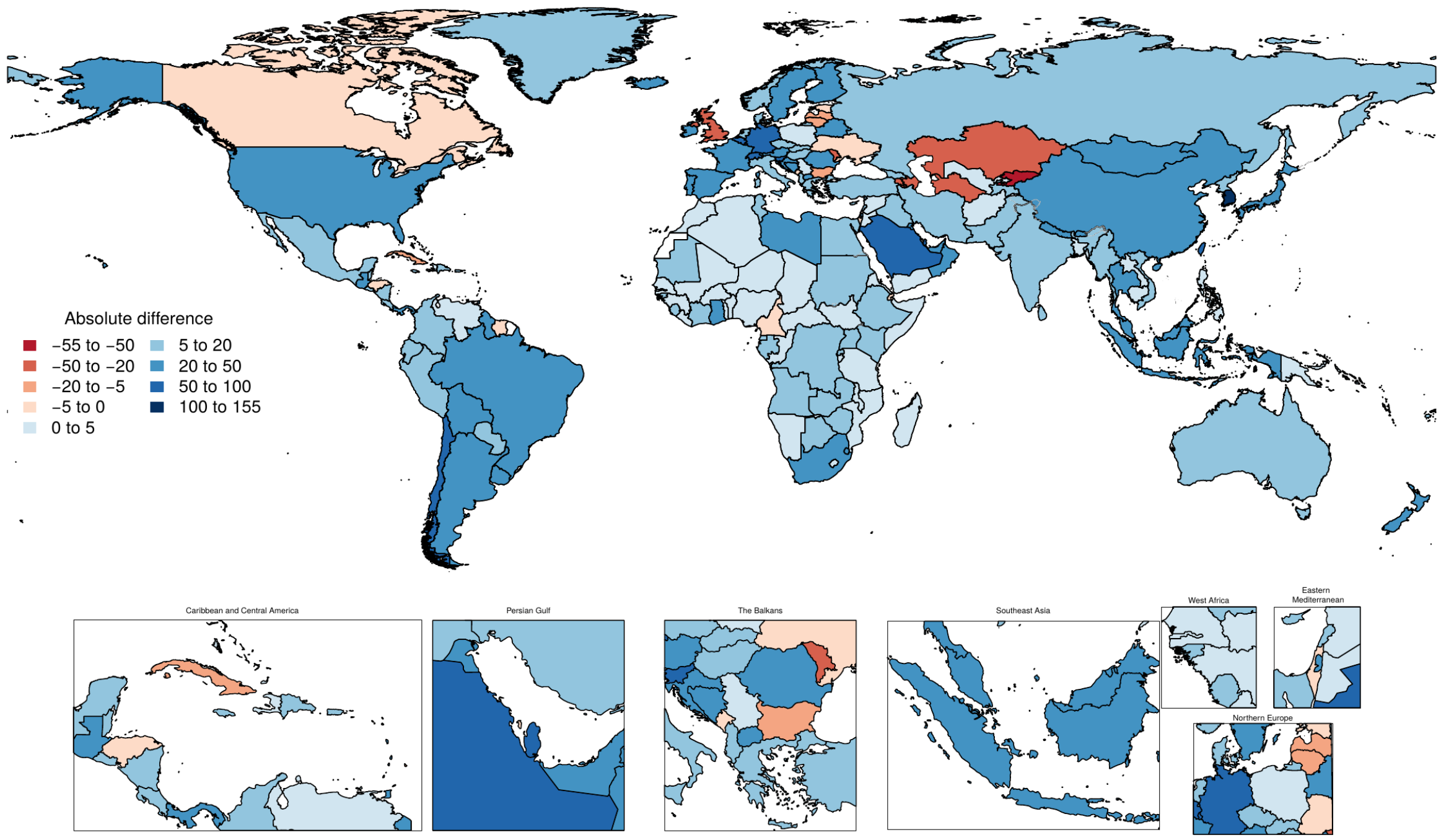

Appendix 2 Figure S10g: Absolute difference in densities of community health workers by country, 1990−2023

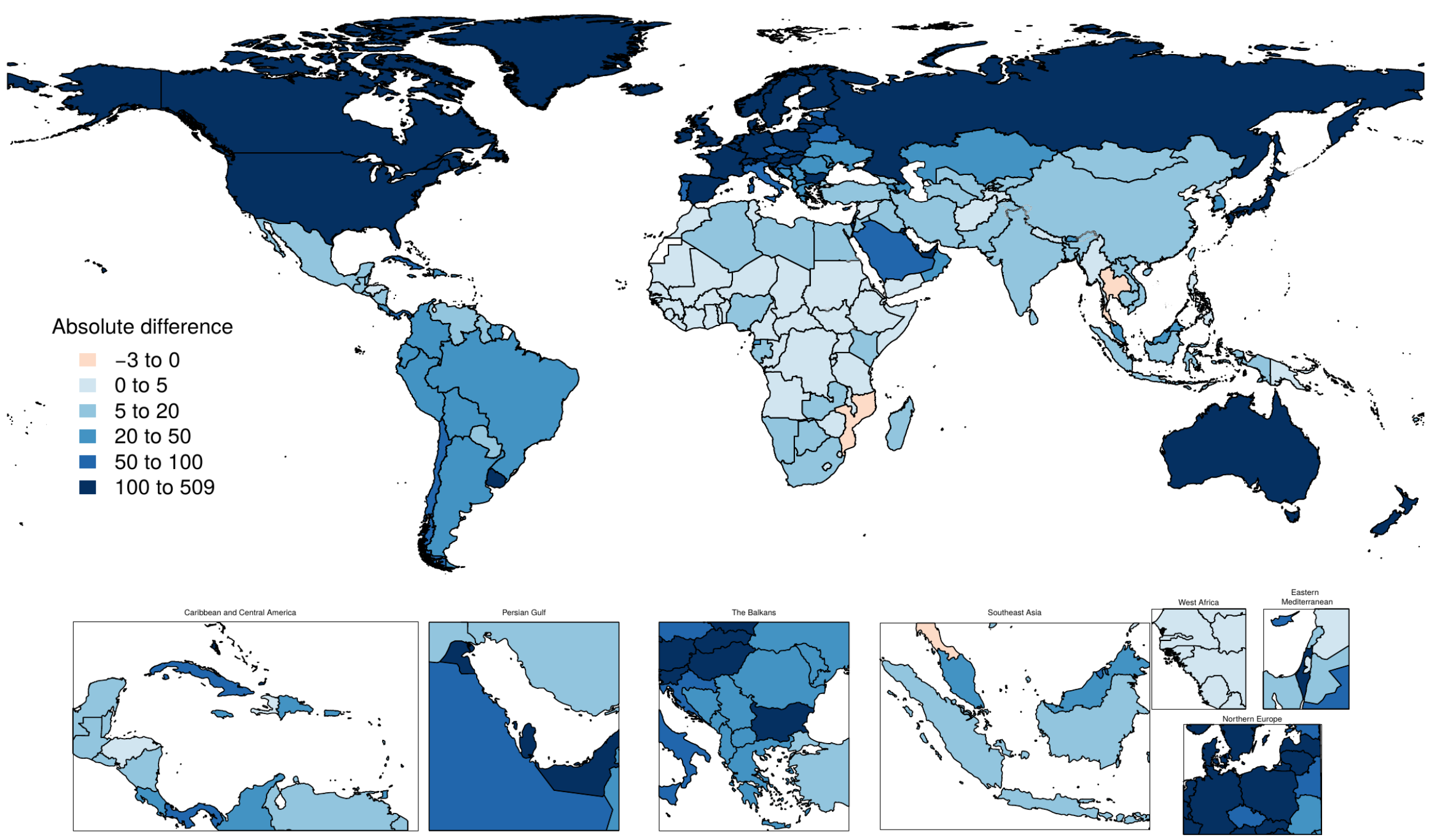

Appendix 2 Figure S10h: Absolute difference in densities of other health workers by country, 1990−2023

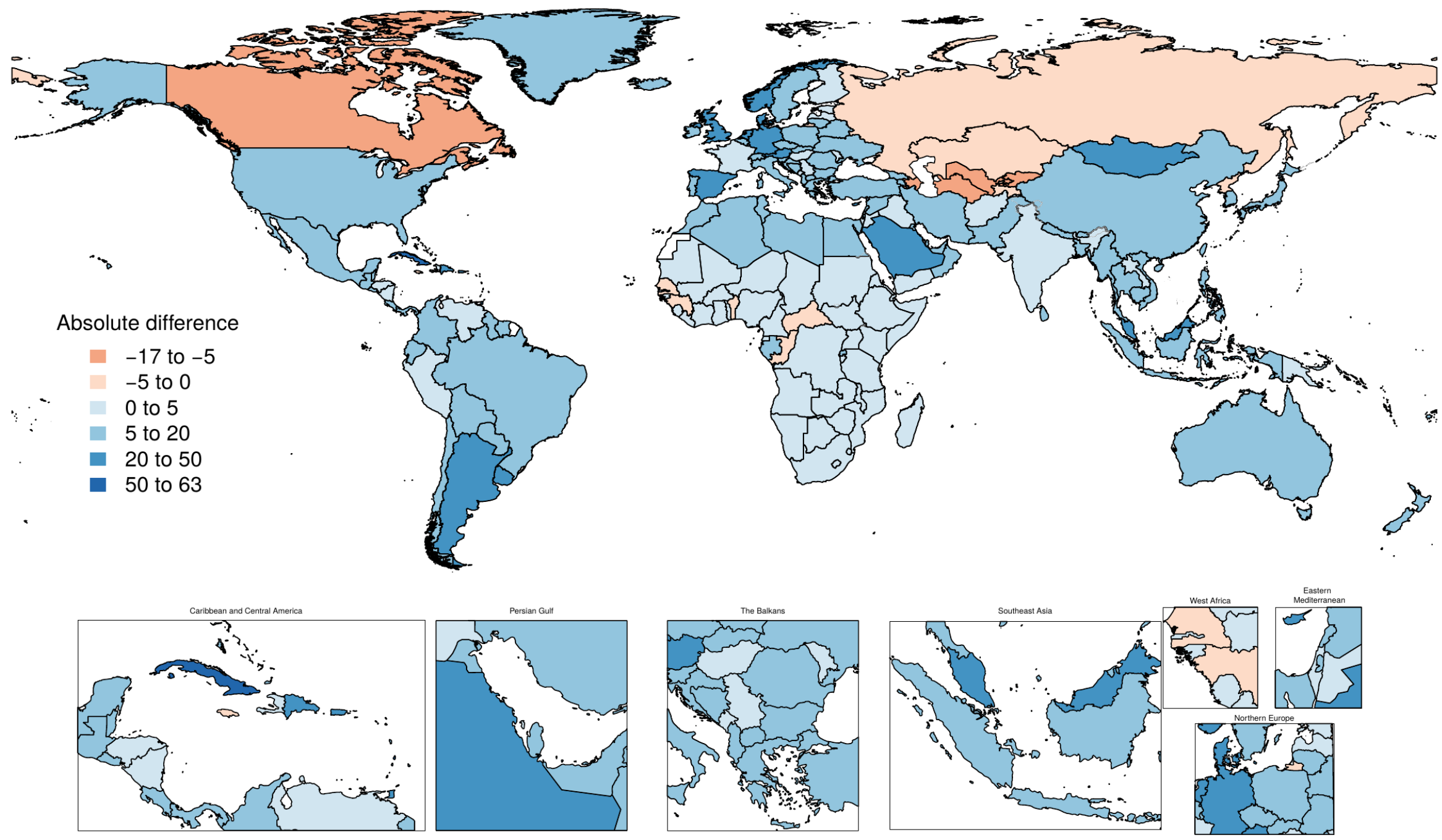

Appendix 2 Table S4: Density of health workers (95% UI) for all SDI quintiles, GBD super-regions, regions, and 204 countries and territories in 2023

Location	Cadre	Density per 10,000
**Global**	Audiologists, speech therapists, and counsellors	4.9 (4.1 to 5.7)
**Global**	Clinical officers and medical assistants	4.8 (4.3 to 5.4)
**Global**	Dieticians and nutritionists	1.8 (1.6 to 2.0)
**Global**	Environmental health workers	2.6 (2.1 to 3.1)
**Global**	Healthcare aides, paramedics, and ambulance workers	14.0 (12.7 to 15.7)
**Global**	Home-based personal care workers	15.3 (13.0 to 18.2)
**Global**	Medical imaging and therapeutic equipment technicians	3.0 (2.5 to 3.7)
**Global**	Medical laboratory technicians	5.3 (4.5 to 6.3)
**Global**	Optometrists and opticians	1.8 (1.5 to 2.1)
**Global**	Physiotherapists and prosthetic technicians	7.0 (6.5 to 7.6)
**Global**	Psychologists	4.2 (3.5 to 5.1)
**Global**	Traditional and complementary medicine practitioners	3.2 (2.3 to 4.4)
High SDI	Audiologists, speech therapists, and counsellors	11.1 (9.6 to 12.9)
High SDI	Clinical officers and medical assistants	13.4 (12.1 to 15.0)
High SDI	Dieticians and nutritionists	6.5 (5.7 to 7.7)
High SDI	Environmental health workers	6.2 (5.2 to 7.3)
High SDI	Healthcare aides, paramedics, and ambulance workers	55.1 (50.1 to 61.7)
High SDI	Home-based personal care workers	45.6 (41.5 to 50.8)
High SDI	Medical imaging and therapeutic equipment technicians	8.9 (7.8 to 10.5)
High SDI	Medical laboratory technicians	10.8 (8.6 to 13.7)
High SDI	Optometrists and opticians	5.5 (4.7 to 6.4)
High SDI	Physiotherapists and prosthetic technicians	27.1 (25.2 to 29.3)
High SDI	Psychologists	9.5 (8.6 to 10.9)
High SDI	Traditional and complementary medicine practitioners	4.4 (3.4 to 6.1)
High-middle SDI	Audiologists, speech therapists, and counsellors	7.6 (5.6 to 10.0)
High-middle SDI	Clinical officers and medical assistants	7.5 (5.9 to 9.7)
High-middle SDI	Dieticians and nutritionists	2.0 (1.5 to 2.8)

High-middle SDI	Environmental health workers	3.1 (2.3 to 4.2)
High-middle SDI	Healthcare aides, paramedics, and ambulance workers	25.5 (20.9 to 30.9)
High-middle SDI	Home-based personal care workers	8.1 (6.1 to 10.3)
High-middle SDI	Medical imaging and therapeutic equipment technicians	3.2 (2.2 to 4.4)
High-middle SDI	Medical laboratory technicians	8.9 (7.2 to 11.1)
High-middle SDI	Optometrists and opticians	1.6 (1.2 to 2.2)
High-middle SDI	Physiotherapists and prosthetic technicians	6.8 (5.2 to 8.8)
High-middle SDI	Psychologists	6.5 (4.9 to 8.4)
High-middle SDI	Traditional and complementary medicine practitioners	1.2 (0.8 to 1.7)
Middle SDI	Audiologists, speech therapists, and counsellors	3.5 (2.4 to 5.1)
Middle SDI	Clinical officers and medical assistants	3.4 (2.4 to 4.7)
Middle SDI	Dieticians and nutritionists	1.0 (0.9 to 1.2)
Middle SDI	Environmental health workers	3.2 (2.5 to 3.8)
Middle SDI	Healthcare aides, paramedics, and ambulance workers	4.0 (3.1 to 5.4)
Middle SDI	Home-based personal care workers	13.0 (9.6 to 17.1)
Middle SDI	Medical imaging and therapeutic equipment technicians	2.3 (1.5 to 3.3)
Middle SDI	Medical laboratory technicians	4.7 (3.4 to 6.6)
Middle SDI	Optometrists and opticians	1.3 (0.9 to 1.7)
Middle SDI	Physiotherapists and prosthetic technicians	3.0 (2.7 to 3.3)
Middle SDI	Psychologists	5.6 (4.4 to 7.2)
Middle SDI	Traditional and complementary medicine practitioners	5.2 (3.7 to 7.2)
Low-middle SDI	Audiologists, speech therapists, and counsellors	3.9 (3.0 to 5.0)
Low-middle SDI	Clinical officers and medical assistants	2.3 (1.7 to 2.9)
Low-middle SDI	Dieticians and nutritionists	0.3 (0.2 to 0.3)
Low-middle SDI	Environmental health workers	0.5 (0.4 to 0.7)
Low-middle SDI	Healthcare aides, paramedics, and ambulance workers	2.8 (2.4 to 3.3)
Low-middle SDI	Home-based personal care workers	6.3 (4.6 to 8.0)
Low-middle SDI	Medical imaging and therapeutic equipment technicians	1.1 (0.9 to 1.4)
Low-middle SDI	Medical laboratory technicians	3.7 (3.0 to 4.5)
Low-middle SDI	Optometrists and opticians	0.7 (0.6 to 0.9)

Low-middle SDI	Physiotherapists and prosthetic technicians	1.7 (1.5 to 2.0)
Low-middle SDI	Psychologists	0.6 (0.5 to 0.7)
Low-middle SDI	Traditional and complementary medicine practitioners	1.1 (0.8 to 1.6)
Low SDI	Audiologists, speech therapists, and counsellors	0.8 (0.6 to 1.2)
Low SDI	Clinical officers and medical assistants	1.1 (0.8 to 1.6)
Low SDI	Dieticians and nutritionists	0.2 (0.1 to 0.3)
Low SDI	Environmental health workers	0.6 (0.5 to 0.7)
Low SDI	Healthcare aides, paramedics, and ambulance workers	1.7 (1.2 to 2.4)
Low SDI	Home-based personal care workers	2.0 (1.5 to 2.7)
Low SDI	Medical imaging and therapeutic equipment technicians	0.4 (0.3 to 0.5)
Low SDI	Medical laboratory technicians	1.4 (1.0 to 2.1)
Low SDI	Optometrists and opticians	0.2 (0.2 to 0.3)
Low SDI	Physiotherapists and prosthetic technicians	0.8 (0.6 to 1.1)
Low SDI	Psychologists	0.3 (0.2 to 0.4)
Low SDI	Traditional and complementary medicine practitioners	1.8 (1.3 to 2.4)
**Central Europe, eastern Europe, and central Asia**	Audiologists, speech therapists, and counsellors	10.7 (8.4 to 13.8)
**Central Europe, eastern Europe, and central Asia**	Clinical officers and medical assistants	8.9 (7.4 to 11.0)
**Central Europe, eastern Europe, and central Asia**	Dieticians and nutritionists	3.2 (2.4 to 4.2)
**Central Europe, eastern Europe, and central Asia**	Environmental health workers	4.2 (3.2 to 5.3)
**Central Europe, eastern Europe, and central Asia**	Healthcare aides, paramedics, and ambulance workers	30.6 (26.1 to 36.4)
**Central Europe, eastern Europe, and central Asia**	Home-based personal care workers	9.3 (8.0 to 10.9)
**Central Europe, eastern Europe, and central Asia**	Medical imaging and therapeutic equipment technicians	4.5 (3.4 to 6.1)
**Central Europe, eastern Europe, and central Asia**	Medical laboratory technicians	9.3 (7.7 to 11.4)
**Central Europe, eastern Europe, and central Asia**	Optometrists and opticians	3.8 (3.0 to 4.8)
**Central Europe, eastern Europe, and central Asia**	Physiotherapists and prosthetic technicians	8.5 (6.8 to 10.8)
**Central Europe, eastern Europe, and central Asia**	Psychologists	8.6 (7.2 to 10.5)
**Central Europe, eastern Europe, and central Asia**	Traditional and complementary medicine practitioners	1.1 (0.7 to 1.6)
Central Asia	Audiologists, speech therapists, and counsellors	4.2 (2.8 to 5.8)
Central Asia	Clinical officers and medical assistants	1.1 (0.7 to 1.6)
Central Asia	Dieticians and nutritionists	0.7 (0.5 to 1.1)

Central Asia	Environmental health workers	1.0 (0.7 to 1.4)
Central Asia	Healthcare aides, paramedics, and ambulance workers	3.6 (2.4 to 5.4)
Central Asia	Home-based personal care workers	4.6 (3.3 to 6.1)
Central Asia	Medical imaging and therapeutic equipment technicians	0.8 (0.6 to 1.1)
Central Asia	Medical laboratory technicians	2.2 (1.4 to 3.4)
Central Asia	Optometrists and opticians	0.7 (0.5 to 1.0)
Central Asia	Physiotherapists and prosthetic technicians	2.0 (1.3 to 2.7)
Central Asia	Psychologists	1.6 (1.1 to 2.3)
Central Asia	Traditional and complementary medicine practitioners	1.2 (0.8 to 1.8)
Armenia	Audiologists, speech therapists, and counsellors	5.5 (3.5 to 8.4)
Armenia	Clinical officers and medical assistants	1.5 (0.9 to 2.3)
Armenia	Dieticians and nutritionists	0.7 (0.5 to 1.0)
Armenia	Environmental health workers	1.4 (0.9 to 1.9)
Armenia	Healthcare aides, paramedics, and ambulance workers	9.9 (6.3 to 14.6)
Armenia	Home-based personal care workers	10.4 (7.3 to 14.0)
Armenia	Medical imaging and therapeutic equipment technicians	5.2 (3.5 to 7.4)
Armenia	Medical laboratory technicians	9.2 (6.0 to 13.6)
Armenia	Optometrists and opticians	0.7 (0.5 to 0.9)
Armenia	Physiotherapists and prosthetic technicians	2.5 (1.7 to 3.5)
Armenia	Psychologists	1.3 (0.9 to 1.8)
Armenia	Traditional and complementary medicine practitioners	1.7 (1.1 to 2.5)
Azerbaijan	Audiologists, speech therapists, and counsellors	8.1 (5.6 to 11.1)
Azerbaijan	Clinical officers and medical assistants	2.5 (1.5 to 3.9)
Azerbaijan	Dieticians and nutritionists	1.2 (0.7 to 1.8)
Azerbaijan	Environmental health workers	1.8 (1.2 to 2.6)
Azerbaijan	Healthcare aides, paramedics, and ambulance workers	2.2 (1.4 to 3.4)
Azerbaijan	Home-based personal care workers	2.5 (1.7 to 3.5)
Azerbaijan	Medical imaging and therapeutic equipment technicians	0.1 (0.1 to 0.2)
Azerbaijan	Medical laboratory technicians	2.8 (1.7 to 4.3)
Azerbaijan	Optometrists and opticians	1.2 (0.8 to 1.7)

Azerbaijan	Physiotherapists and prosthetic technicians	3.7 (2.4 to 5.1)
Azerbaijan	Psychologists	0.6 (0.4 to 0.9)
Azerbaijan	Traditional and complementary medicine practitioners	1.1 (0.7 to 1.7)
Georgia	Audiologists, speech therapists, and counsellors	9.0 (6.3 to 12.2)
Georgia	Clinical officers and medical assistants	0.4 (0.3 to 0.6)
Georgia	Dieticians and nutritionists	0.5 (0.4 to 0.8)
Georgia	Environmental health workers	0.6 (0.4 to 0.8)
Georgia	Healthcare aides, paramedics, and ambulance workers	8.2 (5.7 to 11.1)
Georgia	Home-based personal care workers	10.3 (7.6 to 13.4)
Georgia	Medical imaging and therapeutic equipment technicians	1.1 (0.7 to 1.6)
Georgia	Medical laboratory technicians	0.6 (0.3 to 0.9)
Georgia	Optometrists and opticians	0.5 (0.4 to 0.7)
Georgia	Physiotherapists and prosthetic technicians	1.7 (1.2 to 2.4)
Georgia	Psychologists	5.7 (4.2 to 7.8)
Georgia	Traditional and complementary medicine practitioners	1.1 (0.8 to 1.7)
Kazakhstan	Audiologists, speech therapists, and counsellors	6.8 (4.4 to 9.8)
Kazakhstan	Clinical officers and medical assistants	1.4 (0.8 to 2.3)
Kazakhstan	Dieticians and nutritionists	1.4 (0.9 to 2.1)
Kazakhstan	Environmental health workers	1.5 (0.9 to 2.1)
Kazakhstan	Healthcare aides, paramedics, and ambulance workers	6.1 (4.1 to 9.4)
Kazakhstan	Home-based personal care workers	8.9 (6.4 to 11.7)
Kazakhstan	Medical imaging and therapeutic equipment technicians	1.4 (0.8 to 2.2)
Kazakhstan	Medical laboratory technicians	3.7 (2.3 to 5.7)
Kazakhstan	Optometrists and opticians	1.4 (1.0 to 2.1)
Kazakhstan	Physiotherapists and prosthetic technicians	3.5 (2.3 to 4.9)
Kazakhstan	Psychologists	3.4 (2.3 to 5.0)
Kazakhstan	Traditional and complementary medicine practitioners	1.6 (1.0 to 2.4)
Kyrgyzstan	Audiologists, speech therapists, and counsellors	2.1 (1.4 to 3.0)
Kyrgyzstan	Clinical officers and medical assistants	0.6 (0.4 to 1.0)
Kyrgyzstan	Dieticians and nutritionists	0.4 (0.2 to 0.6)

Kyrgyzstan	Environmental health workers	0.6 (0.4 to 0.9)
Kyrgyzstan	Healthcare aides, paramedics, and ambulance workers	2.0 (1.3 to 3.1)
Kyrgyzstan	Home-based personal care workers	2.6 (1.8 to 3.5)
Kyrgyzstan	Medical imaging and therapeutic equipment technicians	0.4 (0.2 to 0.6)
Kyrgyzstan	Medical laboratory technicians	1.3 (0.8 to 2.0)
Kyrgyzstan	Optometrists and opticians	0.4 (0.2 to 0.5)
Kyrgyzstan	Physiotherapists and prosthetic technicians	1.1 (0.7 to 1.5)
Kyrgyzstan	Psychologists	0.9 (0.6 to 1.3)
Kyrgyzstan	Traditional and complementary medicine practitioners	1.0 (0.6 to 1.5)
Mongolia	Audiologists, speech therapists, and counsellors	3.6 (2.5 to 5.1)
Mongolia	Clinical officers and medical assistants	2.0 (1.3 to 3.0)
Mongolia	Dieticians and nutritionists	1.4 (1.0 to 1.9)
Mongolia	Environmental health workers	1.4 (1.0 to 1.9)
Mongolia	Healthcare aides, paramedics, and ambulance workers	2.8 (1.8 to 4.0)
Mongolia	Home-based personal care workers	5.8 (4.2 to 7.5)
Mongolia	Medical imaging and therapeutic equipment technicians	0.6 (0.4 to 0.9)
Mongolia	Medical laboratory technicians	2.9 (2.1 to 3.9)
Mongolia	Optometrists and opticians	0.6 (0.4 to 0.8)
Mongolia	Physiotherapists and prosthetic technicians	2.1 (1.5 to 2.9)
Mongolia	Psychologists	1.8 (1.3 to 2.3)
Mongolia	Traditional and complementary medicine practitioners	3.3 (2.3 to 4.9)
Tajikistan	Audiologists, speech therapists, and counsellors	0.4 (0.3 to 0.6)
Tajikistan	Clinical officers and medical assistants	0.2 (0.1 to 0.3)
Tajikistan	Dieticians and nutritionists	0.1 (0.0 to 0.1)
Tajikistan	Environmental health workers	0.1 (0.1 to 0.2)
Tajikistan	Healthcare aides, paramedics, and ambulance workers	0.3 (0.2 to 0.5)
Tajikistan	Home-based personal care workers	0.6 (0.4 to 0.8)
Tajikistan	Medical imaging and therapeutic equipment technicians	0.1 (0.0 to 0.1)
Tajikistan	Medical laboratory technicians	0.5 (0.3 to 0.7)
Tajikistan	Optometrists and opticians	0.1 (0.0 to 0.1)

Tajikistan	Physiotherapists and prosthetic technicians	0.2 (0.1 to 0.3)
Tajikistan	Psychologists	0.1 (0.1 to 0.2)
Tajikistan	Traditional and complementary medicine practitioners	0.4 (0.3 to 0.6)
Turkmenistan	Audiologists, speech therapists, and counsellors	3.6 (2.3 to 5.2)
Turkmenistan	Clinical officers and medical assistants	0.9 (0.5 to 1.4)
Turkmenistan	Dieticians and nutritionists	0.7 (0.4 to 1.0)
Turkmenistan	Environmental health workers	0.9 (0.6 to 1.3)
Turkmenistan	Healthcare aides, paramedics, and ambulance workers	2.9 (1.9 to 4.5)
Turkmenistan	Home-based personal care workers	4.9 (3.6 to 6.5)
Turkmenistan	Medical imaging and therapeutic equipment technicians	0.7 (0.4 to 1.1)
Turkmenistan	Medical laboratory technicians	2.0 (1.2 to 3.1)
Turkmenistan	Optometrists and opticians	0.7 (0.5 to 1.0)
Turkmenistan	Physiotherapists and prosthetic technicians	1.9 (1.2 to 2.6)
Turkmenistan	Psychologists	1.4 (0.9 to 2.1)
Turkmenistan	Traditional and complementary medicine practitioners	1.5 (0.9 to 2.2)
Uzbekistan	Audiologists, speech therapists, and counsellors	2.6 (1.7 to 3.7)
Uzbekistan	Clinical officers and medical assistants	0.7 (0.4 to 1.2)
Uzbekistan	Dieticians and nutritionists	0.5 (0.3 to 0.7)
Uzbekistan	Environmental health workers	0.8 (0.5 to 1.2)
Uzbekistan	Healthcare aides, paramedics, and ambulance workers	3.0 (2.0 to 4.4)
Uzbekistan	Home-based personal care workers	3.0 (2.2 to 4.1)
Uzbekistan	Medical imaging and therapeutic equipment technicians	0.5 (0.3 to 0.7)
Uzbekistan	Medical laboratory technicians	1.5 (0.9 to 2.3)
Uzbekistan	Optometrists and opticians	0.4 (0.3 to 0.6)
Uzbekistan	Physiotherapists and prosthetic technicians	1.3 (0.8 to 1.8)
Uzbekistan	Psychologists	1.0 (0.7 to 1.5)
Uzbekistan	Traditional and complementary medicine practitioners	1.1 (0.7 to 1.6)
Central Europe	Audiologists, speech therapists, and counsellors	10.7 (9.0 to 12.9)
Central Europe	Clinical officers and medical assistants	6.1 (4.9 to 7.9)
Central Europe	Dieticians and nutritionists	4.8 (4.0 to 6.0)

Central Europe	Environmental health workers	4.4 (3.9 to 5.2)
Central Europe	Healthcare aides, paramedics, and ambulance workers	25.0 (21.1 to 31.1)
Central Europe	Home-based personal care workers	20.5 (18.5 to 23.0)
Central Europe	Medical imaging and therapeutic equipment technicians	7.1 (5.9 to 8.8)
Central Europe	Medical laboratory technicians	9.5 (7.7 to 12.1)
Central Europe	Optometrists and opticians	9.5 (7.8 to 11.5)
Central Europe	Physiotherapists and prosthetic technicians	14.2 (12.0 to 17.0)
Central Europe	Psychologists	15.6 (14.1 to 17.6)
Central Europe	Traditional and complementary medicine practitioners	1.8 (1.2 to 2.6)
Albania	Audiologists, speech therapists, and counsellors	1.9 (1.3 to 2.9)
Albania	Clinical officers and medical assistants	2.3 (1.5 to 3.3)
Albania	Dieticians and nutritionists	0.8 (0.5 to 1.2)
Albania	Environmental health workers	1.2 (0.8 to 1.6)
Albania	Healthcare aides, paramedics, and ambulance workers	7.0 (4.4 to 10.3)
Albania	Home-based personal care workers	5.5 (3.9 to 7.5)
Albania	Medical imaging and therapeutic equipment technicians	2.4 (1.6 to 3.6)
Albania	Medical laboratory technicians	4.3 (2.8 to 6.2)
Albania	Optometrists and opticians	1.0 (0.7 to 1.4)
Albania	Physiotherapists and prosthetic technicians	2.3 (1.6 to 3.3)
Albania	Psychologists	17.2 (12.0 to 23.2)
Albania	Traditional and complementary medicine practitioners	0.7 (0.5 to 1.0)
Bosnia and Herzegovina	Audiologists, speech therapists, and counsellors	2.4 (1.5 to 3.5)
Bosnia and Herzegovina	Clinical officers and medical assistants	1.7 (1.0 to 2.8)
Bosnia and Herzegovina	Dieticians and nutritionists	1.1 (0.7 to 1.6)
Bosnia and Herzegovina	Environmental health workers	1.3 (0.8 to 1.9)
Bosnia and Herzegovina	Healthcare aides, paramedics, and ambulance workers	3.8 (2.5 to 6.0)
Bosnia and Herzegovina	Home-based personal care workers	5.3 (3.8 to 7.2)
Bosnia and Herzegovina	Medical imaging and therapeutic equipment technicians	1.4 (0.8 to 2.2)
Bosnia and Herzegovina	Medical laboratory technicians	3.3 (2.0 to 5.0)
Bosnia and Herzegovina	Optometrists and opticians	1.6 (1.1 to 2.3)

Bosnia and Herzegovina	Physiotherapists and prosthetic technicians	2.6 (1.7 to 3.7)
Bosnia and Herzegovina	Psychologists	3.1 (2.1 to 4.6)
Bosnia and Herzegovina	Traditional and complementary medicine practitioners	0.5 (0.3 to 0.8)
Bulgaria	Audiologists, speech therapists, and counsellors	14.8 (9.5 to 22.1)
Bulgaria	Clinical officers and medical assistants	4.6 (2.8 to 7.0)
Bulgaria	Dieticians and nutritionists	2.9 (2.0 to 4.3)
Bulgaria	Environmental health workers	5.6 (3.7 to 7.9)
Bulgaria	Healthcare aides, paramedics, and ambulance workers	23.7 (15.4 to 35.5)
Bulgaria	Home-based personal care workers	17.2 (12.1 to 23.5)
Bulgaria	Medical imaging and therapeutic equipment technicians	10.5 (6.5 to 15.7)
Bulgaria	Medical laboratory technicians	14.1 (8.7 to 21.5)
Bulgaria	Optometrists and opticians	6.4 (4.4 to 9.0)
Bulgaria	Physiotherapists and prosthetic technicians	8.3 (5.8 to 11.8)
Bulgaria	Psychologists	18.0 (12.2 to 24.7)
Bulgaria	Traditional and complementary medicine practitioners	0.9 (0.6 to 1.4)
Croatia	Audiologists, speech therapists, and counsellors	8.5 (6.4 to 11.1)
Croatia	Clinical officers and medical assistants	12.0 (8.9 to 15.8)
Croatia	Dieticians and nutritionists	4.5 (3.4 to 5.9)
Croatia	Environmental health workers	8.8 (6.6 to 11.3)
Croatia	Healthcare aides, paramedics, and ambulance workers	7.8 (5.8 to 10.3)
Croatia	Home-based personal care workers	12.1 (9.8 to 15.2)
Croatia	Medical imaging and therapeutic equipment technicians	7.7 (5.5 to 10.5)
Croatia	Medical laboratory technicians	11.6 (8.3 to 15.7)
Croatia	Optometrists and opticians	4.4 (3.4 to 5.6)
Croatia	Physiotherapists and prosthetic technicians	9.6 (7.5 to 12.2)
Croatia	Psychologists	13.0 (9.6 to 16.4)
Croatia	Traditional and complementary medicine practitioners	1.2 (0.8 to 1.7)
Czechia	Audiologists, speech therapists, and counsellors	13.5 (10.3 to 17.7)
Czechia	Clinical officers and medical assistants	6.9 (5.1 to 8.9)
Czechia	Dieticians and nutritionists	8.3 (6.3 to 10.7)

Czechia	Environmental health workers	5.7 (4.4 to 7.4)
Czechia	Healthcare aides, paramedics, and ambulance workers	27.0 (19.1 to 36.7)
Czechia	Home-based personal care workers	24.7 (19.2 to 31.5)
Czechia	Medical imaging and therapeutic equipment technicians	8.5 (6.2 to 11.6)
Czechia	Medical laboratory technicians	18.2 (12.9 to 24.7)
Czechia	Optometrists and opticians	10.3 (8.3 to 13.1)
Czechia	Physiotherapists and prosthetic technicians	15.1 (11.9 to 19.3)
Czechia	Psychologists	8.1 (6.1 to 10.4)
Czechia	Traditional and complementary medicine practitioners	0.9 (0.6 to 1.4)
Hungary	Audiologists, speech therapists, and counsellors	10.2 (7.7 to 13.7)
Hungary	Clinical officers and medical assistants	16.0 (11.5 to 21.2)
Hungary	Dieticians and nutritionists	7.4 (5.8 to 9.7)
Hungary	Environmental health workers	4.5 (3.4 to 5.8)
Hungary	Healthcare aides, paramedics, and ambulance workers	31.6 (21.1 to 44.4)
Hungary	Home-based personal care workers	16.2 (12.3 to 20.7)
Hungary	Medical imaging and therapeutic equipment technicians	9.5 (6.9 to 13.1)
Hungary	Medical laboratory technicians	6.4 (4.6 to 8.6)
Hungary	Optometrists and opticians	11.8 (9.3 to 14.4)
Hungary	Physiotherapists and prosthetic technicians	12.5 (9.6 to 15.4)
Hungary	Psychologists	17.4 (12.9 to 22.1)
Hungary	Traditional and complementary medicine practitioners	1.0 (0.7 to 1.4)
Montenegro	Audiologists, speech therapists, and counsellors	5.7 (3.6 to 8.5)
Montenegro	Clinical officers and medical assistants	3.7 (2.2 to 6.0)
Montenegro	Dieticians and nutritionists	2.7 (1.7 to 4.0)
Montenegro	Environmental health workers	2.9 (1.9 to 4.3)
Montenegro	Healthcare aides, paramedics, and ambulance workers	11.5 (7.7 to 17.5)
Montenegro	Home-based personal care workers	12.7 (9.2 to 17.1)
Montenegro	Medical imaging and therapeutic equipment technicians	3.8 (2.2 to 5.7)
Montenegro	Medical laboratory technicians	6.5 (4.0 to 10.0)
Montenegro	Optometrists and opticians	3.2 (2.1 to 4.6)

Montenegro	Physiotherapists and prosthetic technicians	5.6 (3.7 to 7.9)
Montenegro	Psychologists	7.3 (4.9 to 10.8)
Montenegro	Traditional and complementary medicine practitioners	0.8 (0.5 to 1.3)
North Macedonia	Audiologists, speech therapists, and counsellors	3.7 (2.3 to 5.6)
North Macedonia	Clinical officers and medical assistants	2.4 (1.4 to 3.9)
North Macedonia	Dieticians and nutritionists	1.8 (1.2 to 2.7)
North Macedonia	Environmental health workers	1.8 (1.1 to 2.6)
North Macedonia	Healthcare aides, paramedics, and ambulance workers	6.2 (4.0 to 9.6)
North Macedonia	Home-based personal care workers	8.4 (5.9 to 11.3)
North Macedonia	Medical imaging and therapeutic equipment technicians	4.0 (2.4 to 6.0)
North Macedonia	Medical laboratory technicians	7.8 (5.2 to 11.5)
North Macedonia	Optometrists and opticians	2.6 (1.7 to 3.7)
North Macedonia	Physiotherapists and prosthetic technicians	4.0 (2.6 to 5.6)
North Macedonia	Psychologists	5.7 (3.8 to 8.2)
North Macedonia	Traditional and complementary medicine practitioners	0.6 (0.4 to 0.9)
Poland	Audiologists, speech therapists, and counsellors	13.8 (8.9 to 20.8)
Poland	Clinical officers and medical assistants	7.1 (4.3 to 10.7)
Poland	Dieticians and nutritionists	4.7 (3.2 to 6.9)
Poland	Environmental health workers	4.3 (2.8 to 6.0)
Poland	Healthcare aides, paramedics, and ambulance workers	38.5 (25.2 to 57.3)
Poland	Home-based personal care workers	24.6 (17.6 to 33.4)
Poland	Medical imaging and therapeutic equipment technicians	10.0 (6.1 to 14.9)
Poland	Medical laboratory technicians	10.0 (6.2 to 15.3)
Poland	Optometrists and opticians	14.2 (9.8 to 20.1)
Poland	Physiotherapists and prosthetic technicians	21.4 (15.3 to 30.0)
Poland	Psychologists	17.6 (11.8 to 24.6)
Poland	Traditional and complementary medicine practitioners	3.5 (2.4 to 5.2)
Romania	Audiologists, speech therapists, and counsellors	10.0 (6.8 to 14.2)
Romania	Clinical officers and medical assistants	0.7 (0.5 to 1.0)
Romania	Dieticians and nutritionists	4.5 (3.3 to 6.4)

Romania	Environmental health workers	5.0 (3.5 to 6.7)
Romania	Healthcare aides, paramedics, and ambulance workers	13.1 (9.1 to 17.3)
Romania	Home-based personal care workers	17.1 (13.3 to 21.6)
Romania	Medical imaging and therapeutic equipment technicians	2.7 (2.0 to 3.4)
Romania	Medical laboratory technicians	5.1 (3.5 to 7.1)
Romania	Optometrists and opticians	7.0 (5.3 to 9.4)
Romania	Physiotherapists and prosthetic technicians	11.8 (8.5 to 15.9)
Romania	Psychologists	23.5 (18.0 to 29.2)
Romania	Traditional and complementary medicine practitioners	0.5 (0.3 to 0.8)
Serbia	Audiologists, speech therapists, and counsellors	2.1 (1.3 to 3.2)
Serbia	Clinical officers and medical assistants	3.5 (2.2 to 5.4)
Serbia	Dieticians and nutritionists	1.5 (1.0 to 2.2)
Serbia	Environmental health workers	1.3 (0.8 to 1.9)
Serbia	Healthcare aides, paramedics, and ambulance workers	4.4 (2.9 to 6.8)
Serbia	Home-based personal care workers	4.5 (3.2 to 6.3)
Serbia	Medical imaging and therapeutic equipment technicians	2.4 (1.4 to 3.6)
Serbia	Medical laboratory technicians	3.7 (2.3 to 5.6)
Serbia	Optometrists and opticians	2.4 (1.6 to 3.4)
Serbia	Physiotherapists and prosthetic technicians	4.2 (2.8 to 5.9)
Serbia	Psychologists	4.1 (2.8 to 6.0)
Serbia	Traditional and complementary medicine practitioners	0.4 (0.3 to 0.6)
Slovakia	Audiologists, speech therapists, and counsellors	7.5 (5.5 to 10.2)
Slovakia	Clinical officers and medical assistants	6.8 (4.9 to 9.2)
Slovakia	Dieticians and nutritionists	7.7 (5.8 to 10.3)
Slovakia	Environmental health workers	4.3 (3.1 to 5.5)
Slovakia	Healthcare aides, paramedics, and ambulance workers	31.9 (23.7 to 41.5)
Slovakia	Home-based personal care workers	55.0 (44.9 to 68.9)
Slovakia	Medical imaging and therapeutic equipment technicians	3.8 (2.7 to 5.3)
Slovakia	Medical laboratory technicians	16.0 (11.1 to 22.0)
Slovakia	Optometrists and opticians	11.1 (8.6 to 14.3)

Slovakia	Physiotherapists and prosthetic technicians	14.7 (11.4 to 19.3)
Slovakia	Psychologists	13.4 (10.2 to 17.2)
Slovakia	Traditional and complementary medicine practitioners	0.5 (0.3 to 0.7)
Slovenia	Audiologists, speech therapists, and counsellors	9.9 (6.3 to 14.8)
Slovenia	Clinical officers and medical assistants	7.5 (4.6 to 11.6)
Slovenia	Dieticians and nutritionists	5.7 (3.9 to 8.1)
Slovenia	Environmental health workers	6.7 (4.4 to 9.5)
Slovenia	Healthcare aides, paramedics, and ambulance workers	26.7 (18.0 to 38.5)
Slovenia	Home-based personal care workers	29.8 (21.8 to 39.1)
Slovenia	Medical imaging and therapeutic equipment technicians	11.0 (6.9 to 16.2)
Slovenia	Medical laboratory technicians	16.0 (10.3 to 23.4)
Slovenia	Optometrists and opticians	7.4 (5.2 to 10.2)
Slovenia	Physiotherapists and prosthetic technicians	17.6 (12.6 to 24.2)
Slovenia	Psychologists	17.2 (11.7 to 23.8)
Slovenia	Traditional and complementary medicine practitioners	7.9 (5.3 to 11.8)
Eastern Europe	Audiologists, speech therapists, and counsellors	14.0 (10.4 to 18.3)
Eastern Europe	Clinical officers and medical assistants	14.3 (11.0 to 19.3)
Eastern Europe	Dieticians and nutritionists	3.4 (2.6 to 4.7)
Eastern Europe	Environmental health workers	5.6 (4.0 to 7.3)
Eastern Europe	Healthcare aides, paramedics, and ambulance workers	46.9 (37.9 to 58.0)
Eastern Europe	Home-based personal care workers	5.4 (4.2 to 6.9)
Eastern Europe	Medical imaging and therapeutic equipment technicians	5.0 (3.4 to 7.1)
Eastern Europe	Medical laboratory technicians	12.5 (9.7 to 16.3)
Eastern Europe	Optometrists and opticians	2.2 (1.7 to 2.9)
Eastern Europe	Physiotherapists and prosthetic technicians	8.6 (6.3 to 11.2)
Eastern Europe	Psychologists	8.1 (6.1 to 10.8)
Eastern Europe	Traditional and complementary medicine practitioners	0.6 (0.4 to 0.9)
Belarus	Audiologists, speech therapists, and counsellors	11.2 (7.6 to 16.4)
Belarus	Clinical officers and medical assistants	9.3 (5.9 to 13.4)
Belarus	Dieticians and nutritionists	3.2 (2.2 to 4.6)

Belarus	Environmental health workers	4.5 (3.0 to 6.3)
Belarus	Healthcare aides, paramedics, and ambulance workers	52.2 (33.2 to 76.6)
Belarus	Home-based personal care workers	10.4 (7.4 to 13.9)
Belarus	Medical imaging and therapeutic equipment technicians	3.0 (1.8 to 4.5)
Belarus	Medical laboratory technicians	10.5 (6.6 to 15.6)
Belarus	Optometrists and opticians	2.3 (1.6 to 3.1)
Belarus	Physiotherapists and prosthetic technicians	10.0 (7.0 to 14.0)
Belarus	Psychologists	9.9 (6.7 to 14.0)
Belarus	Traditional and complementary medicine practitioners	0.6 (0.4 to 0.9)
Estonia	Audiologists, speech therapists, and counsellors	13.7 (10.0 to 18.8)
Estonia	Clinical officers and medical assistants	4.4 (3.1 to 5.9)
Estonia	Dieticians and nutritionists	3.6 (2.6 to 4.8)
Estonia	Environmental health workers	4.7 (3.4 to 6.2)
Estonia	Healthcare aides, paramedics, and ambulance workers	48.3 (34.2 to 64.7)
Estonia	Home-based personal care workers	24.4 (19.4 to 30.8)
Estonia	Medical imaging and therapeutic equipment technicians	4.6 (3.3 to 6.3)
Estonia	Medical laboratory technicians	6.1 (4.2 to 8.6)
Estonia	Optometrists and opticians	2.3 (1.8 to 2.9)
Estonia	Physiotherapists and prosthetic technicians	13.8 (10.2 to 18.3)
Estonia	Psychologists	7.0 (5.1 to 9.3)
Estonia	Traditional and complementary medicine practitioners	0.8 (0.5 to 1.1)
Latvia	Audiologists, speech therapists, and counsellors	7.0 (5.2 to 9.1)
Latvia	Clinical officers and medical assistants	12.6 (9.4 to 16.6)
Latvia	Dieticians and nutritionists	2.2 (1.7 to 2.9)
Latvia	Environmental health workers	4.7 (3.5 to 6.1)
Latvia	Healthcare aides, paramedics, and ambulance workers	34.3 (25.5 to 46.5)
Latvia	Home-based personal care workers	13.3 (10.3 to 16.7)
Latvia	Medical imaging and therapeutic equipment technicians	2.8 (1.9 to 3.9)
Latvia	Medical laboratory technicians	6.3 (4.2 to 8.8)
Latvia	Optometrists and opticians	8.2 (6.5 to 10.5)

Latvia	Physiotherapists and prosthetic technicians	12.9 (10.1 to 16.5)
Latvia	Psychologists	9.6 (7.2 to 12.4)
Latvia	Traditional and complementary medicine practitioners	0.8 (0.6 to 1.2)
Lithuania	Audiologists, speech therapists, and counsellors	11.5 (7.7 to 15.7)
Lithuania	Clinical officers and medical assistants	6.7 (4.7 to 9.7)
Lithuania	Dieticians and nutritionists	10.8 (8.1 to 14.4)
Lithuania	Environmental health workers	12.9 (9.4 to 17.4)
Lithuania	Healthcare aides, paramedics, and ambulance workers	37.6 (27.7 to 49.5)
Lithuania	Home-based personal care workers	11.3 (9.1 to 14.2)
Lithuania	Medical imaging and therapeutic equipment technicians	5.6 (3.9 to 7.4)
Lithuania	Medical laboratory technicians	12.0 (8.7 to 15.8)
Lithuania	Optometrists and opticians	2.4 (1.8 to 3.3)
Lithuania	Physiotherapists and prosthetic technicians	17.3 (12.9 to 22.6)
Lithuania	Psychologists	9.5 (6.9 to 12.9)
Lithuania	Traditional and complementary medicine practitioners	0.6 (0.4 to 0.9)
Moldova	Audiologists, speech therapists, and counsellors	3.7 (2.1 to 5.8)
Moldova	Clinical officers and medical assistants	3.9 (2.3 to 6.4)
Moldova	Dieticians and nutritionists	1.3 (0.8 to 2.1)
Moldova	Environmental health workers	2.8 (1.8 to 4.2)
Moldova	Healthcare aides, paramedics, and ambulance workers	24.1 (18.4 to 31.3)
Moldova	Home-based personal care workers	2.9 (1.9 to 4.2)
Moldova	Medical imaging and therapeutic equipment technicians	1.3 (0.7 to 2.1)
Moldova	Medical laboratory technicians	4.0 (2.4 to 6.3)
Moldova	Optometrists and opticians	0.8 (0.5 to 1.3)
Moldova	Physiotherapists and prosthetic technicians	4.0 (2.5 to 5.9)
Moldova	Psychologists	3.6 (2.3 to 5.4)
Moldova	Traditional and complementary medicine practitioners	0.3 (0.2 to 0.5)
Russia	Audiologists, speech therapists, and counsellors	16.8 (12.3 to 22.2)
Russia	Clinical officers and medical assistants	15.9 (10.7 to 23.9)
Russia	Dieticians and nutritionists	3.7 (2.7 to 5.1)

Russia	Environmental health workers	6.0 (4.3 to 8.0)
Russia	Healthcare aides, paramedics, and ambulance workers	46.3 (32.5 to 62.5)
Russia	Home-based personal care workers	4.7 (3.5 to 6.0)
Russia	Medical imaging and therapeutic equipment technicians	5.9 (4.0 to 8.5)
Russia	Medical laboratory technicians	12.7 (8.3 to 18.5)
Russia	Optometrists and opticians	2.4 (1.8 to 3.2)
Russia	Physiotherapists and prosthetic technicians	7.5 (5.5 to 9.9)
Russia	Psychologists	8.7 (6.5 to 11.7)
Russia	Traditional and complementary medicine practitioners	0.6 (0.4 to 0.9)
Ukraine	Audiologists, speech therapists, and counsellors	5.2 (3.3 to 7.9)
Ukraine	Clinical officers and medical assistants	11.2 (6.8 to 16.8)
Ukraine	Dieticians and nutritionists	2.0 (1.4 to 3.0)
Ukraine	Environmental health workers	3.9 (2.5 to 5.4)
Ukraine	Healthcare aides, paramedics, and ambulance workers	50.8 (32.4 to 75.0)
Ukraine	Home-based personal care workers	5.6 (3.9 to 7.6)
Ukraine	Medical imaging and therapeutic equipment technicians	2.3 (1.4 to 3.5)
Ukraine	Medical laboratory technicians	13.5 (8.4 to 20.4)
Ukraine	Optometrists and opticians	1.3 (0.9 to 1.8)
Ukraine	Physiotherapists and prosthetic technicians	11.4 (7.9 to 16.2)
Ukraine	Psychologists	5.6 (3.7 to 7.9)
Ukraine	Traditional and complementary medicine practitioners	0.6 (0.4 to 0.9)
**High income**	Audiologists, speech therapists, and counsellors	13.8 (12.2 to 15.9)
**High income**	Clinical officers and medical assistants	17.6 (15.6 to 19.9)
**High income**	Dieticians and nutritionists	7.6 (6.8 to 8.7)
**High income**	Environmental health workers	7.2 (6.3 to 8.3)
**High income**	Healthcare aides, paramedics, and ambulance workers	69.9 (64.6 to 76.2)
**High income**	Home-based personal care workers	59.5 (54.4 to 65.4)
**High income**	Medical imaging and therapeutic equipment technicians	10.6 (9.6 to 11.9)
**High income**	Medical laboratory technicians	11.8 (9.6 to 14.5)
**High income**	Optometrists and opticians	6.0 (5.3 to 6.9)

**High income**	Physiotherapists and prosthetic technicians	35.8 (33.3 to 38.4)
**High income**	Psychologists	9.5 (8.9 to 10.4)
**High income**	Traditional and complementary medicine practitioners	5.0 (3.9 to 6.7)
Australasia	Audiologists, speech therapists, and counsellors	23.0 (15.3 to 33.4)
Australasia	Clinical officers and medical assistants	8.4 (5.2 to 12.7)
Australasia	Dieticians and nutritionists	6.1 (4.3 to 8.6)
Australasia	Environmental health workers	9.9 (6.6 to 13.8)
Australasia	Healthcare aides, paramedics, and ambulance workers	76.0 (51.3 to 106.2)
Australasia	Home-based personal care workers	71.7 (52.4 to 94.8)
Australasia	Medical imaging and therapeutic equipment technicians	18.1 (11.5 to 26.5)
Australasia	Medical laboratory technicians	19.8 (12.7 to 29.2)
Australasia	Optometrists and opticians	6.4 (4.5 to 8.6)
Australasia	Physiotherapists and prosthetic technicians	30.1 (21.7 to 41.3)
Australasia	Psychologists	17.7 (12.1 to 24.6)
Australasia	Traditional and complementary medicine practitioners	4.6 (3.1 to 6.9)
Australia	Audiologists, speech therapists, and counsellors	21.3 (14.1 to 30.9)
Australia	Clinical officers and medical assistants	6.8 (4.2 to 10.2)
Australia	Dieticians and nutritionists	5.7 (4.0 to 8.1)
Australia	Environmental health workers	9.5 (6.3 to 13.4)
Australia	Healthcare aides, paramedics, and ambulance workers	76.0 (51.5 to 105.8)
Australia	Home-based personal care workers	75.1 (54.5 to 99.1)
Australia	Medical imaging and therapeutic equipment technicians	19.7 (12.6 to 28.9)
Australia	Medical laboratory technicians	20.4 (13.1 to 30.1)
Australia	Optometrists and opticians	5.7 (4.0 to 7.6)
Australia	Physiotherapists and prosthetic technicians	29.7 (20.9 to 41.2)
Australia	Psychologists	18.9 (12.8 to 26.4)
Australia	Traditional and complementary medicine practitioners	3.6 (2.4 to 5.4)
New Zealand	Audiologists, speech therapists, and counsellors	31.4 (20.7 to 45.6)
New Zealand	Clinical officers and medical assistants	16.5 (10.3 to 24.5)
New Zealand	Dieticians and nutritionists	8.0 (5.6 to 11.6)

New Zealand	Environmental health workers	11.6 (7.8 to 15.9)
New Zealand	Healthcare aides, paramedics, and ambulance workers	75.7 (50.1 to 108.3)
New Zealand	Home-based personal care workers	55.3 (40.7 to 72.5)
New Zealand	Medical imaging and therapeutic equipment technicians	9.8 (6.1 to 14.7)
New Zealand	Medical laboratory technicians	16.6 (10.6 to 24.7)
New Zealand	Optometrists and opticians	10.0 (7.0 to 14.0)
New Zealand	Physiotherapists and prosthetic technicians	32.2 (23.1 to 44.2)
New Zealand	Psychologists	11.9 (8.1 to 16.4)
New Zealand	Traditional and complementary medicine practitioners	9.4 (6.2 to 14.1)
High-income Asia Pacific	Audiologists, speech therapists, and counsellors	13.1 (9.1 to 18.4)
High-income Asia Pacific	Clinical officers and medical assistants	6.4 (4.4 to 9.0)
High-income Asia Pacific	Dieticians and nutritionists	18.0 (13.3 to 24.6)
High-income Asia Pacific	Environmental health workers	3.5 (2.4 to 4.8)
High-income Asia Pacific	Healthcare aides, paramedics, and ambulance workers	77.2 (53.7 to 107.0)
High-income Asia Pacific	Home-based personal care workers	40.0 (29.4 to 52.4)
High-income Asia Pacific	Medical imaging and therapeutic equipment technicians	9.4 (5.9 to 14.0)
High-income Asia Pacific	Medical laboratory technicians	12.5 (8.2 to 18.4)
High-income Asia Pacific	Optometrists and opticians	10.2 (7.5 to 13.2)
High-income Asia Pacific	Physiotherapists and prosthetic technicians	24.8 (17.5 to 33.7)
High-income Asia Pacific	Psychologists	6.3 (4.2 to 8.9)
High-income Asia Pacific	Traditional and complementary medicine practitioners	8.2 (5.6 to 12.2)
Brunei	Audiologists, speech therapists, and counsellors	7.7 (4.7 to 11.6)
Brunei	Clinical officers and medical assistants	4.3 (2.5 to 7.2)
Brunei	Dieticians and nutritionists	8.5 (5.3 to 13.0)
Brunei	Environmental health workers	1.9 (1.2 to 2.9)
Brunei	Healthcare aides, paramedics, and ambulance workers	43.6 (31.8 to 61.5)
Brunei	Home-based personal care workers	19.9 (13.7 to 27.8)
Brunei	Medical imaging and therapeutic equipment technicians	6.3 (3.6 to 9.9)
Brunei	Medical laboratory technicians	8.0 (4.8 to 12.3)
Brunei	Optometrists and opticians	4.1 (2.7 to 6.1)

Brunei	Physiotherapists and prosthetic technicians	10.0 (6.6 to 14.5)
Brunei	Psychologists	7.2 (4.6 to 10.5)
Brunei	Traditional and complementary medicine practitioners	3.7 (2.3 to 5.7)
Japan	Audiologists, speech therapists, and counsellors	13.2 (9.2 to 18.1)
Japan	Clinical officers and medical assistants	7.2 (5.0 to 10.1)
Japan	Dieticians and nutritionists	19.5 (14.2 to 27.1)
Japan	Environmental health workers	2.0 (1.3 to 2.6)
Japan	Healthcare aides, paramedics, and ambulance workers	91.4 (62.7 to 127.7)
Japan	Home-based personal care workers	46.2 (34.1 to 60.2)
Japan	Medical imaging and therapeutic equipment technicians	9.2 (5.7 to 13.4)
Japan	Medical laboratory technicians	13.6 (9.0 to 19.9)
Japan	Optometrists and opticians	9.0 (6.7 to 11.6)
Japan	Physiotherapists and prosthetic technicians	27.2 (19.3 to 36.7)
Japan	Psychologists	5.2 (3.5 to 7.3)
Japan	Traditional and complementary medicine practitioners	10.4 (7.1 to 15.5)
South Korea	Audiologists, speech therapists, and counsellors	12.6 (8.3 to 18.9)
South Korea	Clinical officers and medical assistants	4.3 (2.6 to 6.4)
South Korea	Dieticians and nutritionists	14.3 (9.8 to 20.6)
South Korea	Environmental health workers	7.1 (4.6 to 9.9)
South Korea	Healthcare aides, paramedics, and ambulance workers	38.9 (24.9 to 56.0)
South Korea	Home-based personal care workers	25.1 (17.8 to 33.7)
South Korea	Medical imaging and therapeutic equipment technicians	9.8 (6.0 to 14.7)
South Korea	Medical laboratory technicians	9.3 (5.8 to 14.0)
South Korea	Optometrists and opticians	13.1 (9.3 to 18.0)
South Korea	Physiotherapists and prosthetic technicians	19.0 (13.3 to 26.7)
South Korea	Psychologists	8.1 (5.3 to 11.4)
South Korea	Traditional and complementary medicine practitioners	3.2 (2.1 to 4.8)
Singapore	Audiologists, speech therapists, and counsellors	14.6 (8.7 to 22.1)
Singapore	Clinical officers and medical assistants	7.2 (4.2 to 12.0)
Singapore	Dieticians and nutritionists	19.1 (12.0 to 29.2)

Singapore	Environmental health workers	4.1 (2.7 to 6.1)
Singapore	Healthcare aides, paramedics, and ambulance workers	116.1 (87.0 to 159.4)
Singapore	Home-based personal care workers	42.7 (29.5 to 60.2)
Singapore	Medical imaging and therapeutic equipment technicians	12.7 (7.3 to 19.9)
Singapore	Medical laboratory technicians	16.6 (10.0 to 25.6)
Singapore	Optometrists and opticians	10.1 (6.7 to 15.1)
Singapore	Physiotherapists and prosthetic technicians	26.2 (16.9 to 38.0)
Singapore	Psychologists	14.4 (9.2 to 21.3)
Singapore	Traditional and complementary medicine practitioners	6.4 (3.9 to 9.8)
High-income North America	Audiologists, speech therapists, and counsellors	19.5 (17.0 to 22.6)
High-income North America	Clinical officers and medical assistants	33.8 (28.7 to 39.5)
High-income North America	Dieticians and nutritionists	4.3 (3.7 to 5.0)
High-income North America	Environmental health workers	6.9 (4.4 to 9.7)
High-income North America	Healthcare aides, paramedics, and ambulance workers	58.7 (44.4 to 77.3)
High-income North America	Home-based personal care workers	63.0 (57.8 to 68.5)
High-income North America	Medical imaging and therapeutic equipment technicians	14.4 (12.9 to 16.0)
High-income North America	Medical laboratory technicians	12.9 (9.6 to 16.8)
High-income North America	Optometrists and opticians	3.7 (2.9 to 4.5)
High-income North America	Physiotherapists and prosthetic technicians	53.2 (47.8 to 60.2)
High-income North America	Psychologists	5.8 (4.6 to 7.2)
High-income North America	Traditional and complementary medicine practitioners	1.9 (1.3 to 2.8)
Canada	Audiologists, speech therapists, and counsellors	12.2 (8.0 to 17.9)
Canada	Clinical officers and medical assistants	29.7 (18.7 to 44.0)
Canada	Dieticians and nutritionists	7.6 (5.3 to 10.9)
Canada	Environmental health workers	3.1 (2.1 to 4.3)
Canada	Healthcare aides, paramedics, and ambulance workers	34.2 (21.9 to 51.4)
Canada	Home-based personal care workers	26.9 (19.0 to 36.6)
Canada	Medical imaging and therapeutic equipment technicians	23.5 (14.7 to 34.7)
Canada	Medical laboratory technicians	17.7 (10.9 to 26.8)
Canada	Optometrists and opticians	6.0 (4.2 to 8.2)

Canada	Physiotherapists and prosthetic technicians	34.1 (24.1 to 46.8)
Canada	Psychologists	6.1 (4.1 to 8.4)
Canada	Traditional and complementary medicine practitioners	13.5 (8.9 to 20.0)
Greenland	Audiologists, speech therapists, and counsellors	11.9 (7.2 to 18.0)
Greenland	Clinical officers and medical assistants	32.1 (20.0 to 50.6)
Greenland	Dieticians and nutritionists	5.5 (3.4 to 8.3)
Greenland	Environmental health workers	6.5 (4.2 to 9.7)
Greenland	Healthcare aides, paramedics, and ambulance workers	43.6 (30.2 to 65.6)
Greenland	Home-based personal care workers	49.1 (35.1 to 66.1)
Greenland	Medical imaging and therapeutic equipment technicians	15.7 (9.1 to 23.8)
Greenland	Medical laboratory technicians	17.6 (10.8 to 28.0)
Greenland	Optometrists and opticians	4.9 (3.3 to 7.1)
Greenland	Physiotherapists and prosthetic technicians	27.5 (18.1 to 38.6)
Greenland	Psychologists	9.8 (6.5 to 14.8)
Greenland	Traditional and complementary medicine practitioners	2.4 (1.5 to 3.7)
USA	Audiologists, speech therapists, and counsellors	20.3 (17.7 to 23.9)
USA	Clinical officers and medical assistants	34.3 (28.1 to 41.0)
USA	Dieticians and nutritionists	3.9 (3.3 to 4.8)
USA	Environmental health workers	7.3 (4.7 to 10.3)
USA	Healthcare aides, paramedics, and ambulance workers	61.5 (44.4 to 82.7)
USA	Home-based personal care workers	67.1 (61.4 to 73.9)
USA	Medical imaging and therapeutic equipment technicians	13.3 (11.2 to 15.8)
USA	Medical laboratory technicians	12.3 (9.2 to 15.9)
USA	Optometrists and opticians	3.4 (2.7 to 4.2)
USA	Physiotherapists and prosthetic technicians	55.5 (48.9 to 64.0)
USA	Psychologists	5.8 (4.3 to 7.5)
USA	Traditional and complementary medicine practitioners	0.6 (0.4 to 0.8)
Southern Latin America	Audiologists, speech therapists, and counsellors	10.5 (6.9 to 15.6)
Southern Latin America	Clinical officers and medical assistants	7.9 (6.6 to 9.4)
Southern Latin America	Dieticians and nutritionists	4.2 (3.5 to 5.3)

Southern Latin America	Environmental health workers	3.6 (2.4 to 5.1)
Southern Latin America	Healthcare aides, paramedics, and ambulance workers	43.8 (31.3 to 61.0)
Southern Latin America	Home-based personal care workers	52.1 (40.2 to 66.9)
Southern Latin America	Medical imaging and therapeutic equipment technicians	9.1 (5.8 to 13.5)
Southern Latin America	Medical laboratory technicians	6.0 (4.1 to 8.7)
Southern Latin America	Optometrists and opticians	6.5 (4.6 to 9.1)
Southern Latin America	Physiotherapists and prosthetic technicians	22.9 (18.1 to 29.7)
Southern Latin America	Psychologists	19.8 (16.7 to 23.8)
Southern Latin America	Traditional and complementary medicine practitioners	3.7 (2.5 to 5.5)
Argentina	Audiologists, speech therapists, and counsellors	13.1 (8.4 to 19.9)
Argentina	Clinical officers and medical assistants	5.6 (3.4 to 8.6)
Argentina	Dieticians and nutritionists	3.9 (2.7 to 5.8)
Argentina	Environmental health workers	4.7 (3.1 to 6.6)
Argentina	Healthcare aides, paramedics, and ambulance workers	50.7 (32.3 to 74.6)
Argentina	Home-based personal care workers	67.0 (47.3 to 90.4)
Argentina	Medical imaging and therapeutic equipment technicians	12.3 (7.5 to 18.7)
Argentina	Medical laboratory technicians	6.2 (3.8 to 9.5)
Argentina	Optometrists and opticians	9.1 (6.3 to 12.8)
Argentina	Physiotherapists and prosthetic technicians	26.2 (18.3 to 37.4)
Argentina	Psychologists	19.0 (12.7 to 26.8)
Argentina	Traditional and complementary medicine practitioners	4.7 (3.1 to 7.1)
Chile	Audiologists, speech therapists, and counsellors	5.8 (4.2 to 8.1)
Chile	Clinical officers and medical assistants	14.2 (10.2 to 20.0)
Chile	Dieticians and nutritionists	4.5 (3.4 to 5.8)
Chile	Environmental health workers	1.4 (1.0 to 1.9)
Chile	Healthcare aides, paramedics, and ambulance workers	25.0 (19.0 to 33.1)
Chile	Home-based personal care workers	23.3 (19.2 to 28.6)
Chile	Medical imaging and therapeutic equipment technicians	1.8 (1.2 to 2.5)
Chile	Medical laboratory technicians	4.7 (3.2 to 6.6)
Chile	Optometrists and opticians	0.7 (0.5 to 1.0)

Chile	Physiotherapists and prosthetic technicians	16.1 (13.0 to 19.9)
Chile	Psychologists	19.0 (14.8 to 23.5)
Chile	Traditional and complementary medicine practitioners	1.6 (1.2 to 2.2)
Uruguay	Audiologists, speech therapists, and counsellors	1.7 (1.1 to 2.3)
Uruguay	Clinical officers and medical assistants	5.6 (4.0 to 7.6)
Uruguay	Dieticians and nutritionists	6.6 (5.0 to 8.6)
Uruguay	Environmental health workers	0.4 (0.3 to 0.6)
Uruguay	Healthcare aides, paramedics, and ambulance workers	51.1 (39.9 to 64.5)
Uruguay	Home-based personal care workers	5.6 (4.0 to 7.6)
Uruguay	Medical imaging and therapeutic equipment technicians	5.3 (3.8 to 7.3)
Uruguay	Medical laboratory technicians	10.7 (7.8 to 14.3)
Uruguay	Optometrists and opticians	2.8 (2.1 to 3.7)
Uruguay	Physiotherapists and prosthetic technicians	14.0 (11.2 to 17.8)
Uruguay	Psychologists	33.3 (26.0 to 41.1)
Uruguay	Traditional and complementary medicine practitioners	1.0 (0.7 to 1.5)
Western Europe	Audiologists, speech therapists, and counsellors	8.3 (7.1 to 9.7)
Western Europe	Clinical officers and medical assistants	9.4 (7.8 to 11.3)
Western Europe	Dieticians and nutritionists	6.6 (5.9 to 7.5)
Western Europe	Environmental health workers	9.7 (8.4 to 11.3)
Western Europe	Healthcare aides, paramedics, and ambulance workers	82.1 (75.3 to 92.9)
Western Europe	Home-based personal care workers	65.9 (57.8 to 74.3)
Western Europe	Medical imaging and therapeutic equipment technicians	6.9 (6.2 to 8.0)
Western Europe	Medical laboratory technicians	10.8 (8.8 to 13.4)
Western Europe	Optometrists and opticians	6.1 (5.3 to 7.2)
Western Europe	Physiotherapists and prosthetic technicians	26.7 (23.3 to 30.3)
Western Europe	Psychologists	12.2 (11.5 to 13.4)
Western Europe	Traditional and complementary medicine practitioners	6.7 (5.8 to 8.3)
Andorra	Audiologists, speech therapists, and counsellors	13.8 (8.2 to 21.0)
Andorra	Clinical officers and medical assistants	4.9 (2.8 to 8.1)
Andorra	Dieticians and nutritionists	11.1 (6.9 to 17.0)

Andorra	Environmental health workers	9.6 (6.2 to 14.3)
Andorra	Healthcare aides, paramedics, and ambulance workers	136.6 (101.6 to 184.9)
Andorra	Home-based personal care workers	65.7 (45.8 to 92.1)
Andorra	Medical imaging and therapeutic equipment technicians	13.8 (8.0 to 21.6)
Andorra	Medical laboratory technicians	15.1 (9.1 to 23.3)
Andorra	Optometrists and opticians	9.4 (6.2 to 14.1)
Andorra	Physiotherapists and prosthetic technicians	27.4 (17.7 to 40.5)
Andorra	Psychologists	29.1 (19.0 to 43.0)
Andorra	Traditional and complementary medicine practitioners	4.6 (2.8 to 7.1)
Austria	Audiologists, speech therapists, and counsellors	18.8 (13.9 to 24.4)
Austria	Clinical officers and medical assistants	4.4 (3.3 to 6.1)
Austria	Dieticians and nutritionists	8.0 (5.9 to 10.8)
Austria	Environmental health workers	6.2 (4.4 to 8.3)
Austria	Healthcare aides, paramedics, and ambulance workers	66.5 (49.1 to 86.8)
Austria	Home-based personal care workers	33.1 (25.4 to 42.1)
Austria	Medical imaging and therapeutic equipment technicians	6.4 (4.5 to 9.0)
Austria	Medical laboratory technicians	6.7 (4.5 to 9.7)
Austria	Optometrists and opticians	4.9 (3.6 to 6.4)
Austria	Physiotherapists and prosthetic technicians	16.3 (11.8 to 21.3)
Austria	Psychologists	7.7 (5.6 to 10.3)
Austria	Traditional and complementary medicine practitioners	14.3 (9.7 to 21.4)
Belgium	Audiologists, speech therapists, and counsellors	18.9 (14.1 to 25.0)
Belgium	Clinical officers and medical assistants	7.8 (5.7 to 10.5)
Belgium	Dieticians and nutritionists	5.6 (4.0 to 7.9)
Belgium	Environmental health workers	7.3 (5.1 to 10.0)
Belgium	Healthcare aides, paramedics, and ambulance workers	59.7 (45.6 to 77.2)
Belgium	Home-based personal care workers	61.9 (46.0 to 78.6)
Belgium	Medical imaging and therapeutic equipment technicians	6.3 (4.3 to 9.0)
Belgium	Medical laboratory technicians	7.4 (4.9 to 10.5)
Belgium	Optometrists and opticians	3.4 (2.4 to 4.5)

Belgium	Physiotherapists and prosthetic technicians	35.0 (24.9 to 46.0)
Belgium	Psychologists	13.2 (9.8 to 17.4)
Belgium	Traditional and complementary medicine practitioners	3.7 (2.5 to 5.6)
Cyprus	Audiologists, speech therapists, and counsellors	4.5 (3.4 to 6.0)
Cyprus	Clinical officers and medical assistants	0.8 (0.6 to 1.1)
Cyprus	Dieticians and nutritionists	4.7 (3.6 to 6.3)
Cyprus	Environmental health workers	2.8 (2.1 to 3.7)
Cyprus	Healthcare aides, paramedics, and ambulance workers	24.7 (17.8 to 33.9)
Cyprus	Home-based personal care workers	16.8 (13.0 to 21.6)
Cyprus	Medical imaging and therapeutic equipment technicians	4.3 (3.0 to 6.0)
Cyprus	Medical laboratory technicians	5.1 (3.6 to 6.8)
Cyprus	Optometrists and opticians	8.9 (7.1 to 11.4)
Cyprus	Physiotherapists and prosthetic technicians	11.8 (9.2 to 15.2)
Cyprus	Psychologists	11.6 (8.4 to 15.2)
Cyprus	Traditional and complementary medicine practitioners	1.0 (0.7 to 1.4)
Denmark	Audiologists, speech therapists, and counsellors	11.5 (8.7 to 15.1)
Denmark	Clinical officers and medical assistants	1.5 (1.1 to 2.1)
Denmark	Dieticians and nutritionists	10.2 (8.0 to 13.2)
Denmark	Environmental health workers	7.5 (5.7 to 9.7)
Denmark	Healthcare aides, paramedics, and ambulance workers	213.1 (154.7 to 286.6)
Denmark	Home-based personal care workers	48.3 (37.9 to 60.7)
Denmark	Medical imaging and therapeutic equipment technicians	9.6 (7.1 to 12.3)
Denmark	Medical laboratory technicians	9.7 (6.6 to 13.4)
Denmark	Optometrists and opticians	8.0 (6.2 to 9.9)
Denmark	Physiotherapists and prosthetic technicians	27.7 (21.3 to 34.4)
Denmark	Psychologists	15.0 (11.3 to 19.2)
Denmark	Traditional and complementary medicine practitioners	6.3 (4.2 to 9.0)
Finland	Audiologists, speech therapists, and counsellors	6.8 (4.8 to 9.1)
Finland	Clinical officers and medical assistants	2.5 (1.7 to 3.5)
Finland	Dieticians and nutritionists	4.3 (3.2 to 5.6)

Finland	Environmental health workers	5.6 (4.1 to 7.4)
Finland	Healthcare aides, paramedics, and ambulance workers	151.7 (114.9 to 200.3)
Finland	Home-based personal care workers	52.4 (41.2 to 65.8)
Finland	Medical imaging and therapeutic equipment technicians	4.9 (3.5 to 6.7)
Finland	Medical laboratory technicians	13.8 (9.4 to 19.5)
Finland	Optometrists and opticians	3.9 (3.0 to 5.1)
Finland	Physiotherapists and prosthetic technicians	13.1 (9.9 to 17.1)
Finland	Psychologists	7.8 (5.8 to 9.9)
Finland	Traditional and complementary medicine practitioners	3.0 (2.0 to 4.4)
France	Audiologists, speech therapists, and counsellors	6.0 (3.8 to 9.1)
France	Clinical officers and medical assistants	2.5 (1.5 to 3.8)
France	Dieticians and nutritionists	4.3 (2.9 to 6.3)
France	Environmental health workers	5.4 (3.5 to 7.6)
France	Healthcare aides, paramedics, and ambulance workers	63.0 (39.7 to 95.2)
France	Home-based personal care workers	34.3 (24.2 to 47.1)
France	Medical imaging and therapeutic equipment technicians	5.4 (3.3 to 8.2)
France	Medical laboratory technicians	6.7 (4.1 to 10.3)
France	Optometrists and opticians	3.8 (2.7 to 5.4)
France	Physiotherapists and prosthetic technicians	14.4 (10.0 to 20.6)
France	Psychologists	8.2 (5.4 to 11.6)
France	Traditional and complementary medicine practitioners	4.7 (3.1 to 7.0)
Germany	Audiologists, speech therapists, and counsellors	9.1 (6.9 to 11.9)
Germany	Clinical officers and medical assistants	28.2 (21.5 to 36.4)
Germany	Dieticians and nutritionists	7.0 (5.3 to 9.1)
Germany	Environmental health workers	9.1 (6.8 to 11.7)
Germany	Healthcare aides, paramedics, and ambulance workers	56.6 (42.1 to 74.1)
Germany	Home-based personal care workers	20.0 (16.0 to 24.2)
Germany	Medical imaging and therapeutic equipment technicians	7.3 (5.5 to 9.4)
Germany	Medical laboratory technicians	15.9 (12.3 to 20.1)
Germany	Optometrists and opticians	10.3 (8.1 to 13.2)

Germany	Physiotherapists and prosthetic technicians	42.2 (33.4 to 53.4)
Germany	Psychologists	16.1 (12.3 to 20.6)
Germany	Traditional and complementary medicine practitioners	5.9 (4.3 to 7.8)
Greece	Audiologists, speech therapists, and counsellors	2.7 (2.0 to 3.6)
Greece	Clinical officers and medical assistants	0.9 (0.7 to 1.3)
Greece	Dieticians and nutritionists	3.0 (2.3 to 3.8)
Greece	Environmental health workers	2.6 (1.9 to 3.4)
Greece	Healthcare aides, paramedics, and ambulance workers	25.3 (17.5 to 34.5)
Greece	Home-based personal care workers	12.6 (9.9 to 15.8)
Greece	Medical imaging and therapeutic equipment technicians	3.3 (2.3 to 4.5)
Greece	Medical laboratory technicians	3.8 (2.8 to 5.1)
Greece	Optometrists and opticians	2.5 (2.0 to 3.2)
Greece	Physiotherapists and prosthetic technicians	6.7 (5.2 to 8.8)
Greece	Psychologists	7.2 (5.7 to 9.1)
Greece	Traditional and complementary medicine practitioners	0.9 (0.6 to 1.4)
Iceland	Audiologists, speech therapists, and counsellors	9.1 (6.4 to 12.3)
Iceland	Clinical officers and medical assistants	4.8 (3.4 to 6.5)
Iceland	Dieticians and nutritionists	6.6 (4.8 to 8.9)
Iceland	Environmental health workers	7.4 (5.4 to 9.7)
Iceland	Healthcare aides, paramedics, and ambulance workers	159.4 (120.8 to 210.2)
Iceland	Home-based personal care workers	33.0 (25.9 to 41.4)
Iceland	Medical imaging and therapeutic equipment technicians	8.8 (6.0 to 12.5)
Iceland	Medical laboratory technicians	8.0 (5.5 to 11.2)
Iceland	Optometrists and opticians	5.3 (4.0 to 6.6)
Iceland	Physiotherapists and prosthetic technicians	35.9 (27.0 to 47.7)
Iceland	Psychologists	21.8 (15.9 to 28.3)
Iceland	Traditional and complementary medicine practitioners	3.6 (2.5 to 5.4)
Ireland	Audiologists, speech therapists, and counsellors	12.4 (9.1 to 16.7)
Ireland	Clinical officers and medical assistants	3.5 (2.5 to 4.7)
Ireland	Dieticians and nutritionists	6.3 (4.7 to 8.4)

Ireland	Environmental health workers	13.1 (9.7 to 17.2)
Ireland	Healthcare aides, paramedics, and ambulance workers	160.7 (122.5 to 209.1)
Ireland	Home-based personal care workers	63.1 (50.7 to 75.7)
Ireland	Medical imaging and therapeutic equipment technicians	5.6 (4.3 to 7.6)
Ireland	Medical laboratory technicians	9.7 (7.0 to 13.3)
Ireland	Optometrists and opticians	7.5 (5.9 to 9.6)
Ireland	Physiotherapists and prosthetic technicians	22.0 (17.0 to 28.8)
Ireland	Psychologists	8.0 (6.0 to 10.6)
Ireland	Traditional and complementary medicine practitioners	2.9 (2.0 to 4.4)
Israel	Audiologists, speech therapists, and counsellors	9.4 (6.3 to 13.8)
Israel	Clinical officers and medical assistants	4.3 (2.7 to 6.3)
Israel	Dieticians and nutritionists	5.1 (3.5 to 7.3)
Israel	Environmental health workers	10.7 (7.2 to 14.6)
Israel	Healthcare aides, paramedics, and ambulance workers	61.3 (40.8 to 86.1)
Israel	Home-based personal care workers	112.2 (83.9 to 144.9)
Israel	Medical imaging and therapeutic equipment technicians	9.9 (6.2 to 14.7)
Israel	Medical laboratory technicians	14.0 (9.0 to 20.2)
Israel	Optometrists and opticians	1.7 (1.2 to 2.4)
Israel	Physiotherapists and prosthetic technicians	18.4 (12.9 to 25.5)
Israel	Psychologists	14.6 (10.2 to 20.0)
Israel	Traditional and complementary medicine practitioners	21.4 (14.6 to 31.7)
Italy	Audiologists, speech therapists, and counsellors	6.2 (4.2 to 8.4)
Italy	Clinical officers and medical assistants	2.7 (1.9 to 3.9)
Italy	Dieticians and nutritionists	4.4 (3.3 to 5.9)
Italy	Environmental health workers	10.0 (7.2 to 13.7)
Italy	Healthcare aides, paramedics, and ambulance workers	42.8 (30.9 to 58.2)
Italy	Home-based personal care workers	49.2 (39.0 to 62.4)
Italy	Medical imaging and therapeutic equipment technicians	3.0 (2.2 to 4.2)
Italy	Medical laboratory technicians	6.6 (4.7 to 9.0)
Italy	Optometrists and opticians	3.5 (2.6 to 4.7)

Italy	Physiotherapists and prosthetic technicians	20.9 (15.5 to 27.5)
Italy	Psychologists	7.3 (5.4 to 9.4)
Italy	Traditional and complementary medicine practitioners	3.6 (2.4 to 5.4)
Luxembourg	Audiologists, speech therapists, and counsellors	5.5 (4.1 to 7.3)
Luxembourg	Clinical officers and medical assistants	0.6 (0.4 to 0.9)
Luxembourg	Dieticians and nutritionists	3.7 (2.8 to 4.8)
Luxembourg	Environmental health workers	9.6 (7.0 to 12.7)
Luxembourg	Healthcare aides, paramedics, and ambulance workers	101.1 (74.9 to 136.5)
Luxembourg	Home-based personal care workers	19.9 (16.0 to 25.1)
Luxembourg	Medical imaging and therapeutic equipment technicians	5.5 (3.8 to 7.7)
Luxembourg	Medical laboratory technicians	4.6 (3.2 to 6.3)
Luxembourg	Optometrists and opticians	2.5 (1.9 to 3.2)
Luxembourg	Physiotherapists and prosthetic technicians	12.3 (9.3 to 16.4)
Luxembourg	Psychologists	13.2 (10.4 to 16.6)
Luxembourg	Traditional and complementary medicine practitioners	1.7 (1.2 to 2.6)
Malta	Audiologists, speech therapists, and counsellors	8.8 (5.2 to 13.5)
Malta	Clinical officers and medical assistants	3.6 (2.1 to 6.0)
Malta	Dieticians and nutritionists	7.3 (4.5 to 11.3)
Malta	Environmental health workers	7.8 (5.1 to 11.8)
Malta	Healthcare aides, paramedics, and ambulance workers	103.4 (77.6 to 138.9)
Malta	Home-based personal care workers	40.6 (27.7 to 57.9)
Malta	Medical imaging and therapeutic equipment technicians	8.3 (4.7 to 13.2)
Malta	Medical laboratory technicians	11.7 (7.0 to 18.2)
Malta	Optometrists and opticians	6.5 (4.2 to 9.8)
Malta	Physiotherapists and prosthetic technicians	20.8 (13.4 to 30.8)
Malta	Psychologists	19.2 (12.4 to 28.5)
Malta	Traditional and complementary medicine practitioners	4.1 (2.6 to 6.5)
Monaco	Audiologists, speech therapists, and counsellors	10.8 (6.3 to 16.6)
Monaco	Clinical officers and medical assistants	3.6 (2.1 to 6.2)
Monaco	Dieticians and nutritionists	9.1 (5.6 to 14.3)

Monaco	Environmental health workers	7.9 (5.0 to 11.8)
Monaco	Healthcare aides, paramedics, and ambulance workers	123.6 (92.5 to 165.9)
Monaco	Home-based personal care workers	53.6 (36.9 to 75.8)
Monaco	Medical imaging and therapeutic equipment technicians	11.2 (6.3 to 17.7)
Monaco	Medical laboratory technicians	11.9 (7.1 to 18.6)
Monaco	Optometrists and opticians	7.7 (4.9 to 11.6)
Monaco	Physiotherapists and prosthetic technicians	23.3 (15.0 to 34.3)
Monaco	Psychologists	22.6 (14.6 to 33.7)
Monaco	Traditional and complementary medicine practitioners	3.4 (2.1 to 5.4)
Netherlands	Audiologists, speech therapists, and counsellors	17.4 (13.4 to 22.8)
Netherlands	Clinical officers and medical assistants	2.1 (1.5 to 2.9)
Netherlands	Dieticians and nutritionists	15.0 (11.8 to 19.0)
Netherlands	Environmental health workers	10.4 (8.1 to 13.1)
Netherlands	Healthcare aides, paramedics, and ambulance workers	141.1 (104.7 to 187.7)
Netherlands	Home-based personal care workers	72.8 (58.6 to 91.7)
Netherlands	Medical imaging and therapeutic equipment technicians	13.4 (10.4 to 18.0)
Netherlands	Medical laboratory technicians	13.3 (9.7 to 17.6)
Netherlands	Optometrists and opticians	11.3 (8.9 to 13.8)
Netherlands	Physiotherapists and prosthetic technicians	42.0 (32.7 to 52.0)
Netherlands	Psychologists	26.0 (20.5 to 32.2)
Netherlands	Traditional and complementary medicine practitioners	3.9 (2.7 to 5.7)
Norway	Audiologists, speech therapists, and counsellors	12.6 (9.2 to 16.5)
Norway	Clinical officers and medical assistants	10.4 (7.6 to 14.4)
Norway	Dieticians and nutritionists	8.2 (6.2 to 10.9)
Norway	Environmental health workers	12.3 (9.2 to 16.0)
Norway	Healthcare aides, paramedics, and ambulance workers	314.9 (231.8 to 422.9)
Norway	Home-based personal care workers	28.2 (22.3 to 34.4)
Norway	Medical imaging and therapeutic equipment technicians	9.2 (6.7 to 12.6)
Norway	Medical laboratory technicians	13.6 (9.5 to 18.5)
Norway	Optometrists and opticians	7.5 (5.9 to 9.8)

Norway	Physiotherapists and prosthetic technicians	40.7 (31.9 to 52.7)
Norway	Psychologists	11.7 (8.7 to 15.2)
Norway	Traditional and complementary medicine practitioners	7.4 (5.0 to 11.0)
Portugal	Audiologists, speech therapists, and counsellors	7.3 (5.4 to 9.7)
Portugal	Clinical officers and medical assistants	0.7 (0.5 to 1.0)
Portugal	Dieticians and nutritionists	6.8 (5.2 to 9.1)
Portugal	Environmental health workers	10.5 (7.7 to 13.5)
Portugal	Healthcare aides, paramedics, and ambulance workers	101.7 (75.5 to 132.1)
Portugal	Home-based personal care workers	21.9 (17.7 to 26.8)
Portugal	Medical imaging and therapeutic equipment technicians	6.3 (4.9 to 8.5)
Portugal	Medical laboratory technicians	8.7 (6.2 to 11.7)
Portugal	Optometrists and opticians	3.5 (2.8 to 4.5)
Portugal	Physiotherapists and prosthetic technicians	9.8 (7.6 to 12.7)
Portugal	Psychologists	23.7 (18.3 to 29.9)
Portugal	Traditional and complementary medicine practitioners	1.1 (0.8 to 1.7)
San Marino	Audiologists, speech therapists, and counsellors	9.9 (5.9 to 14.9)
San Marino	Clinical officers and medical assistants	3.4 (1.9 to 5.7)
San Marino	Dieticians and nutritionists	7.6 (4.7 to 11.8)
San Marino	Environmental health workers	6.5 (4.2 to 9.7)
San Marino	Healthcare aides, paramedics, and ambulance workers	84.7 (61.4 to 117.4)
San Marino	Home-based personal care workers	52.0 (36.8 to 72.0)
San Marino	Medical imaging and therapeutic equipment technicians	9.8 (5.7 to 15.1)
San Marino	Medical laboratory technicians	10.3 (6.2 to 16.0)
San Marino	Optometrists and opticians	6.9 (4.5 to 10.2)
San Marino	Physiotherapists and prosthetic technicians	20.0 (13.0 to 29.2)
San Marino	Psychologists	17.9 (11.7 to 26.5)
San Marino	Traditional and complementary medicine practitioners	4.1 (2.5 to 6.2)
Spain	Audiologists, speech therapists, and counsellors	7.6 (5.4 to 10.1)
Spain	Clinical officers and medical assistants	2.7 (1.9 to 3.8)
Spain	Dieticians and nutritionists	2.8 (2.0 to 3.9)

Spain	Environmental health workers	3.2 (2.2 to 4.3)
Spain	Healthcare aides, paramedics, and ambulance workers	116.7 (90.0 to 148.4)
Spain	Home-based personal care workers	37.1 (28.0 to 47.1)
Spain	Medical imaging and therapeutic equipment technicians	5.7 (4.0 to 8.2)
Spain	Medical laboratory technicians	11.8 (7.8 to 16.9)
Spain	Optometrists and opticians	4.4 (3.1 to 6.1)
Spain	Physiotherapists and prosthetic technicians	12.9 (9.2 to 18.0)
Spain	Psychologists	13.1 (9.7 to 17.6)
Spain	Traditional and complementary medicine practitioners	6.3 (4.3 to 9.3)
Sweden	Audiologists, speech therapists, and counsellors	12.1 (8.8 to 16.1)
Sweden	Clinical officers and medical assistants	4.5 (3.1 to 6.5)
Sweden	Dieticians and nutritionists	10.2 (7.6 to 13.7)
Sweden	Environmental health workers	12.6 (9.4 to 16.9)
Sweden	Healthcare aides, paramedics, and ambulance workers	226.5 (168.2 to 295.5)
Sweden	Home-based personal care workers	159.3 (121.3 to 199.9)
Sweden	Medical imaging and therapeutic equipment technicians	10.5 (7.1 to 14.5)
Sweden	Medical laboratory technicians	10.0 (6.9 to 13.8)
Sweden	Optometrists and opticians	7.2 (5.3 to 9.4)
Sweden	Physiotherapists and prosthetic technicians	32.9 (24.3 to 42.8)
Sweden	Psychologists	14.5 (10.7 to 19.1)
Sweden	Traditional and complementary medicine practitioners	8.4 (5.6 to 12.5)
Switzerland	Audiologists, speech therapists, and counsellors	11.9 (9.2 to 15.6)
Switzerland	Clinical officers and medical assistants	21.6 (15.0 to 29.8)
Switzerland	Dieticians and nutritionists	8.9 (6.7 to 12.1)
Switzerland	Environmental health workers	11.2 (8.0 to 14.9)
Switzerland	Healthcare aides, paramedics, and ambulance workers	75.9 (57.5 to 98.9)
Switzerland	Home-based personal care workers	41.0 (32.3 to 52.0)
Switzerland	Medical imaging and therapeutic equipment technicians	11.5 (7.9 to 16.4)
Switzerland	Medical laboratory technicians	6.3 (4.2 to 9.1)
Switzerland	Optometrists and opticians	9.6 (7.1 to 12.9)

Switzerland	Physiotherapists and prosthetic technicians	63.8 (47.0 to 81.7)
Switzerland	Psychologists	14.2 (10.6 to 18.4)
Switzerland	Traditional and complementary medicine practitioners	14.8 (10.1 to 22.1)
UK	Audiologists, speech therapists, and counsellors	3.1 (2.3 to 4.2)
UK	Clinical officers and medical assistants	4.0 (2.8 to 5.5)
UK	Dieticians and nutritionists	8.0 (6.1 to 10.2)
UK	Environmental health workers	15.7 (11.8 to 20.5)
UK	Healthcare aides, paramedics, and ambulance workers	59.3 (45.5 to 76.2)
UK	Home-based personal care workers	165.9 (132.5 to 202.1)
UK	Medical imaging and therapeutic equipment technicians	8.0 (5.5 to 11.1)
UK	Medical laboratory technicians	9.6 (6.6 to 13.4)
UK	Optometrists and opticians	4.3 (3.3 to 5.4)
UK	Physiotherapists and prosthetic technicians	19.7 (14.8 to 24.8)
UK	Psychologists	6.3 (4.9 to 8.1)
UK	Traditional and complementary medicine practitioners	10.2 (6.8 to 14.6)
**Latin America and Caribbean**	Audiologists, speech therapists, and counsellors	2.9 (2.1 to 3.8)
**Latin America and Caribbean**	Clinical officers and medical assistants	1.8 (1.5 to 2.2)
**Latin America and Caribbean**	Dieticians and nutritionists	2.8 (2.4 to 3.1)
**Latin America and Caribbean**	Environmental health workers	10.5 (8.1 to 13.0)
**Latin America and Caribbean**	Healthcare aides, paramedics, and ambulance workers	13.3 (9.8 to 18.1)
**Latin America and Caribbean**	Home-based personal care workers	5.5 (4.1 to 7.0)
**Latin America and Caribbean**	Medical imaging and therapeutic equipment technicians	2.5 (1.7 to 3.6)
**Latin America and Caribbean**	Medical laboratory technicians	5.3 (4.7 to 6.1)
**Latin America and Caribbean**	Optometrists and opticians	1.1 (0.8 to 1.5)
**Latin America and Caribbean**	Physiotherapists and prosthetic technicians	9.1 (8.4 to 9.9)
**Latin America and Caribbean**	Psychologists	8.5 (7.8 to 9.3)
**Latin America and Caribbean**	Traditional and complementary medicine practitioners	1.3 (1.1 to 1.6)
Andean Latin America	Audiologists, speech therapists, and counsellors	2.4 (1.6 to 3.6)
Andean Latin America	Clinical officers and medical assistants	2.1 (1.8 to 2.6)
Andean Latin America	Dieticians and nutritionists	1.4 (0.9 to 1.9)

Andean Latin America	Environmental health workers	0.8 (0.6 to 1.1)
Andean Latin America	Healthcare aides, paramedics, and ambulance workers	12.0 (9.8 to 15.2)
Andean Latin America	Home-based personal care workers	27.1 (20.6 to 35.2)
Andean Latin America	Medical imaging and therapeutic equipment technicians	1.8 (1.3 to 2.4)
Andean Latin America	Medical laboratory technicians	11.7 (8.6 to 16.0)
Andean Latin America	Optometrists and opticians	1.2 (0.8 to 1.6)
Andean Latin America	Physiotherapists and prosthetic technicians	9.3 (7.2 to 12.2)
Andean Latin America	Psychologists	11.3 (9.2 to 13.7)
Andean Latin America	Traditional and complementary medicine practitioners	2.6 (1.8 to 3.8)
Bolivia	Audiologists, speech therapists, and counsellors	0.3 (0.2 to 0.4)
Bolivia	Clinical officers and medical assistants	4.2 (3.0 to 5.7)
Bolivia	Dieticians and nutritionists	1.7 (1.3 to 2.4)
Bolivia	Environmental health workers	1.2 (0.9 to 1.7)
Bolivia	Healthcare aides, paramedics, and ambulance workers	3.3 (2.3 to 4.7)
Bolivia	Home-based personal care workers	2.4 (1.7 to 3.0)
Bolivia	Medical imaging and therapeutic equipment technicians	1.8 (1.3 to 2.4)
Bolivia	Medical laboratory technicians	4.1 (2.8 to 5.6)
Bolivia	Optometrists and opticians	0.8 (0.6 to 1.1)
Bolivia	Physiotherapists and prosthetic technicians	5.7 (4.2 to 7.8)
Bolivia	Psychologists	8.1 (5.9 to 10.6)
Bolivia	Traditional and complementary medicine practitioners	2.7 (1.9 to 3.9)
Ecuador	Audiologists, speech therapists, and counsellors	0.9 (0.6 to 1.2)
Ecuador	Clinical officers and medical assistants	1.5 (1.0 to 2.0)
Ecuador	Dieticians and nutritionists	0.9 (0.6 to 1.2)
Ecuador	Environmental health workers	0.7 (0.4 to 0.9)
Ecuador	Healthcare aides, paramedics, and ambulance workers	14.7 (10.2 to 20.0)
Ecuador	Home-based personal care workers	8.7 (7.2 to 10.7)
Ecuador	Medical imaging and therapeutic equipment technicians	1.6 (1.2 to 2.2)
Ecuador	Medical laboratory technicians	4.7 (3.5 to 6.0)
Ecuador	Optometrists and opticians	1.1 (0.8 to 1.4)

Ecuador	Physiotherapists and prosthetic technicians	5.5 (4.4 to 7.0)
Ecuador	Psychologists	7.9 (6.0 to 9.8)
Ecuador	Traditional and complementary medicine practitioners	1.0 (0.7 to 1.5)
Peru	Audiologists, speech therapists, and counsellors	3.8 (2.4 to 5.8)
Peru	Clinical officers and medical assistants	1.8 (1.1 to 2.8)
Peru	Dieticians and nutritionists	1.5 (1.0 to 2.1)
Peru	Environmental health workers	0.8 (0.5 to 1.1)
Peru	Healthcare aides, paramedics, and ambulance workers	13.6 (8.6 to 20.5)
Peru	Home-based personal care workers	44.4 (32.1 to 59.1)
Peru	Medical imaging and therapeutic equipment technicians	1.9 (1.2 to 2.9)
Peru	Medical laboratory technicians	17.7 (11.2 to 26.4)
Peru	Optometrists and opticians	1.3 (0.9 to 1.9)
Peru	Physiotherapists and prosthetic technicians	12.5 (8.7 to 17.7)
Peru	Psychologists	14.0 (9.3 to 19.6)
Peru	Traditional and complementary medicine practitioners	3.3 (2.2 to 5.0)
Caribbean	Audiologists, speech therapists, and counsellors	1.9 (1.6 to 2.2)
Caribbean	Clinical officers and medical assistants	0.8 (0.7 to 1.1)
Caribbean	Dieticians and nutritionists	1.5 (1.3 to 1.8)
Caribbean	Environmental health workers	2.2 (1.9 to 2.5)
Caribbean	Healthcare aides, paramedics, and ambulance workers	14.7 (12.9 to 16.7)
Caribbean	Home-based personal care workers	7.7 (6.7 to 8.8)
Caribbean	Medical imaging and therapeutic equipment technicians	1.2 (1.1 to 1.4)
Caribbean	Medical laboratory technicians	4.1 (3.4 to 4.8)
Caribbean	Optometrists and opticians	0.7 (0.6 to 1.0)
Caribbean	Physiotherapists and prosthetic technicians	3.1 (2.6 to 3.5)
Caribbean	Psychologists	3.2 (2.5 to 4.3)
Caribbean	Traditional and complementary medicine practitioners	0.9 (0.6 to 1.4)
Antigua and Barbuda	Audiologists, speech therapists, and counsellors	3.5 (2.2 to 5.3)
Antigua and Barbuda	Clinical officers and medical assistants	1.6 (0.9 to 2.6)
Antigua and Barbuda	Dieticians and nutritionists	4.1 (2.5 to 6.1)

Antigua and Barbuda	Environmental health workers	3.1 (2.0 to 4.5)
Antigua and Barbuda	Healthcare aides, paramedics, and ambulance workers	21.4 (14.9 to 31.5)
Antigua and Barbuda	Home-based personal care workers	14.2 (10.0 to 19.6)
Antigua and Barbuda	Medical imaging and therapeutic equipment technicians	2.2 (1.3 to 3.4)
Antigua and Barbuda	Medical laboratory technicians	9.2 (5.7 to 13.9)
Antigua and Barbuda	Optometrists and opticians	2.4 (1.6 to 3.5)
Antigua and Barbuda	Physiotherapists and prosthetic technicians	7.4 (4.8 to 10.7)
Antigua and Barbuda	Psychologists	8.3 (5.5 to 12.2)
Antigua and Barbuda	Traditional and complementary medicine practitioners	0.9 (0.6 to 1.4)
The Bahamas	Audiologists, speech therapists, and counsellors	9.8 (5.8 to 15.3)
The Bahamas	Clinical officers and medical assistants	4.3 (2.5 to 7.3)
The Bahamas	Dieticians and nutritionists	10.3 (6.4 to 15.9)
The Bahamas	Environmental health workers	10.8 (7.0 to 16.2)
The Bahamas	Healthcare aides, paramedics, and ambulance workers	109.0 (82.1 to 146.1)
The Bahamas	Home-based personal care workers	38.7 (26.6 to 55.1)
The Bahamas	Medical imaging and therapeutic equipment technicians	6.6 (3.8 to 10.6)
The Bahamas	Medical laboratory technicians	19.1 (11.6 to 29.3)
The Bahamas	Optometrists and opticians	3.8 (2.4 to 5.7)
The Bahamas	Physiotherapists and prosthetic technicians	17.8 (11.4 to 26.4)
The Bahamas	Psychologists	17.4 (11.1 to 25.8)
The Bahamas	Traditional and complementary medicine practitioners	2.1 (1.2 to 3.2)
Barbados	Audiologists, speech therapists, and counsellors	4.7 (2.9 to 7.3)
Barbados	Clinical officers and medical assistants	2.2 (1.3 to 3.6)
Barbados	Dieticians and nutritionists	5.2 (3.3 to 8.0)
Barbados	Environmental health workers	4.6 (3.0 to 6.8)
Barbados	Healthcare aides, paramedics, and ambulance workers	35.6 (25.2 to 50.8)
Barbados	Home-based personal care workers	18.7 (13.1 to 26.1)
Barbados	Medical imaging and therapeutic equipment technicians	3.0 (1.7 to 4.7)
Barbados	Medical laboratory technicians	11.4 (7.0 to 17.4)
Barbados	Optometrists and opticians	2.6 (1.7 to 3.9)

Barbados	Physiotherapists and prosthetic technicians	9.5 (6.2 to 13.8)
Barbados	Psychologists	10.1 (6.7 to 14.8)
Barbados	Traditional and complementary medicine practitioners	1.2 (0.8 to 1.9)
Belize	Audiologists, speech therapists, and counsellors	0.9 (0.6 to 1.4)
Belize	Clinical officers and medical assistants	0.6 (0.3 to 0.9)
Belize	Dieticians and nutritionists	0.9 (0.6 to 1.4)
Belize	Environmental health workers	1.0 (0.7 to 1.5)
Belize	Healthcare aides, paramedics, and ambulance workers	4.6 (3.1 to 7.0)
Belize	Home-based personal care workers	3.7 (2.6 to 5.0)
Belize	Medical imaging and therapeutic equipment technicians	0.5 (0.3 to 0.8)
Belize	Medical laboratory technicians	2.8 (1.8 to 4.3)
Belize	Optometrists and opticians	0.6 (0.4 to 0.9)
Belize	Physiotherapists and prosthetic technicians	2.0 (1.3 to 2.9)
Belize	Psychologists	1.9 (1.2 to 2.8)
Belize	Traditional and complementary medicine practitioners	0.6 (0.4 to 0.9)
Bermuda	Audiologists, speech therapists, and counsellors	11.6 (6.6 to 19.6)
Bermuda	Clinical officers and medical assistants	5.0 (2.8 to 8.5)
Bermuda	Dieticians and nutritionists	13.1 (7.6 to 20.8)
Bermuda	Environmental health workers	26.1 (15.6 to 39.9)
Bermuda	Healthcare aides, paramedics, and ambulance workers	359.5 (292.3 to 437.7)
Bermuda	Home-based personal care workers	70.0 (46.1 to 102.5)
Bermuda	Medical imaging and therapeutic equipment technicians	8.1 (4.5 to 13.1)
Bermuda	Medical laboratory technicians	23.5 (13.8 to 38.1)
Bermuda	Optometrists and opticians	4.4 (2.7 to 7.0)
Bermuda	Physiotherapists and prosthetic technicians	42.4 (26.3 to 66.6)
Bermuda	Psychologists	11.1 (6.7 to 16.8)
Bermuda	Traditional and complementary medicine practitioners	5.7 (3.4 to 9.2)
Cuba	Audiologists, speech therapists, and counsellors	3.0 (2.0 to 4.3)
Cuba	Clinical officers and medical assistants	0.9 (0.6 to 1.4)
Cuba	Dieticians and nutritionists	2.0 (1.4 to 2.8)

Cuba	Environmental health workers	3.7 (2.6 to 5.3)
Cuba	Healthcare aides, paramedics, and ambulance workers	30.4 (24.1 to 38.5)
Cuba	Home-based personal care workers	9.8 (7.1 to 12.7)
Cuba	Medical imaging and therapeutic equipment technicians	2.4 (1.6 to 3.4)
Cuba	Medical laboratory technicians	8.7 (5.8 to 12.6)
Cuba	Optometrists and opticians	0.9 (0.6 to 1.3)
Cuba	Physiotherapists and prosthetic technicians	3.9 (2.6 to 5.7)
Cuba	Psychologists	1.6 (1.1 to 2.4)
Cuba	Traditional and complementary medicine practitioners	0.9 (0.6 to 1.4)
Dominica	Audiologists, speech therapists, and counsellors	1.2 (0.7 to 1.8)
Dominica	Clinical officers and medical assistants	0.5 (0.3 to 0.8)
Dominica	Dieticians and nutritionists	1.3 (0.8 to 1.9)
Dominica	Environmental health workers	0.7 (0.5 to 1.1)
Dominica	Healthcare aides, paramedics, and ambulance workers	3.5 (2.3 to 5.5)
Dominica	Home-based personal care workers	5.6 (4.1 to 7.4)
Dominica	Medical imaging and therapeutic equipment technicians	0.7 (0.4 to 1.1)
Dominica	Medical laboratory technicians	3.0 (1.9 to 4.5)
Dominica	Optometrists and opticians	1.0 (0.7 to 1.4)
Dominica	Physiotherapists and prosthetic technicians	2.3 (1.5 to 3.3)
Dominica	Psychologists	2.5 (1.7 to 3.7)
Dominica	Traditional and complementary medicine practitioners	0.4 (0.3 to 0.7)
Dominican Republic	Audiologists, speech therapists, and counsellors	2.1 (1.6 to 2.7)
Dominican Republic	Clinical officers and medical assistants	1.0 (0.7 to 1.3)
Dominican Republic	Dieticians and nutritionists	1.9 (1.4 to 2.4)
Dominican Republic	Environmental health workers	2.5 (1.8 to 3.1)
Dominican Republic	Healthcare aides, paramedics, and ambulance workers	13.3 (10.0 to 17.5)
Dominican Republic	Home-based personal care workers	9.1 (7.2 to 11.3)
Dominican Republic	Medical imaging and therapeutic equipment technicians	1.2 (0.8 to 1.8)
Dominican Republic	Medical laboratory technicians	2.6 (1.7 to 3.6)
Dominican Republic	Optometrists and opticians	0.9 (0.7 to 1.2)

Dominican Republic	Physiotherapists and prosthetic technicians	4.1 (3.1 to 5.2)
Dominican Republic	Psychologists	8.6 (6.6 to 10.9)
Dominican Republic	Traditional and complementary medicine practitioners	1.0 (0.7 to 1.5)
Grenada	Audiologists, speech therapists, and counsellors	1.2 (0.7 to 1.8)
Grenada	Clinical officers and medical assistants	0.7 (0.4 to 1.1)
Grenada	Dieticians and nutritionists	1.2 (0.8 to 1.9)
Grenada	Environmental health workers	1.2 (0.8 to 1.8)
Grenada	Healthcare aides, paramedics, and ambulance workers	6.4 (4.4 to 9.6)
Grenada	Home-based personal care workers	4.8 (3.4 to 6.5)
Grenada	Medical imaging and therapeutic equipment technicians	0.7 (0.4 to 1.0)
Grenada	Medical laboratory technicians	3.3 (2.1 to 5.1)
Grenada	Optometrists and opticians	0.7 (0.5 to 1.1)
Grenada	Physiotherapists and prosthetic technicians	2.5 (1.6 to 3.6)
Grenada	Psychologists	2.4 (1.6 to 3.5)
Grenada	Traditional and complementary medicine practitioners	0.6 (0.4 to 0.9)
Guyana	Audiologists, speech therapists, and counsellors	2.6 (1.7 to 3.7)
Guyana	Clinical officers and medical assistants	1.5 (1.0 to 2.2)
Guyana	Dieticians and nutritionists	3.2 (2.2 to 4.6)
Guyana	Environmental health workers	2.2 (1.5 to 3.1)
Guyana	Healthcare aides, paramedics, and ambulance workers	12.3 (8.8 to 17.3)
Guyana	Home-based personal care workers	10.5 (7.8 to 13.5)
Guyana	Medical imaging and therapeutic equipment technicians	2.3 (1.4 to 3.5)
Guyana	Medical laboratory technicians	10.4 (7.3 to 14.1)
Guyana	Optometrists and opticians	1.3 (0.9 to 1.8)
Guyana	Physiotherapists and prosthetic technicians	6.1 (4.3 to 8.4)
Guyana	Psychologists	2.7 (1.8 to 3.7)
Guyana	Traditional and complementary medicine practitioners	1.6 (1.1 to 2.4)
Haiti	Audiologists, speech therapists, and counsellors	0.3 (0.2 to 0.5)
Haiti	Clinical officers and medical assistants	0.3 (0.2 to 0.5)
Haiti	Dieticians and nutritionists	0.3 (0.2 to 0.4)

Haiti	Environmental health workers	0.7 (0.4 to 0.9)
Haiti	Healthcare aides, paramedics, and ambulance workers	2.4 (1.6 to 3.4)
Haiti	Home-based personal care workers	1.1 (0.8 to 1.5)
Haiti	Medical imaging and therapeutic equipment technicians	0.1 (0.1 to 0.2)
Haiti	Medical laboratory technicians	1.1 (0.7 to 1.7)
Haiti	Optometrists and opticians	0.1 (0.1 to 0.2)
Haiti	Physiotherapists and prosthetic technicians	0.7 (0.4 to 1.0)
Haiti	Psychologists	0.5 (0.3 to 0.7)
Haiti	Traditional and complementary medicine practitioners	1.0 (0.6 to 1.4)
Jamaica	Audiologists, speech therapists, and counsellors	1.4 (0.9 to 2.2)
Jamaica	Clinical officers and medical assistants	1.1 (0.7 to 1.8)
Jamaica	Dieticians and nutritionists	1.0 (0.6 to 1.4)
Jamaica	Environmental health workers	1.5 (1.0 to 2.3)
Jamaica	Healthcare aides, paramedics, and ambulance workers	9.0 (6.5 to 12.1)
Jamaica	Home-based personal care workers	16.9 (13.7 to 21.1)
Jamaica	Medical imaging and therapeutic equipment technicians	0.8 (0.5 to 1.3)
Jamaica	Medical laboratory technicians	2.2 (1.4 to 3.4)
Jamaica	Optometrists and opticians	0.8 (0.5 to 1.2)
Jamaica	Physiotherapists and prosthetic technicians	1.6 (1.1 to 2.4)
Jamaica	Psychologists	1.0 (0.6 to 1.4)
Jamaica	Traditional and complementary medicine practitioners	0.7 (0.4 to 1.1)
Puerto Rico	Audiologists, speech therapists, and counsellors	2.0 (1.2 to 3.1)
Puerto Rico	Clinical officers and medical assistants	0.8 (0.5 to 1.4)
Puerto Rico	Dieticians and nutritionists	1.6 (1.0 to 2.5)
Puerto Rico	Environmental health workers	1.4 (0.9 to 2.0)
Puerto Rico	Healthcare aides, paramedics, and ambulance workers	8.5 (5.8 to 12.6)
Puerto Rico	Home-based personal care workers	9.6 (7.0 to 12.8)
Puerto Rico	Medical imaging and therapeutic equipment technicians	1.3 (0.7 to 1.9)
Puerto Rico	Medical laboratory technicians	3.0 (1.8 to 4.7)
Puerto Rico	Optometrists and opticians	0.7 (0.5 to 1.1)

Puerto Rico	Physiotherapists and prosthetic technicians	2.7 (1.7 to 3.9)
Puerto Rico	Psychologists	2.4 (1.6 to 3.6)
Puerto Rico	Traditional and complementary medicine practitioners	0.7 (0.4 to 1.0)
Saint Kitts and Nevis	Audiologists, speech therapists, and counsellors	3.5 (2.2 to 5.4)
Saint Kitts and Nevis	Clinical officers and medical assistants	1.6 (0.9 to 2.6)
Saint Kitts and Nevis	Dieticians and nutritionists	4.1 (2.6 to 6.3)
Saint Kitts and Nevis	Environmental health workers	3.3 (2.1 to 4.8)
Saint Kitts and Nevis	Healthcare aides, paramedics, and ambulance workers	25.3 (17.9 to 36.6)
Saint Kitts and Nevis	Home-based personal care workers	14.0 (9.8 to 19.5)
Saint Kitts and Nevis	Medical imaging and therapeutic equipment technicians	2.3 (1.3 to 3.6)
Saint Kitts and Nevis	Medical laboratory technicians	8.9 (5.5 to 13.5)
Saint Kitts and Nevis	Optometrists and opticians	2.2 (1.4 to 3.2)
Saint Kitts and Nevis	Physiotherapists and prosthetic technicians	7.3 (4.8 to 10.6)
Saint Kitts and Nevis	Psychologists	8.4 (5.5 to 12.2)
Saint Kitts and Nevis	Traditional and complementary medicine practitioners	0.8 (0.5 to 1.3)
Saint Lucia	Audiologists, speech therapists, and counsellors	1.1 (0.7 to 1.7)
Saint Lucia	Clinical officers and medical assistants	0.6 (0.4 to 1.1)
Saint Lucia	Dieticians and nutritionists	1.1 (0.7 to 1.7)
Saint Lucia	Environmental health workers	1.3 (0.8 to 1.9)
Saint Lucia	Healthcare aides, paramedics, and ambulance workers	8.1 (5.7 to 11.7)
Saint Lucia	Home-based personal care workers	4.2 (2.9 to 5.9)
Saint Lucia	Medical imaging and therapeutic equipment technicians	0.7 (0.4 to 1.0)
Saint Lucia	Medical laboratory technicians	3.0 (1.8 to 4.5)
Saint Lucia	Optometrists and opticians	0.5 (0.4 to 0.8)
Saint Lucia	Physiotherapists and prosthetic technicians	2.2 (1.4 to 3.2)
Saint Lucia	Psychologists	2.2 (1.4 to 3.3)
Saint Lucia	Traditional and complementary medicine practitioners	0.5 (0.3 to 0.7)
Saint Vincent and the Grenadines	Audiologists, speech therapists, and counsellors	0.5 (0.3 to 0.7)
Saint Vincent and the Grenadines	Clinical officers and medical assistants	0.3 (0.2 to 0.5)
Saint Vincent and the Grenadines	Dieticians and nutritionists	0.4 (0.3 to 0.7)

Saint Vincent and the Grenadines	Environmental health workers	0.5 (0.3 to 0.8)
Saint Vincent and the Grenadines	Healthcare aides, paramedics, and ambulance workers	2.7 (1.9 to 4.0)
Saint Vincent and the Grenadines	Home-based personal care workers	1.8 (1.3 to 2.5)
Saint Vincent and the Grenadines	Medical imaging and therapeutic equipment technicians	0.3 (0.1 to 0.4)
Saint Vincent and the Grenadines	Medical laboratory technicians	1.2 (0.7 to 1.8)
Saint Vincent and the Grenadines	Optometrists and opticians	0.2 (0.1 to 0.3)
Saint Vincent and the Grenadines	Physiotherapists and prosthetic technicians	0.9 (0.6 to 1.3)
Saint Vincent and the Grenadines	Psychologists	0.8 (0.5 to 1.2)
Saint Vincent and the Grenadines	Traditional and complementary medicine practitioners	0.2 (0.2 to 0.4)
Suriname	Audiologists, speech therapists, and counsellors	1.9 (1.2 to 2.9)
Suriname	Clinical officers and medical assistants	1.8 (1.1 to 2.9)
Suriname	Dieticians and nutritionists	1.5 (0.9 to 2.3)
Suriname	Environmental health workers	2.1 (1.4 to 3.2)
Suriname	Healthcare aides, paramedics, and ambulance workers	14.5 (10.5 to 19.5)
Suriname	Home-based personal care workers	4.4 (3.1 to 6.1)
Suriname	Medical imaging and therapeutic equipment technicians	0.2 (0.1 to 0.3)
Suriname	Medical laboratory technicians	6.8 (4.4 to 10.1)
Suriname	Optometrists and opticians	0.7 (0.4 to 0.9)
Suriname	Physiotherapists and prosthetic technicians	3.0 (2.0 to 4.2)
Suriname	Psychologists	0.5 (0.3 to 0.8)
Suriname	Traditional and complementary medicine practitioners	0.7 (0.4 to 1.1)
Trinidad and Tobago	Audiologists, speech therapists, and counsellors	2.1 (1.2 to 3.2)
Trinidad and Tobago	Clinical officers and medical assistants	0.9 (0.5 to 1.3)
Trinidad and Tobago	Dieticians and nutritionists	2.8 (1.9 to 4.0)
Trinidad and Tobago	Environmental health workers	1.6 (1.0 to 2.3)
Trinidad and Tobago	Healthcare aides, paramedics, and ambulance workers	5.5 (3.8 to 8.0)
Trinidad and Tobago	Home-based personal care workers	7.6 (5.5 to 10.0)
Trinidad and Tobago	Medical imaging and therapeutic equipment technicians	0.9 (0.5 to 1.4)
Trinidad and Tobago	Medical laboratory technicians	2.5 (1.5 to 3.7)
Trinidad and Tobago	Optometrists and opticians	2.0 (1.3 to 2.8)

Trinidad and Tobago	Physiotherapists and prosthetic technicians	5.9 (4.1 to 8.5)
Trinidad and Tobago	Psychologists	2.1 (1.4 to 3.1)
Trinidad and Tobago	Traditional and complementary medicine practitioners	0.6 (0.4 to 0.9)
Virgin Islands	Audiologists, speech therapists, and counsellors	4.0 (2.5 to 6.2)
Virgin Islands	Clinical officers and medical assistants	1.5 (0.9 to 2.6)
Virgin Islands	Dieticians and nutritionists	4.6 (2.9 to 6.9)
Virgin Islands	Environmental health workers	3.0 (1.9 to 4.4)
Virgin Islands	Healthcare aides, paramedics, and ambulance workers	23.6 (16.4 to 34.4)
Virgin Islands	Home-based personal care workers	18.2 (12.9 to 24.9)
Virgin Islands	Medical imaging and therapeutic equipment technicians	2.7 (1.6 to 4.2)
Virgin Islands	Medical laboratory technicians	8.5 (5.3 to 13.0)
Virgin Islands	Optometrists and opticians	2.5 (1.7 to 3.7)
Virgin Islands	Physiotherapists and prosthetic technicians	7.8 (5.1 to 11.3)
Virgin Islands	Psychologists	8.4 (5.6 to 12.4)
Virgin Islands	Traditional and complementary medicine practitioners	0.8 (0.5 to 1.3)
Central Latin America	Audiologists, speech therapists, and counsellors	2.4 (1.6 to 3.5)
Central Latin America	Clinical officers and medical assistants	2.5 (1.8 to 3.2)
Central Latin America	Dieticians and nutritionists	1.8 (1.4 to 2.2)
Central Latin America	Environmental health workers	5.0 (3.8 to 6.2)
Central Latin America	Healthcare aides, paramedics, and ambulance workers	9.7 (7.4 to 13.0)
Central Latin America	Home-based personal care workers	3.6 (2.6 to 4.8)
Central Latin America	Medical imaging and therapeutic equipment technicians	4.8 (3.1 to 7.0)
Central Latin America	Medical laboratory technicians	4.7 (3.7 to 5.9)
Central Latin America	Optometrists and opticians	1.8 (1.3 to 2.4)
Central Latin America	Physiotherapists and prosthetic technicians	5.2 (4.2 to 6.6)
Central Latin America	Psychologists	6.9 (5.5 to 8.9)
Central Latin America	Traditional and complementary medicine practitioners	1.4 (1.1 to 1.9)
Colombia	Audiologists, speech therapists, and counsellors	1.4 (0.9 to 2.2)
Colombia	Clinical officers and medical assistants	2.1 (1.3 to 3.4)
Colombia	Dieticians and nutritionists	1.4 (0.9 to 2.1)

Colombia	Environmental health workers	2.2 (1.5 to 3.3)
Colombia	Healthcare aides, paramedics, and ambulance workers	8.1 (5.4 to 12.1)
Colombia	Home-based personal care workers	4.6 (3.3 to 6.4)
Colombia	Medical imaging and therapeutic equipment technicians	3.1 (1.8 to 4.7)
Colombia	Medical laboratory technicians	5.2 (3.3 to 7.7)
Colombia	Optometrists and opticians	1.4 (0.9 to 2.0)
Colombia	Physiotherapists and prosthetic technicians	4.2 (2.7 to 5.8)
Colombia	Psychologists	6.3 (4.4 to 9.0)
Colombia	Traditional and complementary medicine practitioners	0.7 (0.4 to 1.0)
Costa Rica	Audiologists, speech therapists, and counsellors	6.3 (4.0 to 9.4)
Costa Rica	Clinical officers and medical assistants	4.6 (2.8 to 7.1)
Costa Rica	Dieticians and nutritionists	3.7 (2.5 to 5.2)
Costa Rica	Environmental health workers	3.5 (2.3 to 5.0)
Costa Rica	Healthcare aides, paramedics, and ambulance workers	20.4 (13.1 to 29.2)
Costa Rica	Home-based personal care workers	5.7 (4.0 to 7.9)
Costa Rica	Medical imaging and therapeutic equipment technicians	2.2 (1.3 to 3.3)
Costa Rica	Medical laboratory technicians	11.2 (7.1 to 16.4)
Costa Rica	Optometrists and opticians	3.2 (2.2 to 4.3)
Costa Rica	Physiotherapists and prosthetic technicians	31.8 (22.2 to 45.4)
Costa Rica	Psychologists	14.3 (9.7 to 20.0)
Costa Rica	Traditional and complementary medicine practitioners	0.6 (0.4 to 0.9)
El Salvador	Audiologists, speech therapists, and counsellors	0.8 (0.6 to 1.2)
El Salvador	Clinical officers and medical assistants	1.3 (0.9 to 1.8)
El Salvador	Dieticians and nutritionists	1.3 (0.9 to 1.8)
El Salvador	Environmental health workers	2.6 (1.8 to 3.5)
El Salvador	Healthcare aides, paramedics, and ambulance workers	3.3 (2.4 to 4.5)
El Salvador	Home-based personal care workers	8.1 (6.4 to 9.9)
El Salvador	Medical imaging and therapeutic equipment technicians	1.0 (0.6 to 1.5)
El Salvador	Medical laboratory technicians	3.6 (2.5 to 4.9)
El Salvador	Optometrists and opticians	1.6 (1.2 to 2.0)

El Salvador	Physiotherapists and prosthetic technicians	3.3 (2.4 to 4.3)
El Salvador	Psychologists	5.3 (4.1 to 6.8)
El Salvador	Traditional and complementary medicine practitioners	0.5 (0.3 to 0.7)
Guatemala	Audiologists, speech therapists, and counsellors	0.6 (0.4 to 1.0)
Guatemala	Clinical officers and medical assistants	0.8 (0.5 to 1.3)
Guatemala	Dieticians and nutritionists	0.5 (0.3 to 0.8)
Guatemala	Environmental health workers	1.4 (0.9 to 1.9)
Guatemala	Healthcare aides, paramedics, and ambulance workers	3.7 (2.6 to 5.5)
Guatemala	Home-based personal care workers	1.9 (1.3 to 2.6)
Guatemala	Medical imaging and therapeutic equipment technicians	0.8 (0.5 to 1.2)
Guatemala	Medical laboratory technicians	2.0 (1.2 to 3.0)
Guatemala	Optometrists and opticians	0.5 (0.3 to 0.7)
Guatemala	Physiotherapists and prosthetic technicians	1.4 (0.9 to 1.9)
Guatemala	Psychologists	2.3 (1.6 to 3.3)
Guatemala	Traditional and complementary medicine practitioners	0.6 (0.4 to 0.9)
Honduras	Audiologists, speech therapists, and counsellors	0.6 (0.4 to 1.0)
Honduras	Clinical officers and medical assistants	0.4 (0.3 to 0.7)
Honduras	Dieticians and nutritionists	0.5 (0.4 to 0.8)
Honduras	Environmental health workers	0.5 (0.3 to 0.7)
Honduras	Healthcare aides, paramedics, and ambulance workers	5.1 (3.3 to 7.6)
Honduras	Home-based personal care workers	2.3 (1.6 to 3.2)
Honduras	Medical imaging and therapeutic equipment technicians	1.4 (0.9 to 2.1)
Honduras	Medical laboratory technicians	2.1 (1.3 to 3.2)
Honduras	Optometrists and opticians	0.5 (0.4 to 0.8)
Honduras	Physiotherapists and prosthetic technicians	2.0 (1.4 to 2.8)
Honduras	Psychologists	1.4 (0.9 to 2.0)
Honduras	Traditional and complementary medicine practitioners	0.8 (0.5 to 1.1)
Mexico	Audiologists, speech therapists, and counsellors	3.0 (2.0 to 4.3)
Mexico	Clinical officers and medical assistants	3.3 (2.4 to 4.3)
Mexico	Dieticians and nutritionists	2.5 (1.9 to 3.1)

Mexico	Environmental health workers	8.0 (6.2 to 9.9)
Mexico	Healthcare aides, paramedics, and ambulance workers	11.9 (8.2 to 16.4)
Mexico	Home-based personal care workers	3.8 (2.7 to 5.1)
Mexico	Medical imaging and therapeutic equipment technicians	7.6 (4.9 to 11.4)
Mexico	Medical laboratory technicians	5.3 (3.5 to 7.5)
Mexico	Optometrists and opticians	2.5 (1.8 to 3.3)
Mexico	Physiotherapists and prosthetic technicians	6.3 (4.9 to 8.0)
Mexico	Psychologists	9.4 (7.5 to 11.8)
Mexico	Traditional and complementary medicine practitioners	2.3 (1.8 to 3.1)
Nicaragua	Audiologists, speech therapists, and counsellors	0.4 (0.3 to 0.7)
Nicaragua	Clinical officers and medical assistants	0.8 (0.5 to 1.2)
Nicaragua	Dieticians and nutritionists	0.4 (0.2 to 0.6)
Nicaragua	Environmental health workers	0.9 (0.6 to 1.3)
Nicaragua	Healthcare aides, paramedics, and ambulance workers	5.7 (3.8 to 7.6)
Nicaragua	Home-based personal care workers	1.7 (1.1 to 2.3)
Nicaragua	Medical imaging and therapeutic equipment technicians	1.0 (0.6 to 1.6)
Nicaragua	Medical laboratory technicians	3.2 (2.1 to 4.7)
Nicaragua	Optometrists and opticians	0.4 (0.3 to 0.6)
Nicaragua	Physiotherapists and prosthetic technicians	1.5 (1.0 to 2.2)
Nicaragua	Psychologists	2.4 (1.7 to 3.4)
Nicaragua	Traditional and complementary medicine practitioners	0.3 (0.2 to 0.4)
Panama	Audiologists, speech therapists, and counsellors	3.1 (2.0 to 4.8)
Panama	Clinical officers and medical assistants	3.7 (2.2 to 5.7)
Panama	Dieticians and nutritionists	2.7 (1.9 to 4.0)
Panama	Environmental health workers	3.9 (2.5 to 5.5)
Panama	Healthcare aides, paramedics, and ambulance workers	19.1 (11.3 to 29.6)
Panama	Home-based personal care workers	8.6 (6.0 to 11.8)
Panama	Medical imaging and therapeutic equipment technicians	5.7 (3.6 to 8.4)
Panama	Medical laboratory technicians	6.4 (3.9 to 9.6)
Panama	Optometrists and opticians	3.5 (2.4 to 4.8)

Panama	Physiotherapists and prosthetic technicians	9.1 (6.2 to 13.1)
Panama	Psychologists	8.1 (5.4 to 11.2)
Panama	Traditional and complementary medicine practitioners	0.9 (0.6 to 1.3)
Venezuela	Audiologists, speech therapists, and counsellors	3.0 (1.9 to 4.4)
Venezuela	Clinical officers and medical assistants	1.1 (0.7 to 1.8)
Venezuela	Dieticians and nutritionists	0.5 (0.3 to 0.7)
Venezuela	Environmental health workers	1.4 (0.9 to 2.0)
Venezuela	Healthcare aides, paramedics, and ambulance workers	6.8 (4.9 to 9.2)
Venezuela	Home-based personal care workers	1.3 (0.9 to 1.8)
Venezuela	Medical imaging and therapeutic equipment technicians	0.7 (0.4 to 1.2)
Venezuela	Medical laboratory technicians	3.0 (1.9 to 4.6)
Venezuela	Optometrists and opticians	0.4 (0.3 to 0.6)
Venezuela	Physiotherapists and prosthetic technicians	1.7 (1.1 to 2.5)
Venezuela	Psychologists	1.7 (1.1 to 2.5)
Venezuela	Traditional and complementary medicine practitioners	0.1 (0.1 to 0.1)
Tropical Latin America	Audiologists, speech therapists, and counsellors	3.8 (3.0 to 5.0)
Tropical Latin America	Clinical officers and medical assistants	1.2 (0.9 to 1.5)
Tropical Latin America	Dieticians and nutritionists	4.6 (3.6 to 6.0)
Tropical Latin America	Environmental health workers	21.9 (16.6 to 27.4)
Tropical Latin America	Healthcare aides, paramedics, and ambulance workers	17.6 (11.3 to 26.1)
Tropical Latin America	Home-based personal care workers	0.7 (0.5 to 0.9)
Tropical Latin America	Medical imaging and therapeutic equipment technicians	0.3 (0.2 to 0.4)
Tropical Latin America	Medical laboratory technicians	4.4 (3.2 to 5.9)
Tropical Latin America	Optometrists and opticians	0.3 (0.2 to 0.4)
Tropical Latin America	Physiotherapists and prosthetic technicians	15.0 (12.6 to 18.3)
Tropical Latin America	Psychologists	10.6 (8.5 to 12.6)
Tropical Latin America	Traditional and complementary medicine practitioners	0.9 (0.6 to 1.2)
Brazil	Audiologists, speech therapists, and counsellors	3.9 (3.0 to 5.1)
Brazil	Clinical officers and medical assistants	1.2 (0.9 to 1.5)
Brazil	Dieticians and nutritionists	4.7 (3.6 to 6.2)

Brazil	Environmental health workers	22.5 (17.0 to 28.2)
Brazil	Healthcare aides, paramedics, and ambulance workers	17.7 (11.4 to 26.2)
Brazil	Home-based personal care workers	0.5 (0.4 to 0.7)
Brazil	Medical imaging and therapeutic equipment technicians	0.2 (0.1 to 0.3)
Brazil	Medical laboratory technicians	4.5 (3.2 to 6.0)
Brazil	Optometrists and opticians	0.3 (0.2 to 0.4)
Brazil	Physiotherapists and prosthetic technicians	15.2 (12.8 to 18.6)
Brazil	Psychologists	10.7 (8.6 to 12.8)
Brazil	Traditional and complementary medicine practitioners	0.8 (0.6 to 1.1)
Paraguay	Audiologists, speech therapists, and counsellors	1.1 (0.6 to 1.6)
Paraguay	Clinical officers and medical assistants	0.8 (0.5 to 1.2)
Paraguay	Dieticians and nutritionists	1.3 (0.9 to 1.9)
Paraguay	Environmental health workers	1.0 (0.7 to 1.4)
Paraguay	Healthcare aides, paramedics, and ambulance workers	14.9 (9.7 to 22.2)
Paraguay	Home-based personal care workers	4.5 (3.2 to 6.0)
Paraguay	Medical imaging and therapeutic equipment technicians	3.0 (1.9 to 4.4)
Paraguay	Medical laboratory technicians	1.2 (0.7 to 1.8)
Paraguay	Optometrists and opticians	1.4 (1.0 to 1.9)
Paraguay	Physiotherapists and prosthetic technicians	8.0 (5.7 to 11.2)
Paraguay	Psychologists	7.4 (5.0 to 10.3)
Paraguay	Traditional and complementary medicine practitioners	2.2 (1.4 to 3.3)
**North Africa and Middle East**	Audiologists, speech therapists, and counsellors	0.5 (0.3 to 0.6)
**North Africa and Middle East**	Clinical officers and medical assistants	2.7 (2.3 to 3.3)
**North Africa and Middle East**	Dieticians and nutritionists	2.0 (1.7 to 2.6)
**North Africa and Middle East**	Environmental health workers	1.6 (1.3 to 1.9)
**North Africa and Middle East**	Healthcare aides, paramedics, and ambulance workers	3.9 (2.8 to 5.3)
**North Africa and Middle East**	Home-based personal care workers	1.8 (1.3 to 2.5)
**North Africa and Middle East**	Medical imaging and therapeutic equipment technicians	2.3 (1.8 to 3.1)
**North Africa and Middle East**	Medical laboratory technicians	6.5 (4.7 to 9.0)
**North Africa and Middle East**	Optometrists and opticians	0.5 (0.4 to 0.7)

**North Africa and Middle East**	Physiotherapists and prosthetic technicians	1.1 (0.8 to 1.5)
**North Africa and Middle East**	Psychologists	2.6 (1.9 to 3.7)
**North Africa and Middle East**	Traditional and complementary medicine practitioners	2.5 (1.8 to 3.4)
North Africa and Middle East	Audiologists, speech therapists, and counsellors	0.5 (0.3 to 0.6)
North Africa and Middle East	Clinical officers and medical assistants	2.7 (2.3 to 3.3)
North Africa and Middle East	Dieticians and nutritionists	2.0 (1.7 to 2.6)
North Africa and Middle East	Environmental health workers	1.6 (1.3 to 1.9)
North Africa and Middle East	Healthcare aides, paramedics, and ambulance workers	3.9 (2.8 to 5.3)
North Africa and Middle East	Home-based personal care workers	1.8 (1.3 to 2.5)
North Africa and Middle East	Medical imaging and therapeutic equipment technicians	2.3 (1.8 to 3.1)
North Africa and Middle East	Medical laboratory technicians	6.5 (4.7 to 9.0)
North Africa and Middle East	Optometrists and opticians	0.5 (0.4 to 0.7)
North Africa and Middle East	Physiotherapists and prosthetic technicians	1.1 (0.8 to 1.5)
North Africa and Middle East	Psychologists	2.6 (1.9 to 3.7)
North Africa and Middle East	Traditional and complementary medicine practitioners	2.5 (1.8 to 3.4)
Afghanistan	Audiologists, speech therapists, and counsellors	0.0 (0.0 to 0.0)
Afghanistan	Clinical officers and medical assistants	0.3 (0.2 to 0.4)
Afghanistan	Dieticians and nutritionists	0.0 (0.0 to 0.1)
Afghanistan	Environmental health workers	0.2 (0.1 to 0.3)
Afghanistan	Healthcare aides, paramedics, and ambulance workers	0.2 (0.1 to 0.3)
Afghanistan	Home-based personal care workers	0.0 (0.0 to 0.1)
Afghanistan	Medical imaging and therapeutic equipment technicians	0.0 (0.0 to 0.1)
Afghanistan	Medical laboratory technicians	0.2 (0.1 to 0.3)
Afghanistan	Optometrists and opticians	0.0 (0.0 to 0.0)
Afghanistan	Physiotherapists and prosthetic technicians	0.0 (0.0 to 0.1)
Afghanistan	Psychologists	0.0 (0.0 to 0.1)
Afghanistan	Traditional and complementary medicine practitioners	0.9 (0.7 to 1.2)
Algeria	Audiologists, speech therapists, and counsellors	0.1 (0.1 to 0.2)
Algeria	Clinical officers and medical assistants	0.5 (0.3 to 0.7)
Algeria	Dieticians and nutritionists	0.3 (0.2 to 0.4)

Algeria	Environmental health workers	0.3 (0.2 to 0.5)
Algeria	Healthcare aides, paramedics, and ambulance workers	0.8 (0.5 to 1.3)
Algeria	Home-based personal care workers	0.4 (0.3 to 0.6)
Algeria	Medical imaging and therapeutic equipment technicians	0.8 (0.4 to 1.2)
Algeria	Medical laboratory technicians	1.5 (0.9 to 2.3)
Algeria	Optometrists and opticians	0.1 (0.0 to 0.1)
Algeria	Physiotherapists and prosthetic technicians	0.2 (0.1 to 0.3)
Algeria	Psychologists	0.3 (0.2 to 0.4)
Algeria	Traditional and complementary medicine practitioners	4.2 (3.0 to 5.6)
Bahrain	Audiologists, speech therapists, and counsellors	1.9 (1.2 to 3.0)
Bahrain	Clinical officers and medical assistants	10.4 (6.4 to 16.7)
Bahrain	Dieticians and nutritionists	11.0 (7.1 to 16.3)
Bahrain	Environmental health workers	8.2 (5.4 to 11.8)
Bahrain	Healthcare aides, paramedics, and ambulance workers	25.8 (17.1 to 37.2)
Bahrain	Home-based personal care workers	7.6 (5.3 to 10.8)
Bahrain	Medical imaging and therapeutic equipment technicians	11.9 (7.2 to 18.3)
Bahrain	Medical laboratory technicians	18.8 (11.8 to 28.0)
Bahrain	Optometrists and opticians	2.8 (1.9 to 4.1)
Bahrain	Physiotherapists and prosthetic technicians	5.7 (3.7 to 8.2)
Bahrain	Psychologists	15.5 (10.3 to 22.4)
Bahrain	Traditional and complementary medicine practitioners	5.8 (3.7 to 8.4)
Egypt	Audiologists, speech therapists, and counsellors	0.2 (0.1 to 0.3)
Egypt	Clinical officers and medical assistants	5.9 (4.4 to 7.5)
Egypt	Dieticians and nutritionists	3.4 (2.5 to 4.5)
Egypt	Environmental health workers	1.8 (1.3 to 2.3)
Egypt	Healthcare aides, paramedics, and ambulance workers	2.0 (1.4 to 2.7)
Egypt	Home-based personal care workers	0.6 (0.4 to 0.7)
Egypt	Medical imaging and therapeutic equipment technicians	2.1 (1.5 to 2.8)
Egypt	Medical laboratory technicians	18.8 (13.5 to 25.3)
Egypt	Optometrists and opticians	0.3 (0.2 to 0.4)

Egypt	Physiotherapists and prosthetic technicians	0.4 (0.3 to 0.5)
Egypt	Psychologists	1.4 (1.0 to 1.8)
Egypt	Traditional and complementary medicine practitioners	3.4 (2.2 to 5.1)
Iran	Audiologists, speech therapists, and counsellors	0.3 (0.2 to 0.4)
Iran	Clinical officers and medical assistants	1.5 (1.0 to 2.4)
Iran	Dieticians and nutritionists	1.0 (0.7 to 1.3)
Iran	Environmental health workers	1.1 (0.7 to 1.6)
Iran	Healthcare aides, paramedics, and ambulance workers	1.1 (0.8 to 1.6)
Iran	Home-based personal care workers	0.8 (0.5 to 1.1)
Iran	Medical imaging and therapeutic equipment technicians	1.7 (1.1 to 2.4)
Iran	Medical laboratory technicians	1.2 (0.7 to 1.8)
Iran	Optometrists and opticians	0.3 (0.2 to 0.4)
Iran	Physiotherapists and prosthetic technicians	0.6 (0.4 to 0.9)
Iran	Psychologists	0.3 (0.2 to 0.5)
Iran	Traditional and complementary medicine practitioners	1.9 (1.3 to 2.8)
Iraq	Audiologists, speech therapists, and counsellors	0.3 (0.2 to 0.5)
Iraq	Clinical officers and medical assistants	2.2 (1.5 to 3.1)
Iraq	Dieticians and nutritionists	0.2 (0.1 to 0.3)
Iraq	Environmental health workers	0.8 (0.5 to 1.2)
Iraq	Healthcare aides, paramedics, and ambulance workers	2.9 (2.1 to 3.9)
Iraq	Home-based personal care workers	0.9 (0.6 to 1.2)
Iraq	Medical imaging and therapeutic equipment technicians	0.3 (0.2 to 0.5)
Iraq	Medical laboratory technicians	0.6 (0.3 to 0.9)
Iraq	Optometrists and opticians	0.2 (0.1 to 0.2)
Iraq	Physiotherapists and prosthetic technicians	0.3 (0.2 to 0.4)
Iraq	Psychologists	0.2 (0.1 to 0.3)
Iraq	Traditional and complementary medicine practitioners	0.5 (0.3 to 0.8)
Jordan	Audiologists, speech therapists, and counsellors	0.2 (0.1 to 0.3)
Jordan	Clinical officers and medical assistants	2.4 (1.5 to 3.7)
Jordan	Dieticians and nutritionists	1.1 (0.7 to 1.6)

Jordan	Environmental health workers	7.9 (5.3 to 11.1)
Jordan	Healthcare aides, paramedics, and ambulance workers	4.2 (2.9 to 6.2)
Jordan	Home-based personal care workers	4.3 (3.1 to 5.8)
Jordan	Medical imaging and therapeutic equipment technicians	2.0 (1.3 to 3.0)
Jordan	Medical laboratory technicians	2.4 (1.5 to 3.5)
Jordan	Optometrists and opticians	0.2 (0.1 to 0.3)
Jordan	Physiotherapists and prosthetic technicians	2.6 (1.8 to 3.6)
Jordan	Psychologists	0.9 (0.6 to 1.3)
Jordan	Traditional and complementary medicine practitioners	3.6 (2.4 to 5.4)
Kuwait	Audiologists, speech therapists, and counsellors	2.6 (1.6 to 4.1)
Kuwait	Clinical officers and medical assistants	11.0 (6.7 to 17.8)
Kuwait	Dieticians and nutritionists	17.4 (11.4 to 25.7)
Kuwait	Environmental health workers	8.7 (5.6 to 12.5)
Kuwait	Healthcare aides, paramedics, and ambulance workers	30.1 (19.7 to 44.4)
Kuwait	Home-based personal care workers	12.1 (8.4 to 16.9)
Kuwait	Medical imaging and therapeutic equipment technicians	18.0 (10.9 to 27.2)
Kuwait	Medical laboratory technicians	25.1 (15.8 to 37.2)
Kuwait	Optometrists and opticians	5.5 (3.7 to 7.8)
Kuwait	Physiotherapists and prosthetic technicians	8.7 (5.7 to 12.4)
Kuwait	Psychologists	24.1 (16.3 to 34.4)
Kuwait	Traditional and complementary medicine practitioners	5.5 (3.5 to 8.0)
Lebanon	Audiologists, speech therapists, and counsellors	0.2 (0.1 to 0.3)
Lebanon	Clinical officers and medical assistants	1.5 (0.9 to 2.4)
Lebanon	Dieticians and nutritionists	0.9 (0.6 to 1.4)
Lebanon	Environmental health workers	0.9 (0.6 to 1.4)
Lebanon	Healthcare aides, paramedics, and ambulance workers	1.9 (1.2 to 2.8)
Lebanon	Home-based personal care workers	0.9 (0.6 to 1.3)
Lebanon	Medical imaging and therapeutic equipment technicians	1.2 (0.7 to 1.8)
Lebanon	Medical laboratory technicians	1.8 (1.2 to 2.7)
Lebanon	Optometrists and opticians	0.2 (0.1 to 0.3)

Lebanon	Physiotherapists and prosthetic technicians	0.5 (0.3 to 0.7)
Lebanon	Psychologists	1.1 (0.7 to 1.6)
Lebanon	Traditional and complementary medicine practitioners	1.5 (1.0 to 2.1)
Libya	Audiologists, speech therapists, and counsellors	0.2 (0.1 to 0.3)
Libya	Clinical officers and medical assistants	1.2 (0.7 to 1.9)
Libya	Dieticians and nutritionists	0.9 (0.6 to 1.4)
Libya	Environmental health workers	0.7 (0.4 to 1.0)
Libya	Healthcare aides, paramedics, and ambulance workers	1.3 (0.8 to 1.9)
Libya	Home-based personal care workers	1.0 (0.7 to 1.4)
Libya	Medical imaging and therapeutic equipment technicians	1.2 (0.7 to 1.8)
Libya	Medical laboratory technicians	1.7 (1.1 to 2.5)
Libya	Optometrists and opticians	0.3 (0.2 to 0.4)
Libya	Physiotherapists and prosthetic technicians	0.5 (0.3 to 0.7)
Libya	Psychologists	1.1 (0.7 to 1.6)
Libya	Traditional and complementary medicine practitioners	1.2 (0.8 to 1.6)
Morocco	Audiologists, speech therapists, and counsellors	0.1 (0.1 to 0.2)
Morocco	Clinical officers and medical assistants	0.8 (0.5 to 1.2)
Morocco	Dieticians and nutritionists	0.4 (0.3 to 0.7)
Morocco	Environmental health workers	0.3 (0.2 to 0.5)
Morocco	Healthcare aides, paramedics, and ambulance workers	0.3 (0.2 to 0.5)
Morocco	Home-based personal care workers	0.5 (0.3 to 0.7)
Morocco	Medical imaging and therapeutic equipment technicians	0.5 (0.3 to 0.8)
Morocco	Medical laboratory technicians	1.2 (0.7 to 1.7)
Morocco	Optometrists and opticians	0.2 (0.1 to 0.3)
Morocco	Physiotherapists and prosthetic technicians	0.3 (0.2 to 0.4)
Morocco	Psychologists	0.5 (0.3 to 0.8)
Morocco	Traditional and complementary medicine practitioners	1.7 (1.2 to 2.3)
Oman	Audiologists, speech therapists, and counsellors	0.7 (0.4 to 1.0)
Oman	Clinical officers and medical assistants	3.4 (2.1 to 5.4)
Oman	Dieticians and nutritionists	3.7 (2.4 to 5.5)

Oman	Environmental health workers	2.3 (1.5 to 3.3)
Oman	Healthcare aides, paramedics, and ambulance workers	5.7 (3.7 to 8.5)
Oman	Home-based personal care workers	2.9 (2.0 to 4.0)
Oman	Medical imaging and therapeutic equipment technicians	4.0 (2.4 to 6.1)
Oman	Medical laboratory technicians	6.4 (4.0 to 9.4)
Oman	Optometrists and opticians	1.1 (0.8 to 1.7)
Oman	Physiotherapists and prosthetic technicians	1.9 (1.2 to 2.7)
Oman	Psychologists	5.1 (3.4 to 7.4)
Oman	Traditional and complementary medicine practitioners	2.3 (1.5 to 3.3)
Palestine	Audiologists, speech therapists, and counsellors	0.0 (0.0 to 0.0)
Palestine	Clinical officers and medical assistants	0.1 (0.0 to 0.2)
Palestine	Dieticians and nutritionists	0.0 (0.0 to 0.1)
Palestine	Environmental health workers	0.1 (0.0 to 0.1)
Palestine	Healthcare aides, paramedics, and ambulance workers	0.2 (0.1 to 0.3)
Palestine	Home-based personal care workers	0.1 (0.1 to 0.2)
Palestine	Medical imaging and therapeutic equipment technicians	0.1 (0.0 to 0.1)
Palestine	Medical laboratory technicians	0.1 (0.1 to 0.2)
Palestine	Optometrists and opticians	0.0 (0.0 to 0.0)
Palestine	Physiotherapists and prosthetic technicians	0.1 (0.0 to 0.1)
Palestine	Psychologists	0.2 (0.1 to 0.3)
Palestine	Traditional and complementary medicine practitioners	3.4 (2.9 to 4.0)
Qatar	Audiologists, speech therapists, and counsellors	3.2 (1.9 to 5.0)
Qatar	Clinical officers and medical assistants	13.9 (8.3 to 22.5)
Qatar	Dieticians and nutritionists	24.7 (16.2 to 36.5)
Qatar	Environmental health workers	13.9 (9.0 to 20.1)
Qatar	Healthcare aides, paramedics, and ambulance workers	65.0 (44.7 to 92.4)
Qatar	Home-based personal care workers	13.1 (8.9 to 18.5)
Qatar	Medical imaging and therapeutic equipment technicians	22.8 (13.9 to 34.7)
Qatar	Medical laboratory technicians	36.4 (22.6 to 54.1)
Qatar	Optometrists and opticians	7.1 (4.7 to 10.3)

Qatar	Physiotherapists and prosthetic technicians	12.9 (8.2 to 18.5)
Qatar	Psychologists	38.0 (25.5 to 54.0)
Qatar	Traditional and complementary medicine practitioners	5.0 (3.2 to 7.4)
Saudi Arabia	Audiologists, speech therapists, and counsellors	1.4 (0.8 to 2.1)
Saudi Arabia	Clinical officers and medical assistants	6.4 (3.9 to 10.2)
Saudi Arabia	Dieticians and nutritionists	7.6 (5.0 to 11.3)
Saudi Arabia	Environmental health workers	4.2 (2.7 to 6.1)
Saudi Arabia	Healthcare aides, paramedics, and ambulance workers	10.7 (6.8 to 16.0)
Saudi Arabia	Home-based personal care workers	6.6 (4.6 to 9.2)
Saudi Arabia	Medical imaging and therapeutic equipment technicians	8.6 (5.2 to 13.1)
Saudi Arabia	Medical laboratory technicians	11.9 (7.5 to 17.7)
Saudi Arabia	Optometrists and opticians	2.4 (1.6 to 3.5)
Saudi Arabia	Physiotherapists and prosthetic technicians	3.9 (2.5 to 5.5)
Saudi Arabia	Psychologists	9.6 (6.4 to 13.7)
Saudi Arabia	Traditional and complementary medicine practitioners	4.6 (3.0 to 6.7)
Sudan	Audiologists, speech therapists, and counsellors	0.0 (0.0 to 0.0)
Sudan	Clinical officers and medical assistants	0.2 (0.1 to 0.4)
Sudan	Dieticians and nutritionists	0.1 (0.1 to 0.1)
Sudan	Environmental health workers	0.1 (0.1 to 0.2)
Sudan	Healthcare aides, paramedics, and ambulance workers	0.2 (0.1 to 0.2)
Sudan	Home-based personal care workers	0.1 (0.1 to 0.1)
Sudan	Medical imaging and therapeutic equipment technicians	0.1 (0.1 to 0.2)
Sudan	Medical laboratory technicians	0.2 (0.1 to 0.3)
Sudan	Optometrists and opticians	0.0 (0.0 to 0.0)
Sudan	Physiotherapists and prosthetic technicians	0.1 (0.0 to 0.1)
Sudan	Psychologists	0.1 (0.1 to 0.1)
Sudan	Traditional and complementary medicine practitioners	0.5 (0.3 to 0.7)
Syria	Audiologists, speech therapists, and counsellors	0.1 (0.1 to 0.2)
Syria	Clinical officers and medical assistants	1.2 (0.8 to 2.0)
Syria	Dieticians and nutritionists	0.4 (0.3 to 0.6)

Syria	Environmental health workers	0.8 (0.5 to 1.2)
Syria	Healthcare aides, paramedics, and ambulance workers	1.3 (0.8 to 1.9)
Syria	Home-based personal care workers	0.5 (0.3 to 0.7)
Syria	Medical imaging and therapeutic equipment technicians	0.6 (0.3 to 0.9)
Syria	Medical laboratory technicians	1.1 (0.7 to 1.6)
Syria	Optometrists and opticians	0.1 (0.1 to 0.1)
Syria	Physiotherapists and prosthetic technicians	0.3 (0.2 to 0.4)
Syria	Psychologists	0.4 (0.3 to 0.7)
Syria	Traditional and complementary medicine practitioners	1.9 (1.3 to 2.6)
Tunisia	Audiologists, speech therapists, and counsellors	0.1 (0.0 to 0.1)
Tunisia	Clinical officers and medical assistants	0.5 (0.3 to 0.8)
Tunisia	Dieticians and nutritionists	0.3 (0.2 to 0.5)
Tunisia	Environmental health workers	0.3 (0.2 to 0.4)
Tunisia	Healthcare aides, paramedics, and ambulance workers	0.3 (0.2 to 0.5)
Tunisia	Home-based personal care workers	0.4 (0.3 to 0.6)
Tunisia	Medical imaging and therapeutic equipment technicians	0.4 (0.2 to 0.6)
Tunisia	Medical laboratory technicians	0.8 (0.5 to 1.2)
Tunisia	Optometrists and opticians	0.1 (0.1 to 0.2)
Tunisia	Physiotherapists and prosthetic technicians	0.2 (0.1 to 0.3)
Tunisia	Psychologists	0.4 (0.3 to 0.6)
Tunisia	Traditional and complementary medicine practitioners	0.9 (0.6 to 1.2)
Türkiye	Audiologists, speech therapists, and counsellors	1.3 (0.8 to 1.9)
Türkiye	Clinical officers and medical assistants	2.1 (1.3 to 3.1)
Türkiye	Dieticians and nutritionists	0.8 (0.5 to 1.1)
Türkiye	Environmental health workers	0.9 (0.6 to 1.2)
Türkiye	Healthcare aides, paramedics, and ambulance workers	3.8 (2.4 to 5.5)
Türkiye	Home-based personal care workers	4.4 (3.1 to 5.9)
Türkiye	Medical imaging and therapeutic equipment technicians	3.0 (1.8 to 4.5)
Türkiye	Medical laboratory technicians	8.5 (5.4 to 12.7)
Türkiye	Optometrists and opticians	0.4 (0.3 to 0.5)

Türkiye	Physiotherapists and prosthetic technicians	2.1 (1.5 to 3.0)
Türkiye	Psychologists	4.7 (3.2 to 6.6)
Türkiye	Traditional and complementary medicine practitioners	3.2 (2.1 to 4.8)
United Arab Emirates	Audiologists, speech therapists, and counsellors	3.6 (2.2 to 5.7)
United Arab Emirates	Clinical officers and medical assistants	16.1 (9.7 to 26.2)
United Arab Emirates	Dieticians and nutritionists	27.4 (17.9 to 40.5)
United Arab Emirates	Environmental health workers	16.3 (10.6 to 23.6)
United Arab Emirates	Healthcare aides, paramedics, and ambulance workers	77.0 (53.3 to 108.8)
United Arab Emirates	Home-based personal care workers	14.2 (9.6 to 20.2)
United Arab Emirates	Medical imaging and therapeutic equipment technicians	25.5 (15.4 to 38.8)
United Arab Emirates	Medical laboratory technicians	40.9 (25.4 to 60.8)
United Arab Emirates	Optometrists and opticians	7.5 (4.9 to 10.9)
United Arab Emirates	Physiotherapists and prosthetic technicians	14.1 (9.0 to 20.3)
United Arab Emirates	Psychologists	42.7 (28.7 to 60.9)
United Arab Emirates	Traditional and complementary medicine practitioners	5.7 (3.5 to 8.4)
Yemen	Audiologists, speech therapists, and counsellors	0.0 (0.0 to 0.1)
Yemen	Clinical officers and medical assistants	0.7 (0.4 to 1.1)
Yemen	Dieticians and nutritionists	0.1 (0.1 to 0.2)
Yemen	Environmental health workers	0.5 (0.3 to 0.7)
Yemen	Healthcare aides, paramedics, and ambulance workers	0.5 (0.3 to 0.8)
Yemen	Home-based personal care workers	0.1 (0.1 to 0.2)
Yemen	Medical imaging and therapeutic equipment technicians	0.2 (0.1 to 0.3)
Yemen	Medical laboratory technicians	0.4 (0.2 to 0.7)
Yemen	Optometrists and opticians	0.0 (0.0 to 0.0)
Yemen	Physiotherapists and prosthetic technicians	0.1 (0.1 to 0.1)
Yemen	Psychologists	0.1 (0.1 to 0.2)
Yemen	Traditional and complementary medicine practitioners	1.8 (1.3 to 2.4)
**South Asia**	Audiologists, speech therapists, and counsellors	4.1 (3.1 to 5.6)
**South Asia**	Clinical officers and medical assistants	2.6 (1.9 to 3.3)
**South Asia**	Dieticians and nutritionists	0.3 (0.2 to 0.4)

**South Asia**	Environmental health workers	0.5 (0.3 to 0.6)
**South Asia**	Healthcare aides, paramedics, and ambulance workers	2.4 (1.8 to 3.2)
**South Asia**	Home-based personal care workers	7.2 (5.3 to 9.3)
**South Asia**	Medical imaging and therapeutic equipment technicians	1.2 (1.0 to 1.6)
**South Asia**	Medical laboratory technicians	3.0 (2.0 to 4.3)
**South Asia**	Optometrists and opticians	0.8 (0.6 to 0.9)
**South Asia**	Physiotherapists and prosthetic technicians	1.9 (1.6 to 2.2)
**South Asia**	Psychologists	0.3 (0.2 to 0.3)
**South Asia**	Traditional and complementary medicine practitioners	0.9 (0.7 to 1.3)
South Asia	Audiologists, speech therapists, and counsellors	4.1 (3.1 to 5.6)
South Asia	Clinical officers and medical assistants	2.6 (1.9 to 3.3)
South Asia	Dieticians and nutritionists	0.3 (0.2 to 0.4)
South Asia	Environmental health workers	0.5 (0.3 to 0.6)
South Asia	Healthcare aides, paramedics, and ambulance workers	2.4 (1.8 to 3.2)
South Asia	Home-based personal care workers	7.2 (5.3 to 9.3)
South Asia	Medical imaging and therapeutic equipment technicians	1.2 (1.0 to 1.6)
South Asia	Medical laboratory technicians	3.0 (2.0 to 4.3)
South Asia	Optometrists and opticians	0.8 (0.6 to 0.9)
South Asia	Physiotherapists and prosthetic technicians	1.9 (1.6 to 2.2)
South Asia	Psychologists	0.3 (0.2 to 0.3)
South Asia	Traditional and complementary medicine practitioners	0.9 (0.7 to 1.3)
Bangladesh	Audiologists, speech therapists, and counsellors	3.0 (1.9 to 4.4)
Bangladesh	Clinical officers and medical assistants	3.2 (1.9 to 4.9)
Bangladesh	Dieticians and nutritionists	0.3 (0.2 to 0.4)
Bangladesh	Environmental health workers	0.4 (0.3 to 0.6)
Bangladesh	Healthcare aides, paramedics, and ambulance workers	1.5 (1.0 to 2.4)
Bangladesh	Home-based personal care workers	3.6 (2.5 to 5.1)
Bangladesh	Medical imaging and therapeutic equipment technicians	1.2 (0.7 to 1.8)
Bangladesh	Medical laboratory technicians	3.8 (2.4 to 5.6)
Bangladesh	Optometrists and opticians	0.8 (0.5 to 1.2)

Bangladesh	Physiotherapists and prosthetic technicians	2.0 (1.3 to 2.8)
Bangladesh	Psychologists	0.2 (0.1 to 0.4)
Bangladesh	Traditional and complementary medicine practitioners	2.0 (1.3 to 2.9)
Bhutan	Audiologists, speech therapists, and counsellors	4.3 (3.2 to 5.5)
Bhutan	Clinical officers and medical assistants	8.8 (6.7 to 11.6)
Bhutan	Dieticians and nutritionists	0.5 (0.4 to 0.7)
Bhutan	Environmental health workers	0.9 (0.6 to 1.2)
Bhutan	Healthcare aides, paramedics, and ambulance workers	1.8 (1.3 to 2.6)
Bhutan	Home-based personal care workers	1.4 (1.0 to 1.8)
Bhutan	Medical imaging and therapeutic equipment technicians	3.4 (2.5 to 4.5)
Bhutan	Medical laboratory technicians	9.1 (6.5 to 12.1)
Bhutan	Optometrists and opticians	1.0 (0.7 to 1.2)
Bhutan	Physiotherapists and prosthetic technicians	1.6 (1.2 to 2.2)
Bhutan	Psychologists	0.3 (0.2 to 0.4)
Bhutan	Traditional and complementary medicine practitioners	1.3 (0.9 to 1.9)
India	Audiologists, speech therapists, and counsellors	4.8 (3.3 to 6.9)
India	Clinical officers and medical assistants	2.4 (1.6 to 3.6)
India	Dieticians and nutritionists	0.3 (0.2 to 0.4)
India	Environmental health workers	0.5 (0.3 to 0.6)
India	Healthcare aides, paramedics, and ambulance workers	2.8 (1.9 to 4.0)
India	Home-based personal care workers	8.6 (6.3 to 11.1)
India	Medical imaging and therapeutic equipment technicians	1.4 (0.9 to 2.0)
India	Medical laboratory technicians	3.1 (2.0 to 4.7)
India	Optometrists and opticians	0.7 (0.6 to 1.0)
India	Physiotherapists and prosthetic technicians	1.6 (1.2 to 2.1)
India	Psychologists	0.3 (0.2 to 0.4)
India	Traditional and complementary medicine practitioners	0.4 (0.3 to 0.6)
Nepal	Audiologists, speech therapists, and counsellors	1.1 (0.7 to 1.6)
Nepal	Clinical officers and medical assistants	1.2 (0.8 to 1.9)
Nepal	Dieticians and nutritionists	0.1 (0.1 to 0.1)

Nepal	Environmental health workers	0.2 (0.1 to 0.2)
Nepal	Healthcare aides, paramedics, and ambulance workers	0.5 (0.3 to 0.7)
Nepal	Home-based personal care workers	1.3 (0.9 to 1.9)
Nepal	Medical imaging and therapeutic equipment technicians	0.4 (0.2 to 0.6)
Nepal	Medical laboratory technicians	1.4 (0.9 to 2.1)
Nepal	Optometrists and opticians	0.3 (0.2 to 0.4)
Nepal	Physiotherapists and prosthetic technicians	0.7 (0.5 to 1.0)
Nepal	Psychologists	0.1 (0.0 to 0.1)
Nepal	Traditional and complementary medicine practitioners	1.0 (0.6 to 1.4)
Pakistan	Audiologists, speech therapists, and counsellors	1.6 (1.2 to 2.2)
Pakistan	Clinical officers and medical assistants	2.9 (2.2 to 3.8)
Pakistan	Dieticians and nutritionists	0.1 (0.1 to 0.2)
Pakistan	Environmental health workers	0.4 (0.3 to 0.6)
Pakistan	Healthcare aides, paramedics, and ambulance workers	0.8 (0.6 to 1.2)
Pakistan	Home-based personal care workers	2.5 (1.8 to 3.2)
Pakistan	Medical imaging and therapeutic equipment technicians	0.7 (0.5 to 1.0)
Pakistan	Medical laboratory technicians	2.0 (1.4 to 2.7)
Pakistan	Optometrists and opticians	0.9 (0.6 to 1.1)
Pakistan	Physiotherapists and prosthetic technicians	3.6 (2.6 to 4.8)
Pakistan	Psychologists	0.1 (0.1 to 0.2)
Pakistan	Traditional and complementary medicine practitioners	3.1 (2.3 to 4.4)
**Southeast Asia, east Asia, and Oceania**	Audiologists, speech therapists, and counsellors	3.8 (2.5 to 5.8)
**Southeast Asia, east Asia, and Oceania**	Clinical officers and medical assistants	3.6 (2.3 to 5.3)
**Southeast Asia, east Asia, and Oceania**	Dieticians and nutritionists	0.5 (0.4 to 0.6)
**Southeast Asia, east Asia, and Oceania**	Environmental health workers	1.2 (0.9 to 1.5)
**Southeast Asia, east Asia, and Oceania**	Healthcare aides, paramedics, and ambulance workers	2.1 (1.5 to 2.8)
**Southeast Asia, east Asia, and Oceania**	Home-based personal care workers	15.5 (11.5 to 20.4)
**Southeast Asia, east Asia, and Oceania**	Medical imaging and therapeutic equipment technicians	2.2 (1.4 to 3.3)
**Southeast Asia, east Asia, and Oceania**	Medical laboratory technicians	4.1 (2.8 to 6.0)
**Southeast Asia, east Asia, and Oceania**	Optometrists and opticians	1.5 (1.1 to 2.1)

**Southeast Asia, east Asia, and Oceania**	Physiotherapists and prosthetic technicians	2.0 (1.6 to 2.4)
**Southeast Asia, east Asia, and Oceania**	Psychologists	5.2 (3.6 to 7.3)
**Southeast Asia, east Asia, and Oceania**	Traditional and complementary medicine practitioners	5.8 (4.1 to 8.3)
East Asia	Audiologists, speech therapists, and counsellors	5.2 (3.3 to 7.8)
East Asia	Clinical officers and medical assistants	4.0 (2.4 to 6.0)
East Asia	Dieticians and nutritionists	0.6 (0.4 to 0.8)
East Asia	Environmental health workers	1.2 (0.8 to 1.6)
East Asia	Healthcare aides, paramedics, and ambulance workers	1.9 (1.3 to 2.8)
East Asia	Home-based personal care workers	20.5 (14.4 to 27.8)
East Asia	Medical imaging and therapeutic equipment technicians	3.0 (1.8 to 4.5)
East Asia	Medical laboratory technicians	5.6 (3.5 to 8.5)
East Asia	Optometrists and opticians	1.5 (1.0 to 2.1)
East Asia	Physiotherapists and prosthetic technicians	1.1 (0.8 to 1.6)
East Asia	Psychologists	7.2 (4.8 to 10.1)
East Asia	Traditional and complementary medicine practitioners	7.3 (5.0 to 10.5)
China	Audiologists, speech therapists, and counsellors	5.2 (3.4 to 7.9)
China	Clinical officers and medical assistants	4.0 (2.5 to 6.0)
China	Dieticians and nutritionists	0.5 (0.3 to 0.7)
China	Environmental health workers	1.0 (0.7 to 1.5)
China	Healthcare aides, paramedics, and ambulance workers	1.7 (1.1 to 2.5)
China	Home-based personal care workers	20.9 (14.7 to 28.5)
China	Medical imaging and therapeutic equipment technicians	3.0 (1.8 to 4.5)
China	Medical laboratory technicians	5.6 (3.4 to 8.6)
China	Optometrists and opticians	1.4 (1.0 to 1.9)
China	Physiotherapists and prosthetic technicians	1.0 (0.7 to 1.5)
China	Psychologists	7.1 (4.7 to 10.0)
China	Traditional and complementary medicine practitioners	7.3 (5.0 to 10.6)
North Korea	Audiologists, speech therapists, and counsellors	0.2 (0.1 to 0.3)
North Korea	Clinical officers and medical assistants	0.3 (0.2 to 0.5)
North Korea	Dieticians and nutritionists	0.1 (0.1 to 0.1)

North Korea	Environmental health workers	0.5 (0.3 to 0.7)
North Korea	Healthcare aides, paramedics, and ambulance workers	0.6 (0.4 to 0.9)
North Korea	Home-based personal care workers	0.3 (0.2 to 0.4)
North Korea	Medical imaging and therapeutic equipment technicians	0.1 (0.1 to 0.2)
North Korea	Medical laboratory technicians	0.3 (0.2 to 0.5)
North Korea	Optometrists and opticians	0.1 (0.1 to 0.1)
North Korea	Physiotherapists and prosthetic technicians	0.2 (0.1 to 0.3)
North Korea	Psychologists	0.3 (0.2 to 0.4)
North Korea	Traditional and complementary medicine practitioners	0.8 (0.6 to 1.2)
Taiwan	Audiologists, speech therapists, and counsellors	8.2 (5.3 to 12.3)
Taiwan	Clinical officers and medical assistants	6.1 (3.7 to 9.1)
Taiwan	Dieticians and nutritionists	7.0 (4.9 to 10.2)
Taiwan	Environmental health workers	9.1 (6.1 to 12.6)
Taiwan	Healthcare aides, paramedics, and ambulance workers	16.9 (10.8 to 24.4)
Taiwan	Home-based personal care workers	15.0 (10.8 to 19.9)
Taiwan	Medical imaging and therapeutic equipment technicians	6.1 (3.7 to 9.2)
Taiwan	Medical laboratory technicians	11.6 (7.5 to 17.1)
Taiwan	Optometrists and opticians	9.7 (6.9 to 13.3)
Taiwan	Physiotherapists and prosthetic technicians	9.6 (6.7 to 13.5)
Taiwan	Psychologists	18.6 (12.3 to 26.2)
Taiwan	Traditional and complementary medicine practitioners	12.7 (8.8 to 18.9)
Oceania	Audiologists, speech therapists, and counsellors	0.2 (0.1 to 0.3)
Oceania	Clinical officers and medical assistants	0.1 (0.1 to 0.2)
Oceania	Dieticians and nutritionists	0.1 (0.1 to 0.1)
Oceania	Environmental health workers	0.3 (0.2 to 0.5)
Oceania	Healthcare aides, paramedics, and ambulance workers	1.4 (1.1 to 1.9)
Oceania	Home-based personal care workers	0.4 (0.2 to 0.5)
Oceania	Medical imaging and therapeutic equipment technicians	0.1 (0.0 to 0.1)
Oceania	Medical laboratory technicians	0.1 (0.1 to 0.2)
Oceania	Optometrists and opticians	0.1 (0.0 to 0.1)

Oceania	Physiotherapists and prosthetic technicians	0.5 (0.4 to 0.8)
Oceania	Psychologists	0.1 (0.1 to 0.2)
Oceania	Traditional and complementary medicine practitioners	0.2 (0.1 to 0.3)
American Samoa	Audiologists, speech therapists, and counsellors	0.3 (0.2 to 0.5)
American Samoa	Clinical officers and medical assistants	0.1 (0.1 to 0.2)
American Samoa	Dieticians and nutritionists	0.2 (0.1 to 0.3)
American Samoa	Environmental health workers	0.3 (0.2 to 0.5)
American Samoa	Healthcare aides, paramedics, and ambulance workers	1.9 (1.3 to 2.6)
American Samoa	Home-based personal care workers	0.9 (0.6 to 1.2)
American Samoa	Medical imaging and therapeutic equipment technicians	0.2 (0.1 to 0.3)
American Samoa	Medical laboratory technicians	0.2 (0.1 to 0.3)
American Samoa	Optometrists and opticians	0.2 (0.1 to 0.3)
American Samoa	Physiotherapists and prosthetic technicians	1.0 (0.6 to 1.4)
American Samoa	Psychologists	0.3 (0.2 to 0.4)
American Samoa	Traditional and complementary medicine practitioners	0.1 (0.1 to 0.2)
Cook Islands	Audiologists, speech therapists, and counsellors	2.5 (1.5 to 3.9)
Cook Islands	Clinical officers and medical assistants	1.0 (0.6 to 1.7)
Cook Islands	Dieticians and nutritionists	1.7 (1.1 to 2.7)
Cook Islands	Environmental health workers	2.4 (1.6 to 3.6)
Cook Islands	Healthcare aides, paramedics, and ambulance workers	17.1 (12.6 to 23.7)
Cook Islands	Home-based personal care workers	6.2 (4.2 to 8.7)
Cook Islands	Medical imaging and therapeutic equipment technicians	1.4 (0.8 to 2.2)
Cook Islands	Medical laboratory technicians	1.3 (0.8 to 2.1)
Cook Islands	Optometrists and opticians	1.7 (1.1 to 2.5)
Cook Islands	Physiotherapists and prosthetic technicians	7.6 (5.0 to 10.8)
Cook Islands	Psychologists	2.5 (1.6 to 3.7)
Cook Islands	Traditional and complementary medicine practitioners	0.5 (0.3 to 0.7)
Fiji	Audiologists, speech therapists, and counsellors	0.6 (0.3 to 0.9)
Fiji	Clinical officers and medical assistants	0.2 (0.1 to 0.3)
Fiji	Dieticians and nutritionists	0.4 (0.2 to 0.6)

Fiji	Environmental health workers	0.7 (0.5 to 1.1)
Fiji	Healthcare aides, paramedics, and ambulance workers	5.2 (4.0 to 6.5)
Fiji	Home-based personal care workers	1.2 (0.8 to 1.6)
Fiji	Medical imaging and therapeutic equipment technicians	0.3 (0.2 to 0.4)
Fiji	Medical laboratory technicians	0.2 (0.1 to 0.4)
Fiji	Optometrists and opticians	0.2 (0.2 to 0.4)
Fiji	Physiotherapists and prosthetic technicians	1.9 (1.3 to 2.7)
Fiji	Psychologists	0.4 (0.3 to 0.6)
Fiji	Traditional and complementary medicine practitioners	0.1 (0.1 to 0.2)
Guam	Audiologists, speech therapists, and counsellors	0.6 (0.3 to 0.9)
Guam	Clinical officers and medical assistants	0.2 (0.1 to 0.3)
Guam	Dieticians and nutritionists	0.4 (0.2 to 0.5)
Guam	Environmental health workers	0.4 (0.3 to 0.6)
Guam	Healthcare aides, paramedics, and ambulance workers	2.4 (1.6 to 3.4)
Guam	Home-based personal care workers	1.6 (1.1 to 2.2)
Guam	Medical imaging and therapeutic equipment technicians	0.3 (0.2 to 0.5)
Guam	Medical laboratory technicians	0.2 (0.1 to 0.4)
Guam	Optometrists and opticians	0.4 (0.3 to 0.6)
Guam	Physiotherapists and prosthetic technicians	1.5 (1.1 to 2.2)
Guam	Psychologists	0.5 (0.3 to 0.7)
Guam	Traditional and complementary medicine practitioners	0.1 (0.1 to 0.2)
Kiribati	Audiologists, speech therapists, and counsellors	0.3 (0.1 to 0.4)
Kiribati	Clinical officers and medical assistants	0.2 (0.1 to 0.4)
Kiribati	Dieticians and nutritionists	0.1 (0.1 to 0.2)
Kiribati	Environmental health workers	0.7 (0.4 to 1.0)
Kiribati	Healthcare aides, paramedics, and ambulance workers	3.3 (2.6 to 4.2)
Kiribati	Home-based personal care workers	0.5 (0.3 to 0.7)
Kiribati	Medical imaging and therapeutic equipment technicians	0.1 (0.1 to 0.2)
Kiribati	Medical laboratory technicians	0.1 (0.1 to 0.2)
Kiribati	Optometrists and opticians	0.0 (0.0 to 0.1)

Kiribati	Physiotherapists and prosthetic technicians	0.5 (0.3 to 0.8)
Kiribati	Psychologists	0.1 (0.1 to 0.1)
Kiribati	Traditional and complementary medicine practitioners	0.2 (0.1 to 0.4)
Marshall Islands	Audiologists, speech therapists, and counsellors	0.4 (0.2 to 0.6)
Marshall Islands	Clinical officers and medical assistants	0.2 (0.1 to 0.4)
Marshall Islands	Dieticians and nutritionists	0.2 (0.1 to 0.3)
Marshall Islands	Environmental health workers	0.6 (0.4 to 0.9)
Marshall Islands	Healthcare aides, paramedics, and ambulance workers	2.6 (1.9 to 3.5)
Marshall Islands	Home-based personal care workers	0.9 (0.6 to 1.2)
Marshall Islands	Medical imaging and therapeutic equipment technicians	0.2 (0.1 to 0.3)
Marshall Islands	Medical laboratory technicians	0.2 (0.1 to 0.3)
Marshall Islands	Optometrists and opticians	0.1 (0.1 to 0.2)
Marshall Islands	Physiotherapists and prosthetic technicians	0.9 (0.6 to 1.3)
Marshall Islands	Psychologists	0.2 (0.1 to 0.3)
Marshall Islands	Traditional and complementary medicine practitioners	0.3 (0.2 to 0.4)
Federated States of Micronesia	Audiologists, speech therapists, and counsellors	0.5 (0.3 to 0.9)
Federated States of Micronesia	Clinical officers and medical assistants	0.3 (0.2 to 0.6)
Federated States of Micronesia	Dieticians and nutritionists	0.3 (0.2 to 0.4)
Federated States of Micronesia	Environmental health workers	0.8 (0.5 to 1.2)
Federated States of Micronesia	Healthcare aides, paramedics, and ambulance workers	3.5 (2.6 to 4.9)
Federated States of Micronesia	Home-based personal care workers	1.2 (0.8 to 1.7)
Federated States of Micronesia	Medical imaging and therapeutic equipment technicians	0.2 (0.1 to 0.4)
Federated States of Micronesia	Medical laboratory technicians	0.3 (0.2 to 0.5)
Federated States of Micronesia	Optometrists and opticians	0.2 (0.2 to 0.4)
Federated States of Micronesia	Physiotherapists and prosthetic technicians	1.6 (1.0 to 2.2)
Federated States of Micronesia	Psychologists	0.4 (0.2 to 0.6)
Federated States of Micronesia	Traditional and complementary medicine practitioners	0.3 (0.2 to 0.5)
Nauru	Audiologists, speech therapists, and counsellors	0.5 (0.3 to 0.8)
Nauru	Clinical officers and medical assistants	0.3 (0.2 to 0.5)
Nauru	Dieticians and nutritionists	0.3 (0.2 to 0.4)

Nauru	Environmental health workers	0.8 (0.6 to 1.2)
Nauru	Healthcare aides, paramedics, and ambulance workers	4.0 (2.9 to 5.5)
Nauru	Home-based personal care workers	1.3 (0.8 to 1.8)
Nauru	Medical imaging and therapeutic equipment technicians	0.2 (0.1 to 0.4)
Nauru	Medical laboratory technicians	0.3 (0.2 to 0.5)
Nauru	Optometrists and opticians	0.3 (0.2 to 0.4)
Nauru	Physiotherapists and prosthetic technicians	1.7 (1.1 to 2.4)
Nauru	Psychologists	0.4 (0.2 to 0.5)
Nauru	Traditional and complementary medicine practitioners	0.3 (0.2 to 0.5)
Niue	Audiologists, speech therapists, and counsellors	0.4 (0.2 to 0.5)
Niue	Clinical officers and medical assistants	0.1 (0.1 to 0.2)
Niue	Dieticians and nutritionists	0.2 (0.1 to 0.4)
Niue	Environmental health workers	0.4 (0.3 to 0.6)
Niue	Healthcare aides, paramedics, and ambulance workers	2.8 (2.1 to 3.9)
Niue	Home-based personal care workers	0.9 (0.6 to 1.2)
Niue	Medical imaging and therapeutic equipment technicians	0.2 (0.1 to 0.3)
Niue	Medical laboratory technicians	0.2 (0.1 to 0.3)
Niue	Optometrists and opticians	0.2 (0.1 to 0.3)
Niue	Physiotherapists and prosthetic technicians	1.1 (0.7 to 1.6)
Niue	Psychologists	0.3 (0.2 to 0.4)
Niue	Traditional and complementary medicine practitioners	0.1 (0.1 to 0.1)
Northern Mariana Islands	Audiologists, speech therapists, and counsellors	0.5 (0.3 to 0.8)
Northern Mariana Islands	Clinical officers and medical assistants	0.2 (0.1 to 0.3)
Northern Mariana Islands	Dieticians and nutritionists	0.3 (0.2 to 0.5)
Northern Mariana Islands	Environmental health workers	0.4 (0.2 to 0.6)
Northern Mariana Islands	Healthcare aides, paramedics, and ambulance workers	2.3 (1.6 to 3.3)
Northern Mariana Islands	Home-based personal care workers	1.3 (0.9 to 1.8)
Northern Mariana Islands	Medical imaging and therapeutic equipment technicians	0.3 (0.2 to 0.4)
Northern Mariana Islands	Medical laboratory technicians	0.3 (0.1 to 0.4)
Northern Mariana Islands	Optometrists and opticians	0.4 (0.3 to 0.6)

Northern Mariana Islands	Physiotherapists and prosthetic technicians	1.5 (1.0 to 2.1)
Northern Mariana Islands	Psychologists	0.5 (0.3 to 0.7)
Northern Mariana Islands	Traditional and complementary medicine practitioners	0.1 (0.1 to 0.1)
Palau	Audiologists, speech therapists, and counsellors	3.0 (1.7 to 4.6)
Palau	Clinical officers and medical assistants	1.2 (0.7 to 2.1)
Palau	Dieticians and nutritionists	2.1 (1.3 to 3.3)
Palau	Environmental health workers	3.6 (2.3 to 5.4)
Palau	Healthcare aides, paramedics, and ambulance workers	27.9 (21.1 to 37.5)
Palau	Home-based personal care workers	7.4 (4.9 to 10.6)
Palau	Medical imaging and therapeutic equipment technicians	1.6 (0.9 to 2.6)
Palau	Medical laboratory technicians	1.6 (1.0 to 2.6)
Palau	Optometrists and opticians	2.0 (1.2 to 3.0)
Palau	Physiotherapists and prosthetic technicians	10.5 (6.9 to 15.1)
Palau	Psychologists	2.8 (1.8 to 4.1)
Palau	Traditional and complementary medicine practitioners	0.6 (0.4 to 1.0)
Papua New Guinea	Audiologists, speech therapists, and counsellors	0.1 (0.1 to 0.2)
Papua New Guinea	Clinical officers and medical assistants	0.1 (0.1 to 0.2)
Papua New Guinea	Dieticians and nutritionists	0.0 (0.0 to 0.1)
Papua New Guinea	Environmental health workers	0.3 (0.2 to 0.4)
Papua New Guinea	Healthcare aides, paramedics, and ambulance workers	0.7 (0.5 to 1.0)
Papua New Guinea	Home-based personal care workers	0.2 (0.1 to 0.3)
Papua New Guinea	Medical imaging and therapeutic equipment technicians	0.0 (0.0 to 0.1)
Papua New Guinea	Medical laboratory technicians	0.1 (0.0 to 0.1)
Papua New Guinea	Optometrists and opticians	0.0 (0.0 to 0.0)
Papua New Guinea	Physiotherapists and prosthetic technicians	0.3 (0.2 to 0.4)
Papua New Guinea	Psychologists	0.0 (0.0 to 0.1)
Papua New Guinea	Traditional and complementary medicine practitioners	0.2 (0.1 to 0.3)
Samoa	Audiologists, speech therapists, and counsellors	0.3 (0.2 to 0.4)
Samoa	Clinical officers and medical assistants	0.1 (0.1 to 0.2)
Samoa	Dieticians and nutritionists	0.1 (0.1 to 0.2)

Samoa	Environmental health workers	0.2 (0.2 to 0.4)
Samoa	Healthcare aides, paramedics, and ambulance workers	0.7 (0.4 to 1.0)
Samoa	Home-based personal care workers	0.8 (0.5 to 1.0)
Samoa	Medical imaging and therapeutic equipment technicians	0.1 (0.1 to 0.2)
Samoa	Medical laboratory technicians	0.1 (0.1 to 0.2)
Samoa	Optometrists and opticians	0.2 (0.1 to 0.2)
Samoa	Physiotherapists and prosthetic technicians	0.7 (0.5 to 1.0)
Samoa	Psychologists	0.1 (0.1 to 0.2)
Samoa	Traditional and complementary medicine practitioners	0.2 (0.1 to 0.4)
Solomon Islands	Audiologists, speech therapists, and counsellors	0.4 (0.2 to 0.6)
Solomon Islands	Clinical officers and medical assistants	0.3 (0.2 to 0.5)
Solomon Islands	Dieticians and nutritionists	0.2 (0.1 to 0.3)
Solomon Islands	Environmental health workers	0.9 (0.5 to 1.3)
Solomon Islands	Healthcare aides, paramedics, and ambulance workers	3.9 (3.0 to 5.1)
Solomon Islands	Home-based personal care workers	0.7 (0.4 to 1.0)
Solomon Islands	Medical imaging and therapeutic equipment technicians	0.1 (0.1 to 0.2)
Solomon Islands	Medical laboratory technicians	0.3 (0.2 to 0.4)
Solomon Islands	Optometrists and opticians	0.2 (0.1 to 0.2)
Solomon Islands	Physiotherapists and prosthetic technicians	1.2 (0.8 to 1.8)
Solomon Islands	Psychologists	0.3 (0.2 to 0.4)
Solomon Islands	Traditional and complementary medicine practitioners	0.3 (0.2 to 0.4)
Tokelau	Audiologists, speech therapists, and counsellors	1.3 (0.8 to 2.1)
Tokelau	Clinical officers and medical assistants	0.6 (0.3 to 1.0)
Tokelau	Dieticians and nutritionists	0.8 (0.5 to 1.3)
Tokelau	Environmental health workers	1.4 (1.0 to 2.1)
Tokelau	Healthcare aides, paramedics, and ambulance workers	8.8 (6.4 to 12.2)
Tokelau	Home-based personal care workers	3.7 (2.5 to 5.1)
Tokelau	Medical imaging and therapeutic equipment technicians	0.7 (0.4 to 1.1)
Tokelau	Medical laboratory technicians	0.7 (0.4 to 1.0)
Tokelau	Optometrists and opticians	0.8 (0.5 to 1.2)

Tokelau	Physiotherapists and prosthetic technicians	4.2 (2.8 to 6.0)
Tokelau	Psychologists	1.0 (0.6 to 1.5)
Tokelau	Traditional and complementary medicine practitioners	0.4 (0.3 to 0.7)
Tonga	Audiologists, speech therapists, and counsellors	0.4 (0.2 to 0.6)
Tonga	Clinical officers and medical assistants	0.2 (0.1 to 0.3)
Tonga	Dieticians and nutritionists	0.2 (0.1 to 0.3)
Tonga	Environmental health workers	0.4 (0.3 to 0.6)
Tonga	Healthcare aides, paramedics, and ambulance workers	1.4 (1.0 to 2.1)
Tonga	Home-based personal care workers	1.0 (0.7 to 1.3)
Tonga	Medical imaging and therapeutic equipment technicians	0.2 (0.1 to 0.3)
Tonga	Medical laboratory technicians	0.2 (0.1 to 0.3)
Tonga	Optometrists and opticians	0.2 (0.1 to 0.3)
Tonga	Physiotherapists and prosthetic technicians	1.0 (0.7 to 1.4)
Tonga	Psychologists	0.2 (0.2 to 0.4)
Tonga	Traditional and complementary medicine practitioners	0.2 (0.1 to 0.4)
Tuvalu	Audiologists, speech therapists, and counsellors	0.3 (0.2 to 0.4)
Tuvalu	Clinical officers and medical assistants	0.1 (0.1 to 0.2)
Tuvalu	Dieticians and nutritionists	0.1 (0.1 to 0.2)
Tuvalu	Environmental health workers	0.3 (0.2 to 0.4)
Tuvalu	Healthcare aides, paramedics, and ambulance workers	0.8 (0.6 to 1.2)
Tuvalu	Home-based personal care workers	0.7 (0.5 to 1.0)
Tuvalu	Medical imaging and therapeutic equipment technicians	0.1 (0.1 to 0.2)
Tuvalu	Medical laboratory technicians	0.1 (0.1 to 0.2)
Tuvalu	Optometrists and opticians	0.1 (0.1 to 0.2)
Tuvalu	Physiotherapists and prosthetic technicians	0.6 (0.4 to 0.9)
Tuvalu	Psychologists	0.1 (0.1 to 0.2)
Tuvalu	Traditional and complementary medicine practitioners	0.3 (0.2 to 0.4)
Vanuatu	Audiologists, speech therapists, and counsellors	0.2 (0.1 to 0.3)
Vanuatu	Clinical officers and medical assistants	0.1 (0.1 to 0.3)
Vanuatu	Dieticians and nutritionists	0.1 (0.1 to 0.1)

Vanuatu	Environmental health workers	0.3 (0.2 to 0.5)
Vanuatu	Healthcare aides, paramedics, and ambulance workers	0.9 (0.6 to 1.3)
Vanuatu	Home-based personal care workers	0.4 (0.3 to 0.6)
Vanuatu	Medical imaging and therapeutic equipment technicians	0.1 (0.0 to 0.1)
Vanuatu	Medical laboratory technicians	0.1 (0.1 to 0.2)
Vanuatu	Optometrists and opticians	0.1 (0.1 to 0.1)
Vanuatu	Physiotherapists and prosthetic technicians	0.6 (0.4 to 0.8)
Vanuatu	Psychologists	0.1 (0.1 to 0.2)
Vanuatu	Traditional and complementary medicine practitioners	0.3 (0.2 to 0.4)
Southeast Asia	Audiologists, speech therapists, and counsellors	1.1 (0.7 to 1.6)
Southeast Asia	Clinical officers and medical assistants	2.8 (1.9 to 4.2)
Southeast Asia	Dieticians and nutritionists	0.3 (0.2 to 0.4)
Southeast Asia	Environmental health workers	1.2 (0.8 to 1.7)
Southeast Asia	Healthcare aides, paramedics, and ambulance workers	2.4 (2.0 to 3.0)
Southeast Asia	Home-based personal care workers	5.5 (4.7 to 6.2)
Southeast Asia	Medical imaging and therapeutic equipment technicians	0.7 (0.5 to 1.0)
Southeast Asia	Medical laboratory technicians	1.1 (0.7 to 1.7)
Southeast Asia	Optometrists and opticians	1.6 (1.2 to 2.3)
Southeast Asia	Physiotherapists and prosthetic technicians	3.7 (2.7 to 4.9)
Southeast Asia	Psychologists	1.3 (1.1 to 1.5)
Southeast Asia	Traditional and complementary medicine practitioners	3.0 (2.0 to 4.2)
Cambodia	Audiologists, speech therapists, and counsellors	2.0 (1.4 to 2.7)
Cambodia	Clinical officers and medical assistants	7.2 (5.0 to 10.4)
Cambodia	Dieticians and nutritionists	0.5 (0.4 to 0.6)
Cambodia	Environmental health workers	2.3 (1.6 to 3.0)
Cambodia	Healthcare aides, paramedics, and ambulance workers	6.6 (4.5 to 9.2)
Cambodia	Home-based personal care workers	8.9 (6.9 to 11.4)
Cambodia	Medical imaging and therapeutic equipment technicians	0.3 (0.2 to 0.4)
Cambodia	Medical laboratory technicians	0.5 (0.3 to 0.8)
Cambodia	Optometrists and opticians	2.1 (1.6 to 2.8)

Cambodia	Physiotherapists and prosthetic technicians	6.2 (4.6 to 8.2)
Cambodia	Psychologists	0.5 (0.4 to 0.7)
Cambodia	Traditional and complementary medicine practitioners	0.9 (0.6 to 1.4)
Indonesia	Audiologists, speech therapists, and counsellors	1.0 (0.6 to 1.5)
Indonesia	Clinical officers and medical assistants	2.5 (1.5 to 3.9)
Indonesia	Dieticians and nutritionists	0.3 (0.2 to 0.4)
Indonesia	Environmental health workers	0.8 (0.5 to 1.2)
Indonesia	Healthcare aides, paramedics, and ambulance workers	1.7 (1.1 to 2.7)
Indonesia	Home-based personal care workers	4.4 (3.2 to 6.0)
Indonesia	Medical imaging and therapeutic equipment technicians	0.4 (0.2 to 0.6)
Indonesia	Medical laboratory technicians	0.6 (0.4 to 1.0)
Indonesia	Optometrists and opticians	1.5 (1.0 to 2.2)
Indonesia	Physiotherapists and prosthetic technicians	3.0 (2.0 to 4.1)
Indonesia	Psychologists	0.8 (0.5 to 1.1)
Indonesia	Traditional and complementary medicine practitioners	1.2 (0.8 to 1.9)
Laos	Audiologists, speech therapists, and counsellors	0.4 (0.3 to 0.7)
Laos	Clinical officers and medical assistants	1.7 (1.1 to 2.6)
Laos	Dieticians and nutritionists	0.1 (0.1 to 0.1)
Laos	Environmental health workers	0.6 (0.4 to 0.9)
Laos	Healthcare aides, paramedics, and ambulance workers	1.1 (0.7 to 1.7)
Laos	Home-based personal care workers	1.7 (1.2 to 2.3)
Laos	Medical imaging and therapeutic equipment technicians	0.1 (0.1 to 0.1)
Laos	Medical laboratory technicians	0.3 (0.2 to 0.5)
Laos	Optometrists and opticians	0.4 (0.3 to 0.6)
Laos	Physiotherapists and prosthetic technicians	1.3 (0.9 to 1.8)
Laos	Psychologists	0.1 (0.1 to 0.2)
Laos	Traditional and complementary medicine practitioners	1.3 (0.8 to 1.8)
Malaysia	Audiologists, speech therapists, and counsellors	2.5 (1.6 to 3.8)
Malaysia	Clinical officers and medical assistants	5.0 (3.0 to 7.7)
Malaysia	Dieticians and nutritionists	0.7 (0.5 to 1.1)

Malaysia	Environmental health workers	1.4 (0.9 to 2.0)
Malaysia	Healthcare aides, paramedics, and ambulance workers	10.5 (6.8 to 15.4)
Malaysia	Home-based personal care workers	23.7 (18.0 to 32.1)
Malaysia	Medical imaging and therapeutic equipment technicians	2.3 (1.5 to 3.4)
Malaysia	Medical laboratory technicians	4.2 (2.6 to 6.3)
Malaysia	Optometrists and opticians	4.7 (3.3 to 6.4)
Malaysia	Physiotherapists and prosthetic technicians	8.1 (5.5 to 11.4)
Malaysia	Psychologists	1.1 (0.8 to 1.6)
Malaysia	Traditional and complementary medicine practitioners	1.2 (0.8 to 1.8)
Maldives	Audiologists, speech therapists, and counsellors	1.8 (1.2 to 2.7)
Maldives	Clinical officers and medical assistants	24.8 (18.0 to 32.5)
Maldives	Dieticians and nutritionists	0.6 (0.4 to 0.9)
Maldives	Environmental health workers	0.7 (0.5 to 1.0)
Maldives	Healthcare aides, paramedics, and ambulance workers	10.0 (6.8 to 14.1)
Maldives	Home-based personal care workers	20.5 (15.3 to 26.1)
Maldives	Medical imaging and therapeutic equipment technicians	1.2 (0.8 to 1.9)
Maldives	Medical laboratory technicians	7.9 (5.4 to 11.3)
Maldives	Optometrists and opticians	2.9 (2.0 to 4.1)
Maldives	Physiotherapists and prosthetic technicians	1.7 (1.2 to 2.5)
Maldives	Psychologists	2.0 (1.4 to 2.9)
Maldives	Traditional and complementary medicine practitioners	3.2 (2.2 to 4.8)
Mauritius	Audiologists, speech therapists, and counsellors	0.3 (0.2 to 0.5)
Mauritius	Clinical officers and medical assistants	0.6 (0.4 to 1.0)
Mauritius	Dieticians and nutritionists	0.1 (0.1 to 0.1)
Mauritius	Environmental health workers	0.2 (0.1 to 0.3)
Mauritius	Healthcare aides, paramedics, and ambulance workers	0.3 (0.2 to 0.5)
Mauritius	Home-based personal care workers	1.7 (1.3 to 2.2)
Mauritius	Medical imaging and therapeutic equipment technicians	0.1 (0.1 to 0.2)
Mauritius	Medical laboratory technicians	0.2 (0.1 to 0.3)
Mauritius	Optometrists and opticians	0.6 (0.4 to 0.9)

Mauritius	Physiotherapists and prosthetic technicians	0.9 (0.6 to 1.3)
Mauritius	Psychologists	0.2 (0.1 to 0.3)
Mauritius	Traditional and complementary medicine practitioners	0.3 (0.2 to 0.5)
Myanmar	Audiologists, speech therapists, and counsellors	0.3 (0.2 to 0.4)
Myanmar	Clinical officers and medical assistants	1.0 (0.6 to 1.5)
Myanmar	Dieticians and nutritionists	0.1 (0.0 to 0.1)
Myanmar	Environmental health workers	0.4 (0.2 to 0.5)
Myanmar	Healthcare aides, paramedics, and ambulance workers	0.6 (0.4 to 1.0)
Myanmar	Home-based personal care workers	1.0 (0.7 to 1.4)
Myanmar	Medical imaging and therapeutic equipment technicians	0.1 (0.1 to 0.1)
Myanmar	Medical laboratory technicians	0.2 (0.1 to 0.3)
Myanmar	Optometrists and opticians	0.3 (0.2 to 0.4)
Myanmar	Physiotherapists and prosthetic technicians	0.8 (0.5 to 1.1)
Myanmar	Psychologists	0.2 (0.1 to 0.2)
Myanmar	Traditional and complementary medicine practitioners	0.7 (0.4 to 1.0)
Philippines	Audiologists, speech therapists, and counsellors	1.5 (1.0 to 2.2)
Philippines	Clinical officers and medical assistants	4.7 (2.9 to 7.0)
Philippines	Dieticians and nutritionists	0.4 (0.3 to 0.5)
Philippines	Environmental health workers	3.8 (2.5 to 5.2)
Philippines	Healthcare aides, paramedics, and ambulance workers	3.5 (2.3 to 5.1)
Philippines	Home-based personal care workers	9.4 (6.8 to 12.9)
Philippines	Medical imaging and therapeutic equipment technicians	1.7 (1.1 to 2.5)
Philippines	Medical laboratory technicians	1.9 (1.2 to 2.9)
Philippines	Optometrists and opticians	2.5 (1.8 to 3.5)
Philippines	Physiotherapists and prosthetic technicians	6.7 (4.7 to 9.4)
Philippines	Psychologists	2.9 (2.0 to 4.1)
Philippines	Traditional and complementary medicine practitioners	11.8 (8.2 to 17.4)
Seychelles	Audiologists, speech therapists, and counsellors	0.4 (0.2 to 0.6)
Seychelles	Clinical officers and medical assistants	0.8 (0.5 to 1.3)
Seychelles	Dieticians and nutritionists	0.1 (0.1 to 0.2)

Seychelles	Environmental health workers	0.3 (0.2 to 0.4)
Seychelles	Healthcare aides, paramedics, and ambulance workers	0.7 (0.5 to 1.2)
Seychelles	Home-based personal care workers	1.9 (1.4 to 2.6)
Seychelles	Medical imaging and therapeutic equipment technicians	0.2 (0.1 to 0.3)
Seychelles	Medical laboratory technicians	0.2 (0.1 to 0.4)
Seychelles	Optometrists and opticians	0.7 (0.5 to 1.0)
Seychelles	Physiotherapists and prosthetic technicians	1.3 (0.9 to 1.7)
Seychelles	Psychologists	0.3 (0.2 to 0.5)
Seychelles	Traditional and complementary medicine practitioners	0.3 (0.2 to 0.5)
Sri Lanka	Audiologists, speech therapists, and counsellors	0.4 (0.2 to 0.6)
Sri Lanka	Clinical officers and medical assistants	0.9 (0.5 to 1.4)
Sri Lanka	Dieticians and nutritionists	0.1 (0.1 to 0.1)
Sri Lanka	Environmental health workers	0.2 (0.1 to 0.4)
Sri Lanka	Healthcare aides, paramedics, and ambulance workers	0.4 (0.3 to 0.7)
Sri Lanka	Home-based personal care workers	2.0 (1.4 to 2.7)
Sri Lanka	Medical imaging and therapeutic equipment technicians	0.0 (0.0 to 0.1)
Sri Lanka	Medical laboratory technicians	0.1 (0.0 to 0.1)
Sri Lanka	Optometrists and opticians	0.5 (0.4 to 0.8)
Sri Lanka	Physiotherapists and prosthetic technicians	1.0 (0.6 to 1.3)
Sri Lanka	Psychologists	0.5 (0.4 to 0.8)
Sri Lanka	Traditional and complementary medicine practitioners	2.5 (1.7 to 3.4)
Thailand	Audiologists, speech therapists, and counsellors	0.8 (0.5 to 1.3)
Thailand	Clinical officers and medical assistants	0.9 (0.6 to 1.5)
Thailand	Dieticians and nutritionists	0.1 (0.0 to 0.1)
Thailand	Environmental health workers	0.1 (0.1 to 0.2)
Thailand	Healthcare aides, paramedics, and ambulance workers	1.2 (0.8 to 1.8)
Thailand	Home-based personal care workers	2.1 (1.5 to 2.9)
Thailand	Medical imaging and therapeutic equipment technicians	0.3 (0.2 to 0.5)
Thailand	Medical laboratory technicians	0.6 (0.4 to 0.9)
Thailand	Optometrists and opticians	0.9 (0.6 to 1.3)

Thailand	Physiotherapists and prosthetic technicians	3.2 (2.3 to 4.2)
Thailand	Psychologists	0.8 (0.5 to 1.1)
Thailand	Traditional and complementary medicine practitioners	2.3 (1.5 to 3.1)
Timor-Leste	Audiologists, speech therapists, and counsellors	0.0 (0.0 to 0.1)
Timor-Leste	Clinical officers and medical assistants	0.2 (0.1 to 0.3)
Timor-Leste	Dieticians and nutritionists	0.0 (0.0 to 0.0)
Timor-Leste	Environmental health workers	0.1 (0.0 to 0.1)
Timor-Leste	Healthcare aides, paramedics, and ambulance workers	0.1 (0.0 to 0.1)
Timor-Leste	Home-based personal care workers	0.2 (0.1 to 0.3)
Timor-Leste	Medical imaging and therapeutic equipment technicians	0.0 (0.0 to 0.0)
Timor-Leste	Medical laboratory technicians	0.0 (0.0 to 0.0)
Timor-Leste	Optometrists and opticians	0.0 (0.0 to 0.0)
Timor-Leste	Physiotherapists and prosthetic technicians	0.1 (0.1 to 0.2)
Timor-Leste	Psychologists	0.0 (0.0 to 0.0)
Timor-Leste	Traditional and complementary medicine practitioners	1.7 (1.4 to 2.0)
Viet Nam	Audiologists, speech therapists, and counsellors	1.0 (0.6 to 1.6)
Viet Nam	Clinical officers and medical assistants	2.7 (1.7 to 4.4)
Viet Nam	Dieticians and nutritionists	0.3 (0.2 to 0.4)
Viet Nam	Environmental health workers	0.8 (0.5 to 1.1)
Viet Nam	Healthcare aides, paramedics, and ambulance workers	1.7 (1.1 to 2.8)
Viet Nam	Home-based personal care workers	3.4 (2.4 to 4.7)
Viet Nam	Medical imaging and therapeutic equipment technicians	0.6 (0.4 to 1.0)
Viet Nam	Medical laboratory technicians	2.0 (1.2 to 2.9)
Viet Nam	Optometrists and opticians	1.6 (1.1 to 2.3)
Viet Nam	Physiotherapists and prosthetic technicians	3.3 (2.2 to 4.5)
Viet Nam	Psychologists	2.3 (1.6 to 3.2)
Viet Nam	Traditional and complementary medicine practitioners	0.8 (0.5 to 1.1)
**Sub-Saharan Africa**	Audiologists, speech therapists, and counsellors	1.5 (1.1 to 1.9)
**Sub-Saharan Africa**	Clinical officers and medical assistants	1.1 (0.7 to 1.5)
**Sub-Saharan Africa**	Dieticians and nutritionists	0.2 (0.2 to 0.3)

**Sub-Saharan Africa**	Environmental health workers	0.7 (0.6 to 0.8)
**Sub-Saharan Africa**	Healthcare aides, paramedics, and ambulance workers	3.6 (3.1 to 4.3)
**Sub-Saharan Africa**	Home-based personal care workers	3.1 (2.8 to 3.6)
**Sub-Saharan Africa**	Medical imaging and therapeutic equipment technicians	0.4 (0.3 to 0.6)
**Sub-Saharan Africa**	Medical laboratory technicians	3.4 (2.8 to 4.1)
**Sub-Saharan Africa**	Optometrists and opticians	0.4 (0.3 to 0.4)
**Sub-Saharan Africa**	Physiotherapists and prosthetic technicians	1.0 (0.9 to 1.2)
**Sub-Saharan Africa**	Psychologists	0.9 (0.7 to 1.2)
**Sub-Saharan Africa**	Traditional and complementary medicine practitioners	2.4 (2.0 to 3.2)
Central sub-Saharan Africa	Audiologists, speech therapists, and counsellors	0.3 (0.2 to 0.4)
Central sub-Saharan Africa	Clinical officers and medical assistants	0.4 (0.3 to 0.7)
Central sub-Saharan Africa	Dieticians and nutritionists	0.3 (0.2 to 0.4)
Central sub-Saharan Africa	Environmental health workers	2.0 (1.7 to 2.2)
Central sub-Saharan Africa	Healthcare aides, paramedics, and ambulance workers	3.5 (2.3 to 5.1)
Central sub-Saharan Africa	Home-based personal care workers	1.0 (0.7 to 1.3)
Central sub-Saharan Africa	Medical imaging and therapeutic equipment technicians	0.2 (0.1 to 0.3)
Central sub-Saharan Africa	Medical laboratory technicians	3.0 (1.9 to 4.4)
Central sub-Saharan Africa	Optometrists and opticians	0.1 (0.0 to 0.1)
Central sub-Saharan Africa	Physiotherapists and prosthetic technicians	0.8 (0.5 to 1.1)
Central sub-Saharan Africa	Psychologists	0.2 (0.1 to 0.3)
Central sub-Saharan Africa	Traditional and complementary medicine practitioners	0.6 (0.4 to 0.8)
Angola	Audiologists, speech therapists, and counsellors	0.4 (0.3 to 0.7)
Angola	Clinical officers and medical assistants	0.5 (0.3 to 0.8)
Angola	Dieticians and nutritionists	0.5 (0.3 to 0.7)
Angola	Environmental health workers	4.9 (3.7 to 6.4)
Angola	Healthcare aides, paramedics, and ambulance workers	10.0 (6.5 to 15.0)
Angola	Home-based personal care workers	2.0 (1.4 to 2.8)
Angola	Medical imaging and therapeutic equipment technicians	0.3 (0.2 to 0.4)
Angola	Medical laboratory technicians	9.1 (5.9 to 13.3)
Angola	Optometrists and opticians	0.1 (0.1 to 0.1)

Angola	Physiotherapists and prosthetic technicians	1.3 (0.9 to 1.8)
Angola	Psychologists	0.5 (0.4 to 0.8)
Angola	Traditional and complementary medicine practitioners	0.4 (0.3 to 0.6)
Central African Republic	Audiologists, speech therapists, and counsellors	0.1 (0.0 to 0.1)
Central African Republic	Clinical officers and medical assistants	0.1 (0.1 to 0.2)
Central African Republic	Dieticians and nutritionists	0.0 (0.0 to 0.1)
Central African Republic	Environmental health workers	0.7 (0.5 to 1.0)
Central African Republic	Healthcare aides, paramedics, and ambulance workers	1.3 (1.0 to 1.8)
Central African Republic	Home-based personal care workers	0.2 (0.1 to 0.2)
Central African Republic	Medical imaging and therapeutic equipment technicians	0.0 (0.0 to 0.0)
Central African Republic	Medical laboratory technicians	0.5 (0.3 to 0.8)
Central African Republic	Optometrists and opticians	0.0 (0.0 to 0.0)
Central African Republic	Physiotherapists and prosthetic technicians	0.2 (0.1 to 0.2)
Central African Republic	Psychologists	0.0 (0.0 to 0.1)
Central African Republic	Traditional and complementary medicine practitioners	0.3 (0.2 to 0.4)
Congo (Brazzaville)	Audiologists, speech therapists, and counsellors	0.3 (0.2 to 0.5)
Congo (Brazzaville)	Clinical officers and medical assistants	0.3 (0.2 to 0.5)
Congo (Brazzaville)	Dieticians and nutritionists	0.3 (0.2 to 0.5)
Congo (Brazzaville)	Environmental health workers	0.8 (0.5 to 1.1)
Congo (Brazzaville)	Healthcare aides, paramedics, and ambulance workers	1.5 (1.0 to 2.3)
Congo (Brazzaville)	Home-based personal care workers	1.4 (1.0 to 1.9)
Congo (Brazzaville)	Medical imaging and therapeutic equipment technicians	0.2 (0.1 to 0.3)
Congo (Brazzaville)	Medical laboratory technicians	1.9 (1.2 to 2.8)
Congo (Brazzaville)	Optometrists and opticians	0.1 (0.1 to 0.2)
Congo (Brazzaville)	Physiotherapists and prosthetic technicians	0.8 (0.5 to 1.1)
Congo (Brazzaville)	Psychologists	0.3 (0.2 to 0.4)
Congo (Brazzaville)	Traditional and complementary medicine practitioners	0.3 (0.2 to 0.5)
DR Congo	Audiologists, speech therapists, and counsellors	0.2 (0.1 to 0.3)
DR Congo	Clinical officers and medical assistants	0.4 (0.2 to 0.6)
DR Congo	Dieticians and nutritionists	0.2 (0.1 to 0.3)

DR Congo	Environmental health workers	1.0 (0.7 to 1.4)
DR Congo	Healthcare aides, paramedics, and ambulance workers	1.3 (0.9 to 1.9)
DR Congo	Home-based personal care workers	0.5 (0.3 to 0.7)
DR Congo	Medical imaging and therapeutic equipment technicians	0.1 (0.1 to 0.2)
DR Congo	Medical laboratory technicians	0.8 (0.5 to 1.2)
DR Congo	Optometrists and opticians	0.1 (0.0 to 0.1)
DR Congo	Physiotherapists and prosthetic technicians	0.5 (0.4 to 0.7)
DR Congo	Psychologists	0.1 (0.0 to 0.1)
DR Congo	Traditional and complementary medicine practitioners	0.7 (0.4 to 1.0)
Equatorial Guinea	Audiologists, speech therapists, and counsellors	1.0 (0.6 to 1.5)
Equatorial Guinea	Clinical officers and medical assistants	0.7 (0.4 to 1.2)
Equatorial Guinea	Dieticians and nutritionists	1.1 (0.7 to 1.7)
Equatorial Guinea	Environmental health workers	2.1 (1.4 to 3.1)
Equatorial Guinea	Healthcare aides, paramedics, and ambulance workers	5.5 (3.6 to 8.4)
Equatorial Guinea	Home-based personal care workers	4.2 (2.9 to 5.6)
Equatorial Guinea	Medical imaging and therapeutic equipment technicians	0.7 (0.4 to 1.1)
Equatorial Guinea	Medical laboratory technicians	6.0 (3.9 to 8.8)
Equatorial Guinea	Optometrists and opticians	0.4 (0.3 to 0.7)
Equatorial Guinea	Physiotherapists and prosthetic technicians	2.6 (1.7 to 3.7)
Equatorial Guinea	Psychologists	1.1 (0.7 to 1.7)
Equatorial Guinea	Traditional and complementary medicine practitioners	0.6 (0.4 to 0.9)
Gabon	Audiologists, speech therapists, and counsellors	0.9 (0.6 to 1.4)
Gabon	Clinical officers and medical assistants	0.7 (0.4 to 1.2)
Gabon	Dieticians and nutritionists	1.0 (0.6 to 1.5)
Gabon	Environmental health workers	2.0 (1.3 to 3.0)
Gabon	Healthcare aides, paramedics, and ambulance workers	4.7 (3.1 to 7.2)
Gabon	Home-based personal care workers	4.0 (2.8 to 5.4)
Gabon	Medical imaging and therapeutic equipment technicians	0.6 (0.4 to 1.0)
Gabon	Medical laboratory technicians	5.5 (3.6 to 8.1)
Gabon	Optometrists and opticians	0.4 (0.3 to 0.6)

Gabon	Physiotherapists and prosthetic technicians	2.4 (1.5 to 3.4)
Gabon	Psychologists	0.9 (0.6 to 1.4)
Gabon	Traditional and complementary medicine practitioners	0.7 (0.4 to 1.0)
Eastern sub-Saharan Africa	Audiologists, speech therapists, and counsellors	0.6 (0.4 to 0.8)
Eastern sub-Saharan Africa	Clinical officers and medical assistants	0.7 (0.5 to 1.1)
Eastern sub-Saharan Africa	Dieticians and nutritionists	0.2 (0.1 to 0.3)
Eastern sub-Saharan Africa	Environmental health workers	0.2 (0.1 to 0.3)
Eastern sub-Saharan Africa	Healthcare aides, paramedics, and ambulance workers	1.6 (1.1 to 2.2)
Eastern sub-Saharan Africa	Home-based personal care workers	2.9 (2.2 to 3.9)
Eastern sub-Saharan Africa	Medical imaging and therapeutic equipment technicians	0.3 (0.2 to 0.4)
Eastern sub-Saharan Africa	Medical laboratory technicians	1.0 (0.8 to 1.4)
Eastern sub-Saharan Africa	Optometrists and opticians	0.1 (0.1 to 0.1)
Eastern sub-Saharan Africa	Physiotherapists and prosthetic technicians	0.7 (0.5 to 0.9)
Eastern sub-Saharan Africa	Psychologists	0.2 (0.2 to 0.3)
Eastern sub-Saharan Africa	Traditional and complementary medicine practitioners	2.5 (1.7 to 3.4)
Burundi	Audiologists, speech therapists, and counsellors	0.1 (0.0 to 0.1)
Burundi	Clinical officers and medical assistants	0.1 (0.1 to 0.2)
Burundi	Dieticians and nutritionists	0.0 (0.0 to 0.0)
Burundi	Environmental health workers	0.0 (0.0 to 0.1)
Burundi	Healthcare aides, paramedics, and ambulance workers	0.4 (0.2 to 0.7)
Burundi	Home-based personal care workers	0.2 (0.1 to 0.3)
Burundi	Medical imaging and therapeutic equipment technicians	0.0 (0.0 to 0.0)
Burundi	Medical laboratory technicians	1.9 (1.4 to 2.6)
Burundi	Optometrists and opticians	0.0 (0.0 to 0.0)
Burundi	Physiotherapists and prosthetic technicians	2.0 (1.4 to 2.6)
Burundi	Psychologists	0.0 (0.0 to 0.0)
Burundi	Traditional and complementary medicine practitioners	1.9 (1.4 to 2.5)
Comoros	Audiologists, speech therapists, and counsellors	0.4 (0.2 to 0.5)
Comoros	Clinical officers and medical assistants	0.3 (0.2 to 0.6)
Comoros	Dieticians and nutritionists	0.1 (0.1 to 0.2)

Comoros	Environmental health workers	0.1 (0.1 to 0.1)
Comoros	Healthcare aides, paramedics, and ambulance workers	0.6 (0.4 to 0.9)
Comoros	Home-based personal care workers	1.6 (1.2 to 2.1)
Comoros	Medical imaging and therapeutic equipment technicians	0.1 (0.1 to 0.2)
Comoros	Medical laboratory technicians	0.3 (0.2 to 0.5)
Comoros	Optometrists and opticians	0.1 (0.1 to 0.1)
Comoros	Physiotherapists and prosthetic technicians	0.4 (0.2 to 0.5)
Comoros	Psychologists	0.1 (0.1 to 0.2)
Comoros	Traditional and complementary medicine practitioners	1.2 (0.8 to 1.7)
Djibouti	Audiologists, speech therapists, and counsellors	0.3 (0.2 to 0.4)
Djibouti	Clinical officers and medical assistants	0.2 (0.1 to 0.4)
Djibouti	Dieticians and nutritionists	0.1 (0.0 to 0.1)
Djibouti	Environmental health workers	0.0 (0.0 to 0.1)
Djibouti	Healthcare aides, paramedics, and ambulance workers	0.2 (0.1 to 0.3)
Djibouti	Home-based personal care workers	1.4 (1.0 to 1.8)
Djibouti	Medical imaging and therapeutic equipment technicians	0.1 (0.1 to 0.1)
Djibouti	Medical laboratory technicians	0.2 (0.1 to 0.3)
Djibouti	Optometrists and opticians	0.1 (0.0 to 0.1)
Djibouti	Physiotherapists and prosthetic technicians	0.2 (0.1 to 0.3)
Djibouti	Psychologists	0.1 (0.0 to 0.1)
Djibouti	Traditional and complementary medicine practitioners	1.2 (0.8 to 1.6)
Eritrea	Audiologists, speech therapists, and counsellors	0.4 (0.2 to 0.6)
Eritrea	Clinical officers and medical assistants	0.5 (0.3 to 0.8)
Eritrea	Dieticians and nutritionists	0.1 (0.1 to 0.2)
Eritrea	Environmental health workers	0.1 (0.1 to 0.2)
Eritrea	Healthcare aides, paramedics, and ambulance workers	1.0 (0.7 to 1.5)
Eritrea	Home-based personal care workers	1.5 (1.1 to 2.0)
Eritrea	Medical imaging and therapeutic equipment technicians	0.1 (0.1 to 0.2)
Eritrea	Medical laboratory technicians	0.4 (0.3 to 0.7)
Eritrea	Optometrists and opticians	0.1 (0.0 to 0.1)

Eritrea	Physiotherapists and prosthetic technicians	0.5 (0.3 to 0.6)
Eritrea	Psychologists	0.1 (0.1 to 0.2)
Eritrea	Traditional and complementary medicine practitioners	1.4 (1.0 to 2.0)
Ethiopia	Audiologists, speech therapists, and counsellors	0.3 (0.2 to 0.4)
Ethiopia	Clinical officers and medical assistants	0.3 (0.2 to 0.6)
Ethiopia	Dieticians and nutritionists	0.1 (0.0 to 0.1)
Ethiopia	Environmental health workers	0.1 (0.1 to 0.2)
Ethiopia	Healthcare aides, paramedics, and ambulance workers	1.0 (0.7 to 1.5)
Ethiopia	Home-based personal care workers	0.9 (0.6 to 1.2)
Ethiopia	Medical imaging and therapeutic equipment technicians	0.1 (0.1 to 0.2)
Ethiopia	Medical laboratory technicians	0.3 (0.2 to 0.4)
Ethiopia	Optometrists and opticians	0.0 (0.0 to 0.1)
Ethiopia	Physiotherapists and prosthetic technicians	0.3 (0.2 to 0.4)
Ethiopia	Psychologists	0.1 (0.1 to 0.1)
Ethiopia	Traditional and complementary medicine practitioners	1.0 (0.6 to 1.4)
Kenya	Audiologists, speech therapists, and counsellors	0.7 (0.4 to 1.0)
Kenya	Clinical officers and medical assistants	0.8 (0.5 to 1.4)
Kenya	Dieticians and nutritionists	0.3 (0.2 to 0.4)
Kenya	Environmental health workers	0.2 (0.1 to 0.2)
Kenya	Healthcare aides, paramedics, and ambulance workers	1.3 (0.8 to 2.0)
Kenya	Home-based personal care workers	2.8 (2.0 to 3.9)
Kenya	Medical imaging and therapeutic equipment technicians	1.1 (0.7 to 1.7)
Kenya	Medical laboratory technicians	3.5 (2.4 to 4.9)
Kenya	Optometrists and opticians	0.2 (0.1 to 0.3)
Kenya	Physiotherapists and prosthetic technicians	0.8 (0.5 to 1.1)
Kenya	Psychologists	0.6 (0.4 to 1.0)
Kenya	Traditional and complementary medicine practitioners	1.2 (0.8 to 1.8)
Madagascar	Audiologists, speech therapists, and counsellors	0.7 (0.5 to 1.1)
Madagascar	Clinical officers and medical assistants	1.0 (0.6 to 1.7)
Madagascar	Dieticians and nutritionists	0.3 (0.2 to 0.4)

Madagascar	Environmental health workers	0.4 (0.3 to 0.7)
Madagascar	Healthcare aides, paramedics, and ambulance workers	4.5 (3.2 to 6.4)
Madagascar	Home-based personal care workers	2.2 (1.5 to 3.1)
Madagascar	Medical imaging and therapeutic equipment technicians	0.3 (0.1 to 0.4)
Madagascar	Medical laboratory technicians	1.0 (0.6 to 1.5)
Madagascar	Optometrists and opticians	0.1 (0.1 to 0.2)
Madagascar	Physiotherapists and prosthetic technicians	1.0 (0.6 to 1.4)
Madagascar	Psychologists	0.3 (0.2 to 0.5)
Madagascar	Traditional and complementary medicine practitioners	2.0 (1.3 to 2.9)
Malawi	Audiologists, speech therapists, and counsellors	0.5 (0.3 to 0.7)
Malawi	Clinical officers and medical assistants	0.6 (0.4 to 1.0)
Malawi	Dieticians and nutritionists	0.1 (0.1 to 0.2)
Malawi	Environmental health workers	0.2 (0.1 to 0.3)
Malawi	Healthcare aides, paramedics, and ambulance workers	1.7 (1.2 to 2.5)
Malawi	Home-based personal care workers	1.6 (1.1 to 2.2)
Malawi	Medical imaging and therapeutic equipment technicians	0.2 (0.1 to 0.2)
Malawi	Medical laboratory technicians	0.5 (0.3 to 0.8)
Malawi	Optometrists and opticians	0.1 (0.0 to 0.1)
Malawi	Physiotherapists and prosthetic technicians	0.5 (0.3 to 0.8)
Malawi	Psychologists	0.2 (0.1 to 0.2)
Malawi	Traditional and complementary medicine practitioners	1.6 (1.1 to 2.3)
Mozambique	Audiologists, speech therapists, and counsellors	0.4 (0.3 to 0.6)
Mozambique	Clinical officers and medical assistants	0.8 (0.5 to 1.2)
Mozambique	Dieticians and nutritionists	0.2 (0.2 to 0.4)
Mozambique	Environmental health workers	0.4 (0.2 to 0.5)
Mozambique	Healthcare aides, paramedics, and ambulance workers	2.3 (1.4 to 3.3)
Mozambique	Home-based personal care workers	1.2 (0.9 to 1.7)
Mozambique	Medical imaging and therapeutic equipment technicians	0.3 (0.2 to 0.4)
Mozambique	Medical laboratory technicians	0.4 (0.3 to 0.7)
Mozambique	Optometrists and opticians	0.1 (0.1 to 0.1)

Mozambique	Physiotherapists and prosthetic technicians	0.5 (0.3 to 0.7)
Mozambique	Psychologists	0.2 (0.1 to 0.3)
Mozambique	Traditional and complementary medicine practitioners	3.1 (2.2 to 4.6)
Rwanda	Audiologists, speech therapists, and counsellors	0.3 (0.2 to 0.4)
Rwanda	Clinical officers and medical assistants	1.9 (1.3 to 2.7)
Rwanda	Dieticians and nutritionists	0.2 (0.2 to 0.3)
Rwanda	Environmental health workers	0.1 (0.1 to 0.1)
Rwanda	Healthcare aides, paramedics, and ambulance workers	1.1 (0.7 to 1.6)
Rwanda	Home-based personal care workers	1.2 (0.8 to 1.6)
Rwanda	Medical imaging and therapeutic equipment technicians	0.3 (0.2 to 0.4)
Rwanda	Medical laboratory technicians	1.5 (1.1 to 2.1)
Rwanda	Optometrists and opticians	0.1 (0.1 to 0.1)
Rwanda	Physiotherapists and prosthetic technicians	0.7 (0.5 to 1.0)
Rwanda	Psychologists	0.1 (0.1 to 0.2)
Rwanda	Traditional and complementary medicine practitioners	1.1 (0.7 to 1.6)
Somalia	Audiologists, speech therapists, and counsellors	0.1 (0.0 to 0.1)
Somalia	Clinical officers and medical assistants	0.1 (0.1 to 0.2)
Somalia	Dieticians and nutritionists	0.0 (0.0 to 0.0)
Somalia	Environmental health workers	0.0 (0.0 to 0.1)
Somalia	Healthcare aides, paramedics, and ambulance workers	0.2 (0.1 to 0.2)
Somalia	Home-based personal care workers	0.2 (0.2 to 0.4)
Somalia	Medical imaging and therapeutic equipment technicians	0.0 (0.0 to 0.0)
Somalia	Medical laboratory technicians	0.1 (0.0 to 0.1)
Somalia	Optometrists and opticians	0.0 (0.0 to 0.0)
Somalia	Physiotherapists and prosthetic technicians	0.1 (0.0 to 0.1)
Somalia	Psychologists	0.0 (0.0 to 0.0)
Somalia	Traditional and complementary medicine practitioners	1.2 (0.9 to 1.5)
South Sudan	Audiologists, speech therapists, and counsellors	0.3 (0.2 to 0.4)
South Sudan	Clinical officers and medical assistants	0.4 (0.2 to 0.6)
South Sudan	Dieticians and nutritionists	0.1 (0.1 to 0.1)

South Sudan	Environmental health workers	0.1 (0.1 to 0.2)
South Sudan	Healthcare aides, paramedics, and ambulance workers	0.8 (0.6 to 1.2)
South Sudan	Home-based personal care workers	1.1 (0.8 to 1.5)
South Sudan	Medical imaging and therapeutic equipment technicians	0.1 (0.1 to 0.2)
South Sudan	Medical laboratory technicians	0.3 (0.2 to 0.5)
South Sudan	Optometrists and opticians	0.0 (0.0 to 0.1)
South Sudan	Physiotherapists and prosthetic technicians	0.3 (0.2 to 0.5)
South Sudan	Psychologists	0.1 (0.1 to 0.1)
South Sudan	Traditional and complementary medicine practitioners	1.4 (0.9 to 1.9)
Uganda	Audiologists, speech therapists, and counsellors	0.9 (0.6 to 1.4)
Uganda	Clinical officers and medical assistants	1.4 (0.9 to 2.0)
Uganda	Dieticians and nutritionists	0.2 (0.2 to 0.3)
Uganda	Environmental health workers	0.4 (0.2 to 0.5)
Uganda	Healthcare aides, paramedics, and ambulance workers	0.8 (0.5 to 1.2)
Uganda	Home-based personal care workers	2.8 (2.0 to 3.9)
Uganda	Medical imaging and therapeutic equipment technicians	0.3 (0.2 to 0.5)
Uganda	Medical laboratory technicians	2.2 (1.4 to 3.4)
Uganda	Optometrists and opticians	0.2 (0.1 to 0.3)
Uganda	Physiotherapists and prosthetic technicians	1.7 (1.1 to 2.4)
Uganda	Psychologists	0.3 (0.2 to 0.4)
Uganda	Traditional and complementary medicine practitioners	5.3 (3.7 to 7.6)
Tanzania	Audiologists, speech therapists, and counsellors	0.8 (0.5 to 1.2)
Tanzania	Clinical officers and medical assistants	0.8 (0.5 to 1.1)
Tanzania	Dieticians and nutritionists	0.1 (0.1 to 0.2)
Tanzania	Environmental health workers	0.2 (0.1 to 0.2)
Tanzania	Healthcare aides, paramedics, and ambulance workers	1.8 (1.2 to 2.7)
Tanzania	Home-based personal care workers	9.7 (7.1 to 12.9)
Tanzania	Medical imaging and therapeutic equipment technicians	0.2 (0.1 to 0.3)
Tanzania	Medical laboratory technicians	0.3 (0.2 to 0.4)
Tanzania	Optometrists and opticians	0.2 (0.1 to 0.3)

Tanzania	Physiotherapists and prosthetic technicians	0.4 (0.3 to 0.5)
Tanzania	Psychologists	0.3 (0.2 to 0.4)
Tanzania	Traditional and complementary medicine practitioners	5.4 (3.7 to 7.8)
Zambia	Audiologists, speech therapists, and counsellors	2.0 (1.3 to 2.9)
Zambia	Clinical officers and medical assistants	0.8 (0.5 to 1.2)
Zambia	Dieticians and nutritionists	0.6 (0.4 to 0.9)
Zambia	Environmental health workers	0.4 (0.3 to 0.5)
Zambia	Healthcare aides, paramedics, and ambulance workers	3.4 (2.1 to 4.9)
Zambia	Home-based personal care workers	5.3 (3.9 to 7.0)
Zambia	Medical imaging and therapeutic equipment technicians	0.4 (0.3 to 0.6)
Zambia	Medical laboratory technicians	0.7 (0.5 to 1.1)
Zambia	Optometrists and opticians	0.2 (0.1 to 0.3)
Zambia	Physiotherapists and prosthetic technicians	1.3 (0.9 to 1.8)
Zambia	Psychologists	0.4 (0.3 to 0.6)
Zambia	Traditional and complementary medicine practitioners	1.8 (1.2 to 2.6)
Southern sub-Saharan Africa	Audiologists, speech therapists, and counsellors	1.1 (0.8 to 1.4)
Southern sub-Saharan Africa	Clinical officers and medical assistants	1.3 (0.8 to 1.9)
Southern sub-Saharan Africa	Dieticians and nutritionists	0.5 (0.4 to 0.6)
Southern sub-Saharan Africa	Environmental health workers	0.7 (0.6 to 0.9)
Southern sub-Saharan Africa	Healthcare aides, paramedics, and ambulance workers	14.9 (11.7 to 18.7)
Southern sub-Saharan Africa	Home-based personal care workers	15.3 (13.1 to 18.2)
Southern sub-Saharan Africa	Medical imaging and therapeutic equipment technicians	0.4 (0.3 to 0.5)
Southern sub-Saharan Africa	Medical laboratory technicians	1.7 (1.1 to 2.3)
Southern sub-Saharan Africa	Optometrists and opticians	0.8 (0.6 to 1.1)
Southern sub-Saharan Africa	Physiotherapists and prosthetic technicians	3.1 (2.6 to 4.0)
Southern sub-Saharan Africa	Psychologists	1.7 (1.4 to 2.1)
Southern sub-Saharan Africa	Traditional and complementary medicine practitioners	6.2 (4.7 to 8.2)
Botswana	Audiologists, speech therapists, and counsellors	0.6 (0.4 to 0.9)
Botswana	Clinical officers and medical assistants	2.5 (1.5 to 3.8)
Botswana	Dieticians and nutritionists	0.3 (0.2 to 0.4)

Botswana	Environmental health workers	0.4 (0.3 to 0.6)
Botswana	Healthcare aides, paramedics, and ambulance workers	2.6 (1.6 to 3.9)
Botswana	Home-based personal care workers	1.4 (1.0 to 1.9)
Botswana	Medical imaging and therapeutic equipment technicians	0.3 (0.2 to 0.4)
Botswana	Medical laboratory technicians	5.7 (3.6 to 8.3)
Botswana	Optometrists and opticians	0.4 (0.3 to 0.6)
Botswana	Physiotherapists and prosthetic technicians	1.6 (1.1 to 2.3)
Botswana	Psychologists	0.7 (0.4 to 0.9)
Botswana	Traditional and complementary medicine practitioners	7.8 (5.3 to 11.4)
Eswatini	Audiologists, speech therapists, and counsellors	0.2 (0.1 to 0.3)
Eswatini	Clinical officers and medical assistants	0.2 (0.1 to 0.3)
Eswatini	Dieticians and nutritionists	0.1 (0.0 to 0.1)
Eswatini	Environmental health workers	0.1 (0.1 to 0.2)
Eswatini	Healthcare aides, paramedics, and ambulance workers	1.7 (1.1 to 2.4)
Eswatini	Home-based personal care workers	1.2 (0.8 to 1.7)
Eswatini	Medical imaging and therapeutic equipment technicians	0.1 (0.0 to 0.1)
Eswatini	Medical laboratory technicians	0.3 (0.2 to 0.4)
Eswatini	Optometrists and opticians	0.1 (0.1 to 0.2)
Eswatini	Physiotherapists and prosthetic technicians	0.5 (0.3 to 0.7)
Eswatini	Psychologists	0.3 (0.2 to 0.4)
Eswatini	Traditional and complementary medicine practitioners	1.8 (1.2 to 2.5)
Lesotho	Audiologists, speech therapists, and counsellors	0.2 (0.1 to 0.3)
Lesotho	Clinical officers and medical assistants	0.2 (0.1 to 0.4)
Lesotho	Dieticians and nutritionists	0.1 (0.0 to 0.1)
Lesotho	Environmental health workers	0.2 (0.1 to 0.3)
Lesotho	Healthcare aides, paramedics, and ambulance workers	2.6 (1.9 to 3.5)
Lesotho	Home-based personal care workers	0.8 (0.5 to 1.1)
Lesotho	Medical imaging and therapeutic equipment technicians	0.1 (0.0 to 0.1)
Lesotho	Medical laboratory technicians	0.3 (0.1 to 0.4)
Lesotho	Optometrists and opticians	0.1 (0.0 to 0.1)

Lesotho	Physiotherapists and prosthetic technicians	0.4 (0.3 to 0.6)
Lesotho	Psychologists	0.2 (0.1 to 0.3)
Lesotho	Traditional and complementary medicine practitioners	1.7 (1.1 to 2.4)
Namibia	Audiologists, speech therapists, and counsellors	4.4 (3.1 to 5.9)
Namibia	Clinical officers and medical assistants	1.1 (0.7 to 1.6)
Namibia	Dieticians and nutritionists	0.7 (0.5 to 1.1)
Namibia	Environmental health workers	1.6 (1.1 to 2.2)
Namibia	Healthcare aides, paramedics, and ambulance workers	30.5 (22.3 to 40.0)
Namibia	Home-based personal care workers	4.6 (3.2 to 6.3)
Namibia	Medical imaging and therapeutic equipment technicians	0.3 (0.2 to 0.5)
Namibia	Medical laboratory technicians	0.5 (0.3 to 0.7)
Namibia	Optometrists and opticians	1.1 (0.8 to 1.4)
Namibia	Physiotherapists and prosthetic technicians	1.0 (0.7 to 1.4)
Namibia	Psychologists	2.7 (1.8 to 3.7)
Namibia	Traditional and complementary medicine practitioners	1.2 (0.8 to 1.9)
South Africa	Audiologists, speech therapists, and counsellors	1.2 (0.9 to 1.7)
South Africa	Clinical officers and medical assistants	1.4 (0.9 to 2.1)
South Africa	Dieticians and nutritionists	0.6 (0.4 to 0.8)
South Africa	Environmental health workers	0.8 (0.6 to 1.1)
South Africa	Healthcare aides, paramedics, and ambulance workers	18.5 (14.3 to 23.4)
South Africa	Home-based personal care workers	20.7 (17.6 to 24.8)
South Africa	Medical imaging and therapeutic equipment technicians	0.5 (0.3 to 0.7)
South Africa	Medical laboratory technicians	1.8 (1.3 to 2.6)
South Africa	Optometrists and opticians	1.0 (0.8 to 1.4)
South Africa	Physiotherapists and prosthetic technicians	4.1 (3.2 to 5.3)
South Africa	Psychologists	2.0 (1.4 to 2.6)
South Africa	Traditional and complementary medicine practitioners	7.8 (5.5 to 10.6)
Zimbabwe	Audiologists, speech therapists, and counsellors	0.2 (0.1 to 0.3)
Zimbabwe	Clinical officers and medical assistants	0.9 (0.5 to 1.4)
Zimbabwe	Dieticians and nutritionists	0.1 (0.0 to 0.1)

Zimbabwe	Environmental health workers	0.3 (0.2 to 0.4)
Zimbabwe	Healthcare aides, paramedics, and ambulance workers	1.9 (1.3 to 2.8)
Zimbabwe	Home-based personal care workers	0.7 (0.5 to 1.0)
Zimbabwe	Medical imaging and therapeutic equipment technicians	0.1 (0.0 to 0.1)
Zimbabwe	Medical laboratory technicians	0.9 (0.6 to 1.4)
Zimbabwe	Optometrists and opticians	0.1 (0.1 to 0.1)
Zimbabwe	Physiotherapists and prosthetic technicians	0.5 (0.3 to 0.8)
Zimbabwe	Psychologists	0.8 (0.5 to 1.1)
Zimbabwe	Traditional and complementary medicine practitioners	1.4 (0.9 to 1.9)
Western sub-Saharan Africa	Audiologists, speech therapists, and counsellors	2.6 (1.8 to 3.5)
Western sub-Saharan Africa	Clinical officers and medical assistants	1.5 (1.0 to 2.2)
Western sub-Saharan Africa	Dieticians and nutritionists	0.2 (0.2 to 0.2)
Western sub-Saharan Africa	Environmental health workers	0.7 (0.5 to 0.9)
Western sub-Saharan Africa	Healthcare aides, paramedics, and ambulance workers	3.4 (2.5 to 4.7)
Western sub-Saharan Africa	Home-based personal care workers	1.8 (1.5 to 2.2)
Western sub-Saharan Africa	Medical imaging and therapeutic equipment technicians	0.6 (0.4 to 0.8)
Western sub-Saharan Africa	Medical laboratory technicians	5.8 (4.1 to 7.7)
Western sub-Saharan Africa	Optometrists and opticians	0.6 (0.5 to 0.8)
Western sub-Saharan Africa	Physiotherapists and prosthetic technicians	1.0 (0.8 to 1.3)
Western sub-Saharan Africa	Psychologists	1.5 (1.0 to 2.1)
Western sub-Saharan Africa	Traditional and complementary medicine practitioners	2.3 (1.7 to 3.2)
Benin	Audiologists, speech therapists, and counsellors	0.0 (0.0 to 0.0)
Benin	Clinical officers and medical assistants	1.1 (0.7 to 1.5)
Benin	Dieticians and nutritionists	1.2 (0.9 to 1.7)
Benin	Environmental health workers	0.8 (0.5 to 1.1)
Benin	Healthcare aides, paramedics, and ambulance workers	0.1 (0.0 to 0.1)
Benin	Home-based personal care workers	0.0 (0.0 to 0.1)
Benin	Medical imaging and therapeutic equipment technicians	0.0 (0.0 to 0.0)
Benin	Medical laboratory technicians	0.0 (0.0 to 0.0)
Benin	Optometrists and opticians	0.0 (0.0 to 0.0)

Benin	Physiotherapists and prosthetic technicians	0.0 (0.0 to 0.0)
Benin	Psychologists	0.1 (0.0 to 0.1)
Benin	Traditional and complementary medicine practitioners	1.2 (0.9 to 1.6)
Burkina Faso	Audiologists, speech therapists, and counsellors	0.3 (0.2 to 0.4)
Burkina Faso	Clinical officers and medical assistants	0.5 (0.3 to 0.9)
Burkina Faso	Dieticians and nutritionists	0.1 (0.0 to 0.1)
Burkina Faso	Environmental health workers	0.3 (0.2 to 0.5)
Burkina Faso	Healthcare aides, paramedics, and ambulance workers	0.9 (0.6 to 1.3)
Burkina Faso	Home-based personal care workers	0.4 (0.3 to 0.6)
Burkina Faso	Medical imaging and therapeutic equipment technicians	0.1 (0.1 to 0.2)
Burkina Faso	Medical laboratory technicians	0.4 (0.2 to 0.6)
Burkina Faso	Optometrists and opticians	0.0 (0.0 to 0.0)
Burkina Faso	Physiotherapists and prosthetic technicians	0.1 (0.1 to 0.2)
Burkina Faso	Psychologists	0.2 (0.1 to 0.3)
Burkina Faso	Traditional and complementary medicine practitioners	1.7 (1.2 to 2.3)
Cabo Verde	Audiologists, speech therapists, and counsellors	1.2 (0.7 to 1.8)
Cabo Verde	Clinical officers and medical assistants	1.0 (0.6 to 1.6)
Cabo Verde	Dieticians and nutritionists	0.4 (0.2 to 0.6)
Cabo Verde	Environmental health workers	0.5 (0.3 to 0.7)
Cabo Verde	Healthcare aides, paramedics, and ambulance workers	2.1 (1.4 to 3.3)
Cabo Verde	Home-based personal care workers	2.0 (1.4 to 2.8)
Cabo Verde	Medical imaging and therapeutic equipment technicians	0.5 (0.3 to 0.8)
Cabo Verde	Medical laboratory technicians	1.3 (0.8 to 2.0)
Cabo Verde	Optometrists and opticians	0.3 (0.2 to 0.5)
Cabo Verde	Physiotherapists and prosthetic technicians	0.6 (0.4 to 0.8)
Cabo Verde	Psychologists	1.5 (1.0 to 2.1)
Cabo Verde	Traditional and complementary medicine practitioners	1.4 (0.9 to 2.1)
Cameroon	Audiologists, speech therapists, and counsellors	0.5 (0.3 to 0.7)
Cameroon	Clinical officers and medical assistants	0.7 (0.4 to 1.1)
Cameroon	Dieticians and nutritionists	0.3 (0.2 to 0.4)

Cameroon	Environmental health workers	0.3 (0.2 to 0.5)
Cameroon	Healthcare aides, paramedics, and ambulance workers	2.0 (1.3 to 2.9)
Cameroon	Home-based personal care workers	1.5 (1.1 to 2.1)
Cameroon	Medical imaging and therapeutic equipment technicians	1.3 (0.9 to 1.9)
Cameroon	Medical laboratory technicians	2.2 (1.4 to 3.3)
Cameroon	Optometrists and opticians	0.1 (0.1 to 0.2)
Cameroon	Physiotherapists and prosthetic technicians	0.4 (0.3 to 0.6)
Cameroon	Psychologists	2.0 (1.4 to 2.8)
Cameroon	Traditional and complementary medicine practitioners	4.5 (2.8 to 6.9)
Chad	Audiologists, speech therapists, and counsellors	0.1 (0.1 to 0.1)
Chad	Clinical officers and medical assistants	0.2 (0.1 to 0.4)
Chad	Dieticians and nutritionists	0.0 (0.0 to 0.0)
Chad	Environmental health workers	0.2 (0.1 to 0.2)
Chad	Healthcare aides, paramedics, and ambulance workers	0.4 (0.3 to 0.6)
Chad	Home-based personal care workers	0.1 (0.1 to 0.2)
Chad	Medical imaging and therapeutic equipment technicians	0.0 (0.0 to 0.0)
Chad	Medical laboratory technicians	0.1 (0.1 to 0.2)
Chad	Optometrists and opticians	0.0 (0.0 to 0.0)
Chad	Physiotherapists and prosthetic technicians	0.0 (0.0 to 0.1)
Chad	Psychologists	0.1 (0.0 to 0.1)
Chad	Traditional and complementary medicine practitioners	0.8 (0.6 to 1.1)
Côte d'Ivoire	Audiologists, speech therapists, and counsellors	0.4 (0.3 to 0.7)
Côte d'Ivoire	Clinical officers and medical assistants	0.2 (0.1 to 0.3)
Côte d'Ivoire	Dieticians and nutritionists	0.1 (0.1 to 0.2)
Côte d'Ivoire	Environmental health workers	2.0 (1.6 to 2.5)
Côte d'Ivoire	Healthcare aides, paramedics, and ambulance workers	1.3 (0.9 to 1.9)
Côte d'Ivoire	Home-based personal care workers	0.4 (0.3 to 0.5)
Côte d'Ivoire	Medical imaging and therapeutic equipment technicians	0.2 (0.1 to 0.3)
Côte d'Ivoire	Medical laboratory technicians	0.8 (0.5 to 1.1)
Côte d'Ivoire	Optometrists and opticians	0.1 (0.1 to 0.1)

Côte d'Ivoire	Physiotherapists and prosthetic technicians	0.2 (0.1 to 0.3)
Côte d'Ivoire	Psychologists	0.5 (0.3 to 0.7)
Côte d'Ivoire	Traditional and complementary medicine practitioners	1.1 (0.8 to 1.4)
The Gambia	Audiologists, speech therapists, and counsellors	0.8 (0.5 to 1.1)
The Gambia	Clinical officers and medical assistants	1.0 (0.6 to 1.7)
The Gambia	Dieticians and nutritionists	0.2 (0.1 to 0.3)
The Gambia	Environmental health workers	0.6 (0.4 to 0.9)
The Gambia	Healthcare aides, paramedics, and ambulance workers	2.2 (1.5 to 3.2)
The Gambia	Home-based personal care workers	1.1 (0.7 to 1.6)
The Gambia	Medical imaging and therapeutic equipment technicians	0.3 (0.2 to 0.5)
The Gambia	Medical laboratory technicians	0.8 (0.5 to 1.3)
The Gambia	Optometrists and opticians	0.1 (0.1 to 0.1)
The Gambia	Physiotherapists and prosthetic technicians	0.3 (0.2 to 0.5)
The Gambia	Psychologists	0.6 (0.4 to 0.8)
The Gambia	Traditional and complementary medicine practitioners	2.3 (1.5 to 3.1)
Ghana	Audiologists, speech therapists, and counsellors	1.0 (0.7 to 1.5)
Ghana	Clinical officers and medical assistants	1.4 (0.9 to 2.0)
Ghana	Dieticians and nutritionists	0.3 (0.2 to 0.5)
Ghana	Environmental health workers	0.9 (0.6 to 1.2)
Ghana	Healthcare aides, paramedics, and ambulance workers	4.3 (2.8 to 6.0)
Ghana	Home-based personal care workers	7.1 (5.3 to 9.2)
Ghana	Medical imaging and therapeutic equipment technicians	1.0 (0.6 to 1.5)
Ghana	Medical laboratory technicians	3.3 (2.2 to 4.7)
Ghana	Optometrists and opticians	0.2 (0.1 to 0.3)
Ghana	Physiotherapists and prosthetic technicians	0.7 (0.5 to 1.0)
Ghana	Psychologists	2.8 (1.9 to 3.9)
Ghana	Traditional and complementary medicine practitioners	3.3 (2.3 to 4.9)
Guinea	Audiologists, speech therapists, and counsellors	0.1 (0.1 to 0.2)
Guinea	Clinical officers and medical assistants	0.2 (0.2 to 0.4)
Guinea	Dieticians and nutritionists	0.1 (0.0 to 0.1)

Guinea	Environmental health workers	0.1 (0.1 to 0.2)
Guinea	Healthcare aides, paramedics, and ambulance workers	0.2 (0.1 to 0.3)
Guinea	Home-based personal care workers	0.2 (0.1 to 0.3)
Guinea	Medical imaging and therapeutic equipment technicians	0.1 (0.1 to 0.1)
Guinea	Medical laboratory technicians	0.3 (0.2 to 0.5)
Guinea	Optometrists and opticians	0.0 (0.0 to 0.0)
Guinea	Physiotherapists and prosthetic technicians	0.1 (0.1 to 0.1)
Guinea	Psychologists	0.2 (0.1 to 0.2)
Guinea	Traditional and complementary medicine practitioners	0.2 (0.1 to 0.3)
Guinea-Bissau	Audiologists, speech therapists, and counsellors	0.3 (0.2 to 0.4)
Guinea-Bissau	Clinical officers and medical assistants	0.4 (0.3 to 0.7)
Guinea-Bissau	Dieticians and nutritionists	0.1 (0.0 to 0.1)
Guinea-Bissau	Environmental health workers	0.3 (0.2 to 0.4)
Guinea-Bissau	Healthcare aides, paramedics, and ambulance workers	0.8 (0.5 to 1.2)
Guinea-Bissau	Home-based personal care workers	0.4 (0.2 to 0.5)
Guinea-Bissau	Medical imaging and therapeutic equipment technicians	0.1 (0.1 to 0.1)
Guinea-Bissau	Medical laboratory technicians	0.3 (0.2 to 0.5)
Guinea-Bissau	Optometrists and opticians	0.0 (0.0 to 0.1)
Guinea-Bissau	Physiotherapists and prosthetic technicians	0.1 (0.1 to 0.2)
Guinea-Bissau	Psychologists	0.2 (0.1 to 0.3)
Guinea-Bissau	Traditional and complementary medicine practitioners	1.1 (0.7 to 1.5)
Liberia	Audiologists, speech therapists, and counsellors	0.2 (0.1 to 0.3)
Liberia	Clinical officers and medical assistants	0.4 (0.2 to 0.6)
Liberia	Dieticians and nutritionists	0.1 (0.0 to 0.1)
Liberia	Environmental health workers	0.3 (0.2 to 0.4)
Liberia	Healthcare aides, paramedics, and ambulance workers	1.1 (0.7 to 1.5)
Liberia	Home-based personal care workers	0.3 (0.2 to 0.4)
Liberia	Medical imaging and therapeutic equipment technicians	0.1 (0.0 to 0.1)
Liberia	Medical laboratory technicians	0.3 (0.2 to 0.5)
Liberia	Optometrists and opticians	0.0 (0.0 to 0.0)

Liberia	Physiotherapists and prosthetic technicians	0.1 (0.1 to 0.2)
Liberia	Psychologists	0.2 (0.1 to 0.3)
Liberia	Traditional and complementary medicine practitioners	0.7 (0.5 to 1.0)
Mali	Audiologists, speech therapists, and counsellors	0.2 (0.1 to 0.3)
Mali	Clinical officers and medical assistants	0.4 (0.2 to 0.6)
Mali	Dieticians and nutritionists	0.0 (0.0 to 0.1)
Mali	Environmental health workers	0.3 (0.2 to 0.4)
Mali	Healthcare aides, paramedics, and ambulance workers	0.8 (0.5 to 1.1)
Mali	Home-based personal care workers	0.2 (0.1 to 0.3)
Mali	Medical imaging and therapeutic equipment technicians	0.1 (0.0 to 0.1)
Mali	Medical laboratory technicians	0.3 (0.2 to 0.4)
Mali	Optometrists and opticians	0.0 (0.0 to 0.0)
Mali	Physiotherapists and prosthetic technicians	0.1 (0.1 to 0.1)
Mali	Psychologists	0.1 (0.1 to 0.2)
Mali	Traditional and complementary medicine practitioners	0.4 (0.2 to 0.5)
Mauritania	Audiologists, speech therapists, and counsellors	0.4 (0.2 to 0.6)
Mauritania	Clinical officers and medical assistants	0.8 (0.5 to 1.3)
Mauritania	Dieticians and nutritionists	0.1 (0.1 to 0.1)
Mauritania	Environmental health workers	0.2 (0.1 to 0.3)
Mauritania	Healthcare aides, paramedics, and ambulance workers	0.5 (0.3 to 0.7)
Mauritania	Home-based personal care workers	0.7 (0.5 to 0.9)
Mauritania	Medical imaging and therapeutic equipment technicians	0.2 (0.1 to 0.2)
Mauritania	Medical laboratory technicians	0.4 (0.2 to 0.6)
Mauritania	Optometrists and opticians	0.1 (0.1 to 0.1)
Mauritania	Physiotherapists and prosthetic technicians	0.2 (0.1 to 0.2)
Mauritania	Psychologists	0.3 (0.2 to 0.5)
Mauritania	Traditional and complementary medicine practitioners	0.9 (0.6 to 1.3)
Niger	Audiologists, speech therapists, and counsellors	0.1 (0.0 to 0.1)
Niger	Clinical officers and medical assistants	0.2 (0.1 to 0.3)
Niger	Dieticians and nutritionists	0.0 (0.0 to 0.0)

Niger	Environmental health workers	0.1 (0.1 to 0.2)
Niger	Healthcare aides, paramedics, and ambulance workers	0.4 (0.3 to 0.6)
Niger	Home-based personal care workers	0.1 (0.1 to 0.1)
Niger	Medical imaging and therapeutic equipment technicians	0.0 (0.0 to 0.0)
Niger	Medical laboratory technicians	0.1 (0.1 to 0.2)
Niger	Optometrists and opticians	0.0 (0.0 to 0.0)
Niger	Physiotherapists and prosthetic technicians	0.0 (0.0 to 0.1)
Niger	Psychologists	0.1 (0.1 to 0.2)
Niger	Traditional and complementary medicine practitioners	0.7 (0.5 to 0.9)
Nigeria	Audiologists, speech therapists, and counsellors	5.2 (3.6 to 7.0)
Nigeria	Clinical officers and medical assistants	2.6 (1.8 to 3.7)
Nigeria	Dieticians and nutritionists	0.2 (0.2 to 0.3)
Nigeria	Environmental health workers	0.8 (0.5 to 1.0)
Nigeria	Healthcare aides, paramedics, and ambulance workers	5.7 (3.8 to 8.3)
Nigeria	Home-based personal care workers	2.4 (1.8 to 3.2)
Nigeria	Medical imaging and therapeutic equipment technicians	0.8 (0.5 to 1.1)
Nigeria	Medical laboratory technicians	11.3 (7.5 to 15.7)
Nigeria	Optometrists and opticians	1.2 (0.9 to 1.6)
Nigeria	Physiotherapists and prosthetic technicians	1.9 (1.4 to 2.6)
Nigeria	Psychologists	2.3 (1.6 to 3.2)
Nigeria	Traditional and complementary medicine practitioners	3.0 (2.0 to 4.4)
São Tomé and Príncipe	Audiologists, speech therapists, and counsellors	0.2 (0.1 to 0.3)
São Tomé and Príncipe	Clinical officers and medical assistants	0.2 (0.1 to 0.3)
São Tomé and Príncipe	Dieticians and nutritionists	0.0 (0.0 to 0.1)
São Tomé and Príncipe	Environmental health workers	0.1 (0.1 to 0.1)
São Tomé and Príncipe	Healthcare aides, paramedics, and ambulance workers	3.9 (3.1 to 4.9)
São Tomé and Príncipe	Home-based personal care workers	1.6 (1.1 to 2.3)
São Tomé and Príncipe	Medical imaging and therapeutic equipment technicians	0.2 (0.1 to 0.3)
São Tomé and Príncipe	Medical laboratory technicians	0.2 (0.1 to 0.3)
São Tomé and Príncipe	Optometrists and opticians	0.0 (0.0 to 0.0)

São Tomé and Príncipe	Physiotherapists and prosthetic technicians	0.1 (0.0 to 0.1)
São Tomé and Príncipe	Psychologists	0.1 (0.1 to 0.2)
São Tomé and Príncipe	Traditional and complementary medicine practitioners	0.2 (0.1 to 0.4)
Senegal	Audiologists, speech therapists, and counsellors	0.3 (0.2 to 0.4)
Senegal	Clinical officers and medical assistants	0.5 (0.3 to 0.7)
Senegal	Dieticians and nutritionists	0.1 (0.1 to 0.2)
Senegal	Environmental health workers	0.2 (0.1 to 0.3)
Senegal	Healthcare aides, paramedics, and ambulance workers	0.9 (0.6 to 1.3)
Senegal	Home-based personal care workers	0.5 (0.3 to 0.7)
Senegal	Medical imaging and therapeutic equipment technicians	0.5 (0.3 to 0.8)
Senegal	Medical laboratory technicians	0.6 (0.3 to 0.9)
Senegal	Optometrists and opticians	0.1 (0.0 to 0.1)
Senegal	Physiotherapists and prosthetic technicians	0.2 (0.1 to 0.3)
Senegal	Psychologists	0.8 (0.5 to 1.1)
Senegal	Traditional and complementary medicine practitioners	0.3 (0.2 to 0.5)
Sierra Leone	Audiologists, speech therapists, and counsellors	1.0 (0.7 to 1.6)
Sierra Leone	Clinical officers and medical assistants	1.1 (0.7 to 1.6)
Sierra Leone	Dieticians and nutritionists	0.3 (0.2 to 0.4)
Sierra Leone	Environmental health workers	1.1 (0.7 to 1.5)
Sierra Leone	Healthcare aides, paramedics, and ambulance workers	4.1 (2.6 to 6.1)
Sierra Leone	Home-based personal care workers	1.3 (0.9 to 1.8)
Sierra Leone	Medical imaging and therapeutic equipment technicians	0.4 (0.2 to 0.5)
Sierra Leone	Medical laboratory technicians	1.3 (0.8 to 2.0)
Sierra Leone	Optometrists and opticians	0.5 (0.3 to 0.7)
Sierra Leone	Physiotherapists and prosthetic technicians	1.4 (0.9 to 1.9)
Sierra Leone	Psychologists	0.7 (0.5 to 1.0)
Sierra Leone	Traditional and complementary medicine practitioners	5.1 (3.5 to 7.5)
Togo	Audiologists, speech therapists, and counsellors	0.6 (0.4 to 0.9)
Togo	Clinical officers and medical assistants	0.8 (0.5 to 1.2)
Togo	Dieticians and nutritionists	0.2 (0.1 to 0.3)

Togo	Environmental health workers	0.5 (0.3 to 0.7)
Togo	Healthcare aides, paramedics, and ambulance workers	1.9 (1.3 to 2.7)
Togo	Home-based personal care workers	0.9 (0.6 to 1.2)
Togo	Medical imaging and therapeutic equipment technicians	0.2 (0.1 to 0.4)
Togo	Medical laboratory technicians	0.8 (0.5 to 1.2)
Togo	Optometrists and opticians	0.1 (0.1 to 0.2)
Togo	Physiotherapists and prosthetic technicians	0.3 (0.2 to 0.5)
Togo	Psychologists	0.7 (0.4 to 1.0)
Togo	Traditional and complementary medicine practitioners	1.3 (0.9 to 1.9)

Appendix 2 Table S5: Estimated female share as percent of health workers for all GBD super-regions, regions, and 204 countries and territories in 2023

Location	All health workers	Doctors	Nursing personnel	Midwifery personnel	Dentists	Dental assistants	Pharmacists	Pharmaceutical technicians and assistants
**Central Europe, eastern Europe, and central Asia**								
Central Europe, eastern Europe, and central Asia	88.9 (87.7 to 89.9)	68.4 (63.8 to 72.3)	96.4 (94.5 to 97.2)	99.0 (95.9 to 99.7)	76.7 (73.0 to 80.3)	97.9 (94.4 to 99.3)	98.4 (96.3 to 99.5)	99.1 (95.8 to 99.9)
Central Asia	86.3 (81.9 to 88.3)	58.8 (53.8 to 63.0)	95.9 (88.6 to 98.3)	99.2 (95.1 to 99.8)	74.9 (70.7 to 79.1)	96.2 (90.4 to 98.4)	98.2 (95.5 to 99.3)	99.0 (93.5 to 99.9)
Armenia	86.2 (82.1 to 89.4)	66.3 (52.7 to 75.9)	98.1 (94.0 to 99.7)	99.7 (98.3 to 100.0)	73.9 (69.4 to 78.3)	99.9 (99.7 to 100.0)	97.2 (91.5 to 99.3)	99.8 (98.2 to 100.0)
Azerbaijan	87.1 (82.5 to 90.6)	65.5 (52.1 to 77.5)	96.6 (86.5 to 99.7)	97.3 (78.3 to 100.0)	80.3 (76.5 to 83.8)	100.0 (99.8 to 100.0)	98.6 (95.8 to 99.7)	99.9 (99.4 to 100.0)
Georgia	90.2 (88.0 to 92.5)	79.4 (71.9 to 86.7)	99.5 (99.0 to 99.8)	99.9 (99.4 to 100.0)	75.2 (71.3 to 78.7)	83.8 (61.0 to 95.2)	97.6 (95.3 to 99.0)	98.1 (92.4 to 99.8)
Kazakhstan	88.8 (84.4 to 91.5)	69.6 (59.0 to 79.6)	98.1 (91.1 to 99.9)	99.7 (98.6 to 100.0)	81.0 (77.3 to 84.4)	99.9 (99.5 to 100.0)	99.9 (99.6 to 100.0)	100.0 (99.9 to 100.0)
Kyrgyzstan	80.6 (73.1 to 85.1)	37.0 (25.3 to 47.8)	97.1 (86.6 to 99.8)	99.1 (92.8 to 100.0)	76.4 (72.2 to 80.5)	99.5 (97.7 to 100.0)	98.4 (95.0 to 99.6)	99.7 (96.8 to 100.0)
Mongolia	87.9 (85.3 to 90.5)	76.0 (69.0 to 82.9)	95.8 (92.6 to 97.7)	95.2 (83.5 to 99.4)	75.2 (71.1 to 79.2)	99.8 (99.1 to 100.0)	93.1 (88.0 to 96.7)	96.3 (76.7 to 99.9)
Tajikistan	79.6 (68.7 to 87.7)	49.1 (27.8 to 73.4)	88.2 (75.3 to 96.7)	99.3 (94.6 to 100.0)	63.2 (57.8 to 68.6)	75.5 (35.9 to 97.3)	93.7 (82.1 to 98.4)	94.3 (53.3 to 100.0)

Turkmenistan	79.8 (74.3 to 84.5)	58.4 (46.5 to 69.8)	88.8 (78.6 to 95.7)	99.5 (97.0 to 100.0)	69.1 (64.1 to 74.0)	99.6 (98.1 to 100.0)	97.1 (91.2 to 99.3)	99.5 (95.0 to 100.0)
Uzbekistan	88.9 (80.1 to 92.5)	53.7 (37.5 to 67.5)	96.9 (85.7 to 99.8)	99.7 (98.0 to 100.0)	74.1 (69.6 to 78.5)	98.5 (92.9 to 99.9)	99.4 (98.1 to 99.9)	99.7 (97.6 to 100.0)
Central Europe	86.9 (85.7 to 88.1)	61.7 (55.4 to 68.6)	95.0 (93.3 to 96.0)	99.1 (96.8 to 99.9)	92.3 (90.6 to 93.8)	100.0 (99.9 to 100.0)	97.0 (92.0 to 99.2)	99.9 (99.7 to 100.0)
Albania	83.0 (78.6 to 86.5)	52.0 (38.7 to 64.0)	94.7 (90.3 to 97.5)	99.3 (95.1 to 100.0)	88.3 (85.9 to 90.6)	100.0 (100.0 to 100.0)	91.3 (76.2 to 97.7)	100.0 (99.6 to 100.0)
Bosnia and Herzegovina	78.3 (71.5 to 83.8)	62.6 (44.8 to 75.5)	81.5 (69.4 to 89.3)	99.6 (97.8 to 100.0)	85.2 (82.1 to 88.0)	100.0 (99.8 to 100.0)	91.2 (76.0 to 97.7)	99.9 (99.2 to 100.0)
Bulgaria	85.7 (82.4 to 88.7)	57.8 (44.4 to 70.6)	97.5 (89.7 to 99.8)	99.9 (99.5 to 100.0)	91.0 (89.0 to 92.8)	100.0 (100.0 to 100.0)	98.9 (96.8 to 99.8)	100.0 (100.0 to 100.0)
Croatia	82.3 (78.8 to 85.5)	64.7 (53.9 to 75.8)	89.4 (81.1 to 95.1)	82.6 (27.2 to 99.5)	88.9 (86.6 to 91.1)	99.9 (99.5 to 100.0)	98.2 (94.7 to 99.6)	100.0 (100.0 to 100.0)
Czechia	85.0 (82.1 to 87.7)	56.9 (45.1 to 68.3)	94.0 (87.9 to 97.6)	99.9 (99.6 to 100.0)	86.5 (83.7 to 89.1)	100.0 (100.0 to 100.0)	98.2 (95.7 to 99.5)	99.8 (98.5 to 100.0)
Hungary	90.6 (88.2 to 92.6)	61.1 (51.0 to 72.1)	93.1 (86.0 to 97.0)	99.8 (98.7 to 100.0)	91.2 (89.3 to 93.0)	100.0 (100.0 to 100.0)	97.7 (93.1 to 99.5)	100.0 (100.0 to 100.0)
Montenegro	78.9 (74.3 to 82.7)	64.0 (52.0 to 73.2)	82.9 (75.3 to 88.8)	99.9 (99.6 to 100.0)	86.7 (83.9 to 89.3)	100.0 (99.9 to 100.0)	96.1 (89.3 to 99.0)	100.0 (99.9 to 100.0)
North Macedonia	86.2 (82.2 to 89.4)	62.7 (51.9 to 74.7)	96.5 (89.4 to 99.4)	99.3 (95.2 to 100.0)	86.8 (84.1 to 89.4)	100.0 (99.9 to 100.0)	91.7 (80.9 to 97.4)	100.0 (99.8 to 100.0)
Poland	87.9 (85.3 to 90.5)	59.5 (48.6 to 71.7)	99.2 (96.9 to 99.9)	99.9 (99.6 to 100.0)	100.0 (100.0 to 100.0)	100.0 (100.0 to 100.0)	98.1 (95.1 to 99.5)	100.0 (100.0 to 100.0)
Romania	88.3 (86.1 to 90.6)	68.9 (59.0 to 79.2)	92.6 (87.6 to 96.1)	99.3 (95.2 to 100.0)	88.2 (85.7 to 90.5)	100.0 (100.0 to 100.0)	96.9 (91.4 to 99.2)	100.0 (99.9 to 100.0)
Serbia	85.1 (81.3 to 88.4)	66.1 (51.9 to 76.1)	90.2 (82.5 to 95.7)	99.9 (99.5 to 100.0)	84.8 (81.7 to 87.6)	100.0 (99.8 to 100.0)	94.8 (85.9 to 98.6)	99.8 (97.9 to 100.0)
Slovakia	88.2 (86.0 to 90.4)	59.3 (48.8 to 70.8)	97.6 (94.6 to 99.3)	99.9 (99.6 to 100.0)	90.5 (88.4 to 92.4)	100.0 (100.0 to 100.0)	93.4 (83.8 to 98.1)	100.0 (100.0 to 100.0)
Slovenia	87.0 (83.5 to 89.8)	68.3 (55.6 to 77.3)	88.9 (79.8 to 94.4)	97.4 (79.0 to 100.0)	89.2 (86.9 to 91.4)	99.8 (99.4 to 100.0)	97.6 (93.8 to 99.3)	99.5 (96.4 to 100.0)
Eastern Europe	91.1 (89.7 to 92.2)	76.7 (67.0 to 84.5)	97.4 (95.7 to 98.5)	98.9 (93.7 to 100.0)	68.9 (64.3 to 73.4)	97.6 (91.2 to 99.7)	99.3 (98.8 to 99.7)	98.7 (91.7 to 100.0)
Belarus	91.3 (89.3 to 93.2)	73.6 (63.1 to 83.2)	99.2 (97.0 to 99.9)	100.0 (99.9 to 100.0)	73.6 (69.0 to 78.0)	100.0 (99.8 to 100.0)	100.0 (99.9 to 100.0)	100.0 (100.0 to 100.0)
Estonia	90.7 (88.1 to 92.6)	73.5 (61.3 to 81.9)	97.8 (94.6 to 99.4)	100.0 (100.0 to 100.0)	68.8 (63.8 to 73.7)	100.0 (100.0 to 100.0)	100.0 (99.9 to 100.0)	100.0 (100.0 to 100.0)
Latvia	89.1 (86.7 to 91.4)	74.6 (62.8 to 82.8)	99.3 (97.8 to 99.9)	100.0 (100.0 to 100.0)	67.9 (62.8 to 72.9)	100.0 (100.0 to 100.0)	99.9 (99.8 to 100.0)	100.0 (100.0 to 100.0)

Lithuania	91.5 (89.6 to 93.4)	72.9 (63.1 to 82.4)	99.8 (99.3 to 100.0)	100.0 (100.0 to 100.0)	68.8 (63.8 to 73.7)	100.0 (100.0 to 100.0)	100.0 (100.0 to 100.0)	100.0 (100.0 to 100.0)
Moldova	86.4 (82.9 to 89.0)	59.4 (48.0 to 70.2)	98.3 (93.7 to 99.7)	100.0 (99.9 to 100.0)	76.9 (72.7 to 81.0)	100.0 (100.0 to 100.0)	99.7 (99.0 to 99.9)	100.0 (99.9 to 100.0)
Russia	91.6 (90.3 to 92.9)	79.6 (71.9 to 86.2)	96.8 (94.5 to 98.5)	98.4 (91.3 to 100.0)	69.4 (65.0 to 73.8)	96.7 (87.9 to 99.6)	99.1 (98.3 to 99.6)	98.2 (89.8 to 100.0)
Ukraine	88.4 (81.6 to 92.4)	68.1 (47.0 to 84.9)	98.8 (94.7 to 99.9)	100.0 (99.9 to 100.0)	65.1 (59.9 to 70.4)	99.7 (98.7 to 100.0)	100.0 (99.9 to 100.0)	99.6 (97.2 to 100.0)
High income	78.9 (78.0 to 79.7)	44.3 (42.3 to 46.9)	87.9 (86.9 to 88.6)	99.7 (98.8 to 99.9)	40.2 (36.9 to 43.7)	98.6 (98.3 to 98.8)	74.3 (67.2 to 79.1)	92.7 (89.6 to 94.0)
Australasia	84.5 (82.0 to 87.0)	43.2 (32.6 to 53.3)	89.0 (82.0 to 94.2)	99.9 (99.6 to 100.0)	44.8 (39.3 to 50.7)	100.0 (100.0 to 100.0)	73.1 (55.0 to 87.6)	100.0 (100.0 to 100.0)
Australia	84.7 (82.0 to 87.3)	42.4 (30.5 to 54.6)	88.2 (80.1 to 94.1)	99.9 (99.6 to 100.0)	43.2 (37.7 to 49.1)	100.0 (100.0 to 100.0)	70.9 (52.9 to 86.2)	100.0 (100.0 to 100.0)
New Zealand	83.8 (80.7 to 86.6)	47.2 (34.6 to 58.0)	92.9 (86.8 to 96.8)	100.0 (100.0 to 100.0)	53.3 (47.7 to 59.2)	100.0 (100.0 to 100.0)	84.7 (62.5 to 95.7)	100.0 (100.0 to 100.0)
High-income Asia Pacific	85.8 (83.6 to 88.1)	27.1 (22.0 to 34.8)	96.0 (90.2 to 98.9)	100.0 (100.0 to 100.0)	29.1 (25.1 to 33.4)	100.0 (100.0 to 100.0)	95.5 (89.3 to 98.7)	98.9 (91.2 to 100.0)
Brunei	74.4 (67.1 to 80.1)	43.1 (15.0 to 72.5)	82.7 (63.8 to 94.2)	100.0 (99.8 to 100.0)	42.5 (37.1 to 48.4)	100.0 (99.9 to 100.0)	90.2 (76.3 to 97.1)	99.9 (98.8 to 100.0)
Japan	86.7 (84.6 to 89.2)	26.7 (15.7 to 40.6)	95.4 (89.5 to 98.6)	100.0 (100.0 to 100.0)	39.2 (33.9 to 45.0)	100.0 (100.0 to 100.0)	99.3 (98.1 to 99.8)	98.3 (87.1 to 100.0)
South Korea	86.3 (83.0 to 89.0)	26.5 (15.7 to 39.1)	98.1 (93.5 to 99.8)	100.0 (100.0 to 100.0)	2.9 (2.3 to 3.6)	100.0 (100.0 to 100.0)	86.3 (68.2 to 96.1)	100.0 (100.0 to 100.0)
Singapore	80.3 (75.8 to 85.2)	42.0 (14.4 to 71.4)	90.6 (75.4 to 97.9)	100.0 (99.9 to 100.0)	43.7 (38.2 to 49.6)	100.0 (100.0 to 100.0)	94.8 (85.1 to 98.7)	100.0 (99.8 to 100.0)
High-income North America	78.2 (77.6 to 79.0)	44.2 (43.6 to 44.8)	89.0 (88.1 to 89.7)	99.9 (99.5 to 100.0)	36.4 (35.5 to 37.5)	96.2 (95.6 to 96.8)	60.1 (58.1 to 62.0)	82.9 (81.0 to 84.5)
Canada	83.7 (80.9 to 86.6)	45.4 (34.5 to 56.8)	91.1 (84.8 to 95.4)	99.4 (95.8 to 100.0)	47.6 (42.0 to 53.6)	99.9 (99.5 to 100.0)	62.8 (43.2 to 80.9)	99.3 (93.1 to 100.0)
Greenland	79.8 (70.4 to 85.8)	37.3 (11.9 to 66.9)	92.7 (68.9 to 99.4)	99.9 (99.7 to 100.0)	37.4 (32.2 to 43.1)	99.8 (99.0 to 100.0)	27.9 (8.2 to 54.7)	92.7 (44.5 to 100.0)
USA	77.6 (76.9 to 78.4)	44.1 (42.7 to 45.3)	88.8 (88.3 to 89.2)	100.0 (99.9 to 100.0)	35.2 (33.8 to 36.7)	95.8 (95.1 to 96.4)	59.8 (57.9 to 61.8)	81.0 (79.1 to 82.7)
Southern Latin America	72.2 (68.8 to 75.8)	49.1 (40.6 to 57.5)	83.9 (79.0 to 88.5)	97.8 (90.4 to 99.4)	56.0 (51.1 to 60.8)	97.7 (95.4 to 98.8)	65.1 (53.2 to 75.0)	86.5 (58.4 to 96.0)
Argentina	69.3 (64.5 to 73.7)	50.3 (38.3 to 62.4)	82.6 (75.5 to 88.9)	97.7 (85.9 to 100.0)	57.6 (52.0 to 63.3)	99.5 (97.4 to 100.0)	64.9 (44.8 to 83.2)	93.2 (47.4 to 100.0)
Chile	78.9 (75.4 to 82.1)	44.1 (33.6 to 53.1)	87.3 (80.6 to 92.1)	99.6 (97.1 to 100.0)	50.5 (45.8 to 55.3)	93.8 (84.9 to 98.3)	62.7 (42.8 to 80.0)	72.6 (31.3 to 95.9)

Uruguay	77.4 (74.8 to 79.9)	59.8 (51.7 to 68.5)	83.3 (77.4 to 88.4)	88.7 (55.1 to 99.2)	64.3 (60.2 to 68.1)	95.7 (87.4 to 99.0)	80.7 (61.8 to 93.1)	71.3 (30.7 to 94.6)
Western Europe	77.2 (76.1 to 78.3)	50.7 (47.0 to 55.3)	84.3 (82.8 to 85.0)	99.8 (98.5 to 100.0)	45.1 (39.7 to 50.8)	100.0 (100.0 to 100.0)	79.0 (67.0 to 86.2)	98.9 (96.0 to 99.8)
Andorra	74.3 (68.7 to 79.5)	46.9 (35.1 to 59.2)	87.2 (80.5 to 92.8)	100.0 (99.9 to 100.0)	48.5 (42.9 to 54.5)	100.0 (100.0 to 100.0)	50.9 (20.9 to 78.1)	100.0 (99.7 to 100.0)
Austria	81.8 (79.0 to 84.7)	50.5 (41.2 to 61.2)	87.0 (79.2 to 92.9)	100.0 (99.9 to 100.0)	43.7 (38.2 to 49.6)	100.0 (100.0 to 100.0)	76.0 (47.5 to 92.4)	100.0 (100.0 to 100.0)
Belgium	78.5 (73.7 to 82.3)	48.9 (38.5 to 60.8)	84.9 (74.8 to 92.4)	100.0 (99.9 to 100.0)	39.3 (34.0 to 45.1)	100.0 (100.0 to 100.0)	91.9 (79.3 to 97.7)	98.5 (90.7 to 100.0)
Cyprus	68.8 (63.8 to 73.7)	39.5 (27.8 to 50.0)	83.3 (67.9 to 93.5)	99.7 (98.4 to 100.0)	62.3 (56.9 to 67.8)	100.0 (100.0 to 100.0)	78.8 (53.3 to 93.4)	100.0 (99.9 to 100.0)
Denmark	81.1 (77.6 to 84.0)	53.2 (40.9 to 65.1)	93.5 (86.6 to 97.4)	100.0 (99.8 to 100.0)	40.8 (35.5 to 46.5)	100.0 (100.0 to 100.0)	91.4 (79.0 to 97.5)	97.2 (85.4 to 99.9)
Finland	84.7 (81.8 to 87.4)	57.7 (45.8 to 70.6)	92.1 (85.6 to 96.7)	100.0 (99.8 to 100.0)	48.0 (42.3 to 53.9)	100.0 (100.0 to 100.0)	88.6 (70.1 to 96.9)	100.0 (100.0 to 100.0)
France	73.5 (69.1 to 77.8)	51.9 (46.4 to 57.7)	80.3 (74.4 to 85.2)	99.9 (99.8 to 100.0)	43.9 (38.4 to 49.8)	100.0 (100.0 to 100.0)	72.0 (64.1 to 78.0)	100.0 (99.8 to 100.0)
Germany	84.0 (81.4 to 86.0)	50.6 (40.1 to 60.9)	86.0 (78.2 to 91.1)	99.9 (99.4 to 100.0)	48.4 (42.8 to 54.3)	100.0 (100.0 to 100.0)	75.0 (46.0 to 92.0)	97.2 (87.7 to 99.8)
Greece	64.3 (58.4 to 69.8)	46.0 (36.0 to 57.7)	78.2 (60.7 to 90.8)	94.4 (57.0 to 100.0)	44.6 (39.1 to 50.5)	100.0 (100.0 to 100.0)	69.3 (42.7 to 86.9)	100.0 (99.6 to 100.0)
Iceland	84.2 (81.6 to 86.7)	45.3 (34.2 to 54.9)	95.0 (88.5 to 98.4)	100.0 (99.9 to 100.0)	48.6 (43.0 to 54.6)	100.0 (100.0 to 100.0)	86.1 (74.2 to 94.5)	98.8 (93.7 to 100.0)
Ireland	86.4 (83.6 to 88.8)	51.3 (41.9 to 62.1)	90.2 (83.5 to 94.7)	100.0 (99.9 to 100.0)	36.5 (31.4 to 42.2)	100.0 (100.0 to 100.0)	84.4 (61.1 to 95.5)	100.0 (100.0 to 100.0)
Israel	77.3 (74.1 to 80.8)	44.0 (33.1 to 54.9)	84.4 (77.2 to 90.0)	99.8 (98.7 to 100.0)	52.8 (47.3 to 58.7)	100.0 (100.0 to 100.0)	74.5 (59.8 to 87.6)	100.0 (100.0 to 100.0)
Italy	69.0 (64.8 to 73.3)	44.8 (35.9 to 56.1)	79.0 (68.2 to 87.4)	99.9 (99.7 to 100.0)	42.4 (37.0 to 48.3)	100.0 (100.0 to 100.0)	65.4 (36.5 to 86.4)	99.9 (99.2 to 100.0)
Luxembourg	72.4 (67.7 to 76.8)	50.6 (33.5 to 64.8)	80.3 (73.3 to 86.7)	99.9 (99.8 to 100.0)	35.4 (30.3 to 40.9)	100.0 (100.0 to 100.0)	99.2 (97.5 to 99.8)	100.0 (100.0 to 100.0)
Malta	66.6 (61.3 to 72.6)	45.9 (32.8 to 58.7)	73.8 (61.3 to 83.6)	99.9 (99.8 to 100.0)	54.6 (49.0 to 60.4)	100.0 (100.0 to 100.0)	85.7 (64.1 to 96.0)	100.0 (99.9 to 100.0)
Monaco	82.8 (73.0 to 88.6)	45.6 (21.4 to 66.2)	92.5 (68.0 to 99.4)	100.0 (99.9 to 100.0)	60.8 (55.3 to 66.4)	100.0 (100.0 to 100.0)	98.2 (94.6 to 99.6)	100.0 (100.0 to 100.0)
Netherlands	83.1 (80.6 to 85.6)	58.1 (47.7 to 69.4)	87.3 (79.1 to 93.9)	100.0 (99.9 to 100.0)	55.6 (50.0 to 61.4)	100.0 (100.0 to 100.0)	94.4 (84.1 to 98.6)	99.6 (96.6 to 100.0)
Norway	79.1 (74.8 to 82.6)	51.6 (41.8 to 62.6)	90.8 (84.2 to 95.1)	99.9 (99.6 to 100.0)	32.7 (27.9 to 38.1)	100.0 (100.0 to 100.0)	91.2 (81.2 to 97.0)	99.5 (96.3 to 100.0)

Portugal	73.0 (68.2 to 77.2)	57.7 (46.3 to 68.2)	78.8 (67.9 to 87.8)	99.9 (99.4 to 100.0)	57.9 (52.3 to 63.6)	100.0 (99.9 to 100.0)	92.2 (78.5 to 98.0)	100.0 (99.9 to 100.0)
San Marino	72.2 (66.9 to 77.1)	50.8 (35.8 to 65.7)	81.6 (70.1 to 90.2)	100.0 (99.9 to 100.0)	49.9 (44.3 to 55.8)	100.0 (100.0 to 100.0)	37.1 (12.4 to 65.7)	100.0 (99.8 to 100.0)
Spain	77.1 (73.2 to 80.6)	55.9 (44.2 to 67.1)	84.9 (76.9 to 91.7)	99.9 (99.7 to 100.0)	49.6 (44.0 to 55.4)	100.0 (100.0 to 100.0)	86.9 (74.6 to 94.6)	95.3 (74.5 to 99.9)
Sweden	75.0 (71.1 to 79.0)	62.0 (52.3 to 73.0)	85.7 (74.7 to 93.4)	99.9 (99.7 to 100.0)	42.6 (37.1 to 48.5)	99.7 (98.7 to 100.0)	98.4 (95.2 to 99.6)	100.0 (100.0 to 100.0)
Switzerland	72.6 (68.5 to 76.6)	45.7 (35.7 to 57.7)	83.9 (75.2 to 90.2)	99.9 (99.8 to 100.0)	49.5 (43.9 to 55.4)	100.0 (100.0 to 100.0)	77.7 (56.7 to 91.8)	100.0 (100.0 to 100.0)
UK	79.1 (75.5 to 82.2)	48.2 (37.8 to 58.6)	88.6 (82.0 to 93.6)	99.9 (99.2 to 100.0)	38.3 (33.2 to 44.1)	100.0 (100.0 to 100.0)	85.4 (68.0 to 94.6)	100.0 (99.8 to 100.0)
**Latin America and Caribbean**								
Latin America and Caribbean	71.7 (69.6 to 73.5)	49.3 (45.8 to 52.3)	84.6 (80.8 to 87.0)	96.5 (87.9 to 98.4)	58.6 (56.2 to 61.0)	93.5 (87.1 to 96.0)	72.9 (66.0 to 79.5)	81.7 (70.2 to 91.2)
Andean Latin America	73.0 (70.7 to 75.0)	49.9 (43.7 to 56.3)	87.9 (82.7 to 92.2)	91.2 (81.6 to 96.3)	60.7 (55.9 to 65.4)	95.0 (91.6 to 96.3)	70.7 (56.2 to 83.8)	97.1 (91.4 to 98.6)
Bolivia	75.6 (70.5 to 80.5)	48.8 (37.0 to 62.3)	86.9 (74.7 to 94.2)	100.0 (99.9 to 100.0)	62.4 (57.7 to 66.9)	80.7 (57.2 to 93.9)	92.1 (84.7 to 96.6)	95.9 (82.3 to 99.6)
Ecuador	67.2 (61.3 to 72.7)	55.5 (42.9 to 65.1)	85.3 (74.6 to 92.7)	81.0 (37.9 to 98.2)	55.7 (51.2 to 60.2)	95.2 (87.1 to 98.9)	54.5 (30.6 to 76.3)	92.2 (68.4 to 99.3)
Peru	75.7 (71.4 to 79.8)	47.5 (36.1 to 59.2)	89.6 (82.7 to 94.7)	93.2 (77.8 to 98.9)	62.5 (57.1 to 68.0)	99.6 (98.1 to 100.0)	71.5 (53.3 to 86.8)	100.0 (99.6 to 100.0)
Caribbean	74.9 (66.9 to 81.6)	60.2 (43.2 to 75.9)	87.5 (69.0 to 96.3)	99.1 (94.8 to 99.9)	56.5 (51.0 to 62.2)	94.3 (77.4 to 99.7)	78.4 (59.9 to 91.4)	99.1 (94.0 to 99.9)
Antigua and Barbuda	84.7 (78.0 to 90.2)	49.6 (19.0 to 77.8)	96.4 (88.9 to 99.5)	98.5 (88.8 to 100.0)	62.8 (57.4 to 68.2)	100.0 (99.8 to 100.0)	73.1 (43.3 to 91.2)	100.0 (99.7 to 100.0)
The Bahamas	84.8 (79.7 to 89.1)	65.3 (32.3 to 87.7)	94.4 (81.5 to 99.0)	99.9 (99.5 to 100.0)	57.6 (52.0 to 63.3)	99.8 (99.1 to 100.0)	98.9 (96.5 to 99.7)	100.0 (100.0 to 100.0)
Barbados	81.0 (75.1 to 86.6)	55.3 (23.2 to 81.8)	90.5 (76.9 to 98.0)	99.0 (92.0 to 100.0)	63.7 (58.3 to 69.0)	99.9 (99.6 to 100.0)	91.9 (77.8 to 97.9)	100.0 (99.9 to 100.0)
Belize	77.9 (69.5 to 85.5)	50.2 (19.4 to 78.2)	93.1 (87.9 to 97.0)	99.5 (96.9 to 100.0)	56.0 (50.4 to 61.8)	98.4 (92.4 to 99.9)	59.5 (28.0 to 84.0)	99.5 (95.2 to 100.0)
Bermuda	85.3 (80.6 to 89.6)	60.3 (27.4 to 84.9)	95.4 (79.4 to 99.7)	99.9 (99.7 to 100.0)	34.6 (29.6 to 40.2)	100.0 (99.9 to 100.0)	98.3 (94.9 to 99.6)	100.0 (100.0 to 100.0)
Cuba	77.4 (70.0 to 84.0)	66.7 (47.0 to 83.0)	89.1 (78.0 to 95.6)	99.6 (97.8 to 100.0)	55.3 (49.7 to 61.2)	95.8 (80.4 to 99.8)	98.0 (94.1 to 99.5)	100.0 (99.7 to 100.0)
Dominica	85.1 (71.8 to 92.0)	48.2 (18.1 to 76.7)	95.3 (78.7 to 99.6)	99.8 (99.1 to 100.0)	55.5 (49.8 to 61.3)	99.9 (99.6 to 100.0)	47.7 (18.7 to 75.6)	99.8 (98.3 to 100.0)
Dominican Republic	78.7 (73.4 to 83.1)	69.7 (58.1 to 79.2)	92.4 (85.9 to 96.7)	99.6 (97.3 to 100.0)	57.6 (52.0 to 63.3)	98.7 (93.8 to 100.0)	85.8 (67.2 to 95.6)	99.8 (97.8 to 100.0)

Grenada	82.5 (75.0 to 89.0)	51.2 (20.1 to 79.0)	94.0 (82.7 to 98.8)	99.7 (98.1 to 100.0)	56.7 (51.1 to 62.4)	99.4 (97.1 to 100.0)	67.8 (36.6 to 88.6)	99.8 (98.0 to 100.0)
Guyana	84.0 (78.8 to 88.1)	53.6 (36.9 to 71.0)	93.5 (87.1 to 97.2)	93.4 (70.8 to 99.6)	47.6 (42.0 to 53.6)	94.5 (84.0 to 99.0)	66.7 (35.3 to 88.1)	85.1 (51.4 to 98.4)
Haiti	73.0 (58.3 to 84.5)	47.5 (17.7 to 76.2)	76.5 (30.4 to 97.1)	98.5 (87.0 to 100.0)	60.1 (54.6 to 65.7)	85.9 (48.1 to 99.3)	56.9 (25.7 to 82.3)	98.0 (81.8 to 100.0)
Jamaica	86.9 (82.3 to 90.3)	62.3 (36.2 to 83.8)	95.3 (82.4 to 99.4)	99.9 (99.7 to 100.0)	62.7 (57.4 to 68.2)	99.0 (96.1 to 99.9)	79.3 (61.1 to 92.0)	100.0 (100.0 to 100.0)
Puerto Rico	82.6 (69.8 to 92.0)	66.7 (33.8 to 88.4)	95.4 (79.0 to 99.6)	99.8 (99.2 to 100.0)	39.8 (34.5 to 45.6)	98.2 (91.2 to 99.9)	94.1 (83.3 to 98.5)	99.9 (99.4 to 100.0)
Saint Kitts and Nevis	79.1 (72.1 to 86.1)	49.2 (18.7 to 77.5)	91.0 (79.6 to 97.4)	99.9 (99.4 to 100.0)	62.5 (57.1 to 67.9)	100.0 (99.8 to 100.0)	71.3 (40.9 to 90.3)	100.0 (99.7 to 100.0)
Saint Lucia	74.1 (61.0 to 85.7)	51.1 (20.1 to 78.9)	92.7 (78.9 to 98.5)	99.9 (99.7 to 100.0)	59.7 (54.1 to 65.3)	99.6 (98.2 to 100.0)	72.7 (42.7 to 91.0)	99.9 (98.8 to 100.0)
Saint Vincent and the Grenadines	84.9 (75.7 to 92.0)	53.0 (21.5 to 80.3)	95.0 (87.7 to 99.0)	99.6 (97.5 to 100.0)	53.8 (48.1 to 59.6)	98.3 (92.0 to 99.9)	69.3 (38.4 to 89.4)	99.6 (96.7 to 100.0)
Suriname	79.5 (73.0 to 84.6)	53.2 (31.8 to 73.0)	85.0 (67.5 to 94.3)	92.3 (69.2 to 99.1)	56.9 (51.3 to 62.6)	96.5 (83.5 to 99.9)	72.0 (54.0 to 87.3)	100.0 (99.9 to 100.0)
Trinidad and Tobago	74.5 (67.3 to 80.1)	48.4 (32.7 to 64.6)	95.7 (84.9 to 99.3)	100.0 (99.8 to 100.0)	54.3 (48.7 to 60.1)	100.0 (99.9 to 100.0)	38.6 (13.2 to 67.3)	99.9 (98.9 to 100.0)
Virgin Islands	82.7 (71.2 to 91.3)	59.2 (26.4 to 84.3)	95.9 (81.2 to 99.7)	99.9 (99.7 to 100.0)	62.2 (56.8 to 67.7)	100.0 (99.8 to 100.0)	96.3 (89.0 to 99.1)	100.0 (100.0 to 100.0)
Central Latin America	67.1 (64.2 to 69.8)	48.1 (42.2 to 53.4)	80.9 (76.9 to 84.0)	95.4 (79.9 to 98.5)	58.7 (57.5 to 59.9)	89.8 (85.4 to 93.2)	67.7 (58.9 to 75.8)	98.2 (92.1 to 99.7)
Colombia	70.3 (65.6 to 74.7)	49.6 (36.2 to 64.4)	88.4 (77.0 to 94.6)	99.6 (97.3 to 100.0)	59.4 (53.9 to 65.0)	99.0 (94.9 to 100.0)	54.8 (35.7 to 75.1)	99.8 (98.5 to 100.0)
Costa Rica	71.3 (65.7 to 76.0)	50.3 (37.1 to 60.7)	83.6 (74.7 to 90.4)	99.7 (98.3 to 100.0)	57.3 (51.9 to 62.9)	98.8 (94.0 to 99.9)	67.5 (49.0 to 85.5)	94.1 (63.5 to 99.9)
El Salvador	66.8 (62.2 to 71.0)	45.4 (34.6 to 57.2)	81.1 (72.2 to 87.7)	66.2 (7.6 to 99.0)	67.7 (63.1 to 72.2)	72.8 (53.3 to 86.8)	70.9 (51.8 to 86.6)	95.2 (70.7 to 99.9)
Guatemala	65.4 (58.6 to 71.7)	43.8 (26.2 to 60.3)	80.3 (72.5 to 87.8)	98.9 (90.6 to 100.0)	56.0 (50.4 to 61.8)	97.7 (88.9 to 99.9)	71.8 (50.1 to 88.4)	99.6 (96.6 to 100.0)
Honduras	68.4 (58.0 to 76.8)	48.7 (27.2 to 73.1)	85.9 (65.0 to 96.0)	82.1 (46.9 to 97.6)	62.5 (57.3 to 67.8)	99.8 (99.2 to 100.0)	65.7 (34.7 to 87.7)	99.8 (98.3 to 100.0)
Mexico	64.7 (60.2 to 68.7)	45.3 (36.5 to 52.4)	76.4 (68.7 to 82.4)	94.5 (67.8 to 99.9)	57.0 (52.9 to 61.0)	89.0 (79.6 to 94.7)	65.2 (47.9 to 78.6)	97.0 (85.2 to 99.9)
Nicaragua	73.2 (61.5 to 80.5)	48.2 (25.2 to 72.9)	88.7 (59.0 to 98.5)	99.0 (92.4 to 100.0)	68.7 (63.6 to 73.6)	96.6 (83.8 to 99.9)	79.6 (56.6 to 93.2)	99.8 (98.0 to 100.0)
Panama	76.5 (71.8 to 80.4)	45.3 (32.7 to 59.8)	90.7 (83.5 to 95.5)	99.7 (98.3 to 100.0)	59.4 (53.9 to 65.1)	99.6 (97.9 to 100.0)	81.9 (57.0 to 94.7)	100.0 (99.5 to 100.0)

Venezuela	69.7 (60.8 to 77.7)	62.8 (29.8 to 86.3)	83.0 (61.8 to 95.3)	99.0 (91.9 to 100.0)	62.0 (56.6 to 67.5)	65.2 (17.5 to 96.9)	99.0 (96.9 to 99.8)	100.0 (99.6 to 100.0)
Tropical Latin America	75.7 (73.0 to 78.7)	48.1 (39.5 to 56.8)	87.5 (82.4 to 91.7)	99.0 (92.6 to 100.0)	58.3 (54.4 to 62.0)	97.3 (88.4 to 99.6)	78.6 (70.5 to 84.9)	53.7 (25.6 to 80.1)
Brazil	75.8 (72.9 to 78.9)	47.8 (39.0 to 56.9)	87.7 (82.4 to 92.0)	99.0 (92.4 to 100.0)	58.1 (54.3 to 61.9)	97.5 (88.1 to 99.9)	78.6 (69.9 to 85.3)	52.4 (23.4 to 79.6)
Paraguay	74.4 (67.2 to 79.1)	55.9 (41.2 to 69.0)	82.7 (73.3 to 89.5)	98.0 (89.6 to 99.9)	63.4 (58.2 to 68.5)	90.3 (64.8 to 99.2)	80.0 (59.9 to 92.6)	97.5 (81.3 to 100.0)
**North Africa and Middle East**								
North Africa and Middle East	60.1 (56.6 to 64.0)	45.4 (31.5 to 59.5)	73.8 (68.4 to 76.9)	99.5 (96.0 to 100.0)	42.3 (37.2 to 47.7)	68.5 (55.6 to 78.9)	54.2 (43.7 to 65.9)	66.5 (27.3 to 88.1)
**North Africa and Middle East**								
North Africa and Middle East	60.1 (56.6 to 64.0)	45.4 (31.5 to 59.5)	73.8 (68.4 to 76.9)	99.5 (96.0 to 100.0)	42.3 (37.2 to 47.7)	68.5 (55.6 to 78.9)	54.2 (43.7 to 65.9)	66.5 (27.3 to 88.1)
Afghanistan	26.4 (21.1 to 32.8)	26.1 (12.3 to 43.3)	30.1 (20.2 to 42.0)	97.1 (73.0 to 100.0)	46.1 (40.5 to 52.0)	15.3 (1.0 to 60.5)	12.4 (3.8 to 27.3)	16.0 (0.1 to 78.9)
Algeria	61.1 (51.6 to 69.7)	56.6 (33.7 to 76.2)	66.8 (47.0 to 83.1)	99.2 (94.4 to 100.0)	40.7 (35.3 to 46.5)	67.4 (19.4 to 97.3)	66.4 (45.4 to 84.2)	74.2 (7.9 to 99.7)
Bahrain	75.5 (69.5 to 80.7)	50.7 (33.5 to 68.1)	86.8 (75.1 to 95.2)	99.9 (99.8 to 100.0)	55.4 (49.8 to 61.2)	99.9 (99.4 to 100.0)	76.5 (54.1 to 91.5)	96.6 (69.7 to 100.0)
Egypt	76.6 (71.3 to 81.6)	54.5 (41.0 to 70.1)	93.2 (85.8 to 97.3)	99.7 (98.6 to 100.0)	39.7 (34.9 to 44.9)	41.5 (11.0 to 76.4)	80.7 (67.6 to 90.1)	74.0 (7.8 to 99.7)
Iran	66.6 (60.6 to 72.3)	44.9 (29.6 to 61.7)	79.1 (69.4 to 87.1)	99.9 (99.8 to 100.0)	35.0 (30.0 to 40.6)	80.4 (36.4 to 98.8)	59.5 (40.0 to 78.5)	77.6 (10.4 to 99.8)
Iraq	60.5 (53.0 to 67.1)	50.0 (34.8 to 64.1)	63.7 (50.8 to 75.0)	99.6 (96.7 to 100.0)	42.7 (37.2 to 48.6)	96.6 (84.2 to 99.9)	69.0 (50.3 to 83.2)	66.0 (10.1 to 98.2)
Jordan	65.0 (58.5 to 70.3)	42.6 (29.0 to 58.2)	70.8 (62.3 to 80.0)	98.9 (92.4 to 100.0)	32.9 (28.1 to 38.2)	35.2 (4.0 to 86.1)	91.4 (81.1 to 97.0)	72.8 (21.3 to 97.4)
Kuwait	79.4 (71.1 to 84.8)	46.6 (17.1 to 75.4)	90.7 (62.1 to 99.2)	100.0 (99.9 to 100.0)	56.8 (51.1 to 62.5)	100.0 (99.9 to 100.0)	83.6 (60.1 to 95.3)	99.5 (95.1 to 100.0)
Lebanon	63.4 (54.2 to 73.0)	43.5 (19.8 to 71.8)	77.7 (55.5 to 90.7)	99.9 (99.7 to 100.0)	48.9 (43.3 to 54.8)	93.6 (71.7 to 99.7)	82.6 (58.3 to 95.0)	92.1 (41.5 to 100.0)
Libya	73.1 (51.7 to 84.5)	47.0 (17.3 to 75.7)	85.8 (48.4 to 98.6)	99.9 (99.7 to 100.0)	47.4 (41.8 to 53.3)	98.8 (94.0 to 100.0)	58.4 (27.0 to 83.3)	90.5 (34.9 to 100.0)
Morocco	52.3 (42.7 to 60.7)	43.6 (21.6 to 69.5)	69.1 (56.2 to 79.5)	99.9 (99.7 to 100.0)	42.8 (37.3 to 48.7)	95.3 (78.4 to 99.8)	5.0 (1.1 to 12.8)	42.6 (0.9 to 97.2)
Oman	71.5 (66.2 to 76.0)	44.2 (30.4 to 58.9)	86.3 (75.4 to 92.7)	99.6 (97.7 to 100.0)	52.0 (46.3 to 57.9)	100.0 (99.8 to 100.0)	39.7 (22.5 to 61.4)	87.4 (25.2 to 99.9)
Palestine	64.7 (33.9 to 83.9)	61.3 (31.5 to 85.0)	66.1 (18.9 to 94.5)	99.6 (97.2 to 100.0)	36.7 (31.5 to 42.3)	9.8 (0.6 to 45.4)	98.4 (95.2 to 99.6)	86.6 (23.4 to 99.9)

Qatar	68.5 (62.2 to 74.4)	37.9 (23.4 to 54.3)	75.8 (63.6 to 84.2)	100.0 (100.0 to 100.0)	62.9 (57.6 to 68.4)	100.0 (100.0 to 100.0)	67.3 (48.8 to 83.8)	99.8 (98.4 to 100.0)
Saudi Arabia	61.8 (56.6 to 66.8)	36.0 (24.3 to 48.5)	77.0 (68.5 to 83.6)	100.0 (99.9 to 100.0)	42.6 (37.2 to 48.5)	99.8 (98.9 to 100.0)	31.9 (15.0 to 55.0)	92.8 (44.9 to 100.0)
Sudan	62.0 (40.8 to 78.6)	41.3 (14.0 to 70.8)	71.7 (24.4 to 96.0)	99.8 (99.2 to 100.0)	40.8 (35.4 to 46.6)	69.5 (21.4 to 97.6)	19.7 (5.2 to 42.3)	43.9 (0.9 to 97.5)
Syria	60.0 (44.7 to 72.4)	52.9 (21.4 to 80.2)	68.1 (20.7 to 95.1)	99.5 (96.8 to 100.0)	39.8 (34.5 to 45.6)	34.5 (3.9 to 85.7)	71.2 (40.8 to 90.3)	63.6 (3.6 to 99.3)
Tunisia	65.6 (45.6 to 78.1)	45.6 (23.2 to 70.6)	81.4 (38.9 to 97.9)	99.9 (99.7 to 100.0)	46.2 (40.6 to 52.1)	97.0 (85.9 to 99.9)	26.6 (7.7 to 52.9)	73.2 (7.3 to 99.7)
Türkiye	62.9 (54.4 to 69.9)	42.1 (30.3 to 54.3)	83.1 (73.4 to 90.0)	100.0 (99.9 to 100.0)	51.3 (45.6 to 57.2)	99.5 (97.6 to 100.0)	65.8 (46.5 to 83.2)	76.9 (20.8 to 99.0)
United Arab Emirates	70.3 (64.9 to 76.2)	43.1 (31.6 to 56.3)	79.8 (69.3 to 86.9)	99.8 (98.6 to 100.0)	58.5 (53.0 to 64.2)	100.0 (100.0 to 100.0)	56.5 (37.2 to 76.2)	99.1 (92.0 to 100.0)
Yemen	36.9 (30.8 to 43.3)	36.8 (19.9 to 55.2)	41.8 (31.6 to 52.7)	97.3 (75.4 to 100.0)	36.3 (31.2 to 42.0)	7.2 (0.4 to 36.0)	18.6 (4.8 to 40.4)	22.0 (0.2 to 87.4)
**South Asia**								
South Asia	59.5 (52.4 to 66.4)	35.1 (17.6 to 54.1)	85.8 (73.2 to 91.7)	91.1 (43.9 to 99.8)	30.5 (26.0 to 35.5)	77.1 (46.1 to 89.7)	7.1 (4.2 to 10.9)	39.4 (2.3 to 85.1)
**South Asia**								
South Asia	59.5 (52.4 to 66.4)	35.1 (17.6 to 54.1)	85.8 (73.2 to 91.7)	91.1 (43.9 to 99.8)	30.5 (26.0 to 35.5)	77.1 (46.1 to 89.7)	7.1 (4.2 to 10.9)	39.4 (2.3 to 85.1)
Bangladesh	67.3 (57.3 to 78.3)	42.2 (31.6 to 53.6)	93.5 (86.1 to 97.4)	99.8 (99.1 to 100.0)	32.8 (27.9 to 38.2)	69.1 (21.0 to 97.5)	10.0 (2.3 to 24.0)	49.6 (1.4 to 98.3)
Bhutan	56.5 (52.0 to 61.3)	35.7 (24.3 to 47.7)	58.9 (48.6 to 69.1)	82.7 (33.4 to 99.1)	44.5 (39.5 to 49.6)	71.3 (38.8 to 92.1)	35.1 (21.2 to 49.2)	58.5 (15.6 to 92.1)
India	61.5 (52.8 to 69.8)	35.3 (14.2 to 60.3)	90.4 (74.2 to 97.4)	89.0 (26.8 to 100.0)	31.1 (26.5 to 36.2)	82.7 (40.5 to 98.8)	5.2 (1.3 to 12.2)	43.6 (1.5 to 97.2)
Nepal	82.9 (76.2 to 87.8)	33.4 (22.4 to 46.3)	96.9 (85.7 to 99.8)	99.8 (98.8 to 100.0)	40.0 (34.7 to 45.8)	71.9 (24.0 to 97.9)	14.7 (3.6 to 33.5)	58.9 (2.6 to 99.1)
Pakistan	35.1 (29.9 to 39.7)	29.2 (21.4 to 38.5)	51.6 (36.4 to 66.9)	96.3 (86.5 to 99.5)	24.0 (20.2 to 28.3)	50.5 (15.2 to 83.0)	15.1 (7.4 to 28.3)	5.2 (1.3 to 13.1)
**Southeast Asia, east Asia, and Oceania**								
Southeast Asia, east Asia, and Oceania	74.5 (67.1 to 80.6)	48.7 (34.4 to 61.4)	77.5 (56.5 to 89.2)	99.9 (99.7 to 100.0)	58.1 (52.6 to 63.7)	99.9 (99.6 to 100.0)	67.9 (59.1 to 76.4)	89.9 (53.6 to 99.3)
East Asia	70.1 (58.8 to 79.2)	46.6 (30.4 to 61.8)	76.1 (45.6 to 94.4)	100.0 (99.9 to 100.0)	55.9 (50.3 to 61.7)	100.0 (99.9 to 100.0)	65.6 (50.3 to 76.7)	85.5 (34.4 to 99.1)
China	69.8 (58.0 to 79.0)	47.1 (30.9 to 62.2)	75.4 (43.7 to 94.4)	100.0 (99.9 to 100.0)	55.9 (50.2 to 61.7)	100.0 (99.9 to 100.0)	66.9 (50.6 to 78.7)	85.4 (33.1 to 99.8)
North Korea	83.3 (72.0 to 91.1)	37.6 (12.1 to 67.2)	96.2 (82.5 to 99.7)	100.0 (99.9 to 100.0)	57.7 (52.1 to 63.4)	99.8 (99.2 to 100.0)	34.1 (10.9 to 62.3)	90.5 (35.0 to 100.0)

Taiwan	80.7 (68.4 to 88.5)	30.5 (11.4 to 59.2)	98.8 (96.3 to 99.8)	100.0 (100.0 to 100.0)	55.6 (49.9 to 61.4)	100.0 (100.0 to 100.0)	21.7 (5.7 to 46.5)	86.7 (41.3 to 99.5)
Oceania	75.5 (67.3 to 81.4)	39.2 (25.1 to 54.2)	75.3 (61.4 to 84.2)	96.7 (73.6 to 99.9)	54.4 (48.9 to 60.2)	86.5 (52.8 to 99.3)	54.4 (34.9 to 74.9)	90.8 (39.9 to 100.0)
American Samoa	85.6 (67.9 to 95.0)	52.9 (21.4 to 80.2)	91.1 (63.4 to 99.2)	100.0 (99.9 to 100.0)	55.6 (49.9 to 61.4)	99.9 (99.6 to 100.0)	91.4 (76.4 to 97.8)	99.8 (98.5 to 100.0)
Cook Islands	85.2 (79.1 to 90.0)	46.3 (16.9 to 75.2)	90.7 (76.2 to 97.6)	100.0 (99.9 to 100.0)	65.0 (59.7 to 70.3)	100.0 (100.0 to 100.0)	86.1 (65.0 to 96.2)	99.9 (99.5 to 100.0)
Fiji	80.4 (72.0 to 87.0)	54.6 (34.5 to 75.9)	90.3 (76.8 to 97.4)	99.9 (99.7 to 100.0)	39.7 (34.5 to 45.5)	99.6 (98.1 to 100.0)	60.5 (33.6 to 83.0)	96.8 (71.2 to 100.0)
Guam	87.8 (71.2 to 94.8)	57.2 (24.7 to 83.0)	93.3 (71.1 to 99.5)	100.0 (99.9 to 100.0)	51.1 (45.4 to 57.0)	99.9 (99.7 to 100.0)	94.8 (85.1 to 98.7)	99.9 (99.2 to 100.0)
Kiribati	84.8 (74.6 to 92.7)	57.1 (24.7 to 83.0)	84.8 (65.8 to 96.2)	99.7 (98.6 to 100.0)	43.7 (38.2 to 49.6)	74.8 (27.7 to 98.3)	89.6 (72.4 to 97.3)	95.1 (58.9 to 100.0)
Marshall Islands	73.8 (60.1 to 85.4)	52.6 (21.1 to 80.0)	72.4 (47.4 to 88.6)	99.8 (99.2 to 100.0)	40.6 (35.3 to 46.4)	93.4 (70.9 to 99.7)	72.5 (42.4 to 90.9)	94.4 (54.1 to 100.0)
Federated States of Micronesia	78.5 (68.0 to 87.8)	45.9 (16.7 to 74.9)	88.5 (71.5 to 97.7)	99.9 (99.4 to 100.0)	51.1 (45.5 to 57.0)	98.9 (94.8 to 100.0)	59.7 (28.1 to 84.1)	96.2 (66.6 to 100.0)
Nauru	85.8 (71.7 to 92.6)	41.5 (14.1 to 71.0)	91.5 (68.9 to 99.0)	99.9 (99.7 to 100.0)	54.8 (49.1 to 60.6)	99.9 (99.4 to 100.0)	54.1 (23.3 to 80.4)	98.4 (84.8 to 100.0)
Niue	75.9 (60.3 to 86.7)	49.4 (18.9 to 77.7)	76.6 (53.3 to 91.0)	100.0 (99.9 to 100.0)	61.8 (56.3 to 67.3)	100.0 (100.0 to 100.0)	92.8 (79.8 to 98.2)	100.0 (99.6 to 100.0)
Northern Mariana Islands	85.9 (71.2 to 94.9)	47.9 (17.9 to 76.5)	93.8 (73.0 to 99.5)	100.0 (99.9 to 100.0)	59.1 (53.6 to 64.7)	100.0 (100.0 to 100.0)	81.4 (56.1 to 94.5)	99.9 (98.9 to 100.0)
Palau	81.5 (75.6 to 86.8)	58.2 (35.8 to 76.8)	87.5 (73.0 to 96.7)	100.0 (99.9 to 100.0)	56.2 (50.5 to 61.9)	100.0 (99.9 to 100.0)	85.3 (63.4 to 95.9)	99.9 (99.0 to 100.0)
Papua New Guinea	80.2 (71.2 to 86.7)	37.0 (23.6 to 52.5)	74.5 (56.8 to 85.7)	95.8 (66.4 to 100.0)	54.8 (49.2 to 60.6)	83.6 (42.6 to 99.1)	52.0 (33.1 to 72.7)	89.1 (30.3 to 99.9)
Samoa	81.3 (72.5 to 88.9)	44.1 (20.5 to 72.4)	85.0 (69.6 to 95.6)	99.9 (99.4 to 100.0)	43.2 (37.7 to 49.1)	97.5 (88.2 to 99.9)	51.4 (21.3 to 78.5)	93.1 (46.6 to 100.0)
Solomon Islands	65.9 (59.0 to 73.9)	37.6 (19.4 to 57.3)	61.3 (50.6 to 72.0)	100.0 (99.9 to 100.0)	72.5 (67.9 to 77.1)	99.1 (95.8 to 100.0)	55.1 (35.7 to 75.0)	99.4 (94.0 to 100.0)
Tokelau	88.1 (71.1 to 93.9)	52.3 (20.9 to 79.7)	91.5 (64.7 to 99.3)	100.0 (99.9 to 100.0)	49.6 (44.0 to 55.6)	99.9 (99.7 to 100.0)	89.5 (72.1 to 97.2)	99.8 (98.2 to 100.0)
Tonga	79.5 (69.0 to 86.6)	52.5 (32.0 to 71.9)	81.9 (65.1 to 91.9)	100.0 (99.9 to 100.0)	51.7 (46.1 to 57.6)	98.8 (94.2 to 100.0)	86.3 (69.8 to 95.6)	98.6 (87.4 to 100.0)
Tuvalu	84.8 (69.3 to 93.1)	62.6 (29.6 to 86.2)	89.1 (62.9 to 98.5)	99.8 (99.0 to 100.0)	33.4 (28.5 to 38.9)	67.9 (19.9 to 97.3)	93.5 (81.6 to 98.4)	95.8 (63.3 to 100.0)
Vanuatu	70.6 (55.9 to 82.7)	39.0 (19.0 to 61.4)	62.1 (36.5 to 82.7)	99.7 (98.2 to 100.0)	53.2 (47.6 to 59.1)	93.0 (69.4 to 99.7)	60.0 (28.4 to 84.3)	91.8 (40.4 to 100.0)

Southeast Asia	84.8 (82.1 to 87.2)	53.1 (39.8 to 66.3)	80.6 (77.2 to 83.3)	99.9 (99.6 to 100.0)	62.7 (57.4 to 68.1)	100.0 (100.0 to 100.0)	73.0 (50.0 to 89.3)	99.0 (91.5 to 100.0)
Cambodia	57.0 (46.1 to 69.6)	35.8 (14.9 to 56.6)	42.8 (23.8 to 63.8)	99.9 (99.8 to 100.0)	76.7 (72.4 to 80.7)	100.0 (100.0 to 100.0)	75.5 (46.7 to 92.2)	99.5 (95.7 to 100.0)
Indonesia	78.0 (73.9 to 82.2)	58.0 (46.7 to 69.0)	74.0 (66.3 to 80.7)	100.0 (99.9 to 100.0)	60.9 (55.5 to 66.5)	100.0 (100.0 to 100.0)	71.6 (49.8 to 88.3)	99.0 (90.6 to 100.0)
Laos	73.3 (67.4 to 80.2)	45.2 (25.8 to 65.1)	78.3 (68.5 to 86.9)	95.9 (77.1 to 99.8)	68.8 (63.8 to 73.7)	100.0 (100.0 to 100.0)	61.0 (29.4 to 84.9)	98.4 (85.5 to 100.0)
Malaysia	81.8 (76.3 to 86.5)	55.2 (34.4 to 73.3)	94.4 (84.3 to 98.9)	99.8 (98.9 to 100.0)	58.9 (53.4 to 64.6)	100.0 (100.0 to 100.0)	75.6 (57.5 to 89.6)	99.8 (97.7 to 100.0)
Maldives	80.9 (75.9 to 85.2)	39.6 (26.8 to 55.0)	91.9 (82.4 to 96.7)	100.0 (99.9 to 100.0)	54.6 (49.0 to 60.4)	100.0 (100.0 to 100.0)	75.3 (61.1 to 87.0)	82.4 (49.2 to 97.1)
Mauritius	70.0 (55.1 to 82.2)	42.1 (17.9 to 71.8)	79.0 (49.8 to 96.6)	99.8 (99.1 to 100.0)	52.6 (46.9 to 58.5)	100.0 (100.0 to 100.0)	49.9 (22.4 to 75.7)	99.0 (90.5 to 100.0)
Myanmar	79.4 (70.7 to 87.1)	49.1 (32.7 to 66.4)	93.6 (81.5 to 98.8)	99.9 (99.8 to 100.0)	60.1 (54.6 to 65.7)	100.0 (99.9 to 100.0)	74.4 (46.5 to 91.4)	96.0 (64.7 to 100.0)
Philippines	90.8 (87.1 to 93.7)	56.1 (39.8 to 71.4)	77.2 (65.2 to 87.0)	99.9 (99.2 to 100.0)	58.5 (53.0 to 64.2)	100.0 (100.0 to 100.0)	80.4 (57.9 to 93.5)	100.0 (99.9 to 100.0)
Seychelles	71.7 (65.7 to 77.2)	35.1 (24.2 to 47.2)	90.2 (82.8 to 95.7)	100.0 (100.0 to 100.0)	69.2 (64.2 to 74.1)	100.0 (100.0 to 100.0)	49.7 (30.1 to 71.2)	100.0 (99.6 to 100.0)
Sri Lanka	82.4 (76.0 to 87.3)	50.1 (34.0 to 66.1)	93.7 (86.4 to 98.1)	100.0 (99.9 to 100.0)	47.0 (41.4 to 53.0)	100.0 (100.0 to 100.0)	59.0 (39.6 to 78.6)	97.1 (73.6 to 100.0)
Thailand	94.0 (91.8 to 96.0)	44.6 (29.1 to 62.2)	93.8 (85.8 to 98.4)	100.0 (99.9 to 100.0)	67.7 (62.7 to 72.8)	100.0 (100.0 to 100.0)	66.7 (45.8 to 84.9)	99.7 (97.2 to 100.0)
Timor-Leste	61.4 (51.5 to 69.5)	47.4 (33.1 to 64.3)	55.6 (40.3 to 70.0)	99.9 (99.7 to 100.0)	52.4 (46.8 to 58.3)	99.8 (99.0 to 100.0)	43.8 (25.4 to 66.1)	91.5 (38.9 to 100.0)
Viet Nam	81.3 (71.4 to 88.4)	47.2 (21.9 to 76.0)	86.3 (57.7 to 98.0)	100.0 (99.9 to 100.0)	73.0 (68.4 to 77.5)	100.0 (100.0 to 100.0)	75.5 (46.7 to 92.2)	99.8 (98.5 to 100.0)
**Sub-Saharan Africa**								
Sub-Saharan Africa	66.5 (61.9 to 70.6)	36.6 (31.2 to 43.0)	68.1 (61.8 to 73.3)	89.8 (81.6 to 93.4)	60.7 (55.9 to 65.6)	91.6 (76.9 to 97.5)	54.5 (44.1 to 64.4)	82.6 (55.7 to 94.1)
Central sub-Saharan Africa	68.9 (56.5 to 78.2)	37.3 (21.5 to 56.1)	70.4 (54.5 to 84.3)	84.4 (50.0 to 97.1)	55.5 (49.9 to 61.2)	89.8 (67.1 to 98.7)	61.0 (42.6 to 73.7)	83.9 (29.2 to 99.5)
Angola	80.9 (73.5 to 87.2)	27.5 (15.3 to 42.9)	99.0 (97.8 to 99.6)	95.6 (64.4 to 100.0)	51.1 (45.6 to 56.8)	98.3 (91.6 to 99.9)	47.9 (28.2 to 69.3)	74.4 (24.1 to 98.6)
Central African Republic	55.7 (40.8 to 69.7)	42.1 (14.4 to 71.5)	46.3 (7.7 to 86.1)	90.0 (36.0 to 100.0)	51.6 (46.0 to 57.6)	26.6 (2.4 to 78.6)	68.1 (37.0 to 88.8)	66.3 (4.4 to 99.5)
Congo (Brazzaville)	74.7 (67.6 to 80.4)	54.8 (34.1 to 73.0)	76.6 (62.4 to 86.4)	96.5 (70.5 to 100.0)	50.6 (45.0 to 56.5)	99.0 (95.1 to 100.0)	59.7 (40.2 to 79.1)	93.7 (49.9 to 100.0)
DR Congo	72.1 (55.5 to 82.8)	39.4 (13.0 to 69.0)	60.5 (36.3 to 81.0)	78.5 (24.1 to 99.2)	57.9 (52.4 to 63.7)	89.9 (59.2 to 99.5)	66.0 (34.6 to 87.7)	87.8 (26.4 to 99.9)

Equatorial Guinea	79.8 (67.7 to 87.5)	36.8 (11.7 to 66.4)	87.1 (51.7 to 98.8)	99.3 (94.8 to 100.0)	58.6 (53.1 to 64.3)	100.0 (99.8 to 100.0)	50.3 (20.5 to 77.6)	98.0 (81.5 to 100.0)
Gabon	65.7 (58.2 to 71.9)	48.5 (34.3 to 62.7)	70.1 (58.0 to 79.7)	96.9 (73.8 to 100.0)	44.0 (38.5 to 49.9)	99.7 (98.3 to 100.0)	49.2 (30.3 to 70.4)	89.0 (30.0 to 99.9)
Eastern sub-Saharan Africa	61.1 (56.9 to 64.5)	34.6 (27.0 to 43.9)	60.5 (57.3 to 63.5)	81.2 (70.7 to 87.4)	51.8 (46.4 to 57.5)	88.3 (70.2 to 94.7)	50.7 (33.9 to 68.5)	90.2 (77.5 to 96.1)
Burundi	63.2 (47.0 to 76.7)	35.7 (11.2 to 65.2)	58.3 (13.3 to 91.9)	53.0 (14.2 to 89.1)	65.4 (60.2 to 70.6)	92.4 (67.5 to 99.7)	64.2 (46.3 to 79.0)	98.8 (88.6 to 100.0)
Comoros	68.3 (55.5 to 79.6)	39.0 (12.8 to 68.6)	78.0 (32.9 to 97.3)	99.1 (92.8 to 100.0)	36.6 (31.5 to 42.2)	88.5 (55.0 to 99.4)	30.0 (9.1 to 57.4)	90.8 (36.2 to 100.0)
Djibouti	60.3 (46.0 to 74.6)	43.5 (15.3 to 72.8)	79.4 (35.2 to 97.6)	96.4 (64.3 to 100.0)	22.3 (18.5 to 26.6)	47.5 (7.6 to 92.4)	17.8 (4.6 to 39.2)	70.2 (5.8 to 99.6)
Eritrea	59.5 (52.3 to 67.0)	31.0 (14.1 to 52.0)	61.9 (49.1 to 73.3)	33.6 (18.1 to 51.1)	45.8 (40.3 to 51.8)	89.0 (56.4 to 99.5)	39.1 (21.8 to 60.7)	93.3 (47.5 to 100.0)
Ethiopia	48.3 (42.5 to 54.1)	24.8 (13.7 to 39.2)	49.3 (40.3 to 58.4)	64.7 (51.1 to 76.8)	49.7 (44.1 to 55.7)	86.2 (48.8 to 99.3)	51.6 (32.5 to 72.1)	95.2 (59.1 to 100.0)
Kenya	72.6 (67.8 to 77.3)	42.2 (30.8 to 53.9)	70.2 (61.9 to 77.6)	71.7 (54.3 to 84.6)	51.7 (46.1 to 57.6)	99.5 (97.7 to 100.0)	43.9 (25.7 to 65.8)	99.0 (90.9 to 100.0)
Madagascar	51.0 (40.9 to 59.8)	40.2 (22.2 to 61.2)	47.2 (23.2 to 71.6)	99.2 (93.8 to 100.0)	69.9 (65.0 to 74.8)	98.8 (94.0 to 100.0)	84.1 (61.0 to 95.5)	99.7 (97.3 to 100.0)
Malawi	65.4 (57.5 to 72.3)	40.6 (26.8 to 55.9)	66.8 (55.0 to 76.7)	94.0 (72.0 to 99.7)	55.8 (50.1 to 61.6)	95.2 (77.9 to 99.8)	46.9 (28.1 to 68.4)	98.0 (81.6 to 100.0)
Mozambique	67.7 (62.0 to 73.7)	48.9 (37.2 to 60.9)	61.8 (51.3 to 71.5)	96.2 (65.2 to 100.0)	55.3 (49.7 to 61.1)	85.4 (46.8 to 99.2)	41.6 (23.4 to 63.7)	96.2 (66.5 to 100.0)
Rwanda	72.8 (63.6 to 79.8)	34.7 (27.2 to 42.5)	75.9 (66.4 to 83.8)	82.8 (51.0 to 97.3)	29.9 (25.4 to 35.1)	74.3 (31.3 to 96.9)	47.5 (30.9 to 66.4)	59.8 (15.4 to 93.6)
Somalia	49.3 (30.9 to 68.9)	49.5 (23.6 to 76.9)	40.2 (5.7 to 81.9)	93.3 (39.9 to 100.0)	23.5 (19.6 to 28.0)	0.1 (0.0 to 0.5)	86.5 (65.8 to 96.3)	59.6 (2.7 to 99.1)
South Sudan	70.0 (57.1 to 81.3)	36.5 (11.5 to 66.0)	67.5 (20.1 to 94.9)	96.3 (63.4 to 100.0)	52.9 (47.2 to 58.7)	90.7 (61.4 to 99.6)	53.6 (23.0 to 80.1)	96.3 (67.3 to 100.0)
Uganda	75.5 (67.4 to 81.7)	31.7 (14.7 to 52.4)	70.7 (59.5 to 81.3)	96.8 (71.9 to 100.0)	56.3 (50.7 to 62.1)	97.9 (90.1 to 99.9)	39.8 (21.4 to 62.5)	100.0 (99.8 to 100.0)
Tanzania	63.9 (56.5 to 70.5)	30.9 (12.5 to 50.0)	64.8 (54.5 to 74.5)	89.4 (35.6 to 99.9)	58.7 (53.1 to 64.3)	100.0 (99.9 to 100.0)	43.8 (18.3 to 71.5)	70.6 (15.1 to 98.8)
Zambia	67.8 (62.4 to 74.0)	32.7 (19.9 to 45.9)	79.7 (71.5 to 86.8)	85.3 (70.8 to 94.4)	35.2 (30.3 to 40.7)	97.2 (87.4 to 99.9)	38.8 (23.1 to 56.7)	92.9 (55.8 to 99.9)
Southern sub-Saharan Africa	79.3 (74.5 to 82.7)	48.4 (38.4 to 59.5)	85.0 (79.0 to 88.8)	96.8 (82.6 to 99.6)	33.3 (28.9 to 38.1)	90.8 (75.7 to 98.1)	63.6 (47.6 to 75.7)	77.6 (52.8 to 91.5)
Botswana	64.0 (58.7 to 68.9)	41.9 (31.4 to 53.4)	68.8 (60.9 to 75.3)	99.8 (99.2 to 100.0)	36.2 (31.1 to 41.9)	98.8 (94.1 to 100.0)	57.8 (40.1 to 75.7)	92.8 (45.2 to 100.0)

Eswatini	75.5 (68.1 to 81.3)	45.7 (32.9 to 59.3)	75.9 (66.3 to 83.1)	99.7 (98.1 to 100.0)	27.7 (23.3 to 32.6)	86.2 (48.7 to 99.3)	66.9 (47.2 to 84.4)	78.1 (10.8 to 99.8)
Lesotho	80.4 (65.3 to 88.5)	41.6 (14.2 to 71.1)	74.9 (28.4 to 96.7)	99.4 (96.3 to 100.0)	34.5 (29.5 to 40.0)	79.2 (34.2 to 98.7)	63.5 (31.9 to 86.3)	77.0 (9.9 to 99.8)
Namibia	79.1 (69.5 to 84.9)	45.8 (24.9 to 64.3)	85.6 (66.8 to 96.1)	99.8 (98.9 to 100.0)	41.4 (36.0 to 47.2)	96.8 (86.7 to 99.8)	63.6 (43.1 to 81.6)	78.6 (29.4 to 98.5)
South Africa	81.6 (75.9 to 86.4)	53.5 (43.0 to 65.4)	88.4 (81.1 to 93.6)	97.0 (76.9 to 100.0)	29.2 (25.0 to 33.9)	90.0 (75.1 to 97.8)	65.3 (49.0 to 79.4)	73.7 (37.9 to 94.5)
Zimbabwe	76.3 (69.3 to 81.7)	30.5 (17.7 to 47.2)	75.6 (64.6 to 84.7)	94.1 (73.6 to 99.5)	47.8 (42.2 to 53.7)	93.1 (69.9 to 99.7)	57.4 (38.0 to 77.1)	91.1 (37.3 to 100.0)
Western sub-Saharan Africa	68.2 (64.8 to 71.2)	36.1 (30.3 to 41.5)	71.0 (64.2 to 76.9)	97.6 (93.2 to 99.3)	74.6 (70.2 to 78.8)	95.1 (86.7 to 99.6)	54.4 (49.6 to 58.5)	76.6 (38.3 to 94.9)
Benin	54.0 (48.0 to 59.7)	33.8 (20.3 to 50.5)	61.6 (51.8 to 70.4)	99.8 (98.9 to 100.0)	94.8 (93.5 to 95.8)	99.6 (98.2 to 100.0)	46.4 (27.5 to 66.9)	86.7 (23.5 to 99.9)
Burkina Faso	48.4 (42.7 to 54.4)	33.8 (23.4 to 45.3)	48.5 (40.0 to 56.4)	85.8 (70.2 to 94.9)	66.4 (61.2 to 71.5)	86.8 (50.3 to 99.3)	47.5 (28.8 to 68.6)	57.8 (2.4 to 99.0)
Cabo Verde	74.8 (66.2 to 80.7)	54.7 (32.4 to 74.2)	79.9 (65.6 to 88.6)	99.9 (99.4 to 100.0)	67.0 (61.9 to 72.1)	100.0 (100.0 to 100.0)	55.5 (24.5 to 81.4)	99.5 (95.4 to 100.0)
Cameroon	58.2 (52.0 to 64.6)	44.6 (31.6 to 57.4)	65.5 (55.0 to 75.6)	90.2 (74.0 to 97.7)	69.8 (64.8 to 74.6)	99.7 (98.6 to 100.0)	51.3 (32.3 to 72.2)	84.6 (19.2 to 99.9)
Chad	26.8 (20.2 to 34.3)	21.3 (11.1 to 35.7)	35.6 (25.6 to 46.5)	94.0 (72.1 to 99.7)	70.4 (65.6 to 75.2)	75.2 (28.1 to 98.3)	36.0 (17.0 to 60.3)	56.8 (2.3 to 98.9)
Côte d'Ivoire	59.5 (52.5 to 65.8)	29.0 (14.1 to 47.3)	83.6 (79.9 to 86.8)	99.8 (99.1 to 100.0)	70.7 (65.9 to 75.4)	100.0 (100.0 to 100.0)	39.7 (22.3 to 61.7)	80.8 (13.6 to 99.8)
The Gambia	41.4 (34.4 to 48.4)	38.5 (20.6 to 57.5)	49.9 (37.4 to 61.1)	65.3 (51.7 to 77.3)	66.0 (60.8 to 71.2)	98.8 (94.2 to 100.0)	33.5 (17.3 to 55.4)	73.1 (7.3 to 99.7)
Ghana	70.4 (65.5 to 75.3)	36.0 (25.1 to 48.3)	73.0 (64.9 to 79.9)	94.7 (69.6 to 99.9)	76.8 (72.6 to 80.9)	100.0 (99.9 to 100.0)	40.9 (25.2 to 59.2)	66.1 (13.1 to 97.7)
Guinea	70.4 (52.5 to 83.9)	33.3 (10.1 to 62.6)	78.9 (34.8 to 97.1)	99.5 (97.1 to 100.0)	73.3 (68.7 to 77.8)	97.6 (88.3 to 99.9)	59.3 (27.8 to 83.9)	79.0 (11.7 to 99.8)
Guinea-Bissau	67.9 (55.6 to 76.4)	33.8 (23.1 to 45.8)	68.2 (60.4 to 76.0)	99.7 (98.4 to 100.0)	70.5 (65.6 to 75.3)	97.8 (89.6 to 99.9)	49.2 (30.3 to 70.1)	73.5 (7.5 to 99.7)
Liberia	68.9 (53.6 to 80.1)	35.8 (11.2 to 65.3)	66.2 (39.8 to 85.1)	98.8 (90.1 to 100.0)	75.6 (71.3 to 79.8)	97.9 (89.8 to 99.9)	77.2 (49.3 to 92.9)	87.3 (25.0 to 99.9)
Mali	44.8 (34.7 to 54.7)	27.1 (9.9 to 53.9)	43.1 (23.4 to 64.1)	99.6 (97.2 to 100.0)	69.2 (64.2 to 74.1)	86.2 (48.8 to 99.3)	43.8 (23.9 to 66.5)	57.9 (2.4 to 99.0)
Mauritania	75.8 (67.2 to 83.1)	33.3 (10.1 to 62.5)	77.0 (61.6 to 87.9)	95.0 (55.6 to 100.0)	52.1 (46.4 to 58.0)	98.8 (94.3 to 100.0)	63.4 (51.8 to 73.5)	52.3 (1.7 to 98.6)
Niger	58.7 (43.2 to 71.5)	38.1 (18.1 to 56.3)	56.5 (28.7 to 80.2)	97.8 (79.9 to 100.0)	72.1 (67.4 to 76.7)	52.3 (9.6 to 94.0)	61.9 (37.6 to 82.5)	97.0 (73.1 to 100.0)

Nigeria	81.2 (76.9 to 84.8)	39.2 (24.6 to 53.8)	81.1 (70.1 to 89.2)	99.9 (99.6 to 100.0)	77.4 (73.3 to 81.4)	100.0 (100.0 to 100.0)	62.2 (41.2 to 81.3)	78.6 (32.8 to 98.3)
São Tomé and Príncipe	67.5 (53.7 to 78.2)	37.4 (12.0 to 67.0)	74.6 (50.4 to 90.1)	98.7 (90.4 to 100.0)	61.0 (55.5 to 66.5)	99.2 (95.9 to 100.0)	48.9 (19.5 to 76.6)	79.4 (12.1 to 99.8)
Senegal	63.4 (52.1 to 71.6)	36.2 (26.1 to 48.4)	65.6 (58.4 to 72.7)	99.7 (98.6 to 100.0)	64.7 (59.5 to 70.0)	96.8 (84.9 to 99.9)	46.1 (27.5 to 67.3)	69.5 (5.5 to 99.6)
Sierra Leone	76.5 (69.0 to 82.9)	20.9 (7.7 to 43.2)	84.6 (69.1 to 92.8)	97.2 (83.6 to 99.9)	77.9 (73.8 to 81.8)	99.5 (97.5 to 100.0)	40.4 (20.0 to 65.2)	89.0 (29.7 to 99.9)
Togo	22.1 (18.3 to 26.8)	22.7 (10.0 to 39.4)	23.6 (15.7 to 33.5)	99.9 (99.3 to 100.0)	76.9 (72.7 to 80.9)	99.9 (99.5 to 100.0)	43.8 (25.4 to 65.3)	90.5 (35.0 to 100.0)

Appendix 2 Figure S11: Sex composition of absolute and proportionate change in health worker densities between 1990 and 2023

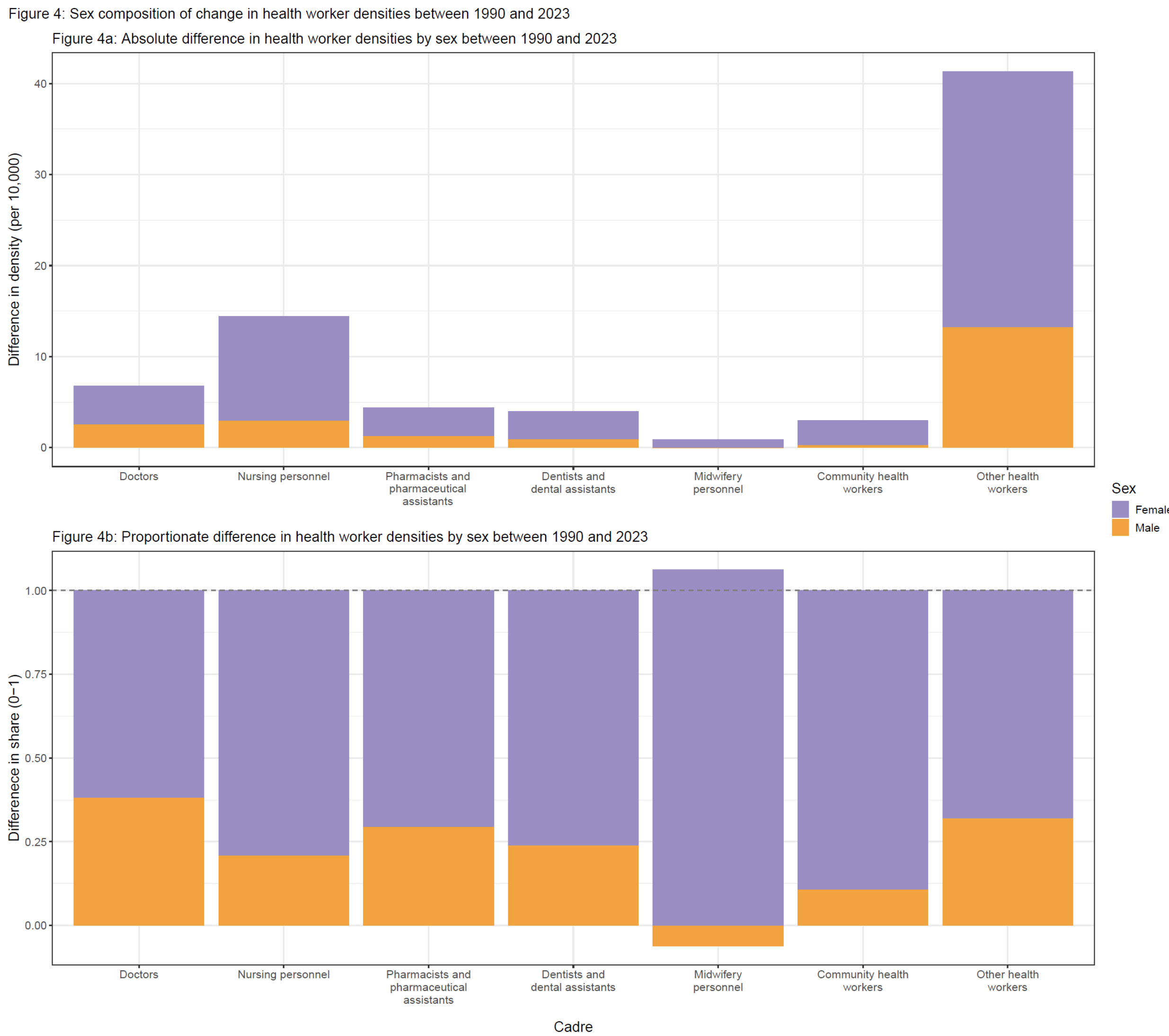

Appendix 2 Figure S12: Maximum possible UHC effective coverage index achievement given densities in 2023 of doctors (A), nurses and midwives (B), dentists (C), and pharmacists (D)

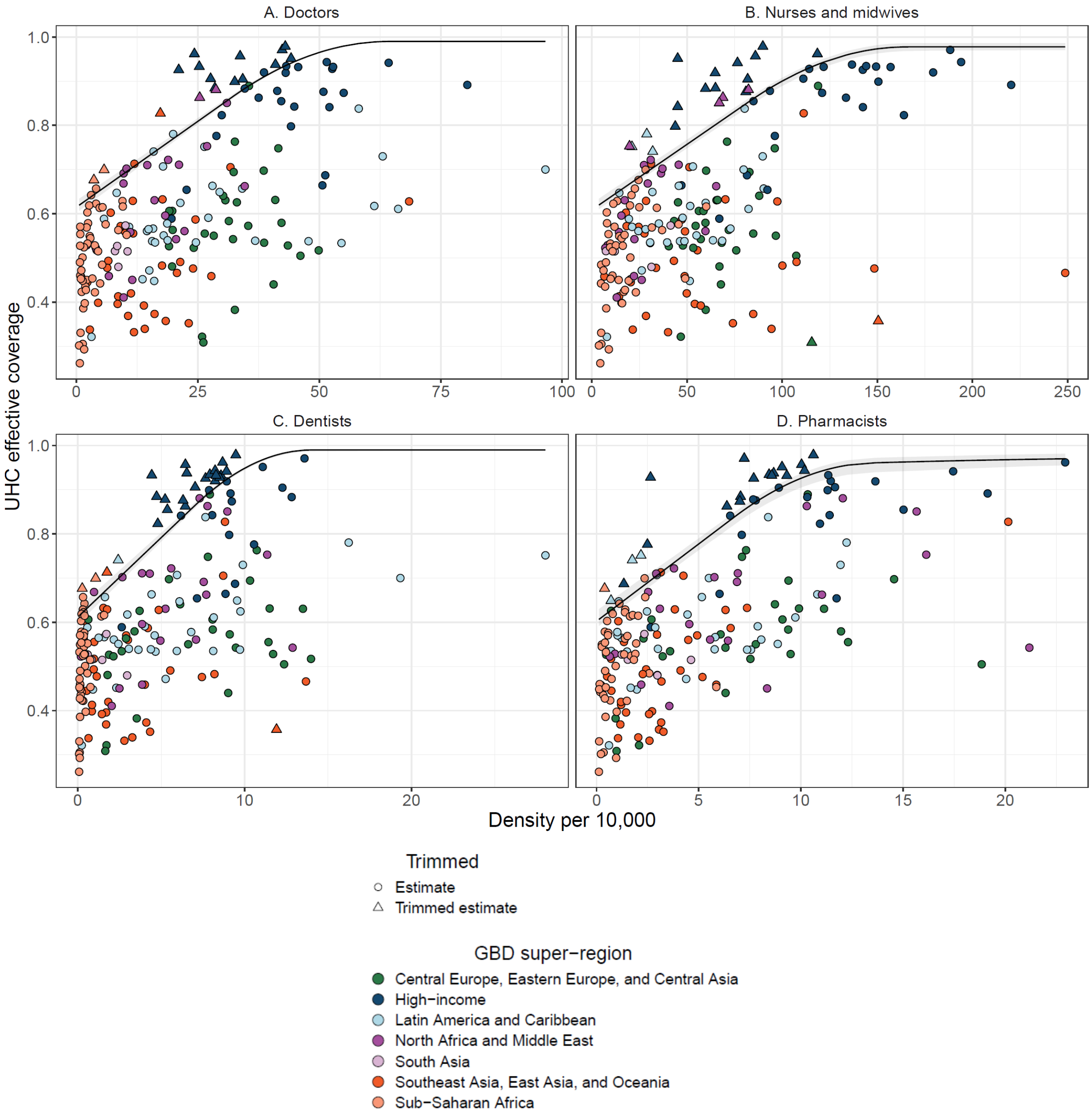

Supplement: Supplementary file 2 [file mmc2.pdf]
