# Supplementary material for: Measuring the composition and availability of human resources for health by sex in 204 countries and territories, 1990–2023, and workforce gaps for universal health coverage: a systematic analysis for the Global Burden of Disease Study 2023
Source: Lancet Public Health. 2026 Aug 18;11(9):e604–29. doi: 10.1016/S2468-2667(26)00145-3 (PMC13501144; doi:10.1016/S2468-2667(26)00145-3)
Supplement: Supplementary file 3 [file mmc3.pdf]

THE LANCET
Public Health

Supplementary appendix 3

This appendix formed part of the original submission and has been peer reviewed. We post it as supplied by the authors.

Supplement to: GBD 2023 Human Resources for Health Collaborators. Measuring the composition and availability of human resources for health by sex in 204 countries and territories, 1990–2023, and workforce gaps for universal health coverage: a systematic analysis for the Global Burden of Disease Study 2023. *Lancet Public Health* 2026;
11: 604–29.

Appendix 3: Authorship appendix to "Measuring the composition and availability of human resources for health by sex in 204 countries, 1990–2023, and workforce gaps for universal health coverage: a systematic analysis for the Global Burden of Disease Study 2023"

This appendix provides further authorship detail for "Measuring the composition and availability of human resources for health by sex in 204 countries, 1990–2023, and workforce gaps for universal health coverage: a systematic analysis for the Global Burden of Disease Study 2023"

Table of Contents

GBD 2023 Human Resources for Health Collaborators **3**

Affiliations **10**

Authors' Contributions **42**

Managing the overall research enterprise 42

Writing the first draft of the manuscript 42

Primary responsibility for applying analytical methods to produce estimates 43

Primary responsibility for seeking, cataloguing, extracting, or cleaning data; designing or coding figures and tables 43

Providing data or critical feedback on data sources 43

Developing methods or computational machinery 46

Providing critical feedback on methods or results 46

Drafting the work or revising it critically for important intellectual content 51

Managing the estimation or publications process 56

GBD 2023 Human Resources for Health Collaborators

Megan Knight, Kelly Bienhoff, Bhoomadevi A, Hazim S Ababneh, Madineh Abbasi, Zahra Abbasi Dolatabadi, Mitra Abbasifard, Samar Abd ElHafeez, Ashraf Nabiel Abdalla, Reda Abdel-Hameed, Atef Abdelkader, Sherief Abd-Elsalam, Deldar Morad Abdulah, Auwal Abdullahi, Meklit Girma Abebe, Tesfaye Birhanu Abebe, Habtamu Abebe Getahun, Armita Abedi, Asrat Agalu Abejew, E S Abhilash, Alemwork Abie, Richard Gyan Aboagye, Abdullahi Tunde Aborode, Nagah M. Abourashed, Mohamed Abouzid, Lucas Guimarães Abreu, Michael R M Abrigo, Dariush Abtahi, Amr Selim Abu Lila, Bilyaminu Abubakar, Rana Kamal Abu-Farha, Eman Abu-Gharbieh, Ahmad Y Abuhelwa, Hana J Abukhadijah, Anirudh Balakrishna Acharya, Krishna Prasad Acharya, Swetha Acharya, Maryam Adabi, Lisa C Adams, Nuhu Lawan Adamu, Isaac Yeboah Addo, Olusegun Adetomiwa Adediran, Kamoru Ademola Adedokun, Oluwatobi E Adegbile, Olumide Thomas Adeleke, Miracle Ayomikun Adesina, Ridwan Olamilekan Adesola, Charles Oluwaseun Adetunji, Juliana Bunmi Adetunji, Ripon Kumar Adhikary, Atman Adiba, Prince Owusu Adoma, Aanuoluwapo Adeyimika Afolabi, Muhammad Sohail Afzal, Saira Afzal, Victor Ibukun Agbajelola, Feleke Doyore Doyore Agide, Percival Delali Agordoh, César Agostinis Sobrinho, Prince Agwu, Williams Agyemang-Duah, Adamu Adamu Ahmad, Aqeel Ahmad, Danish Ahmad, Khabir Ahmad, Muayyad M Ahmad, Rabbiya Ahmad, Sajjad Ahmad, Suleiman Idris Ahmad, Ali Ahmadi, Asma Ahmed, Ayman Ahmed, Jahanzaib Mian Ahmed, Junaid Ahmed, Mehrunnisha Sharif Ahmed, Muktar Beshir Ahmed, Mushood Ahmed, Syed Anees Ahmed, Marjan Ajami, Muhammad Nadeem Akhtar, Samina Akhtar, Wole Akosile, Mohammad Khaled Al Nawayseh, Yazan Al Thaher, Omar Ali Mohammed Al Zaabi, Mohammad Ahmmad Mahmoud Al Zoubi, Tariq Al Zoubi, Timothy Olukunle Aladelusi, Fares Alahdab, Ziyad Al-Aly, Khurshid Alam, Mohammad Khursheed Alam, Mohammad Jahoor Alam, Pravej Alam, Zufishan Alam, Rasmieh Mustafa Al-Amer, Fahad Mashhour Alanezi, Turki M Alanzi, Fahmi Y Al-Ashwal, Hussin Albargi, Mohammed Albashtawy, Jacqueline Elizabeth Alcalde-Rabanal, Khalifah A Aldawsari, Mohammed S Aldossary, Robert W Aldridge, Shereen M Aleidi, Bezawit Abeje Alemayehu, Ayman Al-Eyadhy, Ali M Alfalki, Abdelazeem M Algammal, Robert Kaba Alhassan, Ashraf Alhumaidi, Irfan Ali, Kamran Ali, Liaqat Ali, Mohammad Daud Ali, Mohammed Usman Ali, Rafat Ali, Syed Shujait Ali, Akram Al-Ibraheem, Montaha Al-Iede, Esubalew Muluneh Aligaz, Hamid Alinejad Rokny, Cyrus Alinia, Morteza Alipour, Samah W Al-Jabi, Moath Saleh Aljohani, Syed Mohamed Aljunid, Wesam Taher Almagharbeh, Mohammed J. Almalki, Md. Al-Mamun, Sabah Al-Marwani, Joseph Uy Almazan, Mohmmad Minwer Alnaeem, Khaldoon Aied Alnawafleh, Mahmoud A Alomari, Saleh A Alqahtani, Mohammad AlQudah, Intima Alrimawi, Sahel Majed Alrousan, Ali Salman Al-Shami, Mamdouh Alshammari, Abdalkarem Fedgash Alsharari, Alaa B Al-Tammemi, Malik A Althobiani, Nelson Alvis-Guzman, Yaser Mohammed Al-Worafi,

Mohammad Sharif Ibrahim Alyahya, Karem H Alzoubi, Masoud Aman Mohammadi, McKing Izeiza Amedari, Faten Amer, Amr Amin, Saeed Amini, Majid Aminzare, Sohrab Amiri, Ganiyu Adeniyi Amusa, Jimoh Amzat, Pedro Prata Andrade, Song Peng Ang, Blake Angell, Nguyen Hoang Anh, Samuel Egyakwa Ankomah, Kabilan Annadurai, Boluwatife Stephen Anuoluwa, Iyadunni Adesola Anuoluwa, Saeid Anvari, Sumadi Lukman Anwar, Razique Anwer, Anayochukwu Edward Anyasodor, Jalal Arabloo, Mosab Arafat, Aleksandr Y Aravkin, Demelash Areda, Abdulfatai Aremu, Ghazal Arjmand, Jesu Arockiaraj, Mahwish Arooj, Anton A Artamonov, Nabiy Arystan, Syed Mohammed Basheeruddin Asdaq, Saeed Asgary, Nestor Asiamah, Muhammad Shahzad Aslam, Gizew Dessie Asres, Anil Raj Assariparambil, Batyrbek Assembekov, Seyyed Shamsadin Athari, Sulaiman Olusegun Atiku, Ba-Etilayoo Atinga, Prince Atorkey, Alok Atreya, Avinash Aujayeb, Nyan Min Aung, Sana Javaid Awan, Kemal Lemnuro Awol, Camila Ospina Ayala, Martin Amogre Ayanore, Mohd Yusmaidie Aziz, Ahmed Y Azzam, Zaharaddeen Shuaibu Babandi, Fatemeh Baberi, Rasha Babiker, Abraham Samuel Babu, Syed Babar Jamal Bacha, Alaa Aboelnour Badran, Youngoh Bae, Abdul Rasheed Bahar, Awsan Abdullah Saeed Bahattab, Razieh Bahreini, Ruhai Bai, Atif Amin Baig, Lovenish Bains, Alpha Umaru Bai-Sesay, Mohamad Amin Bakhshali, Shankar M Bakkannavar, Abdulaziz T Bako, Pachamuthu Balakrishnan, Senthilkumar Balakrishnan, Wondu Feyisa Balcha, Ovidiu Constantin Baltatu, Biswajit Banik, Aleksandra Barac, Till Winfried Bärnighausen, Hiba Jawdat Barqawi, Cornelia Anne Barth, Zarrin Basharat, Mohammad-Mahdi Bastan, Abdul-Monim Batiha, Kavita Batra, Samuel Debas Bayable, Mulat Tirfie Bayih, Zemenu Wube Bayleyegn, Narasimha M Beeraka, Diana Fernanda Bejarano Ramirez, Bekalu Mekonen Belay, Asnake Gashaw Belayneh, Aminu K Bello, Samiun Nazrin Bente Kamal Tune, Kelemu Zelalem Berhanu, Robert S Bernstein, Nikha Bhardwaj, Pankaj Bhardwaj, Sonu Bhaskar, Priyadarshini Bhattacharjee, Gurjit Kaur Bhatti, Jasvinder Singh Bhatti, Rajbir Bhatti, Zulfiqar A Bhutta, Sibhatu Kassa Biadgilign, Bijit Biswas, Mohammad Shahangir Biswas, Fassikaw Kebede Bizuneh, Micheal Kofi Boachie, Trupti Bodhare, Somayeh Bohlouli, Lucimere Bohn, Aime Bonny, Sudipta Bose, Souad Bouaoud, Dejana Braithwaite, Datonye Christopher Briggs, Ovidiu-Iulian Bunea, Felix Busch, Reinhard Busse, Yasser Bustanji, Zahid A Butt, Jack Cagney, Ismael Campos-Nonato, Si Cao, Yu Cao, Mate Car, Rosario Cárdenas, Andre F Carvalho, Felix Carvalho, Alberico L Catapano, Pamela Roxana Chacón-Uscamaita, Rama Mohan Chandika, Miyuru Chandradasa, Viswa Chaitanya Chandu, Jung-Chen Chang, Qinghua Chang, Vijay Kumar Chattu, Lam Duc Chau, Anis Ahmad Chaudhary, Sirshendu Chaudhuri, Akhilanand Chaurasia, Che Mohd Nasril Che Mohd Nassir, Fentahun Alemnew Chekole, Haowei Chen, Mingling Chen, Yun-Jung Choi, Bryan Chong, Hitesh Chopra, Shivani Chopra, Sonali Gajanan Choudhari, Dinh-Toi Chu, Jinky Marie T. Chua, Isaac Sunday Chukwu, Sunghyun Chung, Arrigo Francesco Giuseppe Cicero, Fred Cohen, Joao Conde, Claudia Cosma,

Natalia Cruz-Martins, Ali Dabbagh, Omid Dadras, Bishal Dahal Khatri, Xiaochen Dai, Maxwell Ayindenaba Dalaba, Mayank Dalakoti, Emanuele D'Amico, Anh Kim Dang, Samuel Demissie Darcho, Reza Darvishi Cheshmeh Soltani, Mauro Henrique Nogueira Guimarães de Abreu, Jan-Walter De Neve, Ivan Delgado-Enciso, Endalkachew Dellie, Dessalegn Demeke, Ismail Dergaa, Nikolaos Dervenis, Muamer Dervišević, Aragaw Tesfaw Desale, Vinoth Gnana Chellaiyan Devanbu, Syed Masudur Rahman Dewan, Thakur Dhakal, Rajinder K Dhamija, Amol S Dhane, Sreedhar Dharmagadda, Marcello Di Pumpo, Adriana Dima, Delaney D Ding, Isaac Oluwafemi Dipeolu, Huyen Phuc Do, Mai Ngoc Do, Mario D'Oria, Fariba Dorostkar, Ojas Prakashbhai Doshi, Robert Kokou Dowou, Emeka W. Dumbili, Senbagam Duraisamy, Oyewole Christopher Durojaiye, Anar Dushpanova, Ejemai Eboreime, Ikenna D Ebuenyi, Afework Edmealem, David Edvardsson, Ferry Efendi, Abiodun Egwuenu, Michael Ekholuenetale, Rabie Adel El Arab, Mohamed Ahmed Eladl, Muhammed Elhadi, Yasir Ahmed Mohammed Elhadi, Salem Elkahoui, Randa Elsheikh, Chadi Eltaha, Stanley Chinedu Eneh, Majid Eslami, Fahima Nasrin Eva, Elochukwu Ezenwankwo, Adewale Oluwaseun Fadaka, Heidar Fadavian, Adeniyi Francis Fagbamigbe, Ayesha Fahim, Ildar Ravisovich Fakhradiyev, Mohammad Fareed, Aisha Farhana, Liliana Faria, Andre Faro, Fatemeh Farshad, Folorunso Oludayo Fasina, Davood Fathi, Ginenus Fekadu, Abdullah H Feroze, Pietro Ferrara, Marta Figueiredo, Safia Firdous, Florian Fischer, Federica Fogacci, Artem Alekseevich Fomenkov, Arianna Fornari, Alina-Ioana Forray, Alberto Freitas, Takeshi Fukumoto, Nancy Fullman, Adam Fusheini, Peter Andras Gaal, Muktar A Gadanya, Dominic Dormenyo Gadeka, Anna D Gage, Márió Gajdács, Balasubramanian Ganesh, Ezhilkugan Ganessane, Fernando Jr. Barroga Garcia, Chandi Garg, Jacopo Garlasco, Rupesh K Gautam, Tsion S Gebre, Beza A Gelaw, Stefano Gelibter, Julian Lassiter Gendreau, Kabiru Ishola Genty, Gebremariam Wulie Geremew, Genanew Kassie Getahun, Kalab Yigermal Gete, Mustafa Ghaderzadeh, Fataneh Ghadirian, Kazem Ghaffari, Leila Ghalichi, Arin Ghamkhar, Ali Ghandili, Ahmad Ghashghaee, Sailaja Ghimire, Ali Gholamrezanezhad, Vaitsa Giannouli, Artyom Urievich Gil, Syed Abdullah Gilani, Bikash Ranjan Giri, Alem Abera Girmay, Mahaveer Golechha, Pouya Goleij, Davide Golinelli, Melika Golmohammadi, Yitayal Ayalew Goshu, Barbara Niegia Garcia Goulart, Tulasi Govardhan, Ayman Grada, Giovanni Guarducci, Stefano Guicciardi, Sheffali Gulati, Kabiru Abubakar Gulma, Damitha Asanga Gunawardane, Lei Guo, Aayush Gupta, Bhawna Gupta, Rajeev Gupta, Sapna Gupta, Vijai Kumar Gupta, Roberth Steven Gutiérrez-Murillo, Jose Guzman-Esquivel, Farrokh Habibzadeh, Zahra Hadian, Khalid Rehman Rehman Hakeem, Alex Hakuzimana, Pritam Halder, Kosar Hikmat Hama Aziz, Ayman M. Hamdan-Mansour, Mohamed Hamed, Parvaneh Hamian Roumiani, Samer Hamidi, Asif Hanif, Mohd Anul Haq, Josep Maria Haro, Mohammad Jahid Hasan, S. M. Mahmudul Hasan, Towhid Hasan, Ali Hasanpour- Dehkordi, Arezou Hashem Zadeh, Ahmad Shoeb Hashmi, Md Saquib Hasnain, Amr Hassan, Ikrama

Hassan, Md. Imtaiyaz Hassan, Simon I Hay, Ahmad Hayatu, Mohammad Heidari, Majid Heydari, Thomas Kwadwo Hinneh, Yuta Hiraike, Ramesh Holla, Yu Hong, Mehdi Hoseinzadeh, Lubna Hossain, Md Mahbub Hossain, Mohammad Bellal Hossain, Mihaela Hostiuc, Sorin Hostiuc, Guoqing Hu, Junjie Huang, Yongsong Huang, Ayesha Humayun, Javid Hussain, Olayemi Joshua Ibidoja, Aminu Alhassan Ibrahim, Umar Idris Ibrahim, Anel Ibrayeva, Pulwasha Maria Iftikhar, Aqsa Ikram, Jibran Ikram, Olayinka Stephen Ilesanmi, Irena M Ilic, Milena D Ilic, Mohammad Tarique Imam, Arit Inok, Meesha Iqbal, Mujahid Iqbal, Usman Iqbal, Lalu Muhammad Irham, Fahrul Islam, Md Rabiul Islam, Md Sahidul Islam, Md. Shahinul Islam, Nahlah Elkudssiah Ismail, Gaetano Isola, Yussif Issaka, Masao Iwagami, Chidozie Declan Iwu, Jaison Jacob, Louis Jacob, Udeme Samuel Jacob, Ali Jadidi, Shabbar Jaffar, Nader Jahanmehr, Haitham Jahrami, Mihajlo Jakovljevic, Reza Jalilzadeh Yengejeh, Armaan Jamal, Anju James, Jerin James, Masoud Jamshidi, Rajiv Janardhanan, Mu'taman Jarrar, Krishna Mahendrabhai Jasani, Talha Jawaid, Sathish Kumar Jayapal, Shubha Jayaram, Yovanthi Anurangi Jayasinghe, Jae Joon Jeon, Seogsong Jeong, Worku Jimma, Mohammad Jokar, Jobinse Jose, Akaninyene Paul Joseph, Nitin Joseph, Charity Ehimwenma Joshua, Farahnaz Joukar, Jacek Jerzy Jozwiak, Sang-Hyuk Jung, Malik E Juweid, Hema Shree K, Dler Hussein Kadir, Adel Kadri, Ashish Kumar Kakkar, Khalil Kalavani, Feroze Kaliyadan, Aidana Kaliyakparova, Sanjay Kalra, Rajesh Kamath, Ramat T. Kamorudeen, Hesam Kamyab, Jiseung Kang, Kehinde Kazeem Kanmodi, Rami S Kantar, Mehrdad Karajizadeh, Paschalis Karakasis, Jafar Karami, Marina Karanikolos, Mohmed Isaqali Karobari, Faizan Zaffar Kashoo, Nehemia I. Kassa, Nicholas J Kassebaum, Kanica Kaushal, Foad Kazemi, Vikash Ranjan Keshri, Aïmen Khacharem, Yousef Saleh Khader, Himanshu Khajuria, Asaad Khalid, Anees Ahmed Khalil, Ajmal Khan, Faiz Ullah Khan, Iqra Hamid Khan, MD Sazzad Khan, Mohd Faheem Khan, Muhammad Arslan Khan, Muhammad Umer Khan, Ramsha Mushtaq Khan, Serab Khan, Sumaiya Khan, Yusuf Saleem Khan, Zahid Khan, Sameer Uttamaro Khasbage, Khaled Khatab, Haitham Khatatbeh, Moawiah Mohammad Khatatbeh, Hamid Reza Khayat Kashani, Afshin Khazaei, Khalid A Kheirallah, Zahra Khorrami, Parisa Khoshvaght, Mahmood Khosrowjerdi, Jinho Kim, Kwanghyun Kim, Min Seo Kim, Ruth W Kimokoti, Adnan Kisa, Shivakumar KM Kondlahalli, Sonali Kochhar, Prakash Babu Kodali, Michail Kokkorakis, Ali-Asghar Kolahi, Farzad Kompani, Tapos Kormoker, Oleksii Korzh, Michal Koscik, Archana Koul, Sindhura Lakshmi Koulmane Laxminarayana, Arthi S Kozhumam, Irene Akwo Kretchy, Kewal Krishan, Bindu Krishnan, Mohammed Kuddus, Shikha Kukreti, Mukhtar Kulimbet, Emmanuel Kumah, Chandan Kumar, Dewesh Kumar, Kamal Kumar, Laksh Kumar, Manasi Kumar, Mukesh Kumar, Nitesh Kumar, Pradeep Kumar, Rakesh Kumar, Sandeep Kumar, Sanjeet Kumar, Vijay Kumar, Senthil Kumaran D, Satyajit Kundu, Jibin Kunjavara, Almagul Kurmanova, Donata Kurpas, Christina Yeni Kustanti, Dian Kusuma, Tezer Kutluk, Assylkhan Kuttybayev, Michael

Agyemang Kwarteng, Ville Kytö, Muhammad Awwal Ladan, Desireé Alexa LaGrappe, Chandrakant Lahariya, Daphne Teck Ching Lai, Hanpeng Lai, Dharmesh Kumar Lal, Judit Lám, Ariane Laplante-Lévesque, Anders O Larsson, Savita Lasrado, Areeba Latif, Basira Kankia Lawal, Eilean Rathinasamy Lazarus, Caterina Ledda, Munjae Lee, Sergey Vadimovich Lee, Seung Won Lee, Wei-Chen Lee, Yo Han Lee, Vasileios Leivaditis, Francesco Leonforte, Chengfeng Li, Honghong Li, Ming-Chieh Li, Shaojie Li, Virendra S Ligade, Jialing Lin, Jue Liu, Xianliang Liu, Zihao Liu, Madeeha Shahzad Lodhi, Niroshan Chandrajith Lokunarangoda, Jaifred Christian F Lopez, José Francisco López-Gil, Arianna Maever Loreche, Masoud Lotfizadeh, Surbala Devi Lourembam, Giancarlo Lucchetti, Hengliang Lv, Miltiadis D Lytras, Ellina Lytvyak, Hawraz Ibrahim M. Amin, Kevin Sheng-Kai Ma, Monika Machoy-Rakoczy, Samatar Abshir Mahamed, Nozad Hussein Mahmood, Shakeel Ahmed Ibne Mahmood, Kafayat Mahmoud, Hao Xuan Mai, Ghaseb Naser Makhadmeh, Irsa Fatima Makhdoom, Ahmad Azam Malik, Kamaruddeen Mannethodi, Marjan Mansourian, Tahir Maqbool, Hamed Markazi Moghadam, Adolfo Martinez-Valle, J Jenifer Florence Mary, Roy Rillera Marzo, Sammer Marzouk, Yasith Mathangasinghe, Manu Raj Mathur, Neeta Mathur, Fernanda Penido Matozinhos, Khurshid A Mattoo, Richard James Maude, Suleiman Mayaki, Maryam Mazaheri, Martin McKee, Steven M McPhail, Ravi Mehrotra, Vini Mehta, Tadesse Belayneh Melkie, Ziad Ahmed Memish, Godfred Antony Menezes, Emiru Ayalew Mengistie, Leweyehu Alemaw Mengstie, Atte Meretoja, Tuomo J Meretoja, Giovanni Merlino, Muayad Aghali Merza, Tomislav Mestrovic, Chamila Dinushi Kukulege Mettananda, Sachith Mettananda, Mohamed M. M. Metwally, Sandrine Donfack D Mewoabi, Muhammad Agus Mikrajab, Giuseppe Minervini, Wai-Kit Ming, GK Mini, Andreea Mirica, Erkin M Mirrakhimov, Vinaytosh Mishra, Chaitanya Mittal, Dhruti Modi, Mona Gamal Mohamed, Nouh Saad Mohamed, Khabab Abbasher Hussien Mohamed Ahmed, Taj Mohammad, Sakineh Mohammad-Alizadeh-Charandabi, Omer Mohammed, Shafiu Mohammed, Syam Mohan, Yugal Kishore Mohanta, Siba Sankar Mohanty, Ali H Mokdad, TJ Robinson T Moncatar, Himel Mondal, Mohammad Ali Moni, Marco Montalti, Mohammad Moradi-Joo, Danilo Farias Morais, Shane Douglas Morrison, Reza Mosaddeghi Heris, Freiser Eceomo Cruz Mosquera, Elias Mossialos, Kimia Mozahheb Yousefi, Nicollas Mozart Vieira, Matías Mrejen, Rabia Mubarak, Sumaira Mubarik, Jibran Sualeh Muhammad, Amartya Mukhopadhyay, Joshua Kanaabi Muliira, Francesk Mulita, Kavita Munjal, Sani Musa, David Musoke, Ghulam Mustafa, Mubarak Taiwo Mustapha, Sathish Muthu, Claude Mambo Muvunyi, Lutfi Muzaqi, Woojae Myung, Ayoub Nafei, Mobin Naghshbandi, Hiten Naik, Firzan Nainu, Hastyar Hama Rashid Najmuldeen, Noureddin Nakhostin Ansari, Gopal Nambi, Abdulqadir J Nashwan, Mahmoud Nassar, Samidi Nirasha Kumari Navaratna, Biswa Prakash Nayak, Kinjal Nayak, Vinod C Nayak, Javad Nazari, Mojdeh Nazari, Ionut Negoi, Ruxandra Irina Negoi, Alina Gabriela Negru, Chakib Nejjari, Nikita Nekliudov, Cuong Tat

Nguyen, Hien Thu Nguyen, Huong Lan Thi Nguyen, Tham Thi Nguyen, Trang Nguyen, Yeshambel T Nigatu, Ali Nikoobar, Shuhei Nomura, Mamoona Noreen, Emira Noumi, Mohammed Noushad, Chisom Adaobi Nri-Ezedi, Fred Nugen, Bogdan Oancea, Akinyemi O D Ofakunrin, Oluwakemi Christie Ogidan, Ayomide Samuel Ogunrinde, Olusegun Olatunji Ojedoyin, Olalekan John Okesanya, Osaretin Christabel Okonji, John Olayemi Okunlola, Oluwaseyi Isaiah Olabisi, Musa Kolapo Oladejo, Andrew T Olagunju, Oladotun Victor Olalusi, Abimbola Olaniran, Gláucia Maria Moraes Oliveira, Abdulhakeem Abayomi Olorukooba, Bolajoko Olubukunola Olusanya, Oluwafemi G. Oluwole, Ahmed Omar Bali, Obinna E Onwujekwe, Aksoltan Shyhdurdyevna Oradova, Michal Ordak, Verner N Orish, Atakan Orscelik, Esteban Ortiz-Prado, Godwin Osawaru Osaigbovo, Augustus Osborne, George Nkrumah Osei, Godfred Otchere, Adrian Otoiu, Jerry John Ouner, Amel Ouyahia, Simon Øverland, Mayowa O Owolabi, Oladayo Ayobami Oyebanji, Kehinde Adewole Oyeniran, Ilker Ozsahin, Jagadish Rao Padubidri, Vijayakumar Palaniswamy, Tamás Palicz, Adrian Pana, Sujogya Kumar Panda, Bipindra Pandey, Prateek Pandya, Santa Maria Pangaribuan, Anca Pantea Stoian, Ilias Papadimopoulos, Shahina Pardhan, Romil R Parikh, Chulwoo Park, Arpit Parmar, Maja Pasovic, Roberto Passera, Mitesh Patel, Neel Navinkumar Patel, Shankargouda Patil, Dimitrios Patoulias, Apurba Patra, Snigdha Pattnaik, Uttam Paudel, Shrikant Pawar, Hamidreza Pazoki Toroudi, Paolo Pedersini, Veincent Christian Filipino Pepito, Prince Peprah, Maria Odete Pereira, Mario F P Peres, Simone Perna, Olumuyiwa James Peter, Tung Thanh Pham, Michael R Phillips, Zahra Zahid Piracha, Edoardo Pirera, Mapa M Prabhath N Piyasena, Evgenii Plotnikov, Dimitri Poddighe, Ion Popa, Fabio Porru, Sajjad Pourasghary, Mohsen Poursadeqiyan, Manya Prasad, Akila Prashant, Elton Junio Sady Prates, Harsh Priya, Nicola Riccardo Pugliese, Jagadeesh Puvvula, Muhammad Usman Qamar, Nameer Hashim Qasim, Xiang Qi, Navid Rabiee, Reza Rabiei, Venkatraman Radhakrishnan, Pracheth Raghuveer, Sajjad Rahimi, Afarin Rahimi-Movaghar, Vafa Rahimi-Movaghar, Firman Suryadi Rahman, Fryad Majeed Rahman, Mahbubur Rahman, Mosiur Rahman, Muhammad Aziz Rahman, Pramila Rai, Adarsh Raja, Ramya Raja Reddy, Judah Rajendran, Kairolla Dyusenbayevich Rakhimov, Rafał Rakoczy, Shakthi Kumaran Ramasamy, Pramod W Ramteke, Lokesh Rana, Nemanja Rancic, Manjula Rangappa, Magesh Rangasamy, Chythra R Rao, Kumuda Rao, Mithun Rao, Sowmya J Rao, Devarajan Rathish, Jigyasa Rathore, Ramin Ravangard, Rashita Ravi, Renju Ravi, David Laith Rawaf, Salman Rawaf, Lal Rawal, Ramu Rawat, Ayita Ray, Muhammad Liaquat Raza, Bahman Razi, C. Mahony Reategui-Rivera, Najeeb Ur Rehman, Lennart Reifels, Bhagwan Narayan Rekadwad, Serge Resnikoff, Stefano Restaino, Luis Felipe Reyes, Nazila Rezaei, Nima Rezaei, Mohsen Rezaeian, Muhammad Riaz, Jennifer Rickard, Syed Mohd Danish Rizvi, Jefferson Antonio Buendia Rodriguez, Leonardo Roever, Ravi Rohilla, Moustaq Karim Khan Rony, Kevin T Root, Himanshu Sekhar Rout, Adrija Roy, Gaurav Kumar Roy,

Sharmistha Roy, Godfrey Mutashambara Rwegerera, Chandan S N, Aly M A Saad, Korosh Saber, Siamak Sabour, Bashdar Abuzed Sadee, Fatemeh Sadeghi-Ghyassi, Hossein Sadr, Mohammad Reza Saeb, Umar Saeed, Maryam Saeedi, Sahar Saeedi Moghaddam, Muhammad Safiullah, Rajesh Sagar, Amene Saghazadeh, Indranil Saha, Fatemeh Saheb Sharif-Askari, Narjes Saheb Sharif-Askari, Vaibhav Sahni, Ambika Sahoo, Kirti Sundar Sahu, S Mohammad Sajadi, Mirza Rizwan Sajid, Joseph W Sakshaug, Afeez Abolarinwa Salami, Bukola Salami, Samreen Saleem, Mohamed A Saleh, Aanuoluwa James Salemcity, Pegah Salimi Pormehr, Giovanni A Salum, Hossein Samadi Kafil, Janmejaya Samal, Abdallah M Samy, Elaheh Sanjari, Sathish Sankar, Lucas H C C Santos, Milena M Santric-Milicevic, Malik Adebayo Sanusi, Sivan Yegnanarayana Iyer Saraswathy, Yaser Sarikhani, Hemen Sarma, Mohammad Sarmadi, Gargi Sachin Sarode, Sachin C Sarode, Michele Sassano, Mukesh Kumar Sathya Narayanan, Maheswar Satpathy, Amar Saurabh, Sangeeta Gopal Saxena, Yaser Sayadi, Ahmed M. Sayed, Christophe Schinckus, Soraya Seedat, Durairaj Sekar, Chandrabose Selvaraj, Siddharthan Selvaraj, Yuliya Semenova, Fikadu Waltengus Sendeku, Subramanian Senthilkumaran, Sadaf G Sepanlou, Edson Serván-Mori, Francesco Sessa, Yashendra Sethi, Abubakar Sha'aban, Shipraa Arpan Shah, Sweni Shah, Muhammad Shahab, Samiah Shahid, Wajeehah Shahid, Endrit Shahini, Farshad Shahkarami, Ali Shahriari, Moyad Jamal Shahwan, Babar Tasneem Shaikh, Masood Ali Shaikh, Ali Shakerimoghaddam, Alfiya Shamsutdinova, Dan Shan, Mohd Shanawaz, Nadim Sharif, Amin Sharifan, Prof. Fariborz Sharifian Jazi, Avimanu Sharma, Bhoopesh Kumar Sharma, Jyoti Sharma, Kamal Sharma, Kamlesh Sharma, Manoj Sharma, Vishal Sharma, Ramzi Shawahna, Anas Shehadeh, Somia Shehzadi, Ali Sheidaei, Rekha Raghuveer Shenoy, Suchitra M Shenoy, Samendra P Sherchan, Mahabalesh Shetty, Pavanchand Shetty, Premalatha K Shetty, Muhammad J. A. Shiddiky, Tariku Shimels, Md Monir Hossain Shimul, Priyanka Arun Shirali, Shirindokht Shirazi, Aminu Shittu, Km Shivangi, Azad Shokri, Sina Shool, Seyed Afshin Shorofi, Sunil Shrestha, Suleiman Adeiza Shuaibu, Akhenaten Benjamin Siankam Tankwanchi, Negussie Boti Sidamo, Emmanuel Edwar Siddig, Arif J Siddiqui, Gustavo Correia Basto da Silva, Padam Prasad Simkhada, David Larbi Simpong, Colin R Simpson, Abhinav Singh, Baljinder Singh, Bhoopendra Singh, Brij Pal Singh, Harmanjit Singh, Jasvinder A Singh, Jawahar Singh, Mayank Singh, Puneetpal Singh, Shilpy Singh, Siddharth Singh, Mukesh Kumar Sinha, Ratnesh Sinha, Gisha Sivan, Natia Skhvitaridze, Valentin Yurievich Skryabin, Mejdi Snoussi, Farrukh Sobia, Md Salman Sohel, Solikhah Solikhah, Jukun Song, Michael Spartalis, Lokasani Vijaya Sree, Chandrashekhar T Sreeramareddy, Shyamkumar Sriram, Panagiotis Stachteas, Simona Cătălina Ștefan, Brendon Stubbs, Peter Stubbs, Chen-Yang Su, Omer Subasi, Vetriselvan Subramaniyan, Aswin Sugunan, Mahwish Suhaib, Oksana Sulaieva, Sahabi K Sulaiman, Mark J M Sullman, Yan Sun, Zhong Sun, Thanigaivel Sundaram, David Sunkersing, Chandan Kumar Swain,

Shreya P Swami, Lukasz Szarpak, Payam Tabaee Damavandi, Rafael Tabarés-Seisdedos, Seyed-Amir Tabatabaeizadeh, Shima Tabatabai, Ramin Tabibi, Mohd Tabish, Biniyam Beyene Tabor, Zanan Mohammed-Ameen Taha, Niloofar Taheri, Jabeen Taiba, Mircea Tampa, Jianye Tan, Ker-Kan Tan, Baimakhan Tanabayev, Dariga Tanabayeva, Shynar Tanabayeva, Shenglan Tang, Ekamol Tantisattamo, Mohsan Tanveer, Hongliang Tao, Anika Tasnim, Nathan Y Tat, Vivian Y Tat, Aigul Yelgondiyevna Tazhiyeva, Mohamad-Hani Temsah, Wegen Beyene Tesfamariam, Azimeraw Arega Tesfu, Pugazhenthan Thangaraju, Kavumpurathu Raman Thankappan, Ismaeel Tharwat, Rasiah Thayakaran, Jeevarathinam Thirumalai, Muthu Thiruvengadam, Rekha Thiruvengadam, Jansje Henny Vera Ticoalu, Mariya Vladimirovna Titova, Vikas Kumar Tiwari, Madi Tleshev, Santo Imanuel Tonapa, Roman Topor-Madry, Stephanie M Topp, Marcos Roberto Tovani-Palone, Quynh Thuy Huong Tran, Thang Huu Tran, Nguyen Tran Minh Duc, Domenico Trico, Quynh Xuan Nguyen Truong, Gary Tse, Vasilis-Spyridon Tseriotis, Evangelia Tsermpini, Lorainne Tudor Car, Atta Ullah, Riaz Ullah, Saeed Ullah, Shehu Salihu Umar, Era Upadhyay, Jibrin Sammani Usman, Dilber Uzun Ozsahin, Hande Uzunçıbuk, Pascual R Valdez, Sven Vanneste, Joe Varghese, Santosh Varughese, Tommi Juhani Vasankari, Anoop Velayudhan, Balachandar Vellingiri, U Venkatesh, Narayanaswamy Venketasubramanian, David Villarreal-Zegarra, Tuan Vinh, Luciano Magalhães Vitorino, Vasily Vlassov, Ave Voit, Simona Ruxandra Volovat, Linh Vu, Yasir Waheed, Ethan Waisberg, Eric Lee Wan, Jin-Yi Wan, Haozhen Wang, Shaopan Wang, Shu Wang, Wanzhou Wang, Wei Wang, Xuequan Wang, Yan Wang, Yanzhong Wang, Youxin Wang, Mary Njeri Wanjau, Paul R Ward, Wesley Warriner, Toyiba Hiyaru Wassie, Anggi Lukman Wicaksana, Nuwan Darshana Wickramasinghe, Samuel Wiebe, Angga Wilandika, Martin Wiredu Agyekum, Axel Walter Wolf, Zhijia Xia, Wanqing Xie, Saba Yahoo, Haibo Yang, Yuichiro Yano, Haiqiang Yao, Haya Khader Ahmad Yasin, Yuichi Yasufuku, Sanni Yaya, Kuanysh A. Yergaliyev, Subah Abderehim Yesuf, Saber Yezli, Xinglin Yi, Dehui Yin, Yulai Yin, Yazachew Engida Yismaw, Dong Keon Yon, Naohiro Yonemoto, Mustafa Younis, Hairui Yu, Yong Yu, Quan Yuan, Ghazala Yunus, Siddhesh Zadey, Manijeh Zaghampour, Dilmurat Zairov, Nelson Zamora, Aurora Zanghì, Kourosh Zarea, Ahmad Zarei, Michael Zastrozhin, Saad Zbiri, Mohammed G M Zeariya, Zahra Zeinali, Ruba A Zenati, Eyael M Zeru, Julio Min Fei Zhang, Chenyu Zhao, Yingxi Zhao, Jinxin Zheng, Anthony Zhong, Hao Zhou, Bin Zhu, Zhaohua Zhu, Abzal Zhumagaliuly, Mohamed Ali Zoromba, Alimuddin Zumla, Ahed H Zyoud, Sa'ed H Zyoud, Shaher H Zyoud, Enis Bariş, Rafael Lozano, Christopher J L Murray, Annie Haakenstad

Affiliations

Institute for Health Metrics and Evaluation (M Knight BS, K Bienhoff MA, R W Aldridge PhD, A Y Aravkin PhD, J Cagney MSc, X Dai PhD, N Fullman MPH, Prof S I Hay DSc, N J Kassebaum MD, T Mestrovic PhD, Prof A H Mokdad PhD, N Nekliudov MD, M Pasovic MEd, W Warriner BA, Prof E Bariş PhD, Prof R Lozano

MD, Prof C J L Murray DPhil, A Haakenstad ScD), Department of Health Metrics Sciences, School of Medicine (R W Aldridge PhD, A Y Aravkin PhD, X Dai PhD, Prof S I Hay DSc, N J Kassebaum MD, Prof A H Mokdad PhD, Prof R Lozano MD, Prof C J L Murray DPhil), Department of Applied Mathematics (A Y Aravkin PhD), School of Health Systems and Public Health (C Iwu MPH), Department of Anesthesiology & Pain Medicine (N J Kassebaum MD), Department of Global Health (S Kochhar MD, Z Zeinali MD), Health Systems and Population Health (A B Siankam Tankwanchi PhD), University of Washington, Seattle, WA, USA; Amity Institute of Public Health and Hospital administration (Prof B A PhD), Amity University Noida, Uttar Pradesh, India; Department of Radiation Oncology (H S Ababneh MD), Department of Anesthesia, Critical Care and Pain Medicine (Prof J Kang PhD), Department of Orthopaedics (O Subasi PhD), Massachusetts General Hospital, Boston, MA, USA (M Kim MD); Infectious and Tropical Research Center (M Abbasi PhD), Social Determinants of Health Research Center (Prof S Mohammad-Alizadeh-Charandabi PhD), Midwifery Department (Prof S Mohammad-Alizadeh-Charandabi PhD), Neurosciences Research Center (NSRC) (R Mosaddeghi Heris MD), Student Research Committee (R Mosaddeghi Heris MD), Drug Applied Research Center (S Pourasghary MD, H Samadi Kafil PhD), Tabriz University of Medical Sciences, Tabriz, Iran (F Sadeghi-Ghyassi PhD); Department of Medical-surgical Nursing (Z Abbasi Dolatabadi PhD), Non-communicable Diseases Research Center (M Bastan MD, N Rezaei MD), Dentistry Research Institute (F Farshad DDS), Department of Immunology (J Karami PhD), Children's Medical Center (Prof F Kompani MD), Department of Physiotherapy (Prof N Nakhostin Ansari PhD), Research Center for War-affected People (Prof N Nakhostin Ansari PhD), Iranian National Center for Addiction Studies (Prof A Rahimi-Movaghar MD), Sina Trauma and Surgery Research Center (Prof V Rahimi-Movaghar MD, S Shool MD), Research Center for Immunodeficiencies (Prof N Rezaei PhD, A Saghazadeh MD), Endocrinology and Metabolism Population Sciences Institute (S Saeedi Moghaddam MSc), Department of Research and Development (P Salimi Pormehr MSc), Department of Epidemiology and Biostatistics (E Sanjari PhD, E Sanjari PhD), Digestive Diseases Research Institute (S G Sepanlou MD), Department of Internal Medicine (F Shahkarami MD), School of Public Health (A Sheidaei PhD), Tehran University of Medical Sciences, Tehran, Iran (K Ghaffari PhD); Department of Internal Medicine (M Abbasifard MD), Clinical Research Development Unit (M Abbasifard MD), Department of Epidemiology and Biostatistics, Health School, Occupational Environment Research Center (Prof M Rezaeian PhD), Rafsanjan University of Medical Sciences, Rafsanjan, Iran; Department of Epidemiology (S Abd ElHafeez DrPH), Alexandria University, Alexandria, Egypt; College of Pharmacy (Prof A N Abdalla PhD), Umm Al-qura University, Makka, Saudi Arabia; Basic Science Department (Prof R Abdel-Hameed PhD), University of Ha’il, Hail, Saudi Arabia; Chemistry Department (Prof R Abdel-Hameed PhD), Department of Zoology and Entomology (M G M Zeariya PhD), Al-Azhar University, Cairo, Egypt; Department of Mathematics and Sciences (A Abdelkader PhD), College of Engineering and Information Technology (M K Al Nawayseh PhD), Center for Medical and Bio-Allied Health Sciences Research (Prof M J Shahwan PhD), Ajman University, Ajman, United Arab Emirates; Department of Tropical Medicine and Infectious Diseases (S Abd-Elsalam PhD), Tanta University, Egypt, Tanta, Egypt; Community and Maternity Nursing Unit (D M Abdulah MPH), Duhok Research Centre (Z M Taha PhD), University of Duhok, Duhok, Iraq; Department of Physiotherapy (A Abdullahi PhD), Department of Community Medicine (Prof M A Gadanya MD), Department of Nursing Science (M Ladan PhD), Bayero University Kano, Kano, Nigeria; Department of Physiotherapy (A Abdullahi PhD), Federal University Wukari, Wukari, Nigeria; Department of Pediatrics and Child Health (M G Abebe MD), Landmark General Hospital, Addis Ababa, Ethiopia; School of Medicine (T B Abebe MD), Salale University, Fitche, Ethiopia; Department of Epidemiology and Biostatistics (H Abebe Getahun MSc), Department of Social and Administrative pharmacy (Z W

Bayleyegn MSc), Department of Health Systems and Policy (E Dellie MPH), College of Medicine and Health Science (G W Geremew MSc), School of Medicine and Institute of Public Health (T B Melkie PhD), University of Gondar, Gondar, Ethiopia; Department of Emergency Medicine (A Abedi MD), Department of Food Safety and Hygiene (M Aminzare PhD), Department of Immunology (S Athari PhD), Zanjan University of Medical Sciences, Zanjan, Iran; School of Pharmacy (A Abejew MSc), Department of Midwifery (A Abie MSc, B A Alemayehu MSc, W F Balcha MSc, F A Chekole MSc, F W Sendeku MSc, A A Tesfu MSc, T H Wassie MSc), Department of Anesthesia, College of Medicine and Health Sciences (E M Aligaz MSc), Anesthesia and critical care (S D Bayable MSc), Department Nutrition and Dietetics (M T Bayih MSc), Department of Emergency and Critical Care Nursing (A G Belayneh MSc), Department of Physiology (D Demeke MSc), College of Medicine and Health Science (K Y Gete MD), Department of Adult Health Nursing (E A Mengistie MSc), Department of Pharmacology (Y E Yismaw MSc), Bahir Dar University, Bahir Dar, Ethiopia; Department of Botany (E S Abhilash PhD), Sree Narayana Guru College Chelannur, Kozhikode, India; Department of Family and Community Health (R G Aboagye MPH), Department of Nutrition and Dietetics (P D Agordoh MPhil), Institute of Health Sciences (R K Alhassan PhD), Department of Health Policy Planning and Management (M A Ayanore PhD), Institute of Health and Allied Sciences (M A Dalaba PhD), Department of Epidemiology and Biostatistics (R K Dowou MPhil), Department of Microbiology and Immunology (Prof V N Orish PhD), University of Health and Allied Sciences, Ho, Ghana; School of Population Health (R G Aboagye MPH, Prof V R Keshri PhD), Centre for Social Research in Health (I Y Addo PhD), School of Biomedical Engineering (Prof H Alinejad Rokny PhD), International Centre for Future Health Systems (J Lin PhD), School of Optometry and Vision Science (Prof S Resnikoff MD), University of New South Wales, Sydney, NSW, Australia; Department of Research and Development (A T Aborode MSc), Healthy Africans Platform, Ibadan, Nigeria; Zoology Department (N M Abourashed PhD), Benha University, Benha 13518, Egypt; Department of Physical Pharmacy and Pharmacokinetics (M Abouzid PharmD), Poznan University of Medical Sciences, Poznan, Poland; Department of Pediatric Dentistry (Prof L Abreu PhD), Department of Community and Preventive Dentistry (Prof M N G de Abreu PhD), Department of Maternal-Child Nursing and Public Health (Prof F P Matozinhos PhD, E J S Prates BS), Department of Applied Nursing (Prof M O Pereira PhD), Faculty of Dentistry (Prof G C B D Silva PhD), Federal University of Minas Gerais, Belo Horizonte, Brazil; Philippine Institute for Development Studies, Quezon City, Philippines (M R M Abrigo PhD); Department of Anesthesiology (D Abtahi MD), Department of Epidemiology (Prof A Ahmadi PhD, Prof S Sabour PhD), National Nutrition and Food Technology Research Institute (Prof M Ajami PhD, Z Hadian PhD), School of Medicine (G Arjmand MD, M Golmohammadi MD), Research Institute of Dental Sciences (Prof S Asgary MSc), Department of Epidemioloy (F Baberi PhD), Department of Anesthesia, Critical Care and Pain Medicine (Prof A Dabbagh MD), Department of Health Information Technology and Management (M Ghaderzadeh PhD, R Rabiei PhD), School of Nursing and Midwifery (F Ghadirian PhD), Department of Health Policy and Management, School of Public Health And Safety (N Jahanmehr PhD), Department of Neurosurgery (H Khayat Kashani MD), Ophthalmic Epidemiology Research Center, Research Institute for Ophthalmology and Vision Science, Shahid Beheshti University of Medical Sciences, Tehran, Iran (Z Khorrami PhD), Social Determinants of Health Research Center (Prof A Kolahi MD, A Nikoobar MD), School of Allied Medicine (M Nazari PhD), Department of Medical Education (S Tabatabai PhD), Shahid Beheshti University of Medical Sciences, Tehran, Iran; Department of Pharmaceutics (Prof A S Abu Lila PhD, S Rizvi PhD), Department of Biology, College of Science (M Alam PhD), Department of Biology (M Alshammari PhD, A J Siddiqui PhD), Department of Biology, College of Sciences (Prof S Elkahoui PhD), College of Medicine (Y S Khan MD), Department of Biochemistry (Prof M Kuddus PhD), Department of

Health Management (R Kumar PhD), College of Science- Biology Department (Prof E Noumi PhD), Biology Department (Prof M Snoussi PhD), Department of Basic Science (G Yunus PhD), Department of Public Health (M G M Zeariya PhD), University of Hail, Hail, Saudi Arabia; Department of Pharmacology and Toxicology (B Abubakar PhD), Department of Sociology (Prof J Amzat PhD), Department of Veterinary Public Health and Preventive Medicine (A Shittu MSc), Department of Pharmaceutical Microbiology and Biotechnology (S Shuaibu PhD), Usmanu Danfodiyo University, Sokoto, Sokoto, Nigeria; Nigerian Institute of Medical Research, Lagos, Nigeria (B Abubakar PhD); Clinical Pharmacy and Therapeutics Department, Faculty of Pharmacy (Prof R K Abu-Farha PhD), Applied Science Research Center (A B Al-Tammemi MPH), Applied Science Private University, Amman, Jordan; Clinical Sciences Department (Prof E Abu-Gharbieh PhD, H J Barqawi MPhil, N Saheb Sharif-Askari PhD), Department Pharmacy Practice and Pharmacotherapeutics (A Y Abuhelwa PhD), Department of Restorative Dentistry (A B Acharya PhD), College of Pharmacy (S M Aleidi PhD), College of Medicine (Prof A Amin PhD, Prof M A Saleh PhD), Department of Basic Biomedical Sciences (Prof Y Bustanji PhD), Department of Basic Medical Sciences (M A Eladl PhD), Sharjah Institute of Medical Sciences (F Saheb Sharif-Askari PhD), Research Institute for Medical and Health Sciences (Prof D Uzun Ozsahin PhD, R A Zenati MSc), University of Sharjah, Sharjah, United Arab Emirates; Department of Biopharmaceutics and Clinical Pharmacy (Prof E Abu-Gharbieh PhD), School of Nursing (Prof M M Ahmad PhD), School of Business (M K Al Nawayseh PhD), Department of Radiology and Nuclear Medicine (Prof M E Juweid MD), University of Jordan, Amman, Jordan; Clinical Trials Unit (H J Abukhadijah MPH), Nursing & Midwifery Research Department (NMRD) (J Kunjavara PhD, A J Nashwan PhD), Corporate Nursing and Midwifery Research Department (K Mannethodi MPH), Hamad Medical Corporation, Doha, Qatar; Department of Livestock Services (DLS) (K Acharya Mphil), Government of Nepal (GoN), Lalitpur, Nepal; Department of Oral Pathology and Microbiology (S Acharya PhD), Department of Biochemistry (Prof A Prashant PhD), JSS Academy of Higher Education and Research, Mysuru, India; Research Center for Food Hygiene and Safety, School of Public Health, Shahid Sadoughi University of Medical Sciences, Yazd, Iran (M Adabi PhD), Shahid Sadoughi University of Medical Sciences, Yazd, Iran; Department of Diagnostic and Interventional Radiology (L C Adams PhD), School of Medicine and Health (F Busch MD), Technical University of Munich, Munich, Germany; Department of Psychiatry and Behavioral Sciences (N Taheri MD), Department of Biomedical Data Sciences (E Tsermpini PhD), Stanford University, Palo Alto, CA, USA (L C Adams PhD); Department of Nursing Science (N L Adamu PhD), Modibbo Adama University, Yola., Yola, Nigeria; School of Medicine (I Y Addo PhD), University of Sydney, Sydney, NSW, Australia; Department of Ophthalmology, Faculty of Clinical Sciences, College of Medicine (O A Adediran MSc), Department of Physiotherapy (M A Adesina BPT), Department of Oral and Maxillofacial Surgery (T O Aladelusi FWACS), Department of Health Promotion and Education (I O Dipeolu PhD), Department of Epidemiology and Medical Statistics (A F Fagbamigbe PhD), Department of Special Education (U Jacob PhD), College of Medicine (O I Olabisi PhD), Department of Medicine (O V Olalusi MD, Prof M O Owolabi DrM), University of Ibadan, Ibadan, Nigeria; Department of Ophthalmology (O A Adediran MSc), Department of Oral and Maxillofacial Surgery (T O Aladelusi FWACS, A A Salami BDS), Department of Community Medicine (O S Ilesanmi PhD), Department of Neurology (O V Olalusi MD), Department of Medicine (Prof M O Owolabi DrM), University College Hospital, Ibadan, Ibadan, Nigeria; Department of Immunology (K A Adedokun MSc), Roswell Park Comprehensive Cancer Center, Buffalo, NY, USA; Graduate Program Division (K A Adedokun MSc), University at Buffalo, Buffalo, NY, USA; Center for Cardiovascular Risk Research (O E Adegbile MD), East Tennessee State University, Johnson City, TN, USA; Center for Disease Control and Prevention (O E Adegbile MD), Cephas Health Research Initiative Inc.,

Ilesa, Nigeria; Department of Family Medicine (O T Adeleke MD), College of Health Sciences (O I Olabisi PhD), Bowen University, Iwo, Nigeria; Department of Family Medicine (O T Adeleke MD), Bowen University Teaching Hospital, Ogbomoso, Nigeria; Slum and Rural Health Initiative Research Academy (M A Adesina BPT), Slum and Rural Health Initiative, Ibadan, Nigeria; Department of Veterinary Medicine and Surgery (R Adesola DVM), Department of Pathobiology and Intergrative Biomedical Sciences (V I Agbajelola MVSc), Department of Biomedical Informatics, Biostatistics, and Epidemiology (F Alahdab MD), University of Missouri, Columbia, MO, USA; Department of Microbiology (Prof C O Adetunji PhD), Edo State University Uzairue, Iyamho, Nigeria; Department of Biochemistry (J B Adetunji PhD), Osun State University, Osogbo, Nigeria; Department of Fisheries and Marine Bioscience (R K Adhikary PhD), Jashore University of Science and Technology, Jashore, Bangladesh; Research School of Population Health (R K Adhikary PhD), School of Medicine and Psychology (D Ahmad PhD), Australian National University, Canberra, ACT, Australia; Department of Biotechnology (A Adiba PhD), National Institute for Agronomic Research, Morocco, Beni Mellal, Morocco; Department of Health Administration and Education (P O Adoma PhD), University of Education Winneba, Winneba, Ghana; Technical Services Directorate (A A Afolabi MPH), MSI Nigeria Reproductive Choices, Abuja, Nigeria; Department of Life Sciences (M S Afzal PhD), University of Management and Technology, Lahore, Pakistan (A Latif BS); Department of Community Medicine (Prof S Afzal PhD), King Edward Memorial Hospital, Lahore, Pakistan; Department of Public Health (Prof S Afzal PhD), Public Health Institute, Lahore, Pakistan; Department of Public Health (F D Agide PhD), Kampala International University, Kampala, Uganda; SPRINT Sport Physical Activity and Health Research & Innovation Center (C Agostinis Sobrinho PhD), Polytechnic Institute of Guarda, Guarda, Portugal; Health Research and Innovation Sciences Center (C Agostinis Sobrinho PhD), Polytechnic Institute of Guarda, Klaipeda, Lithuania; University of Dundee (P Agwu PhD), University of Dundee, Dundee, UK; Health Policy Research Group (P Agwu PhD), University of Nigeria, Enugu, Nigeria; Department of Public Health Sciences (W Agyemang-Duah PhD), Queen's University, Kingston, ON, Canada; Department of Physical Therapy (A A Ahmad PhD), University of Nizwa, Birkat Al Mawz, Oman; College of Medicine (A Ahmad PhD, Prof G Mustafa MD), Shaqra University, Shaqra, Saudi Arabia; Health Research Institute (D Ahmad PhD), University of Canberra, Canberra, NSW, Australia; King Khaled Eye Specialist Hospital and Research Center, Riyadh, Saudi Arabia (Prof K Ahmad PhD); Department of Clinical Pharmacy (R Ahmad PhD), Advanced Medical & Dental Institute (M Aziz PhD), Universiti Sains Malaysia, Penang, Malaysia; Department of Pharmacy (R Ahmad PhD), Institute of Molecular Biology and Biotechnology (A Ahmed PhD, A Ikram PhD, T Maqbool PhD), University Institute of Diet and Nutritional Sciences (M Akhtar PhD, A Khalil PhD), University College of Medicine & Dentistry (Prof M Arooj PhD), University Institute of Public Health (I H Khan PhD), Institute of Molecular Biology and Biotechnology (IMBB) (M Khan PhD, M S Lodhi PhD, S Shahid PhD), Research Centre for Health Sciences (RCHS) (S Shahid PhD), Department of Physics (W Shahid PhD), University Institute of Medical Lab Technology (S Shehzadi PhD), The University of Lahore, Lahore, Pakistan; Department of Health and Biological Sciences (S Ahmad PhD), Abasyn University, Peshawar, Pakistan; Department of Natural Sciences (S Ahmad PhD), Lebanese American University, Beirut, Lebanon; Vaccines and Immunity Department (S Ahmad MPH), Medical Research Council Unit, The Gambia, Fajara, The Gambia; Department of Epidemiology and Biostatistics (Prof A Ahmadi PhD), Community-Oriented Nursing Midwifery Research Center (M Heidari PhD), Department of Community Health (M Lotfizadeh PhD), Social Determinants of Health Research Center (M Lotfizadeh PhD), Shahrekord University of Medical Sciences, Shahrekord, Iran; INTI International University Malaysia, Nilai, Malaysia (A Ahmed PhD); Institute of Endemic Diseases (A Ahmed PhD), Faculty of Medicine (K Mohamed Ahmed

MD), Unit of Basic Medical Sciences (E E Siddig MD), University of Khartoum, Khartoum, Sudan; Pan-African One Health Institute (PAOHI), Kigali, Rwanda (A Ahmed PhD); Health Department (J M Ahmed PhD), Sandeman Provincial Hospital, Quetta, Pakistan; Faculty of Pharmaceuticals and Allied Health Sciences (J M Ahmed PhD), University of Balochistan Quetta, Quetta, Pakistan; Manipal College of Dental Sciences,Mangalore (Prof J Ahmed MDS), Department of Community Medicine (N Joseph MD), Kasturba Medical College (G A Menezes PhD), Department of Forensic Medicine and Toxicology (Prof J Padubidri MD), Manipal Academy of Higher Education, Mangalore, India; College of Nursing (M S Ahmed MSc), Department of Computer and Information Science (M Haq PhD), Department of Physical Therapy and Health Rehabilitation (Prof F Z Kashoo PhD), Majmaah University, Al Majmaah, Saudi Arabia; College of Medicine and Public Health (M B Ahmed PhD), Department of Nursing and Health Sciences (S Shorofi PhD), Flinders University, Adelaide, SA, Australia; Faculty of Public Health (M B Ahmed PhD), Department of Information Science (W Jimma PhD), Jimma University, Jimma, Ethiopia; Department of Medicine (M Ahmed MBBS), Rawalpindi Medical University, Rawalpindi, Pakistan; Brody School of Medicine (S Ahmed PhD), Department of Political Science (A S Ogunrinde MPA), East Carolina University, Greenville, NC, USA; Institute of Global Health and Development (S Akhtar MPH), Aga Khan University, Karachi, Pakistan; Faculty of Health and Behavioural Sciences (W Akosile PhD), The University of Queensland, Brisbane, QLD, Australia (M Moni PhD); Faculty of Pharmacy (Y Al Thaher PhD), Philadelphia University, Amman, Jordan; School of Pharmacy (Y Al Thaher PhD), Division of Population Medicine (A Sha'aban PhD), Cardiff University, Cardiff, Wales; Department of Adult Health and Critical Care (O A M Al Zaabi PhD), College of Nursing (E Lazarus PhD, J K Muliira DSc), Sultan Qaboos University, Muscat, Oman; School of Public Health (M A M Al Zoubi PhD), McWilliams School of Biomedical Informatics (F Alahdab MD), Management Policy and Community Health (M Iqbal MPH), University of Texas, Houston, TX, USA; College of Engineering and Technology (T Al Zoubi PhD), American University of Ras Al Khaimah, Egaila, Kuwait; Department of Research and Development (Z Al-Aly MD), Washington University in St. Louis, St. Louis, MO, USA; Clinical Epidemiology Center (Z Al-Aly MD), US Department of Veterans Affairs (VA), St. Louis, MO, USA; Murdoch Business School (K Alam PhD), Murdoch University, Perth, WA, Australia; Preventive Dentistry Department (Prof M K Alam PhD), College of Nursing (Prof A F Alsharari PhD), Department of Clinical Laboratory Sciences (A Farhana PhD), Jouf University, Sakaka, Saudi Arabia; Department of Biology, College of Science and Humanities in Al-Kharj (Prof P Alam PhD), College of Nursing (M Zoromba PhD), Prince Sattam bin Abdulaziz University, Al-Kharj, Saudi Arabia; School of Health and Environmental Studies (Z Alam PhD, Prof S Hamidi DrPH), Hamdan Bin Mohammed Smart University, Dubai, United Arab Emirates; School of Nursing (R M Al-Amer PhD), Faculty of Nursing (H Khatatbeh PhD), Department of Basic Medical Sciences (Prof M M Khatatbeh PhD), Yarmouk University, Irbid, Jordan; School of Nursing and Midwifery (R M Al-Amer PhD), Western Sydney University, Sydney, NSW, Australia; Department of Health Information Management and Technology (Prof T M Alanzi PhD), Imam Abdulrahman Bin Faisal University, Dammam, Saudi Arabia (F M Alanezi PhD); Department of Clinical Pharmacy (F Y Al-Ashwal PhD), Al-Ayen Iraqi University, Thi-Qar, Iraq; Department of Clinical Pharmacy and Pharmacy Practice (F Y Al-Ashwal PhD), University of Science and Technology, Sana'a, Yemen; College of nursing and health sciences (H Albargi PhD), College of Nursing and Health Sciences (M J Almalki PhD, M Shanawaz MD), Clinical Nutrition Department (R M Chandika PhD), Health Research Center (Prof A Khalid PhD), Department of Prosthetic Dental Sciences (K A Mattoo MD), Department of Basic Medical Sciences (R Ravi DM), Department of Public Health (F Sobia PhD, J Varghese PhD), Jazan University, Jazan, Saudi Arabia; Department of Community and Mental Health (Prof M Albashtawy PhD), Physics Department, Faculty of Science (G N Makhadmeh PhD), Al al-Bayt

University, Mafraq, Jordan; Center for Health Systems Research (J E Alcalde-Rabanal PhD, E Serván-Mori PhD), Center for Nutrition and Health Research (I Campos-Nonato PhD), National Institute of Public Health, Cuernavaca, Mexico; Division of Pediatric Cardiology (K A Aldawsari MD), University of Colorado, Aurora, CO, USA; Heart Center (K A Aldawsari MD), Liver, Digestive, and Lifestyle Health Research Section and Organ Transplant Center of Excellence (S A Alqahtani MD), Innovation and Research (S Yezli PhD), King Faisal Specialist Hospital & Research Center, Riyadh, Saudi Arabia; General Directorate of Research and Studies (M S Aldossary MClinDent), Research & Innovation Center (Prof Z A Memish MD), Ministry of Health, Riyadh, Saudi Arabia; Institute of Health Informatics (R W Aldridge PhD), Department of Respiratory Medicine (M A Althobiani PhD), Institute of Global Health (B Angell PhD), Department of Population Health Sciences (D Sunkersing PhD), Department of Epidemiology and Public Health (V Tseriotis MSc), Center for Clinical Microbiology (Prof A Zumla PhD), University College London, London, England; School of Pharmacy (S M Aleidi PhD, Prof Y Bustanji PhD), Department of Diagnostic Radiology and Nuclear Medicine (Prof A Al-Ibraheem MD), The School of Medicine (M Al-Iede MD), The University of Jordan (Prof A M Hamdan-Mansour PhD), The University of Jordan, Amman, Jordan; Pediatric Intensive Care Unit (A Al-Eyadhy MD, Prof M Temsah MD), Department of Zoology (M K Oladejo MSc), King Saud University, Riyadh, Saudi Arabia; Department of Epidemiology and Biostatistics (A M Alfalki MPH), University of South Carolina, Columbia, SC, USA; Department of Bacteriology, Immunology, and Mycology (Prof A M Algammal PhD), Suez Canal University, Ismailia, Egypt; Faculty of Dentistry (A Alhumaidi DDS), Ibn Al-Nafis University for Medical Sciences, Sana'a, Yemen; Department of Oral and Maxillofacial Surgery (A Alhumaidi DDS), Xi'an Jiaotong University, Xi'an, China; Department of Statistics and Operations Research (I Ali PhD), Dept. of Periodontia & Community dentistry, Dr Ziauddin Ahmad Dental College (A Hashmi MDS), Aligarh Muslim University, Aligarh, India; College of Dental Medicine (Prof K Ali PhD), Department of Pharmaceutical Sciences (Prof K H Alzoubi PhD), College of Sport Sciences (A Khacharem PhD), Qatar University, Doha, Qatar; Department of Microbiology and Immunology (Prof L Ali PhD), Department of Biological Sciences (S B J Bacha PhD), National University of Medical Sciences (NUMS), Rawalpindi, Pakistan; Department of Pharmacy (M Ali PhD), Mohammed Al-Mana College for Medical Sciences, Dammam, Saudi Arabia (R A El Arab PhD); Department of Medical Rehabilitation (Physiotherapy) (M U Ali PhD), University of Maiduguri, Maiduguri, Nigeria; Nethersole School of Nursing (M U Ali PhD), Faculty of Medicine (J Huang MD), The Chinese University of Hong Kong, Hong Kong, China; Department of Biosciences (R Ali PhD), Centre for Interdisciplinary Research in Basic Sciences (Prof M I Hassan PhD, S Khan MSc), Centre For Interdisciplinary Research In Basic Sciences (CIRBSc) (T Mohammad PhD), Jamia Millia Islamia, New Delhi, India; Biodiversity and Genomic unit, University of Tabuk (S Ali PhD), Faculty of Nursing (W T Almagharbeh PhD), Applied College (A A Baig PhD), University of Tabuk, Tabuk, Saudi Arabia; University of Swat, Swat, Pakistan (S Ali PhD); Department of Nuclear Medicine (Prof A Al-Ibraheem MD), Cell Therapy and Applied Genomics Department (Prof M AlQudah MD), King Hussein Cancer Center, Amman, Jordan; Visiting Scholar (Collaborative Projects), Center of Excellence in Precision Medicine and Digital Health (Prof H Alinejad Rokny PhD), Chulalongkorn University, Bangkok, Thailand; Department of Health Care Management and Economics (C Alinia PhD), Food and Beverages Safety Research Center (M Aman Mohammadi PhD), School of Medicine (S Pourasghary MD), Urmia University of Medical Sciences, Urmia, Iran; Biomedical Physics Group (M Alipour BSc), University of Hamburg, Hamburg, Germany; Department of Clinical and Community Pharmacy (Prof S W Al-Jabi PhD, Prof S H Zyoud PhD), Department of Pharmacy (F Amer PhD), Department of Physiology, Pharmacology, and Toxicology (Prof R Shawahna PhD), Department of Chemistry (Prof A H Zyoud PhD), An-Najah National University, Nablus, Palestine; Family and Community

Medicine Department (M S Aljohani MD), Qassim University, Al Qassim, Saudi Arabia; Department of Public Health and Community Medicine (Prof S M Aljunid PhD, Prof C T Sreeramareddy MD), International Medical University, Kuala Lumpur, Malaysia; International Centre for Casemix and Clinical Coding (Prof S M Aljunid PhD), National University of Malaysia, Bandar Tun Razak, Malaysia; BRAC Institute of Governance and Development (BIGD) (M Al-Mamun MS), BRAC James P Grant School of Public Health (S Bente Kamal Tune MPH), School of Pharmacy (M Islam PhD), BRAC University, Dhaka, Bangladesh; Department of Public and Community Health (M Al-Mamun MS), Frontier University Garowe (FUG), Puntland, Somalia; Independent Consultant, Amman, Jordan (S Al-Marwani MSc); Department of Medicine (J U Almazan PhD), Nazarbayev University (N Arystan MPH), School of Medicine (Y Semenova PhD), Nazarbayev University, Astana, Kazakhstan; Department of Clinical Nursing (M M Alnaeem PhD), Al-Zaytoonah University of Jordan, Amman, Jordan; Nursing Faculty (K A Alnawafleh PhD), Fahad Bin Sultan University, Tabuk, Saudi Arabia; Department of Physical Therapy and Rehabilitation Sciences (Prof M A Alomari PhD), Department of Rehabilitation Sciences and Physical Therapy (Prof M A Alomari PhD), Department of Pathology and Microbiology (Prof M AlQudah MD), Faculty of Medicine (Prof M S I Alyahya PhD), Department of Public Health (Prof Y S Khader PhD, Prof K A Kheirallah PhD), Jordan University of Science and Technology, Irbid, Jordan; Division of Gastroenterology and Hepatology (S A Alqahtani MD), Weill Cornell Medicine, New York, NY, USA; Department of Nursing (I Alrimawi PhD), Georgetown University, Washington, DC, USA; Macro-Fiscal Policy Department (S M Alrousan PhD), Ministry of Finance, Dubai, United Arab Emirates; Department of Pharmacology (A S Al-Shami MS), Sana'a University, Sanaa, Yemen; Department of Basic Medical Sciences (A S Al-Shami MS), Universiti Malaysia Sarawak, Kota Samarahan, Malaysia; Research, Policy, and Training Directorate (A B Al-Tammemi MPH), Jordan Center for Disease Control, Amman, Jordan; Department of Respiratory Therapy (M A Althobiani PhD), Biological Sciences (Prof K R Hakeem PhD), Oral & Maxillofacial Rehabilitation Department (M Hamed DSc), Rabigh Faculty of Medicine (Prof A Malik PhD), King Abdulaziz University, Jeddah, Saudi Arabia; Research Group in Health Economics (Prof N Alvis-Guzman PhD), (University of Cartagena) (Universidad de Cartagena), Cartagena, Colombia; Research Group in Hospital Management and Health Policies (Prof N Alvis-Guzman PhD), (University of the Coast) (Universidad de la Costa), Barranquilla, Colombia; Fakeeh College for Medical Sciences (Prof Y M Al-Worafi PhD), Fakeeh College for Medical Sciences, Jeddah, Saudi Arabia; College of Medical Sciences (Prof Y M Al-Worafi PhD), Azal University for Human Development, Sana'a, Yemen; John D. Bower School of Population Health Science (M I Amedari MPH), University of Mississippi Medical Center, Jackson, MS, USA; Department of Preventive and Community Dentistry (M I Amedari MPH), Department of Community Health (S C Eneh MPH), Department of Biochemistry and Molecular Biology (M A Sanusi MSc), Obafemi Awolowo University, Ile-Ife, Nigeria; Biological Sciences Collegiate Division (Prof A Amin PhD), Department of Fixed Prosthodontics (M Hamed DSc), Department of Neurology (Prof A Hassan MD), Cairo University, Cairo, Egypt; Department of Health and Management Sciences (S Amini PhD), Department of Laboratory Sciences (K Ghaffari PhD), Laboratory Science Department (J Karami PhD), Khomein University of Medical Sciences, Khomein, Iran; Spiritual Health Research Centre (S Amiri PhD), Baqiyatallah University of Medical Sciences, Tehran, Iran; Department of Medicine (G A Amusa MD), Department of Pediatrics (A O D Ofakunrin MD), Faculty of Health Sciences, Department of Medicine and Jos University Teaching Hospital (G O Osaigbovo MD), University of Jos, Jos, Nigeria; Department of Internal Medicine (G A Amusa MD), Department of Pediatrics (A O D Ofakunrin MD), Jos University Teaching Hospital, Jos, Nigeria; Department of Sociology (Prof J Amzat PhD), Education (Prof K Berhanu PhD), Department of Education Leadership and Management (J O Okunlola PhD), University of

Johannesburg, Johannesburg, South Africa; Department of Health Care Management (P P Andrade MD), Technical University of Berlin, Berlin, Germany; European University, Lisbon, Portugal (P P Andrade MD); Department of Internal Medicine (S Ang MD), Rutgers University, Toms River, NJ, USA; Sarver Heart Center (S Ang MD), University of Arizona, Tucson, AZ, USA; Health System Science Program (B Angell PhD), The George Institute for Global Health, Newtown, NSW, Australia; Department of General Medicine (N Anh MD), Thai Binh University of Medicine and Pharmacy in Vietnam, Thai Binh City, Viet Nam; Ophthalmology Department (N Anh MD), Hoa Binh General Hospital, Hoa Binh City, Viet Nam; Department of Management (S E Ankomah PhD, S E Ankomah PhD), Department of Medical Laboratory Science (G Osei BSc), College of Health and Allied Sciences (D Simpong PhD), University of Cape Coast, Cape Coast, Ghana; Department of Public Health (K Annadurai PhD), The Apollo University, Chittoor, India; Department of Environmental and Occupational Health (B S Anuoluwa PhD), Department of Microbiology (I A Anuoluwa PhD), Mathematical and Computer Sciences (O Peter PhD), University of Medical Sciences, Ondo, Ondo, Nigeria; Regenerative Medicine, Organ Procurement and Transplantation Multi-disciplinary Center (S Anvari MD), Gastrointestinal and Liver Diseases Research Center (F Joukar PhD), Caspian Digestive Disease Research Center (F Joukar PhD), Nuroscience Research Center, Trauma Institute (H Sadr PhD), Guilan University of Medical Sciences, Rasht, Iran; Department of Surgery (S Anwar PhD), Faculty of Medicine, Public Health, and Nursing (FK-KMK) (B Dahal Khatri MPH), Department of Medical Surgical Nursing (A L Wicaksana PhD), Gadjah Mada University, Yogyakarta, Indonesia; College of Medicine (R Anwer PhD), Department of Biology (A A Chaudhary PhD), Department of Pharmacology (T Jawaid PhD), Imam Mohammad Ibn Saud Islamic University, Riyadh, Saudi Arabia; Rural Health Research Institute (A E Anyasodor PhD, S A I Mahmood PhD), Charles Sturt University, Orange, NSW, Australia; Health Management and Economics Research Center (J Arabloo PhD), School of Medicine (M Bastan MD), Department of Medical Laboratory Sciences (F Dorostkar PhD), Antimicrobial Resistance Research Center (K Mozahheb Yousefi MD), Hazrat-e Rasool General Hospital (K Mozahheb Yousefi MD), Rajaei Cardiovascular Research Center (M Naghshbandi MD), Physiology Research Center (H Pazoki Toroudi PhD), Department of Physiology (H Pazoki Toroudi PhD), Faculty of Advanced Technologies in Medicine (H Sadr PhD), Center for Technology and Innovation in Cardiovascular Informatics (S Shool MD), Iran University of Medical Sciences, Tehran, Iran; Al Ain University (Prof M Arafat PhD), Al Ain University, Abu Dhabi, United Arab Emirates; College of Art and Science (D Areda PhD), Ottawa University, Surprise, AZ, USA; Department of Veterinary Pharmacology and Toxicology (A Aremu PhD), University of Ilorin, Ilorin, Nigeria; Department of Biotechnology (Prof J Arockiaraj PhD, T Sundaram PhD), SRM Medical College Hospital and Research Centre (J James MD), Division of Medical Research (R Janardhanan PhD), SRM School of Public Health (J Samal MPH), Sri Ramaswamy Memorial Institute of Science and Technology, Kattankulathur, India; Institute of Social, Medical, and Applied Research Technology (A A Artamonov PhD), Institute of Social, Medical, and Applied Research Technology, Moscow, Russia; Department of Pharmacy Practice (Prof S Asdaq PhD), College of Medicine (M Fareed PhD), AlMaarefa University, Riyadh, Saudi Arabia; National Agency for Strategic Research in Medical Education (NASRME) (Prof S Asgary MSc), National Agency for Strategic Research in Medical Sciences Education (M Heydari PhD), Ministry of Health and Medical Education, Tehran, Iran; Health and Social Care (N Asiamah PhD), University of Essex, Colchester, England; School of Traditional Chinese Medicine (M Aslam PhD), Xiamen University Malaysia, Sepang, Malaysia; Researcher (G D Asres MPH), Amhara Public Health Institute, Bahir Dar, Ethiopia; Department of Medical-Surgical Nursing (A Assariparambil PhD), Kasturba Medical College, Manipal (S Koulmane Laxminarayana MD), Manipal Academy of Higher Education, Udupi, India; Atchabarov Scientific Research Institute of Fundamental and

Applied Medicine (B Assembekov PhD, A Zhumagaliuly MD), Scientific and Technological Park (I R Fakhradiyev PhD, A Ibrayeva PhD), Department of Public Health (A Kuttybayev MSc), Scientific Laboratory "Center for Collective Use" (A S Oradova PhD), Department of Clinical Pharmacology (Prof K D Rakhimov DSc), Science Department (A Shamsutdinova MD), Department of Medicine (S Tanabayeva PhD), Department of Science (A Y Tazhiyeva PhD), Department of Prosthetic Dentistry (M Tleshev MSc), Kazakh National Medical University, Almaty, Kazakhstan (S V Lee PhD); Harold Pupkewitz Graduate School of Business (Prof S O Atiku PhD), Namibia University of Science and Technology, Windhoek, Namibia; Department of Economics and Business Sciences (Prof S O Atiku PhD), Walter Sisulu University, Mthatha, South Africa; nursing (B Atinga PhD), University of Energy and Natural Resources, Sunyani, Ghana; School of Medicine and Public Health (P Atorkey PhD), University of Newcastle, Newcastle, NSW, Australia; Discipline of Psychological Science (P Atorkey PhD), ACAP University College, Sydney, NSW, Australia; Department of Forensic Medicine (A Atreya MD), Lumbini Medical College, Palpa, Nepal; Northumbria HealthCare NHS Foundation Trust, Newcastle upon Tyne, UK (A Aujayeb MBBS); Department of Oral Biological Science (N Aung MSc), University of Dental Medicine, Mandalay, Myanmar; Dental Unit (N Aung MSc), U Hla Tun's Hospice Cancer Foundation, Mandalay, Myanmar; Institute of Molecular Biology and Biotechnology (IMBB) (S J Awan PhD), The University of Lahore, lahore, Pakistan; Internal Medicine Department (K L Awol MD), St Paul's Hospital Millennium Medical College, Addis Ababa, Ethiopia; Bloomberg School of Public Health (K L Awol MD), Department of Public Health (B A Gelaw MD), School of Nursing (T K Hinneh MSc), Department of Neurosurgery (F Kazemi MD), Department of Psychiatry (E M Zeru MPH), Johns Hopkins University, Baltimore, MD, USA; Postgraduate Program in Pediatrics and Child Health (C O Ayala MSc), Doctoral Program in Biomedical Gerontology (R S Gutiérrez-Murillo PhD), Pontifical Catholic University of Rio Grande do Sul, Porto Alegre, Brazil; Department of Health Economics (M A Ayanore PhD), Centre for Health Policy Advocacy Innovation & Research in Africa (CHPAIR-Africa), Accra, Ghana; ASIDE Healthcare, Lewes, DE, USA (A Y Azzam MD); Faculty of Medicine (A Y Azzam MD), October 6 University, 6th of October City, Egypt; Community Medicine Department (Z S Babandi MD), Health Systems and Policy Research Unit (Prof S Mohammed PhD), Department of Paediatrics (S Musa MSc), Department of Community Medicine (A A Olorukooba MD, S S Umar FWACS), Ahmadu Bello University, Zaria, Nigeria; Department of Epidemiology (F Baberi PhD), Trauma Research Center (M Karajizadeh PhD), Department of Health Services Management (Prof R Ravangard PhD), Health Policy Research Center (Y Sarikhani PhD), Shiraz University of Medical Sciences, Shiraz, Iran; Physiology department (R Babiker PhD), RAK Medical and Health Sciences University, Ras Alkhaimah, United Arab Emirates; Department of Physiotherapy (A S Babu PhD, S Kumaran D PhD, M K Sinha PhD), Department of Forensic Medicine and Toxicology (S M Bakkannavar MD, Prof V C Nayak MD), Department of Pharmaceutical Regulatory Affairs and Management (S Dharmagadda PhD, V S Ligade PhD), Kasturba Medical College Mangalore (R Holla MD, M Rao MD), Prasanna School of Public Health (R Kamath MHA), Department of Community Medicine (C R Rao MD), Manipal Tata Medical College (G K Roy MPH), Department of Pharmacology (R R Shenoy PhD), Manipal College of Dental Sciences, Mangalore (Prof P K Shetty MDS), Department of Physiology (P A Shirali MD), Manipal Academy of Higher Education, Manipal, India; Department of Clinical Pathology (Prof A A Badran MD), Mansoura Faculty of Medicine, Mansoura, Egypt; Microbiology Department (Prof A A Badran MD), Horus University Egypt, Damietta, Egypt; Department of Neurological Surgery, Asan Medical Center (Y Bae MD), University of Ulsan, Seoul, South Korea; Department of Medicine (A Bahar MD), Wayne State University, Detroit, MI, USA; CRIMEDIM-Center for Research and Training in Disaster Medicine, Humanitarian Aid and Global Health (A A S Bahattab PhD), Università del

Piemonte Orientale, Novara, Italy; Department of Community Medicine and Public Health (A A S Bahattab PhD), University of Aden, Aden, Yemen; College of Optometry (R Bahreini MS), Pacific University, Forest Grove, OR, USA; Clinical Medical Research Center (Prof R Bai PhD), Nanjing Children's Hospital, Nanjing, China; School of Public Affairs (Prof R Bai PhD), Nanjing University of Science and Technology, Nanjing, China; Surgery, Global Surgery, Emergency Medicine Department (Prof L Bains MD), Maulana Azad Medical College, New Delhi, India; WHO Collaborating Centre for Research in Surgical Care Delivery in LMICs, Mumbai, India (Prof L Bains MD); Research and Scientific Division (A Bai-Sesay MPH), Ministry of Health, Free Town, Sierra Leone; Department of Medical Informatics (M Bakhshali PhD), Mashhad University of Medical Sciences, Mashhad, Iran; TIRR Memorial Hermann, Houston, TX, USA (A T Bako PhD); MAHER Centre for Global Health Research (P Balakrishnan PhD), Meenakshi Academy of Higher Education and Research (MAHER), Chennai, India; Royal College of Medicine Perak (P Balakrishnan PhD), Universiti Kuala Lumpur, Ipoh, Malaysia; Division of Biological Sciences (S Balakrishnan PhD), Tamil Nadu State Council for Science and Technology, Chennai, India; Center of Innovation, Technology, and Education (CITE) (Prof O Baltatu PhD), Anhembi Morumbi University, São José dos Campos, Brazil; Institute of Health and Wellbeing (B Banik PhD), Federation University Australia, Melbourne, VIC, Australia; Manna Institute (B Banik PhD), University of New England, Armidale, NSW, Australia; Clinic for Infectious and Tropical Diseases (A Barac PhD), Clinical Center of Serbia, Belgrade, Serbia; Faculty of Medicine (A Barac PhD, I M Ilic PhD, Prof M M Santric-Milicevic PhD), School of Public Health and Health Management (Prof M M Santric-Milicevic PhD), University of Belgrade, Belgrade, Serbia; Heidelberg Institute of Global Health (HIGH) (Prof T W Bärnighausen MD, Prof S Mohammed PhD), Heidelberg University, Heidelberg, Germany; T.H. Chan School of Public Health (Prof T W Bärnighausen MD), Department of Global Health and Population (A D Gage MSc), Harvard Medical School (A Zhong MA), Harvard University, Boston, MA, USA; School of Health Professions (Prof C A Barth PhD), Bern University of Applied Sciences, 3008 Bern, Switzerland; Alpha Genomics Private Limited, Islamabad, Pakistan (Z Basharat PhD); Faculty of Nursing (Prof A Batiha PhD), King Khalid University, Mahyil Asir, Saudi Arabia; Department of Medical Education (K Batra PhD), Department of Social and Behavioral Health (Prof M Sharma PhD), University of Nevada Las Vegas, Las Vegas, NV, USA; Department of Human Anatomy and Histology (Prof N M Beeraka PhD), I.M. Sechenov First Moscow State Medical University, Moscow, Russia; Transplant and Hepatobiliary Surgery Service (D F Bejarano Ramirez MSc), Hospital Universitario Fundación Santa Fe de Bogotá (University Hospital Santa Fe Foundation of Bogotá), Bogota, Colombia; Subdirectorate of Clinical Studies and Clinical Epidemiology (D F Bejarano Ramirez MSc), Hospital Universitorio Fundación Santa Fe de Bogotá, Bogotá, Colombia; Department of Nursing (B M Belay MSc), Department of Public Health (A T Desale MPH), Midwifery Department (Y A Goshu MSc), Debre Tabor University, Debre Tabor, Ethiopia; Department of Medicine (Prof A K Bello PhD, E Lytvyak MD), Department of Psychiatry (E Eboreime PhD), University of Alberta, Edmonton, AB, Canada; Institute of Behavioral Sciences (Prof K Berhanu PhD), College of Medicine and Health Science (A Edmealem MSc), Debre Markos University, Debre Markos, Ethiopia; Hubert Department of Global Health (R S Bernstein MD), Emory University, Atlanta, GA, USA; Department of Global Health (R S Bernstein MD), Department of Neurosurgery (A H Feroze MD), George Washington University, Washington, DC, USA; Department of Anatomy (N Bhardwaj MD), All India Institute of Medical Sciences, Jodhpur, India; National Institute for Implementation Research on Non Communicable Diseases (Prof P Bhardwaj MD), Indian Council of Medical Research, Jodhpur, India; Global Health Neurology Lab (S Bhaskar MD), NSW Brain Clot Bank, Sydney, NSW, Australia; Division of Cerebrovascular Medicine and Neurology (S Bhaskar MD), National Cerebral and Cardiovascular Center,

Suita, Japan; Translational and Clinical Research Institute (P Bhattacharjee MD), Newcastle University, Newcastle upon Tyne, England; University Centre for Research and Development (Prof G K Bhatti PhD, S Kalra DM), University Centre for Research and Development (UCRD) (B Singh PhD), Chandigarh University, Mohali, India; Department of Human Genetics and Molecular Medicine (Prof J S Bhatti PhD), Department of Biochemistry (B Singh PhD), Department of Zoology (Prof B Vellingiri PhD), Central University of Punjab, Bathinda, India; Department of Pharmaceutical Sciences (Prof R Bhatti PhD), Guru Nanak Dev University, Amritsar, India; Centre for Global Child Health (Prof Z A Bhutta PhD), University of Toronto, Toronto, ON, Canada; Institute for Global Health & Development (Prof Z A Bhutta PhD), Aga Khan University, Karachi, Pakistan; Independent Consultant, Addis Ababa, Ethiopia (S K Biadgilign PhD); Department of Community and Family Medicine (B Biswas MD), Department of Physiology (H Mondal MD), All India Institute of Medical Sciences, Deoghar, India; Department of Biochemistry and Biotechnology (M Biswas PhD), University of Science and Technology Chittagong, Chittagong, Bangladesh; Department of Public Health (M Biswas PhD, O J Okesanya MPH, M Shimul MPH), Department of Development Studies (M Sohel MPH), Daffodil International University, Dhaka, Bangladesh; Department of Public Health (F Bizuneh MPH), Woldia University, Woldia, Ethiopia; SAMRC/Wits Centre for Health Economics and Decision Science - PRICELESS SA (M K Boachie PhD), Wits Health Consortium, Johannesburg, South Africa; Department of Community and Family Medicine (Prof T Bodhare MD), All India Institute of Medical Sciences, Ramanathapuram, India; Department of Veterinary Medicine (S Bohlouli PhD), Islamic Azad University, Kermanshah, Iran; Faculty of Sport - Research Centre for Physical Activity, Health, and Leisure (CIAFEL) (L Bohn PhD), Research Unit on Applied Molecular Biosciences (UCIBIO) (Prof F Carvalho PhD), Institute for Research and Innovation in Health (i3S) (Prof N Cruz-Martins PhD), MEDCIDS, Faculty of Medicine of the University of Porto (A Freitas PhD), University of Porto, Porto, Portugal; College of Medecine and Health Sciences (Prof A Bonny MD), College of Medicine and Health Sciences (Prof C M Muvunyi PhD), University of Rwanda, Kigali, Rwanda; Department of Cardiology (Prof A Bonny MD), (Centre Hospitalier Montfermeil) (Montfermeil Hospital Center), Montfermeil, France; Newcastle Business School, College of Human and Social Futures (S Bose PhD), University of Newcastle, Sydney, NSW, Australia; Department of Medicine (Prof S Bouaoud DrPH), Faculty of Medicine (Prof A Ouyahia PhD), University Ferhat Abbas of Setif, Setif, Algeria; Department of Epidemiology and Preventive Medicine (Prof S Bouaoud DrPH), University Hospital Saadna Abdenour, Setif, Algeria; Department of Epidemiology (D Braithwaite PhD, D D Ding BS), College of Medicine (K T Root BS), University of Florida, Gainesville, FL, USA; Cancer Population Sciences Program (D Braithwaite PhD), University of Florida Health Cancer Center, Gainesville, FL, USA; Department of Paediatrics and Child Health (D C Briggs FWACP), Rivers State University, Nkpolu-Oroworukwo in Nigeria, Port Harcourt, Nigeria; Department of Paediatrics and Child Health (D C Briggs FWACP), Rivers State University Teaching Hospital, Port Harcourt, Nigeria; Department of Management (O Bunea PhD), Faculty of Management (Prof A Dima PhD), Department of Statistics and Econometrics (A Mirica PhD, A Otoiu PhD), Management Department (Prof I Popa PhD, Prof S Ștefan PhD), Bucharest University of Economic Studies, Bucharest, Romania; Department of Radiology (F Busch MD), Department of Public Health and Primary Care (M Dalakoti MPH), Department of Medical Genetics (E Waisberg MD), University of Cambridge, Cambridge, England; Department of Health Care Management (Prof R Busse PhD), Technische Universität Berlin (Technical University of Berlin), Berlin, Germany; School of Public Health Sciences (Z A Butt PhD), University of Waterloo, Waterloo, ON, Canada; Al Shifa School of Public Health (Z A Butt PhD), Al Shifa Trust Eye Hospital, Rawalpindi, Pakistan; Department of Anesthesiology (S Cao MD), Third Xiangya Hospital of Central South University, Changsha, China; Faculty of Dentistry (Y Cao

BSc), Department of Medicine (B Chong MBBS), Cardiovascular Metabolic Translational Research Program (M Dalakoti MPH), Department of Surgery (K Tan PhD), Yong Loo Lin School of Medicine (Prof N Venketasubramanian MSc), National University of Singapore, Singapore, Singapore; Centre For Data Driven Policy and Management (M Car PhD), Catholic University of Croatia, Zagreb, Croatia; Department of Primary Care and Public Health (M Car PhD, Prof S Rawaf MD), Department of Surgery and Cancer (Prof E Mossialos PhD), WHO Collaborating Centre for Public Health Education and Training (D L Rawaf MD), The George Institute for Global Health (Prof S Yaya PhD), Imperial College London, London, England (Prof L Tudor Car PhD); Department of Health Care (Prof R Cárdenas DSc), Metropolitan Autonomous University, Mexico City, Mexico; IMPInstitute for Mental and Physical Health and Clinical Translation (IMPACT) (A F Carvalho MD), Deakin University, Geelong, VIC, Australia; Department of Pharmacological and Biomolecular Sciences (Prof A L Catapano PhD), Department of Food, Environmental and Nutritional Sciences (Prof S Perna PhD), University of Milan, Milan, Italy; MultiMedica Sesto San Giovanni IRCCS, Sesto San Giovanni, Italy (Prof A L Catapano PhD); Posgrado de Medicina (Postgraduate Program in Medicine) (P Chacón-Uscamaita DDS), Universidad Cientifica del Sur, Lima, Peru; Department of Psychiatry (Prof M Chandradasa MD), Department of Pharmacology (Prof C D K Mettananda PhD), Department of Paediatrics (Prof S Mettananda DPhil), University of Kelaniya, Ragama, Sri Lanka; University Psychiatry Unit (Prof M Chandradasa MD), Clinical Medicine Department (Prof C D K Mettananda PhD), University Paediatrics Unit (Prof S Mettananda DPhil), Colombo North Teaching Hospital, Ragama, Sri Lanka; Department of Public Health Dentistry (V Chandu MDS), Government Dental College & Hospital, Vijayawada, Vijayawada, India; College of Medicine (J Chang PhD), National Taiwan University, Taipei, Taiwan; Department of Nursing (J Chang PhD), National Taiwan University Hospital, Taipei, Taiwan; Cardiology Department (Q Chang MD), the First Affiliated Hospital of Jinzhou Medical University, Jinzhou, China; Department of Public Health and Health Sciences (Prof V Chattu PhD), Tennessee State University (TSU), Nashville, TN, USA; Department of Community Medicine (Prof V Chattu PhD), Datta Meghe Institute of Medical Sciences, Sawangi, India; Department of Epidemiology (L D Chau BA), The University of Texas Health Science Center at Houston (UTHealth Houston), Houston, TX, USA; ICMR National Institute of Health Research, Dibrugarh (S Chaudhuri MD), Indian Council of Medical Research, Dibrugarh, India; Department of Oral Medicine and Radiology (Prof A Chaurasia MD), Hospital Administration (M Suhaib MHA), King George's Medical University, Lucknow, India; Department of Anatomy and Physiology, Faculty of Medicine (C Che Mohd Nassir PhD), Universiti Sultan Zainal Abidin, Kuala Terengganu, Malaysia; Clinical Research Center (H Chen PhD), Zhujiang Hospital of Southern Medical University, Guangzhou, China; Department of Endocrine and Metabolic Diseases (M Chen PhD), Shanghai Mental Health Center (Prof M R Phillips MD), School of global health (J Zheng PhD), Shanghai Jiao Tong University, Shanghai, China; Red Cross College of Nursing (Prof Y Choi PhD), Chung-Ang University, Seoul, South Korea, Seoul, South Korea; Centre for Research Impact & Outcome (H Chopra PhD), Chitkara University, Rajpura, India; Department of Biosciences (S Chopra MPH), Saveetha Medical College and Hospital (Prof M Karobari PhD), Saveetha Dental College and Hospitals (G Minervini PhD, Prof D Sekar PhD, M Tovani-Palone PhD), Department of Biochemistry (S Mohan PhD), Department of Biomaterials (N Rabiee PhD), Department of Paediatric and Neonatal Sciences, Saveetha College of Physiotherapy, Saveetha Institute of Medical and Technical Sciences (J Thirumalai MPT), Department of Neonatology (R Thiruvengadam PhD), Saveetha University, Chennai, India; Department of Community Medicine (Prof S G Choudhari MD), Jawaharlal Nehru Medical College, Wardha, India; The Interdisciplinary Research Group on Biomedicine and Health (D Chu PhD), Faculty of Applied Sciences (D Chu PhD), VNU International School (VNUIS), Hanoi, Viet Nam; Department of Public

Health (Prof J T Chua PhD), Other, Tuguegarao City, Philippines; Department of Paediatric Surgery (I S Chukwu BMedSc), Federal Medical Centre, Umuahia, Nigeria; Department of Health Behavior (S Chung MPH), Texas A&M University, College Station, TX, USA; Hypertension and Cardiovascular Risk Research Group (Prof A F G Cicero PhD), Hypertension and Cardiovascular Risk Research Center (F Fogacci MDc), Department of Biomedical and Neuromotor Sciences (S Guicciardi MD), Department of Medicine and Surgery (I Papadimopoulos MD), Department of Medical and Surgical Sciences (M Sassano MD), University of Bologna, Bologna, Italy; Heart-Chest-Vascular Department (Prof A F G Cicero PhD), IRCCS Azianda Ospedaliero-Universitaria di Boloogna, Bologna, Italy; The David S. and Ruth L. Gottesman Center for Headache Treatment and Translational Research (F Cohen MD), Department of Medicine (F Cohen MD), Icahn School of Medicine at Mount Sinai, New York, NY, USA; Nova Medical School (Prof J Conde PhD), Nova University of Lisbon, Lisbon, Portugal; Department of Health Sciences (C Cosma MD), University of Florence, Florence, Italy; Life and Health Sciences Research Institute (ICVS) (Prof N Cruz-Martins PhD), University of Minho, Braga, Portugal; Center for Immigrant and Refugee Health (O Dadras PhD), Public Health Institute, Los Angeles, CA, USA; Department of Medical and Surgical Sciences (Prof E D'Amico MD, A Zanghì MD), University of Foggia, Foggia, Italy; Institute for Global Health Innovations (A K Dang MD), Duy Tan University, Hanoi, Viet Nam; Department of Public Health (S D Darcho MPH), Haramaya University, Harar, Ethiopia; Department of Environmental Health (R Darvishi Cheshmeh Soltani PhD), Department of Nursing (A Jadidi PhD), Department of Pediatrics (J Nazari MD), Arak University of Medical Sciences, Arak, Iran; Department of Health Policy & Health Services Research (Prof M N G de Abreu PhD), Department of Orthodontics (S Shirazi DDS), Boston University, Boston, MA, USA; School of Public Health (Prof J De Neve MD), San Diego State University, San Diego, CA, USA; School of Medicine (I Delgado-Enciso DSc), University of Colima, Colima, Mexico; Department of Research (I Delgado-Enciso DSc), State Cancerology Institute of Colima, IMSS-BIENESTAR, Colima, Mexico; Department of Biological Sciences (I Dergaa PhD), University of Manouba, Manouba, Tunisia; Department of Social Sciences (I Dergaa PhD), University of Jendouba, El Kef, Tunisia; St Paul's Eye Unit (N Dervenis MD), Royal Liverpool University Hospital, Liverpool, UK; Clinic for Pulmonary Diseases and Tuberculosis “Podhrastovi” (M Dervišević PhD), University Clinical Center in Sarajevo, Sarajevo, Bosnia and Herzegovina; Chettinad Hospital & Research Institute (Prof V Devanbu MD), Chettinad Academy of Research and Education, Chennai, India; Department of Pharmacy (S Dewan PhD), United International University, Dhaka, Bangladesh; Pharmacology Division (S Dewan PhD), Center for Life Sciences Research Bangladesh, Dhaka, Bangladesh; Yeungnam University (T Dhakal PhD), Yeungnam University, Daegu, South Korea; Neurology Department Institute of Human Behavior and Allied Sciences (Prof R K Dhamija MD), University of Delhi, New Delhi, India; Research and Development Cell (A S Dhane MBA), Department of Oral Pathology and Microbiology (Prof G S Sarode PhD, Prof S C Sarode PhD), Dr. D. Y. Patil Vidyapeeth, Pune (Deemed to be University), Pune, India; Department of Life Science and Public Health (M Di Pumpo DrPH), Università Cattolica del Sacro Cuore (Catholic University of the Sacred Heart), Rome, Italy; College of Health Sciences (H P Do PhD, T T Pham PhD), College of Health Sciences (CHS) (Prof D Poddighe PhD), VinUniversity, Hanoi, Viet Nam; Institute of Health Economics and Technology (iHEAT), Hanoi, Viet Nam (H P Do PhD); International Institute for Training and Research (INSTAR) (M N Do MD), Vietnam National University, Hanoi, Viet Nam; Department of Medical, Surgical, and Health Sciences (Prof M D'Oria MD), University of Trieste, Trieste, Italy; Cardio-Thoraco-Vascular Department (Prof M D'Oria MD), Azienda Sanitaria Universitaria Giuliano Isontina, Trieste, Italy; Independent Consultant, Bridgewater, NJ, USA (O P Doshi MS); School of Sociology (E W Dumbili PhD), University College Dublin, Dublin, Ireland; Faculty of Science and Humanities (S Duraisamy PhD), SRM

Institute of Science and Technology, Kattankulathur, India; Department of Infection and Tropical Medicine (O C Durojaiye MPH), University of Sheffield, Sheffield, England; Health Research Institute (A Dushpanova PhD, M Kulimbet MSc), Department of Clinical Subjects (A Kurmanova MD), Al Farabi Kazakh National University, Almaty, Kazakhstan; Department of Psychiatry (E Eboreime PhD), Dalhousie University, Halifax, NS, Canada; Department of Rehabilitation Science & Technology, School of Health and Rehabilitation Sciences (I D Ebuenyi PhD), University of Pittsburgh, Pittsburgh, PA, USA; School of Nursing and Midwifery (Prof D Edvardsson PhD), La Trobe University, Melbourne, VIC, Australia (Prof F Efendi PhD); Advanced Nursing Department (Prof F Efendi PhD), Department of Public Health (F S Rahman PhD), Universitas Airlangga (Airlangga University), Surabaya, Indonesia; Antimicrobial Resistance - Department of Planning, Research and Statistics, NCDC (A Egwuenu MPH), Independent Consultant, Abuja, Nigeria; Faculty of Science and Health (M Ekholuenetale PhD), University of Portsmouth, Hampshire, UK; Department of Anatomy and Embryology (M A Eladl PhD), Faculty of Pharmacy (Prof M A Saleh PhD), Faculty of Nursing (M Zoromba PhD), Mansoura University, Mansoura, Egypt; College of Medicine (M Elhadi MD), Department of Medicine (I R Fakhradiyev PhD), Department of Biomedical Informatics (Prof S Jeong PhD), School of Health and Environmental Science (Prof J Kang PhD), Department of Health Policy and Management (Prof J Kim PhD), Department of Preventive Medicine (Prof Y Lee PhD), Korea University, Seoul, South Korea; Institute of Public Health (Y A M Elhadi PhD), United Arab Emirates University, Al Ain, United Arab Emirates; Department of Healthcare Policy and Financing (Y A M Elhadi PhD), Global Health Focus, Khartoum, Sudan; Deanery of Biomedical Sciences (R Elsheikh MD), School of Health in Social Science (Y Hong BSc), Usher Institute (Prof C R Simpson PhD), University of Edinburgh, Edinburgh, Scotland; Department of Pediatrics (C Eltaha MD), University of Texas, Dallas, TX, USA; Interdisciplinary Health Sciences (S C Eneh MPH), University of Texas at El Paso, Texas, TX, USA; Department of Bacteriology and Virology (M Eslami PhD), Semnan University of Medical Sciences, Semnan, Iran; Cancer Research Center (M Eslami PhD), Semnan University of Medical Scinces, Semnan, Iran; Department of Public Health Epidemiology (F Eva MPH), Florida International University, Miami, FL, USA; Drexel Dornsife School of Public Health (E Ezenwankwo MPH), College of Medicine (C Garg MD), Drexel University, Philadelphia, PA, USA; Department of Anesthesia (A O Fadaka PhD), Cincinnati Children's Hospital Medical Center, Cincinnati, OH, USA; Department of Biotechnology (A O Fadaka PhD), School of Pharmacy (O C Okonji MSc), University of the Western Cape, Cape Town, South Africa; Department of Electrical and Computer Engineering (H Fadavian MSc), Department of Electrical and Computer Engineering (ECE) (Prof D Fathi PhD), Department of Hematology (B Razi PhD), Tarbiat Modares University, Tehran, Iran; Research Centre for Healthcare and Community (A F Fagbamigbe PhD), Coventry University, Coventry, UK; Tanner College of Dental Medicine (Prof A Fahim PhD), University of Pikeville, Pikeville, KY, USA; School of Human and Social Sciences (FCHS) (L Faria PhD), University of Algarve, Faro, Portugal; University Research Center in Psychology, Faro, Portugal (L Faria PhD); Department of Psychology (A Faro PhD), Federal University of Sergipe, São Cristóvão, Brazil; Department of Agriculture and Animal Health (Prof F O Fasina PhD), University of South Africa, Roodepoort, South Africa; Department of Infectious Diseases and Public Health (G Fekadu PhD, Prof W Ming MD), City University of Hong Kong, Hong Kong, China; Department of Pharmacy (G Fekadu PhD), Wollega University, Nekemte, Ethiopia; Department of Neurosurgery (A H Feroze MD), Children's National Medical Center, Washington, DC, USA; Center for Public Health Research (P Ferrara PhD), University of Milan Bicocca, Monza, Italy; Laboratory of Public Health (P Ferrara PhD), IRCCS Istituto Auxologico Italiano, Milan, Italy; European Network of Occupational Therapy in Higher Education, Vienna, Austria (M Figueiredo Professional Doctorate); Faculty of Rehabilitation and Allied Health

Sciences (S Firdous PhD), Riphah International University, Lahore, Pakistan; Institute of Public Health (F Fischer PhD), Charité Universitätsmedizin Berlin (Charité Medical University Berlin), Berlin, Germany; Department of Medical Pharmacology (F Fogacci MDc), Ataturk University, Erzurum, Türkiye; Department of Cell Biology and Biotechnology (A A Fomenkov PhD), K.A. Timiryazev Institute of Plant Physiology, Moscow, Russia; UO Neurologia, Salute Pubblica e Disabilità (The Neurology, Public Health and Disability Unit) (A Fornari PhD), Fondazione IRCCS Istituto Neurologico Carlo Besta (IRCCS Foundation Carlo Besta Neurological Institute), Milan, Italy; Department of Community Medicine (A Forray PhD), "Iuliu Hațieganu" University of Medicine and Pharmacy, Cluj-Napoca, Romania; Center for Health Technology and Services Research (CINTESIS), Porto, Portugal (A Freitas PhD); Department of Dermatology (Prof T Fukumoto PhD), Kyoto Prefectural University of Medicine, Kyoto, Japan; Gates Foundation, Seattle, WA, USA (N Fullman MPH); Department of Healthcare Management (Prof A Fusheini PhD), University of Doha for Science and Technology, Doha, Qatar; Department of Preventive and Social Medicine (Prof A Fusheini PhD), Department of Public Health (G Otchere MSc), University of Otago, Dunedin, New Zealand; Health Services Management Training Centre (Prof P A Gaal PhD, J Lám PhD, T Palicz PhD), Institute of Digital Health Sciences (T Palicz PhD), Semmelweis University, Budapest, Hungary; Department of Applied Social Sciences (Prof P A Gaal PhD), Sapientia Hungarian University of Transylvania, Târgu-Mureş, Romania; Department of Community Medicine (Prof M A Gadanya MD), Aminu Kano Teaching Hospital, Kano, Nigeria; School of Public Health (D Gadeka PhD), University of Ghana, Legon-Accra, Ghana; Department of Public Health (M Gajdács PhD), University of Szeged, Szeged, Hungary; Laboratory Sciences Division (B Ganesh PhD), National Institute of Epidemiology, Chennai, India; Trauma and Emergency Medicine (E Ganessane MD), All India Institute of Medical Sciences, Madurai, India; Department of Health Policy and Administration (Prof F B Garcia PhD), National Institutes of Health (A Loreche MS), Department of Health Policy and Administration, College of Public Health (T T Moncatar PhD), University of the Philippines Manila, Manila, Philippines; Internal Medicine Department (C Garg MD), University of Pittsburgh Medical Center, Harrisburg, PA, USA; Infectious Diseases Unit (J Garlasco MD), University of Verona, Verona, Italy; Amity Institute of Pharmacy (Prof R K Gautam PhD), Amity University Uttar Pradesh, Noida, India; Cochlear Center for Hearing and Public Health (T S Gebre MD), Johns Hopkins University, Philadelphia, PA, USA; Director General Office (B A Gelaw MD), Ethiopian Public Health Institute, Addis Ababa, Ethiopia; Department of Neurosciences, Neurology and Stroke Unit (S Gelibter MD), ASST Grande Ospedale Metropolitano Niguarda, Milan, Italy; Department of Neurosurgery (J L Gendreau MD), Oregon Health and Science University, Portland, OR, USA; Harold Pupkewitz Graduate School of Business (K I Genty PhD), Polytechnic of Namibia, Windhoek, Namibia; Department of Industrial Relations and Human Resource Management (K I Genty PhD), Lagos State University, Lagos, Nigeria; School of Medical and Health Science (G K Getahun MPH), Edith Cowan University, Australia, Perth, WA, Australia; Department of Applied Health Sciences (L Ghalichi PhD), College of Medicine and Health (J Muhammad PhD), Institute of Applied Health Research (R Thayakaran PhD), University of Birmingham, Birmingham, England; Research Committee of Qom University of Medical Sciences (A Ghamkhar BSc), Qom University of Medical Sciences, Qom, Iran; Department of Medical Biochemical Analysis (A Ghandili PhD), Department of Business Administrations (Prof D H Kadir PhD), Department of Nutrition and Dietetics (B A Sadee PhD), Cihan University-Erbil, Erbil, Iraq; School of Public Health (A Ghashghaee BSc), Qazvin University of Medical Sciences, Qazvin, Iran; Research Department (S Ghimire MPH, U Paudel PhD), Nepal Health Research Council, Kathmandu, Nepal; Department of Radiology (A Gholamrezanezhad MD), University of Southern California, Los Angeles, CA, USA; School of Social Sciences (Prof V Giannouli PhD), Hellenic Open University, Patras, Greece;

Department of Social and Behavioral Sciences (Prof V Giannouli PhD), European University Cyprus, Nicosia, Cyprus; Country Office (A U Gil PhD), World Health Organization (WHO), Astana, Kazakhstan; Department of Biological Sciences and Chemistry (DBSC) (S A Gilani PhD, Prof J Hussain PhD), Natural and Medical Sciences Research Center (A Khan PhD, A Ullah MS), University of Nizwa, Nizwa, Oman; Department of Zoology (B R Giri PhD), KKS Women's College, Balasore, India; Department of Nursing (A A Girmay MSc), Aksum University, Aksum, Ethiopia; Department of Health Systems and Policy Research (Prof M Golechha PhD), Indian Institute of Public Health, Gandhinagar, India; Department of Genetics (P Goleij MSc), Sana Institute of Higher Education, Sari, Iran; Universal Scientific Education and Research Network (USERN) (P Goleij MSc), Substance Abuse Prevention Research Center (Y Sayadi PhD), Kermanshah University of Medical Sciences, Kermanshah, Iran; Department of Life Sciences, Health and Healthcare Professions (Prof D Golinelli MD), Link Campus University, Rome, Italy; Research Institute for Endocrine Sciences, Tehran, Iran (M Golmohammadi MD); Postgraduate Program in Epidemiology (Prof B N G Goulart DSc), Department of Psychiatry and Legal Medicine (Prof G A Salum PhD), Federal University of Rio Grande do Sul, Porto Alegre, Brazil; Department of Pathology (T Govardhan MD), PHYSIOLOGY (Prof R Raja Reddy MD), Apollo Institute of Medical Sciences and Research, Chittoor, India; Department of Dermatology (A Grada MD), University Hospitals (O A Oyebanji MD), Case Western Reserve University, Cleveland, OH, USA; Medical Directorate (G Guarducci MD), Local Health Authority of Ferrara, Ferrara, Italy; Department of the Health Directorate (S Guicciardi MD), Local Health Authority of Bologna, Bologna, Italy; Department of Pediatrics (Prof S Gulati MD), Centre for Community Medicine (P Halder MD), Department of Biophysics (T Mohammad PhD), Centre for Dental Education and Research (H Priya MDS), Department of Psychiatry (Prof R Sagar MD), Department of Radiation Oncology (V Sahni MDS), Medical Oncology Lab (M Singh PhD), Department of Physiology (V K Tiwari MD), All India Institute of Medical Sciences, New Delhi, India; School of Global Health and Bioethics (Prof K A Gulma PhD), Euclid University, Banjul, The Gambia; Department of Community Medicine (Prof D A Gunawardane MD, Prof S N K Navaratna MD), University of Peradeniya, Kandy, Sri Lanka; Global Health Research Center (L Guo MSc, Prof S Tang PhD), Duke Kunshan University, Kunshan, China; Department of Dermatology (A Gupta MD), Dr. D.Y. Patil Medical College, Hospital and Research Centre, Pune, India; Department of Public Health (B Gupta PhD), Torrens University Australia, Melbourne, VIC, Australia; Department of Preventive Cardiology & Medicine (Prof R Gupta MD), Eternal Heart Care Centre & Research Institute, Jaipur, India; Department of Medicine (Prof R Gupta MD), Mahatma Gandhi University Medical Sciences, Jaipur, India; Department of Toxicology (S Gupta PhD), Shriram Institute for Industrial Research, Delhi, India; School of Biotechnology (V Gupta PhD), Dublin City University, Dublin, Ireland; Research Unit in Epidemiology Clinic (J Guzman-Esquivel DSc), Mexican Institute of Social Segurity, Colima, Mexico; Global Virus Network, Middle East Region, Shiraz, Iran (F Habibzadeh MD); Euclid University, Greater Banjul Area, The Gambia (A Hakuzimana PhD); Department of Community Medicine (P Halder MD), Department of Pharmacology (A K Kakkar MD), Post Graduate Institute of Medical Education and Research, Chandigarh, India; Department of Chemistry, College of Science (Prof K H Hama Aziz PhD), Department of Biology (F M Rahman PhD), University of Sulaimani, Sulaymaniyah, Iraq (H H R Najmuldeen PhD); Department of Cardiology (P Hamian Roumiani MD), Azerbaijan Medical University, Baku, Azerbaijan; Sakarya University, Sakarya, Türkiye (A Hanif PhD); Research Unit (J M Haro MD), Parc Sanitari Sant Joan de Deu, Barcelona, Spain; Department of Mental Health (J M Haro MD), Carlos III Health Institute (Prof R Tabarés-Seisdedos PhD), Biomedical Research Networking Center for Mental Health Network (CiberSAM), Madrid, Spain; Department of Public Health (M Hasan MPH), Tropical Disease and Health Research Center, Dhaka, Bangladesh; Department of Biomedical

Engineering and Public Health (S Hasan PhD), World University of Bangladesh, Dhaka, Bangladesh; Department of Food Technology and Nutrition Science (T Hasan PhD), Noakhali Science and Technology University, Noakhali, Bangladesh; Department of Medical Surgical (Prof A Hasanpour- Dehkordi PhD), Shahroud University of Medical Sciences, Shahrekord, Iran; Institute of Radiology and Radiological Sciences (A Hashem Zadeh MD), Tehran University of Medical Sciences, tehran, Iran; Department of Surgery (A Hashem Zadeh MD), University of California San Francisco, Oakland, MD, USA; Department of Pharmaceutical Technology (Prof M S Hasnain PhD), Adamas University, Kolkata, India; Department of Community Medicine (I Hassan PhD), Federal University Teaching Hospital, Lafia, Nigeria; Department of Epidemiology and Community Medicine (I Hassan PhD), Federal University of Lafia, Lafia, Nigeria; Aberdeen Royal Infirmary (A Hayatu MBBS), NHS National Services Scotland, Aberdeen, Scotland; Department of Internal Medicine (A Hayatu MBBS), University of Edinburgh, Scotland, Scotland; Graduate School of Medicine (Y Hiraike PhD), University of Tokyo, Tokyo, Japan; School of Computer Science (Prof M Hoseinzadeh PhD), Institute of Research and Development (P Khoshvaght BSc, A Omar Bali PhD), Institute for Global Health Innovations (C T Nguyen MPH, H T Nguyen MD, H L T Nguyen MPH), Faculty of Medicine (T Nguyen PhD), Duy Tan University, Da Nang, Viet Nam; International Center for Materials Sciences and Technology (Prof M Hoseinzadeh PhD), Research Center of High Technologies and Innovative Engineering (P Khoshvaght BSc), Western Caspian University, Baku, Azerbaijan; Maternal and Child Health Division (MCHD) (L Hossain MPH), International Centre for Diarrhoeal Disease Research, Bangladesh, Dhaka, Bangladesh; Department of Decision and Information Sciences (M Hossain DrPH), Department of Biology and Biochemistry (S Ullah MSc), University of Houston, Houston, TX, USA; Public Health Research Group (M Hossain DrPH), Nature Study Society of Bangladesh, Khulna, Bangladesh; Department of Population Sciences (Prof M B Hossain PhD), University of Dhaka, Dhaka, Bangladesh; Department of Internal Medicine (M Hostiuc PhD), Department of Legal Medicine and Bioethics (Prof S Hostiuc PhD), Department of General Surgery (I Negoi PhD), Department of Anatomy and Embryology (R I Negoi PhD), Department of Diabetes, Nutrition and Metabolic Diseases (Prof A Pantea Stoian PhD), Department of Dermatology (M Tampa PhD), Carol Davila University of Medicine and Pharmacy, Bucharest, Romania; Department of Clinical Legal Medicine (Prof S Hostiuc PhD), National Institute of Legal Medicine Mina Minovici, Bucharest, Romania; Department of Epidemiology and Health Statistics (Prof G Hu PhD), Xiangya School of Nursing (H Tao MSc), Central South University, Changsha, China; Advanced Institute of Convergence Knowledge Informatics (Y Huang PhD), Graduate School of Engineering (Y Huang PhD), Systems Neuroscience (V K Tiwari MD), Tohoku University, Sendai, Japan; Department of Public Health and Community Medicine (Prof A Humayun PhD), Shaikh Zayed Postgraduate Medical Institute, Lahore, Pakistan; Department of Mathematical Sciences (O J Ibidoja PhD), Federal University Gusau, Nigeria, Gusau, Nigeria; Physiotherapy Department (A A Ibrahim PhD), Faculty of Pharmacy (Prof H I M. Amin PhD), Tishk International University, Erbil, Iraq; Faculty of Pharmacy (U I Ibrahim PhD), Sultan Zainal Abidin University, Malaysia, Terengganu, Malaysia; Health Policy and Management Department (P M Iftikhar MD), City University of New York, New York, NY, USA; Department of Cardiovascular Medicine (J Ikram MD, J Rajendran MD), Cleveland Clinic, Cleveland, OH, USA; West Africa RCC (O S Ilesanmi PhD), Africa Centre for Disease Control and Prevention, Abuja, Nigeria; Faculty of Medical Sciences (Prof M D Ilic PhD), University of Kragujevac, Kragujevac, Serbia; Department of Clinical Pharmacy (M Imam PhD), Department of Health and Rehabilitation Sciences (Prof G Nambi PhD), Prince Sattam bin Abdulaziz University, Al Kharj, Saudi Arabia; Faculty of Health and Life Sciences (A Inok PhD), University of Exeter, Exeter, England; Department of Psychology (M Iqbal PhD), Department of Epidemiology and Biostatistics (Prof S Mubarik PhD), Wuhan University, Wuhan, China;

Institute for Evidence-Based Healthcare (Prof U Iqbal PhD), Bond University, Gold Coast, QLD, Australia; Faculty of Pharmacy (L M Irham PhD), Faculty of Public Health (Prof S Solikhah DrPH), Universitas Ahmad Dahlan, Yogyakarta, Indonesia; Department of Environmental Health (F Islam MPH), Poltekkes Kemenkes Mamuju (Health Polytechnic of the Ministry of Health, Mamuju), Mamuju, Indonesia; Research and Publication Department (M Islam MSc), World Health Organization (WHO), Dhaka, Bangladesh; Department of Public Health (M Islam PhD), International University of Business Agriculture and Technology (IUBAT), Dhaka, Bangladesh; 4-Green Research Socoiety, Bangladesh, Dhaka, Bangladesh (M Islam PhD); Department of Clinical Pharmacy & Pharmacy Practice (Prof N E Ismail PhD), Asian Institute of Medicine, Science and Technology, Bedong, Malaysia; Malaysian Academy of Pharmacy, Puchong, Malaysia (Prof N E Ismail PhD); Department of General Surgery and Medical-Surgical Specialties (Prof G Isola PhD), Department of Clinical and Experimental Medicine (Prof C Ledda PhD), Univertisy of Catania (F Leonforte MPH), University of Catania, Catania, Italy; Department of Internal Medicine (Y Issaka MPH), Bridgeport Hospital, Bridgeport, CT, USA; Department of Digital Health (Prof M Iwagami PhD), University of Tsukuba, Tsukuba, Japan; Department of Non-Communicable Disease Epidemiology (Prof M Iwagami PhD), Department of Health Services Research and Policy (M Karanikolos PhD, Prof M McKee DSc), London School of Hygiene & Tropical Medicine, London, England; College of Nursing (J Jacob MSc), Department of Psychiatry (A Parmar DM), All India Institute of Medical Sciences, Bhubaneswar, India; Department of Physical Medicine and Rehabilitation (L Jacob MD), Université Paris Cité, Paris, France; Research and Development Unit (L Jacob MD), Biomedical Research Networking Center for Mental Health Network (CiberSAM), Barcelona, Spain; UCL Institute for Global Health (Prof S Jaffar PhD), University of London, London, England; College of Medicine and Health Sciences (H Jahrami PhD), Arabian Gulf University, Manama, Bahrain; Government Hospitals, Manama, Bahrain (H Jahrami PhD); Section of Economic & Social Sciences, Humanities & Arts (Prof M Jakovljevic PhD), The World Academy of Sciences UNESCO-TWAS, Trieste, Italy; Shaanxi University of Technology, Hanzhong, China (Prof M Jakovljevic PhD); Department of Environmental Engineering (Prof R Jalilzadeh Yengejeh PhD), Islamic Azad University, Ahvaz, Iran; Department of Public Health Sciences (A Jamal BS), Pritzker School of Medicine (Prof H Yao PhD), University of Chicago, Chicago, IL, USA (Prof J Wan PhD); Public Health Dentistry (A James MDS), Department of Dermatology (F Kaliyadan MD), Amrita Institute of Medical Sciences, Kochi, India; Royal Prince Alfred Hospital (M Jamshidi PhD), Sydney Local Health District, Sydney, NSW, Australia; Department of Community Health Nursing, Princess Aisha Bint Al-Hussein College of Nursing (M Jarrar PhD), Al-Hussein Bin Talal University in Jordan, Maan, Jordan; Department of Community and Family Medicine (K M Jasani MD), All India Institute of Medical Sciences, Rajkot, India; The Medical City for Military and Security Services School, Muscat, Oman (S Jayapal PhD); Department of Biochemistry (Prof S Jayaram MD), Government Medical College, Mysuru, India; Centre for Evidence Synthesis and Implementation Research (Y A Jayasinghe BSc), Office of the Executive Director (Prof K K Kanmodi MPH), Cephas Health Research Initiative Inc, Ibadan, Nigeria (A A Salami BDS); Jindal School of Public Health and Human Development (Y A Jayasinghe BSc), O.P. Jindal Global University, Sonipat, India; Department of Dermatology (J Jeon MD), Institute of Evidence Based Medicine (J Jeon MD), Yonsei University Wonju College of Medicine, Wonju, South Korea; Faculty of Veterinary Medicine (M Jokar DVM), Cumming School of Medicine (Prof B Salami PhD), Department of Clinical Neurosciences (Prof S Wiebe MD), Department of Community Health Sciences (Prof S Wiebe MD), University of Calgary, Calgary, AB, Canada; Young Researchers and Elite Club (M Jokar DVM), Islamic Azad University, Karaj, Iran; Department of Community Medicine (J Jose MD), Jubilee Mission Medical College & Research Institute, Thrissur, Thrissur, India; School of Engineering and Technology (A

P Joseph PhD), The University of New South Wales, Canberra, NSW, Australia; Department of Economics (C E Joshua BSc), National Open University, Benin City, Nigeria; Department of Family Medicine and Public Health (J J Jozwiak PhD), University of Opole, Opole, Poland; Department of Medical Informatics (Prof S Jung PhD), Kangwon National University College of Medicine in South Korea, Chuncheon, South Korea; Department of Pathology (H K PhD), Saveetha Institute of Medical and Technical Sciences, Chennai, India; Department of Statistics (Prof D H Kadir PhD), Salahaddin University-Erbil, Erbil, Iraq; Faculty of Science (Prof A Kadri PhD), Al-Baha University, Al-Baha, Saudi Arabia; Department of Health (K Kalavani PhD), Khoy Medical Sciences, Khoy, Iran; Research Institute of Cardiology and Internal Medicine, Almaty (A Kaliyakparova MD), Research Institute of Cardiology and Internal Medicine, Almaty, Almaty, Kazakhstan; Department of Endocrinology (S Kalra DM), Bharti Hospital Karnal, Karnal, India; Care and Public Health Research Institute (CAPHRI) (R Kamath MHA), GROW- Institute for Oncology and Reproduction & NUTRIM - Institute for Nutrition and Translational Research (Y Sun MD), Maastricht University, Maastricht, Netherlands; Elderwood Rehabilitation Clinical Centre, Buffalo, NY, USA (R T Kamorudeen MPH); Department of Public Health (R T Kamorudeen MPH), South Wales University, Treforest, UK; The KU-KIST Graduate School of Energy and Environment (H Kamyab PhD), Korea University, Seongbuk-Gu, South Korea; Department of Public Health (Prof K K Kanmodi MPH), Thomas Adewumi University, Oko, Nigeria; The Hansjörg Wyss Department of Plastic and Reconstructive Surgery (R S Kantar MD), Institute for Excellence in Health Equity (M Kumar PhD), Rory Meyers College of Nursing (X Qi PhD), New York University, New York, NY, USA; 2nd Department of Cardiology (P Karakasis MD, P Stachteas MSc), Second Propedeutic Department of Internal Medicine (Prof D Patoulias PhD), Laboratory of Clinical Pharmacology (V Tseriotis MSc), Aristotle University of Thessaloniki, Thessaloniki, Greece; European Observatory on Health Systems and Policies, London, UK (M Karanikolos PhD); Department of Surgery (N I Kassa MD), MyungSung Medical College, Addis Ababa, Ethiopia; Department of Clinical Research and Epidemiology (K Kaushal MD, M Prasad MD), Institute of Liver and Biliary Sciences, New Delhi, India; Jindal School of Public Health and Human Development (Prof V R Keshri PhD), O. P. Jindal Global University, Sonipat, India, Sonipat, India; Amity Institute of Forensic Sciences (H Khajuria PhD, B P Nayak PhD), Amity institute of Public Health and Hospital Administration (A Koul MHA), Amity Institute of Pharmacy (K Munjal PhD), Amity University, Noida, India; National Center for Research, Khartoum, Sudan (Prof A Khalid PhD); Department of Pharmacy Administration and Clinical Pharmacy (F Khan PhD), Third Hospital (S Li PhD), Department of Epidemiology and Biostatistics (Prof J Liu PhD), National Institute of Health Data Science (W Wang PhD), School of Public Health (Y Wang MD), Peking University, Beijing, China; Neurology (M Khan PhD), University of Tennessee, Memphis, TN, USA; Department of Biotechnology and Microbiology (M Khan PhD), M.J.P. Rohilkhand University, Bareilly, India; Department of Pharmacy (M Khan MPhil), Minhaj University Lahore, Lahore, Pakistan; Department of Community and Preventive Medicine (R Khan MD), Department of Community Medicine and Public Health (M Safiullah MBBS), King Edward Medical University, Lahore, Pakistan; International Center for Chemical and Biological Sciences (S Khan PhD), International Center for Chemical and Biological Sciences, Karachi, Pakistan; Department of Cardiology (Z Khan MD), University of South Wales, Treforest, UK; Department of Cardiology (Z Khan MD), University of Buckingham, Buckingham, UK; Department of Pharmacology (S U Khasbage MD, P Thangaraju MD), All India Institute of Medical Sciences, Raipur, India; College of Health, Wellbeing and Life Sciences (Prof K Khatab PhD), Sheffield Hallam University, Sheffield, UK; College of Arts and Sciences (Prof K Khatab PhD), Ohio University, Zanesville, OH, USA; Asadabad School of Medical Sciences (A Khazaei PhD), Asadabad School of Medical Sciences, Asadabad, Iran; Research Department (M Khosrowjerdi PhD), University of Inland Norway,

Elverum, Norway; School of Medicine (Prof K Kim PhD), Creighton University, Omaha, NE, USA; Cardiovascular Disease Initiative (M Kim MD), Broad Institute of MIT and Harvard, Cambridge, MA, USA; Health and Healing Research, Education, and Service, Inc., Boston, MA, USA (R W Kimokoti MD); Millennium Prevention, Inc., Westwood, MA, USA (R W Kimokoti MD); School of Health Sciences (Prof A Kisa PhD), Kristiania University College, Oslo, Norway; Department of International Health and Sustainable Development (Prof A Kisa PhD), Tulane School of Public Health and Tropical Medicine (E Lytvyak MD), Department of Environmental Health Sciences (S P Sherchan PhD), Tulane University, New Orleans, LA, USA; Department of Public Health Dentistry (Prof S KM Kondlahalli PhD), Krishna Vishwa Vidyapeeth Deemed to be University, Karad, India; Global Healthcare Consulting, New Delhi, India (S Kochhar MD); Department of Public Health and Community Medicine (P Kodali PhD), Central University of Kerala, Kasaragod, India; Center for Tobacco Control Research and Education (P Kodali PhD), Department of Neurosurgery (A Orscelik MD), School of Nursing (J Ouner PhD), Global Brain Health Institute (D Simpong PhD), Department of Bioengineering and Therapeutical Sciences (Prof M Zastrozhin PhD), University of California San Francisco, San Francisco, CA, USA; Department of Internal Medicine (M Kokkorakis MD), Department of Genetics (S Pawar PhD), Yale University, New Haven, CT, USA; Department of Science and Environmental Studies (T Kormoker MSc), The Education University of Hong Kong, Hong Kong, China; Department of General Practice and Family Medicine (Prof O Korzh DSc), Kharkiv National Medical University, Kharkiv, Ukraine; Department of Public Health (M Koscik PhD), Masaryk University, Brno, Czech Republic; Feinberg School of Medicine (A S Kozhumam MSc), Medical Scientist Training Program (S Marzouk MA), Northwestern University, Chicago, IL, USA; School of Pharmacy (Prof I A Kretchy PhD), University of Ghana, Legon, Ghana; Department of Anthropology (Prof K Krishan PhD), Department of Forensic Science (Prof V Sharma PhD), Panjab University, Chandigarh, India; Department of Physiology (B Krishnan MD), Sree Balaji Medical College and Hospital, Chennai, India; College of Medicine (S Kukreti PhD), National Cheng Kung University, Tainan, Taiwan; Department of Research Management (M Kulimbet MSc), Research Institute of Cardiology and Internal Diseases, Almaty, Kazakhstan; Department of Health Administration and Education (E Kumah PhD), University of Education, Winneba, Winneba, Ghana; Amity Institute of Health Allied Sciences (Prof C Kumar PhD), Amity University Noida, Uttar Pradesh, Noida, India; Department of Community Medicine (D Kumar MD), Department of Forensic Medicine and Toxicology (Prof B Singh PhD), Rajendra Institute of Medical Sciences, Ranchi, India; Department of Mathematics (Prof K Kumar PhD), Amity University Haryana, Gurugram, India; Shaheed Mohtarma Benazir Bhutto Medical College Lyari (L Kumar MBBS), Department of Medicine (S Kumar MBBS), Department of Cardiology (A Raja MD), Dow University of Health Sciences, Karachi, Pakistan; Department of Psychiatry (M Kumar PhD), School of Nursing Sciences (M N Wanjau PhD), University of Nairobi, Nairobi, Kenya; Department of Community Medicine (M Kumar MD), Vardhman Mahavir Medical College and Safdarjung Hospital, Delhi, India; Department of Pharmacology and Toxicology (N Kumar PhD), National Institute of Pharmaceutical Education and Research, Hajipur, Hajipur, India; Wits Advanced Drug Delivery Platform Research Unit (Prof P Kumar PhD), Wits Infectious Diseases and Oncology Research Institute (Prof P Kumar PhD), University of the Witwatersrand, Johannesburg, South Africa; Faculty of Education, Shree Guru Gobind Singh Tricentenary University (S Kumar PhD), Department of Forensic Science (Prof B K Sharma PhD), Shree Guru Gobind Singh Tricentenary University, Gurugram, India; Department of Economics (V Kumar PhD), Manipal University Jaipur, Jaipur, India; School of Medicine and Dentistry (S Kundu MPH, M N Wanjau PhD), Griffith University, Gold Coast, QLD, Australia; Division of Research Methodology (Prof D Kurpas MD), Wroclaw Medical University, Wroclaw, Poland; Institute for Health Sciences (C Kustanti PhD), STIKES Bethesda

Yakkum Yogyakarta Indonesia, Yogyakarta, Indonesia; Department of Public Health and Epidemiology (D Kusuma DSc), Khalifa University of Science and Technology, Abu Dhabi, United Arab Emirates; Faculty of Public Health (D Kusuma DSc), University of Indonesia, Depok, Indonesia; Department of Pediatric Oncology (Prof T Kutluk MD), Medicana Health International, Istanbul, Türkiye; Department of Pediatric Oncology (Prof T Kutluk MD), Hacettepe University, Ankara, Türkiye; Centre for Vision across the Life Span (M A Kwarteng PhD), University of Huddersfield, Huddersfield, England; Clinical Research Center (V Kytö MD), Turku University Hospital, Turku, Finland; Heart Center (V Kytö MD), University of Turku and Turku University Hospital, Turku, Finland; School of Nursing and Midwifery (D A LaGrappe MSNMPH), La Trobe University, Bundoora, VIC, Australia; Division of Evidence Synthesis (C Lahariya MD), Foundation for People-centric Health Systems, New Delhi, India; Division of Lifestyle Medicine (C Lahariya MD), Centre for Health: The Specialty Practice, New Delhi, India; School of Digital Science (D Lai PhD), Institute of Applied Data Analytics (D Lai PhD), (University of Brunei Darussalam) (Universiti Brunei Darussalam), Bandar Seri Begawan, Brunei; Department of Occupational and Environmental Health, School of Public Health (H Lai PhD), Huazhong University of Science and Technology, Wuhan, China; Department of Communicable Diseases (A Velayudhan MPH), Indian Council of Medical Research, New Delhi, India (D K Lal MD); NEVES Society for Patient Safety, Budapest, Hungary (J Lám PhD); Department of Behavioural Sciences and Learning (Prof A Laplante-Lévesque PhD), Linköping University, Linköping, Sweden; Department of Medical Sciences (Prof A O Larsson PhD), Uppsala University, Uppsala, Sweden; Department of Clinical Chemistry and Pharmacology (Prof A O Larsson PhD), Uppsala University Hospital, Uppsala, Sweden; Department of Otorhinolaryngology (Prof S Lasrado MS), Father Muller Medical College, Mangalore, India; Department of Clinical Pharmacy and Pharmacy Management (B K Lawal PhD), Kaduna State University, Kaduna, Nigeria; Graduate School of Transdisciplinary Health Sciences (M Lee PhD), Yonsei University, Seoul, South Korea; Department of Precision Medicine (Prof S Lee MD), Sungkyunkwan University, Suwon-si, South Korea; Department of Family Medicine (W Lee PhD), University of Texas Medical Branch, Galveston, TX, USA; Department of Cardiothoracic and Vascular Surgery (V Leivaditis PhD), Westpfalz Klinikum, Kaiserslautern, Germany; Department of Cardiothoracic Surgery (V Leivaditis PhD), University of Patras, Patras, Greece; Department of Integrated Hygiene, Organizational, and Service Activities (F Leonforte MPH), AOU Polyclinc "G.Rodolico-San Marco" Catania, Catania, Italy; Nutrition & Health Innovation Research Institute (C Li MPH), Edith Cowan University, Perth, WA, Australia; Stockholm Resilience Centre (H Li PhD), Stockholm University, Stockholm, Sweden; Department of Health Promotion and Health Education (M Li PhD), National Taiwan Normal University, Taipei, Taiwan; School of Nursing and Health Sciences (X Liu PhD), Department of Population Health (Prof G Tse PhD), Hong Kong Metropolitan University, Hong Kong, China; School of Management (Z Liu PhD), School of Chinese Medicine (Prof J Wan PhD), School of Traditional Chinese Medicine (Prof H Yao PhD), Beijing University of Chinese Medicine, Beijing, China; Department of Medicine and Mental Health (Prof N C Lokunarangoda MD), University of Moratuwa, Sri Lanka, Moratuwa, Sri Lanka; Department of Population Health Sciences (J F Lopez MD), School of Medicine (Prof S Tang PhD), Duke University, Durham, NC, USA; Durham Center of Innovation to Accelerate Discovery and Practice Transformation (J F Lopez MD), US Department of Veterans Affairs (VA), Durham, NC, USA; School of Medicine (J López-Gil PhD), Universidad Espíritu Santo, Samborondón, Ecuador; Vicerrectoría de Investigación y Postgrado (J López-Gil PhD), Universidad de Los Lagos, Osorno, Chile; Center for Research and Innovation (A Loreche MS, V F Pepito MSc), Ateneo De Manila University, Pasig City, Philippines; Ashok & Rita Patel Institute of Physiotherapy (S D Lourembam PhD, D Modi MPT, S P Swami MPT), Charotar University of Science and Technology, Anand, India; School of Medicine (Prof G Lucchetti PhD), Federal University of Juiz de Fora,

Juiz de Fora, Brazil; Department of Epidemiology (H Lv MD), Army Medical University, Chongqing, China; College of Engineering (Prof M D Lytras PhD), Effat University, Jeddah, Saudi Arabia; Management of Information Systems Department (Prof M D Lytras PhD), The American College of Greece, Aghia Paraskevi, Greece; Department of Pharmaceutical Science (Prof H I M. Amin PhD), University of Eastern Piedmont, Novara, Italy; Center for Global Health (K Ma DDS), Department of Biostatistics, Epidemiology, and Informatics (J Puvvula PhD), Children's Hospital of Philadelphia (B B Tabor MD), University of Pennsylvania, Philadelphia, PA, USA; Department of Periodontology (Prof M Machoy-Rakoczy PhD), Pomeranian Medical University, Szczecin, Poland; Department of One Health in Tropical Infectiousness Diseases (S A Mahamed MSc), Jigjiga University, Jigjiga, Ethiopia; College of Health Science (S A Mahamed MSc), Amoud University, Borama, Somalia; Research Center (N H Mahmood PhD), College of Health Sciences (F M Rahman PhD), Cihan University-Sulaimaniya, Sulaymaniyah, Iraq; Edson College of Nursing and Health Innovation (K Mahmoud PhD), Arizona State University, Phoenix, AZ, USA; Gene Solutions, Ho Chi Minh city, Viet Nam (H X Mai MSc); Department of Psychology (I F Makhdoom PhD), University of Sargodha, Sargodha, Pakistan; Department of Epidemiology and Biostatistics (Prof M Mansourian PhD), Department of Medical Physics (K Saber PhD), Isfahan University of Medical Sciences, Isfahan, Iran; Biomedical Engineering Research Center (CREB) (Prof M Mansourian PhD), Universitat Politècnica de Catalunya (Barcelona Tech - UPC), Barcelona, Spain; Institute of Labour Economics (H Markazi Moghadam PhD), Leibniz University Hannover, Hannover, Germany; RGS Econ (H Markazi Moghadam PhD), Ruhr Graduate School in Economics, Essen, Germany; Health, Policy and Population Research Center (CIPPS) (A Martinez-Valle PhD), School of Medicine (Prof R Lozano MD), National Autonomous University of Mexico, Mexico City, Mexico; Harvard University, Boston, VA, USA (A Martinez-Valle PhD); Department of Community Medicine (J Mary MD), Sri Balaji Vidyapeeth (Deemed to be University), Pondicherry, India; Faculty of Humanities and Health Sciences (Prof R R Marzo MD), Curtin University, Sarawak, Malaysia; Jeffrey Cheah School of Medicine and Health Sciences (Prof R R Marzo MD), Monash University, Subang Jaya, Malaysia; Department of Anatomy and Developmental Biology (Y Mathangasinghe PhD), Monash University, Clayton, VIC, Australia; Department of Anatomy, Genetics and Biomedical Informatics (Y Mathangasinghe PhD), Postgraduate Institute of Medicine (Prof S N K Navaratna MD), University of Colombo, Colombo, Sri Lanka; Department of Health Policy Research (M R Mathur PhD), Public Health Foundation of India, Gurugram, India; Institute of Population Health Sciences (M R Mathur PhD), University of Liverpool, Liverpool, England; Department of Community Medicine (N Mathur MD, S Yahoo MD), Apollo Institute of Medical Sciences and Research, Hyderabad, India; Department of Epidemiology (Prof R J Maude PhD), Mahidol-Oxford Tropical Medicine Research Unit, Bangkok, Thailand; Nuffield Department of Medicine (Prof R J Maude PhD, Y Zhao DPhil), Radcliffe Department of Medicine (T Vinh BS), University of Oxford, Oxford, England; Department of International Public Health (S Mayaki MSc), Liverpool School of Tropical Medicine, Liverpool, England; Department of Social Medicine and Family (M Mazaheri PhD), Dezful University of Medical Sciences, Dezful, Iran; Australian Centre for Health Services Innovation (Prof S M McPhail PhD), Queensland University of Technology, Kelvin Grove, QLD, Australia; Digital Health and Informatics Directorate (Prof S M McPhail PhD), Queensland Health, Brisbane, QLD, Australia; Centre for Health Innovation and Policy, Noida, India (Prof R Mehrotra PhD); Department of Dental Research Cell (Prof V Mehta PhD), Dr. D. Y. Patil University, Pune, India; College of Medicine (Prof Z A Memish MD, Prof M Temsah MD), Alfaisal University, Riyadh, Saudi Arabia; Department of Medical Microbiology and Immunology (G A Menezes PhD), Trinity Medical Sciences University, St. Vincent, Saint Vincent and the Grenadines; Debre Berhan university (L A Mengstie MSc), Debre Berhan University, Debre Berhan, Ethiopia; General Administration

Department (A Meretoja MD), Comprehensive Cancer Center (T J Meretoja MD), Helsinki University Hospital, Helsinki, Finland; School of Health Sciences (A Meretoja MD), Melbourne School of Population and Global Health (L Reifels PhD), University of Melbourne, Melbourne, VIC, Australia; University of Helsinki, Helsinki, Finland (T J Meretoja MD); Medical Area Department (G Merlino PhD), University of Udine, Udine, Italy; College of Pharmacy (Prof M A Merza PhD), Nawroz University, Duhok, Iraq; University Centre Varazdin (T Mestrovic PhD), University North, Varazdin, Croatia; Department of Pathology, Faculty of Veterinary Medicine, Zagazig University (Prof M M M Metwally PhD), Cardiovascular Department (Prof A M A Saad MD), Zagazig University, Zagazig, Egypt; Faculty of Sciences (S D D Mewoabi PhD), University of Buea, Cameroon, Buea, Cameroon; Research Center for Public Health and Nutrition (M Mikrajab MPH), National Research and Innovation Agency, Bogor District, Indonesia; Multidisciplinary Department of Medical-Surgical and Dental Specialties (G Minervini PhD), University of Campania Luigi Vanvitelli, Naples, Italy; Department of Public Health Dentistry (Prof G Mini PhD), Saveetha Institute of Medical and Technical Sciences (SIMATS), Chennai, India; Global Institute of Public Health (Prof G Mini PhD), Ananthapuri Hospitals and Research Institute, Trivandrum, India; Internal Medicine Department (Prof E M Mirrakhimov MD), Kyrgyz State Medical Academy, Bishkek, Kyrgyzstan; Department of Atherosclerosis and Coronary Heart Disease (Prof E M Mirrakhimov MD), National Center of Cardiology and Internal Disease, Bishkek, Kyrgyzstan; Thumbay College of Management and AI in Healthcare (V Mishra PhD), Gulf Medical University, Ajman, United Arab Emirates; Research and Development Department (V Mishra PhD), Panacea Institute of Interdisciplinary Research and Education, Varanasi, India; Department of Forensic Medicine and Toxicology (C Mittal MD), All India Institute of Medical Sciences, Patna, India; RAK College of Nursing (M Mohamed PhD), RAK Medicak and Health Sciences University, Ras Al-Khaimah, United Arab Emirates; Nursing College (M Mohamed PhD), Sohag University, Sohag, Egypt; Molecular Biology Unit (N S Mohamed MSc), Bio-Statistical and Molecular Biology Department (N S Mohamed MSc), Sirius Training and Research Centre, Khartoum, Sudan; Department of Pathophysiology (K Mohamed Ahmed MD), St. George's University, St.George’s, Grenada; Department of Medicine (O Mohammed MBBS), Government Medical College Kozhikode, Kozhikode, India; School of Health Sciences (S Mohan PhD), University of Petroleum and Energy Studies, Dehradun, India; Department of Applied Biology (Y Mohanta PhD), University of Science and Technology Meghalaya, Ri-Bhoi, India; Analytical and Applied Economics (S S Mohanty PhD), Department of Analytical and Applied Economics (Prof H Rout PhD, C Swain MPhil), RUSA Centre of Excellence in Public Policy and Governance (Prof H Rout PhD), UGC Centre of Advanced Study in Psychology (Prof M Satpathy PhD), Utkal University, Bhubaneswar, India; AI & Cyber Futures Institute (M Moni PhD), Charles Sturt University, Bathurst, NSW, Australia; Department of Collective Prevention and Public Health (M Montalti MD), General Directorate for Personal Care, Health, and Welfare, Bologna, Italy; Social Determinants of Health Research Center (M Moradi-Joo PhD), Yasuj University of Medical Sciences, Yasuj, Iran; Department of Health Sciences (Ph.D. Program) (Prof D F Morais MSc), Institute for Medical Assistance to State Public Servants, São Paulo, Brazil; Division of Plastic and Reconstructive Surgery (S D Morrison MD), University of Washington Medical Center, Seattle, WA, USA; Facultad de Salud (Prof F E C Mosquera MSc), Universidad Santiago de Cali, Santiago de Cali, Colombia; Department of Health Policy (Prof E Mossialos PhD), London School of Economics and Political Science, London, England; Nutrology Fellowship (N Mozart Vieira MD), Nutrology Academy, São Paulo, Brazil; René Rachou Institute (M Mrejen PhD), Oswaldo Cruz Foundation, Belo Horizonte, Brazil; PMAS Arid Agriculture University Rawalpindi, Rawalpindi, Pakistan (R Mubarak MSc); Unit of Pharmacotherapy, Epidemiology and Economics (Prof S Mubarik PhD), University of Groningen (Rijksuniversiteit

Groningen), Groningen, Netherlands; Department of Medicine, Integrated Medicine Programme, Alexandra Hospital (A Mukhopadhyay MD), National University Health System, Singapore, Singapore; Department of Surgery (F Mulita PhD), General University Hospital of Patras, Patras, Greece; Faculty of Medicine (F Mulita PhD), University of Thessaly, Larissa, Greece; School of Public Health (D Musoke PhD), Makerere University, Kampala, Uganda; Department of Pediatrics & Pediatric Pulmonology (Prof G Mustafa MD), Institute of Mother & Child Care, Multan, Pakistan; Business Analytics and Artificial Intelligence (M T Mustapha PhD), University of Texas, Richardson, TX, USA; Irfan Suat Gunsel Operational Research Institute (M T Mustapha PhD, Prof D Uzun Ozsahin PhD), Near East University, Mersin, Türkiye; Department of Research Methods (S Muthu PhD), Orthopaedic Research Group, Coimbatore, India; Central Research Laboratory (S Muthu PhD), Vinayaka Mission's Research Foundation (Deemed to be University), Puducherry, India; Rwanda Biomedical Centre, Kigali, Rwanda (Prof C M Muvunyi PhD); Department of Public Health (L Muzaqi Master of Occupational Safety and Health), Universitas Negeri Semarang, Semarang, Indonesia; Department of Psychiatry (W Myung PhD), Seoul National University, Seoul, South Korea; Department of Neuropsychiatry (W Myung PhD), Seoul National University Bundang Hospital, Seongnam, South Korea; Elderly Health Research Center (A Nafei PhD), Research and Academic Institution, Tehran, Iran; Neurosciences Research Center (M Naghshbandi MD), Social Determinants of Health Research Center (A Shokri PhD), Kurdistan University of Medical Sciences, Sanandaj, Iran; Department of Medicine (H Naik MS), Department of Science (D Tanabayeva BSc), University of British Columbia, Vancouver, BC, Canada; Faculty of Pharmacy (Prof F Nainu PhD), Hasanuddin University, Makassar, Indonesia; College of Health Sciences (H H R Najmuldeen PhD), Cihan University Sulaimaniya, Sulaimaniya, Iraq; Division of Endocrinology and Diabetes (M Nassar PhD), University of Vermont, South Burlington, VT, USA; College of Public Health (K Nayak MPH), Kent State University, Kent, OH, USA; Department of General Surgery (I Negoi PhD), Emergency University Hospital Bucharest, Bucharest, Romania; Department of Cardiology (R I Negoi PhD), Cardio-Aid, Bucharest, Romania; Department of Cardiology (A G Negru PhD), University of Medicine and Pharmacy "Victor Babes", Timisoara, Romania; Rocordis Heart Center (A G Negru PhD), Cardiology and Cardiovascular Surgery Hospital, Timisoara, Romania; Euromed Research Center (Prof C Nejjari MD), Euromed University of Fes, Fez, Morocco; Faculty of Medicine, Pharmacy, and Dentistry (Prof C Nejjari MD), University Sidi Mohammed Ben Abdellah, Fez, Morocco; Department of Public Health (N Nekliudov MD, F Porru MD), Erasmus University Medical Center, Rotterdam, Netherlands; International Institute of Training and Research (INSTAR) (T T Nguyen MPH), University of Medicine and Pharmacy, Vietnam National University (UMP-VNU), Hanoi, Viet Nam; Faculty of Public Health (T Nguyen PhD, L Vu PhD), International Institute for Training and Research (INSTAR) (L Vu PhD), VNU University of Medicine and Pharmacy, Hanoi, Viet Nam; Institute for Mental Health Policy Research (Y T Nigatu PhD), Centre for Addiction and Mental Health, Toronto, ON, Canada; Global Health Policy Lab (Prof S Nomura PhD), Tohoku University, Miyagi, Japan; Department of Health Policy and Management (Prof S Nomura PhD), Keio University, Tokyo, Japan; Department of Microbiology and Molecular Genetics (M Noreen PhD), The Women University Multan, Multan, Pakistan; Faculty of Medicine (M Noushad PhD), Universiti Malaya, Kuala Lumpur, Malaysia; Department of Paediatrics (C A Nri-Ezedi PhD), Nnamdi Azikiwe University, Awka, Nigeria; Department of Radiology (F Nugen PhD), Mayo Clinic, Rochester, MN, USA; School of Information (F Nugen PhD), University of California Berkeley, Berkeley, CA, USA; Department of Applied Economics and Quantitative Analysis (Prof B Oancea PhD), University of Bucharest, Bucharest, Romania; Bioinformatics Department (Prof B Oancea PhD), National Institute of Research and Development for Biological Sciences, Bucharest, Romania; Department of Nursing Science (O C Ogidan

PhD), Ekiti State University, Ado-Ekiti, Ado-Ekiti, Nigeria; Department of Physiotherapy, Faculty of allied health sciences. (O O Ojedoyin PhD), Federal University of Health Sciences Ila Orangun Osun, Ila-Orangun, Nigeria; Department of Physiotherapy (O O Ojedoyin PhD), University of KwaZulu-Natal, Durban, South Africa; Department of Medical Laboratory Science (O J Okesanya MPH), Federal Neuropsychiatric Hospital, Abeokuta, Nigeria; Department of Psychiatry and Behavioural Neurosciences (Prof A T Olagunju PhD), McMaster University, Hamilton, ON, Canada; Department of Psychiatry (Prof A T Olagunju PhD), University of Lagos, Lagos, Nigeria; Global Health (A Olaniran PhD), KIT Royal Tropical Institute, Amsterdam, Netherlands; Cardiology Department (Prof G M Oliveira PhD), Federal University of Rio de Janeiro, Rio de Janeiro, Brazil; Centre for Healthy Start Initiative, Lagos, Nigeria (B O Olusanya PhD); Department of Pharmacology and Therapeutics (O G Oluwole PhD), Olabisi Onabanjo University, Sagamu, Nigeria; Institute of Infectious Disease and Molecular Medicine (O G Oluwole PhD), University of Cape Town, Cape Town, South Africa; Jadara Research Center (A Omar Bali PhD), Jadara University, Irbid, Jordan; Department of Pharmacology and Therapeutics (Prof O E Onwujekwe PhD), University of Nigeria Nsukka, Enugu, Nigeria; Centre of Regenerative Medicine (M Ordak PhD), Medical University of Bialystok, Bialystok, Poland; Sickle Cell Unit (Prof V N Orish PhD), Ho Teaching Hospital, Ho, Ghana; One Health Research Group (Prof E Ortiz-Prado PhD), Universidad De Las Américas, Quito, Ecuador; Department of Biological Sciences (A Osborne MSc), Njala University, Freetown, Sierra Leone; Division of Infectious Diseases (Prof A Ouyahia PhD), University Hospital of Setif, Setif, Algeria; Department of Global Public Health and Primary Care (Prof S Øverland PhD), University of Bergen, Bergen, Norway; Department of Biological Sciences (K A Oyeniran PhD), Bamidele Olumilua University of Education Science & Technology, Ikere-Ekiti, Nigeria; Plant Systems Biology (K A Oyeniran PhD), International Center for Genetic Engineering & Biotechnology (ICGEB), Cape Town, South Africa; Irfan Suat Gunsel Operational Research Institute (I Ozsahin PhD, Prof Y Waheed PhD), Near East University, Nicosia, Türkiye; Institute of Physiotherapy (V Palaniswamy PhD), Srinivas University, Mangalore, India; Department of Community Medicine (A Pana PhD), Iuliu Hatieganu University of Medicine and Pharmacy Cluj Napoca, Cluj Napoca, Romania; National Center for Statistics in Public Health (A Pana PhD), National Institute of Public Health, Bucharest, Romania; Centre for Biotechnology (S K Panda PhD), Siksha 'O' Anusandhan Deemed to be University, Bhubaneswar, India; Department of Pharmacy (B Pandey PHARMACY), Madan Bhandari Academy of Health Sciences, Hetauda, Nepal; Amity Institute of Forensic Sciences (P Pandya PhD), Amity University, Noida, Gautam Buddh Nagar, India; College of Nursing, Krida Wacana Christian University, Jakarta, Indonesia (S M Pangaribuan MSc), Krida Wacana Christian University, Jakarta, Indonesia, Taipei, Indonesia; Medical University of Vienna, Vienna, Austria (I Papadimopoulos MD); Vision and Eye Research Institute (Prof S Pardhan PhD, M Piyasena PhD), Anglia Ruskin University, Cambridge, England; Division of Health Policy and Management (R R Parikh MD), Department of Surgery (J Rickard MD), University of Minnesota, Minneapolis, MN, USA; Department of Sociology, Anthropology, and Public Health (C Park DrPH), University of Maryland Baltimore County, Baltimore, MD, USA; Department of Medical Sciences (R Passera PhD), University of Torino, Torino, Italy; Department of Imaging (R Passera PhD), AOU Città della Salute e della Scienza di Torino (AOU City of Health and Science of Turin), Torino, Italy; Department of Computer Science and Bioscience (M Patel PhD), Marwadi University, Rajkot, India; Department of Cardiovascular Medicine (N N Patel MD), University of Tennessee, Nashville, TN, USA; College of Dental Medicine (Prof S Patil PhD), Roseman University of Health Sciences, South Jordan, UT, USA; Department of Human Anatomy (A Patra MD), Department of Psychiatry (J Singh MD), All India Institute of Medical Sciences, Bathinda, India; Department of Community Medicine (S Pattnaik MD), Mallareddy Medical College for Women,

Hyderabad, India; Faculty of Humanities and Social Sciences (U Paudel PhD), Tribhuvan University, Kathmandu, Nepal; IRCCS Fondazione Don Carlo Gnocchi, Milan, Italy (P Pedersini MSc); Department of Clinical and Experimental Sciences (P Pedersini MSc), University of Brescia, Brescia, Italy; Australian Institute of Health Innovation (P Peprah MSc), Macquarie University, Sydney, NSW, Australia; Department of Psychiatry (Prof M F P Peres MD), University of Sao Paulo, São Paulo, Brazil; International Institute for Educational Planning (IIEP) (Prof M F P Peres MD), Albert Einstein Hospital, São Paulo, Brazil; Research Advancement Consortium in Health, Hanoi, Viet Nam (T T Pham PhD); Departments of Psychiatry and Epidemiology (Prof M R Phillips MD), Columbia University Mailman School of Public Health (J M F Zhang MD), Columbia University, New York, NY, USA (D Shan PhD); Széchenyi István University, Győr, Pakistan (Z Z Piracha PhD); International Center of Medical Sciences Research (ICMSR), Islamabad, Pakistan (Z Z Piracha PhD); Department of Promoting Health, Maternal-Infant, Excellence and Internal and Specialized Medicine (PROMISE) G. D'Alessandro (E Pirera MD), Department of Promoting Health, Maternal-Infant, Excellence and Internal and Specialized Medicine (ProMISE) "G. D'Alessandro" (E Pirera MD), University of Palermo, Palermo, Italy; Mental Health Research Institute (E Plotnikov PhD), Tomsk National Research Medical Center, Tomsk, Russia; Siberian State Medical University, Tomsk, Russia (E Plotnikov PhD); Academy of Romanian Scientists, Bucharest, Romania (Prof I Popa PhD); Department of Occupational Health and Safety Engineering (Prof M Poursadeqiyan PhD), Ardabil University of Medical Science, Ardabil, Iran; Department of Clinical and Experimental Medicine (Prof N Pugliese PhD, D Trico MD), University of Pisa, Pisa, Italy; Institute of Microbiology (M Qamar PhD), Government College University Faisalabad, Faisalabad, Pakistan; Department of Medical Instrumentation Techniques Engineering (N H Qasim DSc), Al-Rafidain University, Baghdad, Iraq; Department of Mathematics and Informatics (N H Qasim DSc), Luhansk Taras Shevchenko National University, Luhansk, Ukraine; Department of Medical Oncology (Prof V Radhakrishnan MD), Cancer Institute (W.I.A), Chennai, India; Department of Epidemiology (P Raghuveer MD), National Institute of Mental Health and Neurosciences, Bengaluru, India; Department of Environmental Health Engineering (S Rahimi PhD), Health Science Research Centre (S Rahimi PhD), Health Sciences Research Center (M Sarmadi MSc), Torbat Heydariyeh University of Medical Sciences, Torbat Heydariyeh, Iran; Institute of Epidemiology, Disease Control and Research (IEDCR), Dhaka, Bangladesh (M Rahman PhD); Department of Population Science and Human Resource Development (Prof M Rahman DrPH), University of Rajshahi, Rajshahi, Bangladesh; Institute of Health and Wellbeing (Prof M Rahman PhD), Federation University Australia, Berwick, VIC, Australia; School of Public Health and Preventive Medicine (P Rai PhD), Monash University, Melbourne, VIC, Australia; Department of Chemical Engineerinag and Procesiing (Prof R Rakoczy DSc), West Pomeranian University of Technology in Szczecin, Szczecin, Poland; Department of Radiology (S Ramasamy MD), Stanford University, Stanford, CA, USA; Department of Biotechnology (Prof P W Ramteke PhD), Hislop College, Nagpur, India; Department of Molecular Biology & Genetic Engineering (Prof P W Ramteke PhD), RTM Nagpur University, Nagpur, India; AIIMS, Bilaspur (L Rana MD), All India Institute of Medical Sciences, Bilaspur, India; Centre for Clinical Pharmacology (Prof N Rancic PhD), Military Medical Academy, Belgrade, Serbia; Department of Community Medicine (M Rangappa MD), Rajiv Gandhi University, Karnataka, India; Department of Health System Management Studies (M Rangasamy MD), JSS Academy of Higher Education & Research (Deemed to be University), Mysuru, India; Nitte (Deemed to be University), AB Shetty Memorial Institute of Dental Sciences (K Rao PhD), KS Hegde Medical Academy (Prof P Shetty MD), Nitte University, Mangalore, India; Department of Oral Pathology, Microbiology and Forensic Odontology (S J Rao MDS), Sharavathi Dental College and Hospital, Shimogga, India; Department of Family Medicine (Prof D Rathish PhD), Department of

Community Medicine (N Wickramasinghe MD), Rajarata University of Sri Lanka, Anuradhapura, Sri Lanka; Amity Institute of Public Health and Hospital Administration (J Rathore MBA), Amity University, Uttar Pradesh, Uttar Pradesh, India; Department of Public health (R Ravi MHAHSM), Amrita Institute of Medical Sciences, Ernakulam, India; Inovus Medical, St Helens, UK (D L Rawaf MD); Academic Public Health England (Prof S Rawaf MD), Public Health England, London, England; School of Health, Medical and Applied Sciences (L Rawal PhD), CQ University, Sydney, NSW, Australia; Department of Mathematical Demography & Statistics (R Rawat PhD), International Institute for Population Sciences, Mumbai, India; Interventional Cardiology Department (A Ray MD), University of Kentucky, morehead, KY, USA; Infection Prevention & Control (Prof M Raza PhD), King Saud bin Abdulaziz University for Health Sciences, Riyadh, Saudi Arabia; Department of Hematology (B Razi PhD), North Khorasan University of Medical Sciences, Bojnurd, Iran; IDEAS Research Group (C Reategui-Rivera MD), Universidad Científica del Sur, Lima, Peru (D Villarreal-Zegarra MPH); Kohat University of Science and Technology (Prof N U Rehman PhD), Kohat University of Science and Technology, Kohat, Pakistan; Natural and Medical Sciences Research Center (Prof N U Rehman PhD), Natural and Medical Sciences Research Center, Nizwa, Oman; Yenepoya Research Centre (B N Rekadwad PhD), Yenepoya University, Mangalore, India; Brien Holden Vision Institute, Sydney, NSW, Australia (Prof S Resnikoff MD); Department of Obstetrics and Gynecology (S Restaino MD), Azienda Sanitaria Universitaria Friuli Centrale, Udine, Italy; Unisabana Center for Translational Science (L Reyes PhD), Universidad de La Sabana (Savannah University), Chia, Colombia; Critical Care Department (L Reyes PhD), Clinica Universidad De La Sabana (Savannah University Clinic), Chia, Colombia; Network of Immunity in Infection, Malignancy and Autoimmunity (NIIMA) (Prof N Rezaei PhD), Universal Scientific Education and Research Network (USERN), Tehran, Iran; Department of Pharmacy (M Riaz PhD), Shaheed Benazir Bhutto University Sheringal Pakistan, Dir Upper, Pakistan; Department of Surgery (J Rickard MD), University Teaching Hospital of Kigali, Kigali, Rwanda; Department of Pharmacology and Toxicology (Prof J A B Rodriguez PhD), University of Antioquia, Medellin, Colombia; Warwick Medical School (Prof J A B Rodriguez PhD), University of Warwick, Coventry, England; Department of Clinical Research (Prof L Roever PhD), University of Sao Paulo, Ribeirão Preto, Brazil; Department of Community Medicine (R Rohilla MD), Government Medical College, Chandigarh, India; Miyan Research Institute (M Rony MPH), International University of Business Agriculture and Technology, Dhaka, Bangladesh; Department of Community Medicine (A Roy MD), RVM Medical College and Research Centre, Hyderabad, Hyderabad, India; Achutha Menon Centre for Health Science Studies (A Roy MD), Sree Chitra Tirunal Institute for Medical Sciences and Technology, Thiruvananthapuram, India; Department of Public Health (S Roy MD), New Mexico State University, Las Cruces, NM, USA; Department of Internal Medicine (G M Rwegerera MD), Muhimbili University of Health and Allied Sciences, Dar es Salaam, Tanzania; Department of Internal Medicine (G M Rwegerera MD), University of Botswana, Gaborone, Botswana; Department of Oral and Maxillofacial Surgery (C S N PhD), Jagadguru Sri Shivarathreeswara University, Mysore, India; Department of Food Technology (B A Sadee PhD), Salahaddin University, Erbil, Iraq; Department of Pharmaceutical Chemistry (Prof M Saeb PhD), International Medical University, Gdańsk, Poland; Széchenyi István University (Prof U Saeed PhD), Széchenyi István University, Győr, Hungary; Irfan Suat Gunsel Operational Research Institute (Prof U Saeed PhD), Near East University, Turkey, Nicosia, Türkiye; Department of Nursing and Midwifery (M Saeedi PhD), Saveh University of Medical Sciences, Saveh, Iran; Global Health Economy Research Team (S Saeedi Moghaddam MSc), Kiel Institute for the World Economy, Kiel, Germany; Department of Epidemiology (Prof I Saha PhD), Indian Council of Medical Research, Kolkata, India; Centre for Biotechnology (A Sahoo PhD), Siksha 'O' Anusandhan (Deemed to be University), Bhubaneswar, India;

Canadian Red Cross (K Sahu PhD), Red Cross, Ottawa, ON, Canada; College of Pharmacy (Prof S Sajadi PhD), Al-Hadba University, Mosul, Iraq; Department of Statistics (M R Sajid PhD), University of Gujrat, Gujrat, Pakistan; LMU-Munich, Munich, Germany (J W Sakshaug PhD); Institute for Employment Research, Nuremberg, Germany (J W Sakshaug PhD); Department of Human Nutrition & Dietetics (S Saleem PhD), School of Public health (Prof B T Shaikh PhD), Health Services Academy, Islamabad, Pakistan; Department of Biochemistry (A J Salemcity PhD), University of Medical Sciences, Ondo, Ondo city, Nigeria; Department of Global Initiatives (Prof G A Salum PhD), Child Mind Institute, New York, NY, USA; Department of Entomology (A M Samy PhD), Medical Ain Shams Research Institute (MASRI) (A M Samy PhD), Ain Shams University, Cairo, Egypt; Department of Genomics and Biotechnology (S Sankar PhD), Madha Medical College and Research Institute, Chennai, India; University of São Paulo City, São Paulo, Brazil (L H C C Santos MSc); Independent Consultant, Thiruvananthapuram, India (S Y I Saraswathy PhD); Department of Public Health (Y Sarikhani PhD), Jahrom University of Medical Sciences, Jahrom, Iran; Botany Department (H Sarma PhD), Bodoland University, Kokrajhar, India; Faculty of Science (M Sarmadi MSc), Queensland University of Technology, Brisbane, QLD, Australia; Department of Epidemiology (M Sathya Narayanan MBBS), National Institute for Research in Tuberculosis, Chennai, India; Udyam-Global Association for Sustainable Development, Bhubaneswar, India (Prof M Satpathy PhD); Carnegie Mellon University (A Saurabh MS), Carnegie Mellon University, Pittsburgh, PA, USA; Department of Public Health Sciences (S G Saxena DrPH), Coastal Carolina University, Conway, SC, USA; Harvard Extension School (S G Saxena DrPH), Department of Orthopaedics (O Subasi PhD), Harvard University, Cambridge, MA, USA; Department of Educational Sciences (Y Sayadi PhD), Farhangian University, Kermanshah, Iran; Chemistry Department (A Sayed MPharmSc), Purdue University, West Lafayette, IN, USA; Universidad ESAN (Prof C Schinckus PhD), Universidad ESAN, Lima, Peru; Graduate School of Business (Prof C Schinckus PhD), University of the Fraser Valley, Abbotsford, BC, Canada; Department of Psychiatry (Prof S Seedat PhD), Stellenbosch University, Cape Town, South Africa; Department of Marine Biotechnology (Prof C Selvaraj PhD), AMET University, Chennai, India; Faculty of Dentistry (Prof S Selvaraj PhD), University of Puthisastra, Phnom Penh, Cambodia; Emergency Department (S Senthilkumaran PhD), Manian Medical Centre, Erode, India; Department of Psychology and Health Sciences Faculty of Human Sciences, Education, and Sports (Prof F Sessa PhD), Pegaso Telematic University, Naples, Italy; Department of Medicine (Y Sethi MD), Swami Vivekanand Subharti University, Meerut, India; Charotar University of Science and Technology (CHARUSAT) (S A Shah MPT), Ashok and Rita Patel Institute of Physiotherapy, Changa, India; Rita A. Patel Institute of Physiotherapy (S Shah PhD), The Charutar Vidya Mandal(CVM) University, Anand, India; Dongguan Key Laboratory of Computer-Aided Drug Design (M Shahab PhD), Guangdong Medical University, Dongguan, China; State Key Laboratories of Chemical Resources Engineering (M Shahab PhD), Beijing University Of Chemical Technology, Beijing, China; Reasearch Centre for Health Sciences (RCHS) (W Shahid PhD), The University of Lahore, ; National Institute of Gastroenterology-IRCCS "Saverio de Bellis", Castellana Grotte, Italy (E Shahini MD); Department of Transitional Residency (A Shahriari MD), Lakeland Regional Health, lakeland, FL, USA; Independent Consultant, Karachi, Pakistan (M A Shaikh MD); Noncommunicable Diseases Research Center (A Shakerimoghaddam PhD), Neyshabur University of Medical Sciences, Neyshabur, Iran; Lancaster University, Lancaster, England (D Shan PhD); Department of Microbiology and Molecular Genetics (N Sharif MSc), University of Vermont, Burlington, VT, USA; Department of Microbiology (N Sharif MSc), Jahangirnagar University, Dhaka, Bangladesh; Department for Evidence-based Medicine and Evaluation (A Sharifan PharmD), University for Continuing Education Krems, Krems, Austria; School of Science and Technology (Prof P Sharifian Jazi PhD), The University of Georgia, Tbilisi,

Georgia; Amity Institute of Biotechnology (A Sharma PhD, E Upadhyay PhD), Amity University Rajasthan, Jaipur, India; Department of Physiotherapy (J Sharma PhD), Galgotas University, Greater Noida, India; UN Mehta Institute of Cardiology and Research Center (Prof K Sharma MD), B.J. Medical College, Ahmedabad, India; Department of Cardiology (Prof K Sharma MD), Government Medical College, Ahmedabad, India; Division of Physical Sciences (Prof K Sharma PhD), Karunya Institute of Technology and Sciences, Tamil Nadu, India; American University of the Middle East, Egaila, Kuwait (A Shehadeh PhD); Quantitative Department (A Sheidaei PhD), Non-Communicable Diseases Research Center (NCDRC), Tehran, Iran; Department of Microbiology (S M Shenoy MD), Kasturba Medical College, Mangalore, India; Department of Biology (S P Sherchan PhD), Morgan State University, Baltimore, MD, USA; Alva's Institute of Medical Sciences & Research Centre (Prof M Shetty MD), Rajiv Gandhi University of Health Sciences, Moodubidire, India; Rural Health Research Institute (Prof M Shiddiky PhD), Charles Sturt University, Orange, Austria; Department of Pharmacology (T Shimels MSc), Saint Paul's Hospital Millennium Medical College, Addis Ababa, Ethiopia; School of Biotechnology (K Shivangi MSc), Gautam Buddha University, gautam buddh nagar, India; Department of Medical-Surgical Nursing (S Shorofi PhD), Mazandaran University of Medical Sciences, Sari, Iran; Department of Research and Academics (S Shrestha PhD), Kathmandu Cancer Center, Bhaktapur, Nepal; Person-Centred Research Directorate, Eastern Health Clinical, School Faculty of Medicine, Nursing and Health Sciences (S Shrestha PhD), Monash University, Box Hill, VIC, Australia; School of Public Health (N Sidamo PhD), Arba Minch University, Arba Minch, Ethiopia; Department of Medical Microbiology and Infectious Diseases (E E Siddig MD), Erasmus University, Rotterdam, Netherlands; Research and Innovation (Prof P P Simkhada PhD), University of Chester, Chester, UK; School of Health (Prof C R Simpson PhD), Victoria University of Wellington, Wellington, New Zealand; Department of Dentistry (Prof A Singh MDS), All India Institute of Medical Sciences, Bhopal, India; Department of Pharmacology (H Singh DM), Government Medical College and Hospital, Chandigarh, India; School of Medicine (Prof J A Singh MD), Baylor College of Medicine, Houston, TX, USA; Department of Medicine Service (Prof J A Singh MD), US Department of Veterans Affairs (VA), Houston, TX, USA; Department of Human Genetics (P Singh PhD), Punjabi University, Patiala, Patiala, India; Biotechnology, Microbiology and Forensic Science (S Singh PhD), Noida International University, Greater Noida, India; Mehta Family School of Biosciences and Biomedical Engineering (S Singh MSc), Indian Institute of Technology Indore, Indore, India; ESIC Medical College and Hospital, Ranchi, India (R Sinha MD); SRM Medical College Hospital and Research Centre (G Sivan PhD), Sri Ramaswamy Memorial Institute of Science and Technology, Chengalpettu, India; Global and European Health Education and Study Institute (Prof N Skhvitaridze PhD), University of Georgia, Tbilisi, Georgia; National Center for Disease Control and Public Health, Tbilisi, Georgia (Prof N Skhvitaridze PhD); Books Committee (V Y Skryabin MD), Royal College of Psychiatrists, London, UK; Department of Oral and Maxillofacial Surgery (J Song PhD), the Affiliated Stomatological Hospital of Guizhou Medical University, Guiyang, China; 3rd Department of Cardiology (M Spartalis PhD), University of Athens, Athens, Greece; Department of Health (L V Sree MD), A.C.S. Medical College and Hospital, Hyderabad, India; College of Health and Public Service (S Sriram PhD), University of North Texas, Denton, TX, USA; Department of Psychiatry and Psychotherapy (Prof B Stubbs PhD), Medical University of Vienna, Comprehensive Center for Clinical Neurosciences and Mental Health (C3NMH), Vienna, Austria; Division of Psychology and Mental Health (Prof B Stubbs PhD), University of Manchester, Manchester, England; Discipline of Physiotherapy (P Stubbs PhD), University of Technology Sydney, Sydney, NSW, Australia; McGill University, Montreal, QC, Canada (C Su PhD); Department of Medical Sciences (Prof V Subramaniyan PhD), Sunway University, Subang Jaya, Malaysia; Faculty of Management (A Sugunan

PhD), Sri Ramaswamy Memorial Institute of Science and Technology, Tiruchirappalli, India; Hospital Administration (M Suhaib MHA), Sanjay Gandhi Postgraduate Institute of Medical Sciences, Lucknow, India; Department of Pathology (Prof O Sulaieva PhD), Medical Laboratory CSD, Kyiv, Ukraine; Research Unit (Prof O Sulaieva PhD), Ukrainian Association for Precision Medicine, Kyiv, Ukraine; Department of Medicine (S K Sulaiman MD), Yobe State University Teaching Hospital, Yobe, Nigeria; Department of Social Sciences (Prof M J M Sullman PhD), Department of Life and Health Sciences (Prof M J M Sullman PhD), University of Nicosia, Nicosia, Cyprus; School of Public Health (Z Sun PhD), Kunming Medical University, Kunming, China; Institute of Medical Sciences (Prof L Szarpak PhD), The John Paul II Catholic University of Lublin, Lublin, Poland; Department of Neurology (P Tabaee Damavandi MD), Neurocenter of Southern Switzerland (NSI), Lugano, Switzerland; Department of Medicine (Prof R Tabarés-Seisdedos PhD), University of Valencia, Valencia, Spain; Department of Basic Medical Sciences (S Tabatabaeizadeh PhD), Department of Internal Medicine (S Tabatabaeizadeh PhD), Islamic Azad University, Mashhad, Iran; Department of Health, Safety, and Environmental Management (R Tabibi PhD), Abadan University of Medical Sciences, Abadan, Iran; Department of Pharmacy (M Tabish MPH), Mangalayatan University, Aligarh, India; Department of Environmental, Agricultural and Occupational Health (J Taiba PhD), University of Nebraska Medical Center, Omaha, NE, USA; Department of Dermato-Venereology (M Tampa PhD), Dr. Victor Babes Clinical Hospital of Infectious Diseases and Tropical Diseases, Bucharest, Romania; Zhujiang Hospital (J Tan MD, Z Zhu PhD), Southern Medical University, Guangzhou, China; The First Affiliated Hospital of Chongqing Medical University (J Tan MD), Western Institute of Digital-Intelligent Medicine (Z Xia MD), Chongqing Medical University, Chongqing, China; Department of Morphophysiology (B Tanabayev PhD), South Kazakhstan Medical Academy, Shymkent, Kazakhstan; American Heart Association Comprehensive Hypertension Center (E Tantisattamo MD), University of California Irvine, Orange, CA, USA; Excellence Center for Organ Transplantation (E Tantisattamo MD), Mahidol University, Bangkok, Thailand; Department of Software Engineering (M Tanveer PhD), Foundation University Islamabad, Islamabad, Pakistan; AI Developer (M Tanveer PhD), National University of Science and Technology, Islamabad, Pakistan; Department of Public Health and Informatics (A Tasnim MPH), Bangladesh Medical University, Dhaka, Bangladesh; Taking Our Best Shot, Houston, TX, USA (N Y Tat MS); Department of Research and Innovation (N Y Tat MS), Enventure Medical Innovation, Houston, TX, USA; Department of Pathology (V Y Tat BS), University of Texas, Galveston, TX, USA; College of Health Sciences (W Tesfamariam MSc), Mekelle University, Mekelle, Ethiopia; Amrita Vishwa Vidyapeetham, Kochi, India (Prof K R Thankappan MD); Department of Economics (I Tharwat PhD), The American University in Cairo, Cairo, Egypt; Faculty of Medicine (R Thayakaran PhD), University of Southampton, Southampton, England; Department of Applied Bioscience (Prof M Thiruvengadam PhD), Konkuk University, Seoul, South Korea; Faculty of Public Health (J H V Ticoalu MPH), Universitas Sam Ratulangi (Sam Ratulangi University), Manado, Indonesia; K.A. Timiryazev Institute of Plant Physiology (M V Titova PhD), Russian Academy of Sciences, Moscow, Russia; College of Nursing (S I Tonapa PhD), Kaohsiung Medical University, Taiwan, Kaohsiung, Taiwan; Interdisciplinary Health Data Center (R Topor-Madry PhD), Jagiellonian University Medical College, Kraków, Poland; James Cook University, Douglas, QLD, Australia (S M Topp PhD); Centre for Infectious Disease Research in Zambia (CIDRZ), Lusaka, Zambia (S M Topp PhD); Second Department of Internal Medicine (Q T H Tran PhD), Kansai Medical University, Hirakata, Japan; Department of Internal Medicine (T H Tran MD), University of Medicine and Pharmacy at Ho Chi Minh City, Ho Chi Minh City, Viet Nam; Department of Business Analytics (T H Tran MD), University of Massachusetts Dartmouth, Dartmouth, MA, USA; Research and Advocacy Initiative (N Tran Minh Duc MD), ALS Vietnam, Quang Ngai, Viet Nam; College of Health Science (Q X N Truong PhD),

Vin University, Hanoi, Viet Nam; Tianjin Key Laboratory of Ionic-Molecular Function of Cardiovascular Disease (Prof G Tse PhD), Second Hospital of Tianjin Medical University, Tianjin, China; School of Life Course and Population Sciences (Prof Y Wang PhD), King's College London, London, England (Prof L Tudor Car PhD); Department of Biological Sciences (R Ullah PhD), National University of Medical Sciences (NUMS), Rawalpindi/ Islamabad, Pakistan; International Center for Chemical and Biological Sciences (S Ullah MSc), University of Karachi, Karachi, Pakistan; Department of Oncology (S S Umar FWACS), Federal Medical Centre, Gusau, Nigeria; Department of Physiotherapy (J S Usman PhD), Bayero University, Kano, Nigeria; Department of Rehabilitation Sciences (J S Usman PhD), Hong Kong Polytechnic University, Hong Kong, China; Department of Orthodontics (H Uzunçıbuk PhD), University of Trakya, Edirne, Türkiye; Sociedad Argentina de Medicina, Buenos Aires, Argentina (Prof P R Valdez PhD); Hospital Vélez Sarsfield, Buenos Aires, Argentina (Prof P R Valdez PhD); Institute for Global Brain Health (Prof S Vanneste PhD), Trinity College Dublin, Dublin, Ireland; Department of Nephrology (Prof S Varughese FRCP), Christian Medical College and Hospital (CMC), Vellore, India; UKK Institute, Tampere, Finland (Prof T J Vasankari PhD); Faculty of Medicine and Health Technology (Prof T J Vasankari PhD), Tampere University, Tampere, Finland; School of Epidemioloy (A Velayudhan MPH), Indian Institute of Health Management Research University, Jaipur, India; Department of Human Genetics & Molecular Biology (Prof B Vellingiri PhD), Bharathiar University, Coimbatore, India; Department of Community Medicine and Family Medicine (U Venkatesh MD), All India Institute of Medical Sciences, Gorakhpur, India; Raffles Neuroscience Centre (Prof N Venketasubramanian MSc), Raffles Hospital, Singapore, Singapore; Digital Health Research Center (D Villarreal-Zegarra MPH), Digital Health S.A., Lima, Peru; Faculty of Medicine of Itajubá -FMIT, Itajubá, Brazil (Prof L M Vitorino PhD); Universidade do Vale do Sapucaí - UNIVÁS (Prof L M Vitorino PhD), UNIVÁS, Pouso Alegre, Brazil; Department of Health Care Administration and Economics (Prof V Vlassov MD), National Research University Higher School of Economics, Moscow, Russia; Institute of Family Medicine and Public Health (A Voit MSc), University of Tartu, Tartu, Estonia; Heidelberg Institute of Global Health, Section for Oral Health (A Voit MSc), Heidelberg University Hospital, Heidelberg, Germany; Department of Medical Oncology (S Volovat PhD), University of Medicine and Pharmacy "Grigore T Popa" Iasi, Iaşi, Romania; Department of Medical Oncology (S Volovat PhD), Regional Institute of Oncology, Iaşi, Romania; Egyetem Square 1 (Prof Y Waheed PhD), Széchenyi István University, H-9026 Győr, Hungary; School of Medicine (E L Wan MPH), Georgetown University, Arlington, VA, USA; Stomatological Hospital (H Wang MD), Military Medical University, Xi'an, China; Department of Artificial Intelligence (S Wang PhD), Xiamen University, Xiamen, China; Department of Neurosurgery (S Wang MD), Beijing Tiantan Hospital, Beijing, China; Department of Neurosurgery (S Wang MD), Capital Medical University, Beijing, China; School of Public Health (Prof W Wang PhD), Department of Epidemiology (D Yin DrPH), Xuzhou Medical University, Xuzhou, China; Enze Medical Health Academy (X Wang PhD), Taizhou Hospital of Zhejiang Province, Taizhou, China; School of Public Health (Y Wang MPH), Zhengzhou University, Zhengzhou, China; School of Society and Culture (Prof P R Ward PhD), Adelaide University, Adelaide, SA, Australia; Department of Nursing (A Wilandika PhD), Universitas Aisyiyah Bandung, Bandung, Indonesia; Legon Centre for Education Research and Policy (M Wiredu Agyekum PhD), University of Ghana, Accra, Ghana; Institute of Health and Care Sciences (Prof A W Wolf PhD), University of Gothenburg, Gothenburg, Sweden; Faculty of Health Sciences (Prof A W Wolf PhD), Oslo Metropolitan University, Oslo, Norway; Department of Intelligent Medical Engineering (Prof W Xie DrPH), Anhui Medical University, Anhui, China; Department of Surgery (Prof W Xie DrPH), The First Affiliated Hospital of Anhui Medical University, Hefei, Anhui, China; Department of Occupational Diseases (H Yang MD), Linyi People's Hospital, Linyi, China; Department of

General Medicine (Y Yano MD), Department of Public Health (Prof N Yonemoto PhD), Juntendo University, Tokyo, Japan; Institute of Science Tokyo, Tokyo, Japan (Y Yano MD); College of Pharmacy and Health Science (H K A Yasin PhD), Other, Ajman, United Arab Emirates; Division of Public Health (Y Yasufuku PhD), The University of Osaka, Suita, Japan; SDU University, Astana, Kazakhstan (K A Yergaliyev DrPH); Department of Family Medicine (S A Yesuf MSc), St. Paul's Hospital Millennium Medical College, Addis Ababa, Ethiopia; Family Medicine Department (S A Yesuf MSc), St. Peter's Specialized Hospital, Addis Ababa, Ethiopia; Department of Respiratory Medicine (X Yi MD), Military Medical University, Chongqing, China; Nankai University (Y Yin PhD), Other, Tianjin, China; Department of Pediatrics (Prof D Yon MD), Kyung Hee University, Seoul, South Korea; Department of Biostatistics (Prof N Yonemoto PhD), University of Toyama, Toyama, Japan; Department of Health Policy and Management (Prof M Younis PhD), Jackson State University, Jackson, MS, USA; School of Business & Economics (Prof M Younis PhD), Tsinghua University, Beijing, China; School of Public Health (H Yu PhD), University of Alabama, Birmingham, AL, USA; School of Public Health (Prof Y Yu MS), Hubei University of Medicine, Shiyan, China; Department of Breast Surgery (Q Yuan MD), Harbin Medical University Cancer Hospital, Harbin, China; Association for Socially Applicable Research (ASAR), Pune, India (S Zadey MS); Department of Emergency Medicine (S Zadey MS), Global Emergency Medicine Innovation and Implementation (GEMINI) Research Center, Durham, NC, USA; Islamic Azad University, Tehran, Iran (M Zaghampour MD); Department of Oncology and Mammology (D Zairov MD), Kazakh Russian Medical University, Almaty, Kazakhstan; Health Investigation Center (N Zamora MD), Universidad Católica Boliviana San Pablo, Tarija, Bolivia; Nursing Care Research Center in Chronic Diseases (Prof K Zarea PhD), Ahvaz Jundishapur University of Medical Sciences, Ahvaz, Iran; Department of Environmental Health Engineering (A Zarei PhD), Gonabad University of Medical Sciences, Gonabad, Iran; Department of Administration (Prof M Zastrozhin PhD), PGxAI, San Francisco, CA, USA; Mohammed VI International School of Public Health (Prof S Zbiri PhD), Mohammed VI University of Sciences and Health (UM6SS), Casablanca, Morocco; Institute for Healthcare System Analysis (IA2S), Paris, France (Prof S Zbiri PhD); Faculdade de Medicina da Universidade de Sao Paulo (J M F Zhang MD), University of Sao Paulo, Sao Paulo, Brazil; School of Pharmacy (C Zhao PhD), Queen's University Belfast, Belfast, UK; School of Pharmacy (C Zhao PhD), China Medical University, Shenyang, China; National Institute of Parasitic Diseases (J Zheng PhD), Chinese Center for Disease Control and Prevention, Shanghai, China; Department of Surgery (H Zhou MD), Zhejiang University, yiwu, Zhejiang province,China, China; School of Public Health and Emergency Management (B Zhu PhD), Southern University of Science and Technology, Shenzhen, China; NIHR-Biomedical Research Centre (NIHR-BRC) (Prof A Zumla PhD), University College London Hospitals, London, England; Clinical Research Centre (Prof S H Zyoud PhD), An-Najah National University Hospital, Nablus, Palestine; Department of Building Engineering and Environment (S H Zyoud PhD), Civil Engineering and Sustainable Structures (S H Zyoud PhD), Palestine Technical University (Kadoorie).

Authors' Contributions

Managing the overall research enterprise

Annie Haakenstad, Kelly Bienhoff, Rafael Lozano, Enis Baris, Christopher J L Murray

Writing the first draft of the manuscript

Megan Knight

Primary responsibility for applying analytical methods to produce estimates

Megan Knight, Annie Haakenstad

Primary responsibility for seeking, cataloguing, extracting, or cleaning data; designing or coding figures and tables

Megan Knight

Providing data or critical feedback on data sources

Bhoomadevi A, Auwal Abdullahi, Armita Abedi, E S Abhilash, Richard Gyan Aboagye, Abdullahi Tunde Aborode, Lucas Guimarães Abreu, Michael R M Abrigo, Bilyaminu Abubakar, Hana J Abukhadijah, Anirudh Balakrishna Acharya, Krishna Prasad Acharya, Swetha Acharya, Olusegun Adetomiwa Adediran, Kamoru Ademola Adedokun, Oluwatobi E Adegbile, Olumide Thomas Adeleke, Ridwan Olamilekan Adesola, Muhammad Sohail Afzal, Saira Afzal, Feleke Doyore Doyore Agide, Williams Agyemang-Duah, Adamu Adamu Ahmad, Danish Ahmad, Muayyad M Ahmad, Rabbiya Ahmad, Sajjad Ahmad, Suleiman Idris Ahmad, Ali Ahmadi, Asma Ahmed, Ayman Ahmed, Jahanzaib Mian Ahmed, Khabab Abbasher Hussien Mohamed Ahmed, Mehrunnisha Sharif Ahmed, Muktar Beshir Ahmed, Mushood Ahmed, Muhammad Nadeem Akhtar, Wole Akosile, Rasmieh Mustafa Al-Amer, Montaha Al-Iede, Samah W Al-Jabi, Sabah Al-Marwani, Ali Salman Al-Shami, Alaa B Al-Tammemi, Fares Alahdab, Mohammad Jahoor Alam, Fahad Mashhour Alanezi, Turki M Alanzi, Mohammed Albashtawy, Robert W Aldridge, Ali M Alfalki, Abdelazeem M Algammal, Ashraf Alhumaidi, Liaqat Ali, Syed Shujait Ali, Morteza Alipour, Syed Mohamed Aljunid, Wesam Taher Almagharbeh, Joseph Uy Almazan, Mohmmad Minwer Alnaeem, Khaldoon Aied Alnawafleh, Intima Alrimawi, Mamdouh Alshammari, Abdalkarem Fedgash Alsharari, Nelson Alvis-Guzman, McKing Izeiza Amedari, Faten Amer, Amr Amin, Saeed Amini, Majid Aminzare, Ganiyu Adeniyi Amusa, Pedro Prata Andrade, Song Peng Ang, Nguyen Hoang Anh, Samuel Egyakwa Ankomah, Kabilan Annadurai, Saeid Anvari, Sumadi Lukman Anwar, Razique Anwer, Rabie Adel El Arab, Jalal Arabloo, Jesu Arockiaraj, Mahwish Arooj, Anton A Artamonov, Syed Mohammed Basheeruddin Asdaq, Nestor Asiamah, Muhammad Shahzad Aslam, Anil Raj Assariparambil, Batyrbek Assembekov, Seyyed Shamsadin Athari, Prince Atorkey, Alok Atreya, Avinash Aujayeb, Nyan Min Aung, Sana Javaid Awan, Kosar Hikmat Hama Aziz, Mohd Yusmaidie Aziz, Ahmed Y Azzam, Syed Babar Jamal Bacha, Alaa Aboelnour Badran, Ruhai Bai, Atif Amin Baig, Abdulaziz T Bako, Pachamuthu Balakrishnan, Senthilkumar Balakrishnan, Ahmed Omar Bali, Ovidiu Constantin Baltatu, Biswajit Banik, Hiba Jawdat Barqawi, Cornelia Anne Barth, Mohammad-Mahdi Bastan, Abdul-Monim Batiha, Mulat Tirfie Bayih, Zemenu Wube Bayleyegn, Narasimha M Beeraka, Aminu K Bello, Kelemu Zelalem Berhanu, Robert S Bernstein, Nikha Bhardwaj, Sonu Bhaskar, Priyadarshini Bhattacharjee, Gurjit Kaur Bhatti, Jasvinder Singh Bhatti, Zulfiqar A Bhutta, Sibhatu Kassa Biadgilign, Bijit Biswas, Fassikaw Kebede Bizuneh, Lucimere Bohn, Souad Bouaoud, Dejana Braithwaite, Jefferson Antonio Antonio Buendia Buendia, Felix Busch, Jack Cagney, Mate Car, Qinghua Chang, Vijay Kumar Chattu, Lam Duc Chau, Sirshendu Chaudhuri, Akhilanand Chaurasia, Haowei Chen, Bryan Chong, Hitesh Chopra, Shivani Chopra, Dinh-Toi Chu, Jinky Marie T. Chua, Sunghyun Chung, Fred Cohen, Joao Conde, Natalia Cruz-Martins, Xiaochen Dai, Maxwell Ayindenaba Dalaba, Mayank Dalakoti, Anh Kim Dang, Samuel Demissie Darcho, Ali Hasanpour- Dehkordi,

Ivan Delgado-Enciso, Ismail Dergaa, Nikolaos Dervenis, Vinoth Gnana Chellaiyan Devanbu, Thakur Dhakal, Sreedhar Dharmagadda, Huyen Phuc Do, Mai Ngoc Do, Zahra Abbasi Dolatabadi, Fariba Dorostkar, Ojas Prakashbhai Doshi, Robert Kokou Dowou, Senbagam Duraisamy, Anar Dushpanova, Afework Edmealem, Michael Ekholuenetale, Yasir Ahmed Mohammed Elhadi, Samar Abd ElHafeez, Salem Elkahoui, Chadi Eltaha, Majid Eslami, Fahima Nasrin Eva, Adewale Oluwaseun Fadaka, Heidar Fadavian, Adeniyi Francis Fagbamigbe, Ayesha Fahim, Mohammad Fareed, Aisha Farhana, Fatemeh Farshad, Davood Fathi, Ginenus Fekadu, Marta Figueiredo, Safia Firdous, Artem Alekseevich Fomenkov, Arianna Fornari, Takeshi Fukumoto, Nancy Fullman, Peter Andras Gaal, Muktar A Gadanya, Balasubramanian Ganesh, Ezhilkugan Ganessane, Fernando Jr. Barroga Garcia, Chandi Garg, Tsion S Gebre, Julian Lassiter Gendreau, Gebremariam Wulie Geremew, Kalab Yigermal Gete, Mustafa Ghaderzadeh, Kazem Ghaffari, Arin Ghamkhar, Ahmad Ghashghaee, Sailaja Ghimire, Vaitsa Giannouli, Syed Abdullah Gilani, Alem Abera Girmay, Mahaveer Golechha, Pouya Goleij, Davide Golinelli, Yitayal Ayalew Goshu, Ayman Grada, Damitha Asanga Gunawardane, Lei Guo, Aayush Gupta, Bhawna Gupta, Sapna Gupta, Vijai Kumar Gupta, Annie Haakenstad, Khalid Rehman Rehman Hakeem, Alex Hakuzimana, Pritam Halder, Ayman M. Hamdan-Mansour, Mohamed Hamed, Asif Hanif, Josep Maria Haro, Mohammad Jahid Hasan, Towhid Hasan, Ahmad Shoeb Hashmi, Simon I Hay, Mohammad Heidari, Reza Mosaddeghi Heris, Thomas Kwadwo Hinneh, Yu Hong, Mehdi Hoseinzadeh, Md Mahbub Hossain, Mohammad Bellal Hossain, Yongsong Huang, Aminu Alhassan Ibrahim, Jibran Ikram, Olayinka Stephen Ilesanmi, Meesha Iqbal, Usman Iqbal, Lalu Muhammad Irham, Md Sahidul Islam, Md. Shahinul Islam, Nahlah Elkudssiah Ismail, Gaetano Isola, Yussif Issaka, Jaison Jacob, Udeme Samuel Jacob, Haitham Jahrami, Mihajlo Jakovljevic, Armaan Jamal, Jerin James, Rajiv Janardhanan, Krishna Mahendrabhai Jasani, Sathish Kumar Jayapal, Shubha Jayaram, Yovanthi Anurangi Jayasinghe, Jae Joon Jeon, Akaninyene Paul Joseph, Charity Ehimwenma Joshua, Farahnaz Joukar, Jacek Jerzy Jozwiak, Sang-Hyuk Jung, Malik E Juweid, Hema Shree K, Dler Hussein Kadir, Adel Kadri, Hossein Samadi Kafil, Ashish Kumar Kakkar, Khalil Kalavani, Feroze Kaliyadan, Ramat T. Kamorudeen, Hesam Kamyab, Jiseung Kang, Rami S Kantar, Mohmed Isaqali Karobari, Faizan Zaffar Kashoo, Nehemia I. Kassa, Nicholas J Kassebaum, Kanica Kaushal, Foad Kazemi, Aïmen Khacharem, Yousef Saleh Khader, Himanshu Khajuria, Anees Ahmed Khalil, Ajmal Khan, Faiz Ullah Khan, Iqra Hamid Khan, MD Sazzad Khan, Mohd Faheem Khan, Muhammad Arslan Khan, Muhammad Umer Khan, Ramsha Mushtaq Khan, Zahid Khan, Sameer Uttamaro Khasbage, Khaled Khatab, Bishal Dahal Khatri, Khalid A Kheirallah, Parisa Khoshvaght, Jinho Kim, Adnan Kisa, Michail Kokkorakis, Shivakumar KM Kondlahalli, Tapos Kormoker, Oleksii Korzh, Irene Akwo Kretchy, Kewal Krishan, Shikha Kukreti, Chandan Kumar, Dewesh Kumar, Kamal Kumar, Laksh Kumar, Manasi Kumar, Nitesh Kumar, Pradeep Kumar, Sandeep Kumar, Sanjeet Kumar, Vijay Kumar, Satyajit Kundu, Donata Kurpas, Christina Yeni Kustanti, Dian Kusuma, Assylkhan Kuttybayev, Ville Kytö, Muhammad Awwal Ladan, Desireé Alexa LaGrappe, Chandrakant Lahariya, Dharmesh Kumar Lal, Anders O Larsson, Savita Lasrado, Areeba Latif, Sindhura Lakshmi Koulmane Laxminarayana, Caterina Ledda, Munjae Lee, Sergey Vadimovich Lee, Seung Won Lee, Wei-Chen Lee, Vasileios Leivaditis, Honghong Li, Virendra S Ligade, Amr Selim Abu Lila, Jue Liu, Niroshan Chandrajith Lokunarangoda, Jaifred Christian F Lopez, Miltiadis D Lytras, Ellina Lytvyak, Kevin Sheng-Kai Ma, Monika Machoy-Rakoczy, Samatar Abshir Mahamed, Nozad Hussein Mahmood, Kafayat Mahmoud, Ghaseb Naser Makhadmeh, Ahmad Azam Malik, J Jenifer Florence Mary, Roy Rillera Marzo, Sammer Marzouk, Suleiman Mayaki, Maryam Mazaheri, Steven M McPhail, Vini Mehta, Godfred Antony Menezes, Emiru Ayalew Mengistie, Leweyehu Alemaw Mengstie, Sandrine Donfack D Mewoabi, Giuseppe Minervini, Wai-kit Ming, Erkin M Mirrakhimov, Chaitanya Mittal, Mona Gamal Mohamed, Nouh Saad Mohamed, Shafiu Mohammed,

Yugal Kishore Mohanta, Siba Sankar Mohanty, Ali H Mokdad, TJ Robinson T Moncatar, Mohammad Moradi-Joo, Freiser Eceomo Cruz Mosquera, Rabia Mubarak, Sumaira Mubarik, Jibran Sualeh Muhammad, Amartya Mukhopadhyay, JOSHUA KANAABI MULIIRA, Francesk Mulita, Christopher J L Murray, David Musoke, Ghulam Mustafa, Lutfi Muzaqi, Gopal Nambi, Mahmoud Nassar, Samidi Nirasha Kumari Navaratna, Biswa Prakash Nayak, Kinjal Nayak, Vinod C Nayak, Javad Nazari, Mojdeh Nazari, Ionut Negoi, Ruxandra Irina Negoi, Cuong Tat Nguyen, Hien Thu Nguyen, Huong Lan Thi Nguyen, Tham Thi Nguyen, Trang Nguyen, Yeshambel T Nigatu, Shuhei Nomura, Mamoona Noreen, Emira Noumi, Fred Nugen, Bogdan Oancea, Olusegun Olatunji Ojedoyin, Olalekan John Okesanya, Osaretin Christabel Okonji, John Olayemi Okunlola, Andrew T Olagunju, Oladotun Victor Olalusi, Abdulhakeem Abayomi Olorukooba, Bolajoko Olubukunola Olusanya, Obinna E Onwujekwe, Aksoltan Shyhdurdyevna Oradova, Atakan Orscelik, Godwin Osawaru Osaigbovo, Godfred Otchere, Adrian Otoiu, Jerry John Ouner, Amel Ouyahia, Mayowa O Owolabi, Oladayo Ayobami Oyebanji, Kehinde Adewole Oyeniran, Dilber Uzun Ozsahin, Jagadish Rao Padubidri, Vijayakumar Palaniswamy, Adrian Pana, Sujogya Kumar Panda, Bipindra Pandey, Santa Maria Pangaribuan, Shahina Pardhan, Romil R Parikh, Chulwoo Park, Arpit Parmar, Maja Pasovic, Mitesh Patel, Neel Navinkumar Patel, Shankargouda Patil, Apurba Patra, Uttam Paudel, Shrikant Pawar, Prince Peprah, Mario F P Peres, Olumuyiwa James Peter, Zahra Zahid Piracha, Sajjad Pourasghary, Mohsen Poursadeqiyan, Elton Junio Sady Prates, Harsh Priya, Marcello Di Pumpo, Jagadeesh Puvvula, Nameer Hashim Qasim, Xiang Qi, Navid Rabiee, Venkatraman Radhakrishnan, Pracheth Raghuveer, Sajjad Rahimi, Afarin Rahimi-Movaghar, Vafa Rahimi-Movaghar, Firman Suryadi Rahman, Adarsh Raja, Judah Rajendran, Kairolla Dyusenbayevich Rakhimov, Rafał Rakoczy, Shakthi Kumaran Ramasamy, Diana Fernanda Bejarano Ramirez, Lokesh Rana, Nemanja Rancic, Manjula Rangappa, Magesh Rangasamy, Chythra R Rao, Kumuda Rao, Mithun Rao, Sowmya J Rao, Ramin Ravangard, Rashita Ravi, Renju Ravi, David Laith Rawaf, Salman Rawaf, Lal Rawal, Ayita Ray, Bhagwan Narayan Rekadwad, Serge Resnikoff, Stefano Restaino, Luis Felipe Reyes, Nima Rezaei, Muhammad Riaz, Syed Mohd Danish Rizvi, Leonardo Roever, Hamid Alinejad Rokny, Moustaq Karim Khan Rony, Kevin T Root, Sharmistha Roy, Godfrey Mutashambara Rwegerera, Aly M A Saad, Korosh Saber, Siamak Sabour, Bashdar Abuzed Sadee, Hossein Sadr, Umar Saeed, Maryam Saeedi, Rajesh Sagar, Vaibhav Sahni, Ambika Sahoo, Kirti Sundar Sahu, S Mohammad Sajadi, Mirza Rizwan Sajid, Joseph W Sakshaug, Afeez Abolarinwa Salami, Samreen Saleem, Giovanni A Salum, Abdallah M Samy, Elaheh Sanjari, Sathish Sankar, Milena M Santric-Milicevic, Malik Adebayo Sanusi, Sivan Yegnanarayana Iyer Saraswathy, Mohammad Sarmadi, Maheswar Satpathy, Amar Saurabh, Sangeeta Gopal Saxena, Yaser Sayadi, Ahmed M. Sayed, Christophe Schinckus, Durairaj Sekar, Chandrabose Selvaraj, Siddharthan Selvaraj, Fikadu Waltengus Sendeku, Subramanian Senthilkumaran, Edson Serván-Mori, Francesco Sessa, Yashendra Sethi, Shipraa Arpan Shah, Sweni Shah, Muhammad Shahab, Samiah Shahid, Endrit Shahini, Ali Shahriari, Babar Tasneem Shaikh, Masood Ali Shaikh, Alfiya Shamsutdinova, Dan Shan, Nadim Sharif, Fatemeh Saheb Sharif-Askari, Narjes Saheb Sharif-Askari, Amin Sharifan, Avimanu Sharma, Bhoopesh Kumar Sharma, Jyoti Sharma, Kamal Sharma, Kamlesh Sharma, Vishal Sharma, Ramzi Shawahna, Ali Sheidaei, Samendra P Sherchan, Mahabalesh Shetty, Premalatha K Shetty, Md Monir Hossain Shimul, SHIRINDOKHT SHIRAZI, Aminu Shittu, Km Shivangi, Azad Shokri, Sunil Shrestha, Suleiman Adeiza Shuaibu, Negussie Boti Sidamo, Arif J Siddiqui, Padam Prasad Simkhada, David Larbi Simpong, Abhinav Singh, Baljinder Singh, Bhoopendra Singh, Brij Pal Singh, Harmanjit Singh, Jasvinder A Singh, Jawahar Singh, Shilpy Singh, Siddharth Singh, Mukesh Kumar Sinha, Ratnesh Sinha, Valentin Yurievich Skryabin, Mejdi Snoussi, Farrukh Sobia, César Agostinis Sobrinho, Solikhah Solikhah, Michael Spartalis, Chandrashekhar T Sreeramareddy, Shyamkumar Sriram, Anca Pantea Stoian, Brendon Stubbs, Chen-

Yang Su, Vetriselvan Subramaniyan, Mahwish Suhaib, Yan Sun, Thanigaivel Sundaram, Chandan Kumar Swain, Shreya P Swami, Lukasz Szarpak, Rafael Tabarés-Seisdedos, Shima Tabatabai, Ramin Tabibi, Jabeen Taiba, Mircea Tampa, Jianye Tan, Ker-Kan Tan, Shenglan Tang, Ekamol Tantisattamo, Mohsan Tanveer, Anika Tasnim, Aigul Yelgondiyevna Tazhiyeva, Yazan Al Thaher, Pugazhenthan Thangaraju, Ismaeel Tharwat, Jeevarathinam Thirumalai, Mariya Vladimirovna Titova, Vikas Kumar Tiwari, Madi Tleshev, Santo Imanuel Tonapa, Roman Topor-Madry, Stephanie M Topp, Marcos Roberto Tovani-Palone, Quynh Thuy Huong Tran, Domenico Trico, Gary Tse, Samiun Nazrin Bente Kamal Tune, Era Upadhyay, Hande Uzunçıbuk, Pascual R Valdez, Sven Vanneste, Joe Varghese, Tommi Juhani Vasankari, Anoop Velayudhan, Balachandar Vellingiri, Narayanaswamy Venketasubramanian, Nicollas Mozart Vieira, David Villarreal-Zegarra, Tuan Vinh, Luciano Magalhães Vitorino, Vasily Vlassov, Simona Ruxandra Volovat, Linh Vu, Yasir Waheed, Ethan Waisberg, Eric Lee Wan, Shu Wang, Wanzhou Wang, Angga Wilandika, Axel Walter Wolf, Sanni Yaya, Subah Abderehim Yesuf, Yulai Yin, Naohiro Yonemoto, Mustafa Younis, Hairui Yu, Yong Yu, Quan Yuan, Omar Ali Mohammed Al Zaabi, Arezou Hashem Zadeh, Siddhesh Zadey, Michael Zastrozhin, Mohammed G M Zeariya, Ruba A Zenati, Julio Min Fei Zhang, Yingxi Zhao, Jinxin Zheng, Anthony Zhong, Hao Zhou, Abzal Zhumagaliuly, Mohamed Ali Zoromba, Alimuddin Zumla, Ahed H Zyoud, Sa'ed H Zyoud, Shaher H Zyoud, Simon Øverland

Developing methods or computational machinery

Aleksandr Y Aravkin, Xiaochen Dai, Simon I Hay, Nicholas J Kassebaum, Ali H Mokdad, Christopher J L Murray, Maja Pasovic

Providing critical feedback on methods or results

Bhoomadevi A, Hazim S Ababneh, Madineh Abbasi, Mitra Abbasifard, Sherief Abd-Elsalam, Reda Abdel-Hameed, Atef Abdelkader, Deldar Morad Abdulah, Auwal Abdullahi, Meklit Girma Abebe, Tesfaye Birhanu Abebe, Armita Abedi, Asrat Agalu Abejew, E S Abhilash, Alemwork Abie, Richard Gyan Aboagye, Abdullahi Tunde Aborode, Nagah M. Abourashed, Mohamed Abouzid, Lucas Guimarães Abreu, Mauro Henrique Nogueira Guimarães de Abreu, Michael R M Abrigo, Dariush Abtahi, Rana Kamal Abu-Farha, Eman Abu-Gharbieh, Bilyaminu Abubakar, Ahmad Y Abuhelwa, Hana J Abukhadijah, Anirudh Balakrishna Acharya, Krishna Prasad Acharya, Swetha Acharya, Maryam Adabi, Nuhu Lawan Adamu, Isaac Yeboah Addo, Olusegun Adetomiwa Adediran, Kamoru Ademola Adedokun, Oluwatobi E Adegbile, Olumide Thomas Adeleke, Miracle Ayomikun Adesina, Ridwan Olamilekan Adesola, Ripon Kumar Adhikary, Prince Owusu Adoma, Aanuoluwapo Adeyimika Afolabi, Muhammad Sohail Afzal, Saira Afzal, Victor Ibukun Agbajelola, Feleke Doyore Doyore Agide, Percival Delali Agordoh, Prince Agwu, Martin Wiredu Agyekum, Williams Agyemang-Duah, Adamu Adamu Ahmad, Aqeel Ahmad, Danish Ahmad, Khabir Ahmad, Muayyad M Ahmad, Rabbiya Ahmad, Sajjad Ahmad, Suleiman Idris Ahmad, Ali Ahmadi, Asma Ahmed, Ayman Ahmed, Jahanzaib Mian Ahmed, Junaid Ahmed, Khabab Abbasher Hussien Mohamed Ahmed, Mehrunnisha Sharif Ahmed, Muktar Beshir Ahmed, Mushood Ahmed, Syed Anees Ahmed, Muhammad Nadeem Akhtar, Wole Akosile, Ziyad Al-Aly, Rasmieh Mustafa Al-Amer, Fahmi Y Al-Ashwal, Akram Al-Ibraheem, Montaha Al-Iede, Samah W Al-Jabi, Md. Al-Mamun, Sabah Al-Marwani, Ali Salman Al-Shami, Alaa B Al-Tammemi, Yaser Mohammed Al-Worafi, Timothy Olukunle Aladelusi, Fares Alahdab, Khurshid Alam, Mohammad Jahoor Alam, Mohammad Khursheed Alam, PRAVEJ ALAM, Fahad Mashhour Alanezi, Turki M Alanzi, Hussin Albargi, Mohammed Albashtawy, Khalifah A Aldawsari, Robert W Aldridge, Shereen M Aleidi, Bezawit Abeje Alemayehu, Ali M Alfalki, Abdelazeem M Algammal, Robert Kaba

Alhassan, Ashraf Alhumaidi, Irfan Ali, Kamran Ali, Liaqat Ali, Mohammad Daud Ali, Mohammed Usman Ali, Rafat Ali, Syed Shujait Ali, Esubalew Muluneh Aligaz, Cyrus Alinia, Morteza Alipour, Moath Saleh Aljohani, Syed Mohamed Aljunid, Wesam Taher Almagharbeh, Mohammed J. Almalki, Joseph Uy Almazan, Mohmmad Minwer Alnaeem, Khaldoon Aied Alnawafleh, Mahmoud A Alomari, Saleh A Alqahtani, Mohammad AlQudah, Intima Alrimawi, Mamdouh Alshammari, Abdalkarem Fedgash Alsharari, Malik A Althobiani, Nelson Alvis-Guzman, Mohammad Sharif Ibrahim Alyahya, Karem H Alzoubi, McKing Izeiza Amedari, Faten Amer, Amr Amin, Hawraz Ibrahim M. Amin, Saeed Amini, Majid Aminzare, Sohrab Amiri, Ganiyu Adeniyi Amusa, Jimoh Amzat, Pedro Prata Andrade, Song Peng Ang, Blake Angell, Nguyen Hoang Anh, Samuel Egyakwa Ankomah, Kabilan Annadurai, Boluwatife Stephen Anuoluwa, Iyadunni Adesola Anuoluwa, Saeid Anvari, Sumadi Lukman Anwar, Razique Anwer, Anayochukwu Edward Anyasodor, Rabie Adel El Arab, Jalal Arabloo, Mosab Arafat, Demelash Areda, Ghazal Arjmand, Jesu Arockiaraj, Mahwish Arooj, Anton A Artamonov, Nabiy Arystan, Syed Mohammed Basheeruddin Asdaq, Saeed Asgary, Nestor Asiamah, Muhammad Shahzad Aslam, Gizew Dessie Asres, Anil Raj Assariparambil, Batyrbek Assembekov, Seyyed Shamsadin Athari, Sulaiman Olusegun Atiku, Prince Atorkey, Alok Atreya, Avinash Aujayeb, Nyan Min Aung, Sana Javaid Awan, Kemal Lemnuro Awol, Camila Ospina Ayala, Martin Amogre Ayanore, Kosar Hikmat Hama Aziz, Mohd Yusmaidie Aziz, Ahmed Y Azzam, Zaharaddeen Shuaibu Babandi, Fatemeh Baberi, Abraham Samuel Babu, Syed Babar Jamal Bacha, Alaa Aboelnour Badran, Abdul Rasheed Bahar, Awsan Abdullah Saeed Bahattab, Razieh Bahreini, Ruhai Bai, Alpha Umaru Bai-Sesay, Atif Amin Baig, Lovenish Bains, Mohamad Amin Bakhshali, Abdulaziz T Bako, Pachamuthu Balakrishnan, Senthilkumar Balakrishnan, Wondu Feyisa Balcha, Ahmed Omar Bali, Ovidiu Constantin Baltatu, Biswajit Banik, Aleksandra Barac, Hiba Jawdat Barqawi, Zarrin Basharat, Mohammad-Mahdi Bastan, Abdul-Monim Batiha, Kavita Batra, Samuel Debas Bayable, Mulat Tirfie Bayih, Zemenu Wube Bayleyegn, Narasimha M Beeraka, Bekalu Mekonen Belay, Asnake Gashaw Belayneh, Aminu K Bello, Kelemu Zelalem Berhanu, Robert S Bernstein, Nikha Bhardwaj, Pankaj Bhardwaj, Sonu Bhaskar, Priyadarshini Bhattacharjee, Gurjit Kaur Bhatti, Jasvinder Singh Bhatti, Rajbir Bhatti, Zulfiqar A Bhutta, Sibhatu Kassa Biadgilign, Bijit Biswas, Mohammad Shahangir Biswas, Fassikaw Kebede Bizuneh, Micheal Kofi Boachie, Trupti Bodhare, Somayeh Bohlouli, Lucimere Bohn, Aime Bonny, Sudipta Bose, Souad Bouaoud, Dejana Braithwaite, Datonye Christopher Briggs, Jefferson Antonio Antonio Buendia Buendia, Ovidiu-Iulian Bunea, Felix Busch, Reinhard Busse, Yasser Bustanji, Zahid A Butt, Till Winfried Bärnighausen, Jack Cagney, Ismael Campos-Nonato, Si Cao, Yu Cao, Lorainne Tudor Car, Mate Car, Andre F Carvalho, Alberico L Catapano, Pamela Roxana Chacón-Uscamaita, Rama Mohan Chandika, Miyuru Chandradasa, Viswa Chaitanya Chandu, Jung-Chen Chang, Qinghua Chang, Vijay Kumar Chattu, Lam Duc Chau, Sirshendu Chaudhuri, Akhilanand Chaurasia, Fentahun Alemnew Chekole, Haowei Chen, Mingling Chen, Yun-jung Choi, Bryan Chong, Hitesh Chopra, Shivani Chopra, Sonali Gajanan Choudhari, Dinh-Toi Chu, Jinky Marie T. Chua, Isaac Sunday Chukwu, Sunghyun Chung, Arrigo Francesco Giuseppe Cicero, Fred Cohen, Joao Conde, Natalia Cruz-Martins, Rosario Cárdenas, Senthil Kumaran D, Mario D'Oria, Ali Dabbagh, Omid Dadras, Xiaochen Dai, Maxwell Ayindenaba Dalaba, Mayank Dalakoti, Payam Tabaee Damavandi, Anh Kim Dang, Samuel Demissie Darcho, Ali Hasanpour-Dehkordi, Ivan Delgado-Enciso, Endalkachew Dellie, Dessalegn Demeke, Ismail Dergaa, Nikolaos Dervenis, Muamer Dervišević, Aragaw Tesfaw Desale, Vinoth Gnana Chellaiyan Devanbu, Syed Masudur Rahman Dewan, Thakur Dhakal, Rajinder K Dhamija, Amol S Dhane, Sreedhar Dharmagadda, Delaney D Ding, Isaac Oluwafemi Dipeolu, Huyen Phuc Do, Mai Ngoc Do, Zahra Abbasi Dolatabadi, Fariba Dorostkar, Ojas Prakashbhai Doshi, Robert Kokou Dowou, Nguyen Tran Minh Duc, Emeka W. Dumbili, Senbagam Duraisamy, Oyewole Christopher Durojaiye, Ejemai Eboreime, Ikenna D Ebuenyi, Afework

Edmealem, Ferry Efendi, Abiodun Egwuenu, Michael Ekholuenetale, Mohamed Ahmed Eladl, Muhammed Elhadi, Yasir Ahmed Mohammed Elhadi, Samar Abd ElHafeez, Salem Elkahoui, Randa Elsheikh, Chadi Eltaha, Stanley Chinedu Eneh, Majid Eslami, Fahima Nasrin Eva, Adewale Oluwaseun Fadaka, Heidar Fadavian, Adeniyi Francis Fagbamigbe, Ayesha Fahim, Ildar Ravisovich Fakhradiyev, Mohammad Fareed, Aisha Farhana, Liliana Faria, Andre Faro, Fatemeh Farshad, Folorunso Oludayo Fasina, Davood Fathi, Ginenus Fekadu, Pietro Ferrara, Marta Figueiredo, Safia Firdous, Florian Fischer, Federica Fogacci, Artem Alekseevich Fomenkov, Arianna Fornari, Alina-Ioana Forray, Alberto Freitas, Takeshi Fukumoto, Nancy Fullman, Adam Fusheini, Peter Andras Gaal, Muktar A Gadanya, Anna D Gage, Márió Gajdács, Balasubramanian Ganesh, Ezhilkugan Ganessane, Fernando Jr. Barroga Garcia, Chandi Garg, Jacopo Garlasco, Rupesh K Gautam, Tsion S Gebre, Beza A Gelaw, Stefano Gelibter, Julian Lassiter Gendreau, Kabiru Ishola Genty, Gebremariam Wulie Geremew, Genanew Kassie Getahun, Habtamu Abebe Getahun, Kalab Yigermal Gete, Mustafa Ghaderzadeh, Kazem Ghaffari, Leila Ghalichi, Arin Ghamkhar, Ali Ghandili, Ahmad Ghashghaee, Sailaja Ghimire, Ali Gholamrezanezhad, Vaitsa Giannouli, Artyom Urievich Gil, Syed Abdullah Gilani, Bikash Ranjan Giri, Alem Abera Girmay, Mahaveer Golechha, Davide Golinelli, Melika Golmohammadi, Yitayal Ayalew Goshu, Barbara Niegia Garcia Goulart, Tulasi Govardhan, Ayman Grada, Stefano Guicciardi, Kabiru Abubakar Gulma, Damitha Asanga Gunawardane, Lei Guo, Aayush Gupta, Bhawna Gupta, Rajeev Gupta, Sapna Gupta, Vijai Kumar Gupta, Roberth Steven Gutiérrez-Murillo, Jose Guzman-Esquivel, Annie Haakenstad, Farrokh Habibzadeh, Khalid Rehman Rehman Hakeem, Alex Hakuzimana, Pritam Halder, Ayman M. Hamdan-Mansour, Mohamed Hamed, Samer Hamidi, Asif Hanif, Mohd Anul Haq, Mohammad Jahid Hasan, Towhid Hasan, Ahmad Shoeb Hashmi, Md Saquib Hasnain, Ikrama Hassan, Md. Imtaiyaz Hassan, Simon I Hay, Ahmad Hayatu, Mohammad Heidari, Reza Mosaddeghi Heris, Majid Heydari, Thomas Kwadwo Hinneh, Yuta Hiraike, Ramesh Holla, Yu Hong, Mehdi Hoseinzadeh, Lubna Hossain, Md Mahbub Hossain, Mohammad Bellal Hossain, Mihaela Hostiuc, Guoqing Hu, Yongsong Huang, Ayesha Humayun, Javid Hussain, Olayemi Joshua Ibidoja, Aminu Alhassan Ibrahim, Umar Idris Ibrahim, Aqsa Ikram, Jibran Ikram, Olayinka Stephen Ilesanmi, Irena M Ilic, Milena D Ilic, Mohammad Tarique Imam, Arit Inok, Meesha Iqbal, Mujahid Iqbal, Usman Iqbal, Lalu Muhammad Irham, Md Rabiul Islam, Md Sahidul Islam, Md. Shahinul Islam, Nahlah Elkudssiah Ismail, Gaetano Isola, Yussif Issaka, Masao Iwagami, Chidozie Declan Iwu, Jaison Jacob, Louis Jacob, Ali Jadidi, Nader Jahanmehr, Haitham Jahrami, Mihajlo Jakovljevic, Armaan Jamal, Anju James, Jerin James, Masoud Jamshidi, Rajiv Janardhanan, Mu'taman Jarrar, Krishna Mahendrabhai Jasani, Talha Jawaid, Sathish Kumar Jayapal, Shubha Jayaram, Yovanthi Anurangi Jayasinghe, Prof. Fariborz Sharifian Jazi, Jae Joon Jeon, Seogsong Jeong, Worku Jimma, Mohammad Jokar, Jobinse Jose, Akaninyene Paul Joseph, Nitin Joseph, Charity Ehimwenma Joshua, Farahnaz Joukar, Jacek Jerzy Jozwiak, Sang-Hyuk Jung, Malik E Juweid, Hema Shree K, Dler Hussein Kadir, Adel Kadri, Ashish Kumar Kakkar, Khalil Kalavani, Feroze Kaliyadan, Aidana Kaliyakparova, Rajesh Kamath, Ramat T. Kamorudeen, Hesam Kamyab, Jiseung Kang, Kehinde Kazeem Kanmodi, Rami S Kantar, Mehrdad Karajizadeh, Paschalis Karakasis, Jafar Karami, Marina Karanikolos, Mohmed Isaqali Karobari, Faizan Zaffar Kashoo, Nehemia I. Kassa, Nicholas J Kassebaum, Kanica Kaushal, Foad Kazemi, Aïmen Khacharem, Yousef Saleh Khader, Himanshu Khajuria, Asaad Khalid, Anees Ahmed Khalil, Ajmal Khan, Faiz Ullah Khan, Iqra Hamid Khan, MD Sazzad Khan, Mohd Faheem Khan, Muhammad Arslan Khan, Muhammad Umer Khan, Ramsha Mushtaq Khan, Sumaiya Khan, Yusuf Saleem Khan, Zahid Khan, Sameer Uttamaro Khasbage, Khaled Khatab, Haitham Khatatbeh, Moawiah Mohammad Khatatbeh, Bishal Dahal Khatri, Afshin Khazaei, Khalid A Kheirallah, Zahra Khorrami, Parisa Khoshvaght, Mahmood Khosrowjerdi, Jinho Kim, Kwanghyun Kim, Ruth W Kimokoti, Adnan Kisa, Sonali Kochhar, Prakash Babu Kodali, Michail Kokkorakis, Ali-Asghar Kolahi, Farzad

Kompani, Shivakumar KM Kondlahalli, Tapos Kormoker, Oleksii Korzh, Archana Koul, Arthi S Kozhumam, Irene Akwo Kretchy, Kewal Krishan, Mohammed Kuddus, Shikha Kukreti, Emmanuel Kumah, Chandan Kumar, Dewesh Kumar, Kamal Kumar, Laksh Kumar, Manasi Kumar, Mukesh Kumar, Nitesh Kumar, Pradeep Kumar, Sandeep Kumar, Sanjeet Kumar, Vijay Kumar, Satyajit Kundu, Jibin Kunjavara, Almagul Kurmanova, Donata Kurpas, Christina Yeni Kustanti, Dian Kusuma, Tezer Kutluk, Michael Agyemang Kwarteng, Ville Kytö, Muhammad Awwal Ladan, Desireé Alexa LaGrappe, Chandrakant Lahariya, Daphne Teck Ching Lai, Hanpeng Lai, Dharmesh Kumar Lal, Anders O Larsson, Savita Lasrado, Areeba Latif, Basira Kankia Lawal, Sindhura Lakshmi Koulmane Laxminarayana, Eilean Rathinasamy Lazarus, Caterina Ledda, Munjae Lee, Sergey Vadimovich Lee, Seung Won Lee, Wei-Chen Lee, Yo Han Lee, Vasileios Leivaditis, Francesco Leonforte, Chengfeng Li, Honghong Li, Ming-Chieh Li, Virendra S Ligade, Amr Selim Abu Lila, Jialing Lin, Jue Liu, Xianliang Liu, Madeeha Shahzad Lodhi, Niroshan Chandrajith Lokunarangoda, Jaifred Christian F Lopez, Arianna Maever Loreche, Masoud Lotfizadeh, Surbala Devi Lourembam, Giancarlo Lucchetti, Hengliang Lv, Miltiadis D Lytras, Ellina Lytvyak, Judit Lám, José Francisco López-Gil, Kevin Sheng-Kai Ma, Monika Machoy-Rakoczy, Samatar Abshir Mahamed, Nozad Hussein Mahmood, Hao Xuan Mai, Ghaseb Naser Makhadmeh, Ahmad Azam Malik, Marjan Mansourian, Tahir Maqbool, Adolfo Martinez-Valle, J Jenifer Florence Mary, Roy Rillera Marzo, Sammer Marzouk, Yasith Mathangasinghe, Manu Raj Mathur, Neeta Mathur, Fernanda Penido Matozinhos, Khurshid A Mattoo, Richard James Maude, Suleiman Mayaki, Martin McKee, Steven M McPhail, Vini Mehta, Tadesse Belayneh Melkie, Ziad Ahmed Memish, Godfred Antony Menezes, Emiru Ayalew Mengistie, Leweyehu Alemaw Mengstie, Atte Meretoja, Tuomo J Meretoja, Giovanni Merlino, Muayad Aghali Merza, Tomislav Mestrovic, Chamila Dinushi Kukulege Mettananda, Sachith Mettananda, Mohamed M. M. Metwally, Sandrine Donfack D Mewoabi, Giuseppe Minervini, Wai-kit Ming, GK Mini, Andreea Mirica, Erkin M Mirrakhimov, Vinaytosh Mishra, Chaitanya Mittal, Dhruti Modi, Hamed Markazi Moghadam, Sahar Saeedi Moghaddam, Mona Gamal Mohamed, Nouh Saad Mohamed, Taj Mohammad, Sakineh Mohammad-Alizadeh-Charandabi, Masoud Aman Mohammadi, Omer Mohammed, Shafiu Mohammed, Syam Mohan, Yugal Kishore Mohanta, Siba Sankar Mohanty, Ali H Mokdad, TJ Robinson T Moncatar, Himel Mondal, Mohammad Ali Moni, Marco Montalti, Mohammad Moradi-Joo, Danilo Farias Morais, Freiser Eceomo Cruz Mosquera, Elias Mossialos, Rabia Mubarak, Sumaira Mubarik, Jibran Sualeh Muhammad, Amartya Mukhopadhyay, JOSHUA KANAABI MULIIRA, Francesk Mulita, Kavita Munjal, Christopher J L Murray, David Musoke, Ghulam Mustafa, Mubarak Taiwo Mustapha, Sathish Muthu, Claude Mambo Muvunyi, Lutfi Muzaqi, Woojae Myung, Ayoub Nafei, Mobin Naghshbandi, Hiten Naik, Firzan Nainu, Hastyar Hama Rashid Najmuldeen, Gopal Nambi, Mukesh Kumar Sathya Narayanan, Abdulqadir J Nashwan, Mahmoud Nassar, Che Mohd Nasril Che Mohd Nassir, Samidi Nirasha Kumari Navaratna, Mohammad Khaled Al Nawayseh, Biswa Prakash Nayak, Kinjal Nayak, Vinod C Nayak, Javad Nazari, Mojdeh Nazari, Ionut Negoi, Ruxandra Irina Negoi, Chakib Nejjari, Nikita Nekliudov, Jan-Walter De Neve, Cuong Tat Nguyen, Hien Thu Nguyen, Huong Lan Thi Nguyen, Tham Thi Nguyen, Trang Nguyen, Yeshambel T Nigatu, Ali Nikoobar, Shuhei Nomura, Mamoona Noreen, Emira Noumi, Chisom Adaobi Nri-Ezedi, Fred Nugen, Bogdan Oancea, Akinyemi O D Ofakunrin, Oluwakemi Christie Ogidan, Ayomide Samuel Ogunrinde, Olusegun Olatunji Ojedoyin, Olalekan John Okesanya, Osaretin Christabel Okonji, John Olayemi Okunlola, Oluwaseyi Isaiah Olabisi, Musa Kolapo Oladejo, Andrew T Olagunju, Oladotun Victor Olalusi, Abimbola Olaniran, Gláucia Maria Moraes Oliveira, Abdulhakeem Abayomi Olorukooba, Bolajoko Olubukunola Olusanya, Oluwafemi G. Oluwole, Obinna E Onwujekwe, Aksoltan Shyhdurdyevna Oradova, Michal Ordak, Atakan Orscelik, Esteban Ortiz-Prado, Godwin Osawaru Osaigbovo, George Nkrumah Osei, Godfred Otchere, Adrian Otoiu, Jerry John Ouner, Amel Ouyahia, Mayowa O Owolabi, Oladayo Ayobami Oyebanji, Kehinde

Adewole Oyeniran, Dilber Uzun Ozsahin, Ilker Ozsahin, Jagadish Rao Padubidri, Vijayakumar Palaniswamy, Tamás Palicz, Adrian Pana, Sujogya Kumar Panda, Bipindra Pandey, Prateek Pandya, Santa Maria Pangaribuan, Ilias Papadimopoulos, Shahina Pardhan, Romil R Parikh, Chulwoo Park, Arpit Parmar, Roberto Passera, Mitesh Patel, Neel Navinkumar Patel, Shankargouda Patil, Dimitrios Patoulias, Apurba Patra, Uttam Paudel, Shrikant Pawar, Paolo Pedersini, Veincent Christian Filipino Pepito, Prince Peprah, Maria Odete Pereira, Mario F P Peres, Simone Perna, Olumuyiwa James Peter, Tung Thanh Pham, Michael R Phillips, Zahra Zahid Piracha, Mapa M Prabhath N Piyasena, Evgenii Plotnikov, Ion Popa, Pegah Salimi Pormehr, Fabio Porru, Sajjad Pourasghary, Mohsen Poursadeqiyan, Manya Prasad, Akila Prashant, Elton Junio Sady Prates, Harsh Priya, Nicola Riccardo Pugliese, Marcello Di Pumpo, Jagadeesh Puvvula, Muhammad Usman Qamar, Nameer Hashim Qasim, Xiang Qi, Navid Rabiee, Reza Rabiei, Venkatraman Radhakrishnan, Pracheth Raghuveer, Sajjad Rahimi, Afarin Rahimi-Movaghar, Vafa Rahimi-Movaghar, Firman Suryadi Rahman, Fryad Majeed Rahman, Mahbubur Rahman, Mosiur Rahman, Muhammad Aziz Rahman, Pramila Rai, Adarsh Raja, Judah Rajendran, Kairolla Dyusenbayevich Rakhimov, Rafał Rakoczy, Shakthi Kumaran Ramasamy, Pramod W Ramteke, Lokesh Rana, Nemanja Rancic, Manjula Rangappa, Magesh Rangasamy, Chythra R Rao, Kumuda Rao, Mithun Rao, Sowmya J Rao, Devarajan Rathish, Jigyasa Rathore, Ramin Ravangard, Rashita Ravi, Renju Ravi, David Laith Rawaf, Salman Rawaf, Lal Rawal, Ramu Rawat, Ayita Ray, Muhammad Liaquat Raza, Bahman Razi, C. Mahony Reategui-Rivera, Ramya Raja Reddy, Najeeb Ur Rehman, Lennart Reifels, Bhagwan Narayan Rekadwad, Serge Resnikoff, Stefano Restaino, Luis Felipe Reyes, Nazila Rezaei, Nima Rezaei, Mohsen Rezaeian, Muhammad Riaz, Jennifer Rickard, Syed Mohd Danish Rizvi, Leonardo Roever, Ravi Rohilla, Hamid Alinejad Rokny, Moustaq Karim Khan Rony, Kevin T Root, Himanshu Sekhar Rout, Adrija Roy, Gaurav Kumar Roy, Sharmistha Roy, Godfrey Mutashambara Rwegerera, Aly M A Saad, Korosh Saber, Siamak Sabour, Bashdar Abuzed Sadee, Fatemeh Sadeghi-Ghyassi, Hossein Sadr, Mohammad Reza Saeb, Umar Saeed, Maryam Saeedi, Muhammad Safiullah, Rajesh Sagar, Amene Saghazadeh, Indranil Saha, Vaibhav Sahni, Ambika Sahoo, Kirti Sundar Sahu, S Mohammad Sajadi, Mirza Rizwan Sajid, Joseph W Sakshaug, Afeez Abolarinwa Salami, Bukola Salami, Samreen Saleem, Mohamed A Saleh, Giovanni A Salum, Janmejaya Samal, Abdallah M Samy, Elaheh Sanjari, Sathish Sankar, Lucas H C C Santos, Milena M Santric-Milicevic, Malik Adebayo Sanusi, Sivan Yegnanarayana Iyer Saraswathy, Yaser Sarikhani, Hemen Sarma, Mohammad Sarmadi, Gargi Sachin Sarode, Sachin C Sarode, Michele Sassano, Maheswar Satpathy, Amar Saurabh, Sangeeta Gopal Saxena, Yaser Sayadi, Ahmed M. Sayed, Christophe Schinckus, Durairaj Sekar, Chandrabose Selvaraj, Siddharthan Selvaraj, Yuliya Semenova, Fikadu Waltengus Sendeku, Subramanian Senthilkumaran, Sadaf G Sepanlou, Edson Serván-Mori, Francesco Sessa, Yashendra Sethi, Abubakar Sha'aban, Shipraa Arpan Shah, Sweni Shah, Muhammad Shahab, Samiah Shahid, Wajeehah Shahid, Endrit Shahini, Farshad Shahkarami, Ali Shahriari, Babar Tasneem Shaikh, Masood Ali Shaikh, Alfiya Shamsutdinova, Dan Shan, Mohd Shanawaz, Nadim Sharif, Fatemeh Saheb Sharif-Askari, Narjes Saheb Sharif-Askari, Amin Sharifan, Avimanu Sharma, Bhoopesh Kumar Sharma, Jyoti Sharma, Kamal Sharma, Kamlesh Sharma, Vishal Sharma, Ramzi Shawahna, Somia Shehzadi, Ali Sheidaei, Suchitra M Shenoy, Samendra P Sherchan, Mahabalesh Shetty, Muhammad J. A. Shiddiky, Tariku Shimels, Md Monir Hossain Shimul, Shirindokht Shirazi, Aminu Shittu, Km Shivangi, Azad Shokri, Sina Shool, Seyed Afshin Shorofi, Sunil Shrestha, Suleiman Adeiza Shuaibu, Negussie Boti Sidamo, Emmanuel Edwar Siddig, Arif J Siddiqui, Gustavo Correia Basto da Silva, Padam Prasad Simkhada, David Larbi Simpong, Colin R Simpson, Abhinav Singh, Baljinder Singh, Bhoopendra Singh, Brij Pal Singh, Harmanjit Singh, Jasvinder A Singh, Jawahar Singh, Mayank Singh, Puneetpal Singh, Shilpy Singh, Siddharth Singh, Mukesh Kumar Sinha, Ratnesh Sinha, Natia Skhvitaridze, Valentin Yurievich Skryabin, Mejdi Snoussi, Farrukh Sobia, César Agostinis

Sobrinho, Md Salman Sohel, Solikhah Solikhah, Reza Darvishi Cheshmeh Soltani, Michael Spartalis, Lokasani Vijaya Sree, Chandrashekhar T Sreeramareddy, Shyamkumar Sriram, Anca Pantea Stoian, Brendon Stubbs, Peter Stubbs, Chen-Yang Su, Omer Subasi, Vetriselvan Subramaniyan, Aswin Sugunan, Mahwish Suhaib, Oksana Sulaieva, Sahabi K Sulaiman, Mark J M Sullman, Yan Sun, Zhong Sun, Thanigaivel Sundaram, David Sunkersing, Chandan Kumar Swain, Shreya P Swami, Lukasz Szarpak, Rafael Tabarés-Seisdedos, Shima Tabatabai, Ramin Tabibi, Mohd Tabish, Biniyam Beyene Tabor, Zanan Mohammed-Ameen Taha, Niloofar Taheri, Jabeen Taiba, Mircea Tampa, Jianye Tan, Ker-Kan Tan, Baimakhan Tanabayev, Dariga Tanabayeva, Shynar Tanabayeva, Shenglan Tang, Ekamol Tantisattamo, Mohsan Tanveer, Hongliang Tao, Anika Tasnim, Aigul Yelgondiyevna Tazhiyeva, Mohamad-Hani Temsah, Azimeraw Arega Tesfu, Yazan Al Thaher, Pugazhenthan Thangaraju, Kavumpurathu Raman Thankappan, Ismaeel Tharwat, Rasiah Thayakaran, Jeevarathinam Thirumalai, Muthu Thiruvengadam, Rekha Thiruvengadam, Jansje Henny Vera Ticoalu, Mariya Vladimirovna Titova, Vikas Kumar Tiwari, Madi Tleshev, Santo Imanuel Tonapa, Roman Topor-Madry, Stephanie M Topp, Marcos Roberto Tovani-Palone, Quynh Thuy Huong Tran, Domenico Trico, Quynh Xuan Nguyen Truong, Gary Tse, Vasilis-Spyridon Tseriotis, Evangelia Tsermpini, Samiun Nazrin Bente Kamal Tune, Atta Ullah, Riaz Ullah, Saeed Ullah, Shehu Salihu Umar, Era Upadhyay, Jibrin Sammani Usman, Hande Uzunçıbuk, Pascual R Valdez, Sven Vanneste, Joe Varghese, Anoop Velayudhan, Balachandar Vellingiri, U Venkatesh, Narayanaswamy Venketasubramanian, Nicollas Mozart Vieira, David Villarreal-Zegarra, Tuan Vinh, Luciano Magalhães Vitorino, Vasily Vlassov, Ave Voit, Simona Ruxandra Volovat, Linh Vu, Yasir Waheed, Ethan Waisberg, Eric Lee Wan, Jin-Yi Wan, Shaopan Wang, Shu Wang, Wanzhou Wang, Wei Wang, Xuequan Wang, Yan Wang, Yanzhong Wang, Youxin Wang, Mary Njeri Wanjau, Paul R Ward, Toyiba Hiyaru Wassie, Anggi Lukman Wicaksana, Nuwan Darshana Wickramasinghe, Samuel Wiebe, Angga Wilandika, Axel Walter Wolf, Zhijia Xia, Wanqing Xie, Saba Yahoo, Haibo Yang, Yuichiro Yano, Haiqiang Yao, Haya Khader Ahmad Yasin, Yuichi Yasufuku, Sanni Yaya, Reza Jalilzadeh Yengejeh, Kuanysh A. Yergaliyev, Subah Abderehim Yesuf, Saber Yezli, Xinglin Yi, Dehui Yin, Yulai Yin, Yazachew Engida Yismaw, Naohiro Yonemoto, Mustafa Younis, Kimia Mozahheb Yousefi, Hairui Yu, Yong Yu, Quan Yuan, Ghazala Yunus, Omar Ali Mohammed Al Zaabi, Arezou Hashem Zadeh, Siddhesh Zadey, Manijeh Zaghampour, Dilmurat Zairov, Nelson Zamora, Kourosh Zarea, Michael Zastrozhin, Saad Zbiri, Mohammed G M Zeariya, Zahra Zeinali, Ruba A Zenati, Eyael M Zeru, Julio Min Fei Zhang, Chenyu Zhao, Yingxi Zhao, Jinxin Zheng, Anthony Zhong, Hao Zhou, Bin Zhu, Zhaohua Zhu, Abzal Zhumagaliuly, Mohamed Ali Zoromba, Mohammad Ahmmad Mahmoud Al Zoubi, Tariq Al Zoubi, Alimuddin Zumla, Ahed H Zyoud, Sa'ed H Zyoud, Shaher H Zyoud, Simon Øverland, Simona Cătălina Ștefan

Drafting the work or revising it critically for important intellectual content

Bhoomadevi A, Hazim S Ababneh, Sherief Abd-Elsalam, Ashraf Nabiel Abdalla, Reda Abdel-Hameed, Atef Abdelkader, Auwal Abdullahi, Tesfaye Birhanu Abebe, Armita Abedi, Abdullahi Tunde Aborode, Nagah M. Abourashed, Mohamed Abouzid, Lucas Guimarães Abreu, Mauro Henrique Nogueira Guimarães de Abreu, Rana Kamal Abu-Farha, Eman Abu-Gharbieh, Bilyaminu Abubakar, Ahmad Y Abuhelwa, Hana J Abukhadijah, Anirudh Balakrishna Acharya, Swetha Acharya, Lisa C Adams, Nuhu Lawan Adamu, Isaac Yeboah Addo, Olusegun Adetomiwa Adediran, Kamoru Ademola Adedokun, Oluwatobi E Adegbile, Olumide Thomas Adeleke, Charles Oluwaseun Adetunji, Juliana Bunmi Adetunji, Atman Adiba, Prince Owusu Adoma, Aanuoluwapo Adeyimika Afolabi, Muhammad Sohail Afzal, Saira Afzal, Feleke Doyore Doyore Agide, Percival Delali Agordoh, Martin Wiredu Agyekum, Adamu Adamu Ahmad, Danish Ahmad,

Khabir Ahmad, Muayyad M Ahmad, Suleiman Idris Ahmad, Ali Ahmadi, Asma Ahmed, Ayman Ahmed, Jahanzaib Mian Ahmed, Junaid Ahmed, Khabab Abbasher Hussien Mohamed Ahmed, Mehrunnisha Sharif Ahmed, Muktar Beshir Ahmed, Mushood Ahmed, Syed Anees Ahmed, Marjan Ajami, Muhammad Nadeem Akhtar, Samina Akhtar, Wole Akosile, Rasmieh Mustafa Al-Amer, Fahmi Y Al-Ashwal, Ayman Al-Eyadhy, Akram Al-Ibraheem, Montaha Al-Iede, Samah W Al-Jabi, Md. Al-Mamun, Ali Salman Al-Shami, Alaa B Al-Tammemi, Yaser Mohammed Al-Worafi, Timothy Olukunle Aladelusi, Fares Alahdab, Khurshid Alam, Mohammad Jahoor Alam, Zufishan Alam, Hussin Albargi, Mohammed Albashtawy, Jacqueline Elizabeth Alcalde-Rabanal, Khalifah A Aldawsari, Mohammed S Aldossary, Robert W Aldridge, Abdelazeem M Algammal, Robert Kaba Alhassan, Ashraf Alhumaidi, Kamran Ali, Liaqat Ali, Mohammed Usman Ali, Syed Shujait Ali, Morteza Alipour, Moath Saleh Aljohani, Wesam Taher Almagharbeh, Mohmmad Minwer Alnaeem, Khaldoon Aied Alnawafleh, Mahmoud A Alomari, Saleh A Alqahtani, Intima Alrimawi, Sahel Majed Alrousan, Abdalkarem Fedgash Alsharari, Malik A Althobiani, Nelson Alvis-Guzman, Mohammad Sharif Ibrahim Alyahya, Karem H Alzoubi, Faten Amer, Amr Amin, Saeed Amini, Majid Aminzare, Sohrab Amiri, Ganiyu Adeniyi Amusa, Jimoh Amzat, Pedro Prata Andrade, Blake Angell, Samuel Egyakwa Ankomah, Kabilan Annadurai, Noureddin Nakhostin Ansari, Boluwatife Stephen Anuoluwa, Iyadunni Adesola Anuoluwa, Saeid Anvari, Anayochukwu Edward Anyasodor, Rabie Adel El Arab, Jalal Arabloo, Abdulfatai Aremu, Ghazal Arjmand, Jesu Arockiaraj, Mahwish Arooj, Syed Mohammed Basheeruddin Asdaq, Muhammad Shahzad Aslam, Gizew Dessie Asres, Anil Raj Assariparambil, Seyyed Shamsadin Athari, Sulaiman Olusegun Atiku, Ba-Etilayoo Atinga, Prince Atorkey, Alok Atreya, Avinash Aujayeb, Kemal Lemnuro Awol, Camila Ospina Ayala, Martin Amogre Ayanore, Kosar Hikmat Hama Aziz, Ahmed Y Azzam, Rasha Babiker, Abraham Samuel Babu, Syed Babar Jamal Bacha, Alaa Aboelnour Badran, Youngoh Bae, Abdul Rasheed Bahar, Awsan Abdullah Saeed Bahattab, Alpha Umaru Bai-Sesay, Atif Amin Baig, Lovenish Bains, Shankar M Bakkannavar, Abdulaziz T Bako, Pachamuthu Balakrishnan, Senthilkumar Balakrishnan, Ahmed Omar Bali, Ovidiu Constantin Baltatu, Biswajit Banik, Aleksandra Barac, Enis Baris, Hiba Jawdat Barqawi, Cornelia Anne Barth, Mohammad-Mahdi Bastan, Abdul-Monim Batiha, Zemenu Wube Bayleyegn, Narasimha M Beeraka, Aminu K Bello, Kelemu Zelalem Berhanu, Nikha Bhardwaj, Sonu Bhaskar, Priyadarshini Bhattacharjee, Gurjit Kaur Bhatti, Jasvinder Singh Bhatti, Sibhatu Kassa Biadgilign, Bijit Biswas, Mohammad Shahangir Biswas, Fassikaw Kebede Bizuneh, Somayeh Bohlouli, Lucimere Bohn, Aime Bonny, Sudipta Bose, Souad Bouaoud, Dejana Braithwaite, Datonye Christopher Briggs, Jefferson Antonio Antonio Buendia Buendia, Ovidiu-Iulian Bunea, Felix Busch, Yasser Bustanji, Till Winfried Bärnighausen, Jack Cagney, Ismael Campos-Nonato, Mate Car, Andre F Carvalho, Felix Carvalho, Alberico L Catapano, Pamela Roxana Chacón-Uscamaita, Rama Mohan Chandika, Miyuru Chandradasa, Viswa Chaitanya Chandu, Vijay Kumar Chattu, Lam Duc Chau, Anis Ahmad Chaudhary, Sirshendu Chaudhuri, Akhilanand Chaurasia, Fentahun Alemnew Chekole, Haowei Chen, Mingling Chen, Bryan Chong, Hitesh Chopra, Shivani Chopra, Jinky Marie T. Chua, Sunghyun Chung, Arrigo Francesco Giuseppe Cicero, Fred Cohen, Joao Conde, Claudia Cosma, Natalia Cruz-Martins, Senthil Kumaran D, Emanuele D'Amico, Mario D'Oria, Maxwell Ayindenaba Dalaba, Mayank Dalakoti, Payam Tabaee Damavandi, Anh Kim Dang, Samuel Demissie Darcho, Ali Hasanpour-Dehkordi, Ivan Delgado-Enciso, Ismail Dergaa, Nikolaos Dervenis, Muamer Dervišević, Vinoth Gnana Chellaiyan Devanbu, Syed Masudur Rahman Dewan, Thakur Dhakal, Amol S Dhane, Adriana Dima, Delaney D Ding, Isaac Oluwafemi Dipeolu, Huyen Phuc Do, Mai Ngoc Do, Zahra Abbasi Dolatabadi, Ojas Prakashbhai Doshi, Nguyen Tran Minh Duc, Emeka W. Dumbili, Senbagam Duraisamy, Oyewole Christopher Durojaiye, Ejemai Eboreime, Ikenna D Ebuenyi, David Edvardsson, Ferry Efendi, Abiodun Egwuenu, Michael Ekholuenetale, Muhammed Elhadi, Yasir Ahmed Mohammed Elhadi, Samar Abd

ElHafeez, Salem Elkahoui, Randa Elsheikh, Chadi Eltaha, Majid Eslami, Fahima Nasrin Eva, Elochukwu Ezenwankwo, Heidar Fadavian, Adeniyi Francis Fagbamigbe, Ayesha Fahim, Mohammad Fareed, Aisha Farhana, Liliana Faria, Andre Faro, Fatemeh Farshad, Folorunso Oludayo Fasina, Davood Fathi, Abdullah H Feroze, Pietro Ferrara, Marta Figueiredo, Florian Fischer, Federica Fogacci, Alina-Ioana Forray, Alberto Freitas, Takeshi Fukumoto, Adam Fusheini, Peter Andras Gaal, Muktar A Gadanya, Dominic Dormenyo Gadeka, Anna D Gage, Márió Gajdács, Balasubramanian Ganesh, Ezhilkugan Ganessane, Chandi Garg, Jacopo Garlasco, Rupesh K Gautam, Stefano Gelibter, Julian Lassiter Gendreau, Gebremariam Wulie Geremew, Kalab Yigermal Gete, Mustafa Ghaderzadeh, Fataneh Ghadirian, Kazem Ghaffari, Leila Ghalichi, Arin Ghamkhar, Ahmad Ghashghaee, Sailaja Ghimire, Vaitsa Giannouli, Artyom Urievich Gil, Syed Abdullah Gilani, Alem Abera Girmay, Davide Golinelli, Melika Golmohammadi, Yitayal Ayalew Goshu, Barbara Niegia Garcia Goulart, Tulasi Govardhan, Ayman Grada, Giovanni Guarducci, Stefano Guicciardi, Sheffali Gulati, Damitha Asanga Gunawardane, Lei Guo, Bhawna Gupta, Rajeev Gupta, Sapna Gupta, Roberth Steven Gutiérrez-Murillo, Annie Haakenstad, Farrokh Habibzadeh, Zahra Hadian, Khalid Rehman Rehman Hakeem, Alex Hakuzimana, Mohamed Hamed, Mohd Anul Haq, Josep Maria Haro, Mohammad Jahid Hasan, S. M. Mahmudul Hasan, Towhid Hasan, Ahmad Shoeb Hashmi, Md Saquib Hasnain, Amr Hassan, Md. Imtaiyaz Hassan, Simon I Hay, Ahmad Hayatu, Reza Mosaddeghi Heris, Thomas Kwadwo Hinneh, Yuta Hiraike, Ramesh Holla, Mehdi Hoseinzadeh, Lubna Hossain, Md Mahbub Hossain, Mohammad Bellal Hossain, Sorin Hostiuc, Junjie Huang, Yongsong Huang, Ayesha Humayun, Aminu Alhassan Ibrahim, Umar Idris Ibrahim, Anel Ibrayeva, Pulwasha Maria Iftikhar, Jibran Ikram, Olayinka Stephen Ilesanmi, Irena M Ilic, Milena D Ilic, Arit Inok, Mujahid Iqbal, Lalu Muhammad Irham, Fahrul Islam, Md Rabiul Islam, Md Sahidul Islam, Nahlah Elkudssiah Ismail, Gaetano Isola, Chidozie Declan Iwu, Jaison Jacob, Louis Jacob, Udeme Samuel Jacob, Shabbar Jaffar, Nader Jahanmehr, Haitham Jahrami, Mihajlo Jakovljevic, Armaan Jamal, Anju James, Jerin James, Masoud Jamshidi, Rajiv Janardhanan, Mu'taman Jarrar, Sathish Kumar Jayapal, Shubha Jayaram, Yovanthi Anurangi Jayasinghe, Prof. Fariborz Sharifian Jazi, Seogsong Jeong, Akaninyene Paul Joseph, Nitin Joseph, Charity Ehimwenma Joshua, Farahnaz Joukar, Jacek Jerzy Jozwiak, Malik E Juweid, Hema Shree K, Adel Kadri, Hossein Samadi Kafil, Ashish Kumar Kakkar, Khalil Kalavani, Feroze Kaliyadan, Rajesh Kamath, Ramat T. Kamorudeen, Jiseung Kang, Kehinde Kazeem Kanmodi, Rami S Kantar, Paschalis Karakasis, Marina Karanikolos, Mohmed Isaqali Karobari, Hamid Reza Khayat Kashani, Faizan Zaffar Kashoo, Nehemia I. Kassa, Nicholas J Kassebaum, Kanica Kaushal, Foad Kazemi, Vikash Ranjan Keshri, Yousef Saleh Khader, Himanshu Khajuria, Asaad Khalid, Anees Ahmed Khalil, Ajmal Khan, Faiz Ullah Khan, Iqra Hamid Khan, MD Sazzad Khan, Mohd Faheem Khan, Muhammad Umer Khan, Ramsha Mushtaq Khan, Serab Khan, Sumaiya Khan, Yusuf Saleem Khan, Zahid Khan, Sameer Uttamaro Khasbage, Khaled Khatab, Haitham Khatatbeh, Moawiah Mohammad Khatatbeh, Bishal Dahal Khatri, Afshin Khazaei, Khalid A Kheirallah, Parisa Khoshvaght, Mahmood Khosrowjerdi, Jinho Kim, Kwanghyun Kim, Min Seo Kim, Adnan Kisa, Megan Knight, Sonali Kochhar, Prakash Babu Kodali, Michail Kokkorakis, Shivakumar KM Kondlahalli, Tapos Kormoker, Oleksii Korzh, Michal Koscik, Irene Akwo Kretchy, Kewal Krishan, Bindu Krishnan, Mohammed Kuddus, Shikha Kukreti, Mukhtar Kulimbet, Chandan Kumar, Dewesh Kumar, Kamal Kumar, Laksh Kumar, Mukesh Kumar, Nitesh Kumar, Pradeep Kumar, Rakesh Kumar, Sandeep Kumar, Satyajit Kundu, Donata Kurpas, Christina Yeni Kustanti, Dian Kusuma, Tezer Kutluk, Ville Kytö, Muhammad Awwal Ladan, Chandrakant Lahariya, Daphne Teck Ching Lai, Hanpeng Lai, Ariane Laplante-Lévesque, Anders O Larsson, Savita Lasrado, Areeba Latif, Sindhura Lakshmi Koulmane Laxminarayana, Eilean Rathinasamy Lazarus, Caterina Ledda, Sergey Vadimovich Lee, Vasileios Leivaditis, Francesco Leonforte, Honghong Li, Shaojie Li, Jue Liu, Xianliang Liu, Zihao Liu, Madeeha Shahzad Lodhi, Niroshan Chandrajith

Lokunarangoda, Jaifred Christian F Lopez, Rafael Lozano, Giancarlo Lucchetti, Miltiadis D Lytras, Ellina Lytvyak, Judit Lám, José Francisco López-Gil, Kevin Sheng-Kai Ma, Monika Machoy-Rakoczy, Samatar Abshir Mahamed, Nozad Hussein Mahmood, Shakeel Ahmed Ibne Mahmood, Kafayat Mahmoud, Ghaseb Naser Makhadmeh, Irsa Fatima Makhdoom, Ahmad Azam Malik, Kamaruddeen Mannethodi, Marjan Mansourian, Tahir Maqbool, Adolfo Martinez-Valle, J Jenifer Florence Mary, Roy Rillera Marzo, Sammer Marzouk, Yasith Mathangasinghe, Fernanda Penido Matozinhos, Khurshid A Mattoo, Suleiman Mayaki, Steven M McPhail, Ravi Mehrotra, Vini Mehta, Tadesse Belayneh Melkie, Ziad Ahmed Memish, Godfred Antony Menezes, Emiru Ayalew Mengistie, Leweyehu Alemaw Mengstie, Atte Meretoja, Tuomo J Meretoja, Giovanni Merlino, Muayad Aghali Merza, Tomislav Mestrovic, Chamila Dinushi Kukulege Mettananda, Sachith Mettananda, Mohamed M. M. Metwally, Sandrine Donfack D Mewoabi, Muhammad Agus Mikrajab, Giuseppe Minervini, Wai-kit Ming, Chaitanya Mittal, Sahar Saeedi Moghaddam, Mona Gamal Mohamed, Nouh Saad Mohamed, Taj Mohammad, Sakineh Mohammad-Alizadeh-Charandabi, Omer Mohammed, Shafiu Mohammed, Yugal Kishore Mohanta, Ali H Mokdad, TJ Robinson T Moncatar, Himel Mondal, Mohammad Ali Moni, Marco Montalti, Mohammad Moradi-Joo, Danilo Farias Morais, Shane Douglas Morrison, Freiser Eceomo Cruz Mosquera, Matías Mrejen, Jibran Sualeh Muhammad, Amartya Mukhopadhyay, JOSHUA KANAABI MULIIRA, Francesk Mulita, Christopher J L Murray, Sani Musa, David Musoke, Ghulam Mustafa, Sathish Muthu, Claude Mambo Muvunyi, Woojae Myung, Chandan S N, Ayoub Nafei, Mobin Naghshbandi, Hiten Naik, Gopal Nambi, Mukesh Kumar Sathya Narayanan, Abdulqadir J Nashwan, Mahmoud Nassar, Samidi Nirasha Kumari Navaratna, Biswa Prakash Nayak, Kinjal Nayak, Javad Nazari, Mojdeh Nazari, Ionut Negoi, Ruxandra Irina Negoi, Alina Gabriela Negru, Nikita Nekliudov, Jan-Walter De Neve, Cuong Tat Nguyen, Hien Thu Nguyen, Huong Lan Thi Nguyen, Tham Thi Nguyen, Trang Nguyen, Yeshambel T Nigatu, Mamoona Noreen, Emira Noumi, Mohammed Noushad, Chisom Adaobi Nri-Ezedi, Fred Nugen, Bogdan Oancea, Akinyemi O D Ofakunrin, Oluwakemi Christie Ogidan, Ayomide Samuel Ogunrinde, Olusegun Olatunji Ojedoyin, Olalekan John Okesanya, Osaretin Christabel Okonji, John Olayemi Okunlola, Musa Kolapo Oladejo, Andrew T Olagunju, Oladotun Victor Olalusi, Abimbola Olaniran, Abdulhakeem Abayomi Olorukooba, Bolajoko Olubukunola Olusanya, Obinna E Onwujekwe, Aksoltan Shyhdurdyevna Oradova, Michal Ordak, Verner N Orish, Atakan Orscelik, Esteban Ortiz-Prado, Godwin Osawaru Osaigbovo, Augustus Osborne, George Nkrumah Osei, Godfred Otchere, Adrian Otoiu, Jerry John Ouner, Amel Ouyahia, Mayowa O Owolabi, Oladayo Ayobami Oyebanji, Kehinde Adewole Oyeniran, Dilber Uzun Ozsahin, Ilker Ozsahin, Jagadish Rao Padubidri, Vijayakumar Palaniswamy, Tamás Palicz, Sujogya Kumar Panda, Bipindra Pandey, Ilias Papadimopoulos, Shahina Pardhan, Romil R Parikh, Chulwoo Park, Roberto Passera, Mitesh Patel, Neel Navinkumar Patel, Shankargouda Patil, Dimitrios Patoulias, Apurba Patra, Snigdha Pattnaik, Uttam Paudel, Shrikant Pawar, Paolo Pedersini, Veincent Christian Filipino Pepito, Maria Odete Pereira, Mario F P Peres, Simone Perna, Michael R Phillips, Zahra Zahid Piracha, Edoardo Pirera, Dimitri Poddighe, Ion Popa, Fabio Porru, Sajjad Pourasghary, Manya Prasad, Akila Prashant, Elton Junio Sady Prates, Harsh Priya, Nicola Riccardo Pugliese, Marcello Di Pumpo, Jagadeesh Puvvula, Xiang Qi, Navid Rabiee, Venkatraman Radhakrishnan, Pracheth Raghuveer, Sajjad Rahimi, Afarin Rahimi-Movaghar, Vafa Rahimi-Movaghar, Mahbubur Rahman, Adarsh Raja, Judah Rajendran, Kairolla Dyusenbayevich Rakhimov, Rafał Rakoczy, Shakthi Kumaran Ramasamy, Diana Fernanda Bejarano Ramirez, Lokesh Rana, Nemanja Rancic, Manjula Rangappa, Magesh Rangasamy, Chythra R Rao, Kumuda Rao, Mithun Rao, Sowmya J Rao, Devarajan Rathish, Jigyasa Rathore, Ramin Ravangard, Renju Ravi, David Laith Rawaf, Salman Rawaf, Lal Rawal, Ramu Rawat, Ayita Ray, Muhammad Liaquat Raza, Bahman Razi, C. Mahony Reategui-Rivera, Ramya Raja Reddy, Bhagwan Narayan Rekadwad, Serge Resnikoff, Stefano Restaino,

Luis Felipe Reyes, Nima Rezaei, Muhammad Riaz, Jennifer Rickard, Leonardo Roever, Ravi Rohilla, Hamid Alinejad Rokny, Kevin T Root, Parvaneh Hamian Roumiani, Sharmistha Roy, Godfrey Mutashambara Rwegerera, Aly M A Saad, Korosh Saber, Siamak Sabour, Bashdar Abuzed Sadee, Hossein Sadr, Umar Saeed, Maryam Saeedi, Muhammad Safiullah, Rajesh Sagar, Indranil Saha, Vaibhav Sahni, Ambika Sahoo, Kirti Sundar Sahu, Mirza Rizwan Sajid, Joseph W Sakshaug, Afeez Abolarinwa Salami, Bukola Salami, Samreen Saleem, Aanuoluwa James Salemcity, Giovanni A Salum, Abdallah M Samy, Elaheh Sanjari, Sathish Sankar, Lucas H C C Santos, Milena M Santric-Milicevic, Malik Adebayo Sanusi, Yaser Sarikhani, Mohammad Sarmadi, Gargi Sachin Sarode, Sachin C Sarode, Michele Sassano, Maheswar Satpathy, Amar Saurabh, Sangeeta Gopal Saxena, Yaser Sayadi, Soraya Seedat, Siddharthan Selvaraj, Yuliya Semenova, Subramanian Senthilkumaran, Sadaf G Sepanlou, Francesco Sessa, Yashendra Sethi, Abubakar Sha'aban, Shipraa Arpan Shah, Sweni Shah, Muhammad Shahab, Samiah Shahid, Endrit Shahini, Farshad Shahkarami, Ali Shahriari, Moyad Jamal Shahwan, Babar Tasneem Shaikh, Ali Shakerimoghaddam, Alfiya Shamsutdinova, Dan Shan, Mohd Shanawaz, Nadim Sharif, Fatemeh Saheb Sharif-Askari, Amin Sharifan, Avimanu Sharma, Jyoti Sharma, Kamal Sharma, Kamlesh Sharma, Manoj Sharma, Vishal Sharma, Ramzi Shawahna, Anas Shehadeh, Somia Shehzadi, Rekha Raghuveer Shenoy, Suchitra M Shenoy, Samendra P Sherchan, Mahabalesh Shetty, Pavanchand Shetty, Premalatha K Shetty, Muhammad J. A. Shiddiky, Md Monir Hossain Shimul, Priyanka Arun Shirali, SHIRINDOKHT SHIRAZI, Aminu Shittu, Sina Shool, Seyed Afshin Shorofi, Sunil Shrestha, Suleiman Adeiza Shuaibu, Negussie Boti Sidamo, Emmanuel Edwar Siddig, Arif J Siddiqui, Gustavo Correia Basto da Silva, Padam Prasad Simkhada, David Larbi Simpong, Colin R Simpson, Abhinav Singh, Bhoopendra Singh, Brij Pal Singh, Harmanjit Singh, Jasvinder A Singh, Jawahar Singh, Shilpy Singh, Siddharth Singh, Mukesh Kumar Sinha, Ratnesh Sinha, Gisha Sivan, Natia Skhvitaridze, Valentin Yurievich Skryabin, Mejdi Snoussi, Farrukh Sobia, César Agostinis Sobrinho, Solikhah Solikhah, Jukun Song, Michael Spartalis, Lokasani Vijaya Sree, Chandrashekhar T Sreeramareddy, Shyamkumar Sriram, Panagiotis Stachteas, Anca Pantea Stoian, Brendon Stubbs, Peter Stubbs, Chen-Yang Su, Omer Subasi, Vetriselvan Subramaniyan, Aswin Sugunan, Mahwish Suhaib, Oksana Sulaieva, Mark J M Sullman, Yan Sun, Thanigaivel Sundaram, David Sunkersing, Lukasz Szarpak, Seyed-Amir Tabatabaeizadeh, Shima Tabatabai, Ramin Tabibi, Zanan Mohammed-Ameen Taha, Mircea Tampa, Jianye Tan, Ker-Kan Tan, Shenglan Tang, Akhenaten Benjamin Siankam Tankwanchi, Anika Tasnim, Nathan Y Tat, Vivian Y Tat, Wegen Beyene Tesfamariam, Azimeraw Arega Tesfu, Yazan Al Thaher, Pugazhenthan Thangaraju, Ismaeel Tharwat, Jeevarathinam Thirumalai, Vikas Kumar Tiwari, Santo Imanuel Tonapa, Roman Topor-Madry, Stephanie M Topp, Hamidreza Pazoki Toroudi, Marcos Roberto Tovani-Palone, Thang Huu Tran, Domenico Trico, Gary Tse, Vasilis-Spyridon Tseriotis, Evangelia Tsermpini, Samiun Nazrin Bente Kamal Tune, Shehu Salihu Umar, Era Upadhyay, Jibrin Sammani Usman, Hande Uzunçıbuk, Pascual R Valdez, Sven Vanneste, Joe Varghese, Santosh Varughese, Tommi Juhani Vasankari, Anoop Velayudhan, Balachandar Vellingiri, Narayanaswamy Venketasubramanian, Nicollas Mozart Vieira, David Villarreal-Zegarra, Tuan Vinh, Luciano Magalhães Vitorino, Vasily Vlassov, Simona Ruxandra Volovat, Linh Vu, Yasir Waheed, Ethan Waisberg, Eric Lee Wan, Jin-Yi Wan, Haozhen Wang, Shaopan Wang, Shu Wang, Wanzhou Wang, Wei Wang, Xuequan Wang, Yan Wang, Yanzhong Wang, Youxin Wang, Mary Njeri Wanjau, Paul R Ward, Wesley Warriner, Toyiba Hiyaru Wassie, Anggi Lukman Wicaksana, Nuwan Darshana Wickramasinghe, Samuel Wiebe, Axel Walter Wolf, Saba Yahoo, Haibo Yang, Yuichiro Yano, Haiqiang Yao, Haya Khader Ahmad Yasin, Sanni Yaya, Kuanysh A. Yergaliyev, Saber Yezli, Yulai Yin, Dong Keon Yon, Naohiro Yonemoto, Kimia Mozahheb Yousefi, Hairui Yu, Yong Yu, Quan Yuan, Ghazala Yunus, Omar Ali Mohammed Al Zaabi, Arezou Hashem Zadeh, Nelson Zamora, Aurora Zanghì, Kourosh Zarea, Ahmad Zarei, Michael Zastrozhin, Saad Zbiri, Mohammed G M Zeariya, Zahra

Zeinali, Ruba A Zenati, Julio Min Fei Zhang, Yingxi Zhao, Jinxin Zheng, Anthony Zhong, Bin Zhu, Zhaohua Zhu, Mohamed Ali Zoromba, Mohammad Ahmmad Mahmoud Al Zoubi, Tariq Al Zoubi, Alimuddin Zumla, Ahed H Zyoud, Sa'ed H Zyoud, Shaher H Zyoud, Simon Øverland, Simona Cătălina Ștefan

Managing the estimation or publications process

Annie Haakenstad, Simon I Hay, Ali H Mokdad, Christopher J L Murray, Maja Pasovic
